# The Catalytic Asymmetric Mukaiyama–Michael Reaction of Silyl Ketene Acetals with Cyclic Enones: Short Routes to Jasmonates

**DOI:** 10.1021/jacs.5c20804

**Published:** 2026-02-14

**Authors:** Ruigang Xu, Hui Zhou, Han Yong Bae, Vijay N. Wakchaure, Lorenzo Baldinelli, Isaac F. Leach, Giovanni Bistoni, Philip Kraft, Benjamin List

**Affiliations:** † Max-Planck-Institut für Kohlenforschung, Kaiser-Wilhelm-Platz 1, 45470 Mülheim an der Ruhr, Germany; ‡ College of Chemistry, 12446 Central China Normal University, Wuhan 430079, P. R. China; § Department of Chemistry, 35017 Sungkyunkwan University, Suwon 16419, Republic of Korea; ∥ Department of Chemistry, Biology, and Biotechnology, 201791 University of Perugia, 06123 Perugia, Italy; ⊥ 10784 Symrise AG, S&C Global Innovation Fragrances, Building D 209, Mühlenfeldstr. 1, 37603 Holzminden, Germany

## Abstract

A silylium imidodiphosphorimidate (IDPi) Lewis acid catalyst enables a broadly applicable organocatalytic asymmetric Mukaiyama–Michael addition of moderately electrophilic cycloenones with enol silanes, affording 1,4-adducts in up to 98% yield and >99:1 e.r. At 0.05 mol % catalyst loading, the reaction scales to 167 g of product with 96% catalyst recovery. The method accommodates a wide range of enones and silylated nucleophiles, allowing streamlined access to key jasmonates and related valuable targets. Computational studies elucidated the origin of the enantioselectivity in this reaction. This platform thus provides a versatile entry to structurally complex chiral scaffolds with direct relevance to plant signaling, fragrance chemistry, aromatherapy, and pharmaceutical sciences.

## Graphical abstract

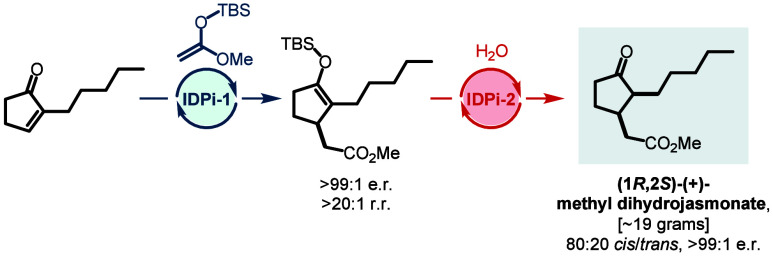

The Mukaiyama–Michael addition of enol silanes to α,β-unsaturated carbonyl compounds constitutes a fundamental and widely employed method for C–C bond formation. Since Mukaiyama’s seminal enantioselective addition of thioester-derived enol silanes to enones using a chiral titanium catalyst,^1^ numerous catalytic asymmetric variants have been developed, particularly via iminium-ion and Lewis acid activation.^2–5^ In metal-catalyzed systems, high enantioselectivity is typically achieved when the acceptor can chelate bidentately to the metal center.^6^ This chelation preferentially aligns the substrate to expose one reactive enantioface within the chiral environment, while significantly lowering its LUMO energy, thereby facilitating selective nucleophilic attack (Figure 1A). In contrast, nonchelating, weakly coordinating α,β-unsaturated acceptors, particularly cyclic enones and α-alkyl-substituted cycloenones,^7^ typically display moderate to poor reactivity and limited stereocontrol under metal-based catalytic conditions. Moreover, substitution at the α- or β-position markedly decreases the electrophilicity, as indicated by the Mayr electrophilicity parameter *E* (Figure 1B). The retarding effect of α- and β-substituted cycloenones can be rationalized by the combined effects of steric constraints around the Michael acceptor and the electron inductive effect of the substituent.^7a,7b^ Consequently, a general enantioselective Mukaiyama–Michael addition to α-substituted cyclic enones remains elusive.

**Figure 1:**
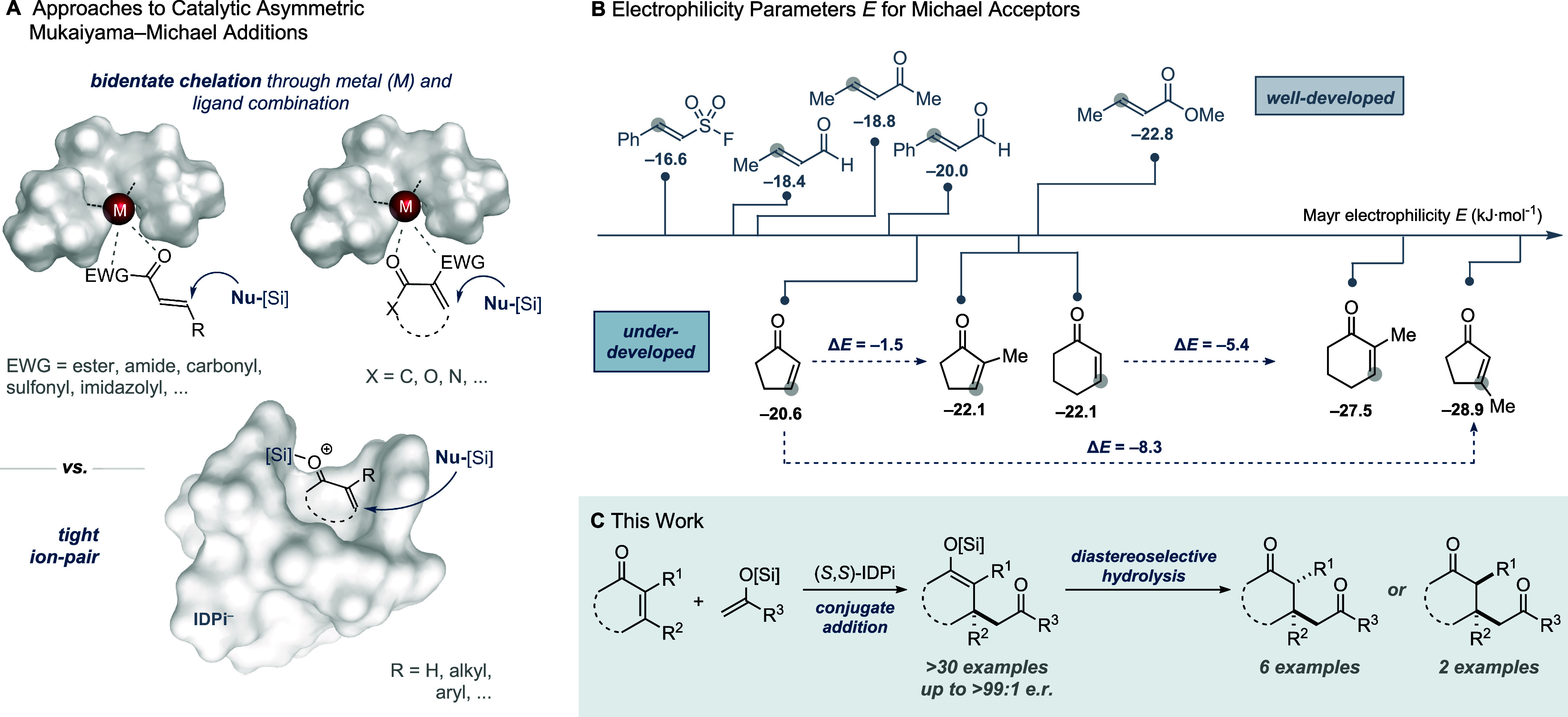
(A) Comparison of catalytic modes. (B) Electrophilicity of acyclic and cyclic Michael acceptors. (C) IDPi-catalyzed Mukaiyama–Michael addition of cyclic enones followed by protodesilylation to access stereochemically complex frameworks.

In 2018 we disclosed a silylium imidodiphosphorimidate (IDPi) Lewis acid catalyzed Mukaiyama–Michael addition of silyl ketene acetals to α,β-unsaturated esters.^4^ In this system, a silylium species is paired with a chiral counteranion to form a tight, well-organized ion pair that engages the acceptor, obviates the need for chelation, and enables precise enantiocontrol within a confined chiral pocket.^9^ Given that a general, catalytic asymmetric Mukaiyama–Michael addition to cyclic enones would represent a powerful tool in natural product synthesis, we initiated a research program toward this goal.^10^ Here we report the fruition of these investigations with a silylium IDPi-catalyzed and broadly applicable and scalable Mukaiyama–Michael reaction followed by a diastereoselective protodesilylation sequence (Figure 1C). Notably, the enol silane intermediate enables stereodivergent hydrolysis, providing access to either diastereomer under kinetic or thermodynamic control.

We began our investigation using 2-pentylcyclopentenone **1c** and commercially available silyl ketene acetal (SKA) **2a** under cryogenic conditions. Moderately acidic chiral Brønsted acids, including CPA,^11,12^ IDP,^13^ *i*IDP^14,15^ families, failed to promote productive conjugate addition, whereas a strongly acidic catalyst (DSI)^16^ favored silicon–hydrogen exchange rather than C–C bond formation (see Supporting Information (SI)). IDPi **4a**, characterized by its high acidity and an exceptionally confined chiral microenvironment^17–23^ enabled quantitative formation of 1,4-adduct **3c** with excellent regioselectivity (1,4/1,2 >20:1) and a moderate enantiomeric ratio of 75:25 (Table 1, entry 1). Among the IDPi variants evaluated (entries 2–8), catalyst **4c** (Ar = 3-^*t*^Bu-C_6_H_4_) provided superior enantiocontrol, affording the product with up to 94:6 e.r. (entry 8). Ultimately, IDPi **4f** bearing a *p*-^*t*^Bu phenyl substituent at the 3,3′-positions and a pentafluorophenylsulfonyl core, was identified as optimal. It not only induced a reversal of enantiofacial selectivity but also delivered the 1,4-adduct with excellent regio- and enantioselectivity (1:99 e.r.) in nearly quantitative yield (entry 11). On this basis, we investigated how the steric properties of different silyl groups in the SKA affect the reactivity, stereo- and chemoselectivity (see SI).

**Table 1:**
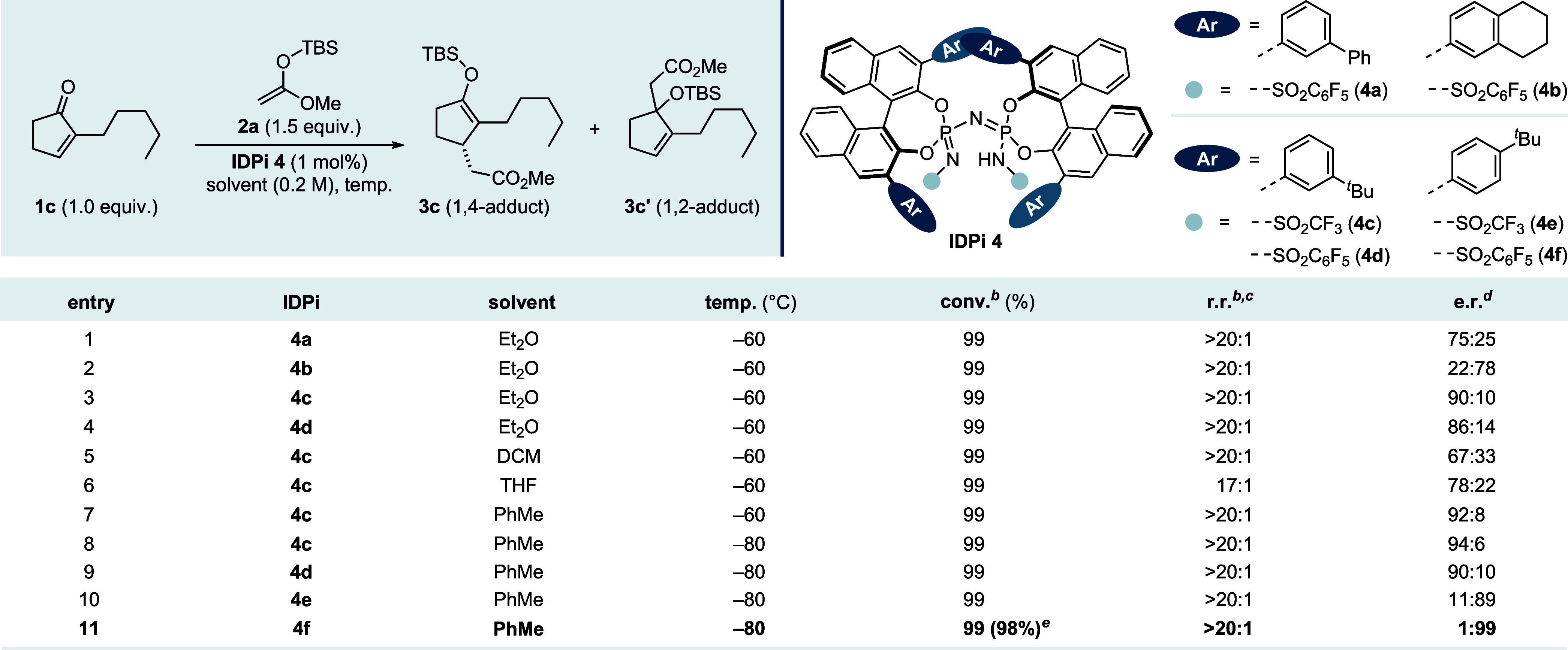
Reaction Development

a Reactions were conducted with enone **1c** (0.05 mmol), silyl ketene acetal **2a** (1.5 equiv), and IDPi **4** (1 mol %) in solvent (0.2 M) for 18 h at the indicated temperature.

b Conversions (conv.) and regioisomeric ratios (r.r.) to **3** were determined by ^1^H NMR of the crude reaction mixture using CH_2_Br_2_ as an internal standard.

c 1,4-Adduct formed as the major product.

d Enantiomeric ratios (e.r.) were measured by HPLC.

e Yield for the isolated product.

With the optimal reaction conditions in hand, we explored the generality of the conjugate addition (Figure 2A). Cycloenones bearing alkyl, alkenyl, alkynyl, and aryl substituents reacted smoothly to provide the corresponding 1,4-adducts in high yields and typically around 95:5 e.r. (**3a**–**q**). A heteroaryl example was also tolerated, furnishing product **3r** with good enantioselectivity. Notably, 3-methyl cyclopentenone also proved to be a competent substrate, affording enol silane **3s**, featuring a quaternary stereocenter, in 87% yield with high regio- and enantioselectivity. However, substituting the methyl group with an *n*-butyl moiety resulted in a preference for the corresponding 1,2-addition (1,2/1,4 = 62:38; see SI). Furthermore, extension to six- and seven-membered cycloenones afforded products **3t**–**w** in moderate yields with diminished regio- and enantioselectivity. Remarkably, heterocyclic enones containing nitrogen, oxygen or sulfur were well tolerated, delivering products **3x**–**z** in good yields and high enantioselectivities (95:5 to 98:2 e.r.). These results demonstrate the broad scope and versatility of the IDPi-catalyzed asymmetric conjugate addition for constructing structurally and stereochemically complex products, and further underscore the potential of silylium IDPi Lewis acid catalysis as a powerful platform for enantioselective synthesis. We next evaluated both silyl ketene acetals and enol silanes (Figure 2B). Under the standard conditions, sterically hindered SKA **2b**, bearing two methyl substituents, showed poor conversion. Increasing the reaction temperature to −50 °C effectively addressed this limitation, affording the desired 1,4-adduct **3aa** in 84% yield with excellent enantioselectivity (93:7 e.r.).

**Figure 2:**
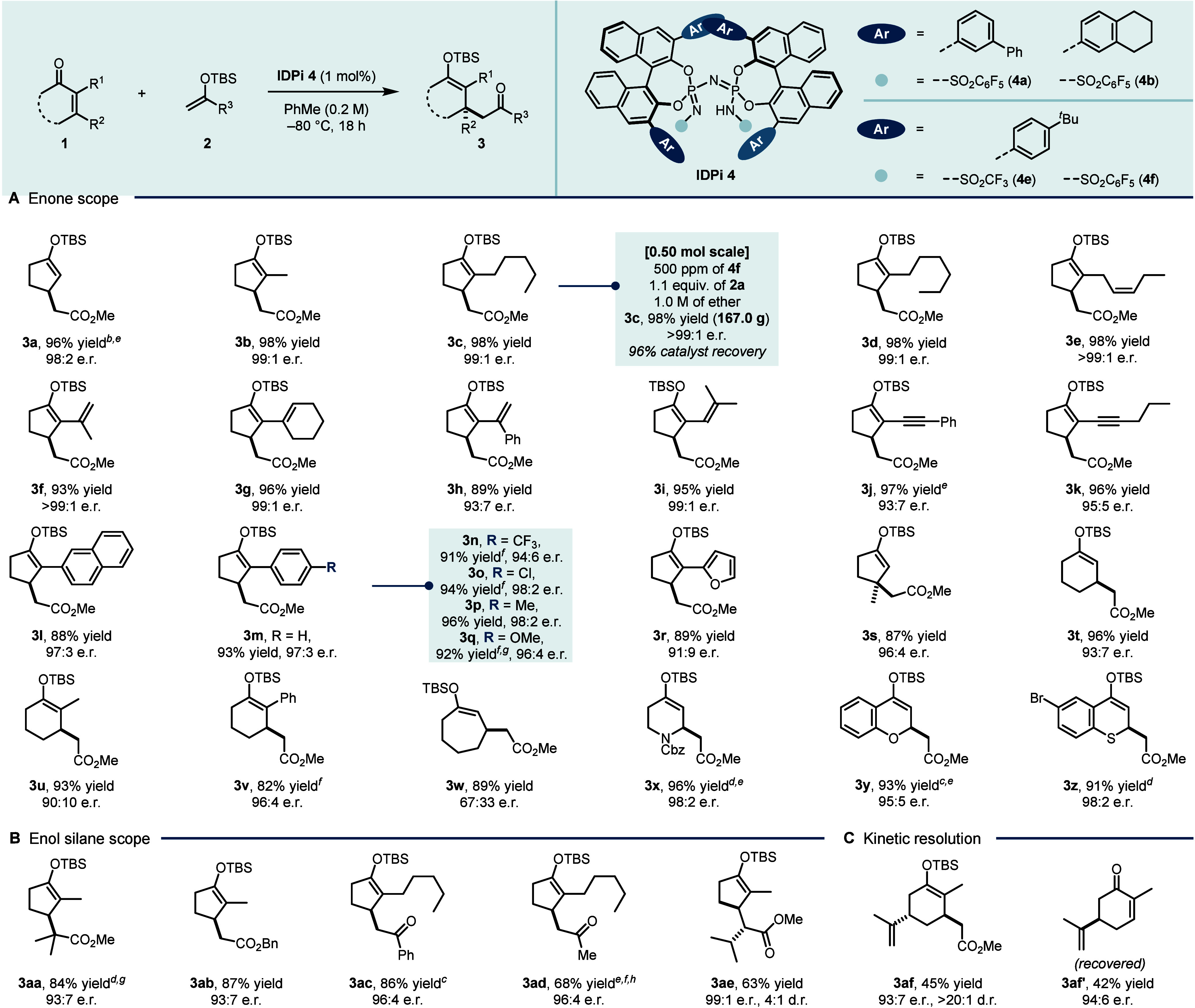
Substrate scope.^*a*^ ^*a*^Unless otherwise stated, reactions were carried out with enones **1** (0.10 mmol, 1.0 equiv), silyl nucleophiles **2** (0.15 mmol, 1.5 equiv), and IDPi **4f** (1 mol %) in PhMe (0.2 M) at −80 °C for 18 h. Yields are reported as isolated yields and enantiomeric ratios (e.r.) were determined by HPLC. ^*b*^IDPi **4b** was used. ^*c*^IDPi **4e** was used. ^*d*^IDPi **4a** was used. ^*e*^Et_2_O instead of PhMe. ^*f*^Reaction over 36 h. ^*g*^Performed at −50 °C. ^*h*^3.0 equiv of silyl enol ether was used.

Despite this success, certain combinations of enone electrophiles and silylated nucleophiles proved challenging, showing diminished reactivity or leading to competing side processes such as silicon–hydrogen exchange or undesired 1,2-addition (see SI). Notably, *rac*-carvone underwent a kinetic resolution with catalyst **4f** to furnish the corresponding 1,4-adduct **3af** with high diastereo- and enantioselectivity. The unreacted enantiomer, (*R*)-carvone **3af′**, was recovered in 42% yield with 94:6 e.r., highlighting the remarkable potential of the methodology also for kinetic resolution.

To demonstrate the synthetic practicality and scalability of our approach, the conjugate addition was performed on a hectogram scale. Under slightly modified conditions, product **3c** was isolated by distillation in high yield (167.0 g, 98% yield) and excellent enantiopurity, with the catalyst recovered in 96% yield from the organic phase after chromatographic purification and acidification, further highlighting the process’s potential industrial relevance.

We subsequently applied our newly established methodology to the *trans*-selective protodesilylation of Michael adducts, enabling streamlined access to bioactive pharmaceuticals and perfumery ingredients^8^ without the need for intermediate chromatographic purification. By carefully tuning the acidity and steric profile of the proton source to each silylated adduct, we successfully developed a thermodynamically controlled *trans*-selective protonation pathway. Thus, treatment of silyl ether **3c** with 10 vol% aqueous HCl in THF at 0 °C selectively furnished *trans*-jasmonates, (1*R*,2*R*)-(−)-methyl dihydrojasmonate (**6a**) with a floral, sweet jasminic odor in agreement to ref.,^8f^ (2*R*,3*R*)-configured Magnolia Ketone (**6b**) with a fruity-fresh floral nuances jasmine odor and in contrast to ref.^8e^ additional metallic and an animalic touch, and finally (1*R*,2*R*)-(−)-(*Z*)-methyl jasmonate (**6c**) with only a weak green-floral odor and very faint jasmine accents—the latter in contrast to the literature,^24^ in which it was described very weak to odorless with a 240 ppb threshold. These products were obtained in good to high yields with moderate to excellent diastereo- and enantioselectivities (Figure 3A). Subsequent LiOH-mediated hydrolysis in THF/H_2_O smoothly transformed methyl ester **6c** to the corresponding acid **8** while retaining its enantiomeric purity. Amidation of methyl dihydrojasmonate **6a** provided derivative **7**, whose absolute configuration was confirmed by single-crystal X-ray diffraction. Furthermore, JA-Ile-lactone, a biologically active regulator of plant growth, can be accessed from **6c** in a few steps.^25^ For substrates bearing electron-rich aryl substituents, the milder HCl protocol proved insufficient to achieve complete hydrolysis. Instead, treatment with TsOH·H_2_O enabled clean protodesilylation, exclusively affording *trans*-products **6d**–**f** with diastereomeric ratios ranging from 12:1 to >20:1.

**Figure 3:**
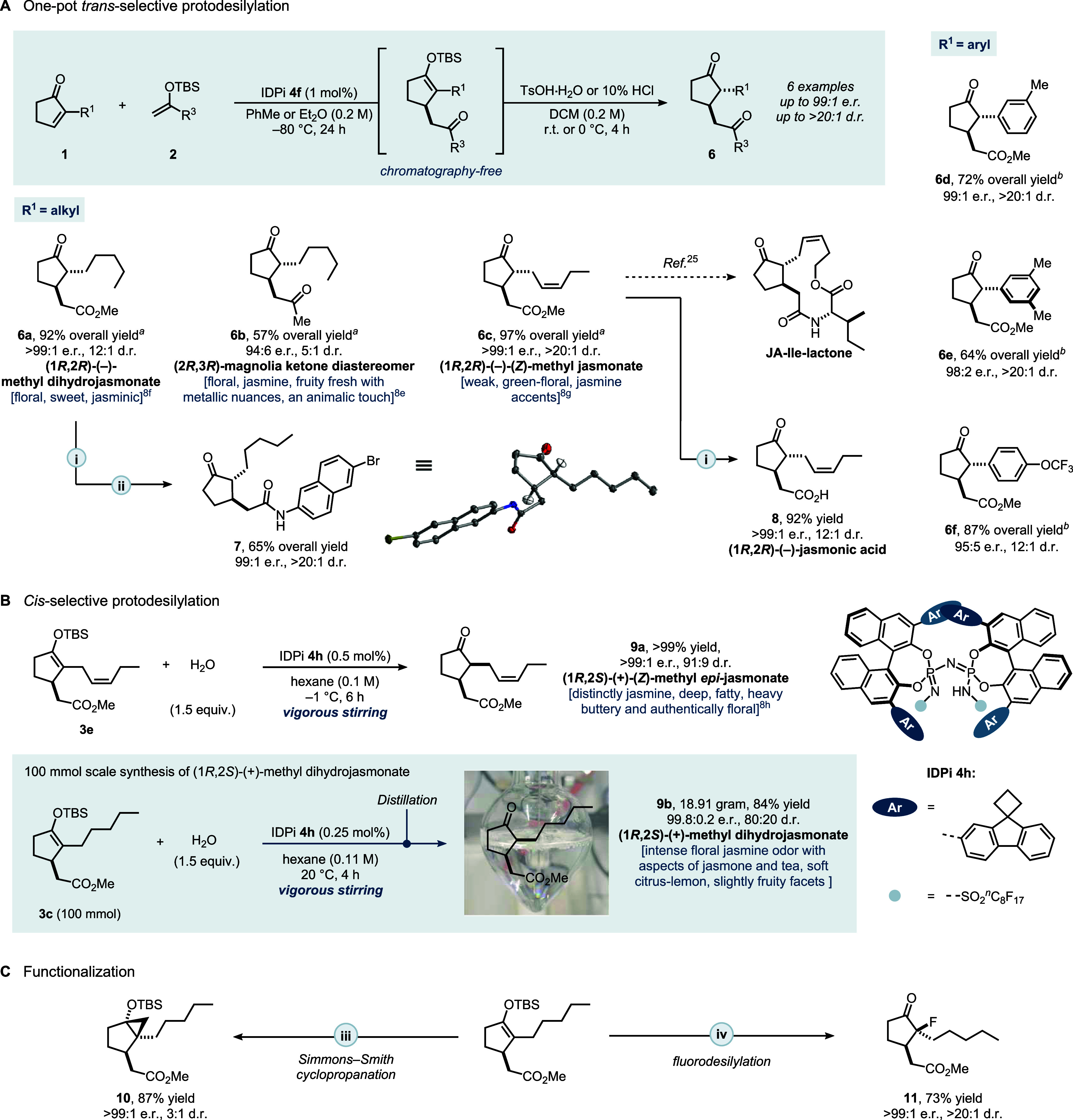
Synthesis of bioactive drugs and representative fragrance molecules. (A) *trans*-Selective protonation. (B) *cis*-Selective protonation. (C) Derivatizations. ^*a*^10% HCl was used. ^*b*^TsOH·H_2_O was used. Reagents and conditions: (i) LiOH (2.5 equiv), THF/H_2_O (*v*/*v* = 1/1), rt, 2 h. (ii) 6-bromonaphthalen-2-amine (1.0 equiv), EDCI (1.5 equiv), triethylamine (1.6 equiv), DCM (0.1 M), rt, 12 h. (iii) diethylzinc (6.0 equiv), diiodomethane (10.0 equiv), DCM (0.1 M), 0 °C, 6 h. (iv) Selectfluor (1.1 equiv), CH_3_CN (0.1 M), 0 °C, 4 h.

In parallel, we investigated the kinetically controlled *cis*- selective protodesilylation. Two commercially valuable perfumery ingredients, (1*R*,2*S*)-(+)-methyl dihydrojasmonate (**9b**) and (1*R*,2*S*)-(+)-(*Z*)-methyl *epi*-jasmonate (**9a**), the olfactory properties of which critically depend on the absolute and relative stereochemistry,^24^ were targeted by protonation of enol silanes **3c** and **3e**, respectively (Figure 3B). An extensive catalyst screening identified IDPi **4h** as optimal (see SI for details), with water serving as an effective proton source. Under these conditions, enol silane **3e** cleanly converted to *cis*-product **9a** in excellent yield with high diastereomeric ratio. Control experiments further indicated that the stereochemical configuration of **4h** is the primary determinant of the diastereoselectivity (see SI). Notably, scale-up to a decagram synthesis of (1*R*,2*S*)-(+)-methyl dihydrojasmonate (**9b**) was achieved in a mechanical agitation perfumery reactor (2000 rpm stirring) without any loss in selectivity. Following careful workup and fractional distillation, the desired product (**9b**) was isolated in high yield with an 80:20 d.r. and >99:1 e.r. Interestingly, the undersired 20% of the *trans*-configured diastereomer (1*R*,2*S*)-(−)-**9b** did not deteriorate the olfactory properties of the mixture which was found to possess an intense floral jasmine odor with aspects of jasmone and tea, and soft citrus-lemon as well as slightly fruity facets. This particular mixture was even found superior to the pure (1*R*,2*R*)-(+)-enantiomer. Overall, these results demonstrate that, by judicious choice of catalyst and proton source, our methodology can be switched between thermodynamic and kinetic protonation pathways, enabling stereodivergent access to both perfumery ingredients and bioactive compounds under operationally simple and scalable conditions.

To further diversify the enantioenriched enol silanes, downstream transformations were demonstrated (Figure 3C). Simmons–Smith cyclopropanation of **3c** furnished bicyclo[3.1.0]­hexane **10** (retaining e.r.), and fluorodesilylation with Selectfluor delivered α-fluorocyclopentanone **11** in good yield with high stereoselectivity.

We next turned our attention to the origin of the enantioselectivity. For the IDPi catalysts, formation of the chiral ion-pair intermediate (CIP) is endothermic, whereas the ensuing C–C bond forming step proceeds with virtually no barrier (see SI for details). We therefore optimized and analyzed the CIPs of **4d** and **4f** with the 2-pentylcyclopentenone–TBS cation to rationalize enantiodiscrimination and the meta/para *tert*-butyl effect (Figure 4A). Steric maps generated using Cavallo’s web tool^26^ reveal that, in each CIP, attack on the less hindered face selectively furnishes the major enantiomer (Figure 4B), in full agreement with the experimental outcomes. Electrostatic analyses further indicate substantial catalyst–substrate interactions within the ion pair (Figure S11). In addition, utilizing our atomic decomposition of London dispersion energy method, ADLD (D4),^27,28^ we uncovered that the pentyl chain plays a larger stabilizing role in the formation of the CIP in **4f** as compared to **4d** (Figure 4C). The role of the alkyl chain can be quantified by taking the difference between its atomic London dispersion contributions in catalyst **4f** vs. **4d** (−2.8 kcal·mol^–1^). Red atoms indicate where **4f** provides stronger dispersion stabilization, and the blue where it is weaker. This enhanced dispersion stabilization is consistent with the higher enantioselectivity observed for **4f** (>99:1 e.r.) relative to **4d** (10:90 e.r.).

**Figure 4:**
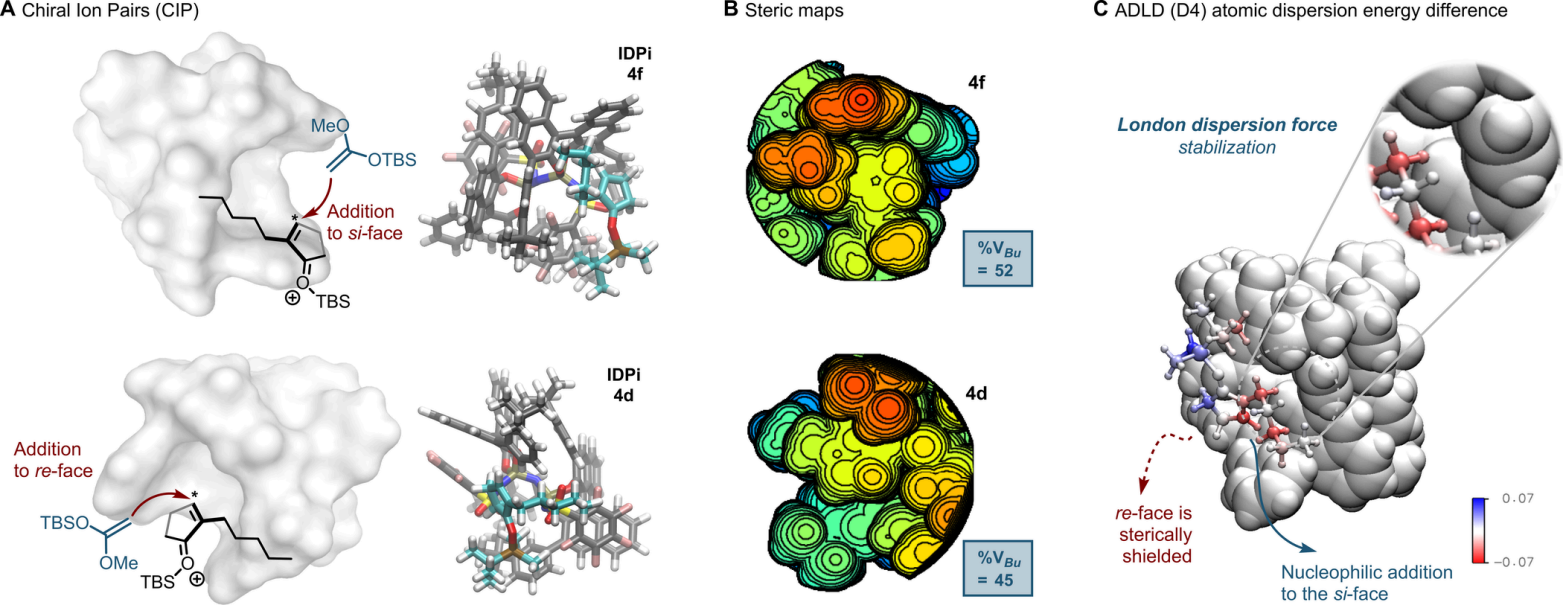
(A) CIPs of **4f** and **4d**: schematic representation (left, reactive carbon*) and DFT-optimized structure (right). The so-selected conformers were reoptimized at PBE-D3­(BJ)/def2-SVP­(-f) + CPCM­(toluene) level of theory. (B) Calculated steric maps of **4f** and **4d** with enone substrates. (C) ADLD (D4) atomic dispersion energy difference (**4f** vs. **4d**) mapped onto the **4f** CIP, using standard (B3LYP-D4) parameters. Catalyst structure shown by its van der Waals radii.

We have developed a scalable catalytic platform for the asymmetric Mukaiyama–Michael reaction of moderately electrophilic cycloenones. The reaction is enabled by chiral silylium ion-based Lewis acids, affording 1,4-adducts with high diastereo- and enantioselectivity across a broad range of substrates and enol silanes. Careful olfactory analysis of the jasmonate products allowed to confirm and complement the literature data on this commercially important class of odorants, and computational studies highlight the pivotal influence of the chiral ion pair on enantioinduction. This methodology offers a versatile route to structurally complex chiral scaffolds, with direct applicability to Haute Parfumerie and bioactive pharmaceuticals, even potential application in aromatherapy.^29^

## Supplementary Material



## Notes

Additional experimental details and analytical data for all new compounds, computational details, crystallographic data for compound **7** (PDF)

## References

[1] Narasaka K., Soai K., Aikawa Y., Mukaiyama T. (1976). The Michael
Reaction of Silyl Enol Ethers with *α*,*β*-Unsaturated Ketones and Acetals in the Presence
of Titanium Tetraalkoxide and Titanium Tetrachloride. Bull. Chem. Soc. Jpn..

[2] Brown S. P., Goodwin N. C., MacMillan D. W. C. (2003). The First Enantioselective Organocatalytic
Mukaiyama-Michael Reaction: A Direct Method for the Synthesis of Enantioenriched
γ-Butenolide Architecture. J. Am. Chem.
Soc..

[3] Frias M., Mas-Ballesté R., Arias S., Alvarado C., Alemán J. (2017). Asymmetric
Synthesis of Rauhut-Currier Type Products
by A Regioselective Mukaiyama Reaction under Bifunctional Catalysis. J. Am. Chem. Soc..

[4] Gatzenmeier T., Kaib P. S. J., Lingnau J. B., Goddard R., List B. (2018). The Catalytic
Asymmetric Mukaiyama-Michael Reaction of Silyl Ketene Acetals with *α*,*β*-Unsaturated Methyl Esters. Angew. Chem., Int. Ed..

[5] Evans D. A., Rovis T., Kozlowski M. C., Downey C. W., Tedrow J. S. (2000). Enantioselective
Lewis Acid Catalyzed Michael Reactions of Alkylidene Malonates. Catalysis
by *C*
_2_-Symmetric Bis­(oxazoline) Copper­(II)
Complexes in the Synthesis of Chiral, Differentiated Glutarate Esters. J. Am. Chem. Soc..

[6] Waulters-Kline Q., Gangadurai C., Thorat S. S., Renner A. C., Sibi M. P. (2025). Mukaiyama-Michael
Reaction: Enantioselective Strategies and Applications in Total Synthesis. Org. Biomol. Chem..

[7] Mayer R. J., Allihn P. W. A., Hampel N., Mayer P., Sieber S. A., Ofial A. R. (2021). Electrophilic Reactivities of Cyclic
Enones and *α*,*β*-Unsaturated
Lactones. Chem. Sci..

[8] Kraft P., Bajgrowicz J. A., Denis C., Fráter G. (2000). Odds and Trends:
Recent Developments in the Chemistry of Odorants. Angew. Chem., Int. Ed..

[9] Schreyer L., Properzi R., List B. (2019). IDPi Catalysis. Angew. Chem., Int. Ed..

[10] Ouyang J., Bae H., Jordi S., Dao Q. M., Dossenbach S., Dehn S., Lingnau J. B., Kanta De C., Kraft P., List B. (2021). The Smelling Principle of Vetiver Oil, Unveiled by Chemical Synthesis. Angew. Chem., Int. Ed..

[11] Parmar D., Sugiono E., Raja S., Rueping M. (2014). Complete Field Guide
to Asymmetric BINOL-Phosphate Derived Brønsted Acid and Metal
Catalysis: History and Classification by Mode of Activation; Brønsted
Acidity, Hydrogen Bonding, Ion Pairing, and Metal Phosphates. Chem. Rev..

[12] Akiyama T. (2007). Stronger Brønsted
Acids. Chem. Rev..

[13] Čorić I., List B. (2012). Asymmetric Spiroacetalization
Catalysed by Confined Brønsted
Acids. Nature.

[14] Liu L. P., Kaib P. S. J., Tap A., List B. (2016). A General Catalytic
Asymmetric Prins Cyclization. J. Am. Chem. Soc..

[15] Grimm J. A. A., Zhou H., Properzi R., Leutzsch M., Bistoni G., Nienhaus J., List B. (2023). Catalytic
Asymmetric Synthesis of
Cannabinoids and Menthol from Neral. Nature.

[16] James T., van Gemmeren M., List B. (2015). Development and Applications of Disulfonimides
in Enantioselective Organo-catalysis. Chem.
Rev..

[17] Zhou H., Zhou Y., Bae H. Y., Leutzsch M., Li Y., De C. K., Cheng G.-J., List B. (2022). Organocatalytic Stereoselective
Cyanosilylation of Small Ketones. Nature.

[18] Singh V. K., Zhu C., De C. K., Leutzsch M., Baldinelli L., Mitra R., Bistoni G., List B. (2023). Taming Secondary Benzylic
Cations in Catalytic Asymmetric S_N_1 Reactions. Science.

[19] Wakchaure V. N., DeSnoo W., Laconsay C. J., Leutzsch M., Tsuji N., Tantillo D. J., List B. (2024). Catalytic Asymmetric Cationic Shifts
of Aliphatic Hydrocarbons. Nature.

[20] Zheng T., Nöthling N., Wang Z., Mitschke B., Leutzsch M., List B. (2024). A Solid Noncovalent
Organic Double-Helix Framework Catalyzes Asymmetric
[6 + 4] Cycloaddition. Science.

[21] Luo N., Turberg M., Leutzsch M., Mitschke B., Brunen S., Wakchaure V. N., Nöthling N., Schelwies M., Pelzer R., List B. (2024). The Catalytic
Asymmetric Polyene
Cyclization of Homofarnesol to Ambrox. Nature.

[22] Raut R. K., Matsutani S., Shi F., Kataoka S., Poje M., Mitschke B., Maeda S., Tsuji N., List B. (2024). Catalytic
Asymmetric Fragmentation of Cyclopropanes. Science.

[23] Guillén M., Leutzsch M., List B. (2024). Catalytic
Asymmetric Cycloaddition
of Olefins with In Situ Generated *N*-Boc-Formaldimine. J. Am. Chem. Soc..

[24] Chapuis C. (2012). The Jubilee
of Methyl Jasmonate and Hedione®. Helv.
Chim. Acta.

[25] Lin S. B., Dong Y. N., Li X. W., Xing Y. X., Liu M. M., Sun X. L. (2020). JA-Ile-Macrolactone
5b Induces Tea Plant (Camellia
sinensis) Resistance to Both Herbivore Ectropis obliqua and Pathogen
Colletotrichum camelliae. Int. J. Mol. Sci..

[26] Falivene L., Credendino R., Poater A., Petta A., Serra L., Oliva R., Scarano V., Cavallo L. (2016). SambVca 2. A Web Tool
for Analyzing Catalytic Pockets with Topographic Steric Maps. Organometallics.

[27] Baldinelli L., De Angelis F., Bistoni G. (2024). Unraveling Atomic Contributions
to
the London Dispersion Energy: Insights into Molecular Recognition
and Reactivity. J. Chem. Theory Comput..

[28] Regni G., Baldinelli L., Bistoni G. (2025). A Quantum Chemical Method for Dissecting
London Dispersion Energy into Atomic Building Blocks. ACS Cent. Sci..

[29] Ashraf, K. ; Rasool, U. ; Younis, H. ; Mehmood, R. ; Mehboob, S. ; Batool, M. ; Fatima, M. ; Iqbal, S. ; Habiba, U. E. ; Ahmad, M. Aromatherapy and Jasmine Oil Inhalation in Improving Brain Activities. In Complementary and Alternative Medicine: Essential oils; Zafar, M. A. ; Abbas, R. Z. ; Imran, M. ; Tahir, S. ; Qamar, W. , Eds.; Unique Scientific Publishers: Faisalabad, Pakistan. 2024; pp 43–53. 10.47278/book.CAM/2024.293.

