# Supplementary material for: The Catalytic Asymmetric Mukaiyama–Michael Reaction of Silyl Ketene Acetals with Cyclic Enones: Short Routes to Jasmonates
Source: J Am Chem Soc. 2026 Feb 14;148(7):6779–85. doi: 10.1021/jacs.5c20804 (PMC12951435; doi:10.1021/jacs.5c20804)
Supplement: Supplementary file 1 [file ja5c20804_si_001.pdf]

The Catalytic Asymmetric Mukaiyama−Michael Reaction of Silyl Ketene Acetals with Cyclic Enones: Short Routes to Jasmonates

(Supporting Information)

Ruigang Xu[a], Hui Zhou[b], Han Yong Bae[c], Vijay N. Wakchaure[a], Lorenzo Baldinelli[d], Isaac F. Leach[d], Giovanni Bistoni[d], Philip Kraft[e], and Benjamin List[a]*

[a] Max-Planck-Institut für Kohlenforschung, Kaiser-Wilhelm-Platz 1, 45470 Mülheim an der Ruhr, Germany

*Email: list@kofo.mpg.de

[b] College of Chemistry, Central China Normal University, Wuhan, P. R. China

[c] Department of Chemistry, Sungkyunkwan University, Suwon, Republic of Korea

[d] Department of Chemistry, Biology, and Biotechnology, University of Perugia, 06123 Perugia, Italy

[e] Symrise AG, S&C Global Innovation Fragrances, Building D 209, Mühlenfeldstr. 1, 37603 Holzminden, Germany

Table of Contents

1. General Information and Instrumentation 2
2. Synthesis and Characterization of 2-Substituted Cycloenones 4
3. Development of Reaction Conditions and Catalyst Investigation 10
4. Enantioselective Mukaiyama–Michael Addition and its Application 19
5. Synthesis and Characterization of Catalysts 45
6. HPLC Traces of the Products 54
7. NMR Study 108
8. Computational Studies 109
9. ECD Experiments for Determination of the Absolute Configuration 165
10. Crystallographic Data of **7** 168
11. ^{1}H , ^{13}C, ^{31}P and ^{19}F NMR Spectra of Substrates and Products 172
12. Reference 302

1. General Information and Instrumentation

Chemicals: Unless otherwise indicated, starting materials were purchased from Sigma-Aldrich, ABCR GmbH, TCI, or BLD Pharm. Commercial reagents were used without further purification. The chiral imidodiphosphorimidate acids (IDPis) **4a**, **4b**, **4e**, **4f** were synthesized according to established literature procedures[1,2].

Solvents: Solvents such as toluene (PhMe), dichloromethane (DCM), tetrahydrofuran (THF), diethyl ether (Et_2O), hexane, and acetonitrile (CH_3CN) were dried by distillation from appropriate drying agents at the Max-Planck-Institut für Kohlenforschung and stored in Schlenk flasks under an argon atmosphere. In addition, more solvents were purchased from commercial suppliers and dried over molecular sieves.

Inert Gas: Dry argon (purity >99.5%) was supplied by Air Liquide.

Thin Layer Chromatography: TLC was performed using pre-coated silica gel plastic sheets (Polygram SIL G/UV_{254}, 0.2 mm, with fluorescent indicator; Macherey-Nagel). Visualization was achieved using a UV lamp (254 nm) and/or phosphomolybdic acid (PMA) stain, and/or Cerium Ammonium Molybdate (CAM) stain, and/or basic $KMnO_4$ stain.

Column Chromatography: Column chromatography was conducted on Merck silica gel (60 Å, 230−400 mesh, particle size 0.040−0.063 mm) using technical grade solvents, with elution accelerated by compressed argon. Yields reported refer to compounds that are spectroscopically and chromatographically pure.

Nomenclature: Nomenclature follows the recommendations provided by the computer program ChemBioDraw (version 12.0.3.1216, CambridgeSoft).

Nuclear Magnetic Resonance Spectroscopy: ^{1}H, ^{13}C, ^{19}F, ^{31}P Nuclear magnetic resonance (NMR) spectra were recorded on a Bruker AVIII-500 MHz spectrometer in appropriate deuterated solvents. The solvent and corresponding measurement frequencies are indicated for each experiment. Chemical shifts (δ) are reported relative to tetramethylsilane (TMS). Multiplicities are denoted as follows: s (singlet), d (doublet), t (triplet), q (quadruplet), p (pentet), h (heptet), m (multiplet), and br (broad). All spectra were recorded at 298 K, processed with MestReNova 14.1.2 suits of program, and coupling constants are reported as observed. The residual deuterated solvent signal relative to tetramethylsilane was used as the internal reference in ^{1}H NMR spectra (e.g. $CDCl_3$ = 7.26 ppm, CD_2Cl_2 = 5.32 ppm, toluene-d_8 = 7.19, 2.30 ppm). Signals are reported as follows: chemical shift δ in ppm (multiplicity, coupling constant *J* in Hz, number of protons). All X-nuclei spectra were acquired proton decoupled unless otherwise noted.

Mass Spectrometry: Electrospray ionization (ESI) mass spectrometry was conducted on a Bruker ESQ 3000 spectrometer. High resolution mass spectrometry (HRMS) was performed on a Finnigan MAT 95 (EI) or Bruker APEX III FTMS (7 T magnet, ESI). The ionization method and mode of detection employed is indicated for the respective experiment. The ionization method and mode of detection employed is indicated for the respective experiment and all masses are reported in atomic units per elementary charge (*m/z*) with an intensity normalized to the most intense peak.

Specific Rotations: Specific rotations ($[\alpha]_D^T$) were measured with a Rudolph RA Autopol IV Automatic Polarimeter at the indicated temperature with a sodium lamp (sodium D line, λ = 589 nm). Measurements were performed in an acid resistant 1 mL cell (50 mm length) with concentrations *c* (g/(100 mL)) reported in the corresponding solvent.

High Performance Liquid Chromatography: High performance liquid chromatography (HPLC) was performed on a Shimadzu LC-20AD liquid chromatograph SIL-20AC auto sampler, CMB-20A using Daicel/Merck columns with a chiral stationary phase. All solvents were HPLC-grade and purchased from Sigma-Aldrich. The specific column and solvent mixture used are indicated for each experiment.

Gas Chromatography: Gas chromatography (GC) analyses using a chiral stationary phase were carried out on HP 6890 and 5890 series instruments equipped with a split-mode capillary injection system, flame ionization detector (FID), and hydrogen as the carrier gas. These analyses were performed in the GC department of the Max-Planck-Institut für Kohlenforschung, with detailed conditions provided for each experiment.

Abbreviations: IDPi = imidodiphosphorimidate, DSI = disulfonimide, CPA = chiral phosphoric acid, IDP = imidodiphosphate, e.r. = enantiomeric ratio, d.r. = diastereometric ratio, TLC = thin layer chromatography, PhMe = toluene, DCM = dichloromethane, Et_2O = diethyl ether; THF = tetrahydrofuran, CH_3CN = acetonitrile, TBS = *tert*-butyldimethylsilyl, Tf = trifluoromethanesulfonyl ($-SO_2CF_3$), C_6F_5 = perfluorophenyl, TFA = trifluoroacetic acid.

2. Synthesis and Characterization of 2-Substituted Cycloenones

Unless otherwise specified, all chemicals were purchased from commercial suppliers (TCI, BLD Pharm, Fluorochem, Sigma-Aldrich, Alfa Aesar, & ABCR) and were used following column purification or distillation. Enol silanes were synthesized according to previously reported procedures[3]. The other enone substrates were prepared using the methods described below.

Preparation of 2-iodocyclopentenone

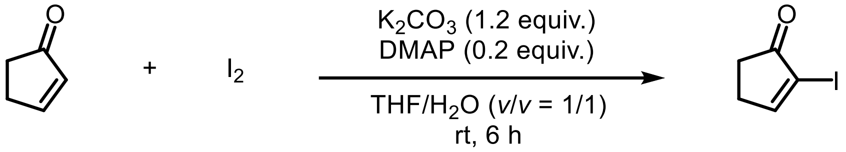

Cyclopentenone (5.0 g, 61.0 mmol) was dissolved in a 1:1 mixture of THF and H_2O (250 mL). To this solution, K_2CO_3 (10.1 g, 73.2 mmol, 1.2 equiv.), I_2 (23.2 g, 91.5 mmol, 1.5 equiv.), and DMAP (1.4 g, 12.2 mmol, 0.2 equiv.) were added sequentially at room temperature. The reaction mixture was stirred at ambient temperature for 6 h until completion. The mixture was then diluted with EtOAc and washed with ice-cooled saturated $Na_2S_2O_3$ solution (100 mL). The organic layer was separated, and the aqueous phase was extracted with EtOAc (3 × 50 mL). The combined organic extracts were dried over anhydrous Na_2SO_4 and concentrated under reduced pressure. The crude product was purified by flash-column chromatography on silica gel using ^{i}hexane/EtOAc (from 10:1 to 4:1 *v*/*v*) as eluent, affording the desired product as a white solid (12.5 g, 99% yield). Spectroscopic data was consistent with the values reported in the literature.[4]
Note: 2-iodocyclopentenone should be stored in argon and avoid the light.
^{1}H NMR (501 MHz, $CDCl_3$): δ 8.04 (s, 1H), 2.80 (ddd, *J* = 7.4, 3.0, 2.0 Hz, 2H), 2.56–2.50 (m, 2H).
^{13}C NMR (126 MHz, $CDCl_3$): δ 204.0, 169.5, 102.9, 31.3, 30.9.
HRMS (GC-EI): calculated for C_5H_5OI ($[M]^+$): 207.9380, found: 207.9377.

General Procedure A:

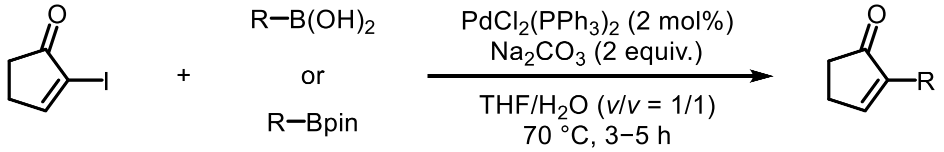

In an oven-dried Schlenk flask under the Ar atmosphere, 2-iodocyclopentenone (0.52 g, 2.5 mmol, 1 equiv.), Na_2CO_3 (0.54 g, 5 mmol, 2 equiv.), the appropriate boronic acid or ester (3.75 mmol, 1.5 equiv.), and $PdCl_2(PPh_3)_2$ (35.1 mg, 0.05 mmol, 0.02 equiv.). The flask was refilled with Ar twice before addition of degassed THF and H_2O (*v*/*v* = 1/1, 0.25 M). The reaction mixture was stirred at 70 °C under Ar for 3 to 5 h and then allowed to cool to ambient temperature. EtOAc was added, and any precipitate was removed by suction filtration. The aqueous layer was extracted 3 times with EtOAc, washed with brine and dried over $MgSO_4$. The combined organic layers was concentrated in vacuo, and the crude product was purified by flash chromatography to afford the corresponding 2-arylcylopentenone or 2-alkenylcyclopentenone.

General Procedure B:

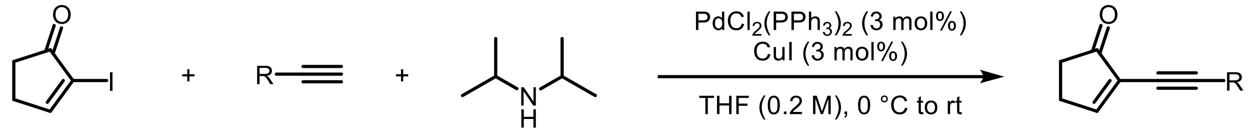

In an oven-dried Schlenk flask under the Ar atmosphere, 2-iodocyclopentenone (0.642 g, 3 mmol), $PdCl_2(PPh_3)_2$ (63.2 mg, 0.09 mmol, 3 mol%), CuI (17.1 mg, 0.09 mmol, 3 mol%) and the terminal alkyne (6 mmol, 2 equiv.) were added to THF (0.2 M) at 0 °C. Diisopropylamine (1.26 mL, 9 mmol, 3.0 equiv.) was then introduced, and the reaction mixture was stirred for 1 h at 0 °C before being allowed to warm to ambient temperature to complete the reaction, as monitored by TLC analysis. After completion, the resulting mixture was diluted with EtOAc (2 × 20 mL). The separated organic layer was washed with water and brine, dried over $MgSO_4$, and concentrated in vacuo. The crude product was purified by column chromatography on silica gel using ^{i}hexane/EtOAc as the eluent to afford the corresponding products.

2-(prop-1-en-2-yl)cyclopent-2-en-1-one (1f)

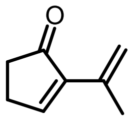

According to *general procedure A*, using 2-isopropenyl-4,4,5,5-tetramethyl-1,3,2-dioxaborolane (CAS: 126726-62-3) as starting material (630 mg, 3.75 mmol). The desired product **1f** was obtained after flash column chromatography on silica gel using ^{i}hexane/EtOAc (from 10:1 to 4:1 *v/v*) as eluent (253 mg, 83% yield, light yellow oil).

^{1}H NMR (501 MHz, CD_2Cl_2): δ 7.55 (t, *J* = 3.0 Hz, 1H), 6.10–6.05 (m, 1H), 5.19–5.15 (m, 1H), 2.65–2.59 (m, 2H), 2.50–2.45 (m, 2H), 1.98 (t, *J* = 1.2 Hz, 3H).

^{13}C NMR (126 MHz, CD_2Cl_2): δ 207.5, 158.2, 142.5, 134.9, 115.8, 35.9, 25.6, 21.7.

HRMS (GC-EI): calculated for $C_8H_{10}O$ ($[M]^+$): 122.0726, found: 122.0728.

2-(cyclohex-1-en-1-yl)cyclopent-2-en-1-one (1g)

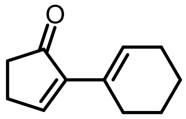

According to *general procedure A*, using cyclohexen-1-yl-boronic acid (CAS: 89490-05-1) as starting material (472 mg, 3.75 mmol). The desired product **1g** was obtained after flash column chromatography on silica gel using ^{i}hexane/EtOAc (from 10:1 to 4:1 *v/v*) as eluent (259 mg, 83% yield, light yellow oil).

^{1}H NMR (501 MHz, CD_2Cl_2): δ 7.43–7.39 (m, 1H), 6.92–6.84 (m, 1H), 2.60–2.56 (m, 2H), 2.46–2.43 (m, 2H), 2.24–2.16 (m, 4H), 1.77–1.71 (m, 2H), 1.66–1.61 (m, 2H).

^{13}C NMR (126 MHz, CD_2Cl_2): δ 208.1, 155.2, 142.9, 128.7, 128.1, 36.1, 26.6, 25.52, 25.48, 22.7, 22.0.

HRMS (GC-EI): calculated for $C_{11}H_{14}O$ ($[M]^+$): 162.1039, found: 162.1042.

2-(1-phenylvinyl)cyclopent-2-en-1-one (1h)

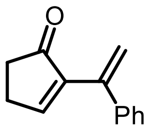

According to *general procedure A*, using 1-phenylvinylboronic acid (CAS: 14900-39-1) as starting material (555 mg, 3.75 mmol). The desired product **1h** was obtained after flash column chromatography on silica gel using ^{i}hexane/EtOAc (from 10:1 to 4:1 *v/v*) as eluent (350 mg, 76% yield, light yellow oil).

^{1}H NMR (501 MHz, CD_2Cl_2): δ 7.45 (t, *J* = 2.9 Hz, 1H), 7.42–7.29 (m, 6H), 6.03 (d, *J* = 1.7 Hz, 1H), 5.49 (d, *J* = 1.7 Hz, 1H), 2.69–2.63 (m, 2H), 2.58–2.52 (m, 2H).
^{13}C NMR (126 MHz, CD_2Cl_2): δ 207.2, 161.5, 143.1, 141.0, 140.7, 128.2, 127.6, 127.5, 116.9, 35.7, 26.0.
HRMS (GC-EI): calculated for $C_{13}H_{12}O$ ($[M]^+$): 184.0883, found: 184.0885.

2-(2-methylprop-1-en-1-yl)cyclopent-2-en-1-one (1i)

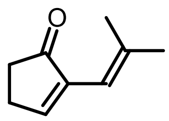

According to *general procedure A*, using 2,2-dimethylethenylboronic acid pinacol ester (CAS: 126689-00-7) as starting material (683 mg, 3.75 mmol). The desired product **1i** was obtained after flash column chromatography on silica gel using ^{i}hexane/EtOAc (from 10:1 to 4:1 *v/v*) as eluent (173 mg, 51% yield, light yellow oil).
^{1}H NMR (501 MHz, CD_2Cl_2): δ 7.49 (s, 1H), 6.06–5.67 (m, 1H), 2.68 (q, *J* = 3.8 Hz, 2H), 2.45–2.27 (m, 2H), 1.93 (s, 3H), 1.87 (s, 3H).
^{13}C NMR (126 MHz, CD_2Cl_2): δ 208.8, 156.7, 141.4, 140.2, 114.0, 33.8, 26.7, 26.7, 20.1.
HRMS (GC-EI): calculated for $C_9H_{12}O$ ($[M]^+$): 136.0883, found: 136.0885.

2-(phenylethynyl)cyclopent-2-en-1-one (1j)

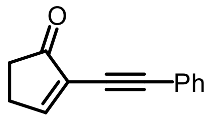

According to *general procedure B*, using phenylacetylene (CAS: 536-74-3) as starting material (0.659 mL, 6 mmol). The desired product **1j** was obtained after flash column chromatography on silica gel using ^{i}hexane/EtOAc (from 10:1 to 4:1 *v/v*) as eluent (520 mg, 91% yield, light yellow solid).
^{1}H NMR (501 MHz, CD_2Cl_2): δ 7.90 (t, *J* = 3.1 Hz, 1H), 7.56–7.53 (m, 2H), 7.41–7.38 (m, 3H), 2.80–2.77 (m, 2H), 2.52–2.49 (m, 2H).
^{13}C NMR (126 MHz, CD_2Cl_2): δ 205.2, 165.5, 131.7, 129.6, 128.8, 128.4, 122.4, 95.1, 80.2, 34.0, 27.4.
HRMS (GC-EI): calculated for $C_{13}H_{10}O$ ($[M]^+$): 182.0726, found: 182.0729.

2-(pent-1-yn-1-yl)cyclopent-2-en-1-one (1k)

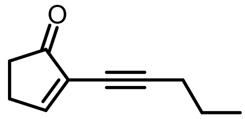

According to *general procedure B,* using 1-pentyne (CAS: 627-19-0) as starting material (0.592 mL, 6 mmol). The desired product **1k** was obtained after flash column chromatography on silica gel using ^{i}hexane/EtOAc (from 10:1 to 4:1 *v/v*) as eluent (409 mg, 92% yield, light yellow oil).
^{1}H NMR (501 MHz, CD_2Cl_2): δ 7.72 (s, 1H), 2.71–2.67 (m, 2H), 2.43–2.38 (m, 4H), 1.62 (h, *J* = 7.3 Hz, 2H), 1.04 (t, *J* = 7.4 Hz, 3H).
^{13}C NMR (126 MHz, CD_2Cl_2): δ 205.9, 164.2, 130.2, 96.8, 71.5, 33.9, 27.0, 22.0, 21.3, 13.2.
HRMS (GC-EI): calculated for $C_{10}H_{12}O$ ($[M]^+$): 148.0883, found: 148.0884.

2-(naphthalen-2-yl)cyclopent-2-en-1-one (1l)

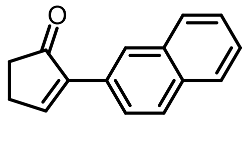

According to *general procedure A,* using 2-naphthylboronic acid (CAS: 32316-92-0) as starting material (645 mg, 3.75 mmol). The desired product **1l** was obtained after flash column chromatography on silica gel using ^{i}hexane/EtOAc (from 10:1 to 4:1 *v/v*) as eluent (338 mg, 65% yield, white solid).

^{1}H NMR (501 MHz, CD_2Cl_2): δ 8.40 (s, 1H), 8.03 (t, *J* = 3.0 Hz, 1H), 7.95–7.91 (m, 1H), 7.91–7.85 (m, 2H), 7.77 (dd, *J* = 8.6, 1.7 Hz, 1H), 7.55–7.51 (m, 2H), 2.81–2.78 (m, 2H), 2.67–2.64 (m, 2H).
^{13}C NMR (126 MHz, CD_2Cl_2): δ 207.5, 159.5, 142.7, 133.3, 133.1, 129.3, 128.4, 127.8, 127.5, 126.24, 126.17, 126.1, 124.8, 35.9, 26.3.
HRMS (GC-EI): calculated for $C_{15}H_{12}O$ ($[M]^+$): 208.0883, found: 208.0886.

2-phenylcyclopent-2-en-1-one (1m)

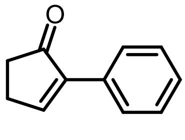

According to *general procedure A*, using phenylboronic acid (CAS: 98-80-6) as starting material (457 mg, 3.75 mmol). The desired product **1m** was obtained after flash column chromatography on silica gel using ^{i}hexane/EtOAc (from 10:1 to 4:1 *v*/*v*) as eluent (285 mg, 72% yield, white solid).
^{1}H NMR (501 MHz, CD_2Cl_2): δ 7.89 (t, *J* = 3.0 Hz, 1H), 7.77–7.68 (m, 2H), 7.44–7.40 (m, 2H), 7.39–7.34 (m, 1H), 2.74 (dt, *J* = 7.5, 2.8 Hz, 2H), 2.61–2.58 (m, 2H).
^{13}C NMR (126 MHz, CD_2Cl_2): δ 207.3, 159.1, 143.1, 132.0, 128.3, 128.2, 127.0, 35.7, 26.2
HRMS (GC-EI): calculated for $C_{11}H_{10}O$ ($[M]^+$): 158.0726, found: 158.0728.

2-(4-(trifluoromethyl)phenyl)cyclopent-2-en-1-one (1n)

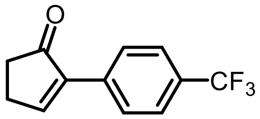

According to *general procedure A*, using 4-(trifluoromethyl)phenylboronic acid (CAS: 128796-39-4) as starting material (712 mg, 3.75 mmol). The desired product **1n** was obtained after column chromatography on silica gel using ^{i}hexane/EtOAc (from 10:1 to 4:1 *v*/*v*) as eluent (362 mg, 64% yield, yellowish solid).
^{1}H NMR (501 MHz, CD_2Cl_2): δ 8.00 (t, *J* = 3.0 Hz, 1H), 7.88 (d, *J* = 8.1 Hz, 2H), 7.68 (d, *J* = 8.2 Hz, 2H), 2.78 (dt, *J* = 7.6, 2.8 Hz, 2H), 2.66–2.52 (m, 2H).
^{13}C NMR (151 MHz, CD_2Cl_2): δ 207.2, 161.4, 142.3, 135.9 (q, *J* = 1.3 Hz), 130.2 (q, *J* = 32.3 Hz), 127.8, 125.6 (q, *J* = 3.8 Hz), 124.7 (q, *J* = 271.8 Hz), 36.1, 26.8.
^{19}F NMR (471 MHz, CD_2Cl_2): δ –62.97.
HRMS (GC-EI): calculated for $C_{12}H_9OF$ ($[M]^+$): 226.0600, found: 226.0602.

2-(4-chlorophenyl)cyclopent-2-en-1-one (1o)

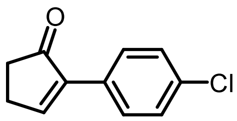

According to *general procedure A*, using 4-chlorophenylboronic acid (CAS: 1679-18-1) as starting material (586 mg, 3.75 mmol). The desired product **1o** was obtained after flash column chromatography on silica gel using ^{i}hexane/EtOAc (from 10:1 to 4:1 *v*/*v*) as eluent (265 mg, 55% yield, white solid).
^{1}H NMR (501 MHz, CD_2Cl_2): δ 7.90 (t, *J* = 3.0 Hz, 0H), 7.75–7.68 (m, 1H), 7.43–7.37 (m, 1H), 2.77–2.71 (m, 1H), 2.62–2.57 (m, 1H).
^{13}C NMR (126 MHz, CD_2Cl_2): δ 207.0, 159.5, 141.8, 133.9, 130.5, 128.5, 128.3, 35.7, 26.2.
HRMS (GC-EI): calculated for $C_{11}H_9OCl$ ($[M]^+$): 192.0336, found: 192.0340.

2-(p-tolyl)cyclopent-2-en-1-one (1p)

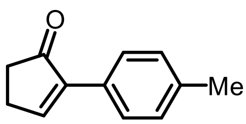

According to *general procedure A*, using 4-methylphenylboronic acid (CAS: 5720-05-8) as starting material (510 mg, 3.75 mmol). The desired

product **1p** was obtained after flash column chromatography on silica gel using ^{i}hexane/EtOAc (from 10:1 to 4:1 *v*/*v*) as eluent (310 mg, 72% yield, yellowish solid).
^{1}H NMR (501 MHz, CD_2Cl_2): δ 7.84 (t, *J* = 3.0 Hz, 1H), 7.67–7.58 (m, 2H), 7.23 (d, *J* = 7.9 Hz, 2H), 2.72 (dt, *J* = 7.6, 2.8 Hz, 2H), 2.59–2.56 (m, 2H), 2.39 (s, 3H).
^{13}C NMR (126 MHz, CD_2Cl_2): δ 207.5, 158.3, 142.9, 138.2, 129.05, 128.95, 126.8, 35.7, 26.1, 21.0.
HRMS (GC-EI): calculated for $C_{12}H_{12}O$ ($[M]^+$): 172.0883, found: 172.0884.

2-(4-methoxyphenyl)cyclopent-2-en-1-one (1q)

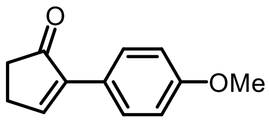

According to *general procedure A*, using 4-methoxyphenylboronic acid (CAS: 5720-07-0) as starting material (570 mg, 3.75 mmol). The desired product **1q** was obtained after flash column chromatography on silica gel using ^{i}hexane/EtOAc (from 10:1 to 4:1 *v*/*v*) as eluent (409 mg, 87% yield, yellowish solid).
^{1}H NMR (501 MHz, CD_2Cl_2): δ 7.79 (t, *J* = 3.0 Hz, 1H), 7.72–7.68 (m, 2H), 6.96–6.93 (m, 2H), 3.85 (s, 3H), 2.73–2.69 (m, 2H), 2.58–2.55 (m, 2H).
^{13}C NMR (126 MHz, CD_2Cl_2): δ 207.6, 159.7, 157.3, 142.4, 128.2, 124.5, 113.7, 55.2, 35.7, 26.0.
HRMS (GC-EI): calculated for $C_{12}H_{12}O_2$ ($[M]^+$): 188.0832, found: 188.0834.

2-(furan-2-yl)cyclopent-2-en-1-one (1r)

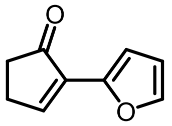

According to *general procedure A*, using furan-2-boronic acid (CAS: 13331-23-2) as starting material (420 mg, 3.75 mmol). The desired product **1r** was obtained after flash column chromatography on silica gel using ^{i}hexane/EtOAc (from 10:1 to 4:1 *v*/*v*) as eluent (152 mg, 41% yield, brown solid).
^{1}H NMR (501 MHz, CD_2Cl_2): δ 7.84 (t, *J* = 3.2 Hz, 1H), 7.48 (d, *J* = 1.8 Hz, 1H), 7.02 (d, *J* = 3.3 Hz, 1H), 6.49 (dd, *J* = 3.4, 1.8 Hz, 1H), 2.77 (dt, *J* = 7.7, 3.0 Hz, 2H), 2.56–2.52 (m, 2H).
^{13}C NMR (126 MHz, CD_2Cl_2): δ 205.2, 154.2, 147.1, 142.3, 134.4, 111.2, 108.8, 35.2, 26.6.
HRMS (GC-EI): calculated for $C_9H_8O_2$ ($[M]^+$): 148.0519, found: 148.0521.

4,5-dihydro-[1,1'-biphenyl]-2(3H)-one (1v)

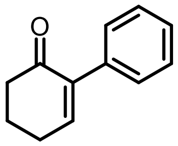

According to *general procedure A*, using phenylboronic acid (CAS: 98-80-6) as starting material (420 mg, 3.75 mmol). The desired product **1v** was obtained after flash column chromatography on silica gel using ^{i}hexane/EtOAc (from 10:1 to 4:1 *v*/*v*) as eluent (379 mg, 88% yield, white solid).
^{1}H NMR (501 MHz, CD_2Cl_2): δ 7.40–7.30 (m, 5H), 7.07 (t, *J* = 4.3 Hz, 1H), 2.61–2.55 (m, 4H), 2.16–2.11 (m, 2H).
^{13}C NMR (126 MHz, CD_2Cl_2): δ 197.6, 148.1, 140.2, 137.0, 128.7, 127.8, 127.4, 39.1, 26.6, 23.0.
HRMS (GC-EI): calculated for $C_{12}H_{12}O$ ($[M]^+$): 172.0883, found: 172.0885.

2-(m-tolyl)cyclopent-2-en-1-one (1aa)

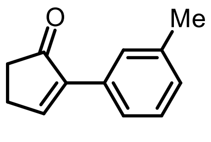

According to *general procedure A*, using 3-methylphenylboronic acid (CAS: 17933-03-8) as starting material (510 mg, 3.75 mmol). The desired product

1aa was obtained after flash column chromatography on silica gel using ^{i}hexane/EtOAc (from 10:1 to 4:1 *v*/*v*) as eluent (293 mg, 68% yield, yellowish solid).
^{1}H NMR (501 MHz, CD_2Cl_2): δ 7.86 (t, *J* = 3.0 Hz, 1H), 7.53 (s, 1H), 7.50 (d, *J* = 7.8 Hz, 1H), 7.30 (t, *J* = 7.6 Hz, 1H), 7.19 (d, *J* = 7.6 Hz, 1H), 2.73 (dt, *J* = 7.5, 2.8 Hz, 2H), 2.60–2.57 (m, 2H), 2.40 (s, 3H).
^{13}C NMR (126 MHz, CD_2Cl_2): δ 207.3, 159.0, 143.2, 138.0, 131.9, 128.9, 128.1, 127.6, 124.1, 35.7, 26.2, 21.2.
HRMS (GC-EI): calculated for $C_{12}H_{12}O$ ($[M]^+$): 172.0883, found: 172.0884.

2-(3,5-dimethylphenyl)cyclopent-2-en-1-one (1ab)

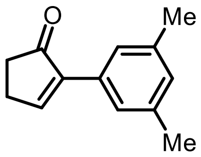

According to *general procedure A*, using 3,5-dimethylphenylboronic acid (CAS: 172975-69-8) as starting material (562 mg, 3.75 mmol). The desired product **1ab** was obtained after flash column chromatography on silica gel using ^{i}hexane/EtOAc (from 10:1 to 4:1 *v*/*v*) as eluent (284 mg, 61% yield, yellowish solid).
^{1}H NMR (501 MHz, CD_2Cl_2): δ 7.83 (t, *J* = 3.0 Hz, 1H), 7.32 (s, 2H), 7.02 (s, 1H), 2.73–2.70 (m, 2H), 2.57 (ddd, *J* = 7.3, 2.8, 1.4 Hz, 2H), 2.36 (s, 6H).
^{13}C NMR (126 MHz, CD_2Cl_2): δ 207.4, 158.9, 143.4, 137.8, 131.8, 129.8, 124.8, 35.8, 26.1, 21.0.
HRMS (GC-EI): calculated for $C_{13}H_{14}O$ ($[M]^+$): 186.1039, found: 186.1042.

2-(4-(trifluoromethoxy)phenyl)cyclopent-2-en-1-one (1ac)

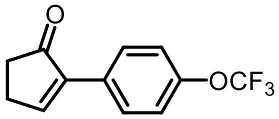

According to *general procedure A*, using 4-(trifluoromethoxy) phenylboronic acid (CAS: 139301-27-2) as starting material (772 mg, 3.75 mmol). The desired product **1ac** was obtained after flash column chromatography on silica gel using ^{i}hexane/EtOAc (from 10:1 to 4:1 *v*/*v*) as eluent (400 mg, 66% yield, white solid).
^{1}H NMR (501 MHz, CD_2Cl_2): δ 7.91 (t, *J* = 3.0 Hz, 1H), 7.83–7.73 (m, 2H), 7.34–7.24 (m, 2H), 2.78–2.71 (m, 2H), 2.64–2.58 (m, 2H).
^{13}C NMR (126 MHz, CD_2Cl_2): δ 207.0, 159.7, 149.0, 141.8, 130.8, 128.5, 120.8, (q, *J* = 257.0 Hz), 35.6, 26.2.
^{19}F NMR (471 MHz, CD_2Cl_2): δ –58.19.
HRMS (GC-EI): calculated for $C_{12}H_9O_2F_3$ ($[M]^+$): 242.0549, found: 242.0552.

3. Development of Reaction Conditions and Catalyst Investigation

Reaction conditions were optimized as described below using 2-pentyl cyclopentenone as the representative substrate.

In an oven-dried screw-cap vial equipped with a magnetic stirring bar, the **catalyst** (1 mol%) was charged under inert Ar atmosphere. Freshly distilled solvent (0.2 M) and silyl ketene acetal **2** (0.075 mmol, 1.5 equiv.) were added sequentially. The reaction mixture was stirred at room temperature for 15 min before fully cooled down to the indicated temperature. Subsequently, the corresponding enone **1** (0.05 mmol, 1.0 equiv.) was added slowly and the mixture was stirred for additional 18 h. After completion, the reaction was quenched with 5 μL of trimethylamine and carefully warmed to room temperature. The reaction progress was monitored by ^{1}H NMR spectroscopy using CH_2Br_2 as an internal standard for conversion determination. Following solvent evaporation, the crude product was purified by preparative TLC (silica gel: 8% EtOAc /pentane; partially stained with $KMnO_4$) and analyzed by HPLC to determine the enantiomeric ratio.

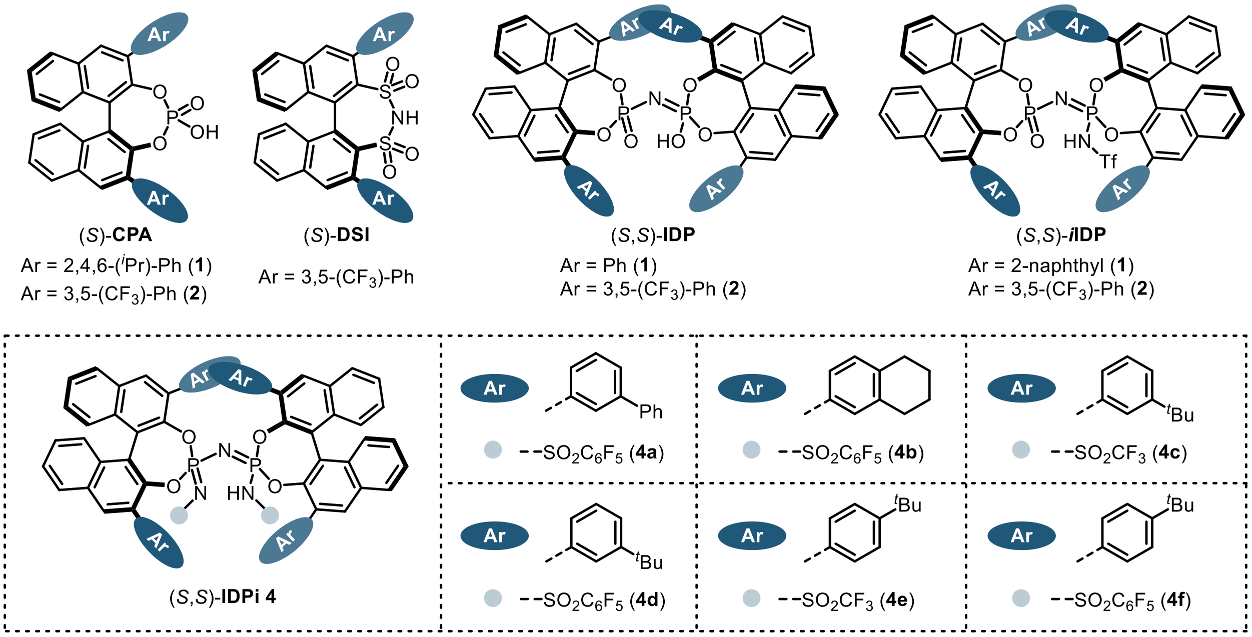

Figure S1. Initial screening of commonly used Brønsted acid catalysts.

Table S1. Reaction optimization for the Mukaiyama–Michael addition[a]

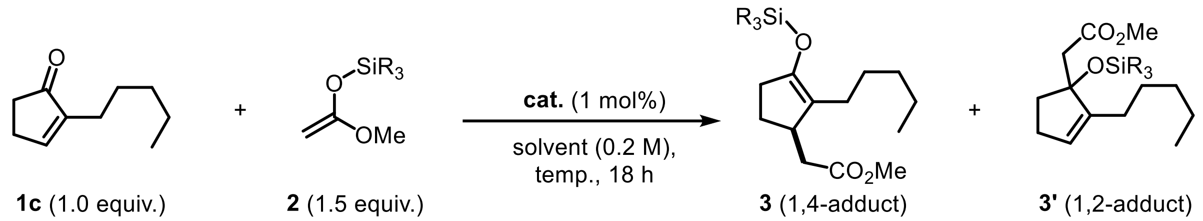

entry	cat.	[Si]	solvent	temp. (°C)	conv. (%)	3c/3c'	e.r.
1	**CPA 1**	TBS	Et_2O	–60	--	--	--
2	**CPA 2**	TBS	Et_2O	–60	--	--	--
3	**IDP 1**	TBS	Et_2O	–60	--	--	--
4	**IDP 2**	TBS	Et_2O	–60	--	--	--
5	***i*IDP 1**	TBS	Et_2O	–60	--	--	--
6	***i*IDP 2**	TBS	Et_2O	–60	--	--	--
7	**DSI**	TBS	Et_2O	–60	99	enolate	50:50
8	**IDPi 4a**	TBS	Et_2O	–60	99	>20:1	25:75
9	**IDPi 4a**	TMS	Et_2O	–60	99	12:1	30:70
10	**IDPi 4a**	TIPS	Et_2O	–60	99	>20:1	44:56
11	**IDPi 4b**	TBS	Et_2O	–60	99	>20:1	78:22
12	**IDPi 4c**	TBS	Et_2O	–60	99	>20:1	10:90
13	**IDPi 4d**	TBS	Et_2O	–60	99	>20:1	14:86
14	**IDPi 4c**	TBS	DCM	–60	99	20:1	33:67
15	**IDPi 4c**	TBS	THF	–60	99	17:1	22:78
16	**IDPi 4c**	TBS	PhMe	–60	99	>20:1	8:92
17	**IDPi 4c**	TBS	hexane	–60	99	>20:1	11:89
18	**IDPi 4c**	TBS	PhMe	0	99	>20:1	27:73
19	**IDPi 4c**	TBS	PhMe	–20	99	>20:1	20:80
20	**IDPi 4c**	TBS	PhMe	–40	99	>20:1	12:88
21	**IDPi 4c**	TBS	PhMe	–80	99	>20:1	6:94
22	**IDPi 4d**	TBS	PhMe	–80	99	>20:1	10:90
23	**IDPi 4e**	TBS	PhMe	–80	99	>20:1	89:11
24	**IDPi 4f**	TBS	PhMe	–80	99	>20:1	99:1

[a]Reactions were conducted with enone **1c** (0.05 mmol), silyl ketene acetal **2a** (1.5 equiv.), and **cat.** (1 mol%) in solvent (0.2 M) for 18 h at the indicated temperature. All conversions (conv.), site selectivities (**3**/**3'**) were determined by ^1H NMR of the crude reaction mixture using CH_2Br_2 as an internal standard. Enantiomeric ratios (e.r.) were measured by HPLC.

Table S2. Effect of silyl group on reactivity and selectivity[a]

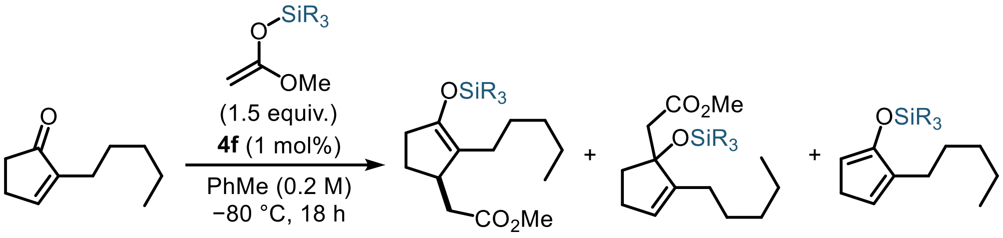

1c **3** **3'** **3''**

	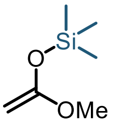 TMS-SKA	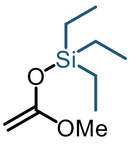 TES-SKA	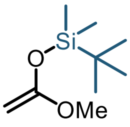 TBS-SKA	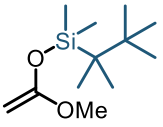 TDS-SKA	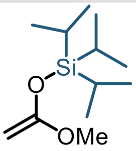 TIPS-SKA
conv.	full	full	full	93%	56%
e.r.	86:14	93:7	99:1	>99:1	98:2
**3/3'**	12:1	19:1	>20:1	>20:1	>20:1
**3/3''**	>20:1	>20:1	>20:1	18:1	10:1

[a]Reactions were conducted with enone **1c** (0.05 mmol), silyl ketene acetals (1.5 equiv.), and **4f** (1 mol%) in PhMe (0.2 M) for 18 h at −80 °C. All conversions (conv.), site selectivities (**3**/**3'**) and chemoselectivities (**3**/**3''**) were determined by ^{1}H NMR of the crude reaction mixture using CH_2Br_2 as an internal standard. Enantiomeric ratios (e.r.) were measured by HPLC.

Increasing the steric hindrance of the silyl group in the SKA (TMS < TES < TBS < TDS < TIPS) has a clear and systematic impact on both reactivity and selectivity. As illustrated in the **Table S2**, bulkier -SiR_3 impose stronger conformational constraints within the tight silylium-IDPi ion pair, thereby enhancing asymmetric induction and kinetically biasing the reaction away from the competing 1,2-addition. In this sense, both the enantioselectivity (e.r.) and site selectivity (**3**/**3'**) improve as the silyl group is enlarged from TMS to TDS. By contrast, overly bulky -SiR_3 introduce a substantial steric penalty for ion pair combination, impeding efficient silyl transfer and nucleophilic approach to the activated enone. This leads to reduced catalytic turnover and a larger contribution from competitive enolization pathways. For example, the TIPS-SKA shows a marked drop in conversion and reduced chemoselectivity (**3**/**3''** = 10:1). Overall, a medium-sized silyl group (TBS) provides the optimal balance, delivering high reactivity while maintaining excellent stereo-, regio- and chemoselectivity.

Table S3. Reoptimization for enone substrates[a]

enone	cat.	solvent	temp. (°C)	time (h)	conv. (%)	e.r.
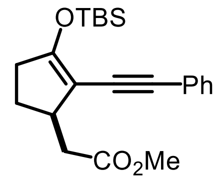 **3j**	**IDPi 4f**	PhMe	–80	18	99	84:16
	**IDPi 4a**	PhMe	–80	18	99	57:43
	**IDPi 4b**	PhMe	–80	18	99	64:36
	**IDPi 4e**	PhMe	–80	18	99	79:21
	**IDPi 4f**	Et_2O	–80	18	99 (97% yield)	93:7
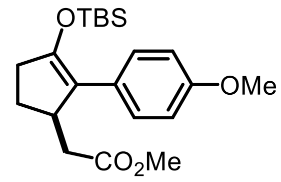 **3q**	**IDPi 4f**	PhMe	–80	18	<5	--
	**IDPi 4e**	PhMe	–80	18	40	85:15
	**IDPi 4b**	PhMe	–80	18	<5	--
	**IDPi 4f**	PhMe	–50	18	68	96:4
	**IDPi 4f**	PhMe	–50	36	95 (92% yield)	96:4
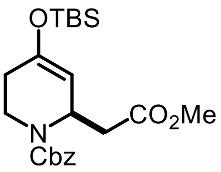 **3x**	**IDPi 4f**	PhMe	–80	18	99	61:39
	**IDPi 4a**	PhMe	–80	18	99	95:5
	**IDPi 4e**	PhMe	–80	18	99	58:42
	**IDPi 4b**	PhMe	–80	18	99	65:35
	**IDPi 4a**	Et_2O	–80	18	99 (96% yield)	98:2
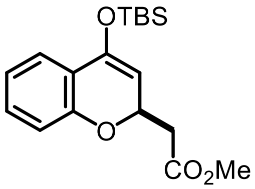 **3y**	**IDPi 4f**	PhMe	–80	18	99	76:24
	**IDPi 4a**	PhMe	–80	18	99	20:80
	**IDPi 4b**	PhMe	–80	18	99	54:46
	**IDPi 4e**	PhMe	–80	18	99	93:7
	**IDPi 4e**	Et_2O	–80	18	99 (93% yield)	95:5
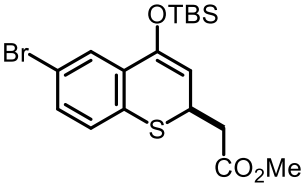 **3z**	**IDPi 4f**	PhMe	–80	18	99	65:35
	**IDPi 4a**	PhMe	–80	18	99 (91% yield)	98:2
	**IDPi 4b**	PhMe	–80	18	99	61:39
	**IDPi 4e**	PhMe	–80	18	99	11:89
	**IDPi 4f**	Et_2O	–50	18	99	93:7
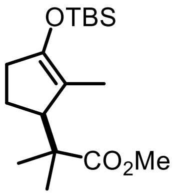 **5a**	**IDPi 4f**	PhMe	–80	18	n.r.	--
	**IDPi 4f**	Et_2O	–80	18	n.r.	--
	**IDPi 4a**	PhMe	–80	18	90 (84% yield)	93:7
	**IDPi 4a**	Et_2O	–80	18	99	92:8
	**IDPi 4a**	PhMe	–80	36	91	93:7

enone	cat.	solvent	temp. (°C)	time (h)	conv. (%)	e.r.
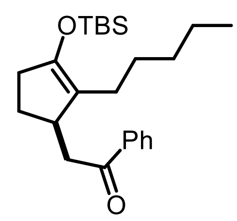	**IDPi 4f**	PhMe	–80	18	68	89:11
	**IDPi 4a**	PhMe	–80	18	99	70:30
	**IDPi 4b**	PhMe	–80	18	60	72:28
	**IDPi 4e**	PhMe	–80	18	89 (86% yield)	96:4
**5c**	**IDPi 4e**	PhMe	–80	36	88	96:4
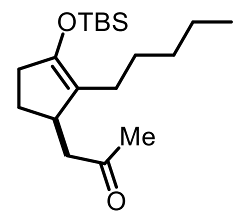	**IDPi 4f**	PhMe	–80	18	50	88:12
	**IDPi 4a**	PhMe	–80	18	99	71:29
	**IDPi 4b**	PhMe	–80	18	38	82:18
	**IDPi 4f**	Et_2O	–80	18	20	96:4
**5d**	**IDPi 4f**	Et_2O 3 equiv. of enol silane	–80	36	72 (68% yield)	96:4

[a]All conversions (conv.) were determined by ^{1}H NMR of the crude reaction mixture using CH_2Br_2 as an internal standard. Enantiomeric ratios (e.r.) were measured by HPLC.

Table S4. Current limited substrates

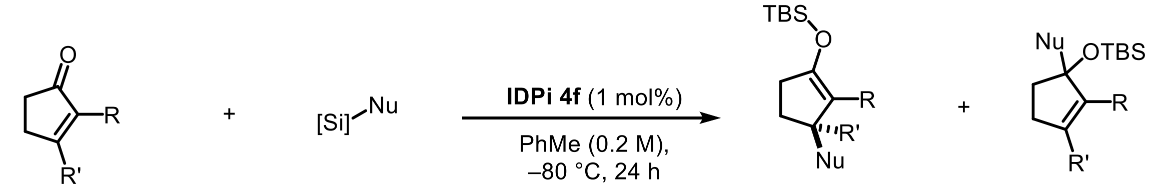

1 (1.0 equiv.) **2** (1.5 equiv.) **3** (1,4-adduct) **3'** (1,2-adduct)

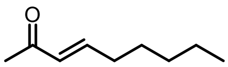

3/3' = 6:94

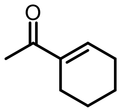

3/3' = 17:83

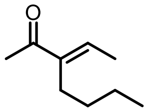

3/3' = 12:88

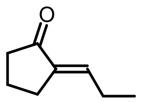

not determined

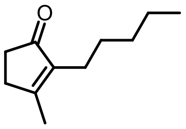

no reaction

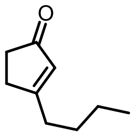

3/3' = 38:62

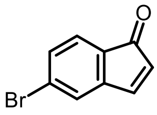

messy

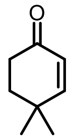

3/3' = 29:71

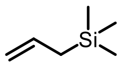

silylated pdt

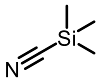

not determined

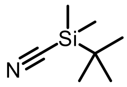

no reaction

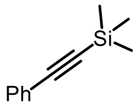

no reaction

Table S5. Reaction optimization for the *cis*-selective protodesilylation (catalyst screening)[a]

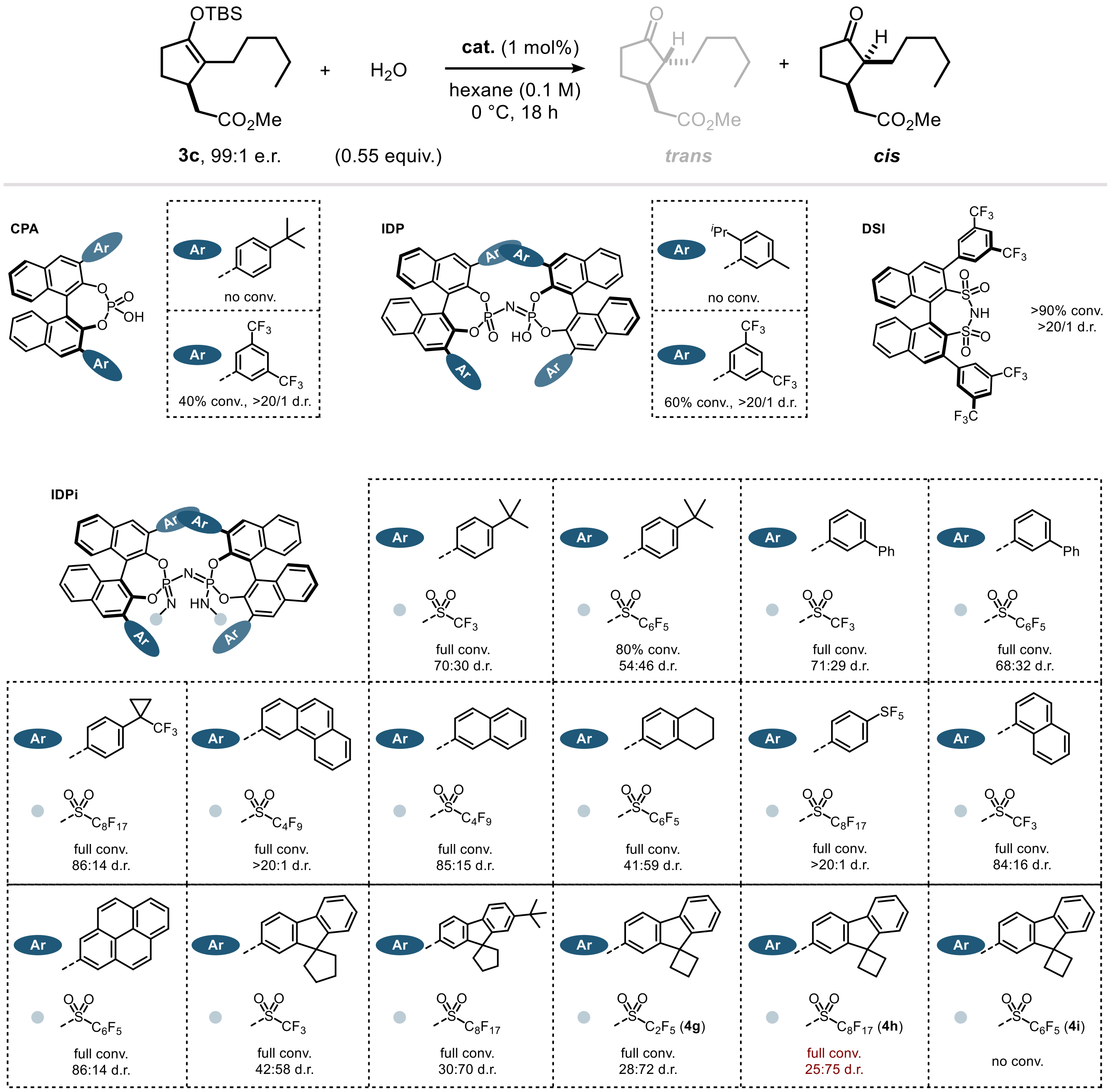

[a]Reactions were conducted with enol silane **3c** (0.05 mmol), distilled water (0.55 equiv.), and **cat.** (1 mol%) in dry hexane (0.1 M) for 18 h at 0 °C. All conversions (conv.) and diastereomeric ratios (d.r.) were determined by ^{1}H NMR of the crude reaction mixture using CH_2Br_2 as an internal standard. d.r. = *trans*:*cis*.

Table S6. Reaction optimization for the *cis*-selective protodesilylation (condition development)[a]

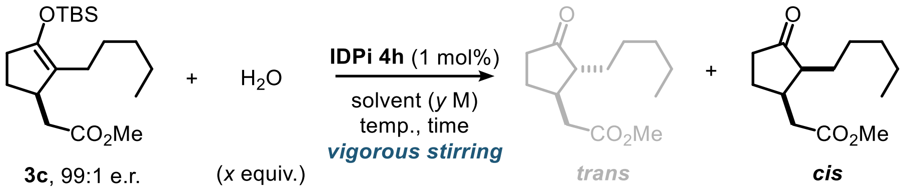

screening 1

entry	equiv. of water	conv. (%)	d.r.
1	0.55 (mild stirring, 300 rpm)	full	28:72
2	0.55 (vigorous stirring, 1200 rpm)	full	22:78
3	1.05 (1200 rpm)	full	21:79
4	1.5 (1200 rpm, 3 h)	full	19:81
5	1.5 (1200 rpm, 18 h)	full	19:81
6	2.0 (1200 rpm, 3 h)	full	20:80

screening 2

entry	temp.	conv. (%)	d.r.
1	rt	full	25:75
2	–1 °C	full	19:81
3	–10 °C	full	20:80
4	–20 °C	full	20:80
5	–30 °C	full	20:80
6	–50 °C	full	20:80

screening 3

entry	solvent	conv. (%)	d.r.
1	pentane	full	19:81
2	hexane	full	19:81
3	cyclohexane	--(*frozen*)	--
4	CH_2Cl_2	full	49:51
5	toluene	full	30:70
6	Et_2O	no reaction	--

screening 4

entry	conc. of hexane	conv. (%)	d.r.
1	0.05 M	full	19:81
2	0.1 M	full	19:81
3	0.2 M	full	20:80
4	0.5 M	full	26:74
5	1.0 M	full	26:74
6	0.1 M (0.5 mol% cat. loading)	full	19:81

[a]All conversions (conv.) and diastereomeric ratios (d.r.) were determined by ^{1}H NMR of the crude reaction mixture using CH_2Br_2 as an internal standard. d.r. = *trans*:*cis*.

A

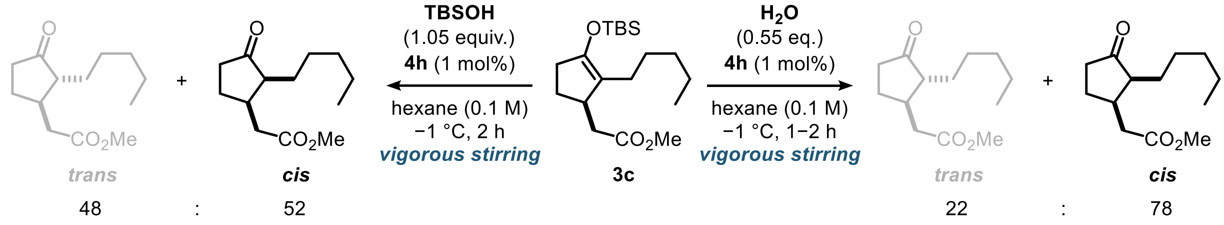

B

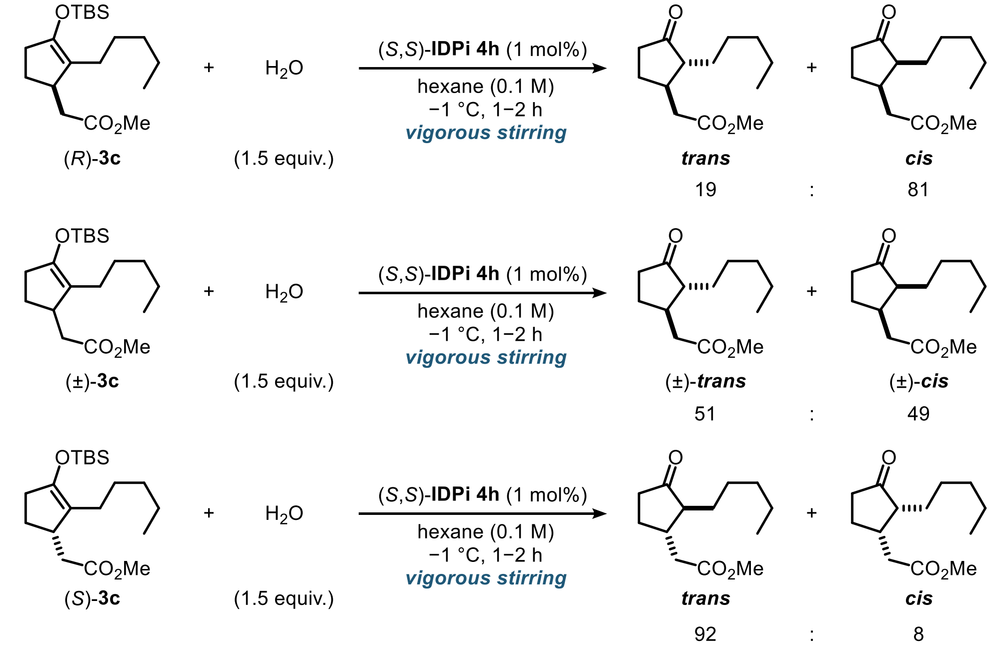

Figure S2. Control experiments. A) Influence of water and TBSOH on diastereoselectivity. B) Diatereoselective protodesilylation of different configured substrates with (*S*,*S*)-**IDPi 4h**.

Table S7. Reaction optimization for the *cis*-selective desilyl-protonation (H source screening)[a]

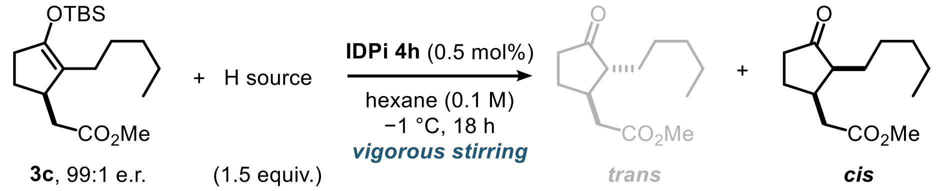

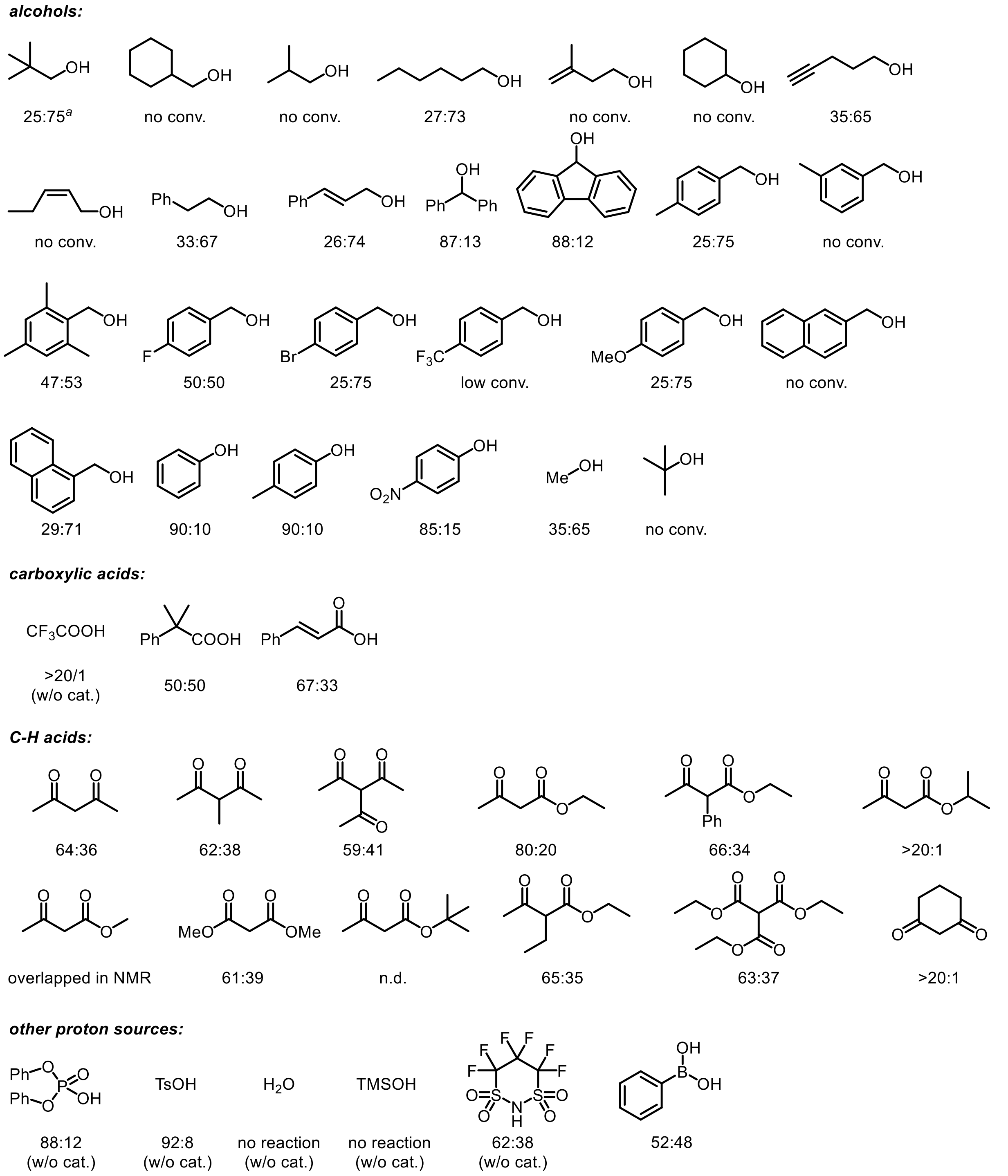

[a]Reactions were conducted with enol silane **3c** (0.05 mmol), proton source (1.5 equiv.), and **IDPi 4h** (1 mol%) in dry hexane (0.1 M) for 18 h at −1 °C. All conversions (conv.) and diastereomeric ratios (d.r.) were determined by ^{1}H NMR of the crude reaction mixture using CH_2Br_2 as an internal standard. d.r. = *trans*:*cis*.

4. Enantioselective Mukaiyama–Michael Addition and its Application

General Procedure for the Asymmetric Mukaiyama–Michael Addition:

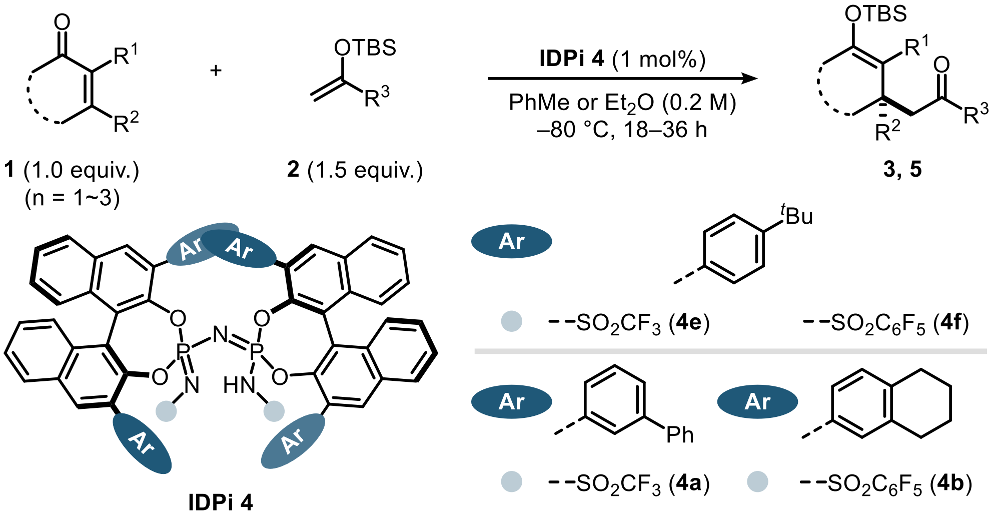

Unless specified otherwise, an oven-dried screw-cap vial equipped with a magnetic stirring bar was charged with **IDPi 4** (1 mol%) under Ar atmosphere. Freshly distilled solvent (PhMe or Et_2O, 0.2 M) and enol silane **2** (0.15 mmol, 1.5 equiv.) were added sequentially. The reaction mixture was stirred at room temperature for 15 min before fully cooled down to the indicated temperature (−50 to −80 °C). Subsequently, the corresponding enone **1** (0.1 mmol, 1.0 equiv.) was added slowly and the mixture was stirred for additional 18 or 36 h. Afterwards, the reaction was quenched with 5–10 μL of trimethylamine, then carefully warmed to room temperature, and directly purified via flash column chromatography using ^{i}hexane/Et_2O as eluents afford the desired adducts.

Alternatively, for solid enone **1**, an oven-dried screw-cap vial equipped with a magnetic stirring bar was charged with **IDPi 4** (1 mol%) and enone **1** (0.1 mmol, 1.0 equiv.) under Ar atmosphere, followed by the addition of freshly distilled solvent (PhMe or Et_2O, 0.2 M). Once completely dissolved, the mixture was transferred to a cryostat set at the indicated temperature (−50 to −80 °C). Enol silane **2** (0.15 mmol, 1.5 equiv.) was then added dropwise, and the reaction was stirred for 18–36 h. Afterwards, the reaction was quenched with 5–10 μL of trimethylamine, then carefully warmed to room temperature, and directly purified via flash column chromatography using ^{i}hexane/Et_2O as eluents to afford the desired adducts.

Note: *All enones and silyl nucleophiles needed to be further purified by recrystallization or distillation before use.*

Racemate Preparation for the Conjugate Addition:

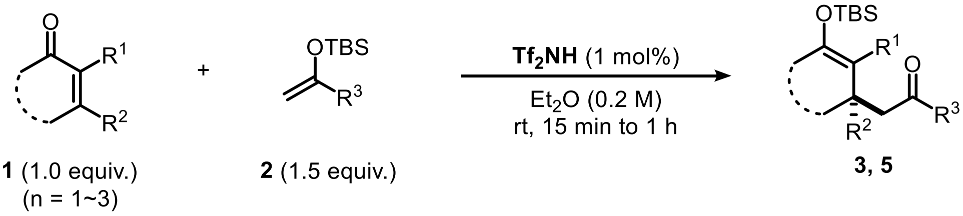

All racemic products were synthesized at room temperature for 15 min to 1 hour by using triflimide as the catalyst instead of **IDPi** with the same purification process.[5]

Methyl (*R*)-2-(3-((*tert*-butyldimethylsilyl)oxy)cyclopent-2-en-1-yl)acetate (3a)

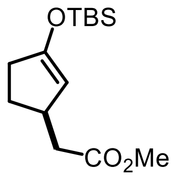

The reaction was conducted according to general procedure with enone **1a** (8.2 mg, 0.1 mmol, 1.0 equiv.), silyl ketene acetal **2a** (28.2 mg, 0.15 mmol, 1.5 equiv.) and **IDPi 4b** (1.5 mg, 1 mol%) in Et_2O at –80 °C for 18 h. Product **3a** as a colorless oil was afforded after purification via silica gel flash chromatography using ^{i}hexane/Et_2O (from 30:1 to 18:1 *v*/*v*) as eluent (26.0 mg, 96% yield).

^{1}H NMR (501 MHz, CD_2Cl_2): δ 4.63 (q, *J* = 1.9 Hz, 1H), 3.67 (s, 3H), 3.08–3.00 (m, 1H), 2.38–2.27 (m, 4H), 2.17–2.10 (m, 1H), 1.51 (dddd, *J* = 12.9, 8.9, 6.6, 5.2 Hz, 1H), 0.95 (s, 9H), 0.18 (s, 6H).

^{13}C NMR (126 MHz, CD_2Cl_2): δ 173.1, 156.0, 105.9, 51.1, 41.3, 38.6, 32.9, 28.0, 25.4, 18.0, –4.97, –5.02.

HRMS (GC-EI): calculated for $C_{14}H_{26}O_3Si$ ($[M]^+$): 270.1646, found: 270.1641.

$[\alpha]_D^{25}$ = –37.71 (*c* 0.35, $CHCl_3$)

TLC (SIL-plate): ^{i}hexane/EtOAc = 16/1, R_f = 0.60 [very slight spot, stained with $KMnO_4$].

HPLC (OJ-3R, CH_3CN:H_2O = 45:55, 1.0 mL/min, 220 nm, 298 K): t_R(minor) = 29.159 min, t_R(major) = 30.853 min. e.r. = 98:2 (96% ee).

Methyl (*R*)-2-(3-((*tert*-butyldimethylsilyl)oxy)-2-methylcyclopent-2-en-1-yl)acetate (3b)

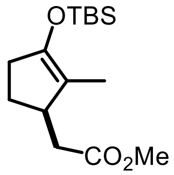

The reaction was conducted according to general procedure with enone **1b** (9.6 mg, 0.1 mmol, 1.0 equiv.), silyl ketene acetal **2a** (28.2 mg, 0.15 mmol, 1.5 equiv.) and **IDPi 4f** (1.6 mg, 1 mol%) in PhMe at –80 °C for 18 h. Product **3b** as a colorless oil was afforded after purification via silica gel flash chromatography using ^{i}hexane/Et_2O (from 30:1 to 18:1 *v*/*v*) as eluent (28.6 mg, 98% yield).

^{1}H NMR (501 MHz, CD_2Cl_2): δ 3.67 (s, 3H), 2.96–2.76 (m, 1H), 2.55 (dd, *J* = 14.9, 4.6 Hz, 1H), 2.36–2.21 (m, 2H), 2.16–2.06 (m, 2H), 1.55–1.49 (m, 4H), 0.97 (s, 9H), 0.15 (s, 6H).

^{13}C NMR (126 MHz, CD_2Cl_2): δ 173.4, 147.8, 114.3, 51.2, 41.8, 39.1, 32.2, 26.7, 25.4, 17.9, 9.7, –4.4.

HRMS (GC-EI): calculated for $C_{15}H_{28}O_3Si$ ($[M]^+$): 284.1802, found: 284.1798.

$[\alpha]_D^{25}$ = +33.79 (*c* 0.66, $CHCl_3$)

TLC (SIL-plate): ^{i}hexane/EtOAc = 16/1, R_f = 0.57 [very slight spot, stained with $KMnO_4$].

HPLC (OJ-3R, CH_3CN:H_2O = 60:40, 0.7 mL/min, 220 nm, 298 K): t_R(minor) = 9.736 min, t_R(major) = 10.043 min. e.r. = 99:1 (98% ee).

Methyl (*R*)-2-(3-((*tert*-butyldimethylsilyl)oxy)-2-pentylcyclopent-2-en-1-yl)acetate (3c)

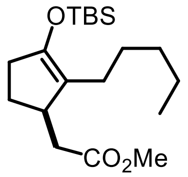

The reaction was conducted according to general procedure with enone **1c** (15.2 mg, 0.1 mmol, 1.0 equiv.), silyl ketene acetal **2a** (28.2 mg, 0.15 mmol, 1.5 equiv.) and **IDPi 4f** (1.6 mg, 1 mol%) in PhMe at –80 °C for 18 h. Product **3c** as a colorless oil was afforded after purification via silica gel flash chromatography using ^{i}hexane/Et_2O (from 30:1 to 18:1 *v*/*v*) as eluent (33.4 mg, 98% yield).

^{1}H NMR (501 MHz, CD_2Cl_2): δ 3.67 (s, 3H), 3.00–2.94 (m, 1H), 2.55 (dd, *J* = 14.9, 4.2 Hz, 1H), 2.37–2.31 (m, 1H), 2.28–2.21 (m, 2H), 2.13–2.05 (m, 2H), 1.79–1.72 (m, 1H), 1.55–1.49 (m, 1H), 1.46–1.26 (m, 6H), 0.97 (s, 9H), 0.92 (t, *J* = 7.1 Hz, 3H), 0.15 (d, *J* = 1.7 Hz, 6H).
^{13}C NMR (126 MHz, CD_2Cl_2): δ 173.5, 147.6, 118.8, 51.1, 39.4, 39.0, 32.2, 32.0, 27.3, 26.8, 25.4, 24.2, 22.5, 17.9, 13.9, –4.34, –4.38.
HRMS (GC-EI): calculated for $C_{19}H_{36}O_3Si$ ($[M]^+$): 340.2428, found: 340.2423.
$[\alpha]_D^{25}$ = +14.80 (*c* 1.00, $CHCl_3$)
TLC (SIL-plate): ^{i}hexane/EtOAc = 16/1, R_f = 0.56 [very slight spot, stained with $KMnO_4$].
HPLC (OJ-3R, $CH_3CN:H_2O$ = 60:40, 1.0 mL/min, 220 nm, 298 K): t_R(minor) = 11.446 min, t_R(major) = 13.700 min. e.r. = 99:1 (98% ee).

Methyl (*R*)-2-(3-((*tert*-butyldimethylsilyl)oxy)-2-hexylcyclopent-2-en-1-yl)acetate (3d)

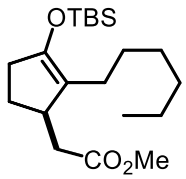

The reaction was conducted according to general procedure with enone **1d** (16.6 mg, 0.1 mmol, 1.0 equiv.), silyl ketene acetal **2a** (28.2 mg, 0.15 mmol, 1.5 equiv.) and **IDPi 4f** (1.6 mg, 1 mol%) in PhMe at –80 °C for 18 h. Product **3d** as a colorless oil was afforded after purification via silica gel flash chromatography using ^{i}hexane/Et_2O (from 30:1 to 18:1 *v*/*v*) as eluent (34.8 mg, 93% yield).
^{1}H NMR (501 MHz, CD_2Cl_2): δ 3.67 (s, 3H), 2.99–2.94 (m, 1H), 2.55 (dd, *J* = 15.0, 4.1 Hz, 1H), 2.37–2.30 (m, 1H), 2.28–2.22 (m, 2H), 2.12–2.04 (m, 2H), 1.79–1.72 (m, 1H), 1.52 (ddt, *J* = 12.8, 9.2, 5.0 Hz, 1H), 1.46–1.39 (m, 1H), 1.35–1.25 (m, 7H), 0.97 (s, 9H), 0.91 (t, *J* = 6.8 Hz, 3H), 0.15 (d, *J* = 1.7 Hz, 6H).
^{13}C NMR (126 MHz, CD_2Cl_2): δ 173.5, 147.6, 118.8, 51.2, 39.4, 39.0, 32.2, 31.8, 29.4, 27.6, 26.8, 25.4, 24.2, 22.7, 17.9, 13.9, –4.34, –4.38.
HRMS (GC-EI): calculated for $C_{20}H_{38}O_3Si$ ($[M]^+$): 354.2585, found: 354.2581.
$[\alpha]_D^{25}$ = +38.12 (*c* 0.34, $CHCl_3$)
TLC (SIL-plate): ^{i}hexane/EtOAc = 16/1, R_f = 0.58 [very slight spot, stained with $KMnO_4$].
HPLC (OJ-3R, $CH_3CN:H_2O$ = 60:40, 1.0 mL/min, 220 nm, 298 K): t_R(minor) = 14.466 min, t_R(major) = 17.230 min. e.r. = 99:1 (98% ee).

Methyl (*R*,*Z*)-2-(3-((*tert*-butyldimethylsilyl)oxy)-2-(pent-2-en-1-yl)cyclopent-2-en-1-yl) acetate (3e)

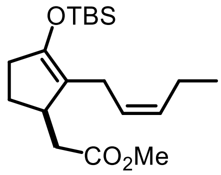

The reaction was conducted according to general procedure with enone **1e** (15.0 mg, 0.1 mmol, 1.0 equiv.), silyl ketene acetal **2a** (28.2 mg, 0.15 mmol, 1.5 equiv.) and **IDPi 4f** (1.6 mg, 1 mol%) in PhMe at –80 °C for 18 h. Product **3e** as a colorless oil was afforded after purification via silica gel flash chromatography using ^{i}hexane/Et_2O (from 30:1 to 18:1 *v*/*v*) as eluent (33.2 mg, 98% yield).
^{1}H NMR (501 MHz, CD_2Cl_2): δ 5.47–5.37 (m, 1H), 5.32–5.26 (m, 1H), 3.66 (s, 3H), 3.02–2.97 (m, 1H), 2.97–2.91 (m, 1H), 2.62–2.55 (m, 2H), 2.40–2.32 (m, 1H), 2.29–2.22 (m, 1H), 2.16–2.04 (m, 4H), 1.57–1.51 (m, 1H), 1.00 (t, *J* = 7.5 Hz, 3H), 0.97 (s, 9H), 0.17 (d, *J* = 2.2 Hz, 6H).
^{13}C NMR (126 MHz, CD_2Cl_2): δ 173.3, 147.8, 132.0, 126.3, 117.2, 51.1, 39.5, 38.9, 32.2, 26.7, 25.4, 22.5, 20.5, 18.0, 14.0, –4.4.

HRMS (GC-EI): calculated for $C_{19}H_{34}O_3Si$ ($[M]^+$): 338.2271, found: 338.2268.
$[\alpha]_D^{25}$ = –27.57 (*c* 0.91, $CHCl_3$)
TLC (SIL-plate): ^{i}hexane/EtOAc = 16/1, R_f = 0.4 [slight spot, stained with $KMnO_4$].
HPLC (OJ-3R, CH_3CN:H_2O = 60:40, 1.0 mL/min, 220 nm, 298 K): t_R(minor) = 9.056 min, t_R(major) = 10.452 min. e.r. = 99.5:0.5 (99% ee).

Methyl (*R*)-2-(3-((*tert*-butyldimethylsilyl)oxy)-2-(prop-1-en-2-yl)cyclopent-2-en-1-yl)acetate (3f)

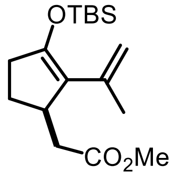

The reaction was conducted according to general procedure with enone **1f** (12.2 mg, 0.1 mmol, 1.0 equiv.), silyl ketene acetal **2a** (28.2 mg, 0.15 mmol, 1.5 equiv.) and **IDPi 4f** (1.6 mg, 1 mol%) in PhMe at –80 °C for 18 h. Product **3f** as a colorless oil was afforded after purification via silica gel flash chromatography using pentane/Et_2O (from 30:1 to 12:1 *v*/*v*) as eluent (28.9 mg, 93% yield).
^{1}H NMR (501 MHz, CD_2Cl_2): δ 4.81 (s, 1H), 4.76 (s, 1H), 3.67 (s, 3H), 3.22–3.16 (m, 1H), 2.64 (dd, *J* = 15.2, 3.1 Hz, 1H), 2.60–2.53 (m, 1H), 2.32 (ddd, *J* = 16.2, 9.7, 2.9 Hz, 1H), 2.14 (dd, *J* = 15.2, 10.7 Hz, 1H), 2.08–2.01 (m, 1H), 2.04 (s, 3H), 1.65–1.60 (m, 1H), 0.97 (s, 9H), 0.21 (d, *J* = 3.2 Hz, 6H).
^{13}C NMR (126 MHz, CD_2Cl_2): δ 173.5, 151.0, 139.0, 118.6, 110.8, 51.14 39.8, 38.2, 33.5, 26.1, 25.6, 25.5, 22.7, 18.1, –3.8, –3.9.
HRMS (GC-EI): calculated for $C_{17}H_{30}O_3Si$ ($[M]^+$): 310.1959, found: 310.1954.
$[\alpha]_D^{25}$ = +40.38 (*c* 0.43, $CHCl_3$)
TLC (SIL-plate): pentane/EtOAc = 12/1, R_f = 0.55.
HPLC (OJ-3R, CH_3CN:H_2O = 60:40, 1.0 mL/min, 220 nm, 298 K): t_R(minor) = 8.165 min, t_R(major) = 9.875 min. e.r. = 99.2:0.8 (98.4% ee).

Methyl (*R*)-2-(3-((*tert*-butyldimethylsilyl)oxy)-2-(cyclohex-1-en-1-yl)cyclopent-2-en-1-yl) acetate (3g)

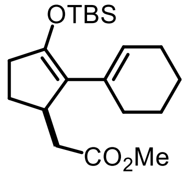

The reaction was conducted according to general procedure with enone **1g** (16.2 mg, 0.1 mmol, 1.0 equiv.), silyl ketene acetal **2a** (28.2 mg, 0.15 mmol, 1.5 equiv.) and **IDPi 4f** (1.6 mg, 1 mol%) in PhMe at –80 °C for 18 h. Product **3g** as a colorless oil was afforded after purification via silica gel flash chromatography using pentane/Et_2O (from 30:1 to 12:1 *v*/*v*) as eluent (33.7 mg, 96% yield).
^{1}H NMR (501 MHz, CD_2Cl_2): δ 5.60–5.54 (m, 1H), 3.66 (s, 3H), 3.19–3.12 (m, 1H), 2.58 (dd, *J* = 15.2, 3.4 Hz, 1H), 2.54–2.43 (m, 2H), 2.29 (ddd, *J* = 15.9, 9.6, 3.7 Hz, 1H), 2.14–2.01 (m, 5H), 1.70–1.53 (m, 6H), 0.96 (s, 9H), 0.17 (d, *J* = 2.4 Hz, 6H).
^{13}C NMR (126 MHz, CD_2Cl_2): δ 173.6, 148.3, 132.2, 123.7, 120.0, 51.1, 39.7, 38.8, 33.4, 27.9, 26.3, 25.6, 25.5, 23.0, 22.4, 18.0, –4.0, –4.1.
HRMS (GC-EI): calculated for $C_{20}H_{34}O_3Si$ ($[M]^+$): 350.2271, found: 350.2268.
$[\alpha]_D^{25}$ = +49.69 (*c* 0.32, $CHCl_3$)
TLC (SIL-plate): pentane/EtOAc = 12/1, R_f = 0.58.
HPLC (OJ-3R, CH_3CN:H_2O = 70:30, 1.0 mL/min, 220 nm, 298 K): t_R(minor) = 5.907 min, t_R(major) = 6.367 min. e.r. = 99:1 (98% ee).

Methyl (*R*)-2-(3-((*tert*-butyldimethylsilyl)oxy)-2-(1-phenylvinyl)cyclopent-2-en-1-yl)acetate (3h)

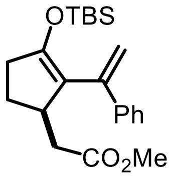

The reaction was conducted according to general procedure with enone **1h** (18.4 mg, 0.1 mmol, 1.0 equiv.), silyl ketene acetal **2a** (28.2 mg, 0.15 mmol, 1.5 equiv.) and **IDPi 4f** (1.6 mg, 1 mol%) in PhMe at –80 °C for 18 h. Product **3h** as a colorless oil was afforded after purification via silica gel flash chromatography using pentane/Et_2O (from 30:1 to 12:1 *v*/*v*) as eluent (33.2 mg, 89% yield).

^{1}H NMR (501 MHz, CD_2Cl_2): δ 7.38–7.35 (m, 2H), 7.34–7.30 (m, 2H), 7.29–7.25 (m, 1H), 5.45 (d, *J* = 1.8 Hz, 1H), 5.20 (d, *J* = 1.9 Hz, 1H), 3.60 (s, 3H), 3.24–3.18 (m, 1H), 2.57–2.42 (m, 3H), 2.23 (dddd, *J* = 13.0, 9.3, 8.3, 5.9 Hz, 1H), 2.15 (dd, *J* = 15.4, 10.4 Hz, 1H), 1.64 (dddd, *J* = 13.0, 9.2, 5.7, 4.7 Hz, 1H), 0.78 (s, 9H), 0.07 (d, *J* = 2.6 Hz, 6H).

^{13}C NMR (126 MHz, CD_2Cl_2): δ 173.2, 151.6, 143.2, 140.9, 127.9, 127.1, 127.0, 118.1, 114.8, 51.1, 40.5, 39.0, 33.4, 26.6, 25.2, 17.7, –4.24, –4.29.

HRMS (GC-EI): calculated for $C_{22}H_{32}O_3Si$ ($[M]^+$): 372.2115, found: 372.2113.

$[\alpha]_D^{25}$ = +25.35 (*c* 0.78, $CHCl_3$)

TLC (SIL-plate): pentane/EtOAc = 7/1, R_f = 0.61.

HPLC (IB-3R, CH_3CN:H_2O = 60:40, 1.0 mL/min, 220 nm, 298 K): t_R(minor) = 18.050 min, t_R(major) = 19.232 min. e.r. = 93:7 (86% ee).

Methyl (*R*)-2-(3-((*tert*-butyldimethylsilyl)oxy)-2-(2-methylprop-1-en-1-yl)cyclopent-2-en-1-yl)acetate (3i)

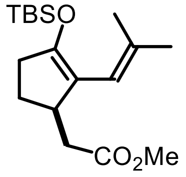

The reaction was conducted according to general procedure with enone **1i** (13.6 mg, 0.1 mmol, 1.0 equiv.), silyl ketene acetal **2a** (28.2 mg, 0.15 mmol, 1.5 equiv.) and **IDPi 4f** (1.6 mg, 1 mol%) in PhMe at –80 °C for 18 h. Product **3i** as a colorless oil was afforded after purification via silica gel flash chromatography using pentane/Et_2O (from 30:1 to 12:1 *v*/*v*) as eluent (30.8 mg, 95% yield).

^{1}H NMR (501 MHz, CD_2Cl_2): δ 5.65–5.56 (m, 1H), 3.66 (s, 3H), 3.24–3.18 (m, 1H), 2.53 (dd, *J* = 15.2, 3.8 Hz, 1H), 2.45–2.37 (m, 1H), 2.31–2.24 (m, 1H), 2.14–2.06 (m, 2H), 1.80 (s, 3H), 1.72 (s, 3H), 1.62–1.56 (m, 2H), 0.95 (s, 9H), 0.14 (s, 6H).

^{13}C NMR (126 MHz, CD_2Cl_2): δ 173.4, 149.7, 132.9, 118.1, 117.7, 51.1, 40.6, 39.4, 32.2, 26.7, 26.3, 25.4, 19.8, 18.0, –4.4, –4.5.

HRMS (GC-EI): calculated for $C_{18}H_{32}O_3Si$ ($[M]^+$): 324.2115, found: 324.2111.

$[\alpha]_D^{25}$ = +32.98 (*c* 0.47, $CHCl_3$)

TLC (SIL-plate): pentane/EtOAc = 12/1, R_f = 0.58.

HPLC (OJ-3R, CH_3CN:H_2O = 60:40, 1.0 mL/min, 220 nm, 298 K): t_R(minor) = 8.082 min, t_R(major) = 8.763 min. e.r. = 99.5:0.5 (99% ee).

Methyl (*R*)-2-(3-((*tert*-butyldimethylsilyl)oxy)-2-(phenylethynyl)cyclopent-2-en-1-yl)acetate (3j)

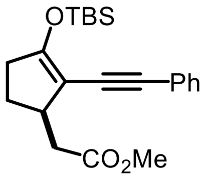

The reaction was conducted according to general procedure with enone **1j** (18.2 mg, 0.1 mmol, 1.0 equiv.), silyl ketene acetal **2a** (28.2 mg, 0.15 mmol, 1.5 equiv.) and **IDPi 4f** (1.6 mg, 1 mol%) in Et_2O at –80 °C for 18 h. Product **3j** as a colorless oil was afforded after purification via silica gel flash

chromatography using pentane/Et_2O (from 30:1 to 12:1 *v*/*v*) as eluent (35.9 mg, 97% yield).
^{1}H NMR (501 MHz, CD_2Cl_2): δ 7.47–7.38 (m, 2H), 7.37–7.27 (m, 3H), 3.69 (s, 3H), 3.23–3.16 (m, 1H), 2.81 (dd, *J* = 15.5, 4.8 Hz, 1H), 2.46–2.42 (m, 2H), 2.31 (dd, *J* = 15.5, 9.6 Hz, 1H), 2.28–2.20 (m, 1H), 1.65–1.58 (m, 1H), 1.02 (s, 9H), 0.29 (s, 6H).
^{13}C NMR (126 MHz, CD_2Cl_2): δ 172.9, 161.2, 131.0, 128.3, 127.5, 124.1, 102.0, 93.8, 84.9, 51.3, 40.9, 39.8, 33.2, 27.2, 25.3, 18.1, –4.28, –4.31.
HRMS (GC-EI): calculated for $C_{22}H_{30}O_3Si$ ($[M]^+$): 370.1959, found: 370.1956.
$[\alpha]_D^{25}$ = +28.50 (*c* 0.37, $CHCl_3$)
TLC (SIL-plate): pentane/EtOAc = 12/1, R_f = 0.42.
HPLC (OJ-3R, CH_3CN:H_2O = 60:40, 1.0 mL/min, 220 nm, 298 K): t_R(minor) = 10.949 min, t_R(major) = 13.032 min. e.r. = 93:7 (86% ee).

Methyl (*R*)-2-(3-((*tert*-butyldimethylsilyl)oxy)-2-(pent-1-yn-1-yl)cyclopent-2-en-1-yl)acetate (3k)

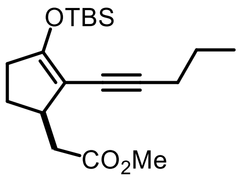

The reaction was conducted according to general procedure with enone **1k** (14.8 mg, 0.1 mmol, 1.0 equiv.), silyl ketene acetal **2a** (28.2 mg, 0.15 mmol, 1.5 equiv.) and **IDPi 4f** (1.6 mg, 1 mol%) in PhMe at –80 °C for 18 h. Product **3k** as a colorless oil was afforded after purification via silica gel flash chromatography using pentane/Et_2O (from 30:1 to 12:1 *v*/*v*) as eluent (32.3 mg, 96% yield).
^{1}H NMR (501 MHz, CD_2Cl_2): δ 3.68 (s, 3H), 3.06–2.98 (m, 1H), 2.72 (dd, *J* = 15.4, 4.4 Hz, 1H), 2.37–2.32 (m, 4H), 2.22–2.11 (m, 2H), 1.60–1.50 (m, 3H), 1.02 (t, *J* = 7.4 Hz, 3H), 0.98 (s, 9H), 0.23 (s, 6H).
^{13}C NMR (126 MHz, CD_2Cl_2): δ 173.1, 158.9, 102.5, 94.5, 75.2, 51.2, 41.0, 39.7, 32.9, 27.0, 25.3, 22.5, 21.6, 18.0, 13.3, –4.36, –4.39.
HRMS (GC-EI): calculated for $C_{19}H_{32}O_3Si$ ($[M]^+$): 336.2115, found: 336.2112.
$[\alpha]_D^{25}$ = +37.80 (*c* 0.58, $CHCl_3$)
TLC (SIL-plate): pentane/EtOAc = 12/1, R_f = 0.4.
HPLC (OJ-3R, CH_3CN:H_2O = 60:40, 1.0 mL/min, 220 nm, 298 K): t_R(minor) = 7.709 min, t_R(major) = 8.063 min. e.r. = 95:5 (90% ee).

Methyl (*R*)-2-(3-((*tert*-butyldimethylsilyl)oxy)-2-(naphthalen-2-yl)cyclopent-2-en-1-yl) acetate (3l)

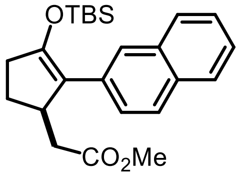

The reaction was conducted according to general procedure with enone **1l** (20.8 mg, 0.1 mmol, 1.0 equiv.), silyl ketene acetal **2a** (28.2 mg, 0.15 mmol, 1.5 equiv.) and **IDPi 4f** (1.6 mg, 1 mol%) in PhMe at –80 °C for 18 h. Product **3l** as a colorless oil was afforded after purification via silica gel flash chromatography using ^{i}hexane/Et_2O (from 30:1 to 12:1 *v*/*v*) as eluent (34.9 mg, 88% yield).
^{1}H NMR (501 MHz, CD_2Cl_2): δ 7.96 (s, 1H), 7.85–7.78 (m, 4H), 7.49–7.41 (m, 2H), 3.72–3.67 (m, 1H), 3.64 (s, 3H), 2.73 (dddd, *J* = 16.3, 9.3, 7.3, 2.3 Hz, 1H), 2.67 (dd, *J* = 15.4, 3.4 Hz, 1H), 2.51 (ddd, *J* = 16.1, 9.6, 3.9 Hz, 1H), 2.29 (ddt, *J* = 13.0, 9.5, 7.7 Hz, 1H), 2.21 (dd, *J* = 15.4, 10.6 Hz, 1H), 1.76 (ddt, *J* = 12.6, 8.8, 3.6 Hz, 1H), 0.99 (s, 9H), 0.26 (s, 3H), 0.15 (s, 3H).

^{13}C NMR (126 MHz, CD_2Cl_2): δ 173.4, 151.8, 133.6, 132.9, 131.7, 127.7, 127.4, 127.2, 126.2, 125.7, 125.3, 125.1, 117.0, 51.2, 39.8, 38.9, 33.8, 26.3, 25.5, 18.1, –3.9, –4.0.
HRMS (GC-EI): calculated for $C_{24}H_{32}O_3Si$ ($[M]^+$): 396.2115, found: 396.2114.
$[\alpha]_D^{25}$ = –40.40 (*c* 0.50, $CHCl_3$)
TLC (SIL-plate): ^{i}hexane/EtOAc = 6/1, R_f = 0.65.
HPLC (OJ-3R, CH_3CN:H_2O = 60:40, 1.0 mL/min, 220 nm, 298 K): t_R(minor) = 20.270 min, t_R(major) = 23.598 min. e.r. = 97:3 (94% ee).

Methyl (*R*)-2-(3-((*tert*-butyldimethylsilyl)oxy)-2-phenylcyclopent-2-en-1-yl)acetate (3m)

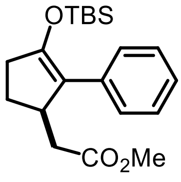

The reaction was conducted according to general procedure with enone **1m** (15.8 mg, 0.1 mmol, 1.0 equiv.), silyl ketene acetal **2a** (28.2 mg, 0.15 mmol, 1.5 equiv.) and **IDPi 4f** (1.6 mg, 1 mol%) in PhMe at –80 °C for 18 h. Product **3m** as a colorless oil was afforded after purification via silica gel flash chromatography using ^{i}hexane/Et_2O (from 30:1 to 12:1 *v*/*v*) as eluent (32.2 mg, 93% yield).
^{1}H NMR (501 MHz, CD_2Cl_2): δ 7.65–7.48 (m, 2H), 7.38–7.29 (m, 2H), 7.23–7.09 (m, 1H), 3.64 (s, 3H), 3.58–3.52 (m, 1H), 2.66 (dddd, *J* = 16.1, 9.2, 7.2, 2.3 Hz, 1H), 2.60 (dd, *J* = 15.3, 3.4 Hz, 1H), 2.46 (dddd, *J* = 16.1, 9.7, 4.0, 0.9 Hz, 1H), 2.26–2.19 (m, 1H), 2.16 (dd, *J* = 15.3, 10.6 Hz, 1H), 1.73–1.67 (m, 1H), 0.96 (s, 9H), 0.22 (s, 3H), 0.13 (s, 3H).
^{13}C NMR (126 MHz, CD_2Cl_2): δ 173.4, 151.0, 135.3, 127.9, 127.3, 125.4, 117.0, 51.2, 39.8, 38.8, 33.6, 26.2, 25.5, 18.0, –4.0.
HRMS (GC-EI): calculated for $C_{20}H_{30}O_3Si$ ($[M]^+$): 346.1959, found: 346.1956.
$[\alpha]_D^{25}$ = –39.68 (*c* 0.25, $CHCl_3$)
TLC (SIL-plate): ^{i}hexane/EtOAc = 12/1, R_f = 0.52.
HPLC (OJ-3R, CH_3CN:H_2O = 60:40, 1.0 mL/min, 220 nm, 298 K): t_R(minor) = 9.719 min, t_R(major) = 10.893 min. e.r. = 97:3 (94% ee).

Methyl (*R*)-2-(3-((*tert*-butyldimethylsilyl)oxy)-2-(4- (trifluoromethyl)phenyl)cyclopent-2-en-1-yl)acetate (3n)

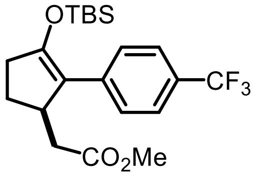

The reaction was conducted according to general procedure with enone **1n** (22.6 mg, 0.1 mmol, 1.0 equiv.), silyl ketene acetal **2a** (28.2 mg, 0.15 mmol, 1.5 equiv.) and **IDPi 4f** (1.6 mg, 1 mol%) in PhMe at –80 °C for 36 h. Product **3n** as a colorless oil was afforded after purification via silica gel flash chromatography using ^{i}hexane/Et_2O (from 30:1 to 12:1 *v*/*v*) as eluent (37.7 mg, 91% yield).
^{1}H NMR (501 MHz, CD_2Cl_2): δ 7.71 (d, *J* = 8.2 Hz, 2H), 7.59 (d, *J* = 8.2 Hz, 2H), 3.64 (s, 3H), 3.61–3.55 (m, 1H), 2.74–2.67 (m, 1H), 2.57 (dd, *J* = 15.3, 3.4 Hz, 1H), 2.52–2.46 (m, 1H), 2.32–2.22 (m, 1H), 2.19 (dd, *J* = 15.4, 10.5 Hz, 1H), 1.74 (ddt, *J* = 12.7, 9.0, 3.5 Hz, 1H), 0.97 (s, 9H), 0.25 (s, 3H), 0.17 (s, 3H).
^{13}C NMR (126 MHz, CD_2Cl_2): δ 173.1, 153.7, 139.1, 127.2, 126.7 (q, *J* = 32.8 Hz), 124.6 (q, *J* = 272.2 Hz), 124.8 (q, *J* = 3.8 Hz), 115.8, 51.2, 39.5, 38.6, 33.7, 26.2, 25.4, 18.0, 3.9.
^{19}F NMR (471 MHz, CD_2Cl_2) δ –62.55.
HRMS (ESI, positive ions): calculated for $C_{21}H_{29}F_3NaO_3Si$ ($[M+Na]^+$): 437.1730, found: 437.1732.

$[\alpha]_D^{25}$ = –4.65 (*c* 0.69, $CHCl_3$)
TLC (SIL-plate): ^{i}hexane/EtOAc = 8/1, R_f = 0.62.
HPLC (OJ-3R, CH_3CN:H_2O = 60:40, 1.0 mL/min, 220 nm, 298 K): t_R(minor) = 12.304 min, t_R(major) = 15.667 min. e.r. = 94:6 (88% ee).

Methyl (*R*)-2-(3-((*tert*-butyldimethylsilyl)oxy)-2-(4-chlorophenyl)cyclopent-2-en-1-yl)acetate (3o)

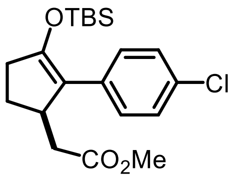

The reaction was conducted according to general procedure with enone **1o** (19.2 mg, 0.1 mmol, 1.0 equiv.), silyl ketene acetal **2a** (28.2 mg, 0.15 mmol, 1.5 equiv.) and **IDPi 4f** (1.6 mg, 1 mol%) in PhMe at –80 °C for 36 h. Product **3o** as a colorless oil was afforded after purification via silica gel flash chromatography using ^{i}hexane/Et_2O (from 30:1 to 12:1 *v*/*v*) as eluent (34.7 mg, 94% yield).
^{1}H NMR (501 MHz, CD_2Cl_2): δ 7.52 (d, *J* = 8.6 Hz, 2H), 7.30 (d, *J* = 8.6 Hz, 2H), 3.64 (s, 3H), 3.54–3.48 (m, 1H), 2.66 (dddd, *J* = 16.2, 9.3, 7.4, 2.3 Hz, 1H), 2.56 (dd, *J* = 15.3, 3.4 Hz, 1H), 2.45 (dddd, *J* = 16.2, 9.7, 3.9, 0.9 Hz, 1H), 2.26–2.20 (m, 1H), 2.16 (dd, *J* = 15.3, 10.5 Hz, 1H), 1.71 (ddt, *J* = 12.7, 8.9, 3.6 Hz, 1H), 0.96 (s, 9H), 0.23 (s, 3H), 0.15 (s, 3H).
^{13}C NMR (126 MHz, CD_2Cl_2): δ 173.2, 151.8, 133.9, 130.5, 128.6, 128.0, 115.9, 51.2, 39.7, 38.7, 33.6, 26.2, 25.4, 18.0, –4.0.
HRMS (GC-EI): calculated for $C_{20}H_{29}O_3ClSi$ ($[M]^+$): 380.1569, found: 380.1566.
$[\alpha]_D^{25}$ = –72.42 (*c* 0.42, $CHCl_3$)
TLC (SIL-plate): ^{i}hexane/EtOAc = 8/1, R_f = 0.63.
HPLC (OJ-3R, CH_3CN:H_2O = 60:40, 1.0 mL/min, 220 nm, 298 K): t_R(minor) = 14.989 min, t_R(major) = 17.789 min. e.r. = 96:4 (92% ee).

Methyl (*R*)-2-(3-((*tert*-butyldimethylsilyl)oxy)-2-(p-tolyl)cyclopent-2-en-1-yl)acetate (3p)

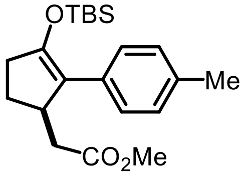

The reaction was conducted according to general procedure with enone **1p** (17.2 mg, 0.1 mmol, 1.0 equiv.), silyl ketene acetal **2a** (28.2 mg, 0.15 mmol, 1.5 equiv.) and **IDPi 4f** (1.6 mg, 1 mol%) in PhMe at –80 °C for 18 h. Product **3p** as a colorless oil was afforded after purification via silica gel flash chromatography using ^{i}hexane/Et_2O (from 30:1 to 12:1 *v*/*v*) as eluent (34.6 mg, 96% yield).
^{1}H NMR (501 MHz, CD_2Cl_2): δ 7.45 (d, *J* = 8.3 Hz, 2H), 7.15 (d, *J* = 8.2 Hz, 2H), 3.64 (s, 3H), 3.55–3.49 (m, 1H), 2.65 (dddd, *J* = 16.2, 9.3, 7.3, 2.3 Hz, 1H), 2.58 (dd, *J* = 15.3, 3.3 Hz, 1H), 2.44 (ddd, *J* = 16.0, 9.7, 3.9 Hz, 1H), 2.34 (s, 3H), 2.24–2.18 (m, 1H), 2.14 (dd, *J* = 15.3, 10.7 Hz, 1H), 1.69 (ddt, *J* = 12.7, 8.9, 3.6 Hz, 1H), 0.96 (s, 9H), 0.21 (s, 3H), 0.13 (s, 3H).
^{13}C NMR (126 MHz, CD_2Cl_2): δ 173.4, 150.3, 135.0, 132.2, 128.6, 127.1, 116.9, 51.2, 39.8, 38.8, 33.6, 26.2, 25.5, 20.8, 18.0, –3.97, –3.99.
HRMS (GC-EI): calculated for $C_{21}H_{32}O_3Si$ ($[M]^+$): 360.2115, found: 360.2114.
$[\alpha]_D^{25}$ = –14.70 (*c* 0.38, $CHCl_3$)
TLC (SIL-plate): ^{i}hexane/EtOAc = 8/1, R_f = 0.47.
HPLC (OJ-3R, CH_3CN:H_2O = 60:40, 1.0 mL/min, 220 nm, 298 K): t_R(minor) = 11.239 min, t_R(major) = 17.747 min. e.r. = 98:2 (96% ee).

Methyl (*R*)-2-(3-((*tert*-butyldimethylsilyl)oxy)-2-(4-methoxyphenyl)cyclopent-2-en-1-yl) acetate (3q)

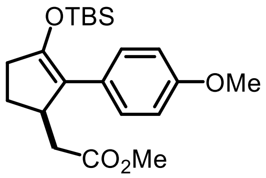

The reaction was conducted according to general procedure with enone **1q** (18.8 mg, 0.1 mmol, 1.0 equiv.), silyl ketene acetal **2a** (28.2 mg, 0.15 mmol, 1.5 equiv.) and **IDPi 4f** (1.6 mg, 1 mol%) in PhMe at –50 °C for 36 h. Product **3q** as a colorless oil was afforded after purification via silica gel flash chromatography using ^{i}hexane/Et_2O (from 30:1 to 10:1 *v*/*v*) as eluent (34.6 mg, 92% yield).

^{1}H NMR (501 MHz, CD_2Cl_2): δ 7.49 (d, *J* = 8.9 Hz, 2H), 6.88 (d, *J* = 8.9 Hz, 2H), 3.82 (s, 3H), 3.64 (s, 3H), 3.52–3.47 (m, 1H), 2.68–2.61 (m, 1H), 2.57 (dd, *J* = 15.3, 3.3 Hz, 1H), 2.43 (dddd, *J* = 16.0, 9.7, 3.8, 0.9 Hz, 1H), 2.24–2.16 (m, 1H), 2.17–2.11 (m, 1H), 1.72–1.65 (m, 1H), 0.96 (s, 9H), 0.21 (s, 3H), 0.13 (s, 3H).

^{13}C NMR (126 MHz, CD_2Cl_2): δ 173.4, 157.4, 149.4, 128.4, 127.8, 116.6, 113.3, 55.1, 51.2, 39.9, 38.8, 33.5, 26.2, 25.5, 18.0, –3.99, –4.01.

HRMS (GC-EI): calculated for $C_{21}H_{32}O_4Si$ ($[M]^+$): 376.2064, found: 376.2062.

$[\alpha]_D^{25}$ = –18.42 (*c* 0.15, $CHCl_3$)

TLC (SIL-plate): ^{i}hexane/EtOAc = 6/1, R_f = 0.62.

HPLC (OJ-3R, CH_3CN:H_2O = 60:40, 1.0 mL/min, 220 nm, 298 K): t_R(minor) = 10.806 min, t_R(major) = 14.083 min. e.r. = 96:4 (92% ee).

Methyl (*R*)-2-(3-((*tert*-butyldimethylsilyl)oxy)-2-(furan-2-yl)cyclopent-2-en-1-yl)acetate (3r)

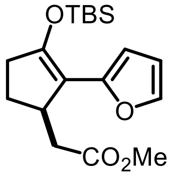

The reaction was conducted according to general procedure with enone **1r** (14.8 mg, 0.1 mmol, 1.0 equiv.), silyl ketene acetal **2a** (28.2 mg, 0.15 mmol, 1.5 equiv.) and **IDPi 4f** (1.6 mg, 1 mol%) in PhMe at –80 °C for 18 h. Product **3r** as a colorless oil was afforded after purification via silica gel flash chromatography using ^{i}hexane/Et_2O (from 30:1 to 12:1 *v*/*v*) as eluent (29.9 mg, 89% yield).

^{1}H NMR (501 MHz, CD_2Cl_2): δ 7.34 (d, *J* = 1.9 Hz, 1H), 6.47–6.43 (m, 2H), 3.68 (s, 3H), 3.44 (tt, *J* = 8.1, 3.0 Hz, 1H), 2.82 (dd, *J* = 15.2, 3.5 Hz, 1H), 2.68–2.61 (m, 1H), 2.41 (ddd, *J* = 16.4, 9.7, 3.2 Hz, 1H), 2.27 (dd, *J* = 15.3, 10.6 Hz, 1H), 2.23–2.16 (m, 1H), 1.72 (ddt, *J* = 13.0, 8.7, 2.9 Hz, 1H), 1.01 (s, 9H), 0.24 (d, *J* = 5.9 Hz, 6H).

^{13}C NMR (126 MHz, CD_2Cl_2): δ 173.2, 151.1, 150.8, 139.4, 110.9, 110.0, 105.8, 51.2, 39.2, 38.8, 32.9, 26.7, 25.5, 18.1, –4.0.

HRMS (GC-EI): calculated for $C_{18}H_{28}O_4Si$ ($[M]^+$): 336.1751, found: 336.1747.

$[\alpha]_D^{25}$ = +33.77 (*c* 0.31, $CHCl_3$)

TLC (SIL-plate): ^{i}hexane/EtOAc = 6/1, R_f = 0.52 [slight spot, stained with $KMnO_4$].

HPLC (OJ-3R, CH_3CN:H_2O = 60:40, 1.0 mL/min, 220 nm, 298 K): t_R(minor) = 7.870 min, t_R(major) = 9.377 min. e.r. = 91:9 (82% ee).

Methyl (*R*)-2-(3-((*tert*-butyldimethylsilyl)oxy)-1-methylcyclopent-2-en-1-yl)acetate (3s)

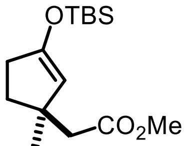

The reaction was conducted according to general procedure with enone **1s** (9.6 mg, 0.1 mmol, 1.0 equiv.), silyl ketene acetal **2a** (28.2 mg, 0.15 mmol, 1.5 equiv.) and **IDPi 4f** (1.6 mg, 1 mol%) in PhMe at –80 °C for 18 h. Product **3s** as a colorless oil was afforded after purification via silica gel flash chromatography

using ^{i}hexane/Et_2O (from 30:1 to 18:1 *v*/*v*) as eluent (24.7 mg, 87% yield).
^{1}H NMR (501 MHz, CD_2Cl_2): δ 4.64 (s, 1H), 3.65 (s, 3H), 2.35 (d, *J* = 1.9 Hz, 2H), 2.34–2.30 (m, 2H), 1.97 (ddd, *J* = 12.8, 8.2, 6.5 Hz, 1H), 1.69 (ddd, *J* = 12.8, 8.4, 6.3 Hz, 1H), 1.15 (s, 3H), 0.95 (s, 9H), 0.19 (d, *J* = 1.0 Hz, 6H).
^{13}C NMR (126 MHz, CD_2Cl_2): δ 172.4, 154.2, 111.4, 50.9, 46.4, 44.1, 35.2, 32.7, 27.5, 25.4, 18.0, –4.99, –5.06.
HRMS (GC-EI): calculated for $C_{15}H_{28}O_3Si$ ($[M]^+$): 284.1802, found: 284.1797.
$[\alpha]_D^{25}$ = +9.84 (*c* 0.37, $CHCl_3$)
TLC (SIL-plate): ^{i}hexane/EtOAc = 16/1, R_f = 0.55 [very slight spot, stained with $KMnO_4$].
HPLC (OJ-3R, CH_3OH:H_2O = 75:25, 1.0 mL/min, 220 nm, 298 K): t_R(minor) = 14.528 min, t_R(major) = 15.866 min. e.r. = 96:4 (92% ee).

Methyl (*R*)-2-(3-((*tert*-butyldimethylsilyl)oxy)cyclohex-2-en-1-yl)acetate (3t)

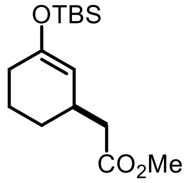

The reaction was conducted according to general procedure with enone **1t** (9.6 mg, 0.1 mmol, 1.0 equiv.), silyl ketene acetal **2a** (28.2 mg, 0.15 mmol, 1.5 equiv.) and **IDPi 4f** (1.6 mg, 1 mol%) in PhMe at –80 °C for 18 h. Product **3t** as a colorless oil was afforded after purification via silica gel flash chromatography using ^{i}hexane/Et_2O (from 30:1 to 18:1 *v*/*v*) as eluent (27.3 mg, 96% yield).
^{1}H NMR (501 MHz, CD_2Cl_2): δ 4.82–4.75 (m, 1H), 3.68 (s, 3H), 2.70–2.62 (m, 1H), 2.28 (d, *J* = 7.3 Hz, 2H), 2.05–1.97 (m, 2H), 1.81–1.74 (m, 2H), 1.66–1.59 (m, 1H), 1.23–1.16 (m, 1H), 0.95 (s, 9H), 0.16 (s, 6H).
^{13}C NMR (126 MHz, CD_2Cl_2): δ 173.0, 151.7, 107.6, 51.2, 41.2, 31.8, 29.7, 28.5, 25.4, 21.2, 17.9, –4.7, –4.8.
HRMS (GC-EI): calculated for $C_{15}H_{28}O_3Si$ ($[M]^+$): 284.1802, found: 284.1798.
$[\alpha]_D^{25}$ = +33.74 (*c* 0.17, $CHCl_3$)
TLC (SIL-plate): ^{i}hexane/EtOAc = 16/1, R_f = 0.60 [very slight spot, stained with $KMnO_4$].
HPLC (OD-3R, CH_3CN:H_2O = 50:50, 1.0 mL/min, 220 nm, 298 K): t_R(minor) = 25.581 min, t_R(major) = 24.456 min. e.r. = 93:7 (86% ee).

Methyl (*R*)-2-(3-((*tert*-butyldimethylsilyl)oxy)-2-methylcyclohex-2-en-1-yl)acetate (3u)

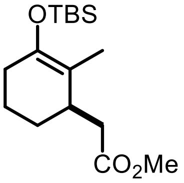

The reaction was conducted according to general procedure with enone **1u** (11.0 mg, 0.1 mmol, 1.0 equiv.), silyl ketene acetal **2a** (28.2 mg, 0.15 mmol, 1.5 equiv.) and **IDPi 4f** (1.6 mg, 1 mol%) in PhMe at –80 °C for 18 h. Product **3u** as a colorless oil was afforded after purification via silica gel flash chromatography using ^{i}hexane/Et_2O (from 30:1 to 18:1 *v*/*v*) as eluent (27.8 mg, 93% yield).
^{1}H NMR (501 MHz, CD_2Cl_2): δ 3.68 (s, 3H), 2.59–2.51 (m, 2H), 2.20 (dd, *J* = 14.4, 9.7 Hz, 1H), 2.07–1.98 (m, 2H), 1.75–1.62 (m, 3H), 1.61 (dt, *J* = 1.9, 1.2 Hz, 3H), 1.48–1.42 (m, 1H), 0.97 (s, 9H), 0.15 (s, 6H).
^{13}C NMR (126 MHz, CD_2Cl_2): δ 173.6, 145.0, 113.1, 51.2, 38.2, 36.4, 30.4, 27.8, 25.6, 19.8, 18.1, 14.2, –4.1, –4.2.
HRMS (GC-EI): calculated for $C_{16}H_{30}O_3Si$ ($[M]^+$): 298.1959, found: 298.1953.
$[\alpha]_D^{25}$ = –9.72 (*c* 0.14, $CHCl_3$)
TLC (SIL-plate): ^{i}hexane/EtOAc = 16/1, R_f = 0.60 [very slight spot, stained with $KMnO_4$].

HPLC (IE-3R, $CH_3CN:H_2O$ = 50:50, 1.0 mL/min, 220 nm, 298 K): t_R(minor) = 24.698 min, t_R(major) = 23.376 min. e.r. = 89.5:10.5 (79% ee).

Methyl (*R*)-2-(6-((*tert*-butyldimethylsilyl)oxy)-2,3,4,5-tetrahydro-[1,1'-biphenyl]-2-yl) acetate (3v)

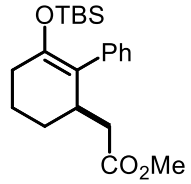

The reaction was conducted according to general procedure with enone **1v** (17.2 mg, 0.1 mmol, 1.0 equiv.), silyl ketene acetal **2a** (28.2 mg, 0.15 mmol, 1.5 equiv.) and **IDPi 4f** (1.6 mg, 1 mol%) in PhMe at –80 °C for 36 h. Product **3v** as a colorless oil was afforded after purification via silica gel flash chromatography using ^{i}hexane/Et_2O (from 30:1 to 12:1 *v*/*v*) as eluent (29.6 mg, 82% yield).

^{1}H NMR (501 MHz, CD_2Cl_2): δ 7.33–7.28 (m, 2H), 7.26–7.23 (m, 2H), 7.22–7.17 (m, 1H), 3.55 (s, 3H), 3.11–3.06 (m, 1H), 2.26–2.15 (m, 4H), 1.83–1.73 (m, 3H), 1.68–1.63 (m, 1H), 0.72 (s, 9H), 0.03 (s, 3H), –0.30 (s, 3H).

^{13}C NMR (126 MHz, CD_2Cl_2): δ 173.2, 146.8, 139.8, 129.8, 127.8, 125.9, 119.8, 51.1, 38.2, 35.5, 31.0, 27.5, 25.3, 19.2, 17.8, –4.5, –4.9.

HRMS (ESI, positive ions): calculated for $C_{21}H_{32}NaO_3Si$ ($[M+Na]^+$): 383.2013, found: 383.2011.

$[\alpha]_D^{25}$ = +65.56 (*c* 0.60, $CHCl_3$)

TLC (SIL-plate): ^{i}hexane/EtOAc = 8/1, R_f = 0.58.

HPLC (OJ-3R, $CH_3CN:H_2O$ = 60:40, 1.0 mL/min, 220 nm, 298 K): t_R(minor) = 11.246 min, t_R(major) = 10.241 min. e.r. = 96:4 (92% ee).

Methyl (*R*)-2-(3-((*tert*-butyldimethylsilyl)oxy)cyclohept-2-en-1-yl)acetate (3w)

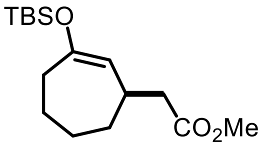

The reaction was conducted according to general procedure with enone **1w** (11.0 mg, 0.1 mmol, 1.0 equiv.), silyl ketene acetal **2a** (28.2 mg, 0.15 mmol, 1.5 equiv.) and **IDPi 4f** (1.6 mg, 1 mol%) in PhMe at –80 °C for 18 h. Product **3w** as a colorless oil was afforded after purification via silica gel flash chromatography using ^{i}hexane/Et_2O (from 30:1 to 18:1 *v*/*v*) as eluent (26.6 mg, 89% yield).

^{1}H NMR (501 MHz, CD_2Cl_2): δ 4.77 (d, *J* = 4.5 Hz, 1H), 3.67 (s, 3H), 2.71–2.64 (m, 1H), 2.43–2.29 (m, 3H), 2.17–2.11 (m, 1H), 1.93–1.86 (m, 1H), 1.74–1.57 (m, 3H), 1.54–1.45 (m, 1H), 1.43–1.34 (m, 1H), 0.94 (s, 9H), 0.15 (d, *J* = 2.7 Hz, 6H).

^{13}C NMR (126 MHz, CD_2Cl_2): δ 173.0, 155.6, 112.1, 51.2, 41.7, 35.2, 33.9, 33.4, 29.5, 25.4, 25.0, 17.8, –4.8.

HRMS (GC-EI): calculated for $C_{16}H_{30}O_3Si$ ($[M]^+$): 298.1959, found: 298.1954.

$[\alpha]_D^{25}$ = –1.39 (*c* 0.14, $CHCl_3$)

TLC (SIL-plate): ^{i}hexane/EtOAc = 16/1, R_f = 0.55 [very slight spot, stained with $KMnO_4$].

HPLC (OD-3R, $CH_3CN:H_2O$ = 50:50, 1.0 mL/min, 220 nm, 298 K): t_R(minor) = 29.762 min, t_R(major) = 32.178 min. e.r. = 67:33 (34% ee).

Benzyl (*R*)-4-((*tert*-butyldimethylsilyl)oxy)-6-(2-methoxy-2-oxoethyl)-3,6-dihydropyridine-1(2H)-carboxylate (3x)

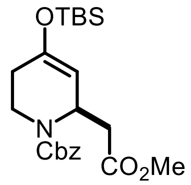

The reaction was conducted according to general procedure with enone **1x** (23.0 mg, 0.1 mmol, 1.0 equiv.), silyl ketene acetal **2a** (28.2 mg, 0.15 mmol, 1.5 equiv.) and **IDPi 4a** (1.5 mg, 1 mol%) in Et_2O at –80 °C for 18 h. Product **3x** as a colorless oil was afforded after purification via silica gel flash chromatography using ^{i}hexane/Et_2O (from 20:1 to 5:1 *v*/*v*) as eluent (40.3 mg, 96% yield).

^{1}H NMR (600 MHz, tol-d_8): δ 7.23–7.18 (m, 2H), 7.11–7.07 (m, 2H), 7.05–7.01 (m, 1H), 5.08–5.03 (m, 3H), 4.91 (dd, *J* = 4.3, 2.1 Hz, 1H), 4.12 (s, 1H), 3.34 (s, 3H), 2.81 (ddd, *J* = 13.4, 11.7, 4.2 Hz, 1H), 2.48 (dd, *J* = 14.2, 6.1 Hz, 1H), 2.32 (dd, *J* = 14.2, 7.7 Hz, 1H), 2.14 (dddt, *J* = 16.5, 11.8, 6.4, 2.3 Hz, 1H), 1.64–1.60 (m, 1H), 0.89 (s, 9H), 0.05 (d, *J* = 9.0 Hz, 6H).

^{13}C NMR (151 MHz, tol-d_8): δ 170.8, 155.0, 150.9, 137.9, 137.7, 128.7, 128.4, 104.8, 67.5, 51.0, 49.4, 39.9, 38.0, 30.3, 26.0, 18.4, –4.2, –4.3.

HRMS (ESI, positive ions): calculated for $C_{22}H_{33}NNaO_5Si$ ($[M+Na]^+$): 442.2020, found: 442.2025.

$[\alpha]_D^{25}$ = –202.21 (*c* 0.81, $CHCl_3$)

TLC (SIL-plate): ^{i}hexane/EtOAc = 4/1, R_f = 0.53.

HPLC (IC-3, ^{n}heptane:^{i}PrOH = 95:5, 1.0 mL/min, 220 nm, 298 K): t_R(minor) = 10.035 min, t_R(major) = 11.687 min. e.r. = 98:2 (96% ee).

Methyl (*R*)-2-(4-((*tert*-butyldimethylsilyl)oxy)-2H-chromen-2-yl)acetate (3y)

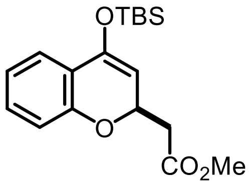

The reaction was conducted according to general procedure with enone **1y** (14.6 mg, 0.1 mmol, 1.0 equiv.), silyl ketene acetal **2a** (28.2 mg, 0.15 mmol, 1.5 equiv.) and **IDPi 4e** (1.4 mg, 1 mol%) in Et_2O at –80 °C for 18 h. Product **3y** as a colorless oil was afforded after purification via silica gel flash chromatography using ^{i}hexane/Et_2O (from 20:1 to 8:1 *v*/*v*) as eluent (32.1 mg, 93% yield).

^{1}H NMR (501 MHz, $CDCl_3$): δ 7.38 (dd, *J* = 7.6, 1.7 Hz, 1H), 7.17 (td, *J* = 7.7, 1.7 Hz, 1H), 6.93 (td, *J* = 7.5, 1.1 Hz, 1H), 6.81 (dd, *J* = 8.1, 1.1 Hz, 1H), 5.39 (ddd, *J* = 7.7, 5.8, 3.9 Hz, 1H), 4.90 (d, *J* = 4.0 Hz, 1H), 3.74 (s, 3H), 2.88 (dd, *J* = 15.2, 7.7 Hz, 1H), 2.65 (dd, *J* = 15.2, 5.8 Hz, 1H), 1.03 (s, 9H), 0.24 (d, *J* = 3.5 Hz, 6H).

^{13}C NMR (126 MHz, $CDCl_3$): δ 170.8, 153.9, 146.2, 129.8, 122.5, 121.2, 121.0, 116.1, 100.7, 72.4, 51.8, 41.0, 25.8, 18.3, –4.5, –4.6.

HRMS (GC-EI): calculated for $C_{18}H_{26}O_4Si$ ($[M]^+$): 334.1595, found: 334.1593.

$[\alpha]_D^{25}$ = +122.32 (*c* 0.88, $CHCl_3$)

TLC (SIL-plate): ^{i}hexane/EtOAc = 8/1, R_f = 0.55.

HPLC (AD-3R, CH_3CN:H_2O = 60:40, 1.0 mL/min, 220 nm, 298 K): t_R(minor) = 7.822 min, t_R(major) = 6.705 min. e.r. = 95:5 (90% ee).

Methyl (*R*)-2-(6-bromo-4-((*tert*-butyldimethylsilyl)oxy)-2H-thiochromen-2-yl)acetate (3z)

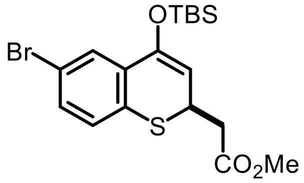

The reaction was conducted according to general procedure with enone **1z** (24.1 mg, 0.1 mmol, 1.0 equiv.), silyl ketene acetal **2a** (28.2 mg, 0.15 mmol, 1.5 equiv.) and **IDPi 4a** (1.7 mg, 1 mol%) in PhMe at –80 °C for 18 h. Product **3z** as a colorless oil was afforded after purification via silica gel flash chromatography using ^{i}hexane/Et_2O

(from 20:1 to 8:1 *v*/*v*) as eluent (39.1 mg, 91% yield).
^{1}H NMR (501 MHz, CD_2Cl_2): δ 7.74 (d, *J* = 2.2 Hz, 1H), 7.32 (dd, *J* = 8.3, 2.3 Hz, 1H), 7.16 (d, *J* = 8.3 Hz, 1H), 5.41 (d, *J* = 7.2 Hz, 1H), 3.96 (q, *J* = 7.4 Hz, 1H), 3.69 (s, 3H), 2.72–2.62 (m, 2H), 1.04 (s, 9H), 0.24 (d, *J* = 17.0 Hz, 6H).
^{13}C NMR (126 MHz, CD_2Cl_2): δ 170.7, 148.3, 132.8, 131.2, 130.8, 128.8, 127.2, 118.6, 105.2, 51.6, 41.2, 34.3, 25.5, 18.2, –4.7, –5.0.
HRMS (ESI, positive ions): calculated for $C_{18}H_{25}BrNaO_3SSi$ ($[M+Na]^+$): 451.0369, found: 451.0371.
$[\alpha]_D^{25}$ = +1.04 (*c* 0.58, $CHCl_3$)
TLC (SIL-plate): ^{i}hexane/EtOAc = 5/1, R_f = 0.68.
HPLC (IE-3R, CH_3CN:H_2O = 60:40, 1.0 mL/min, 220 nm, 298 K): t_R(minor) = 23.312 min, t_R(major) = 22.303 min. e.r. = 98:2 (96% ee).

Methyl (*S*)-2-(3-((*tert*-butyldimethylsilyl)oxy)-2-methylcyclopent-2-en-1-yl)-2-methylpropanoate (3aa)

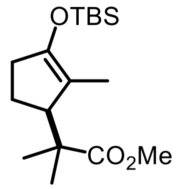

The reaction was conducted according to general procedure with enone **1b** (9.6 mg, 0.1 mmol, 1.0 equiv.), silyl ketene acetal **2b** (32.4 mg, 0.15 mmol, 1.5 equiv.) and **IDPi 4a** (1.7 mg, 1 mol%) in PhMe at –80 °C for 18 h. Product **3aa** as a colorless oil was afforded after purification via silica gel flash chromatography using ^{i}hexane/Et_2O (from 30:1 to 18:1 *v*/*v*) as eluent (26.3 mg, 84% yield).
^{1}H NMR (501 MHz, CD_2Cl_2): δ 3.67 (s, 3H), 3.05–2.98 (m, 1H), 2.31–2.15 (m, 2H), 1.97–1.89 (m, 1H), 1.59–1.52 (m, 1H), 1.46 (q, *J* = 1.1 Hz, 3H), 1.14 (s, 3H), 1.09 (s, 3H), 0.97 (s, 9H), 0.15 (d, *J* = 2.0 Hz, 6H).
^{13}C NMR (126 MHz, CD_2Cl_2): δ 178.7, 149.4, 113.5, 52.0, 51.3, 45.4, 32.4, 25.4, 23.3, 22.5, 19.9, 17.9, 11.3, –4.3, –4.4.
HRMS (ESI, positive ions): calculated for $C_{17}H_{33}O_3Si$ ($[M+H]^+$): 313.2193, found: 313.2192.
$[\alpha]_D^{25}$ = +68.03 (*c* 0.15, $CHCl_3$)
TLC (SIL-plate): ^{i}hexane/EtOAc = 16/1, R_f = 0.56 [very slight spot, stained with $KMnO_4$].
HPLC (OJ-3R, CH_3OH:H_2O = 80:20, 1.0 mL/min, 220 nm, 298 K): t_R(minor) = 8.223 min, t_R(major) = 8.982 min. e.r. = 93:7 (86% ee).

Benzyl (*R*)-2-(3-((*tert*-butyldimethylsilyl)oxy)-2-methylcyclopent-2-en-1-yl)acetate (3ab)

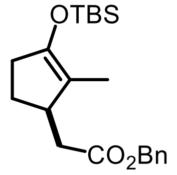

The reaction was conducted according to general procedure with enone **1b** (9.6 mg, 0.1 mmol, 1.0 equiv.), silyl ketene acetal **2c** (39.4 mg, 0.15 mmol, 1.5 equiv.) and **IDPi 4f** (1.6 mg, 1 mol%) in PhMe at –80 °C for 18 h. Product **3ab** as a colorless oil was afforded after purification via silica gel flash chromatography using ^{i}hexane/Et_2O (from 30:1 to 16:1 *v*/*v*) as eluent (31.4 mg, 87% yield).
^{1}H NMR (501 MHz, CD_2Cl_2): δ 7.45–7.32 (m, 2H), 7.39 (s, 3H), 5.13 (d, *J* = 1.8 Hz, 2H), 2.93–2.87 (m, 1H), 2.61 (dd, *J* = 14.9, 4.5 Hz, 1H), 2.36–2.23 (m, 2H), 2.19 (dd, *J* = 14.9, 9.7 Hz, 1H), 2.09 (dddd, *J* = 13.5, 9.2, 8.3, 5.4 Hz, 1H), 1.56–1.50 (m, 4H), 0.97 (s, 9H), 0.15 (s, 6H).
^{13}C NMR (126 MHz, CD_2Cl_2): δ 172.8, 147.8, 136.4, 128.4, 128.1, 128.0, 114.3, 65.9, 41.8, 39.4, 32.2, 26.7, 25.4, 18.0, 9.8, –4.4.
HRMS (GC-EI): calculated for $C_{21}H_{32}O_3Si$ ($[M]^+$): 360.2115, found: 360.2111.

$[\alpha]_D^{25}$ = +30.77 (*c* 0.16, $CHCl_3$)
TLC (SIL-plate): ^{i}hexane/EtOAc = 10/1, R_f = 0.55.
HPLC (OJ-3R, CH_3OH:H_2O = 90:10, 1.0 mL/min, 220 nm, 298 K): t_R(minor) = 8.657 min, t_R(major) = 9.440 min. e.r. = 93:7 (86% ee).

(*R*)-2-(3-((*tert*-butyldimethylsilyl)oxy)-2-pentylcyclopent-2-en-1-yl)-1-phenylethan-1-one (3ac)

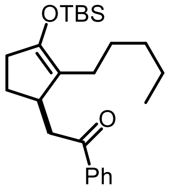

The reaction was conducted according to general procedure with enone **1c** (15.2 mg, 0.1 mmol, 1.0 equiv.), enol silane **2d** (35.2 mg, 0.15 mmol, 1.5 equiv.) and **IDPi 4e** (1.4 mg, 1 mol%) in PhMe at –80 °C for 18 h. Product **3ac** as a colorless oil was afforded after purification via silica gel flash chromatography using ^{i}hexane/Et_2O (from 20:1 to 12:1 *v*/*v*) as eluent (33.3 mg, 86% yield).
^{1}H NMR (501 MHz, CD_2Cl_2): δ 8.00–7.96 (m, 2H), 7.63–7.58 (m, 1H), 7.53–7.49 (m, 2H), 3.23 (dd, *J* = 16.1, 3.6 Hz, 1H), 3.19–3.14 (m, 1H), 2.77 (dd, *J* = 16.1, 10.1 Hz, 1H), 2.39–2.24 (m, 3H), 2.16–2.08 (m, 1H), 1.83 (d, *J* = 7.0 Hz, 1H), 1.50–1.44 (m, 2H), 1.38–1.27 (m, 5H), 0.99 (s, 9H), 0.92 (t, *J* = 7.0 Hz, 3H), 0.17 (d, *J* = 3.0 Hz, 6H).
^{13}C NMR (126 MHz, CD_2Cl_2): δ 200.0, 147.5, 137.5, 132.7, 128.5, 127.9, 119.1, 43.5, 38.8, 32.3, 32.0, 27.4, 27.2, 25.4, 24.3, 22.6, 18.0, 13.9, –4.3, –4.4.
HRMS (GC-EI): calculated for $C_{24}H_{38}O_2Si$ ($[M]^+$): 386.2636, found: 386.2632.
$[\alpha]_D^{25}$ = +53.17 (*c* 0.71, $CHCl_3$)
TLC (SIL-plate): ^{i}hexane/EtOAc = 10/1, R_f = 0.60.
HPLC (OJ-3R, CH_3OH:H_2O = 85:15, 1.0 mL/min, 220 nm, 298 K): t_R(minor) = 13.33 min, t_R(major) = 17.36 min. e.r. = 96:4 (92% ee).

(*R*)-1-(3-((*tert*-butyldimethylsilyl)oxy)-2-pentylcyclopent-2-en-1-yl)propan-2-one (3ad)

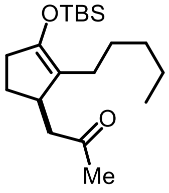

The reaction was conducted according to general procedure with enone **1c** (15.2 mg, 0.1 mmol, 1.0 equiv.), enol silane **2e** (51.7 mg, 0.3 mmol, 3.0 equiv.) and **IDPi 4f** (1.6 mg, 1 mol%) in Et_2O at –80 °C for 36 h. Product **3ad** as a colorless oil was afforded after purification via silica gel flash chromatography using ^{i}hexane/Et_2O (from 20:1 to 8:1 *v*/*v*) as eluent (22.1 mg, 68% yield).
^{1}H NMR (501 MHz, CD_2Cl_2): δ 3.01–2.95 (m, 1H), 2.67 (dd, *J* = 16.1, 3.6 Hz, 1H), 2.35–2.19 (m, 4H), 2.14 (s, 3H), 2.11–2.04 (m, 1H), 1.77–1.71 (m, 1H), 1.46–1.24 (m, 7H), 0.97 (s, 9H), 0.92 (t, *J* = 7.1 Hz, 3H), 0.15 (d, *J* = 1.5 Hz, 6H).
^{13}C NMR (126 MHz, CD_2Cl_2): δ 208.5, 147.3, 118.8, 48.6, 38.3, 32.3, 32.0, 30.1, 27.3, 27.1, 25.4, 24.2, 22.5, 17.9, 13.9, –4.3, –4.4.
HRMS (GC-EI): calculated for $C_{19}H_{36}O_2Si$ ($[M]^+$): 324.2479, found: 324.2474.
$[\alpha]_D^{25}$ = +42.11 (*c* 0.91, $CHCl_3$)
TLC (SIL-plate): ^{i}hexane/EtOAc = 16/1, R_f = 0.60 [very slight spot, stained with $KMnO_4$]
HPLC (OJ-3R, CH_3CN:H_2O = 50:50, 1.0 mL/min, 220 nm, 298 K): t_R(minor) = 24.987 min, t_R(major) = 26.829 min. e.r. = 96:4 (92% ee).

(*R*)-3-((*R*)-3-((*tert*-butyldimethylsilyl)oxy)-2-methylcyclopent-2-en-1-yl)-4-methylpentan-2-one (3ae)

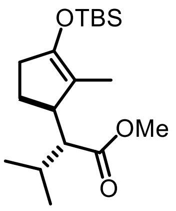

In accordance with the general procedure, the reaction was performed using enone **1b** (9.6 mg, 0.1 mmol, 1.0 equiv.), silyl ketene acetal **2f** (34.6 mg, 1.5 mmol, 1.5 equiv., 96:4 *E*/*Z*) and **IDPi 4f** (1.6 mg, 1 mol%) in PhMe at –80 °C for 18 h. Product **3ae** as a colorless oil was afforded after purification via silica gel flash chromatography using ^{i}hexane/Et_2O (from 30:1 to 16:1 *v*/*v*) as eluent (19.6 mg, 63% yield). NMR analysis revealed a diastereomeric ratio of 4:1.

^{1}H NMR (600 MHz, CD_2Cl_2, for major isomer): δ 3.61 (s, 3H), 2.91 – 2.83 (m, 1H), 2.22 – 2.17 (m, 2H), 2.15 (dd, *J* = 8.2, 6.2 Hz, 1H), 2.07 – 2.01 (m, 1H), 1.99 – 1.93 (m, 1H), 1.57 (dddd, *J* = 12.7, 8.8, 7.3, 6.3 Hz, 1H), 1.44 (td, *J* = 2.1, 1.1 Hz, 3H), 0.93 (s, 9H), 0.91 (dd, *J* = 20.3, 6.7 Hz, 6H), 0.11 (d, *J* = 3.2 Hz, 6H).

^{1}H NMR (600 MHz, CD_2Cl_2, for minor isomer): 3.55 (s, 0.74H), 2.84 – 2.79 (m, 0.26H), 2.29 (dd, *J* = 10.4, 4.5 Hz, 0.27H), 2.25 – 2.22 (m, 0.27H), 2.14 – 2.12 (m, 0.12H), 2.12 – 2.07 (m, 0.27H), 2.00 – 1.97 (m, 0.66H), 1.82 (dddd, *J* = 13.1, 10.0, 9.3, 5.5 Hz, 0.29H), 1.52 (td, *J* = 2.1, 1.1 Hz, 0.77H), 0.92 (s, 2.26H), 0.88 (dd, *J* = 12.0, 6.6 Hz, 1.5H), 0.09 (d, *J* = 2.7 Hz, 1.5H).

^{13}C NMR (151 MHz, CD_2Cl_2, for major isomer): δ 175.4, 148.6, 115.3, 56.3, 51.1, 45.1, 32.5, 28.1, 25.8, 25.5, 21.5, 18.7, 18.3, 10.9, –3.97, –3.98

^{13}C NMR (151 MHz, CD_2Cl_2, for minor isomer): δ 175.7, 148.3, 113.9, 55.2, 51.1, 45.6, 32.9, 28.5, 25.8, 22.0, 20.6, 20.3, 18.3, 10.5, –3.96, –4.07.

HRMS (ESI, positive ion): calculated for $C_{18}H_{34}NaO_3Si$ $(M+Na)^+$ = 349.2169, found: 349.2168.

[α]25 *D* = + 37.04 (*c* 0.42, $CHCl_3$)

TLC (SIL-plate): ^{i}hexane/EtOAc = 16/1, R_f = 0.65 [very slight spot, stained with $KMnO_4$]

The enantiomeric ratio was measured by **2D-LC** analysis: 1-dimension using 100 mm RX-SIL, 4.6 mm i.D, ^{n}heptane/^{i}PrOH = 99.9:0.1, 1.0 mL/min, λ = 220 nm, 308 K, t_R = 3.72 min and then 2-dimension using 150 mm Chiralcel OZ-3, 4.6 mm i.D, ^{n}heptane/^{i}PrOH = 99.99:0.01, 1.0 mL/min, λ = 220 nm, 298 K, t_R(minor) = 7.446 min, t_R(minor) = 8.152 min. e.r. = 99:1 (98% ee).

Methyl 2-((1*R*,5*S*)-3-((*tert*-butyldimethylsilyl)oxy)-2-methyl-5-(prop-1-en-2-yl)cyclohex-2-en-1-yl)acetate (3af)

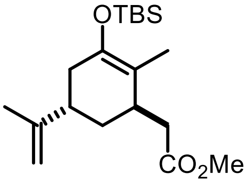

The reaction was conducted according to general procedure with enone **1ae** (15.0 mg, 0.1 mmol, 2.0 equiv.), silyl ketene acetal **2a** (10.3 mg, 0.055 mmol, 1.1 equiv.) and **IDPi 4f** (1.6 mg, 1 mol%) in PhMe at –80 °C for 18 h. Product **3af** as a colorless oil was afforded after purification via silica gel flash chromatography using pentane/Et_2O (from 30:1 to 18:1 *v*/*v*) as eluent (15.2 mg, 45% yield).

^{1}H NMR (600 MHz, CD_2Cl_2): δ 4.74 (~pd, *J* = 1.8, 0.5 Hz, 1H), 4.72 (tt, *J* = 2.0, 0.8 Hz, 1H), 3.64 (s, 3H), 2.56 (~dt(m), *J* = 10.3, 5.3, 4.3 Hz, 1H), 2.53 (ddd, *J* = 14.9, 3.7, 1.2 Hz, 1H), 2.34 (tt, *J* = 11.4, 5.5 Hz, 1H), 2.24 (dd, *J* = 15.0, 10.4 Hz, 1H), 2.05 (ddq, *J* = 16.5, 5.6, 1.6, 0.8 Hz, 1H), 1.98 (dddt, *J* = 16.5, 11.0, 3.8, 1.8 Hz, 1H), 1.73 (dt, *J* = 1.4, 0.4 Hz, 3H), 1.61 (ddt, *J* = 13.2, 3.1, 1.6 Hz, 1H), 1.59 (ddd, *J* = 2.2, 1.6, 0.6 Hz, 3H), 1.53 (tdd, *J* = 13.2, 12.6, 5.4, 1.4 Hz, 1H), 0.94 (s, 9H), 0.12 (q, *J* = 0.5 Hz, 6H).

^{13}C NMR (151 MHz, CD_2Cl_2): δ 173.9, 149.4, 144.7, 113.1, 109.1, 54.2, 54.0, 53.8, 53.7, 53.5, 51.7, 38.2, 37.5, 36.8, 36.0, 32.4, 26.0, 20.9, 18.5, 14.9, –3.56, –3.58, –3.8.

HRMS (GC-EI): calculated for $C_{19}H_{34}O_3Si$ ($[M]^+$): 338.2272, found: 324.2268.

$[\alpha]_D^{25}$ = +23.01 (*c* 0.93, $CHCl_3$)

TLC (SIL-plate): pentane/EtOAc = 16/1, R_f = 0.62 [very slight spot, stained with $KMnO_4$].
HPLC (OJ-3R, CH_3CN:H_2O = 60:40, 1.0 mL/min, 220 nm, 298 K): t_R(minor) = 8.592 min, t_R(major) = 9.798 min. e.r. = 93:7 (86% ee).

General Procedure for Scale-up Synthesis of 3c

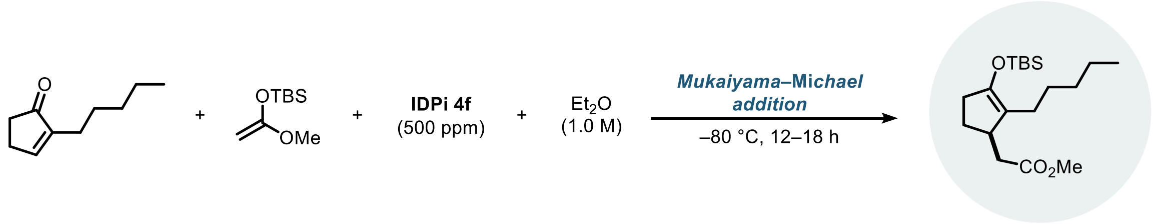

1st batch	1 mmol<br>152 mg	1.1 mmol<br>207 mg	0.05 μmol<br>0.832 mg	1 mL	full conv., 99.1:0.9 e.r.<br>326.9 mg (96%)
2nd batch	10 mmol<br>1.52 g	11 mmol<br>2.07 g	0.5 μmol<br>8.32 mg	10 mL	full conv., 99.3:0.7 e.r.<br>3.2 g (95%)
3rd batch	50 mmol<br>7.61 g	55 mmol<br>10.36 g	2.5 μmol<br>41.6 mg	50 mL	full conv., 99.3:0.7 e.r.<br>16.2 g (95%)
4th batch	100 mmol<br>15.22 g	110 mmol<br>20.72 g	0.005 mmol<br>83.2 mg	100 mL	full conv., 99.4:0.6 e.r.<br>33.6 g (98%)
5th batch	200 mmol<br>30.45 g	220 mmol<br>41.43 g	0.01 mmol<br>166.4 mg	200 mL	full conv., 99.4:0.6 e.r.<br>65.0 g (95.4%)*
6th batch	500 mmol<br>76.12 g	550 mmol<br>103.59 g	0.25 mmol<br>416.16 mg	500 mL	full conv., 99.5:0.5 e.r.<br>167.0 g (98.1%)*

*Isolated yield after distillation

For a 500 mmol scale synthesis, a 2.0 L of flame-dried Schlenk flask was refilled with argon 3 times, and charged with **IDPi 4f** (416.16 mg, 500 ppm). Dry ether (500 mL, 1.0 M) and freshly distilled silyl ketene acetal **2a** (103.59 g, 1.1 equiv., 550 mmol) were added sequentially at room temperature. The mixture was stirred for 20 min before completely cooled down to –80 °C while stirring (takes 35 min to reach –80 °C). The freshly distilled **2-pentyl cyclopentenone 1c** (76.12 g, 500 mmol) was then added dropwise using an automatic syringe pump (divided into 4 portions, each portion: 0.350 mL/min, over 60 min). After 18 h, the reaction was quenched with 1 mL of saturated $NaHCO_3$ at –80 °C, stirred for additional 15 min, and then slowly warmed to room temperature.
Work-up procedure: The reaction mixture was transferred to a 2.0 L round bottom flask, washed 3 times with dry ether, and extracted 3 times with dry pentane, followed by washes with distilled water and brine. The combined organic phase was dried over Na_2SO_4 and concentrated in vacuo. The crude product was purified by careful distillation (0.032 mbar, oil bath temp.: 162 °C, internal temp.: 106 °C) to afford **3c** as a colorless oil (167.0 g, 98.1% distilled yield), which was stored under an Ar atmosphere and avoid the light.
Additionally, the recovered catalyst **4f** was isolated in 96% yield (399.5 mg) following purification by column chromatography and acidification.

General procedure for the one-pot trans-selective protodesilylation:

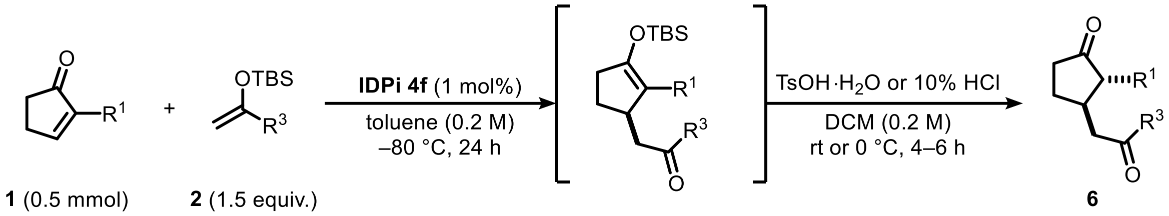

1 (0.5 mmol) **2** (1.5 equiv.) **6**

An oven-dried screw-cap vial equipped with a magnetic stirring bar was charged with **IDPi 4f** (1 mol%) and enone **1** (0.5 mmol, 1.0 equiv.) under Ar atmosphere, followed by the addition of freshly distilled PhMe (0.2 M). Once completely dissolved, the mixture was transferred to a cryostat set at −80 °C. Enol silane **2** (0.75 mmol, 1.5 equiv.) was then added dropwise, and the reaction was stirred for 24 h. Upon completion, the reaction was quenched with 10 µL of saturated $NaHCO_3$ and stirred for additional 15 min, and then slowly warmed to room temperature. The reaction mixture was subsequently filtrated through a pad of celite on neutral Al_2O_3 and eluted with ^{i}hexane/EtOAc (10:1 *v*/*v*) 2−3 times. The combined organic layers were concentrated under the reduced pressure.

For 2-alkyl substituted starting material, to the crude product in the screw-cap vial was added DCM (0.2 M) at 0 °C, followed by 10% aqueous HCl (*v*/*v* = 5%). The reaction was stirred for 4 h. After completion, the resulting reaction mixture was extracted with Et_2O (3 x 20 mL), and the combined organic phase was dried over anhydrous Na_2SO_4, filtered, and the solvents were removed under the reduced pressure. The target product was afforded by flash column chromatography purification on silica gel using ^{i}hexane/EtOAc as eluent.

For 2-aryl substituted starting material, to the crude product in the screw-cap vial was added DCM (0.2 M) at 0 °C, followed by TsOH·H_2O (1.5 equiv.). The reaction was stirred for 4-6 h. After completion, the resulting reaction mixture was extracted with Et_2O (3 x 20 mL), and the combined organic phase was dried over anhydrous Na_2SO_4, filtered, and the solvents were removed under the reduced pressure. The target product **6** was afforded by flash column chromatography purification on silica gel using ^{i}hexane/EtOAc as eluent.

Methyl 2-((1*R*,2*R*)-3-oxo-2-pentylcyclopentyl)acetate (6a)

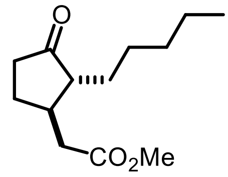

In accordance with the general procedure, the reaction was performed using enone **1c** (76.1 mg, 0.5 mmol, 1.0 equiv.), silyl ketene acetal **2a** (141.3 mg, 0.75 mmol, 1.5 equiv.) and **IDPi 4f** (8.3 mg, 1 mol%) in PhMe at –80 °C for 24 h. The resulting silylated intermediate was treated with 10% aqueous HCl. After purification by silica gel flash chromatography using ^{i}hexane/EtOAc (from 20:1 to 8:1 *v*/*v*) as eluent, the desired product **6a** was obtained as a colorless oil (104.1 mg, 92% yield over 2 steps). GC analysis revealed a diastereomeric ratio of 12:1.

^{1}H NMR (501 MHz, CD_2Cl_2) δ 3.70 (s, 3H), 2.68–2.63 (m, 1H), 2.39–2.29 (m, 3H), 2.27–2.21 (m, 1H), 2.13 (ddd, *J* = 18.6, 10.9, 8.8 Hz, 1H), 1.83–1.78 (m, 1H), 1.58–1.41 (m, 4H), 1.36–1.24 (m, 5H), 0.91 (t, *J* = 7.1 Hz, 4H).

^{13}C NMR (126 MHz, CD_2Cl_2) δ 219.2, 172.5, 54.1, 51.4, 38.8, 38.2, 37.6, 32.1, 27.8, 27.1, 26.3, 22.5, 13.8.

HRMS (GC-EI): calculated for $C_{13}H_{22}O_3$ ($[M]^+$): 226.1564, found: 226.1563.

$[\alpha]_D^{25}$ = –39.92 (*c* 0.95, $CHCl_3$)

TLC (SIL-plate): ^{i}hexane/EtOAc = 4/1, R_f = 0.5 [stained with CAM].

Chiral-GC (Column: 30.0 m, BGB-176/BGB-15 0.25/0.25df G/618; Temp.: 220/120, iso. /350; Gas: 0.60 bar H_2):

For major product: t_R(minor) = 102.60 min, t_R(major) = 95.55 min. e.r. = 99:1 (98% ee), for minor product: t_R(minor) = 117.84 min, t_R(major) = 113.54 min. e.r. = >99:1 (99% ee); d.r. = 12:1.

Preparation of *N*-(6-bromonaphthalen-2-yl)-2-((1*R*,2*R*)-3-oxo-2-pentylcyclopentyl) acetamide (7)

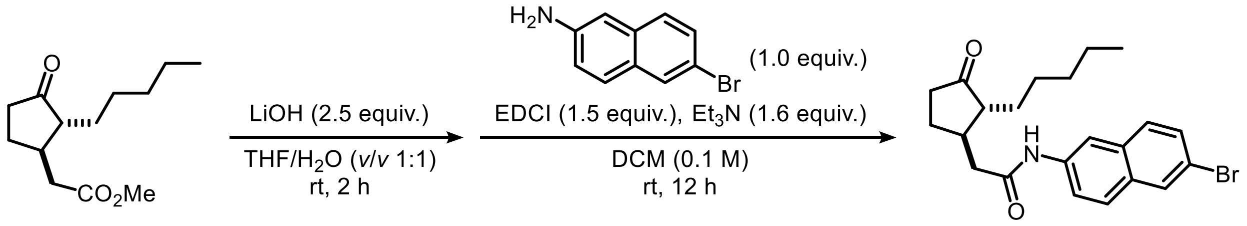

6a, 99% ee, 12:1 dr

7

In a round-bottom flask equipped with methyl dihydrojasmonate **6a** (106.1 mg, 0.5 mmol) and a magnetic stir bar, was dissolved in a mixture of THF (3 mL) and water (3 mL). LiOH (30.0 mg, 1.25 mmol) was added in portions, and the reaction mixture was stirred at room temperature for 2 h. After full consumption of the starting material, as monitored by TLC analysis, the reaction was neutralized to pH = 7.0 by adding 10% HCl aqueous. The mixture was extracted with ether (3 x 20 mL). The combined organic layers were subsequently washed with water, followed by brine, dried over anhydrous Na_2SO_4 and filtered before concentrated in vacuo. The resulting crude hydrolysis product was used directly in the next step without further purification.

For the amidation, a solution of EDCI (144 mg, 0.75 mmol, 1.5 equiv.) in dry DCM (5 mL, 0.1 M) was prepared, to which trimethylamine (112 μL, 0.8 mmol, 1.6 equiv.) was added. The mixture was stirred for 10 min, and then the crude carboxylic acid along with 2-amino-6-bromonaphthalene (111.0 mg, 0.5 mmol, 1.0 equiv.) were introduced. The reaction

was stirred for 12 h at room temperature. Upon completion, the reaction was diluted with DCM (30 mL) and water (30 mL), and the organic phase was extracted with DCM (3 x 20 mL) twice. The combined layers were washed with water and brine, then separated, dried over anhydrous Na_2SO_4, filtered. The solvent was removed under the reduced pressure, and the residue was purified via flash column chromatography using ^{i}hexane/EtOAc (from 10:1 to 4:1 *v*/*v*) as eluent to provide the amide **7** as a white solid (135.3 mg, 65% yield over 2 steps).

^{1}H NMR (501 MHz, CD_2Cl_2): δ 8.27 (d, *J* = 2.2 Hz, 1H), 8.00 (d, *J* = 2.0 Hz, 1H), 7.77 (d, *J* = 8.9 Hz, 1H), 7.73 (d, *J* = 8.8 Hz, 1H), 7.58 (dd, *J* = 8.8, 2.0 Hz, 1H), 7.53 (dd, *J* = 8.8, 2.2 Hz, 1H), 7.48 (s, 1H), 2.76 (dd, *J* = 14.4, 4.8 Hz, 1H), 2.49 (ddt, *J* = 15.0, 10.1, 5.0 Hz, 1H), 2.44–2.31 (m, 3H), 2.22–2.13 (m, 1H), 1.89 (dt, *J* = 10.7, 5.5 Hz, 1H), 1.64–1.57 (m, 3H), 1.36–1.26 (m, 6H), 0.90 (t, *J* = 7.0 Hz, 3H).

^{13}C NMR (126 MHz, CD_2Cl_2): δ 219.2, 169.8, 135.9, 132.3, 131.6, 129.8, 129.6, 129.3, 127.8, 120.8, 118.6, 116.2, 54.2, 42.5, 38.5, 37.7, 32.1, 28.0, 27.3, 26.5, 22.5, 13.8.

HRMS (ESI, positive ions): calculated for $C_{22}H_{26}NO_2Br$ ($[M+Na]^+$): 438.1039, found: 438.1043.

$[\alpha]_D^{25}$ = –16.58 (*c* 0.78, $CHCl_3$)

TLC (SIL-plate): ^{i}hexane/EtOAc = 2/1, R_f = 0.6.

HPLC (OJ-3R, CH_3CN:H_2O = 50:50, 1.0 mL/min, 220 nm, 298 K):

For major product: t_R(minor) = 24.899 min, t_R(major) = 26.294 min. e.r. = 99:1 (98% ee); d.r. >20:1.

(2*R*,3*R*)-3-(2-oxopropyl)-2-pentylcyclopentan-1-one (6b)

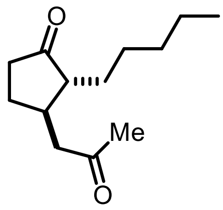

In accordance with the general procedure, the reaction was performed using enone **1c** (76.1 mg, 0.5 mmol, 1.0 equiv.), enol silane **2e** (282.6 mg, 1.5 mmol, 3.0 equiv.) and **IDPi 4f** (8.3 mg, 1 mol%) in Et_2O at –80 °C for 36 h. The resulting silylated intermediate was subsequently treated with 10% aqueous HCl. After purification by silica gel flash chromatography using ^{i}hexane/EtOAc (from 20:1 to 5:1 *v*/*v*) as eluent, the desired product **6b** was obtained as a colorless oil (59.9 mg, 57% yield over 2 steps). HPLC analysis revealed a diastereomeric ratio of 5:1.

^{1}H NMR (600 MHz, CD_2Cl_2): δ 2.74 (dd, *J* = 16.9, 4.1 Hz, 1H), 2.44 (dd, *J* = 16.9, 9.2 Hz, 1H), 2.35–2.17 (m, 3H), 2.14 (s, 3H), 1.74–1.66 (m, 1H), 1.53–1.45 (m, 2H), 1.44–1.33 (m, 2H), 1.33–1.17 (m, 6H), 0.88 (t, *J* = 7.2 Hz, 3H).

^{13}C NMR (151 MHz, CD_2Cl_2): δ 219.9, 207.7, 54.5, 48.7, 38.1, 37.5, 32.5, 30.6, 28.3, 27.7, 26.8, 22.9, 14.2.

HRMS (GC-EI): calculated for $C_{13}H_{22}O_2$ ($[M]^+$): 210.1614, found: 210.1613.

$[\alpha]_D^{25}$ = –26.38 (*c* 0.69, $CHCl_3$)

TLC (SIL-plate): ^{i}hexane/EtOAc = 3/1, R_f = 0.47 [stained with CAM].

Chiral-GC (Column: 30.0 m, BGB-176/BGB-15 0.25/0.25df G/618; Temp.: 220/130, 70 min. iso. 8/min. 240/350; Gas: 0.60 bar H_2):

For major product: t_R(minor) = 56.99 min, t_R(major) = 52.84 min. e.r. = 94:6 (86% ee), for minor product: t_R(minor) = 65.38 min, t_R(major) = 61.26 min. e.r. = 96:4 (92% ee); d.r. = 5:1.

Methyl 2-((1*R*,2*R*)-3-oxo-2-((*Z*)-pent-2-en-1-yl)cyclopentyl)acetate (6c)

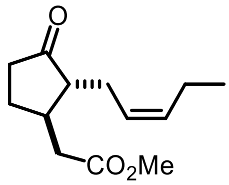

In accordance with the general procedure, the reaction was performed using enone **1e** (75.1 mg, 0.5 mmol, 1.0 equiv.), silyl ketene acetal **2a** (141.3 mg, 0.75 mmol, 1.5 equiv.) and **IDPi 4f** (8.3 mg, 1 mol%) in PhMe at –80 °C for 24 h. The resulting silylated intermediate was subsequently treated with 10% aqueous HCl. After purification by silica gel flash chromatography using ^{i}hexane/EtOAc (from 20:1 to 8:1 *v*/*v*) as eluent, the desired product **6c** was obtained as a colorless oil (108.8 mg, 97% yield over 2 steps). HPLC analysis revealed a diastereomeric ratio of >20:1.

^{1}H NMR (501 MHz, CD_2Cl_2): δ 5.52–5.44 (m, 1H), 5.34–5.26 (m, 1H), 3.70 (s, 3H), 2.75–2.68 (m, 1H), 2.39–2.29 (m, 5H), 2.28–2.22 (m, 1H), 2.15–2.06 (m, 3H), 1.94–1.88 (m, 1H), 1.51 (dddd, *J* = 12.3, 11.4, 9.9, 8.5 Hz, 1H), 0.99 (t, *J* = 7.5 Hz, 3H).

^{13}C NMR (151 MHz, CD_2Cl_2): δ 218.5, 172.4, 133.8, 125.2, 53.9, 51.4, 38.7, 38.0, 37.6, 27.1, 25.4, 20.5, 13.9.

HRMS (GC-EI): calculated for $C_{13}H_{20}O_3$ ($[M]^+$): 224.1407, found: 224.1409.

$[\alpha]_D^{22}$ = –93.42 (*c* 0.88, CH_3OH) [Literature report: $[\alpha]_D^{22}$ = –90.2 (*c* 1.03, CH_3OH)][6]

TLC (SIL-plate): ^{i}hexane/EtOAc = 4/1, R_f = 0.55 [very slight spot, stained with CAM].

HPLC (IG-3R, MeOH:H_2O = 70:30, 1.0 mL/min, 220 nm, 298 K):

For major product: t_R(minor) = 33.606 min, t_R(major) = 28.082 min. e.r. = 99.5:0.5 (99% ee), d.r. >20:1.

Preparation of 2-((1*R*,2*R*)-3-oxo-2-((*Z*)-pent-2-en-1-yl)cyclopentyl)acetic acid (8)

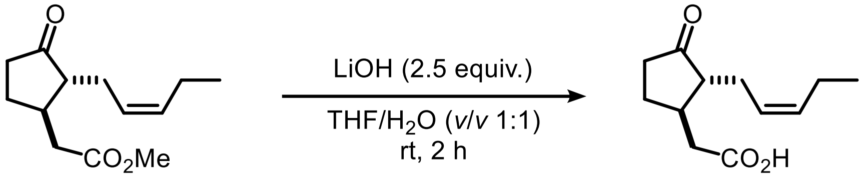

6c, 99% ee, >20/1 dr — **8**

In a round bottom flask equipped with methyl jasmonate **6c** (89.7 mg, 0.4 mmol) and stir bar, dissolved in 2 mL of THF and 2 mL of water. The LiOH (24.0 mg, 1 mmol) was added in portions. The reaction mixture was stirred at room temperature for 2 h. After full consumption, monitored by TLC, the reaction was neutralized to pH = 7.0 by introducing 10% HCl aqueous, then extracted with ether (3 x 20 mL) and water (30 mL). The combined organic layers were washed with water and brine, dried over anhydrous Na_2SO_4 and filtered before concentrated in vacuo. The crude product was purified through column chromatography using ^{i}hexane/EtOAc (from 10:1 to 3:1 *v*/*v*) as eluent to afford the desired carboxylic acid **8** as a light yellow oil (77.4 mg, 92% yield). NMR analysis revealed a diastereomeric ratio of 12:1.

^{1}H NMR (501 MHz, CD_2Cl_2): δ 5.53–5.46 (m, 1H), 5.34–5.27 (m, 1H), 2.83–2.78 (m, 1H), 2.42–2.28 (m, 6H), 2.18–2.06 (m, 3H), 1.97–1.91 (m, 1H), 1.60–1.51 (m, 1H), 0.99 (t, *J* = 7.5 Hz, 3H).

^{13}C NMR (126 MHz, CD_2Cl_2): δ 218.6, 178.0, 133.9, 125.1, 53.8, 38.6, 37.8, 37.6, 27.1, 25.4, 20.6, 13.9.

HRMS (GC-EI): calculated for $C_{12}H_{18}O_3$ ($[M]^+$): 210.1250, found: 210.1250.

$[\alpha]_D^{25}$ = –226.05 (*c* 0.62, $CHCl_3$)

TLC (SIL-plate): ^{i}hexane/EtOAc = 2/1, R_f = 0.43 [light spot, stained with CAM].
HPLC (OJ-3, ^{n}heptane:^{i}PrOH:TFA = 98:2:0.1, 1.0 mL/min, 210 nm, 298 K):
For major product: t_R(minor) = 20.48 min, t_R(major) = 15.02 min. e.r. = 99.5:0.5 (99% ee).

Methyl 2-((1*R*,2*S*)-3-oxo-2-(m-tolyl)cyclopentyl)acetate (6d)

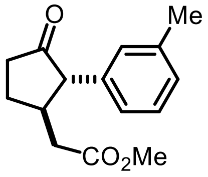

In accordance with the general procedure, the reaction was performed using enone **1aa** (86.1 mg, 0.5 mmol, 1.0 equiv.), silyl ketene acetal **2a** (141.3 mg, 0.75 mmol, 1.5 equiv.) and **IDPi 4f** (8.3 mg, 1 mol%) in PhMe at –80 °C for 24 h. The resulting silylated intermediate was subsequently treated with TsOH·H_2O. After purification by silica gel flash chromatography using ^{i}hexane/EtOAc (from 20:1 to 6:1 *v*/*v*) as eluent, the desired product **6d** was obtained as a colorless oil (88.7 mg, 72% yield over 2 steps). NMR analysis revealed a diastereomeric ratio of >20:1.
^{1}H NMR (501 MHz, CD_2Cl_2): δ 7.28–7.24 (m, 1H), 7.15–7.11 (m, 1H), 6.95–6.90 (m, 2H), 3.57 (s, 3H), 2.98 (d, *J* = 12.3 Hz, 1H), 2.74–2.65 (m, 1H), 2.58–2.51 (m, 2H), 2.45–2.35 (m, 7H), 1.71–1.64 (m, 1H).
^{13}C NMR (126 MHz, CD_2Cl_2): δ 216.3, 172.1, 138.3, 137.3, 129.6, 128.4, 127.9, 125.9, 61.8, 51.3, 41.7, 38.3, 38.1, 27.2, 21.1.
HRMS (GC-EI): calculated for $C_{15}H_{18}O_3$ ($[M]^+$): 246.1250, found: 246.1253.
$[\alpha]_D^{25}$ = +31.17 (*c* 0.15, $CHCl_3$)
TLC (SIL-plate): ^{i}hexane/EtOAc = 4/1, R_f = 0.50.
Chiral-GC (Column: 21.5 m, Ivadex-5/OV-1701 0.25/0.15df G/616; Temp.: 220/100, 860 min. iso. 8/min. 220, 3 min. iso. /350; Gas: 0.50 bar H_2): t_R(minor) = 816.67 min, t_R(major) = 769.91 min. e.r. = 99:1 (98% ee).

Methyl 2-((1*R*,2*S*)-2-(3,5-dimethylphenyl)-3-oxocyclopentyl)acetate (6e)

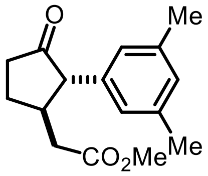

In accordance with the general procedure, the reaction was performed using enone **1ab** (93.1 mg, 0.5 mmol, 1.0 equiv.), silyl ketene acetal **2a** (141.3 mg, 0.75 mmol, 1.5 equiv.) and **IDPi 4f** (8.3 mg, 1 mol%) in PhMe at –80 °C for 24 h. The resulting silylated intermediate was subsequently treated with TsOH·H_2O. After purification by silica gel flash chromatography using ^{i}hexane/EtOAc (from 20:1 to 6:1 *v*/*v*) as eluent, the desired product **6e** was obtained as a colorless oil (83.3 mg, 64% yield over 2 steps). NMR analysis revealed a diastereomeric ratio of >20:1.
^{1}H NMR (501 MHz, CD_2Cl_2): δ 6.95 (s, 1H), 6.72 (s, 2H), 3.58 (s, 3H), 2.92 (dd, *J* = 12.3, 1.3 Hz, 1H), 2.73–2.64 (m, 1H), 2.58–2.50 (m, 2H), 2.43–2.35 (m, 3H), 2.33 (s, 6H), 1.69–1.62 (m, 1H).
^{13}C NMR (126 MHz, CD_2Cl_2): δ 216.5, 172.2, 138.1, 137.2, 128.8, 126.7, 61.7, 51.3, 41.7, 38.4, 38.1, 27.2, 20.9.
HRMS (GC-EI): calculated for $C_{16}H_{20}O_3$ ($[M]^+$): 260.1407, found: 260.1409.
$[\alpha]_D^{25}$ = –19.67 (*c* 0.18, $CHCl_3$)
TLC (SIL-plate): ^{i}hexane/EtOAc = 4/1, R_f = 0.52.

Chiral-GC (Column: 30.0 m, BGB-176/BGB-15 0.25/0.25df G/618; Temp.: 220/120, 800 min. iso. 8/min. 240, 3 min. iso. /350; Gas: 0.60 bar H_2): t_R(minor) = 769.97 min, t_R(major) = 752.62 min. e.r. = 98:2 (96% ee).

Methyl 2-((1*R*,2*S*)-3-oxo-2-(4-(trifluoromethoxy)phenyl)cyclopentyl)acetate (6f)

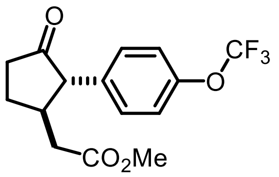

In accordance with the general procedure, the reaction was performed using enone **1ac** (121.1 mg, 0.5 mmol, 1.0 equiv.), silyl ketene acetal **2a** (141.3 mg, 0.75 mmol, 1.5 equiv.) and **IDPi 4f** (8.3 mg, 1 mol%) in PhMe at –80 °C for 24 h. The resulting silylated intermediate was subsequently treated with TsOH·H_2O. After purification by silica gel flash chromatography using ^{i}hexane/EtOAc (from 20:1 to 6:1 *v*/*v*) as eluent, the desired product **6f** was obtained as a white solid (137.6 mg, 87% yield over 2 steps). GC analysis revealed a diastereomeric ratio of 12:1.

^{1}H NMR (501 MHz, CD_2Cl_2): δ 7.28–7.23 (m, 2H), 7.20–7.17 (m, 2H), 3.55 (s, 3H), 3.09 (d, *J* = 11.8 Hz, 1H), 2.75–2.66 (m, 1H), 2.60–2.53 (m, 2H), 2.46–2.36 (m, 3H), 1.75–1.65 (m, 1H).

^{13}C NMR (126 MHz, CD_2Cl_2): δ 215.5, 171.9, 148.3, 136.3, 130.4, 120.5 (q, *J* = 256.6 Hz), 121.1, 61.0, 51.3, 41.5, 38.0, 38.0, 27.2.

^{19}F NMR (471 MHz, CD_2Cl_2): δ –58.27.

HRMS (GC-EI): calculated for $C_{15}H_{15}O_4F_3$ ($[M]^+$): 316.0917, found: 316.0922.

$[\alpha]_D^{25}$ = –141.05 (*c* 0.46, $CHCl_3$)

TLC (SIL-plate): ^{i}hexane/EtOAc = 4/1, R_f = 0.45.

Chiral-GC (Column: 25.0 m, Hydrodex-beta-TBDAc-CD 0.25/?df G/681; Temp.: 220/120, 600 min. iso. 8/min. 220, 3 min. iso. /350; Gas: 0.60 bar H_2):

For major product: t_R(minor) = 512.68 min, t_R(major) = 561.62 min. e.r. = 95:5 (90% ee); d.r. = 12:1.

General procedure for the cis-selective desilyl-protonation:

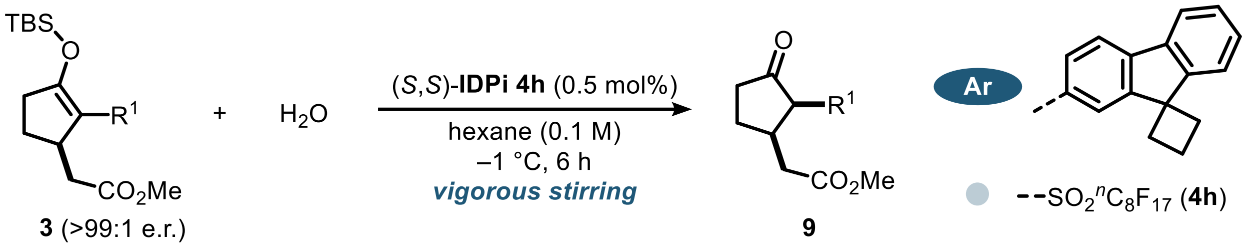

In an oven-dried screw-cap vial equipped with a magnetic stirring bar, **IDPi 4h** (1.22 mg, 0.5 mol%) and freshly distilled hexane (1 mL, 0.1 M) were added. The mixture was then transferred to a cryostat set at −1 °C, and (*R*)-enolsilane **3** (0.1 mmol, 1.0 equiv.) was added subsequently, followed by distilled water (2.7 μL, 0.15 mmol, 1.5 equiv.). The reaction was vigorously stirred for 6 h. Upon completion, the reaction mixture was directly concentrated under reduced pressure at 15 °C, and the crude product was rapidly purified via flash column chromatography using pentane/MTBE as eluent, affording the desired *cis*-product.

Note: *All reactions should be vigorously stirred to eliminate potential influence on diastereoselectivity arising from the heterogeneous system.*

Methyl 2-((1*R*,2*S*)-3-oxo-2-((*Z*)-pent-2-en-1-yl)cyclopentyl)acetate (9a)

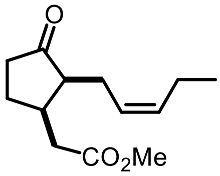

In accordance with the general procedure, the reaction was performed using (*R*)-enolsilane **3e** (33.9 mg, 0.1 mmol, 1.0 equiv.), distilled water (2.7 µL, 0.15 mmol, 1.5 equiv.) and **IDPi 4h** (1.2 mg, 0.5 mol%) in hexane at –10 °C for 6 h. Product **9b** as a colorless oil was afforded after purification via silica gel flash chromatography using pentane/MTBE (from 10:1 to 6:1 *v*/*v*) as eluent (22.2 mg, 99% yield). HPLC or NMR analysis revealed a diastereomeric ratio of 91:9.

^{1}H NMR (600 MHz, CD_2Cl_2) δ 5.46–5.40 (m, 1H), 5.36–5.30 (m, 1H), 3.66 (s, 3H), 2.85–2.76 (m, 1H), 2.41 (ddd, *J* = 15.7, 5.5, 0.7 Hz, 1H), 2.36–2.28 (m, 2H), 2.27–2.17 (m, 1H), 2.15–2.09 (m, 1H), 2.07–1.98 (m, 4H), 1.80 (dddd, *J* = 13.3, 8.6, 5.3, 4.7 Hz, 2H), 0.96 (t, *J* = 7.5 Hz, 3H).

^{13}C NMR (151 MHz, CD_2Cl_2) δ 218.8, 173.2, 133.6, 126.2, 53.0, 51.9, 36.0, 35.6, 34.1, 26.0, 23.3, 21.0, 14.3.

HRMS (GC-EI): calculated for $C_{13}H_{20}O_3$ ($[M]^+$): 224.1407, found: 224.1406.

$[\alpha]_D^{25}$ = +91.80 (*c* 0.98, $CHCl_3$)

TLC (SIL-plate): pentane/EtOAc = 4/1, R_f = 0.55 [stained with CAM].

HPLC (IG-3R, MeOH:H_2O = 70:30, 1.0 mL/min, 220 nm, 298 K):

For major product: t_R(minor) = 37.213 min, t_R(major) = 50.907 min. e.r. = 99.65:0.35 (99.3% ee), for minor product: t_R(minor) = 32.422 min, t_R(major) = 28.575 min. e.r. = 98.5:1.5 (97% ee); d.r. = 91:9.

Methyl 2-((1*R*,2*S*)-3-oxo-2-pentylcyclopentyl)acetate (9b)

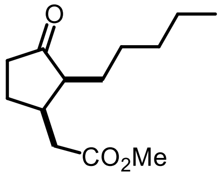

In accordance with the general procedure, the reaction was performed using (*R*)-enolsilane **3c** (34.1 mg, 0.1 mmol, 1.0 equiv.), distilled water (2.7 µL, 0.15 mmol, 1.5 equiv.) and **IDPi 4h** (1.2 mg, 0.5 mol%) in hexane at –10 °C for 6 h. Product **9a** as a colorless oil was afforded after purification via silica gel flash chromatography using pentane/MTBE (from 10:1 to 6:1 *v*/*v*) as eluent (22.6 mg, 99% yield). HPLC or NMR analysis revealed a diastereomeric ratio of 80:20.

^{1}H NMR (600 MHz, tol-d_8): δ 3.33 (s, 3H), 2.53–2.47 (m, 1H), 2.04 (dd, *J* = 15.5, 5.4 Hz, 1H), 1.82–1.76 (m, 3H), 1.71 (dd, *J* = 15.5, 10.1 Hz, 1H), 1.57–1.37 (m, 3H), 1.33–1.12 (m, 6H), 1.03–0.96 (m, 1H), 0.88 (t, *J* = 7.2 Hz, 3H).

^{13}C NMR (151 MHz, tol-d_8): δ 216.0, 172.3, 52.4, 51.0, 35.8, 35.0, 33.6, 32.3, 27.6, 25.8, 25.0, 22.9, 14.3.

HRMS (GC-EI): calculated for $C_{13}H_{22}O_3$ ($[M]^+$): 226.1563, found: 226.1565.

$[\alpha]_D^{25}$ = +27.55 (*c* 0.97, $CHCl_3$)

TLC (SIL-plate): pentane/EtOAc = 4/1, R_f = 0.5 [stained with CAM].

HPLC (IG-3, CH_3CN:H_2O = 50:50 to 70:30, 1.0 mL/min, 210 nm, 298 K):

For major product: t_R(minor) = 10.08 min, t_R(major) = 11.46 min. e.r. >99:1 (99.3% ee), for minor product: t_R(minor) = 11.26 min, t_R(major) = 8.66 min. e.r. = 96:4 (92% ee); d.r. = 80:20.

100 mmol scale process optimization of 9b:

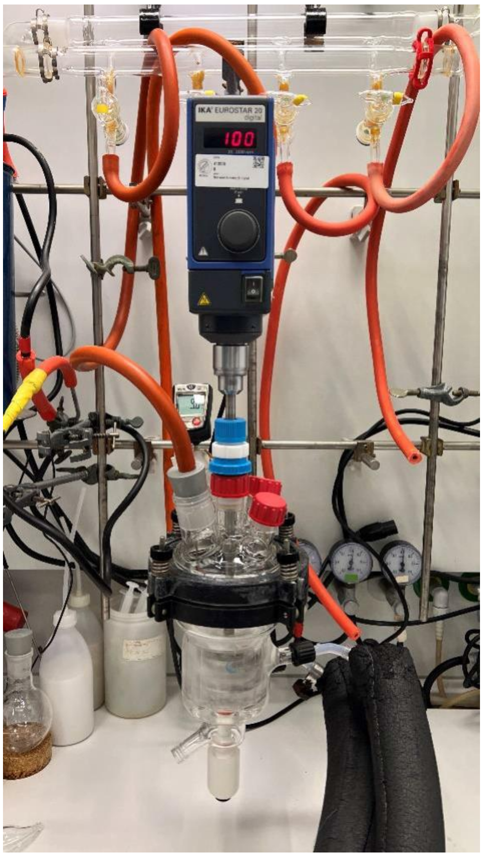

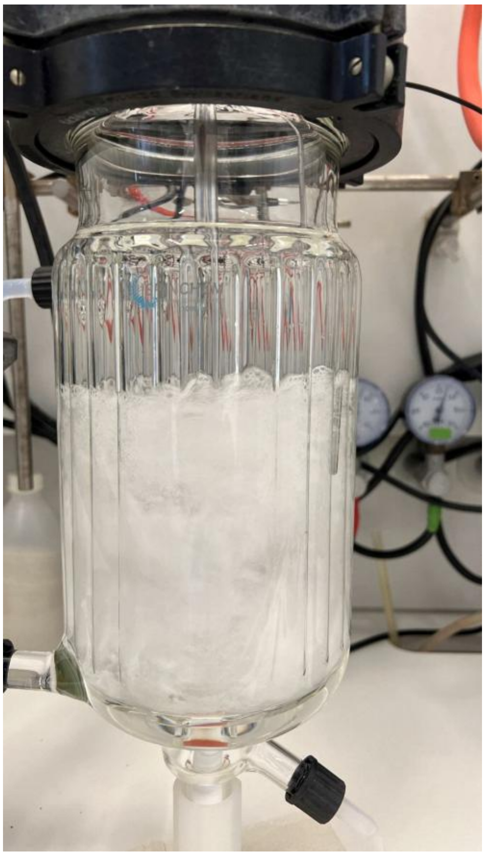

In a 1.0 L oven dried Spinchem® Vessel V3 flask [Mini Starter Kit S3] equiped with a cooling jacket and an overhead stirrer was evacuated with vaccum and argon three times, **IDPi 4h** (614 mg, 0.25 mmol, 0.25 mol%) was added to the above vessel and dissolved in *n*-hexane (833 mL, 0.12 M) at room temperature under Ar. The solution was cooled to 20 °C and after 5 min, the enolsilane **3c** (36 mL, 100 mmol) was added. 10 min later, distilled water (2.7 mL, 1.5 equiv.) was added in a single portion, and the reaction mixture was stirred at 20 °C at 2000 rpm. Reaction progress was monitored by crude NMR in benzene-d_6 without quenching with base. After 4 h, full conversion was confirmed by crude NMR, and the reaction was quenched with saturated $NaHCO_3$, followed by stirring for an additional 1 min at 1100 rpm. The mixture was then extracted with pentane (3 x 80 mL). The combined organic layer was washed with water and brine (50 mL), dried over anhydrous Na_2SO_4 and concentrated under the reduce pressure at 15 °C.

The crude product was purified by distillation (0.014 mmbar, 150 °C oil-bath temp., 80–90 °C internal thermometer temp.) in four fractions to afford the product **9b** as a colorless oil (18.91 g, 84% distilled yield).

Note: *TBSOH was first distilled at* $1.4{\times}10^{-2}$ *mbar, oil bath 75 °C, internal up to 40 °C.*

HPLC (IG-3, CH_3CN:H_2O = 50:50 to 70:30, 1.0 mL/min, 210 nm, 298 K):

For major product: t_R(minor) = 10.14 min, t_R(major) = 11.54 min. e.r. >99:1 (99.7% ee), for minor product: t_R(minor) = 11.38 min, t_R(major) = 8.68 min. e.r. = 98.6:1.4 (97.2% ee); d.r. = 80:20.

Preparation of methyl 2-((1*S*,2*R*,5*R*)-5-((*tert*-butyldimethylsilyl)oxy)-1-pentylbicyclo [3.1.0] hexan-2-yl) acetate (10)

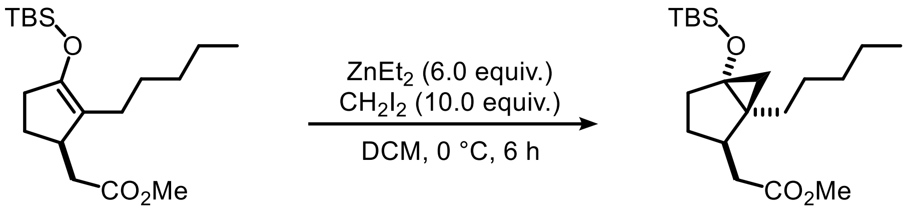

3c **10**

To a solution of the enantiopure enol silane **3c** (0.20 mmol) in dry dichloromethane (2 mL, 0.1 M) at 0 °C was added diethylzinc (1.0 M in hexane, 1.2 mL, 1.2 mmol, 6.0 equiv.) dropwise. After 15 min, diiodomethane (0.16 mL, 2.0 mmol, 10 equiv.) was introduced, resulting in the formation of a white precipitate. The reaction mixture was stirred at this temperature for 6 h, then diluted with EtOAc (10 mL). The mixture was washed sequentially with saturated NH_4Cl (5 mL) and 10 % $Na_2S_2O_3$ (5 mL), the aqueous layers were combined, and the organics were extracted with EtOAc (2 × 10 mL). The combined organic extracts were dried over $MgSO_4$, filtered, and concentrated under reduced pressure. The crude mixture was purified by flash column chromatography using pentane/Et_2O (from 30:1 to 18:1 *v*/*v*) as eluent to afford the desired product **10** as a colorless oil (61.7 mg, 87% yield).

^{1}H NMR (501 MHz, CD_2Cl_2): δ 3.62 (s, 3H), 2.55–2.47 (m, 1H), 2.41 (dd, *J* = 14.7, 5.2 Hz, 1H), 2.00–1.92 (m, 2H), 1.89–1.82 (m, 1H), 1.72 (dt, *J* = 13.1, 7.8 Hz, 1H), 1.68–1.61 (m, 1H), 1.43–1.17 (m, 7H), 0.88 (t, *J* = 7.0 Hz, 3H), 0.87 (s, 9H), 0.69–0.61 (m, 1H), 0.59 (d, *J* = 5.7 Hz, 1H), 0.25 (m, 1H), 0.10 (s, 3H), 0.07 (s, 3H).

^{13}C NMR (126 MHz, CD_2Cl_2): δ 173.9, 67.2, 51.6, 37.8, 37.0, 34.5, 33.9, 32.8, 30.3, 27.4, 26.8, 25.9, 23.2, 18.06, 18.05, 14.3, –3.3, –3.9.

HRMS (GC-EI): calculated for $C_{20}H_{39}O_3Si$ ($[M+H]^+$): 355.2663, found: 355.2664.

$[\alpha]_D^{25}$ = –19.05 (*c* 0.63, $CHCl_3$)

TLC (SIL-plate): pentane/EtOAc = 16/1, R_f = 0.57 [stained with PMA].

HPLC (IG-3R, CH_3CN:H_2O = 80:20, 1.0 mL/min, 220 nm, 298 K):

For major product: t_R(major) = 8.24 min, t_R(minor) = 8.65 min. e.r. >99:1 (98.5% ee), for minor product: t_R(major) = 7.79 min, t_R(minor) = 7.49 min. e.r. >99:1 (99% ee); d.r. = 3:1.

Preparation of benzyl 2-((1*R*,2*R*)-2-fluoro-3-oxo-2-pentylcyclopentyl)acetate (11)

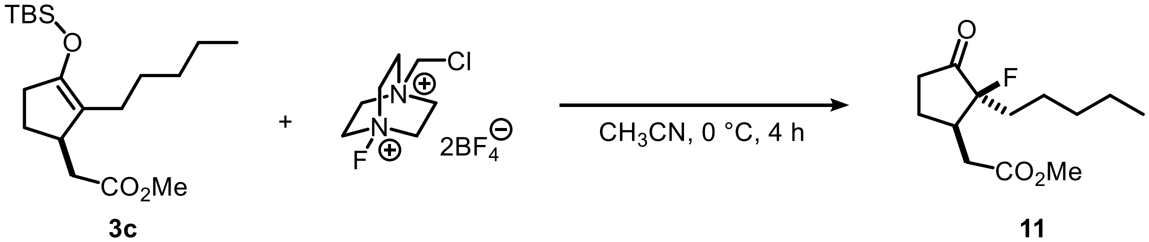

3c **11**

To a solution of the enantiopure enol silane **3c** (68 mg, 0.2 mmol) in CH_3CN (2 mL, 0.1 M) at 0 °C was added Selectfluor (78 mg, 0.22 mmol, 1.1 equiv.). The reaction mixture was maintained at this same temperature and stirred for 4 h. After the reaction was completed (monitored by TLC), the reaction was quenched with water (1 mL) and extracted with EtOAc (3 × 5 mL). The

combined organic layers were dried over Na_2SO_4, filtered through celite, and concentrated under reduced pressure. The crude material was purified by flash column chromatography using pentane/Et_2O (from 10:1 to 6:1 *v*/*v*) as eluent to afford the desired product **11** as a colorless oil (35.7 mg, 73 % yield). NMR analysis revealed a diastereomeric ratio of >20:1.

^{1}H NMR (501 MHz, CD_2Cl_2): δ 3.68 (s, 3H), 2.66–2.61 (m, 1H), 2.61–2.53 (m, 1H), 2.46–2.38 (m, 1H), 2.38–2.32 (m, 1H), 2.25–2.13 (m, 2H), 1.85–1.63 (m, 3H), 1.37–1.23 (m, 5H), 1.22–1.14 (m, 1H), 0.90–0.85 (m, 3H).

^{13}C NMR (126 MHz, CD_2Cl_2): δ 212.1 (d, *J* = 16.7 Hz), 172.8, 98.5 (d, *J* = 183.7 Hz), 52.0, 39.8 (d, *J* = 19.1 Hz), 35.0, 33.2 (d, *J* = 7.7 Hz), 32.4, 31.7 (d, *J* = 23.4 Hz), 24.4 (d, *J* = 1.5 Hz), 23.2 (d, *J* = 7.8 Hz), 22.8, 14.1.

^{19}F NMR (565 MHz, CD_2Cl_2): δ –168.66.

HRMS (ESI): calculated for $C_{13}H_{21}FO_3Na$ ($[M+Na]^+$): 267.1367, found: 267.1365.

$[\alpha]_D^{25}$ = –449.20 (*c* 0.81, $CHCl_3$)

TLC (SIL-plate): pentane/EtOAc = 5/1, R_f = 0.52.

Chiral-GC (Column: 24.0 m, Cyclodextrin-H 0.25/0.125df G/632; Temp.: 220/110, 60 min. iso. 8/min 180, 3 min. iso. /350; Gas: 0.50 bar H_2):

For major product: t_R(major) = 49.17 min, t_R(minor) = 52.56 min. e.r. >99:1 (98.7% ee), for minor product: not determined; d.r. >20:1.

5. Synthesis and Characterization of Catalysts

General procedure A for the synthesis of diol derivatives[2]

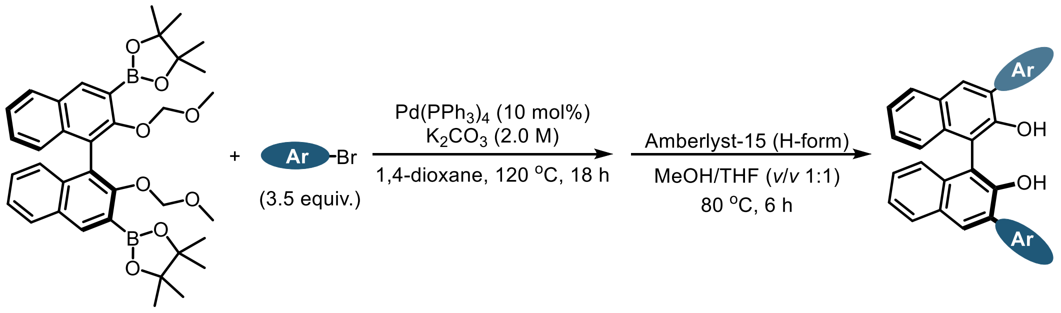

In a flame-dried two-neck round-bottom flask equipped with a condenser, 2,2'-(2,2'-bis(methoxymethoxy)-[1,1'-binaphthalene]-3,3'-diyl)bis(4,4,5,5-tetramethyl-1,3,2-dioxaborolane) (632 mg, 1.0 mmol, 1.0 equiv.), aryl bromide (3.5 mmol, 3.5 equiv.), $Pd(PPh_3)_4$ (116.1 mg, 0.1 mmol, 0.1 equiv.), and potassium carbonate (2.0 M in degassed water, 3 mL) were charged under an inert atmosphere. The reaction mixture was degassed with Ar for 15 min, after which 1,4-dioxane (10 mL) was added. The resulting mixture was heated to 120 °C and stirred for 18 h. After completion, which was monitored by TLC analysis, the reaction was cooled to room temperature and filtered through a pad of celite, washing with DCM (3 × 30 mL). The organic and aqueous layers were separated, and the aqueous phase was extracted with DCM (2 × 30 mL). The combined organic layers were washed with brine (30 mL), dried over anhydrous Na_2SO_4, filtered, and concentrated in vacuo to yield a white solid, which was used directly in the next step without further purification.

In the subsequent step, the obtained solid was dissolved in a mixture of MeOH (10 mL) and THF (10 mL). An excess of Amberlyst-15 (1.5 g) was added at room temperature, and the reaction mixture was heated to 80 °C and stirred for 6 h until TLC indicated complete consumption of the starting material. After cooling to room temperature, the reaction was quenched with water (20 mL) and extracted with DCM (2 × 20 mL). The aqueous layer was further extracted with DCM (20 mL), and the combined organic extracts were dried over anhydrous Na_2SO_4, filtered, and concentrated under reduced pressure. The resulting residue was purified by column chromatography on silica gel using a gradient of ^{i}hexane/EtOAc to afford the target diol.

General procedure B for the synthesis of diol derivatives[1]

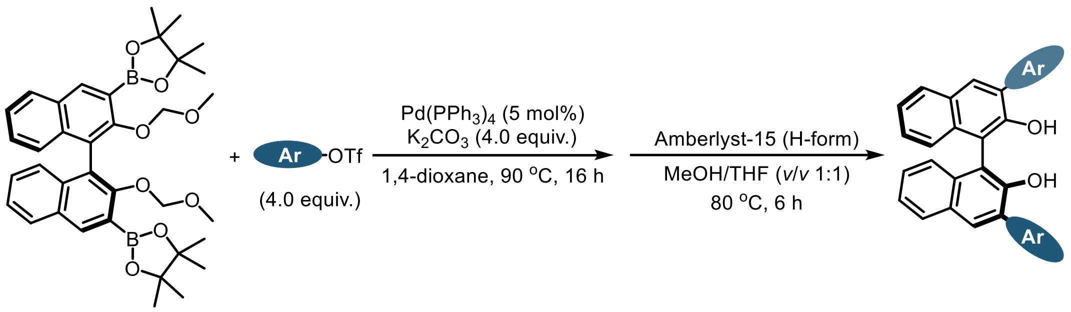

In a flame-dried two-neck round-bottom flask equipped with a condenser, 2,2'-(2,2'-bis(methoxymethoxy)-[1,1'-binaphthalene]-3,3'-diyl)bis(4,4,5,5-tetramethyl-1,3,2-dioxaborolane) (632 mg, 1.0 mmol, 1.0 equiv.), 5,6,7,8-tetrahydronaphthalen-2-yl

trifluoromethanesulfonate (1.13 g, 4.0 mmol, 4.0 equiv.), $Pd(PPh_3)_4$ (58.0 mg, 0.05 mmol, 0.05 equiv.), and potassium carbonate (556.0 mg, 4.0 mmol, 4.0 equiv.) were charged under an inert atmosphere. The reaction mixture was degassed with Ar for 15 min, after which 1,4-dioxane (10 mL) and degassed water (5 mL) were added sequentially. The resulting mixture was heated to 90 °C and stirred for 4 h. After completion, which monitored by TLC analysis, the reaction was cooled to room temperature and filtered through a pad of celite, washing with DCM (3 × 20 mL). The organic and aqueous layers were separated, and the aqueous phase was extracted with DCM (2 × 20 mL). The combined organic layers were washed with brine (30 mL), dried over anhydrous Na_2SO_4, filtered, and concentrated in vacuo to yield a white solid, which was used directly in the next step without further purification.

In the subsequent step, the obtained solid was dissolved in a mixture of MeOH (10 mL) and THF (15 mL). An excess of Amberlyst-15 (1.5 g) was added at room temperature, and the reaction mixture was heated to 80 °C and stirred for 6 h until TLC indicated complete consumption of the starting material. After cooling to room temperature, the reaction was quenched with water (20 mL) and extracted with DCM (2 × 20 mL). The aqueous layer was further extracted with DCM (20 mL), and the combined organic extracts were dried over anhydrous Na_2SO_4, filtered, and concentrated under reduced pressure. The resulting residue was purified by column chromatography on silica gel using a gradient of ^{i}hexane/EtOAc to afford the target diol.

(*S*)-3,3'-bis(4-(*tert*-butyl)phenyl)-[1,1'-binaphthalene]-2,2'-diol (S1)

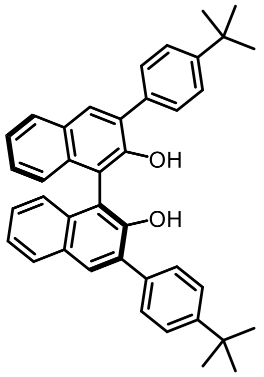

The title compound was synthesized following ***general procedure A*** using 1-bromo-4-(*tert*-butyl)benzene (746 mg, 3.5 mmol) as the starting material, the (*S*)-diol **S1** as a yellowish solid was afforded after purification via silica gel flash chromatography using ^{i}hexane/EtOAc (from 18:1 to 12:1 *v*/*v*) as eluent (457.1 mg, 83% yield over 2 steps).

^{1}H NMR (501 MHz, CD_2Cl_2): δ 8.07 (s, 2H), 7.98 (d, *J* = 8.0 Hz, 2H), 7.74–7.70 (m, 4H), 7.60–7.56 (m, 4H), 7.43 (ddd, *J* = 8.1, 6.8, 1.3 Hz, 2H), 7.35 (ddd, *J* = 8.2, 6.8, 1.3 Hz, 2H), 7.23 (d, *J* = 7.3 Hz, 2H), 5.49 (s, 2H), 1.43 (s, 18H).

^{13}C NMR (126 MHz, CD_2Cl_2): δ 150.9, 150.4, 134.6, 133.0, 131.1, 130.6, 129.6, 129.2, 128.4, 127.1, 125.5, 124.2, 124.1, 112.6, 34.5, 31.1.

HRMS (ESI, negative ions): calculated for $C_{40}H_{37}O_2$ ($[M–H]^–$): 549.2799, found: 549.2805.

(*S*)-5,5''',6,6''',7,7''',8,8'''-octahydro-[2,2':4',1'':3'',2'''-quaternaphthalene]-2'',3'-diol (S2)

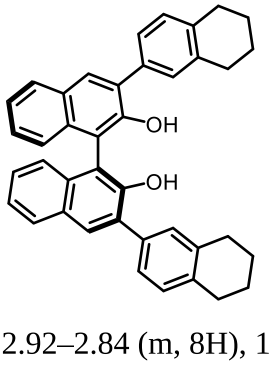

The title compound was synthesized following ***general procedure B*** using 5,6,7,8-tetrahydronaphthalen-2-yl trifluoromethanesulfonate (981 mg, 3.5 mmol) as the starting material, the (*S*)-diol **S2** as an off-white solid was afforded after purification via silica gel flash chromatography using ^{i}hexane/EtOAc (from 18:1 to 12:1 *v*/*v*) as eluent (415.5 mg, 76% yield over 2 steps).

^{1}H NMR (501 MHz, CD_2Cl_2): δ 8.02 (s, 2H), 7.96 (dt, *J* = 8.1, 0.9 Hz, 2H), 7.48–7.39 (m, 6H), 7.37–7.30 (m, 2H), 7.25–7.19 (m, 4H), 5.48 (s, 2H), 2.92–2.84 (m, 8H), 1.93–1.84 (m, 8H).

^{13}C NMR (126 MHz, CD_2Cl_2): δ 150.2, 137.5, 137.1, 134.5, 133.0, 130.9, 130.8, 130.0, 129.5, 129.2, 128.3, 126.9, 126.6, 124.2, 124.1, 112.8, 29.5, 29.2, 23.2.
HRMS (ESI, negative ions): calculated for $C_{40}H_{33}O_2$ ($[M–H]^–$): 545.2486, found: 545.2492.

(*S*)-3,3'-bis(3-(*tert*-butyl)phenyl)-[1,1'-binaphthalene]-2,2'-diol (S3)

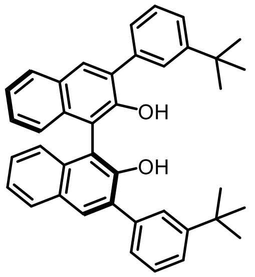

The title compound was synthesized following ***general procedure A*** using 1-bromo-3-(*tert*-butyl)benzene (746 mg, 3.5 mmol) as the starting material, the (*S*)-diol **S3** as a white solid was afforded after purification via silica gel flash chromatography using ^{i}hexane/EtOAc (from 18:1 to 12:1 *v*/*v*) as eluent (479.1 mg, 87% yield over 2 steps).
^{1}H NMR (501 MHz, CD_2Cl_2): δ 8.07 (s, 2H), 7.99 (d, *J* = 8.1 Hz, 2H), 7.79 (t, *J* = 1.8 Hz, 2H), 7.58 (dt, *J* = 7.2, 1.6 Hz, 2H), 7.52–7.42 (m, 6H), 7.38–7.34 (m, 2H), 7.25 (d, *J* = 8.4 Hz, 2H), 5.50 (s, 2H), 1.43 (s, 18H).
^{13}C NMR (126 MHz, CD_2Cl_2): δ 151.6, 150.2, 137.1, 133.1, 131.2, 131.1, 129.5, 128.4, 128.2, 127.1, 126.74, 126.71, 124.8, 124.20, 124.16, 112.8, 34.7, 31.1.
HRMS (ESI, negative ions): calculated for $C_{40}H_{37}O_2$ ($[M–H]^–$): 549.2799, found: 549.2806.

(*S*)-3,3'-di([1,1'-biphenyl]-3-yl)-[1,1'-binaphthalene]-2,2'-diol (S4)

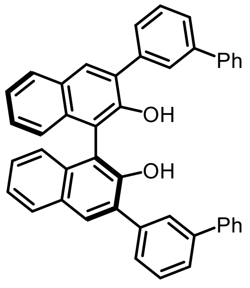

The title compound was synthesized following ***general procedure A*** using 3-bromo-1,1'-biphenyl (816 mg, 3.5 mmol) as the starting material, the (*S*)-diol **S4** as a white solid was afforded after purification via silica gel flash chromatography using ^{i}hexane/EtOAc (from 18:1 to 10:1 *v*/*v*) as eluent (413.5 mg, 70% yield over 2 steps).
^{1}H NMR (501 MHz, CD_2Cl_2): δ 8.15 (s, 2H), 8.03–8.00 (m, 4H), 7.78 (dt, *J* = 7.6, 1.5 Hz, 2H), 7.74–7.69 (m, 6H), 7.62 (t, *J* = 7.6 Hz, 2H), 7.52–7.37 (m, 11H), 7.27 (dd, *J* = 8.4, 1.1 Hz, 2H), 5.54 (s, 2H).
^{13}C NMR (126 MHz, CD_2Cl_2): δ 150.3, 141.4, 140.9, 138.1, 133.1, 131.5, 130.6, 129.6, 128.9, 128.8, 128.53, 128.49, 128.46, 127.5, 127.3, 127.2, 126.4, 124.3, 124.1, 112.5.
HRMS (ESI, negative ions): calculated for $C_{44}H_{29}O_2$ ($[M–H]^–$): 589.2173, found: 589.2180.

(*S*)-3,3'-di(spiro[cyclobutane-1,9'-fluoren]-2'-yl)-[1,1'-binaphthalene]-2,2'-diol (S5)

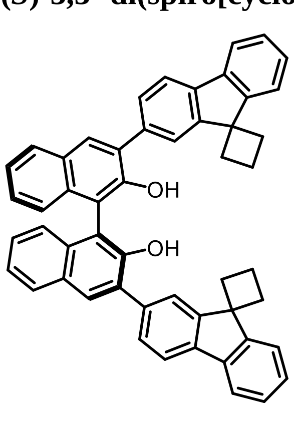

The title compound was synthesized following ***general procedure A*** using 2'-bromospiro[cyclobutane-1,9'-fluorene] (998 mg, 3.5 mmol) as the starting material, the (*S*)-diol **S5** as a white solid was afforded after purification via silica gel flash chromatography using ^{i}hexane/EtOAc (from 18:1 to 12:1 *v*/*v*) as eluent (576.7 mg, 83% yield over 2 steps).
^{1}H NMR (501 MHz, CD_2Cl_2): δ 8.24–8.18 (m, 4H), 8.07–8.03 (m, 2H), 7.90–7.84 (m, 4H), 7.81–7.75 (m, 4H), 7.48 (ddd, *J* = 8.1, 6.8, 1.3 Hz, 2H), 7.46–7.39 (m, 6H), 7.32 (d, *J* = 8.3 Hz, 2H), 5.62 (s, 2H), 2.81–2.75 (m, 4H), 2.74–2.67 (m, 4H), 2.48 (p, *J* = 8.5 Hz, 4H).
^{13}C NMR (126 MHz, CD_2Cl_2): δ 152.7, 152.6, 150.3, 138.9, 138.8, 136.8, 133.1, 131.22, 131.17, 129.6, 128.6, 128.4, 127.8, 127.2, 127.1, 124.28, 124.25, 124.2, 122.8, 119.6, 119.4, 112.8, 52.1, 33.29, 33.27, 16.9.
HRMS (ESI, negative ions): calculated for $C_{52}H_{37}O_2$ ($[M–H]^–$): 693.2799, found: 693.2810.

General procedure A for the synthesis of imidodiphosphorimidates (IDPis)

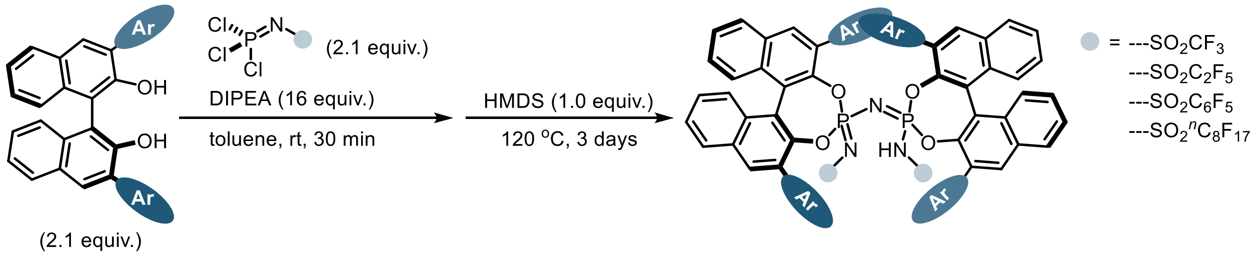

Under an argon atmosphere, an oven-dried Schlenk tube was charged with a suspension of the (*S*)-diol (0.21 mmol, 2.1 equiv.) in toluene (0.8 mL). Subsequently, (substituted sulfonyl)phosphorimidoyl trichloride (0.21 mmol, 2.1 equiv.) and DIPEA (1.6 mmol, 16 equiv.) were added, and the resulting mixture was stirred at room temperature for 30 min. Neat hexamethyl disilazane (HMDS, 21 μL, 0.1 mmol, 1.0 equiv.) was then introduced dropwise, with stirring continued for an additional 20 min. The reaction vessel was sealed and heated to 120 °C for 3 days. After cooling to room temperature, 10% aqueous HCl was added, and the mixture was extracted with DCM. The combined organic layers were washed with brine, dried over anhydrous Na_2SO_4, and concentrated under reduced pressure to yield a crude salt. The crude product was purified by column chromatography on silica gel to afford the imidodiphosphorimidate salt.
To convert the salt into the corresponding IDPi Brønsted acid, the purified salt was dissolved in 5 mL of DCM and treated with 10 mL of 6.0 M aqueous HCl. After vigorous stirring for 20 min, the organic layer was separated, and the aqueous phase was extracted repeatedly with DCM until TLC indicated complete removal of the product. The combined organic layers were concentrated under reduced pressure, and the residue was dried under high vacuum at room temperature overnight to yield the final IDPi acid.

General procedure B for the synthesis of imidodiphosphorimidates (IDPis)

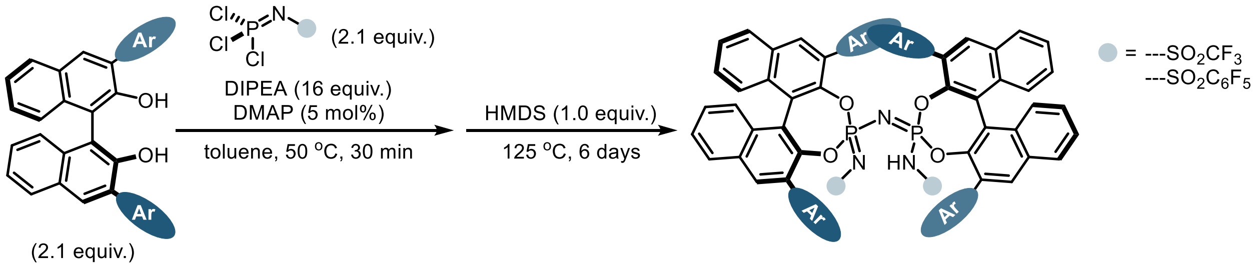

Under an argon atmosphere, an oven-dried Schlenk tube was charged with a suspension of the (*S*)-diol (0.21 mmol, 2.1 equiv.) in toluene (0.8 mL). Subsequently, (substituted sulfonyl)phosphorimidoyl trichloride (0.21 mmol, 2.1 equiv.), DMAP (0.005 mmol, 5 mol%) and DIPEA (1.6 mmol, 16 equiv.) were added, and the resulting mixture was stirred for 30 min at 50 °C. Neat hexamethyl disilazane (HMDS, 21 μL, 0.1 mmol, 1.0 equiv.) was then introduced dropwise, with stirring continued for an additional 20 min. The reaction vessel was sealed and heated to 125 °C for 6 days. After cooling to room temperature, 10% aqueous HCl was added, and the mixture was extracted with DCM. The combined organic layers were washed with brine, dried over anhydrous Na_2SO_4, and concentrated under reduced pressure to yield a crude salt. The

crude product was purified by column chromatography on silica gel to afford the imidodiphosphorimidate salt.

To convert the salt into the corresponding IDPi Brønsted acid, the purified salt was dissolved in 5 mL of DCM and treated with 10 mL of 6.0 M aqueous HCl. After vigorous stirring for 20 min, the organic layer was separated, and the aqueous phase was extracted repeatedly with DCM until TLC indicated complete removal of the product. The combined organic layers were concentrated under reduced pressure, and the residue was dried under high vacuum at room temperature overnight to yield the final IDPi acid.

(*S*,*S*)-imidodiphosphorimidate 4a

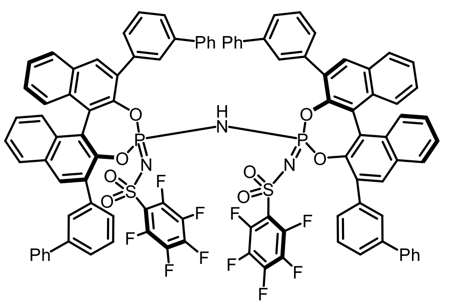

The title compound was synthesized following ***general procedure A*** using (*S*)-diol **S4** (124.1 mg, 0.21 mmol) as the starting material, the catalyst **4a** as a white solid was afforded after chromatographic purification using ^{i}hexane:DCM (from 10:1 to 3:1) as eluent, and acidification (127.4 mg, 73% yield). [Chromatography conditions: pentane/EtOAc 16:1 to 5:1 *v*/*v*]

^{1}H NMR (501 MHz, CD_2Cl_2): δ 8.16 (s, 2H), 8.05 (d, *J* = 8.2 Hz, 2H), 8.00 (d, *J* = 8.2 Hz, 2H), 7.83 (ddd, *J* = 8.1, 6.8, 1.1 Hz, 2H), 7.75 (s, 2H), 7.62–7.56 (m, 8H), 7.46–7.43 (m, 10H), 7.41–7.31 (m, 10H), 7.29–7.24 (m, 10H), 7.09–7.05 (m, 4H), 6.96 (t, *J* = 7.7 Hz, 2H), 6.56 (d, *J* = 7.9 Hz, 2H), 5.96 (brs, 1H).
^{13}C NMR (126 MHz, CD_2Cl_2): δ 143.7, 142.9, 141.3, 140.9, 140.53, 140.48, 136.2, 136.0, 133.0, 131.8, 131.7, 131.63, 131.59, 131.2, 129.1, 129.0, 128.7, 128.6, 128.4, 128.3, 128.2, 128.0, 127.9, 127.8, 127.5, 127.4, 127.3, 127.2, 127.1, 126.83, 126.78, 126.6, 126.5, 126.2, 123.3, 122.1.
^{19}F NMR (471 MHz, CD_2Cl_2): δ –136.5 (d, *J* = 27.3 Hz), –146.2– –147.7 (m), –159.7 (t, *J* = 21.6 Hz).
^{31}P NMR (203 MHz, CD_2Cl_2): δ –15.15.
HRMS (ESI, negative ions): calculated for $C_{100}H_{56}O_8N_3F_{10}S_2P_2$ ([M–H]$^{-}$): 1742.2830, found: 1742.2834.

(*S*,*S*)-imidodiphosphorimidate 4b

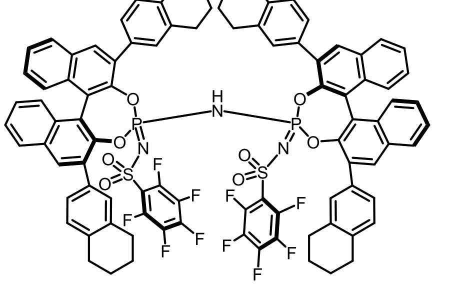

The title compound was synthesized following ***general procedure A*** using (*S*)-diol **S2** (114.8 mg, 0.21 mmol) as the starting material, the catalyst **4b** as a white solid was afforded after chromatographic purification and acidification (109.3 mg, 66% yield). [Chromatography conditions: pentane/DCM/MTBE 8:2:0.5 to 4:3:0.5 *v*/*v*/*v*]

^{1}H NMR (501 MHz, CD_2Cl_2): δ 8.15 (s, 2H), 8.06 (d, *J* = 8.3 Hz, 2H), 7.97 (d, *J* = 8.2 Hz, 2H), 7.83–7.74 (m, 2H), 7.62–7.54 (m, 6H), 7.39–7.32 (m, 2H), 7.32–7.27 (m, 2H), 7.10–7.03 (m, 6H), 7.03–6.98 (m, 2H), 6.59 (q, *J* = 8.0 Hz, 4H), 6.49 (s, 2H), 2.96–2.68 (m, 9H), 2.60–2.47 (m, 6H), 2.13–1.99 (m, 2H), 1.88–1.76 (m, 9H), 1.64–1.41 (m, 3H).

^{13}C NMR (126 MHz, CD_2Cl_2): δ 137.2, 137.1, 136.9, 136.7, 133.6, 133.3, 132.8, 132.6, 131.9, 131.8, 131.5, 131.4, 130.8, 129.8, 129.5, 129.0, 128.9, 128.4, 128.1, 127.3, 126.8, 126.7, 126.64, 126.57, 126.5, 126.4, 126.3, 122.2, 29.2, 29.1, 29.07, 29.03, 23.14, 23.10, 22.94, 22.87.
^{19}F NMR (471 MHz, CD_2Cl_2): −136.3 (d, *J* = 27.2 Hz), –147.5 (t, *J* = 25.4 Hz), –160.3 (t, *J* = 24.2 Hz).
^{31}P NMR (203 MHz, CD_2Cl_2): δ –15.84.
HRMS (ESI, negative ions): calculated for $C_{92}H_{64}O_8N_3F_{10}S_2P_2$ ($[M–H]^–$): 1654.3456, found: 1654.3458.

(*S*,*S*)-imidodiphosphorimidate 4c

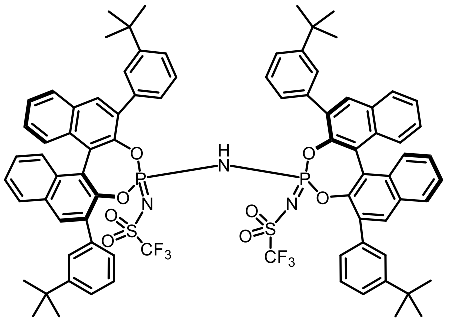

The title compound was synthesized following ***general procedure B*** using (*S*)-diol **S3** (115.7 mg, 0.21 mmol) as the starting material, the catalyst **4c** as a white solid was afforded after chromatographic purification and acidification (67.6 mg, 46% yield). [Chromatography conditions: pentane/EtOAc 20:1 to 6:1 *v*/*v*]
^{1}H NMR (501 MHz, $CDCl_3$): δ 8.14 (s, 2H), 8.09 (d, *J* = 8.3 Hz, 2H), 8.01 (d, *J* = 8.2 Hz, 2H), 7.76 (t, *J* = 7.5 Hz, 2H), 7.73 (d, *J* = 8.5 Hz, 2H), 7.62–7.55 (m, 6H), 7.44 (d, *J* = 8.5 Hz, 2H), 7.40–7.36 (m, 4H), 7.31–7.28 (m, 4H), 7.14 (s, 2H), 7.11 (t, *J* = 7.8 Hz, 2H), 6.84–6.78 (m, 4H), 5.65 (d, *J* = 7.5 Hz, 2H), 1.37 (s, 18H), 1.27 (s, 18H).
^{13}C NMR (126 MHz, CD_2Cl_2): δ 151.7, 150.6, 135.5, 135.1, 134.3, 132.1, 132.0, 131.9, 131.7, 131.5, 128.8, 128.6, 127.4, 127.3, 126.95, 126.86, 126.8, 126.4, 126.3, 125.1, 123.0, 121.9, 34.6, 34.5, 31.03, 30.96.
^{19}F NMR (471 MHz, $CDCl_3$): δ –78.0.
^{31}P NMR (203 MHz, $CDCl_3$): δ –16.38.
HRMS (ESI, negative ions): calculated for $C_{82}H_{72}O_8N_3F_6S_2P_2$ ($[M–H]^–$): 1466.4146, found: 1466.4146.

(*S*,*S*)-imidodiphosphorimidate 4d

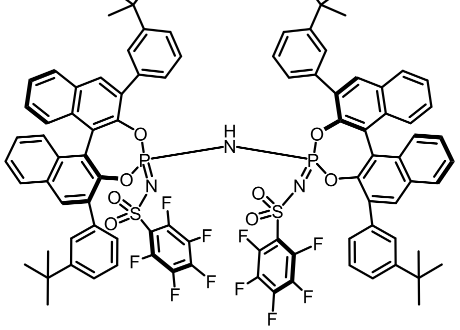

The title compound was synthesized following ***general procedure B*** using (*S*)-diol **S3** (115.7 mg, 0.21 mmol) as the starting material, the catalyst **4d** as a white solid was afforded after chromatographic purification and acidification (58.3 mg, 35% yield). [Chromatography conditions: pentane/EtOAc 20:1 to 10:1 *v*/*v*]
^{1}H NMR (501 MHz, CD_2Cl_2): δ 8.13 (s, 2H), 8.07 (d, *J* = 8.2 Hz, 2H), 7.98 (d, *J* = 8.2 Hz, 2H), 7.72 (ddd, *J* = 8.1, 5.9, 2.0 Hz, 2H), 7.63 (t, *J* = 1.9 Hz, 2H), 7.60 (ddd, *J* = 8.1, 6.8, 1.1 Hz, 2H), 7.48–7.43 (m, 4H), 7.36–7.32 (m, 4H), 7.28 (ddd, *J* = 7.8, 2.0, 1.2 Hz, 2H), 7.25–7.22 (m, 4H), 7.19 (ddd, *J* = 8.0, 2.0, 1.1 Hz, 2H), 7.12 (t, *J* = 7.7 Hz, 2H), 7.08–7.05 (m, 2H), 6.70 (t, *J* = 7.8 Hz, 2H), 6.60 (brs, 1H), 6.19–6.15 (m, 2H), 1.34 (s, 18H), 1.25 (s, 18H).
^{13}C NMR (126 MHz, CD_2Cl_2): δ 151.3, 150.8, 143.94, 143.89, 143.8, 142.9, 142.93, 142.90, 142.8, 135.3, 135.2, 134.0, 133.9, 131.8, 131.61, 131.56, 131.4, 128.7, 128.3, 127.9, 127.0, 126.9,

126.84, 126.77, 126.7, 126.6, 126.5, 126.4, 126.1, 124.53, 124.48, 122.8, 122.2, 34.6, 34.5, 31.02, 31.00.

^{19}F NMR (471 MHz, CD_2Cl_2): δ –136.1 (d, *J* = 26.9 Hz), –146.96 (t, *J* = 23.6 Hz), –159.6– –160.8 (m).

^{31}P NMR (203 MHz, CD_2Cl_2): δ –15.54.

HRMS (ESI, negative ions): calculated for $C_{92}H_{72}O_8N_3F_{10}S_2P_2$ ([M–H]$^-$): 1462.4082, found: 1662.4086.

(*S*,*S*)-imidodiphosphorimidate 4e

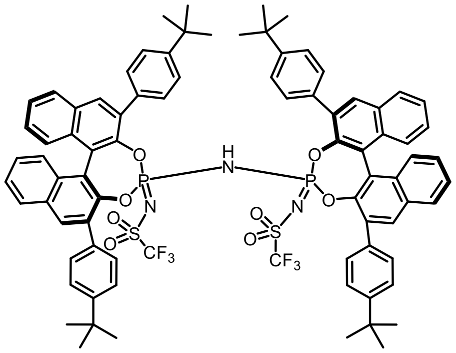

The title compound was synthesized following ***general procedure A*** using (*S*)-diol **S1** (115.7 mg, 0.21 mmol) as the starting material, the catalyst **4e** as a white solid was afforded after chromatographic purification and acidification (111.6 mg, 76% yield). [Chromatography conditions: pentane/EtOAc 16:1 to 6:1 *v*/*v*]

^{1}H NMR (501 MHz, CD_2Cl_2): δ 8.19 (s, 2H), 8.11 (dd, *J* = 16.5, 8.3 Hz, 4H), 7.86 (dt, *J* = 8.1, 4.0 Hz, 2H), 7.67 (d, *J* = 3.3 Hz, 4H), 7.61 (ddd, *J* = 8.1, 5.7, 2.3 Hz, 2H), 7.41–7.38 (m, 8H), 7.31–7.27 (m, 6H), 7.05 (d, *J* = 8.5 Hz, 4H), 6.61 (d, *J* = 8.5 Hz, 4H), 5.96 (s, 1H), 1.33 (s, 18H), 1.12 (s, 18H).

^{13}C NMR (126 MHz, CD_2Cl_2): δ 151.1, 150.8, 143.6, 143.0, 133.2, 133.0, 132.6, 132.3, 132.2, 132.0, 131.9, 131.6, 131.4, 131.2, 129.5, 128.8, 128.7, 127.2, 127.1, 126.8, 126.7, 126.4, 125.3, 124.6, 123.3, 122.0, 34.4, 34.3, 30.85, 30.81, 29.7.

^{19}F NMR (471 MHz, CD_2Cl_2): δ –78.5.

^{31}P NMR (203 MHz, CD_2Cl_2): δ –16.33.

HRMS (ESI, negative ions): calculated for $C_{82}H_{72}O_8N_3F_6S_2P_2$ ([M–H]$^-$): 1466.4146, found: 1466.4144.

(*S*,*S*)-imidodiphosphorimidate 4f

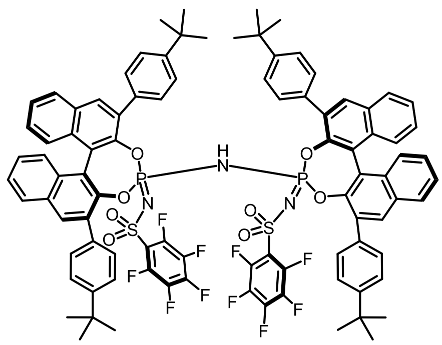

The title compound was synthesized following ***general procedure A*** using (*S*)-diol **S1** (115.7 mg, 0.21 mmol) as the starting material, the catalyst **4f** as a white solid was afforded after chromatographic purification and acidification (120.0 mg, 72% yield). [Chromatography conditions: pentane/EtOAc 16:1 to 10:1 *v*/*v*]

^{1}H NMR (501 MHz, CD_2Cl_2): δ 8.25 (d, *J* = 8.2 Hz, 2H), 8.11 (s, 2H), 8.04 (d, *J* = 8.3 Hz, 2H), 7.95 (s, 2H), 7.77 (ddd, *J* = 8.1, 6.8, 1.1 Hz, 2H), 7.62 (ddd, *J* = 8.2, 5.2, 2.7 Hz, 2H), 7.55 (ddd, *J* = 8.3, 6.8, 1.3 Hz, 2H), 7.47–7.45 (m, 8H), 7.42–7.39 (m, 6H), 6.78 (d, *J* = 8.4 Hz, 4H), 6.62 (d, *J* = 8.5 Hz, 4H), 1.29 (s, 18H), 0.95 (s, 18H).

^{13}C NMR (126 MHz, CD_2Cl_2): δ 151.0, 150.2, 143.43, 143.39, 143.3, 134.1, 133.0, 132.8, 132.4, 132.0, 131.69, 131.65, 131.2, 131.1, 130.4, 129.2, 128.84, 128.80, 128.3, 127.2, 126.79, 126.75, 126.7, 126.5, 125.8, 124.6, 123.2, 122.5, 34.4, 34.2, 30.82, 30.78.

^{19}**F NMR** (471 MHz, CD_2Cl_2): δ –135.6 (d, *J* = 27.3 Hz), –146.4 (t, *J* = 25.6 Hz), –160.3– –160.6 (m).
^{31}**P NMR** (203 MHz, CD_2Cl_2): δ –8.72.
HRMS (ESI, negative ions): calculated for $C_{92}H_{72}O_8N_3F_{10}S_2P_2$ ([M–H]$^-$): 1662.4082, found: 1662.4090.

(*S*,*S*)-imidodiphosphorimidate 4g

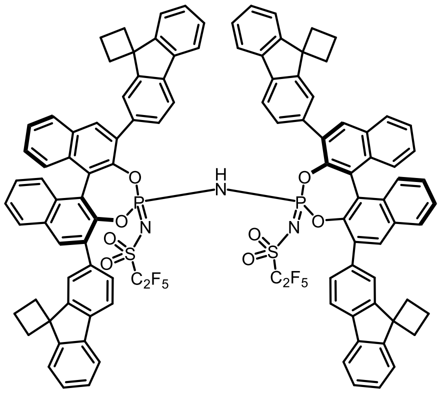

The title compound was synthesized following ***general procedure A*** using (*S*)-diol **S5** (145.9 mg, 0.21 mmol) as the starting material, the catalyst **4g** as a white solid was afforded after chromatographic purification and acidification (113.3 mg, 61% yield). [Chromatography conditions: first pentane/DCM 10:1 *v*/*v*; second: pentane/MTBE 10:1 to 2:1 *v*/*v*]
^{1}**H NMR** (501 MHz, CD_2Cl_2): δ 8.20 (s, 2H), 8.14 (dd, *J* = 12.4, 8.2 Hz, 4H), 7.95 (t, *J* = 7.5 Hz, 2H), 7.92–7.88 (m, 4H), 7.84 (d, *J* = 7.5 Hz, 2H), 7.81–7.77 (m, 2H), 7.71 (d, *J* = 7.5 Hz, 2H), 7.69–7.65 (m, 2H), 7.62–7.58 (m, 4H), 7.54–7.43 (m, 6H), 7.38–7.21 (m, 12H), 2.86–2.78 (m, 2H), 2.74–2.56 (m, 8H), 2.46–2.27 (m, 12H), 2.23–2.17 (m, 2H).
^{13}**C NMR** (126 MHz, CD_2Cl_2): δ 153.0, 152.7, 152.4, 151.9, 143.8, 142.7, 139.5, 138.7, 138.6, 138.5, 135.1, 134.6, 134.5, 134.4, 133.7, 132.2, 131.9, 131.8, 131.7, 131.6, 131.4, 129.3, 129.0, 128.7, 128.6, 127.9, 127.6, 127.3, 127.0, 126.9, 126.8, 126.7, 126.59, 126.56, 123.7, 123.4, 122.5, 122.4, 121.9, 119.6, 119.0, 118.6, 118.0, 52.0, 51.92, 51.90, 33.2, 32.9, 32.7, 32.5, 32.4, 16.8, 16.7.
^{19}**F NMR** (471 MHz, CD_2Cl_2): δ –79.3, –115.9, –116.1.
^{31}**P NMR** (203 MHz, CD_2Cl_2): δ –17.87.
HRMS (ESI, negative ions): calculated for $C_{108}H_{72}O_8N_3F_{10}S_2P_2$ ([M–H]$^-$): 1854.4082, found: 1854.4078.

(*S*,*S*)-imidodiphosphorimidate 4h

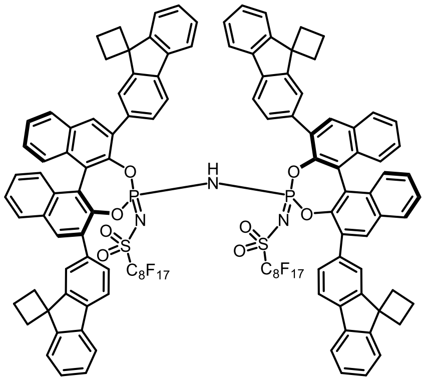

The title compound was synthesized following ***general procedure A*** using (*S*)-diol **S5** (145.9 mg, 0.21 mmol) as the starting material, the catalyst **4h** as a white solid was afforded after chromatographic purification and acidification (134.8 mg, 69% yield). [Chromatography conditions: first pentane/DCM 10:1 *v*/*v*; second: pentane/MTBE 10:1 to 2:1 *v*/*v*]
^{1}**H NMR** (501 MHz, CD_2Cl_2): δ 8.20 (s, 2H), 8.14 (d, *J* = 8.3 Hz, 2H), 8.08 (d, *J* = 8.2 Hz, 2H), 7.95 (t, *J* = 7.5 Hz, 2H), 7.91–7.88 (m, 4H), 7.82–7.77 (m, 4H), 7.69 (d, *J* = 7.6 Hz, 2H), 7.67–7.63 (m, 2H), 7.60 (d, *J* = 7.5 Hz, 2H), 7.56 (s, 2H), 7.50 (d, *J* = 7.9 Hz, 2H), 7.45–7.42 (m, 4H), 7.36 (d, *J* = 7.5 Hz, 2H), 7.34–7.18 (m, 16H), 7.15 (s, 2H), 6.79 (d, *J* = 7.9 Hz, 2H), 6.67 (d, *J* = 8.0 Hz, 2H), 6.37 (dd, *J* = 8.0, 1.7 Hz, 2H), 2.80 (q, *J* = 9.3 Hz, 2H), 2.72–2.66 (m, 2H), 2.65–2.56 (m, 6H), 2.38–2.10 (m, 14H).

^{13}**C NMR** (126 MHz, CD_2Cl_2): δ 153.0, 152.6, 152.4, 152.1, 143.9, 142.8, 139.5, 138.8, 138.7, 138.5, 135.1, 134.3, 134.2, 133.4, 132.2, 132.0, 131.8, 131.62, 131.56, 131.2, 129.5, 129.1, 128.7, 128.5, 127.9, 127.5, 127.3, 127.0, 126.81, 126.75, 126.7, 126.5, 126.4, 123.7, 123.42, 123.36, 122.4, 122.3, 122.0, 119.4, 119.0, 118.6, 118.1, 51.92, 51.88, 33.2, 33.0, 32.7, 32.4, 16.7, 16.6.
^{19}**F NMR** (471 MHz, CD_2Cl_2): −81.1 (t, *J* = 9.4 Hz), −111.8 (qt, *J* = 143.3 Hz, *J* = 18.8 Hz), −120.3, −121.7, −122.1, −122.9, −126.3.
^{31}**P NMR** (203 MHz, CD_2Cl_2): δ –17.18.
HRMS (ESI, negative ions): calculated for $C_{120}H_{72}O_8N_3F_{34}S_2P_2$ ($[M–H]^–$): 2454.3699, found: 2454.3718.

(*S*,*S*)-imidodiphosphorimidate 4i

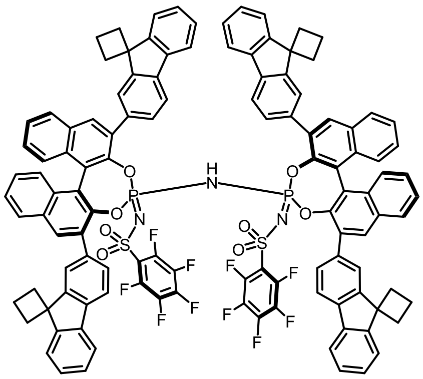

The title compound was synthesized following ***general procedure A*** using (*S*)-diol **S5** (145.9 mg, 0.21 mmol) as the starting material, the catalyst **4i** as a white solid was afforded after chromatographic purification and acidification (140.0 mg, 57% yield). [Chromatography conditions: first pentane/DCM 10:1 *v*/*v*; second: pentane/MTBE 12:1 to 3:1 *v*/*v*]
^{1}**H NMR** (501 MHz, CD_2Cl_2): δ 8.20 (s, 2H), 8.12 (d, *J* = 8.2 Hz, 2H), 8.07 (d, *J* = 8.2 Hz, 2H), 7.89–7.82 (m, 4H), 7.78 (d, *J* = 7.5 Hz, 2H), 7.73 (d, *J* = 1.6 Hz, 2H), 7.71–7.63 (m, 8H), 7.52–7.46 (m, 4H), 7.44–7.38 (m, 2H), 7.36–7.17 (m, 14H), 6.97 (d, *J* = 8.0 Hz, 2H), 6.76 (dd, *J* = 8.0, 1.7 Hz, 2H), 6.59 (dd, *J* = 7.9, 1.6 Hz, 2H), 5.66 (brs, 1H), 3.00–2.89 (m, 2H), 2.75–2.66 (m, 2H), 2.63–2.49 (m, 4H), 2.43–2.24 (m, 16H).
^{13}**C NMR** (126 MHz, CD_2Cl_2): δ 152.9, 152.3, 152.13, 152.07, 144.0 (t, *J* = 5.4 Hz), 143.0 (t, *J* = 5.4 Hz), 142.9, 139.1, 138.62, 138.59, 138.4, 135.0, 134.6, 133.9, 133.7, 131.9, 131.8, 131.7, 131.6, 131.4, 131.2, 129.2, 128.7, 127.7, 127.4, 127.3, 126.9, 126.84, 126.80, 126.70, 126.66, 126.6, 126.5, 124.1, 123.2, 123.1, 122.45, 122.40, 119.5, 119.3, 118.9, 118.3, 51.9, 51.6, 33.3, 33.2, 32.9, 32.6, 16.71, 16.69.
^{19}**F NMR** (471 MHz, CD_2Cl_2): δ –136.4 (d, *J* = 25.3 Hz), –146.7, –160.0 (t, *J* = 23.6 Hz).
^{31}**P NMR** (203 MHz, CD_2Cl_2): δ –15.65.
HRMS (ESI, negative ions): calculated for $C_{116}H_{72}O_8N_3F_{10}S_2P_2$ ($[M–H]^–$): 1950.4082, found: 1950.4107.

6. HPLC Traces of the Products

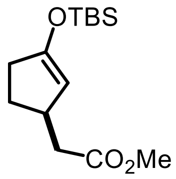

HPLC data of *rac*-**3a**

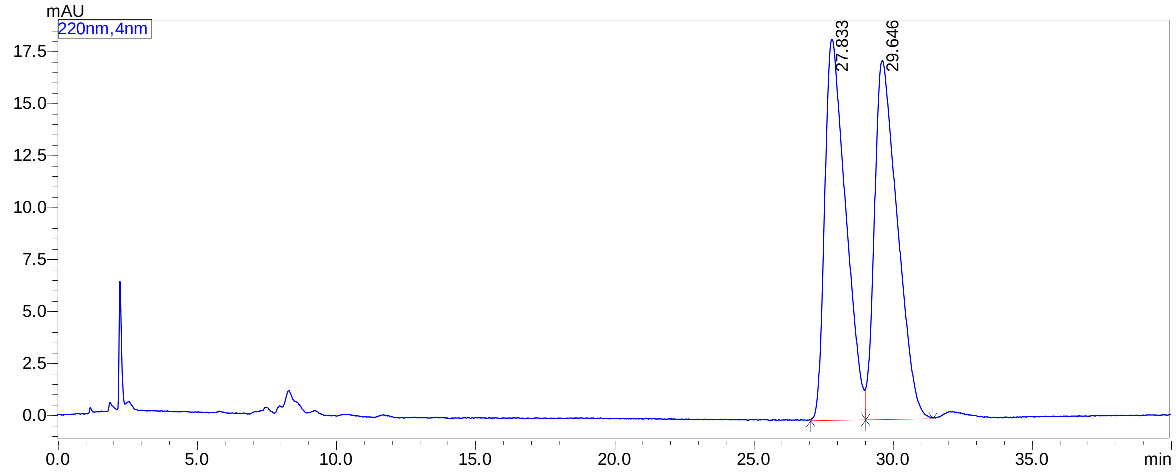

Peak#	Ret. Time	Area%
1	27.833	49.281
2	29.646	50.719
Total		100.000

HPLC data of (*R*)-**3a**

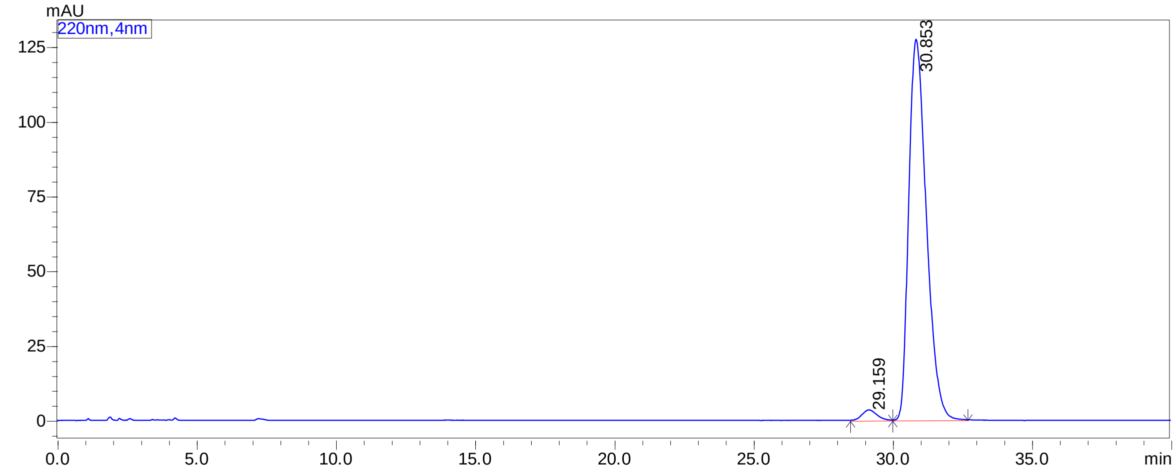

Peak#	Ret. Time	Area%
1	29.159	2.151
2	30.853	97.849
Total		100.000

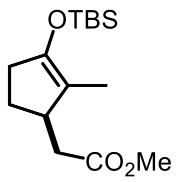

HPLC data of *rac*-**3b**

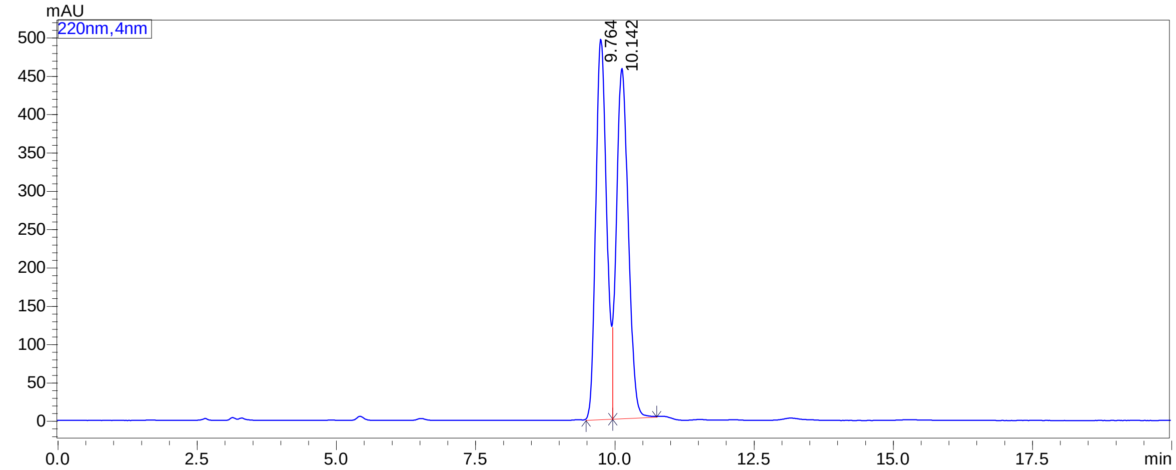

Peak#	Ret. Time	Area%
1	9.764	49.509
2	10.142	50.491
Total		100.000

HPLC data of (*R*)-**3b**

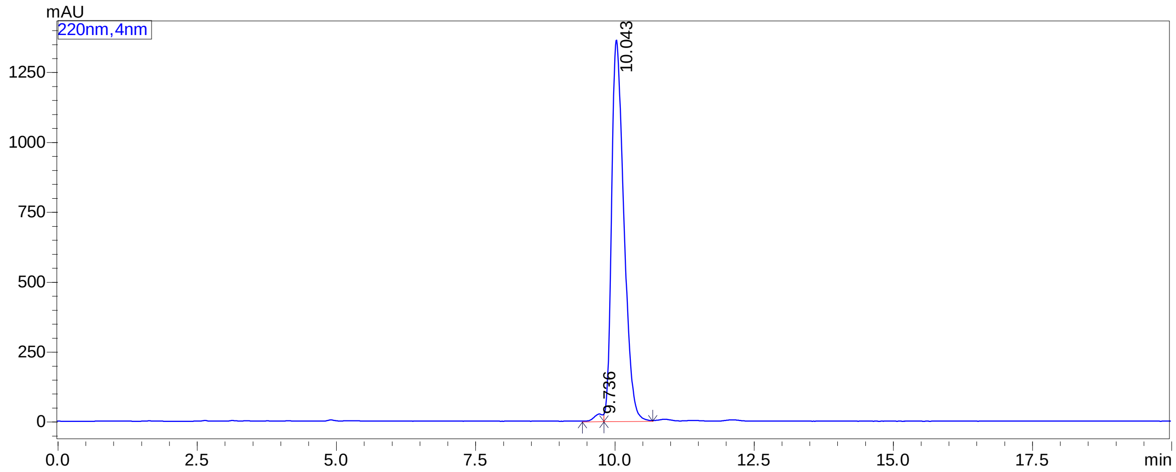

Peak#	Ret. Time	Area%
1	9.736	1.184
2	10.043	98.816
Total		100.000

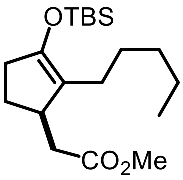

HPLC data of *rac*-**3c**

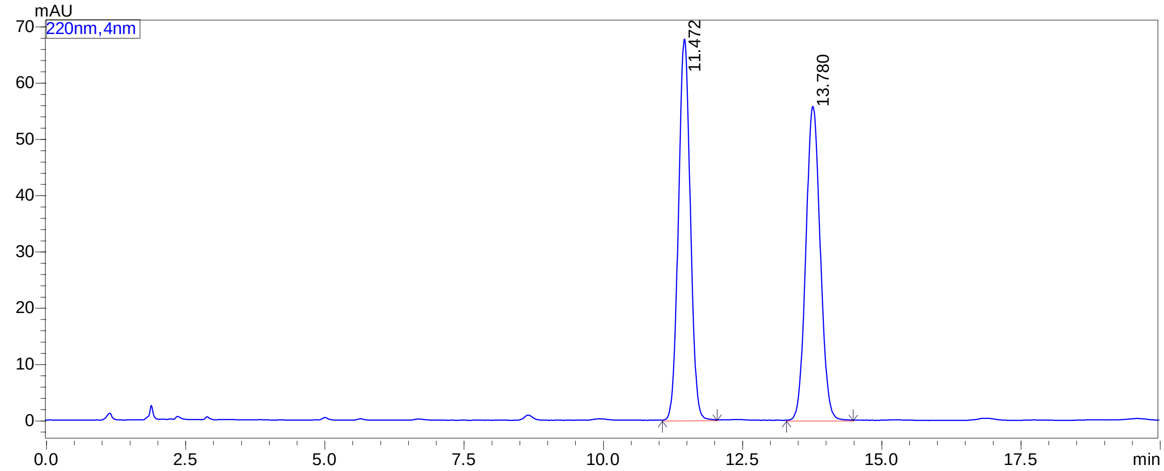

Peak#	Ret. Time	Area%
1	11.472	49.958
2	13.780	50.042
Total		100.000

HPLC data of (*R*)-**3c**

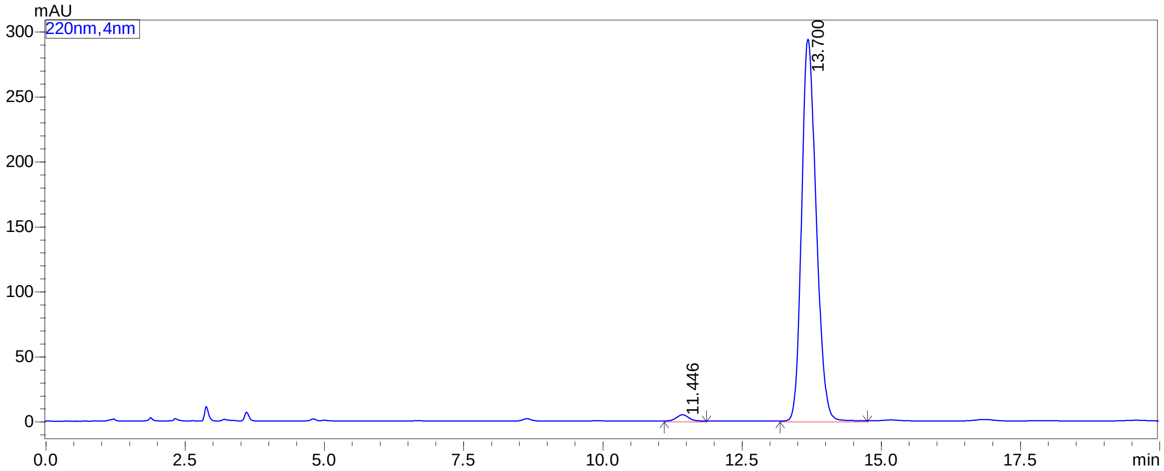

Peak#	Ret. Time	Area%
1	11.446	1.286
2	13.700	98.714
Total		100.000

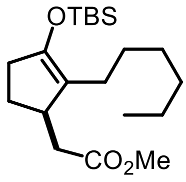

HPLC data of *rac*-**3d**

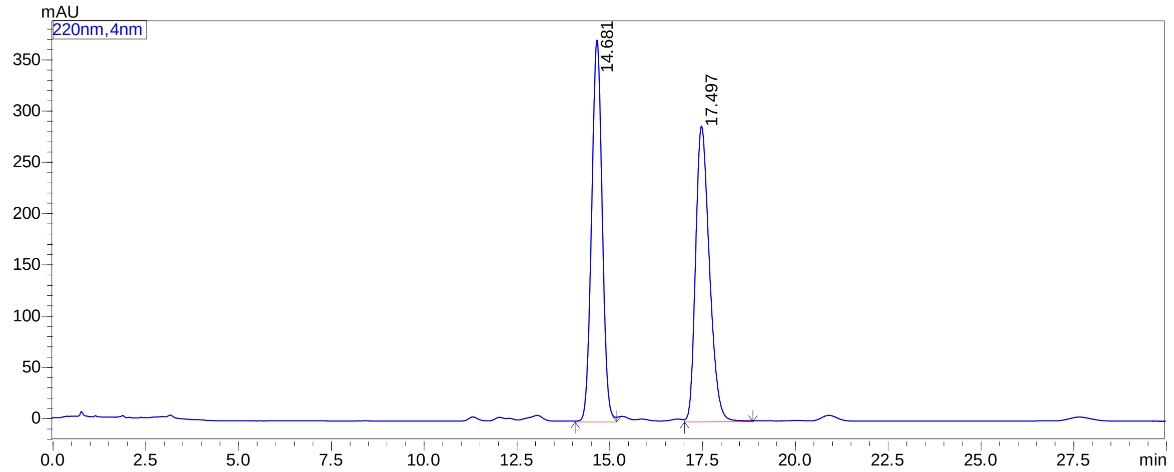

Peak#	Ret. Time	Area%
1	14.681	49.907
2	17.497	50.093
Total		100.000

HPLC data of (*R*)-**3d**

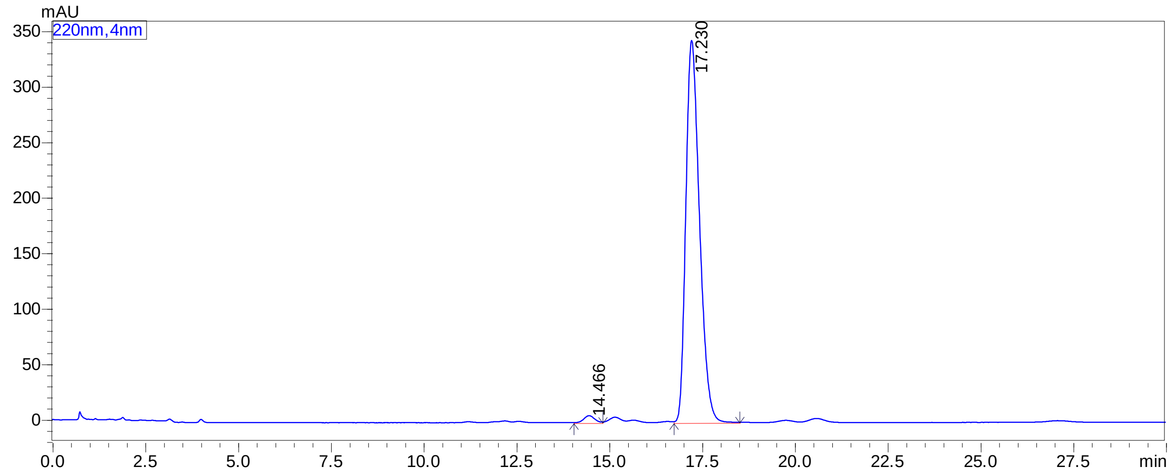

Peak#	Ret. Time	Area%
1	14.466	1.362
2	17.230	98.638
Total		100.000

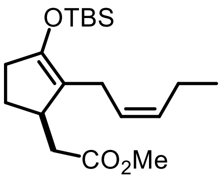

HPLC data of *rac*-**3e**

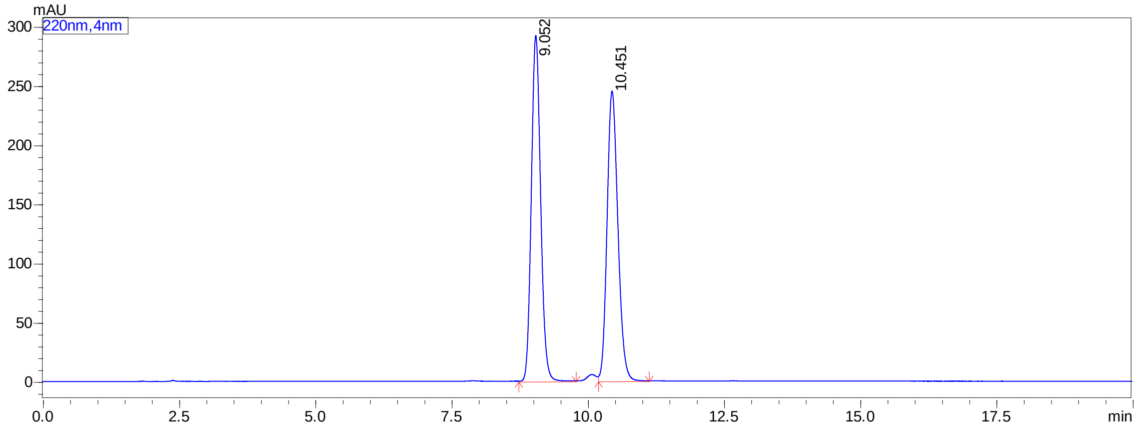

Peak	Ret. Time	Area%
1	9.052	50.471
2	10.451	49.529
Total		100.000

HPLC data of (*R*)-**3e**

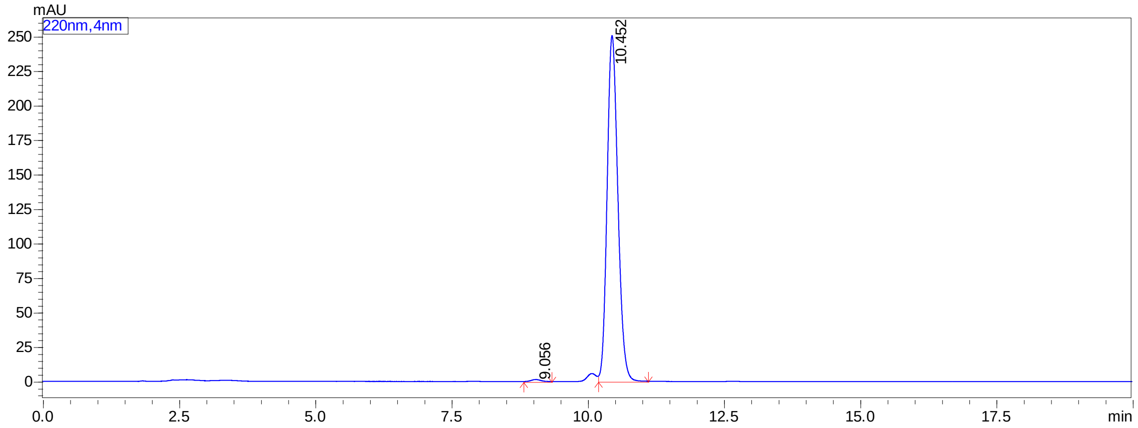

Peak	Ret. Time	Area%
1	9.056	0.497
2	10.452	99.503
Total		100.000

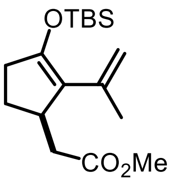

HPLC data of *rac*-**3f**

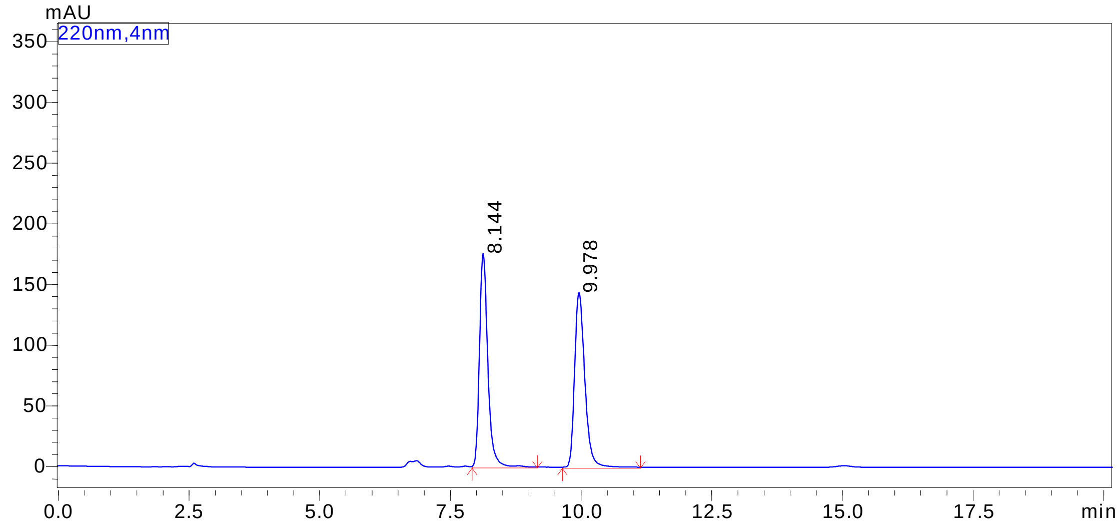

Peak#	Ret. Time	Area%
1	8.144	50.233
2	9.978	49.767
Total		100.000

HPLC data of (*R*)-**3f**

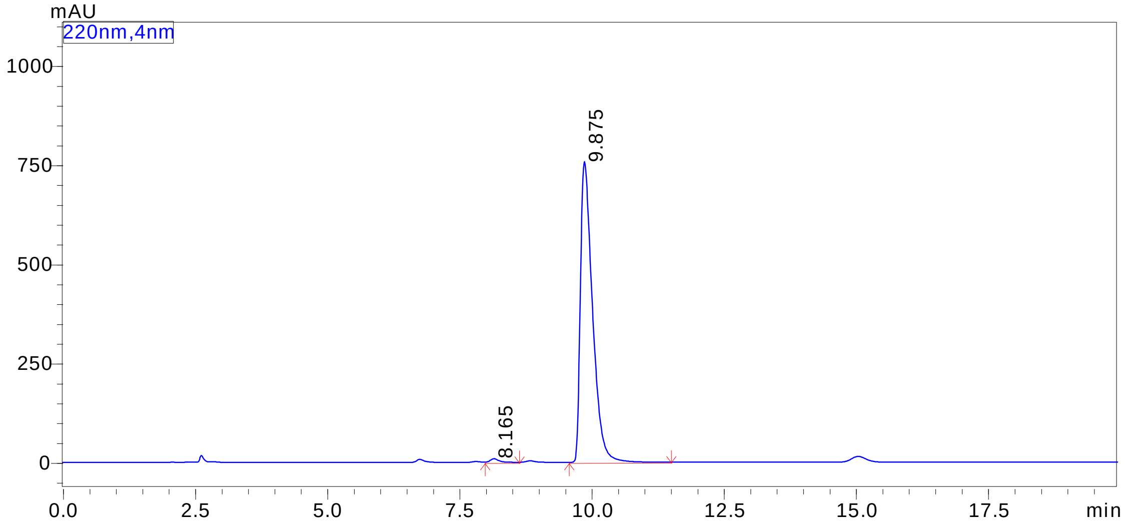

Peak#	Ret. Time	Area%
1	8.165	0.832
2	9.875	99.168
Total		100.000

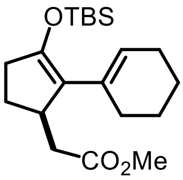

HPLC data of *rac*-**3g**

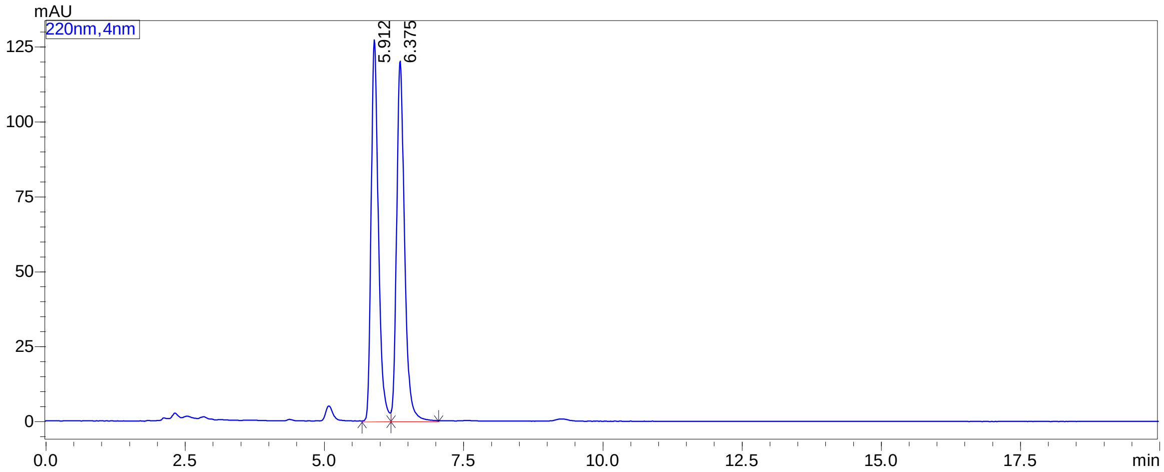

Peak#	Ret. Time	Area%
1	5.912	49.234
2	6.375	50.766
Total		100.000

HPLC data of (*R*)-**3g**

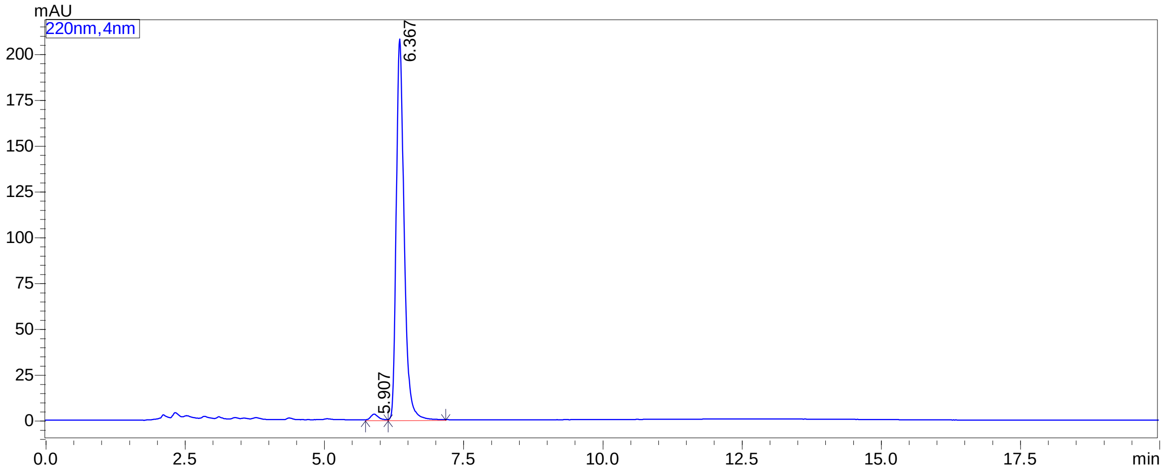

Peak#	Ret. Time	Area%
1	5.907	1.302
2	6.367	98.698
Total		100.000

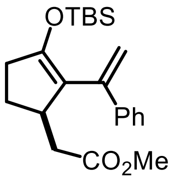

HPLC data of *rac*-**3h**

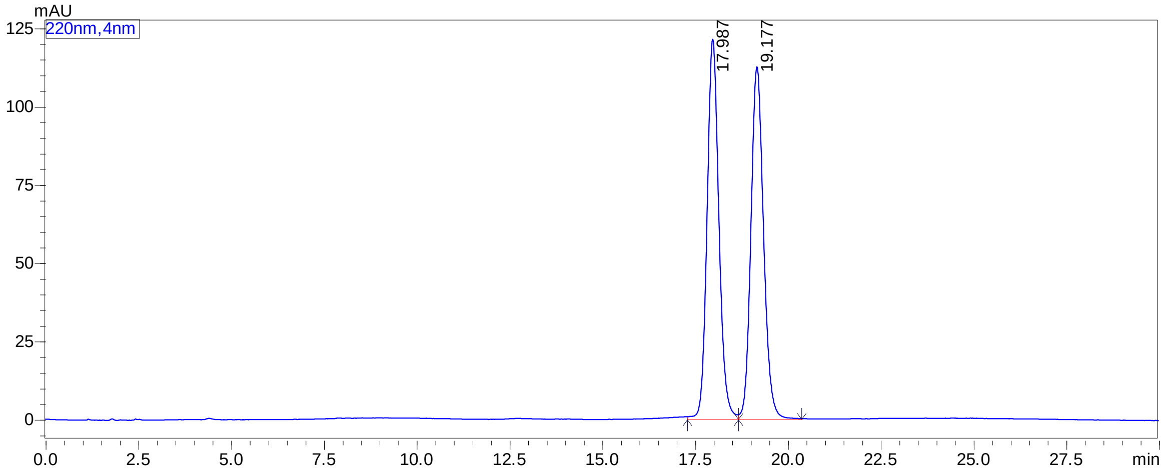

Peak#	Ret. Time	Area%
1	17.987	50.232
2	19.177	49.768
Total		100.000

HPLC data of (*R*)-**3h**

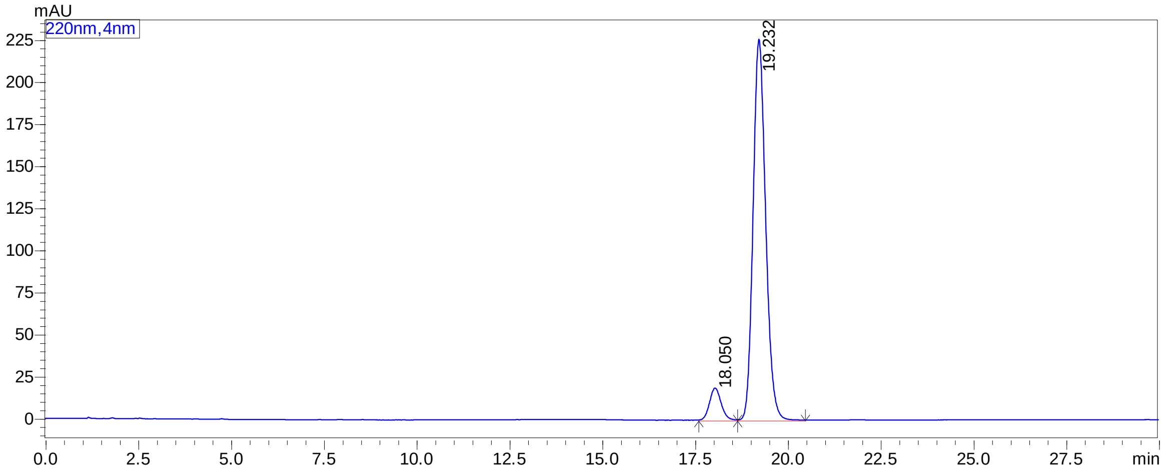

Peak#	Ret. Time	Area%
1	18.050	7.158
2	19.232	92.842
Total		100.000

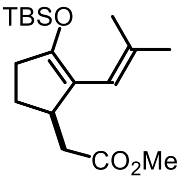

HPLC data of *rac*-**3i**

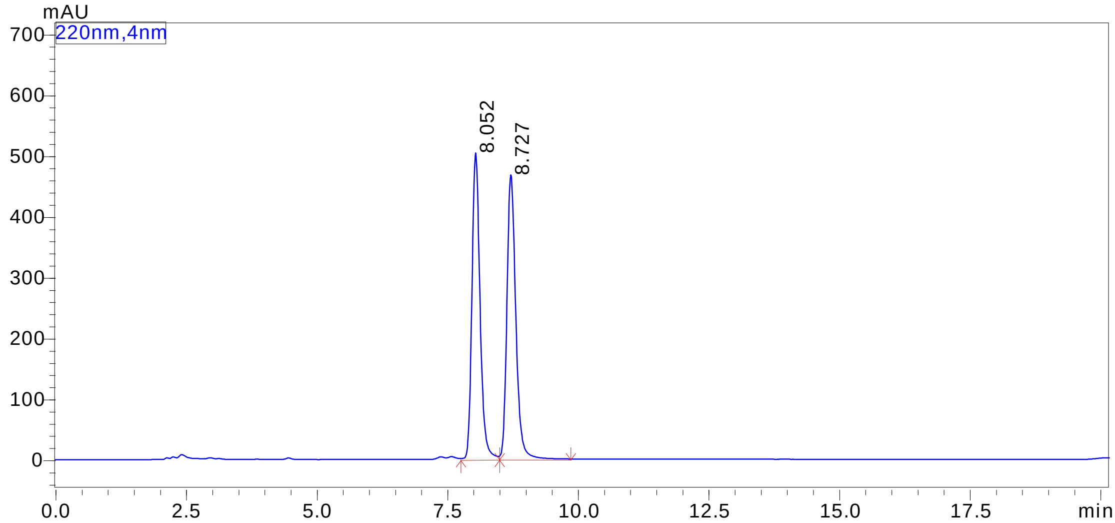

Peak#	Ret. Time	Area%
1	8.052	49.531
2	8.727	50.469
Total		100.000

HPLC data of (*R*)-**3i**

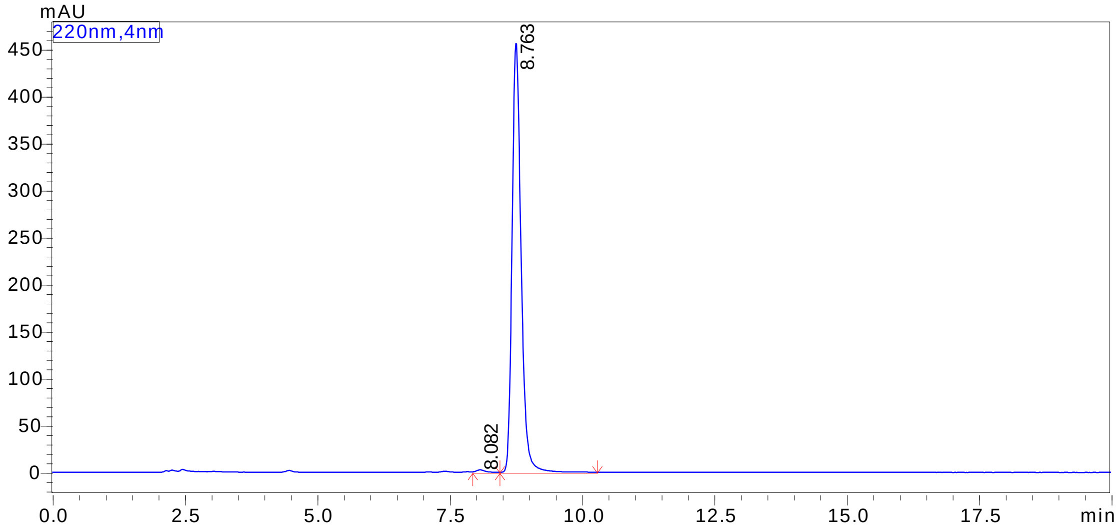

Peak#	Ret. Time	Area%
1	8.082	0.509
2	8.763	99.491
Total		100.000

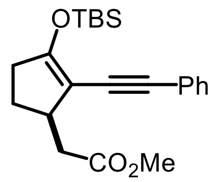

HPLC data of *rac*-**3j**

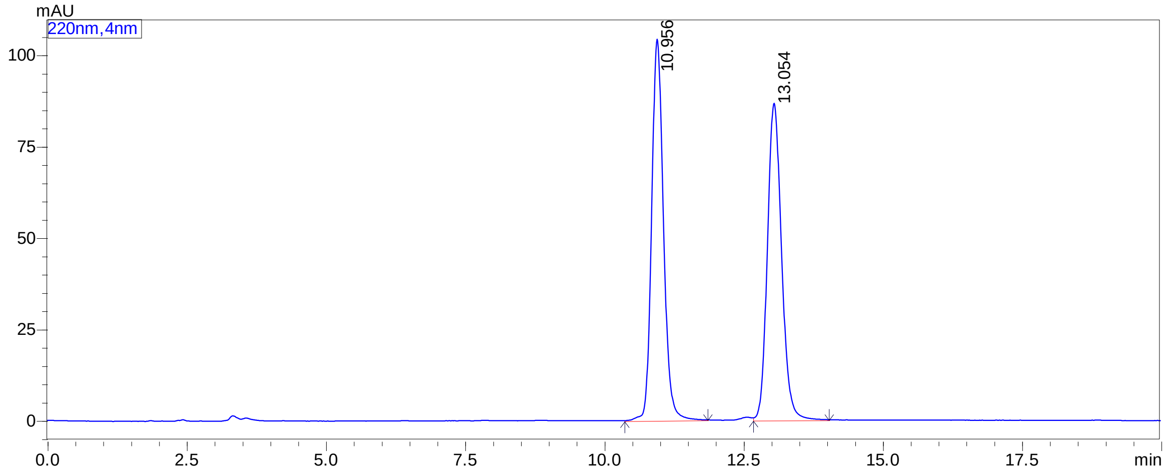

Peak#	Ret. Time	Area%
1	10.956	50.149
2	13.054	49.851
Total		100.000

HPLC data of (*R*)-**3j**

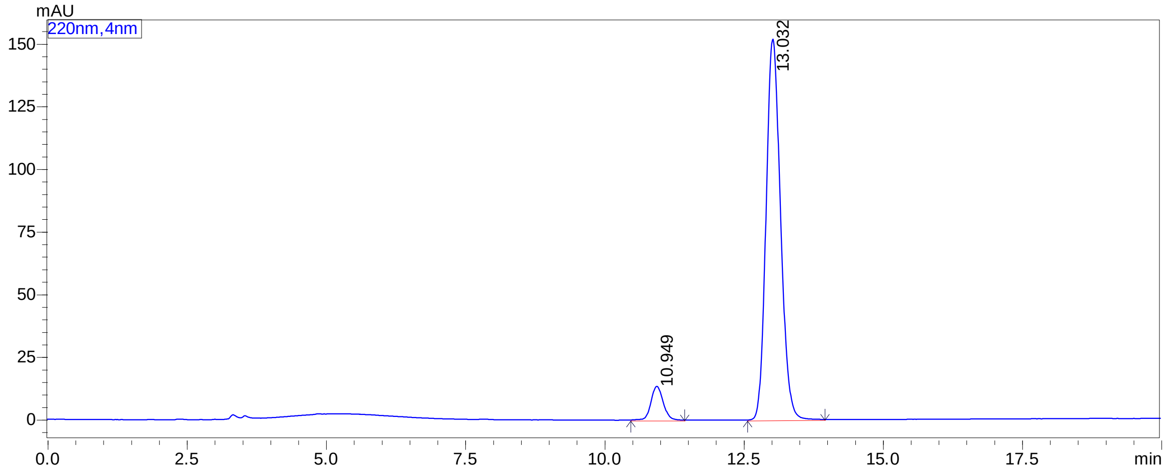

Peak#	Ret. Time	Area%
1	10.949	6.918
2	13.032	93.082
Total		100.000

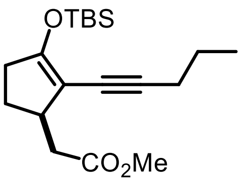

HPLC data of *rac*-**3k**

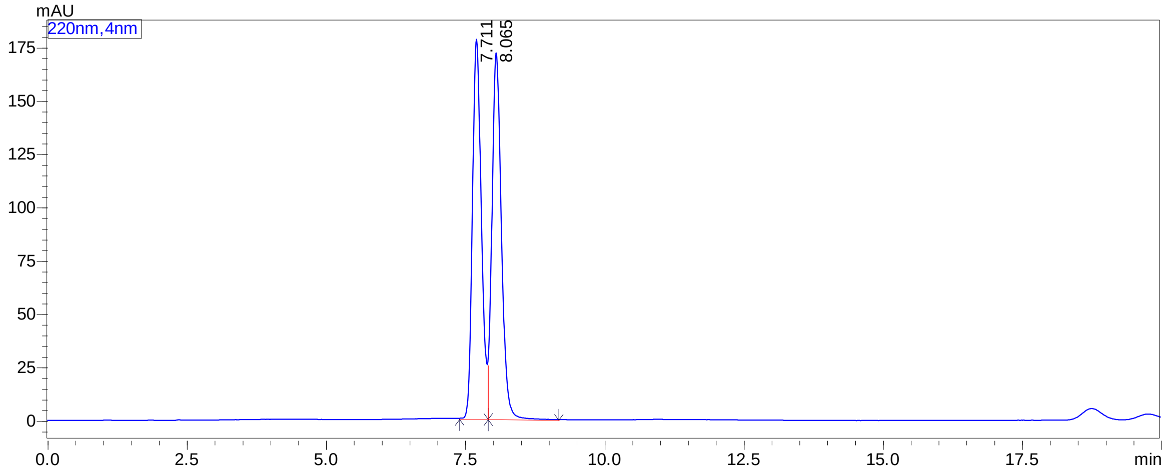

Peak#	Ret. Time	Area%
1	7.711	49.185
2	8.065	50.815
Total		100.000

HPLC data of (*R*)-**3k**

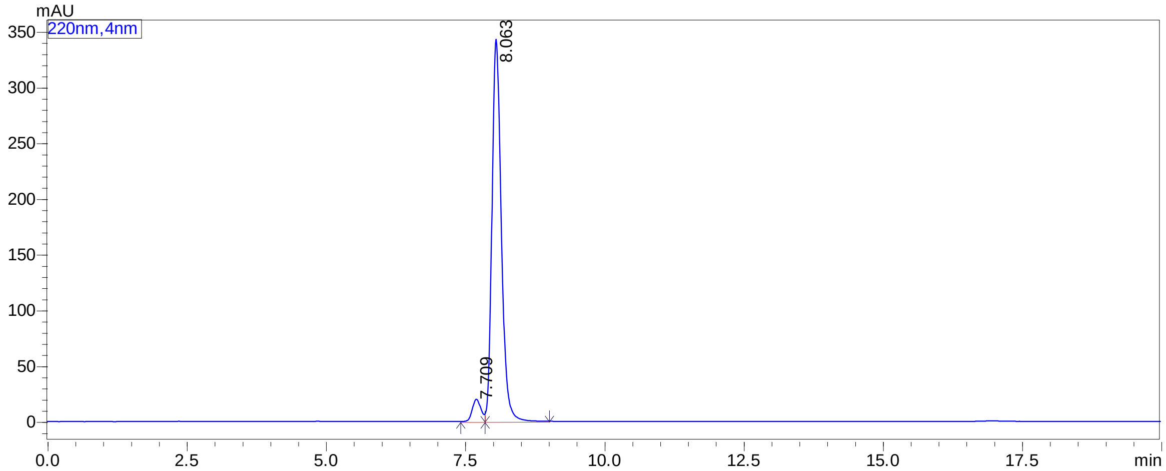

Peak#	Ret. Time	Area%
1	7.709	5.020
2	8.063	94.980
Total		100.000

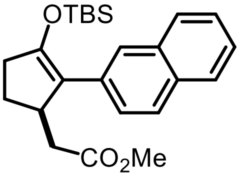

HPLC data of *rac*-**3l**

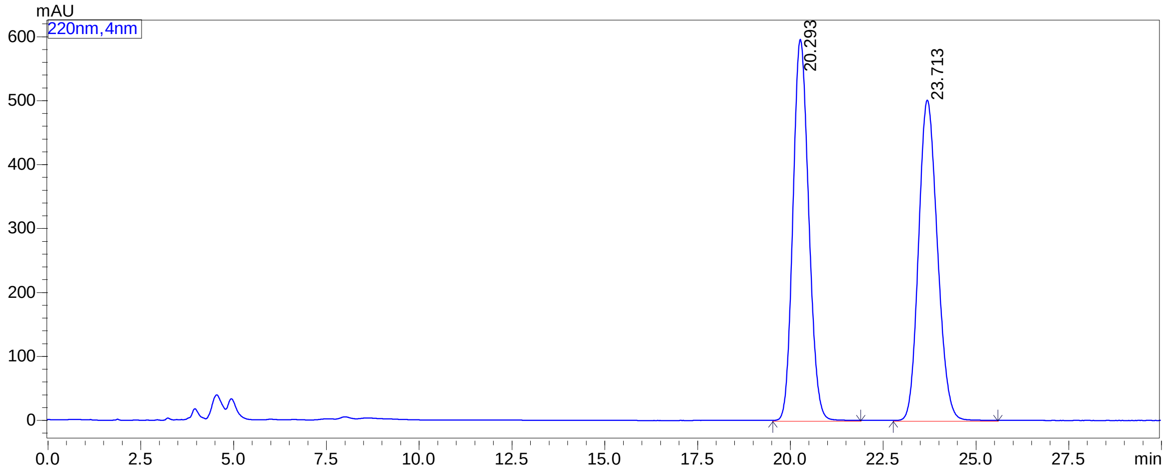

Peak#	Ret. Time	Area%
1	20.293	49.863
2	23.713	50.137
Total		100.000

HPLC data of (*R*)-**3l**

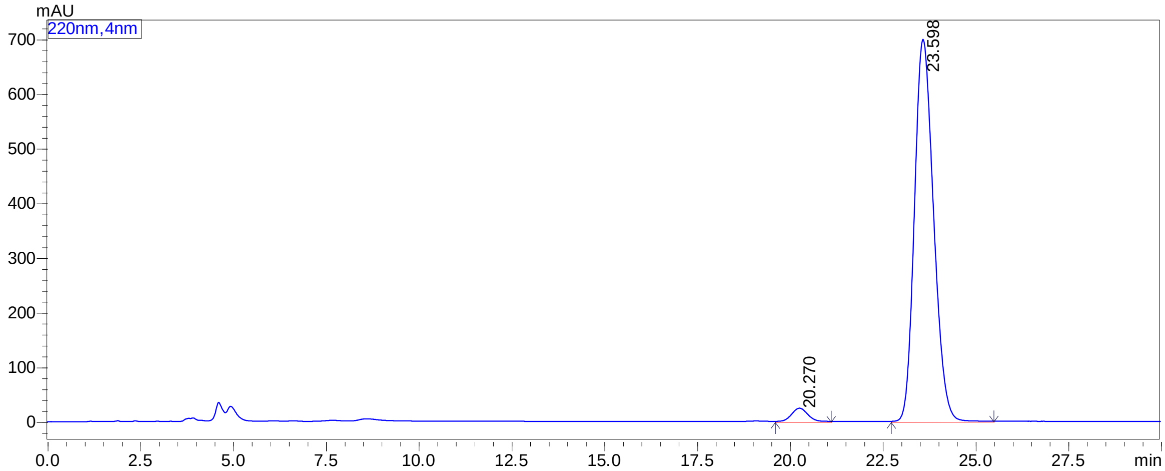

Peak#	Ret. Time	Area%
1	20.270	2.736
2	23.598	97.264
Total		100.000

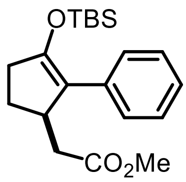

HPLC data of *rac*-**3m**

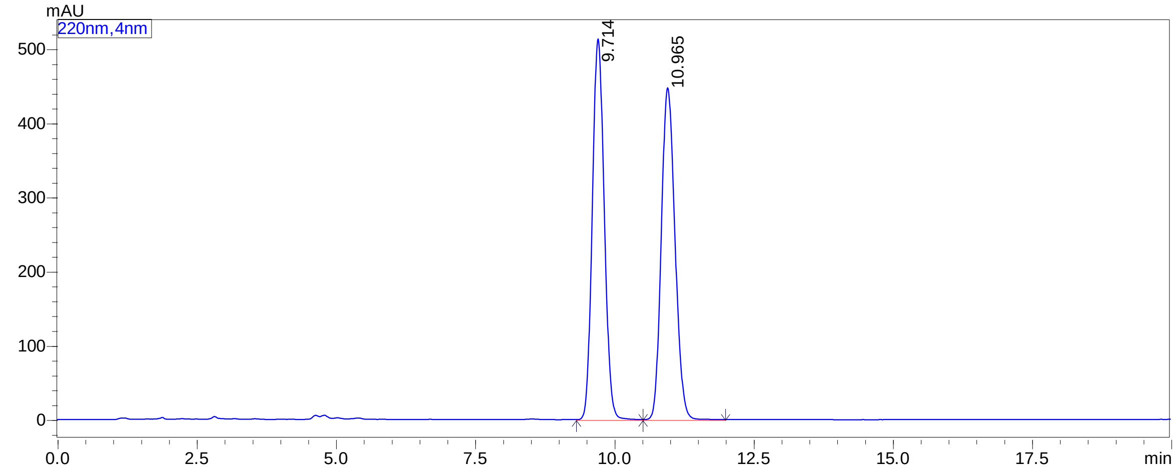

Peak#	Ret. Time	Area%
1	9.714	50.012
2	10.965	49.988
Total		100.000

HPLC data of (*R*)-**3m**

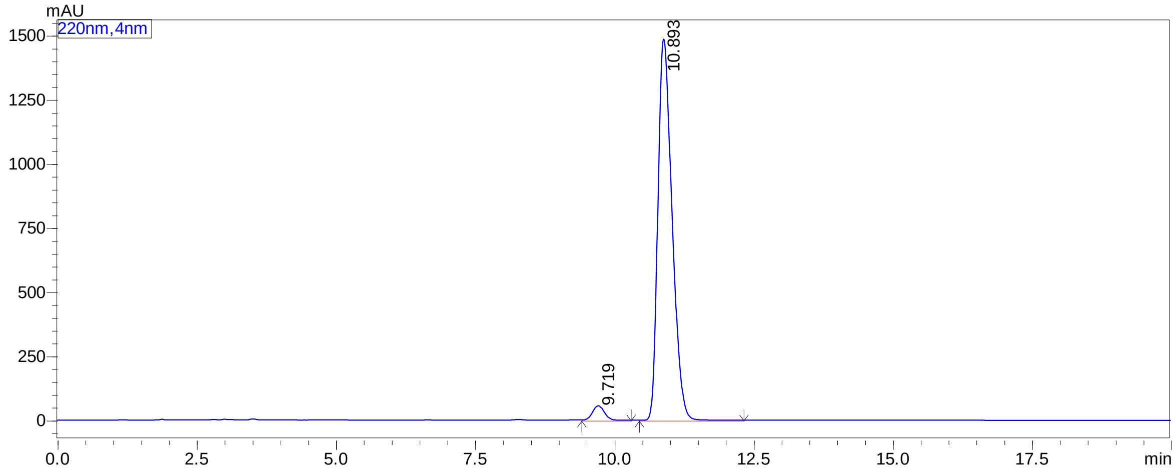

Peak#	Ret. Time	Area%
1	9.719	3.062
2	10.893	96.938
Total		100.000

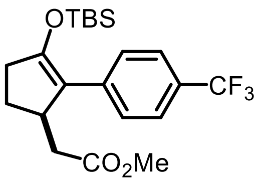

HPLC data of *rac*-**3n**

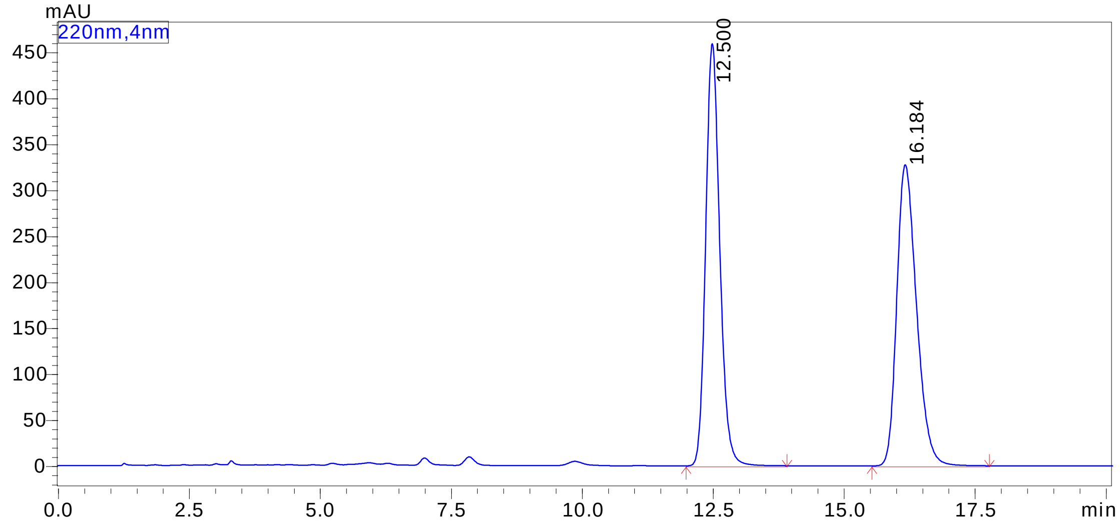

Peak#	Ret. Time	Area%
1	12.500	49.987
2	16.184	50.013
Total		100.000

HPLC data of (*R*)-**3n**

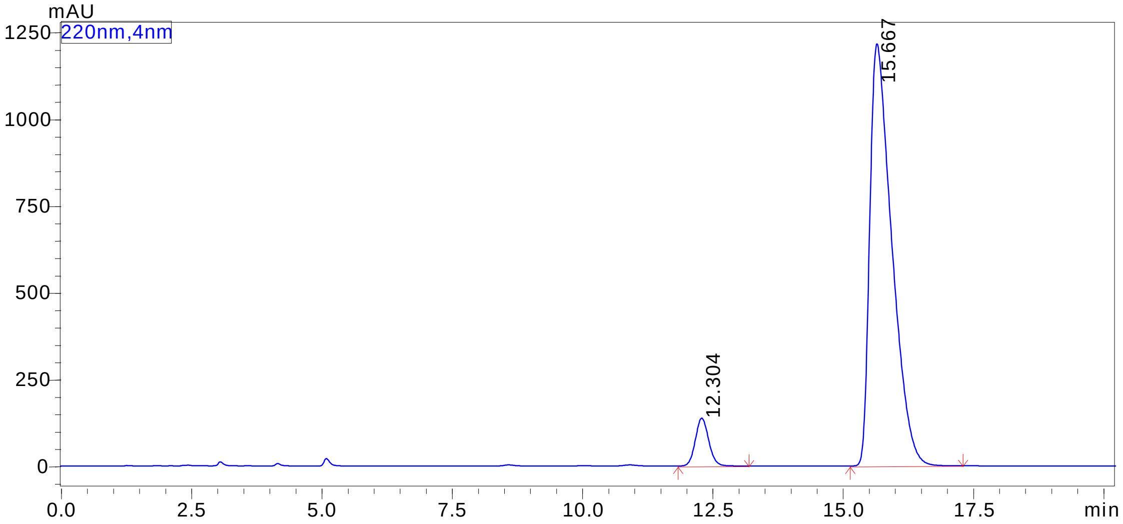

Peak#	Ret. Time	Area%
1	12.304	6.407
2	15.667	93.593
Total		100.000

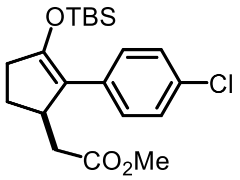

HPLC data of *rac*-**3o**

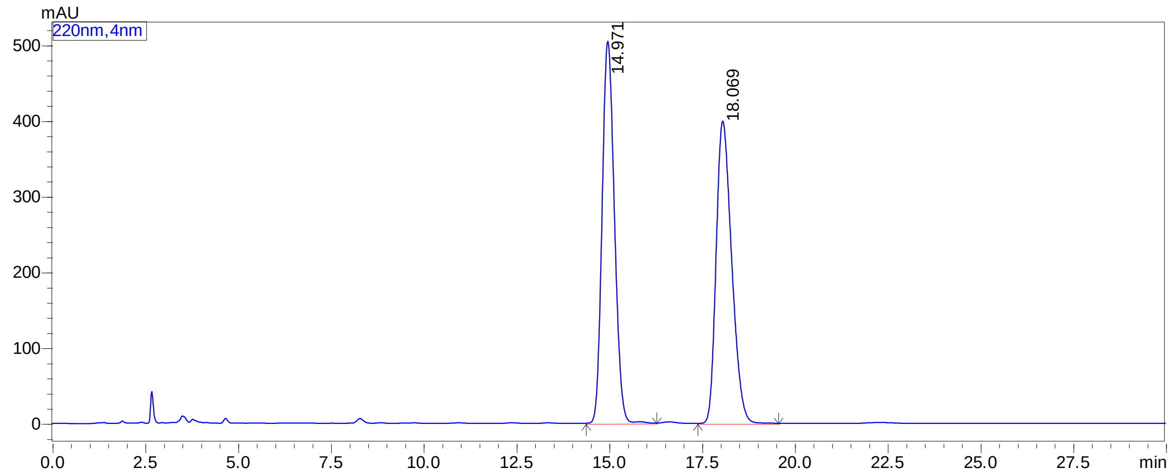

Peak#	Ret. Time	Area%
1	14.971	50.041
2	18.069	49.959
Total		100.000

HPLC data of (*R*)-**3o**

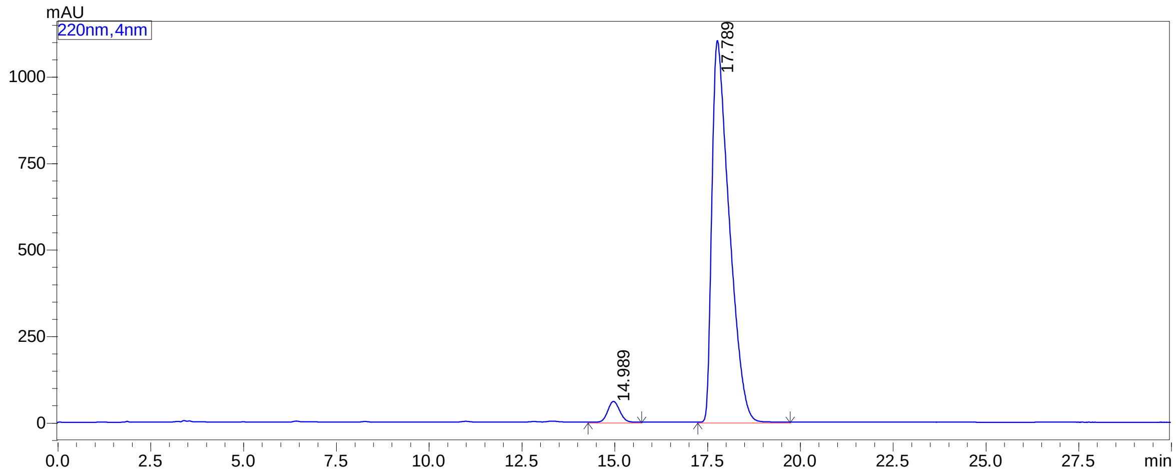

Peak#	Ret. Time	Area%
1	14.989	3.583
2	17.789	96.417
Total		100.000

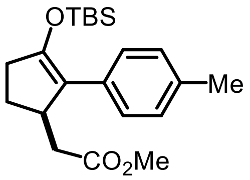

HPLC data of *rac*-**3p**

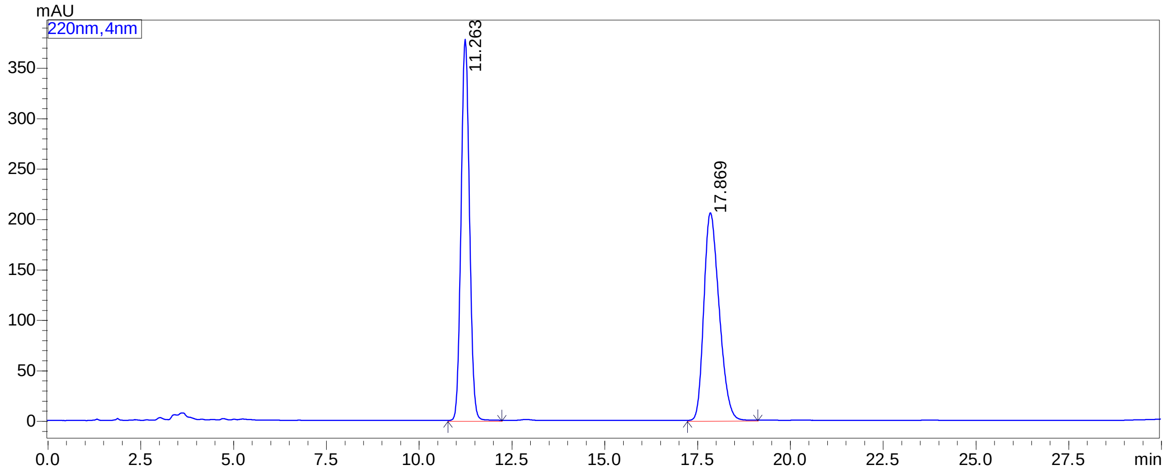

Peak#	Ret. Time	Area%
1	11.263	50.139
2	17.869	49.861
Total		100.000

HPLC data of (*R*)-**3p**

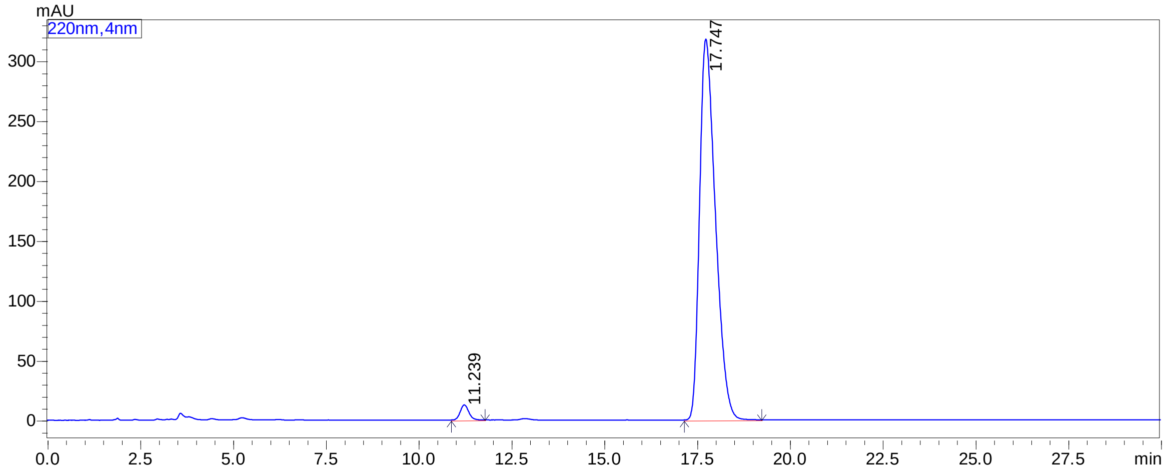

Peak#	Ret. Time	Area%
1	11.239	2.068
2	17.747	97.932
Total		100.000

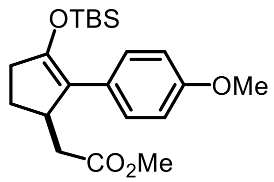

HPLC data of *rac*-**3q**

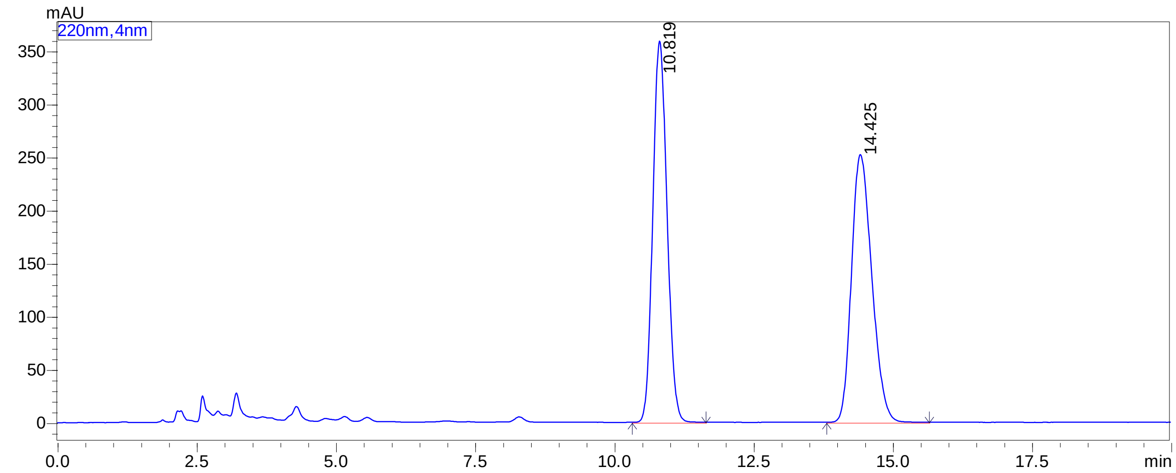

Peak#	Ret. Time	Area%
1	10.819	49.976
2	14.425	50.024
Total		100.000

HPLC data of (*R*)-**3q**

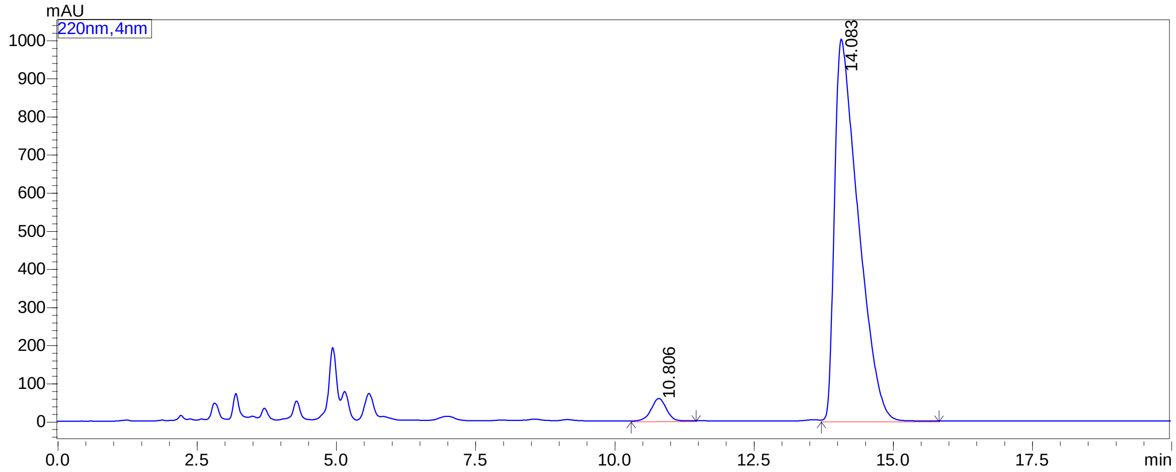

Peak#	Ret. Time	Area%
1	10.806	3.557
2	14.083	96.443
Total		100.000

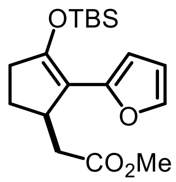

HPLC data of *rac*-**3r**

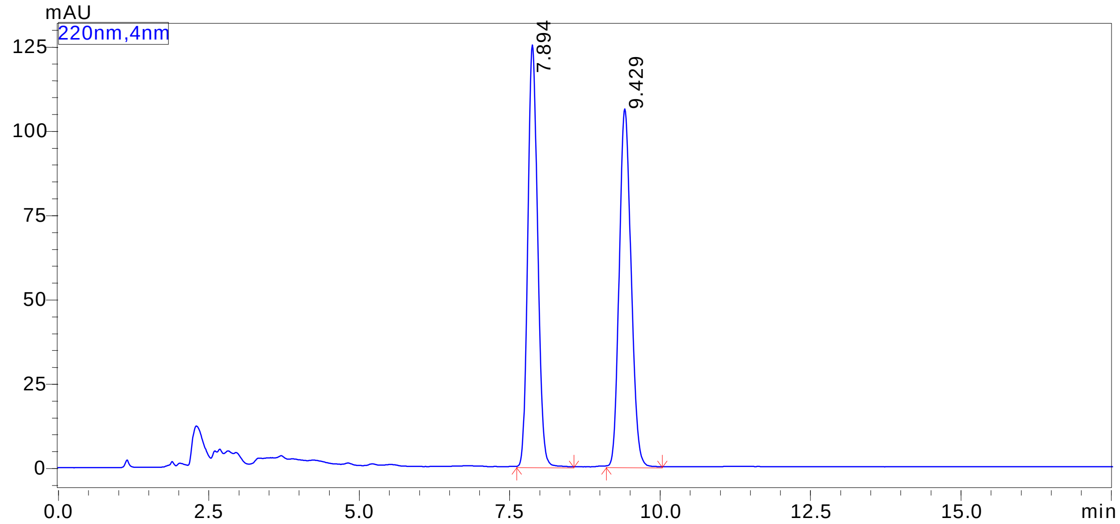

Peak#	Ret. Time	Area%
1	7.894	49.492
2	9.429	50.508
Total		100.000

HPLC data of (*R*)-**3r**

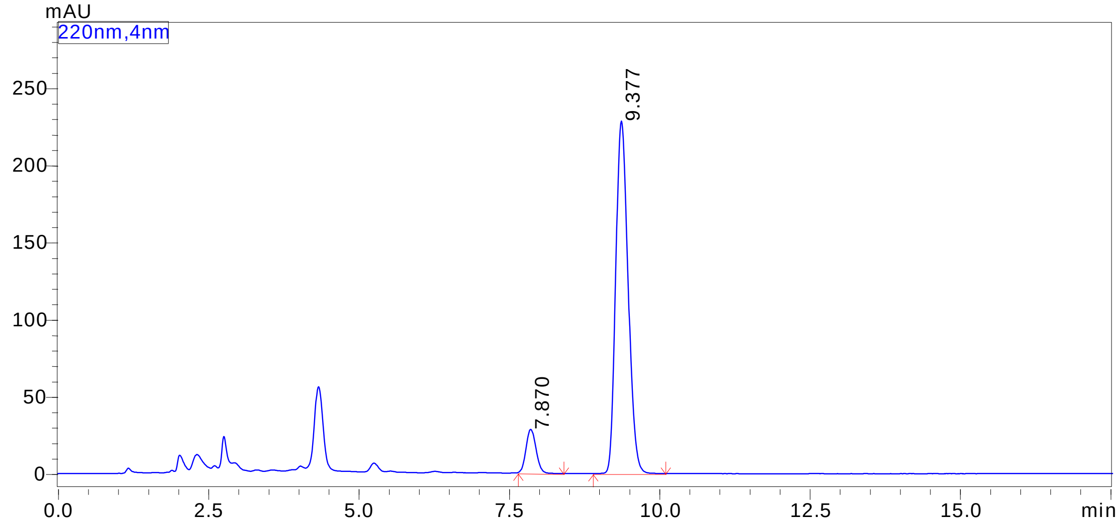

Peak#	Ret. Time	Area%
1	7.870	9.046
2	9.377	90.954
Total		100.000

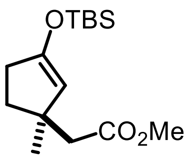

HPLC data of *rac*-**3s**

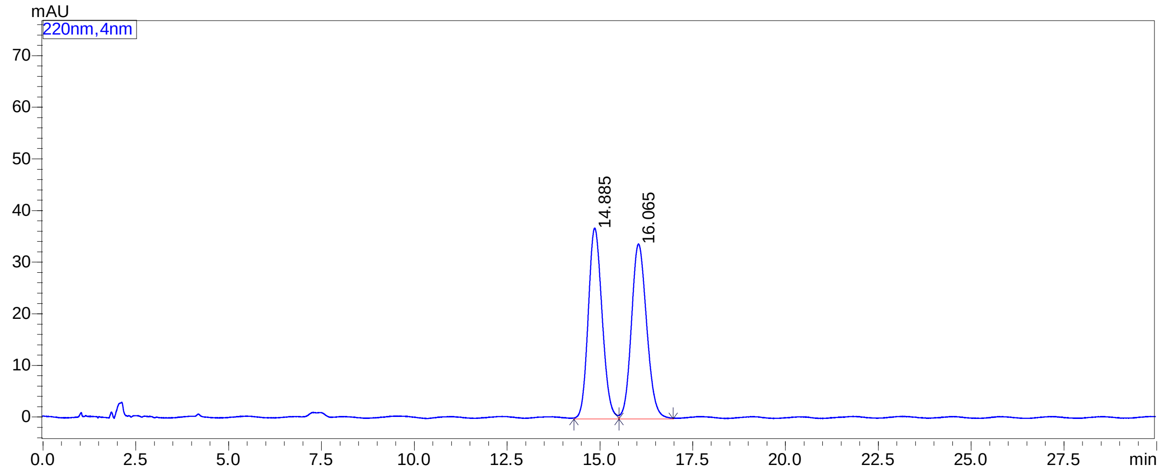

Peak#	Ret. Time	Area%
1	14.885	49.832
2	16.065	50.168
Total		100.000

HPLC data of (*R*)-**3s**

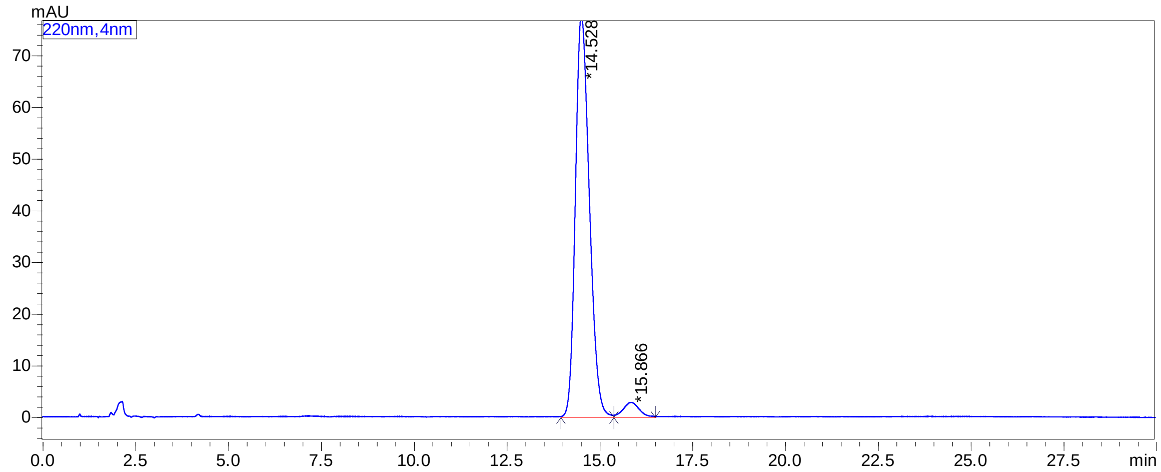

Peak#	Ret. Time	Area%
1	14.528	96.291
2	15.866	3.709
Total		100.000

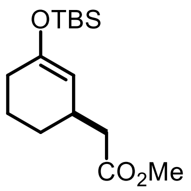

HPLC data of *rac*-**3t**

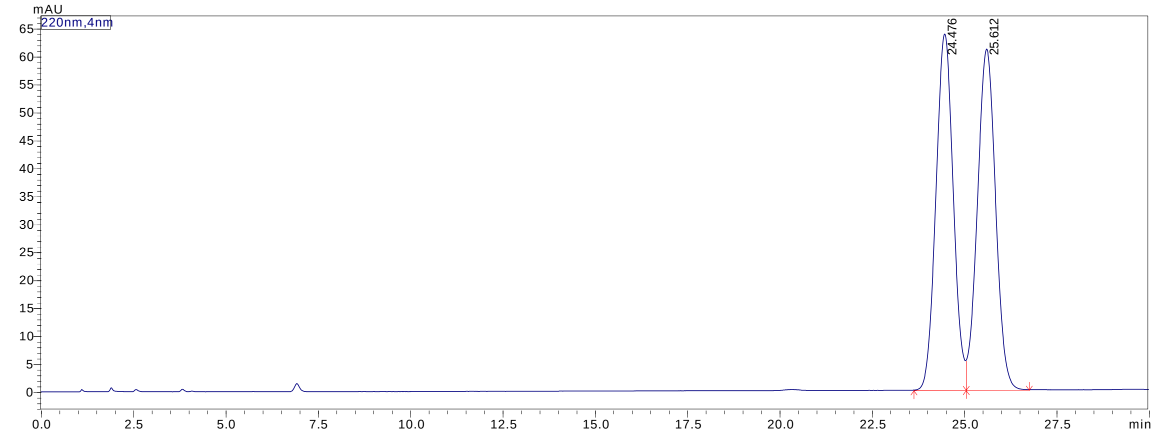

Peak	Ret. Time	Area%
1	24.476	49.827
2	25.612	50.173
Total		100.000

HPLC data of (*R*)-**3t**

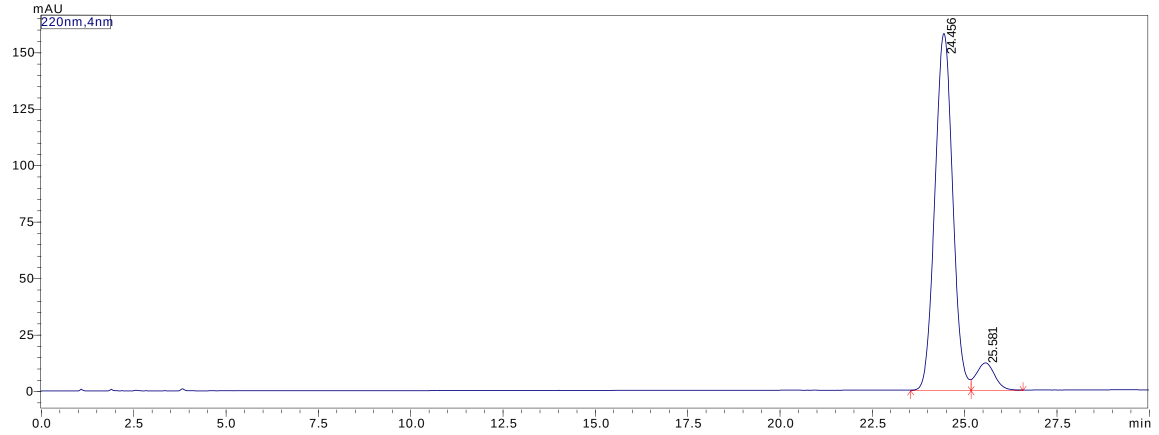

Peak	Ret. Time	Area%
1	24.456	92.736
2	25.581	7.264
Total		100.000

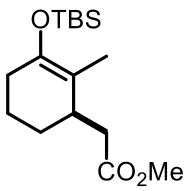

HPLC data of *rac*-**3u**

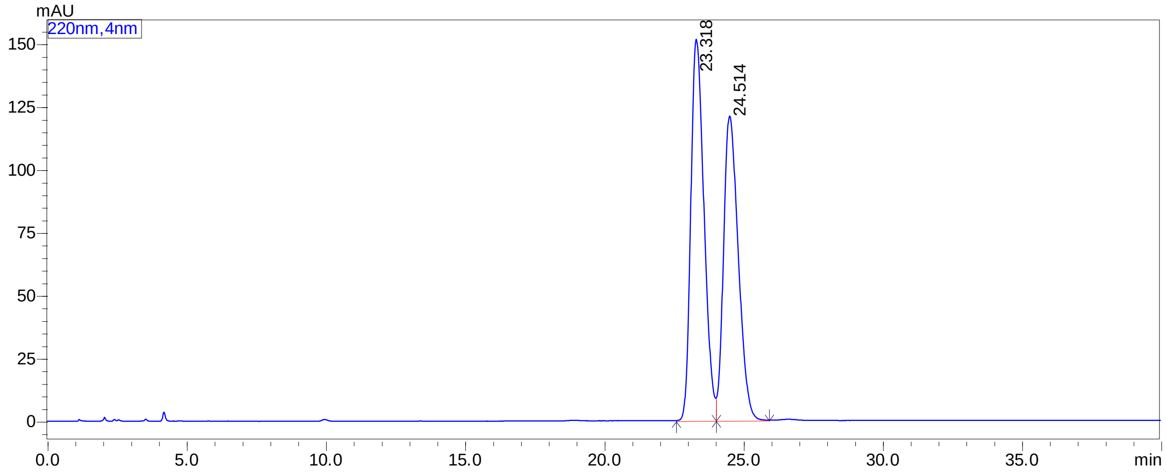

Peak#	Ret. Time	Area%
1	23.318	52.726
2	24.514	47.274
Total		100.000

HPLC data of (*R*)-**3u**

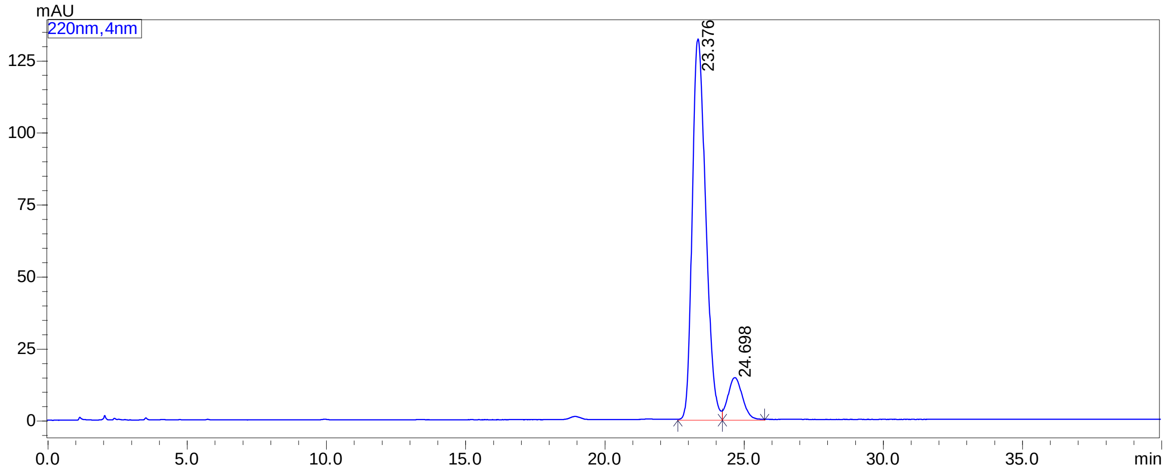

Peak#	Ret. Time	Area%
1	23.376	89.515
2	24.698	10.485
Total		100.000

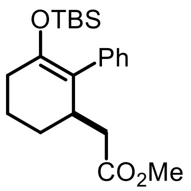

HPLC data of *rac*-**3v**

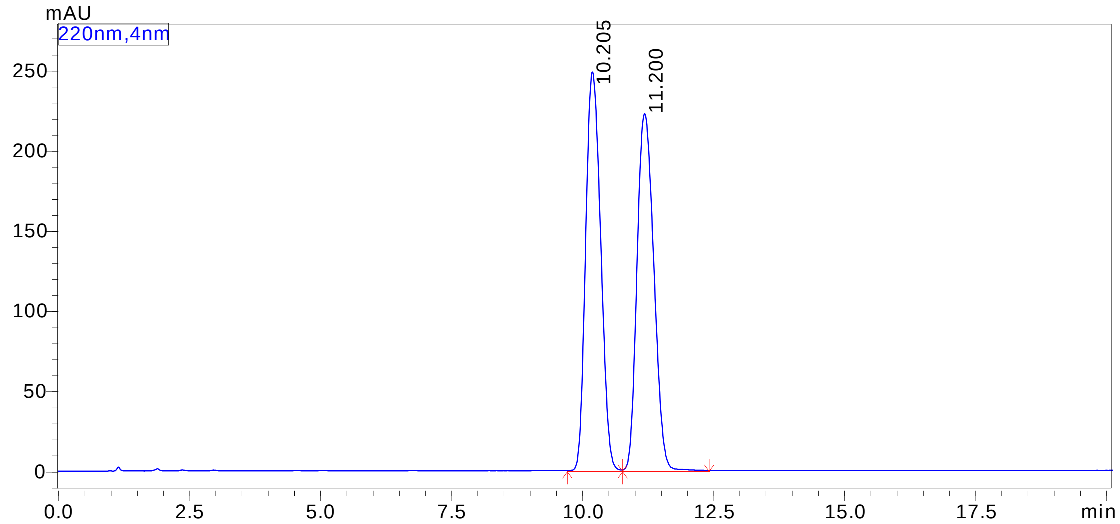

Peak#	Ret. Time	Area%
1	10.205	49.903
2	11.200	50.097
Total		100.000

HPLC data of (*R*)-**3v**

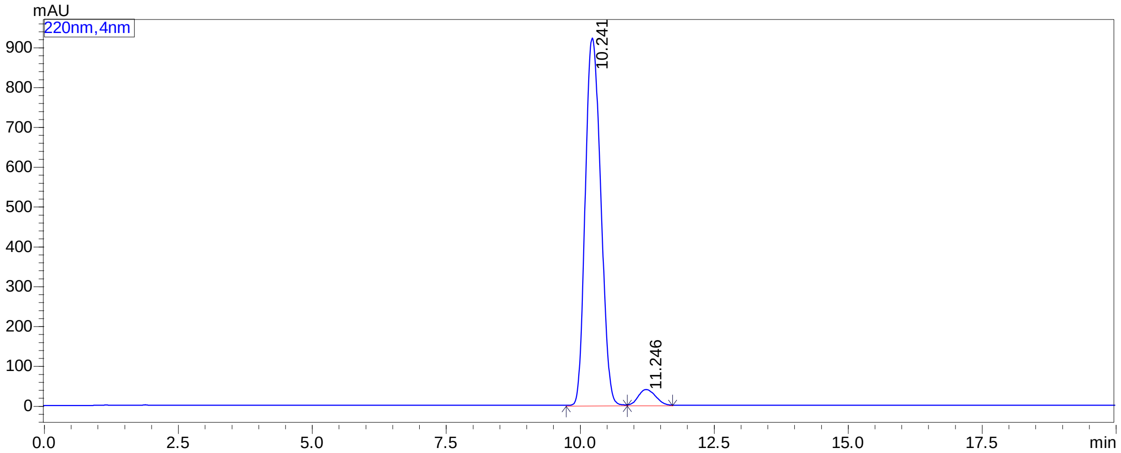

Peak#	Ret. Time	Area%
1	10.241	95.630
2	11.246	4.370
Total		100.000

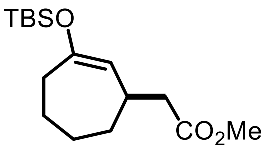

HPLC data of *rac*-**3w**

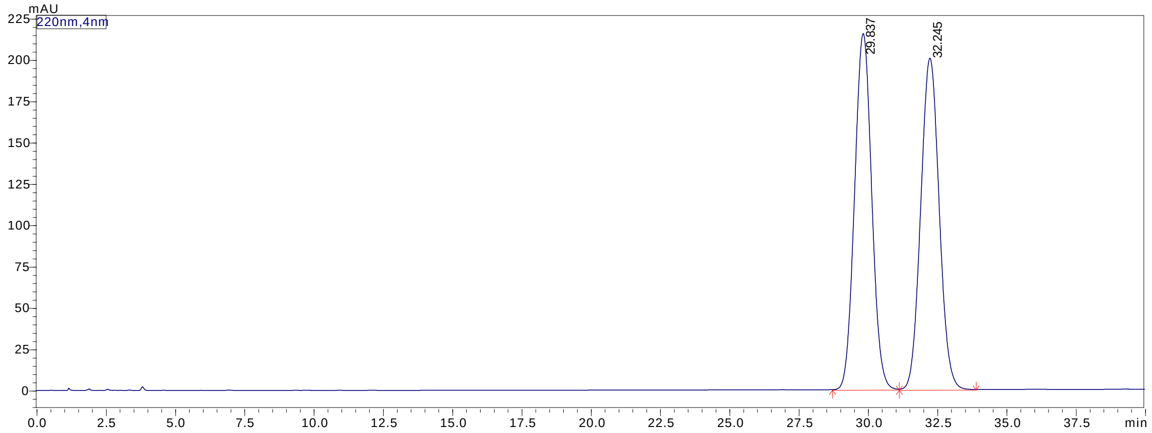

Peak	Ret. Time	Area%
1	29.837	49.955
2	32.245	50.045
Total		100.000

HPLC data of (*R*)-**3w**

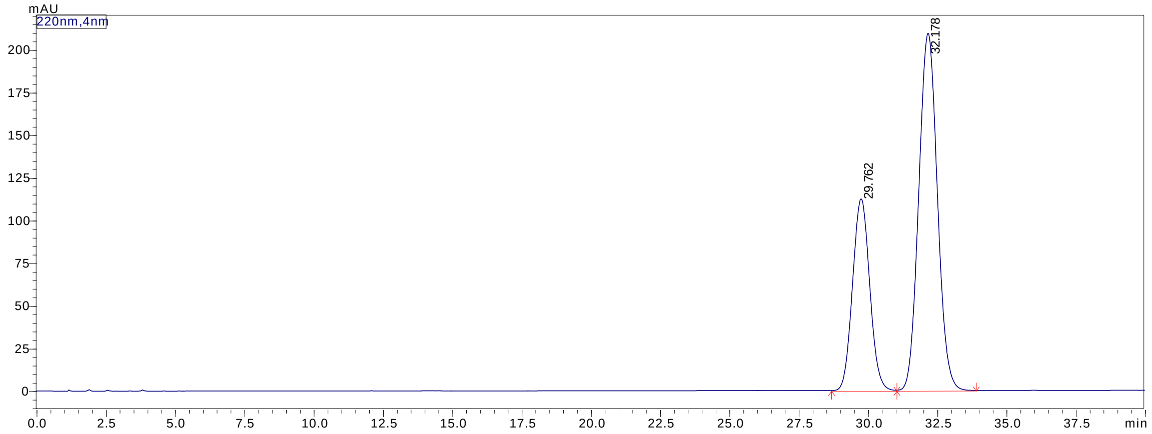

Peak	Ret. Time	Area%
1	29.762	33.102
2	32.178	66.898
Total		100.000

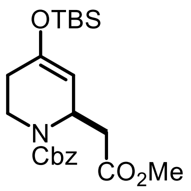

HPLC data of *rac*-**3x**

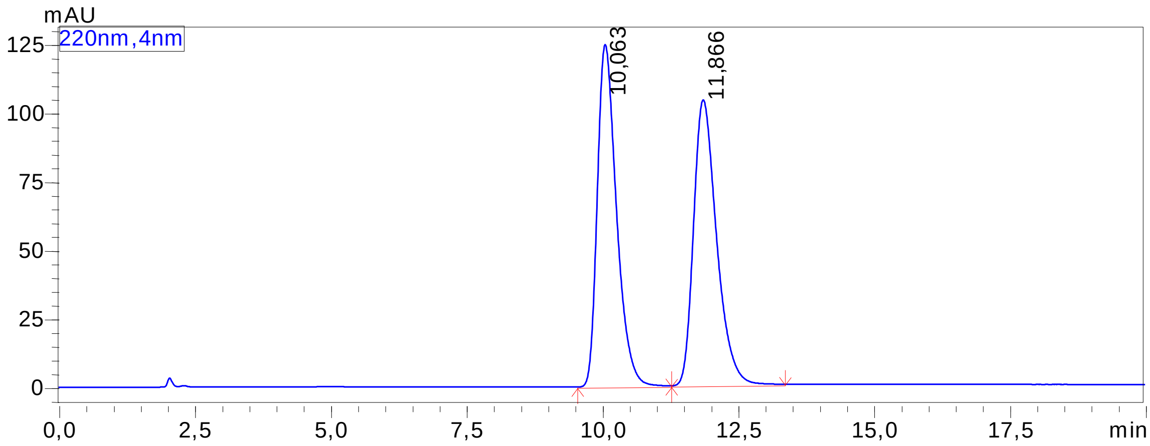

Peak	Ret. Time	Area%
1	10.063	49.965
2	11.866	50.035
Total		100.000

HPLC data of (*R*)-**3x**

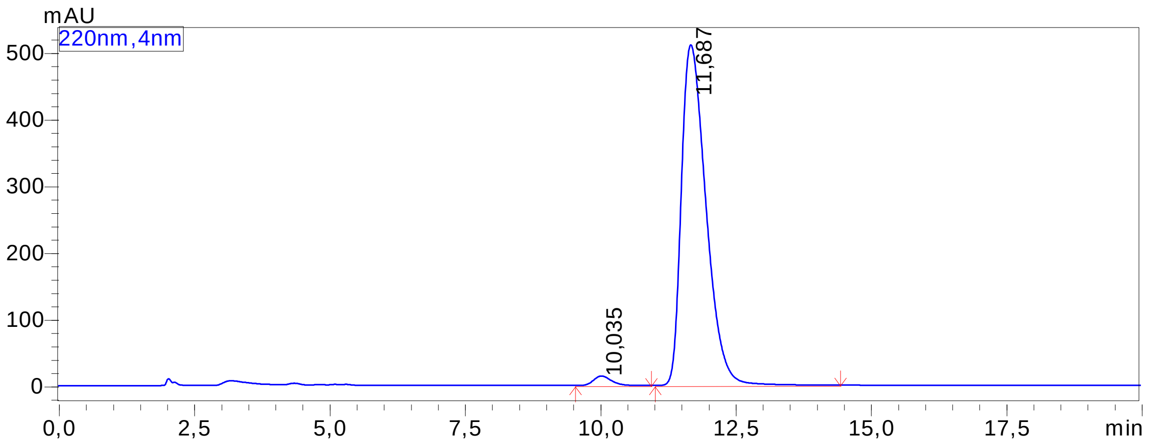

Peak	Ret. Time	Area%
1	10.035	1.980
2	11.687	98.020
Total		100.000

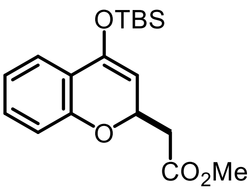

HPLC data of *rac*-**3y**

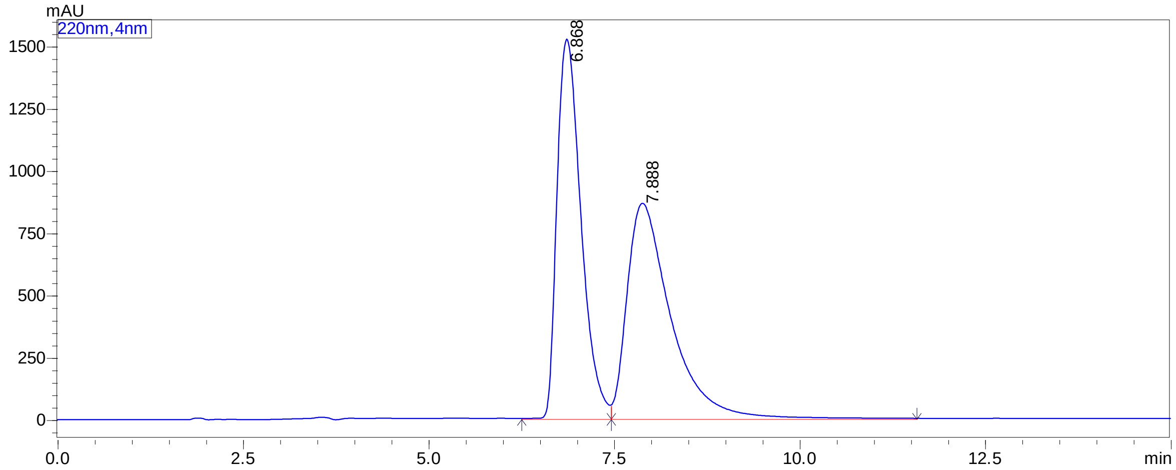

Peak#	Ret. Time	Area%
1	6.868	49.319
2	7.888	50.681
Total		100.000

HPLC data of (*R*)-**3y**

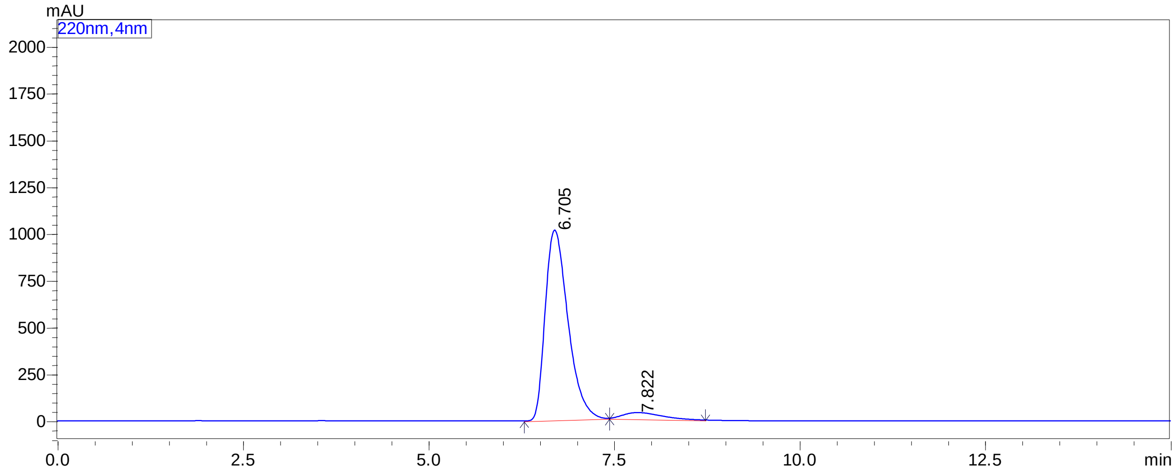

Peak#	Ret. Time	Area%
1	6.705	94.954
2	7.822	5.046
Total		100.000

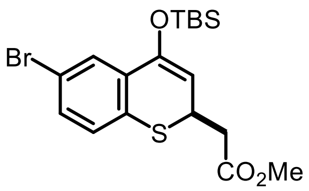

HPLC data of *rac*-**3z**

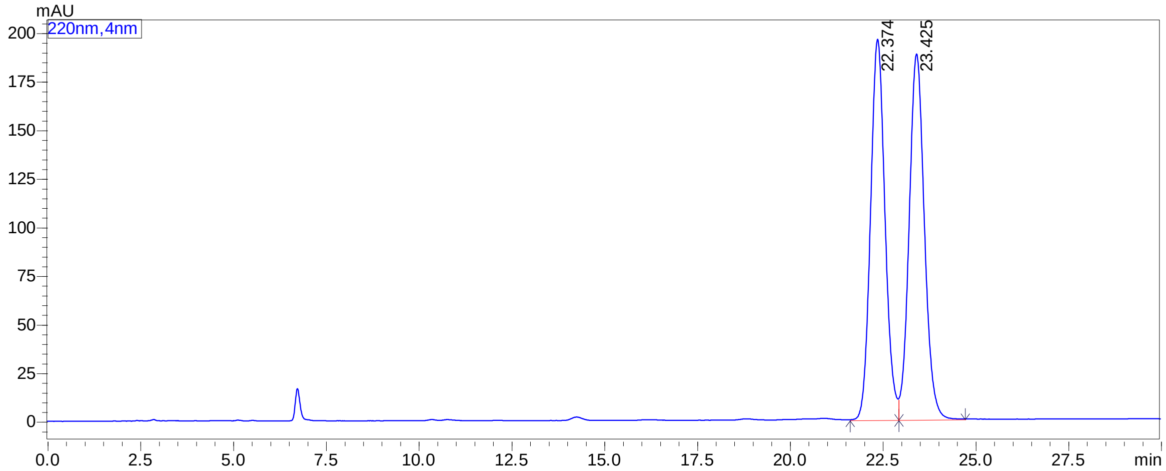

Peak#	Ret. Time	Area%
1	22.374	49.684
2	23.425	50.316
Total		100.000

HPLC data of (*R*)-**3z**

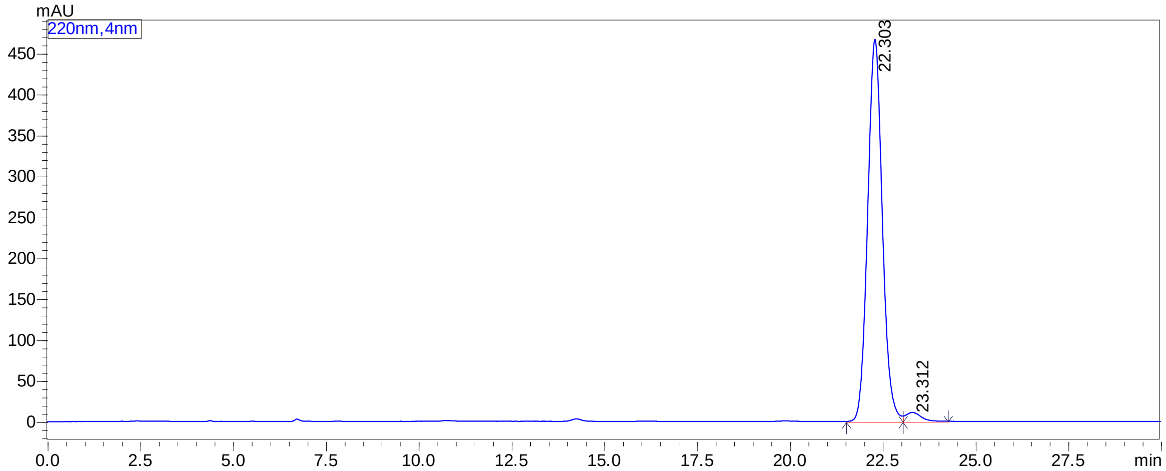

Peak#	Ret. Time	Area%
1	22.303	97.631
2	23.312	2.369
Total		100.000

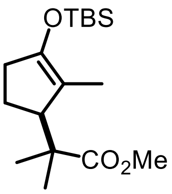

HPLC data of *rac*-**3aa**

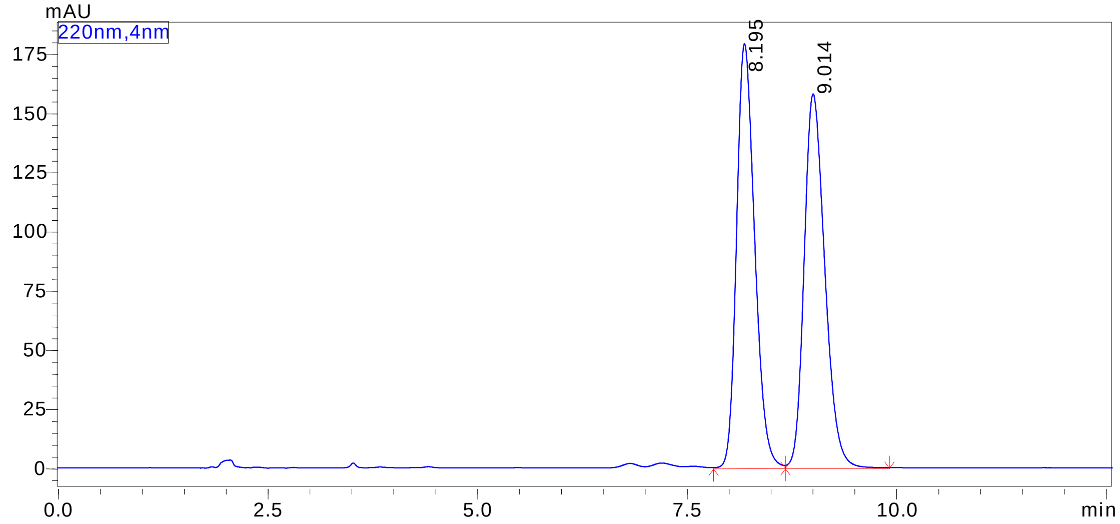

Peak#	Ret. Time	Area%
1	8.195	49.883
2	9.014	50.117
Total		100.000

HPLC data of (*S*)-**3aa**

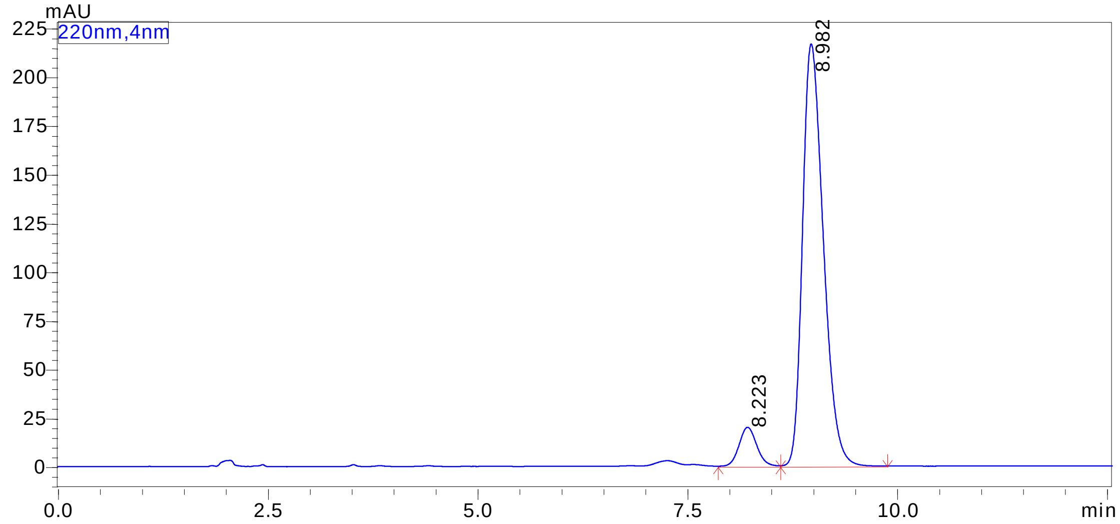

Peak#	Ret. Time	Area%
1	8.223	7.233
2	8.982	92.767
Total		100.000

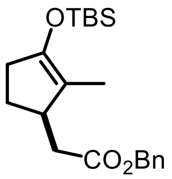

HPLC data of *rac*-**3ab**

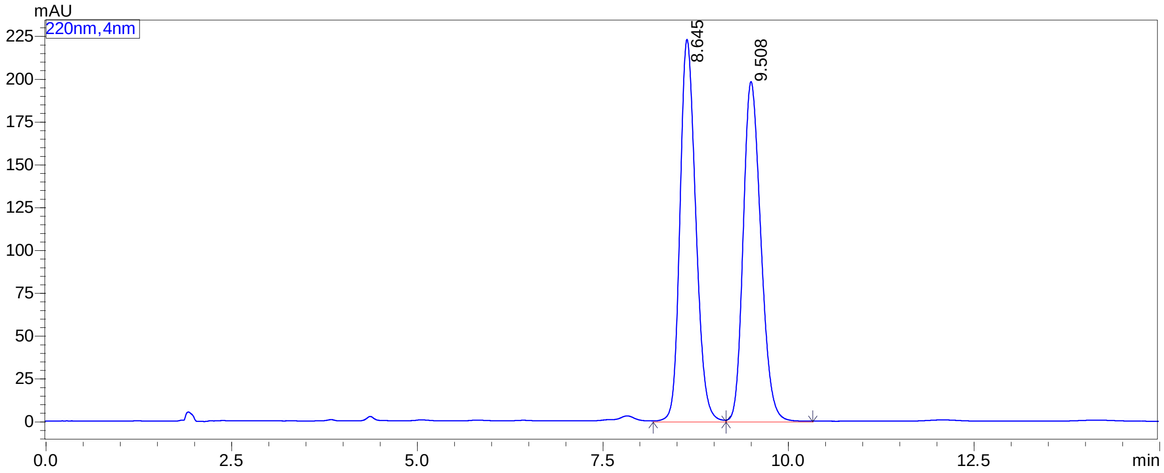

Peak#	Ret. Time	Area%
1	8.645	50.182
2	9.508	49.818
Total		100.000

HPLC data of (*R*)-**3ab**

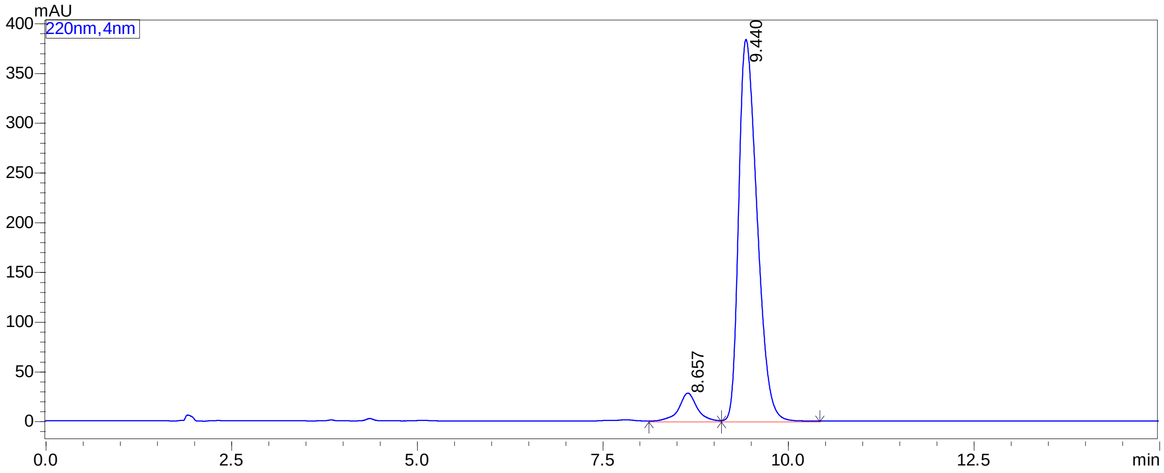

Peak#	Ret. Time	Area%
1	8.657	6.617
2	9.440	93.383
Total		100.000

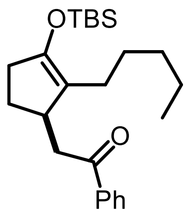

HPLC data of *rac*-**3ac**

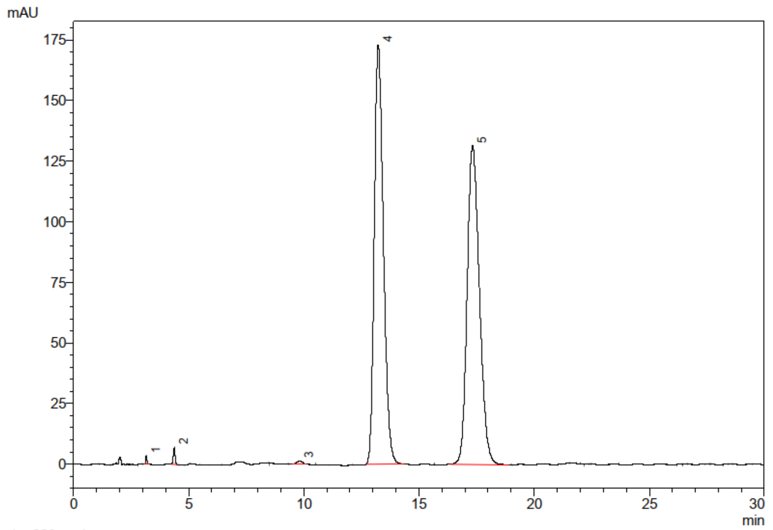

1 220nm,4nm

PDA Ch1 220nm

Peak #	Ret. Time	Area %	Name
1	3.16	0.13	
2	4.37	0.40	
3	9.80	0.27	
4	13.23	49.15	1. enantiomer
5	17.33	50.05	2. enantiomer
Total		100.00	

HPLC data of (*R*)-**3ac**

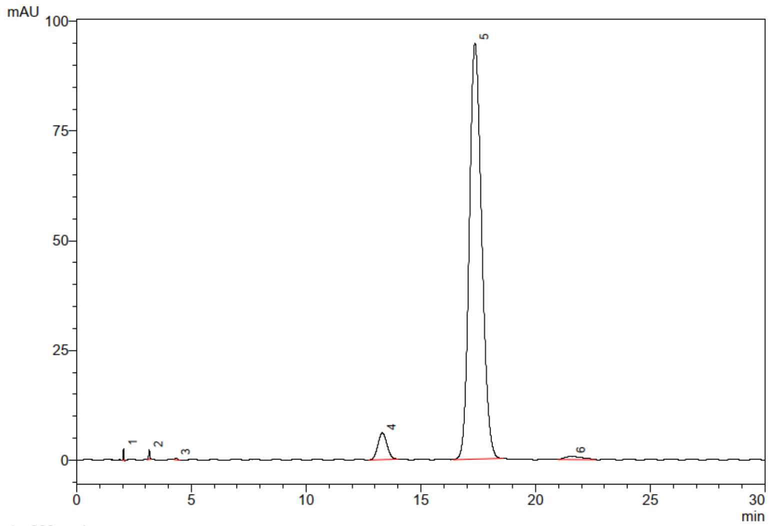

1 220nm,4nm

PDA Ch1 220nm

Peak #	Ret. Time	Area %	Name
1	2.05	0.18	
2	3.17	0.21	
3	4.35	0.06	
4	13.33	4.45	1st enantiomer
5	17.36	94.10	2nd enantiomer
6	21.58	1.00	
Total		100.00	

ee = 90.9 %

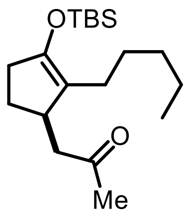

HPLC data of *rac*-**3ad**

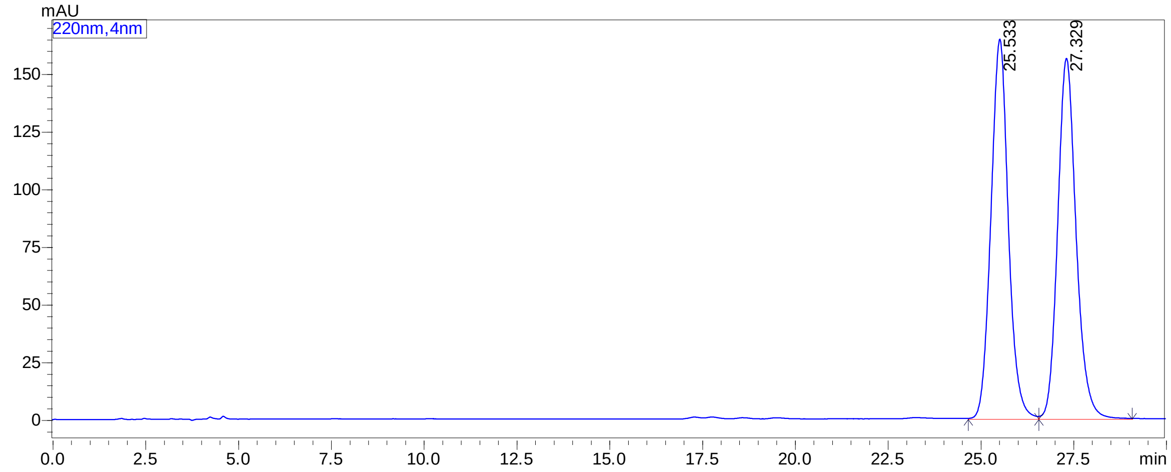

Peak#	Ret. Time	Area%
1	25.533	49.887
2	27.329	50.113
Total		100.000

HPLC data of (*R*)-**3ad**

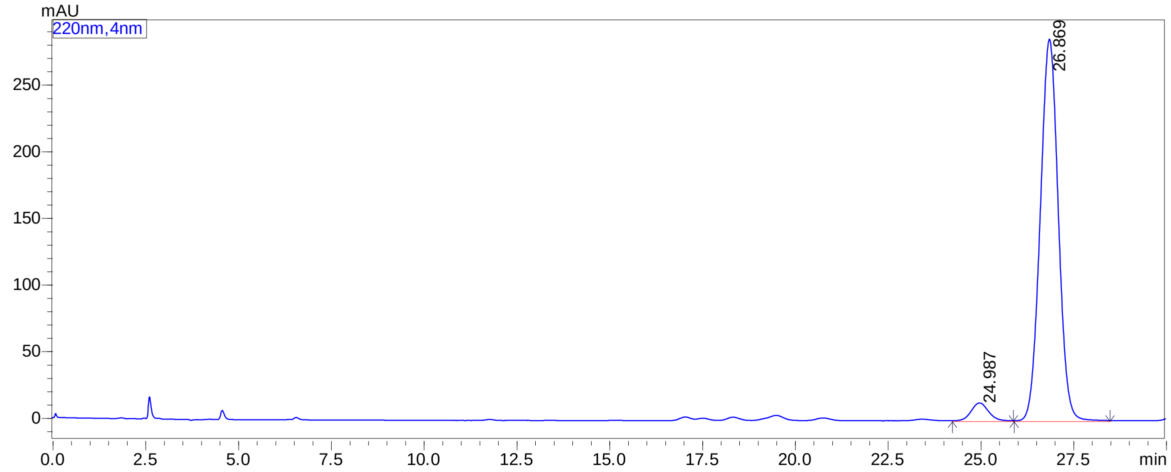

Peak#	Ret. Time	Area%
1	24.987	4.186
2	26.869	95.814
Total		100.000

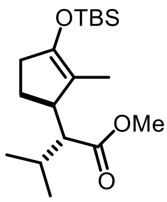

HPLC data of *rac*-**3ae**

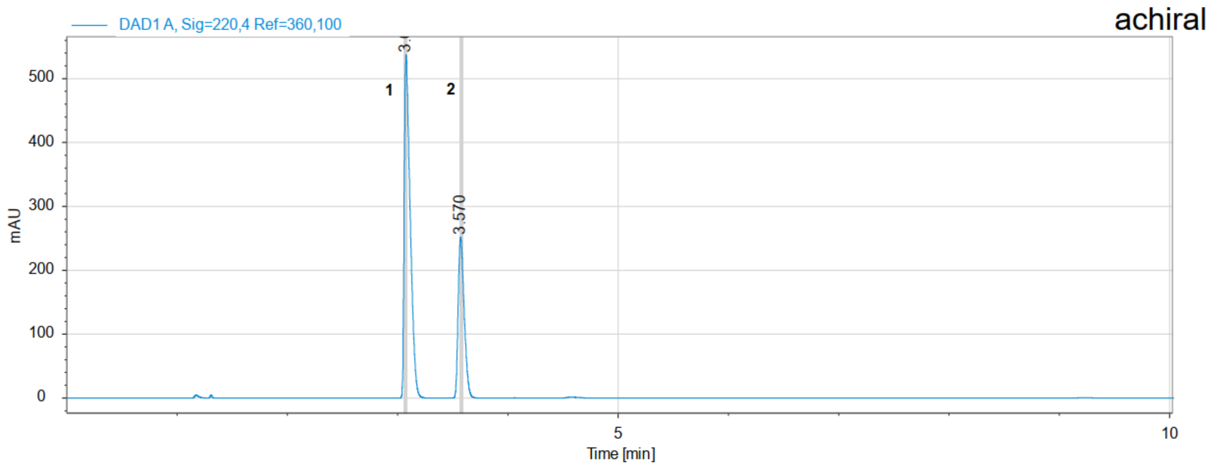

Peak#	Ret. Time
2	3.570

HPLC data of (*R*,*R*)-**3ae**

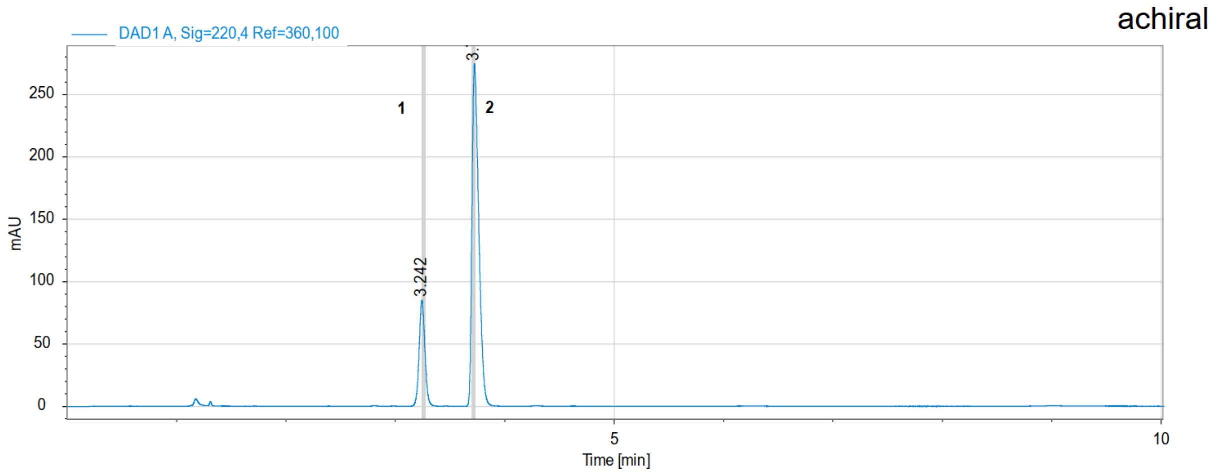

Peak#	Ret. Time
2	3.723

HPLC data of *rac*-**3ae**

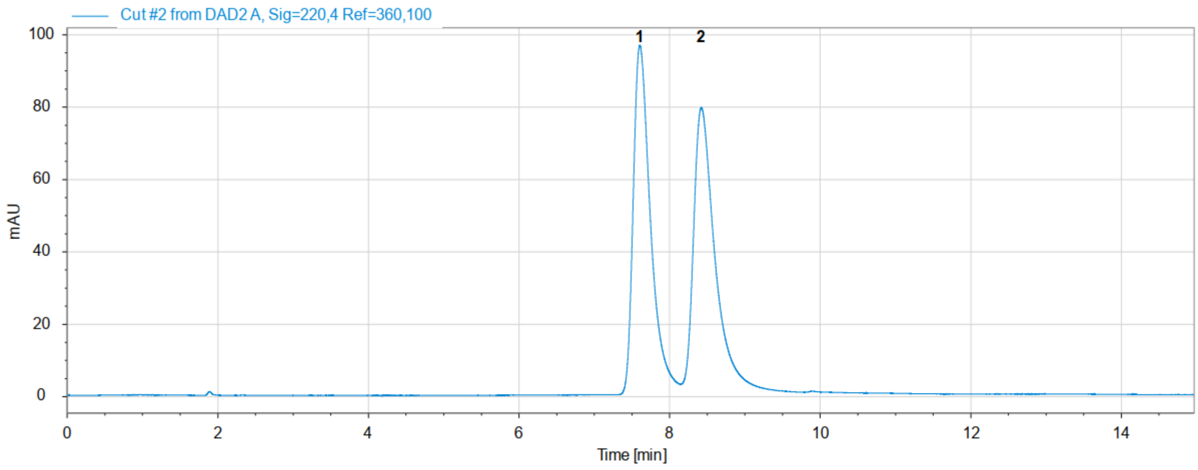

Peak#	Ret. Time	Area%
1	7.605	49.788
2	8.421	50.212
Total		100.000

HPLC data of (*R*,*R*)-**3ae**

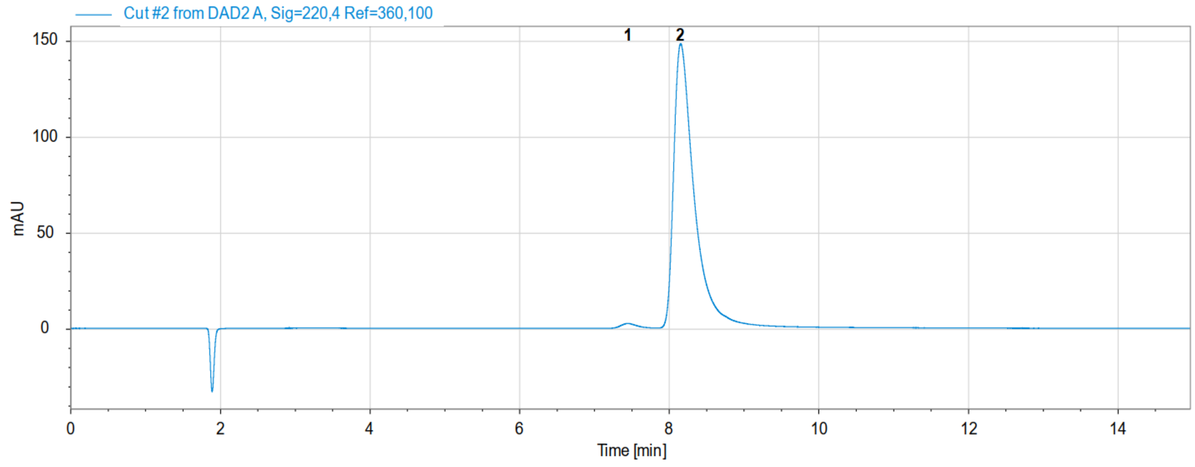

Peak#	Ret. Time	Area%
1	7.446	1.281
2	8.152	98.719
Total		100.000

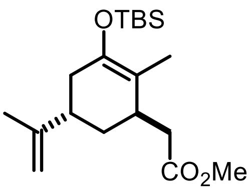

HPLC data of *rac*-**3af**

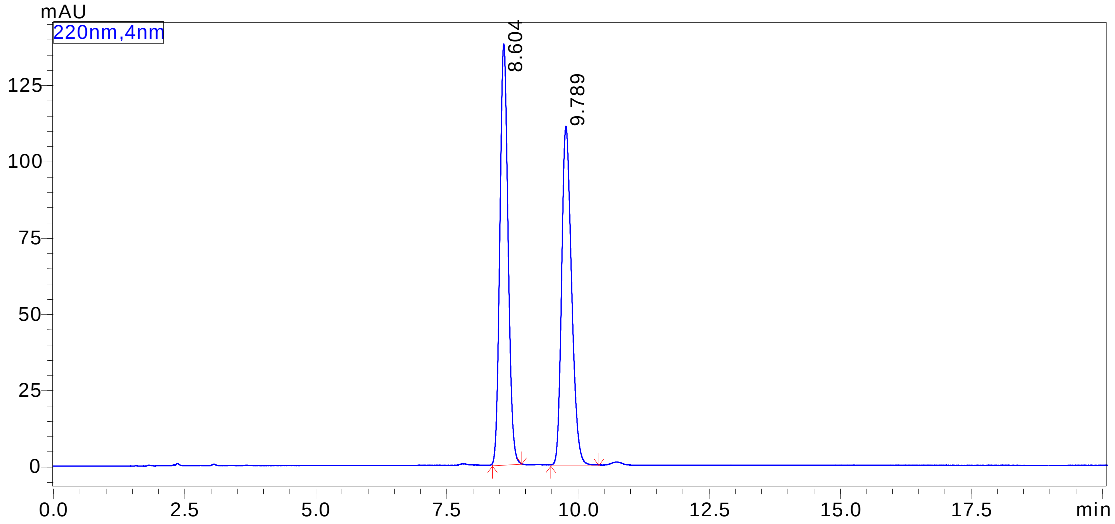

Peak	Ret. Time	Area%
1	8.604	50.604
2	9.789	49.396
Total		100.000

HPLC data of (1*R*,5*S*)-**3af**

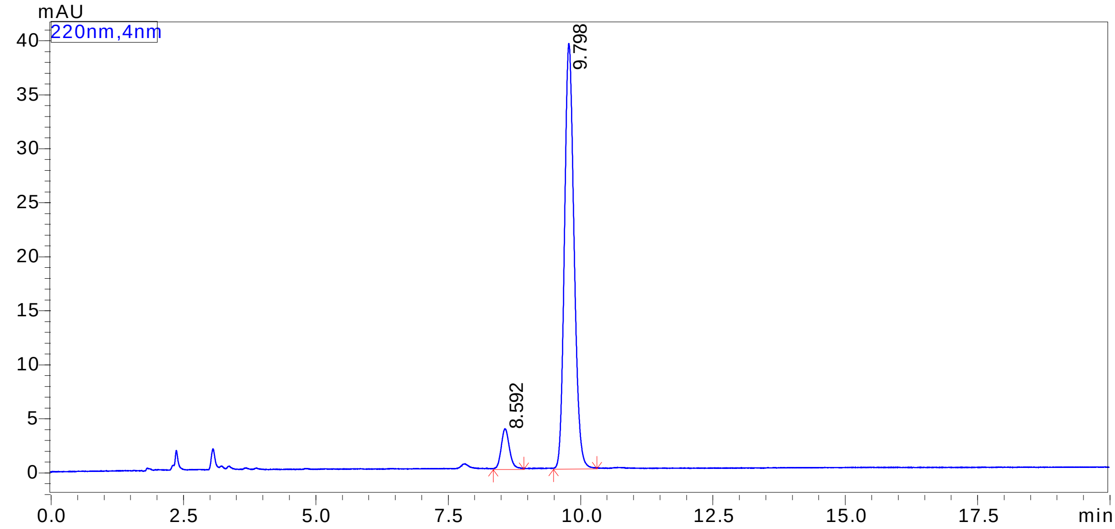

Peak	Ret. Time	Area%
1	8.592	7.319
2	9.798	92.681
Total		100.000

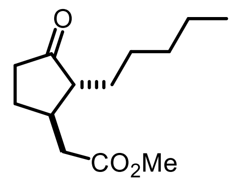

GC data of *rac*-**6a**

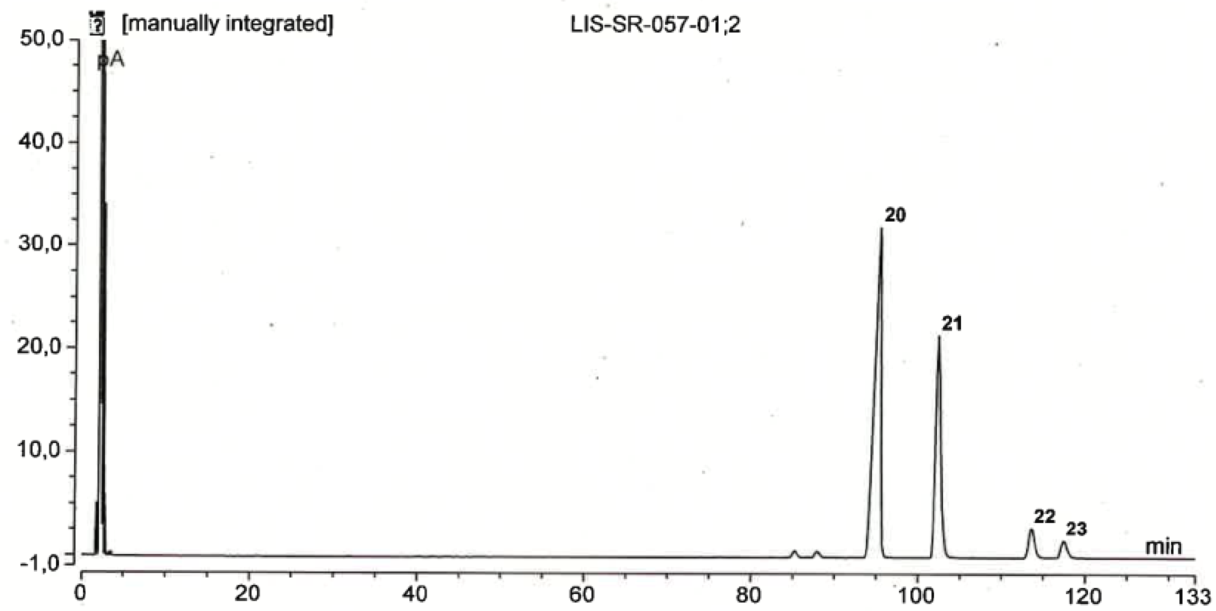

Sample:	**LIS-SR-057-01;2**	Instrument:	**GC_313**
Sequenz:	**11100 LIS-SR SE**	Measured:	**11.03.24 12:35**
Sequenz date:	**11.03.24**	Processing M.:	**ee 057**
		Report-File:	**Verhältnis 057-01**

Racemat
Verhältnis der Enantiomere
Zuordnung achiral nach GCMS 48130

No.	Ret.Time min	Rel.Area %	Peak Name
20	95,29	59,32	.
21	102,29	32,94	.
22	113,54	4,84	.
23	117,36	2,90	.

Instrument parameters:

Column:	30,0 m	BGB-176/BGB-15 0,25/0,25df G/618	
Temperature:	220/120 iso/350		
Gas:	0,60 bar	H2	
Sample size:	1,0 µL	Split ratio:	20 : 1

GC data of (1*R*,2*R*)-**6a**

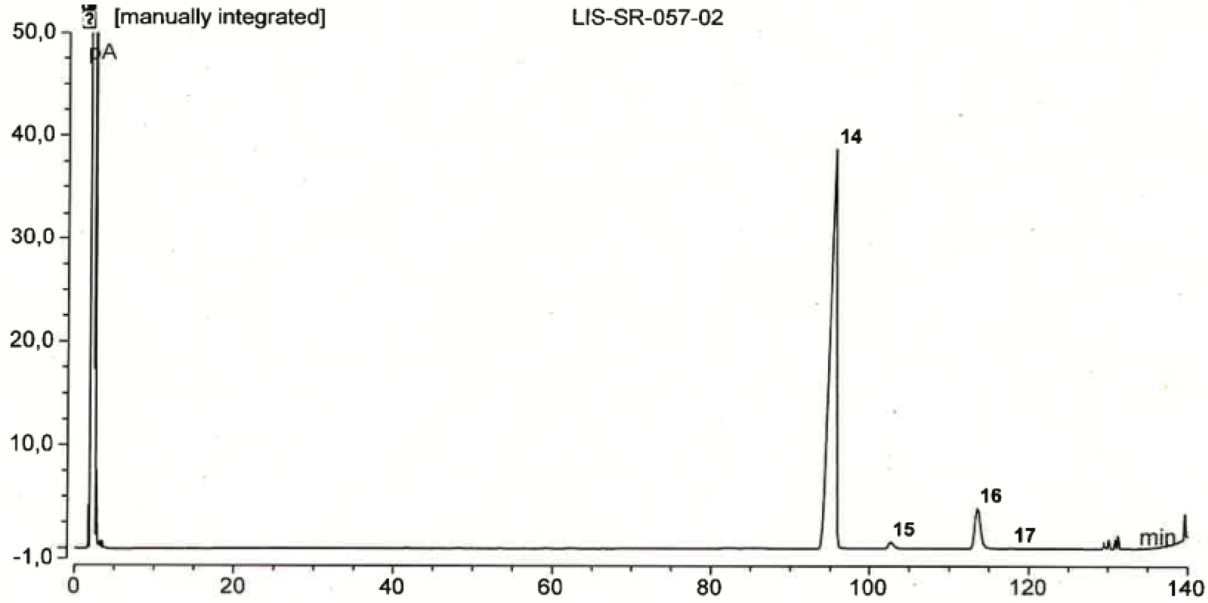

Sample:	**LIS-SR-057-02**	Instrument:	**GC_313**
Sequenz:	**11100 LIS-SR SE**	Measured:	**12.03.24 06:17**
Sequenz date:	**11.03.24**	Processing M.:	**ee 057**
		Report-File:	**Verhältnis 057-02**

Verhältnis der Enantiomere
Zuordnung nach Racemat GCMS 48130 LIS-SR-057-01 24/11100

No.	Ret.Time min	Rel.Area %	Peak Name
14	95,55	91,18	.
15	102,60	1,09	.
16	113,54	7,67	.
17	117,84	0,07	.

Instrument parameters:

Column:	30,0 m	BGB-176/BGB-15 0,25/0,25df G/618	
Temperature:	220/120 125 min iso 8/min 240/350		
Gas:	0,60 bar	H2	
Sample size:	1,0 µL	Split ratio:	10 : 1

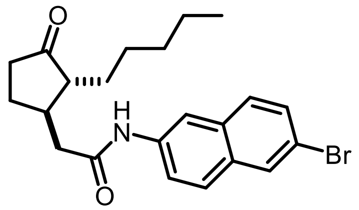

HPLC data of *rac*-**7**

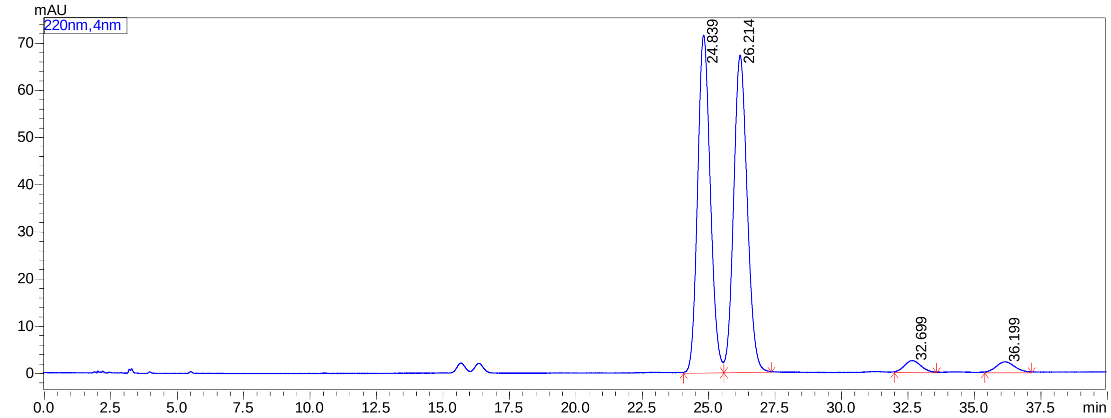

Peak	Ret. Time	Area%
1	24.839	47.878
2	26.214	48.077
3	32.699	2.031
4	36.199	2.014
Total		100.000

HPLC data of (1*R*,2*R*)-**7**

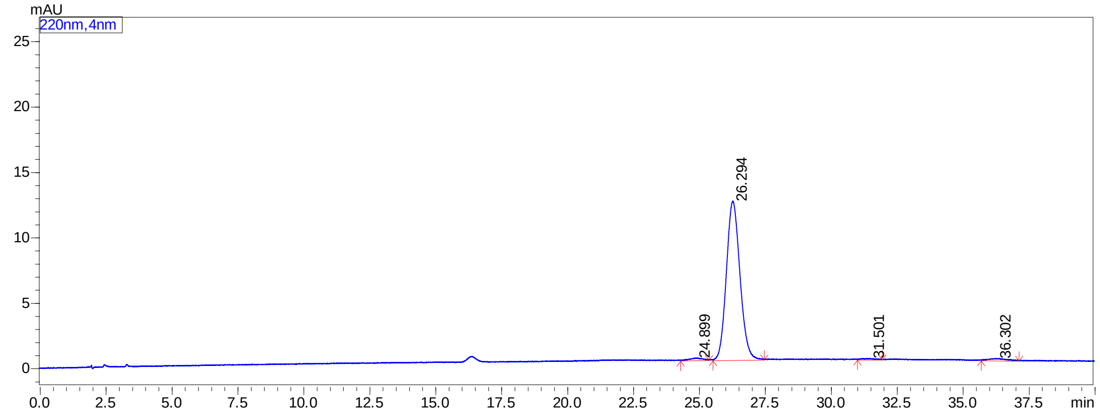

Peak	Ret. Time	Area%
1	24.899	0.971
2	26.294	97.760
3	31.501	0.236
4	36.302	1.033
Total		100.000

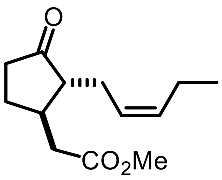

HPLC data of *rac*-**6c**

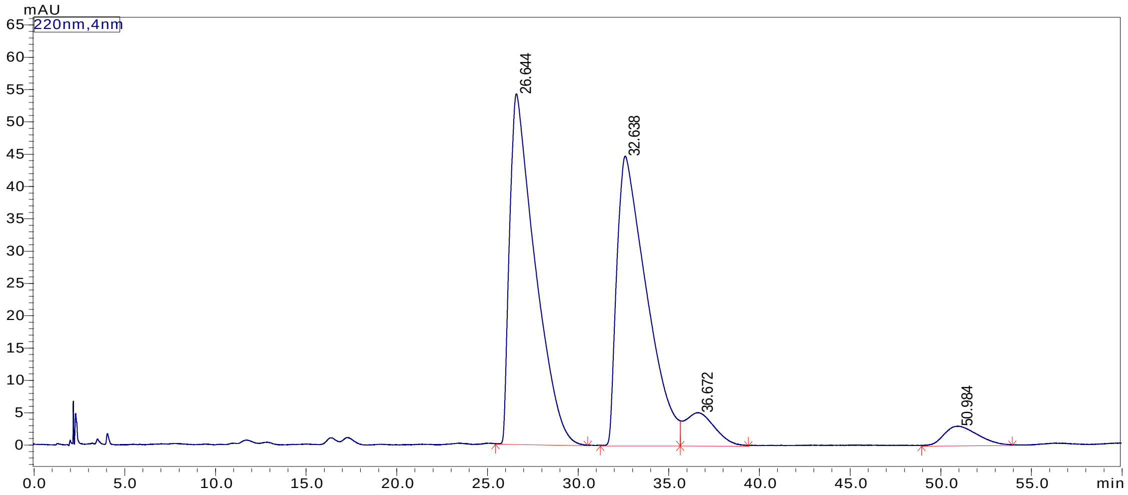

Peak	Ret. Time	Area%
1	26.644	46.525
2	32.638	45.022
3	36.672	5.202
4	50.984	3.251
Total		100.000

HPLC data of (1*R*,2*R*)-**6c**

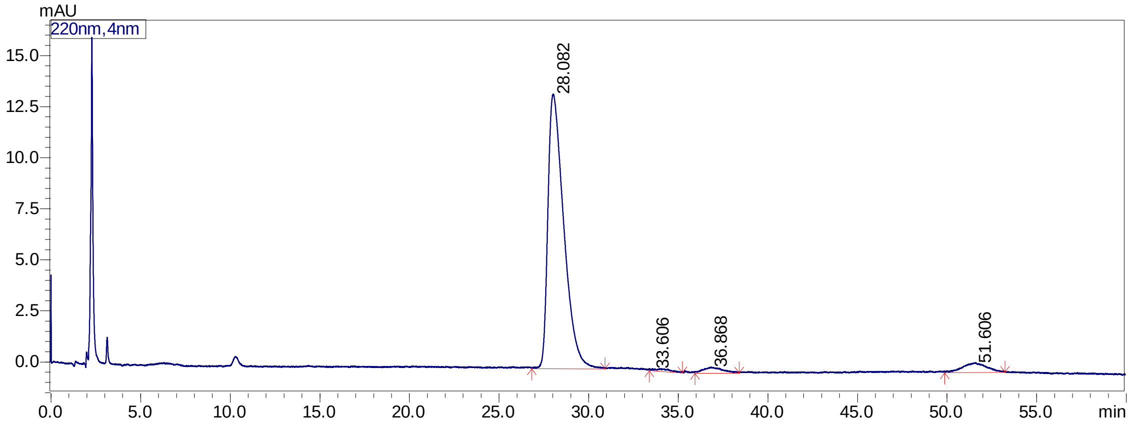

Peak	Ret. Time	Area%
1	28.082	93.550
2	33.606	0.467
3	36.868	1.780
4	51.606	4.203
Total		100.000

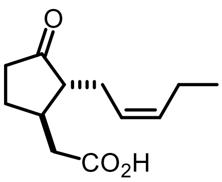

HPLC data of *rac*-**8**

**Instrument:**	NP-06 geclustert				
**Column**	Chiralcel OJ-3	length	150.00	i.D.	4.60

**Injection date:**	6/3/2024 1:12:30 PM	**Location:**	D1F-A1
**Acq. method:**	Xu.M	**Injection volume:**	1.000
**Analysis method:**	Guillén-HC.M	**Acq. operator:**	SYSTEM
**Last changed:**	6/4/2024 10:20:43 AM (modified after loading)		

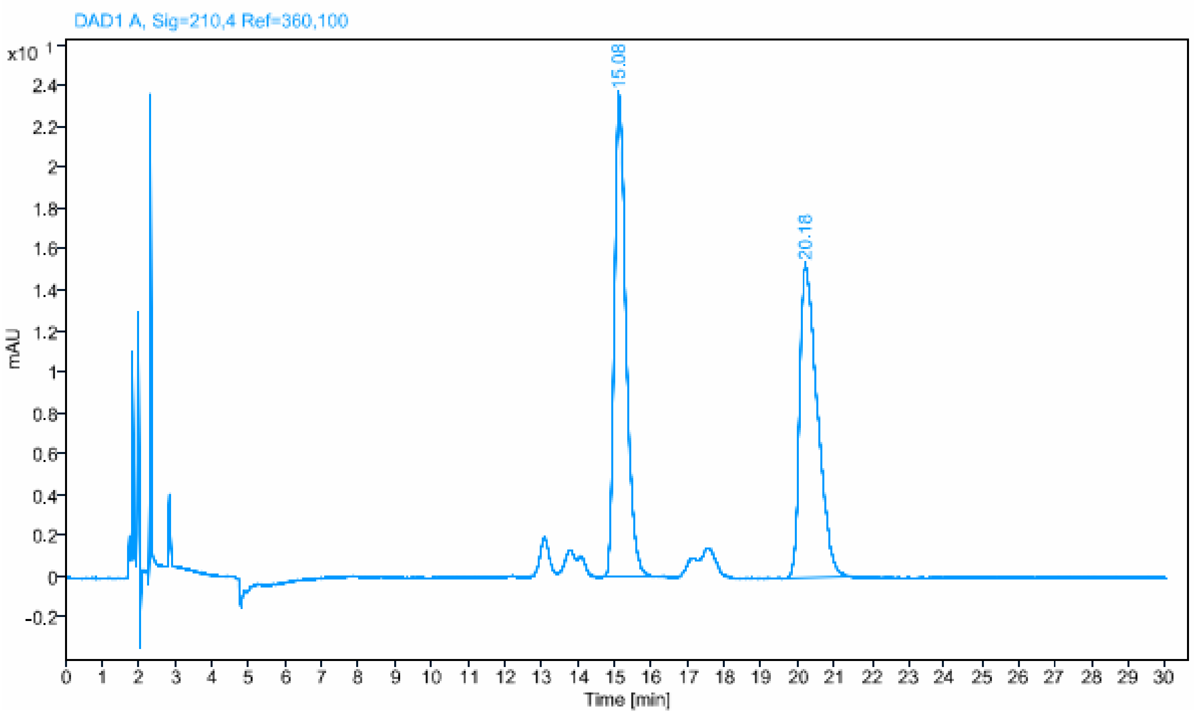

Signal: DAD1 A, Sig=210,4 Ref=360,100

RT [min]	Area	Area%	Name
15.08	518.98	49.97	
20.18	519.57	50.03	
Sum	1038.5541		

HPLC data of (1*R*,2*R*)-**8**

**Instrument:**	NP-06 geclustert				
**Column**	Chiralcel OJ-3	length	150.00	i.D.	4.60

Injection date: 6/3/2024 1:46:13 PM
Acq. method: Xu.M
Analysis method: Guillén-HC.M
Last changed: 6/4/2024 10:20:43 AM
(modified after loading)

Location: D1F-A2
Injection volume: 5.000
Acq. operator: SYSTEM

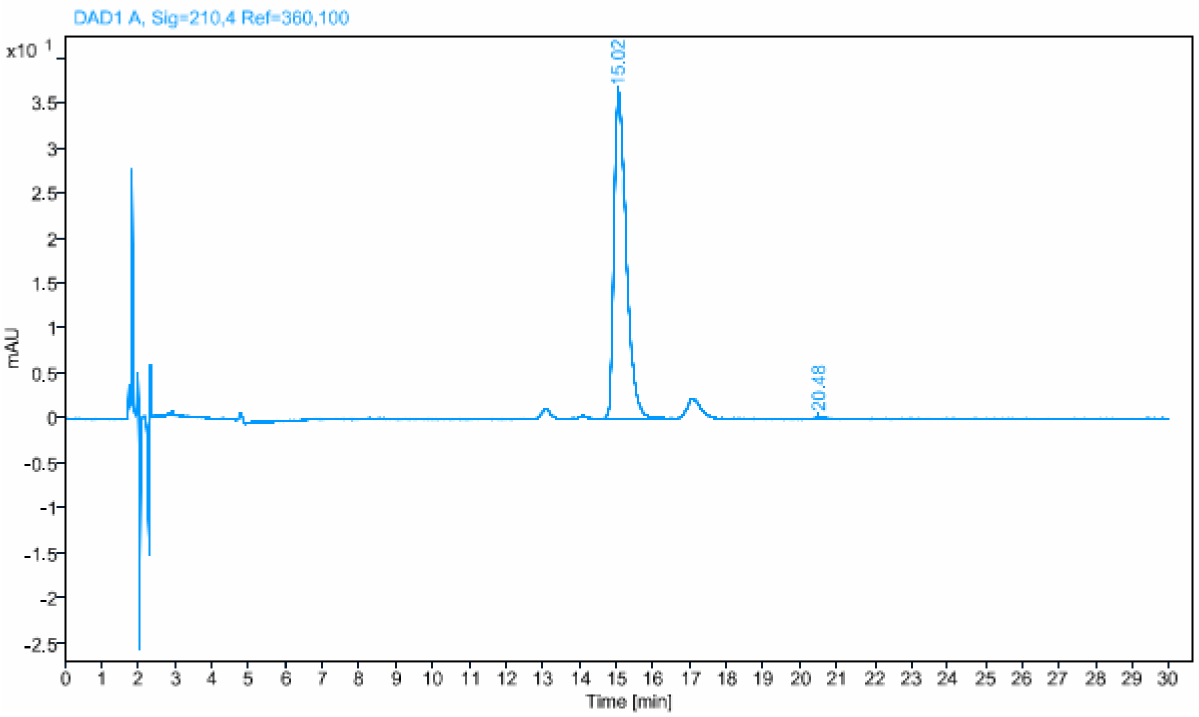

Signal: DAD1 A, Sig=210,4 Ref=360,100

RT [min]	Area	Area%	Name
15.02	827.51	99.51	
20.48	4.04	0.49	
Sum	831.5494		

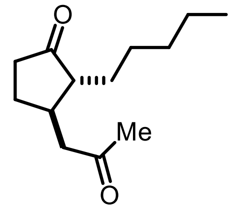

GC data of *rac*-**6b**

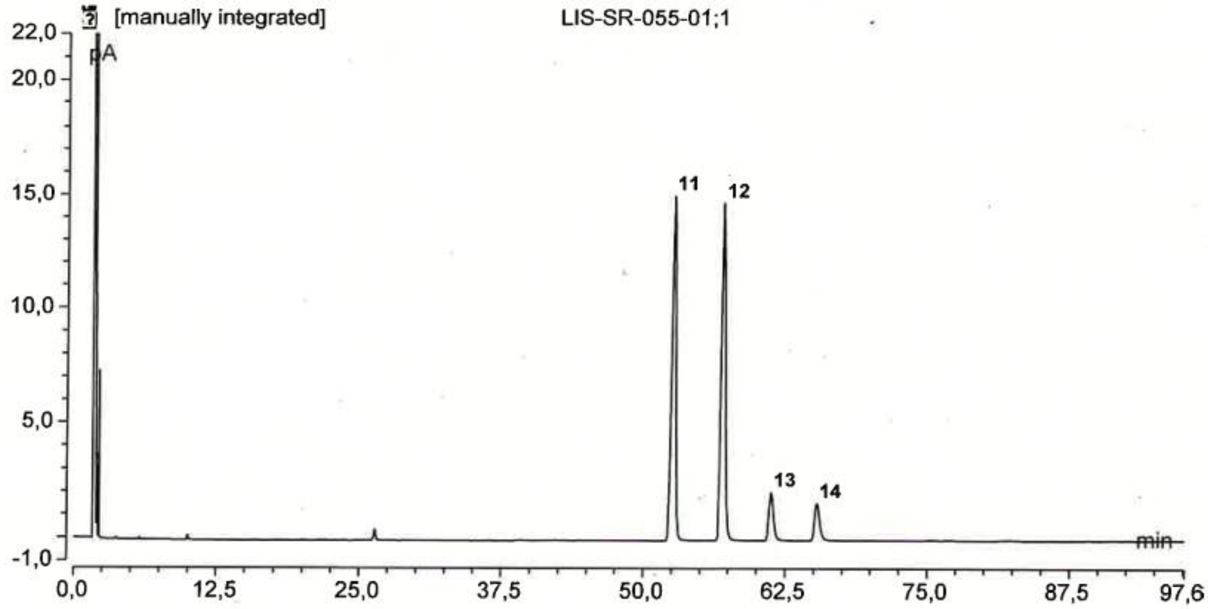

Sample:	**LIS-SR-055-01;1**	Instrument:	**GC_313**
Sequenz:	**11079 LIS-SR SE**	Measured:	**01.03.24 10:04**
Sequenz date:	**01.03.24**	Processing M.:	**ee LIS**
		Report-File:	**Verhältnis 055-01**

Racemat
Verhältnis der Enantiomere
Zuordnung achiral nach GCMS 47990 LIS-SR-055-01 24/11079

No.	Ret.Time min	Rel.Area %	Peak Name
11	52;61	43,12	.
12	56,95	44,26	.
13	61,24	6,88	.
14	65,26	5,75	.

Instrument parameters:

Column:	30,0 m	BGB-176/BGB-15 0,25/0,25df G/618	
Temperature:	220/130 iso/350		
Gas:	0,60 bar	H2	
Sample size:	1,0 µL	Split ratio:	20 : 1

GC data of (1*R*,2*R*)-**6b**

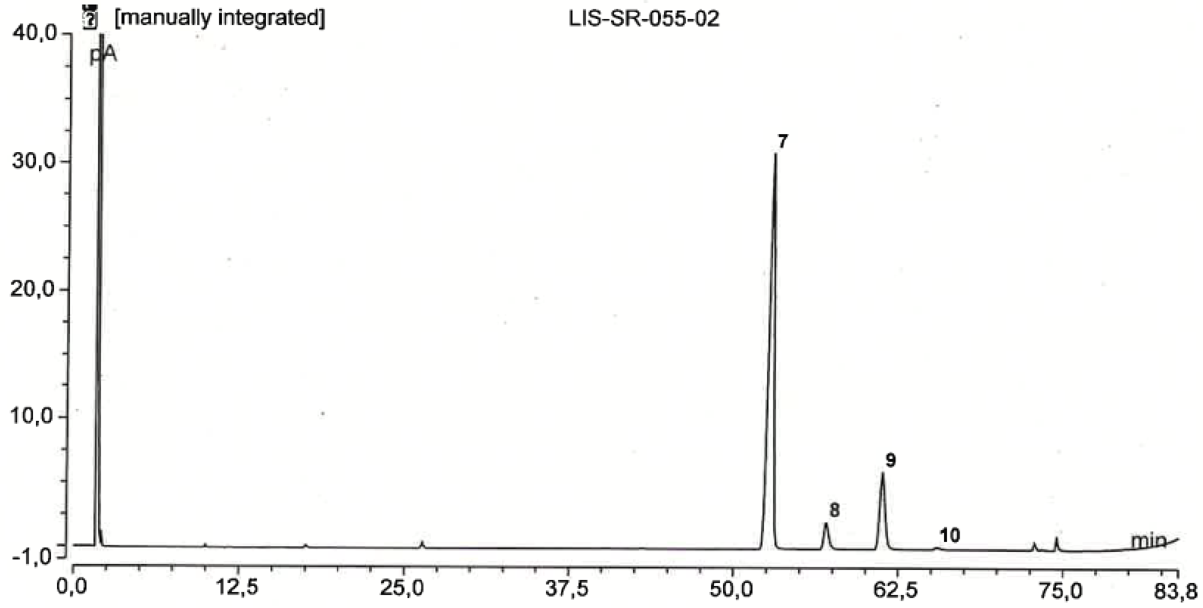

Sample:	**LIS-SR-055-02**	Instrument:	**GC_313**
Sequenz:	**11079 LIS-SR SE**	Measured:	**01.03.24 12:04**
Sequenz date:	**01.03.24**	Processing M.:	**ee LIS**
		Report-File:	**Verhältnis 055-02**

Verhältnis der Enantiomere
Zuordnung achiral nach Racemat GCMS 47990 LIS-SR-055-01 24/11079

**No.**	**Ret.Time min**	**Rel.Area %**	**Peak Name**
7	52,84	78,95	.
8	56,99	5,04	.
9	61,26	15,43	.
10	65,38	0,58	.

Instrument parameters:

Column:	30,0 m	BGB-176/BGB-15 0,25/0,25df G/618	
Temperature:	220/130 70min iso 8/min 240/350		
Gas:	0,60 bar	H2	
Sample size:	1,0 µL	Split ratio:	20 : 1

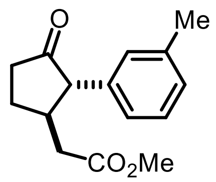

GC data of *rac*-**6d**

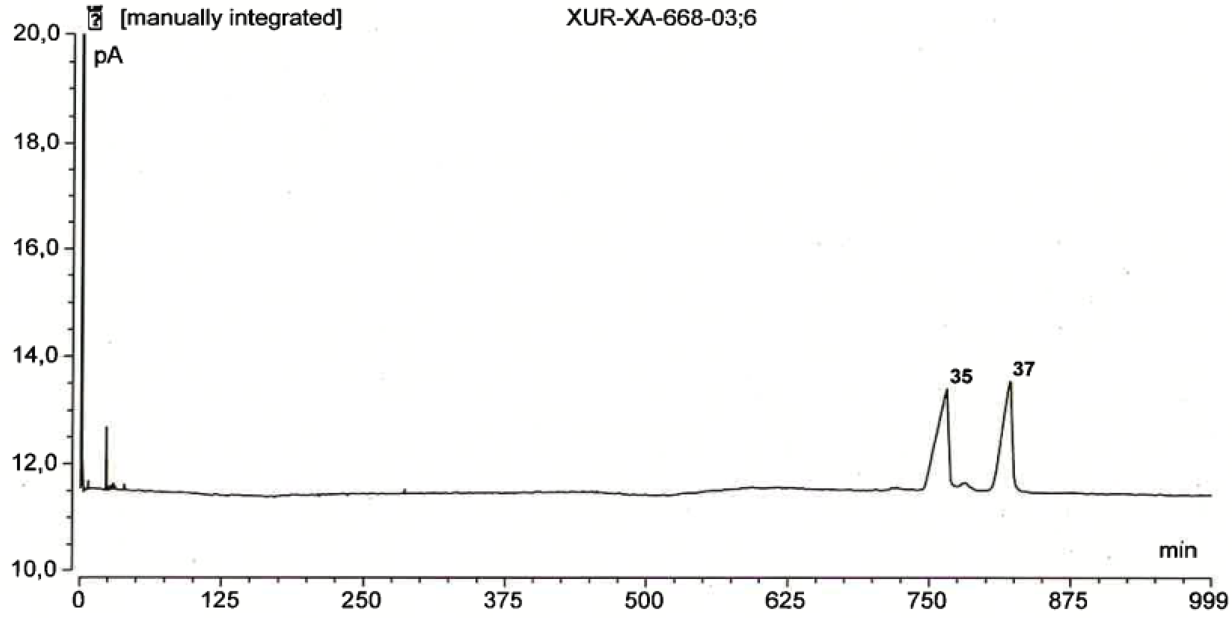

Sample:	**XUR-XA-668-03;6**	Instrument:	**GC_511**
Sequenz:	**11113 XUR-XA JK**	Measured:	**19.03.24 08:41**
Sequenz date:	**13.03.24**	Processing M.:	**ee XUR-XA**
		Report-File:	**668-03**

chirale Messung des Racemates
Verhältnis der Enantiomere aufgrund geringer Konzentration ungenau
Es konnte nur für ein Enantiomerenpaar eine Trennung erzielt werden

No.	Ret.Time min	Rel.Area %	Peak Name
35	764,90	52,09	.
37	820,26	47,91	.

Instrument parameters:

Column:	21,5 m	Ivadex-5/OV-1701 0,25/0,15df G/616	
Temperature:	220/ 100 iso/350		
Gas:	0,50 bar	Hydrogen	
Sample size:	1,0 µL	Split ratio:	10 : 1

GC data of (1*R*,2*S*)-**6d**

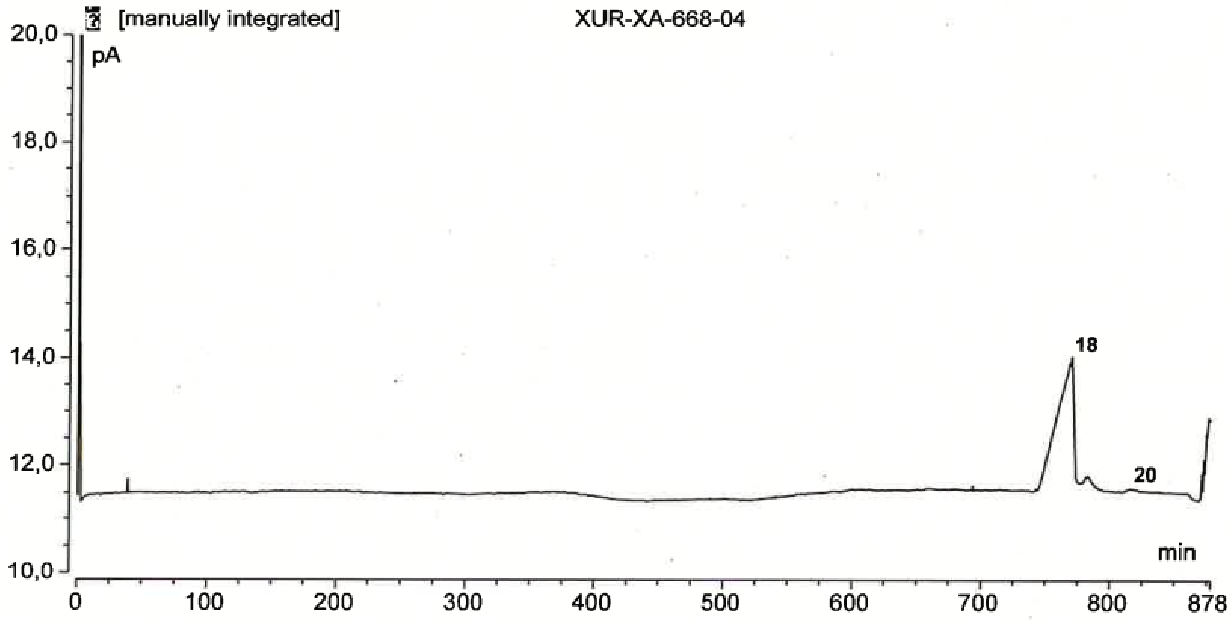

Sample: **XUR-XA-668-04**
Sequenz: **11114 XUR-XA JK**
Sequenz date: **20.03.24**

Instrument: **GC_511**
Measured: **20.03.24 08:21**
Processing M.: **ee XUR-XA**
Report-File: **668-04**

chirale Messung der Probe
Verhältnis der Enantiomere aufgrund geringer Konzentration ungenau
Es konnte nur für ein Enantiomerenpaar eine Trennung erzielt werden

No.	Ret.Time min	Rel.Area %	Peak Name
18	769,91	98,85	.
20	816,67	1,15	.

Instrument parameters:

Column:	21,5 m	Ivadex-5/OV-1701 0,25/0,15df G/616	
Temperature:	220 /100, 860min iso 8/min 220 3min iso/ 350		
Gas:	0,50 bar	Hydrogen	
Sample size:	1,0 µL	Split ratio:	5 : 1

1 1. 01

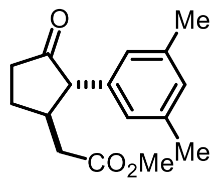

GC data of *rac*-**6e**

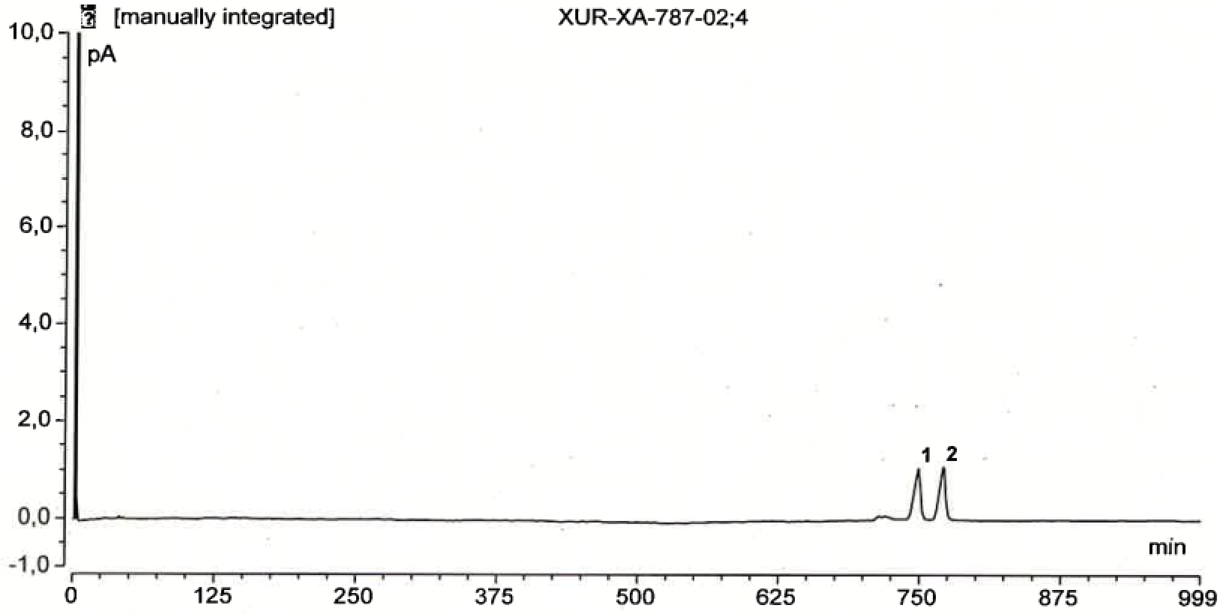

Sample:	**XUR-XA-787-02;4**	Instrument:	**GC_313**
Sequenz:	**11109 XUR-XA Sch**	Measured:	**19.03.24 08:40**
Sequenz date:	**13.03.24**	Processing M.:	**ee XUR-XA**
		Report-File:	**787-02**

chirale Messung des Racemates
Verhältnis der Enantiomere aufgrund geringer Konzentration ungenau
Es konnte nur für ein Enantiomerenpaar eine Trennung erzielt werden

**No.**	**Ret.Time** min	**Rel.Area** %	**Peak Name**
1	749,14	50,27	.
2	771,08	49,73	.

Instrument parameters:

Column:	30,0 m	BGB-176/BGB-15 0,25/0,25df G/618	
Temperature:	220/ 120 iso/350		
Gas:	0,60 bar	H2	
Sample size:	1,0 µL	Split ratio:	20 : 1

J. Keßler

GC data of (1*R*,2*S*)-**6e**

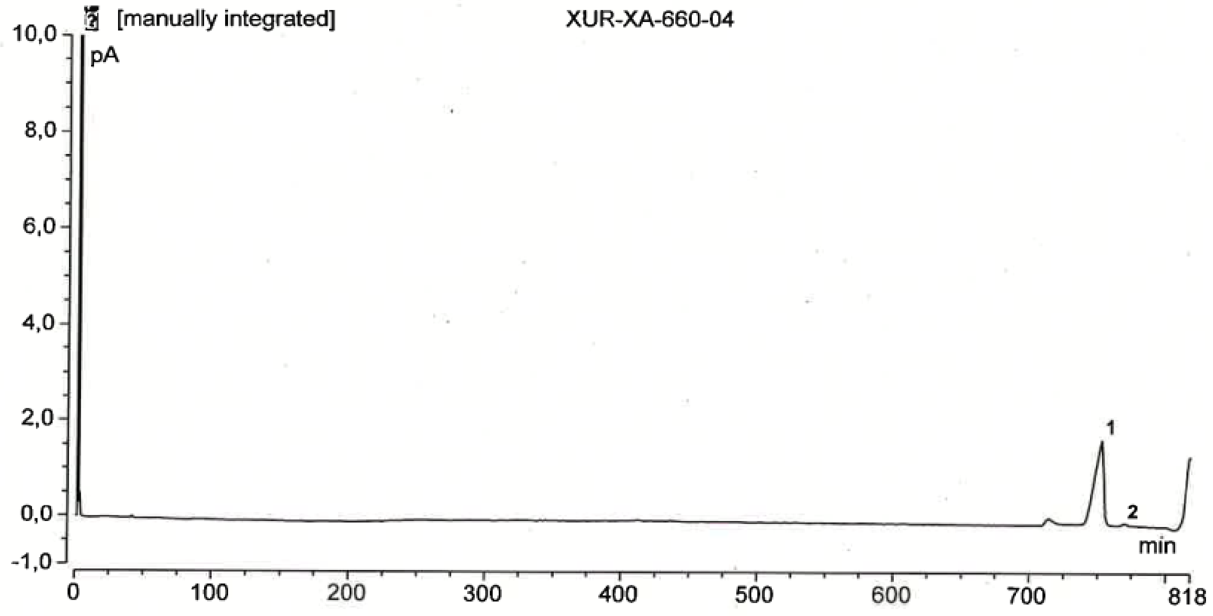

Sample:	**XUR-XA-660-04**	Instrument:	**GC_313**
Sequenz:	**11112 XUR-XA Sch**	Measured:	**25.03.24 14:42**
Sequenz date:	**25.03.24**	Processing M.:	**ee XUR-XA**
		Report-File:	**660-04**

chirale Messung der Probe
Verhältnis der Enantiomere aufgrund geringer Konzentration ungenau
Es konnte nur für ein Enantiomerenpaar eine Trennung erzielt werden

No.	Ret.Time min	Rel.Area %	Peak Name
1	752,62	98,10 .	
2	769,97	1,90 .	

Instrument parameters:

Column:	30,0 m	BGB-176/BGB-15 0,25/0,25df G/618	
Temperature:	220 / 120 800min iso 8/min 240 3min iso /350		
Gas:	0,60 bar	H2	
Sample size:	1,0 µL	Split ratio:	20 : 1

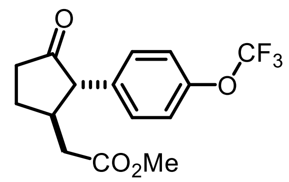

GC data of *rac*-**6f**

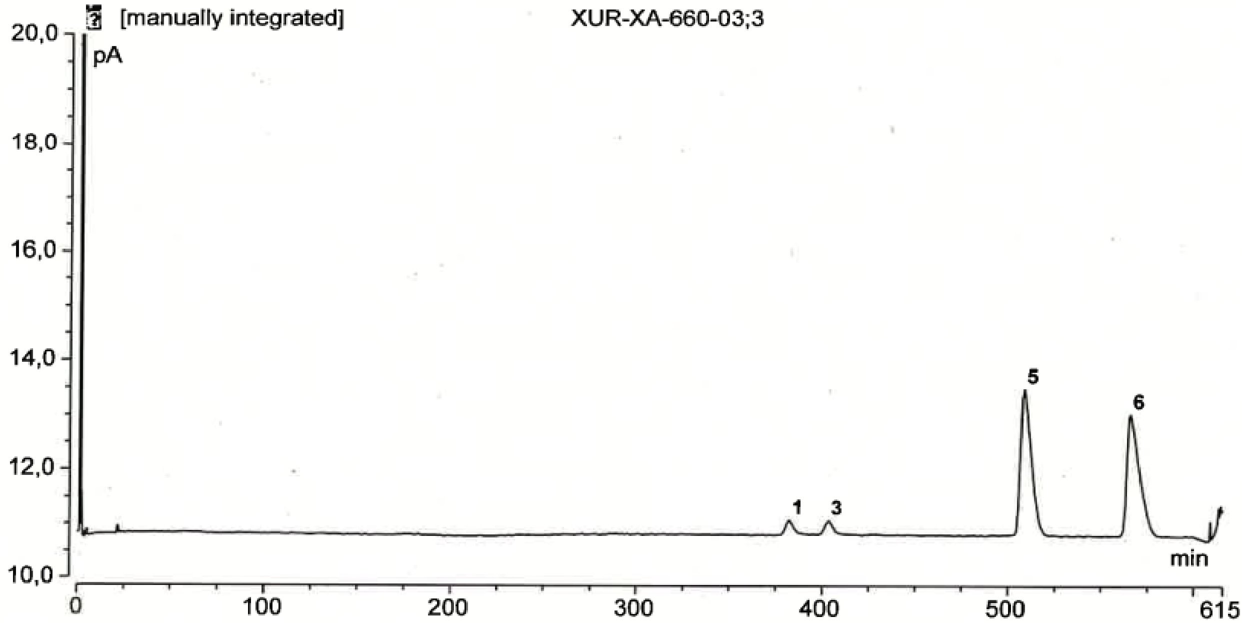

Sample:	XUR-XA-660-03;3	Instrument:	GC_413
Sequenz:	11111 XUR-XA Sch	Measured:	14.03.24 14:22
Sequenz date:	12.03.24	Processing M.:	XUR-XA
		Report-File:	XUR-XA-660-03

chirale Messung des Racemates
Verhältnis der Enantiomere aufgrund zu geringer Konzentration ungenau

No.	Ret.Time min	Rel.Area %	Peak Name
1	382,37	3,71	.
3	403,26	3,83	.
5	508,32	46,42	.
6	565,39	46,04	.

Instrument parameters:

Column:	25,0 m	Hydrodex-beta-TBDAc-CD 0,25/?df G/681
Temperature:	220/ 120, 600 min	iso 8/min 220, 3 min iso / 350
Gas:	0,60 bar	Hydrogen
Sample size:	1,0 µL	Split ratio: 5 : 1

GC data of (1*R*,2*S*)-**6f**

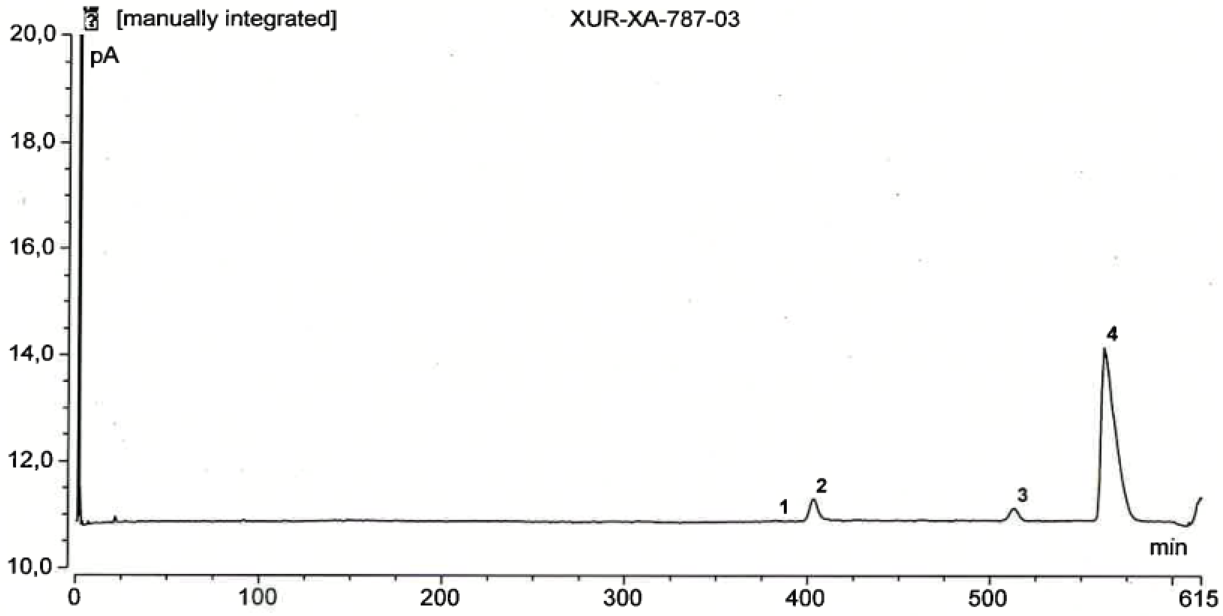

Sample:	**XUR-XA-787-03**	Instrument:	**GC_413**
Sequenz:	**11110 XUR-XA Sch**	Measured:	**20.03.24 12:15**
Sequenz date:	**20.03.24**	Processing M.:	**XUR-XA**
		Report-File:	**787-03**

chirale Messung der Probe
Verhältnis der Enantiomere aufgrund zu geringer Konzentration ungenau

No.	Ret.Time min	Rel.Area %	Peak Name
1	382,93	0,29	? Konzentration zu gering
2	402,85	7,10	.
3	512,68	4,36	.
4	561,62	88,25	.

Instrument parameters:

Column:	25,0 m	Hydrodex-beta-TBDAc-CD 0,25/?df G/681
Temperature:	220/ 120, 600 min	iso 8/min 220, 3 min iso / 350
Gas:	0,60 bar	Hydrogen
Sample size:	1,0 µL	Split ratio: 5 : 1

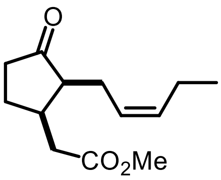

HPLC data of *rac*-**9a**

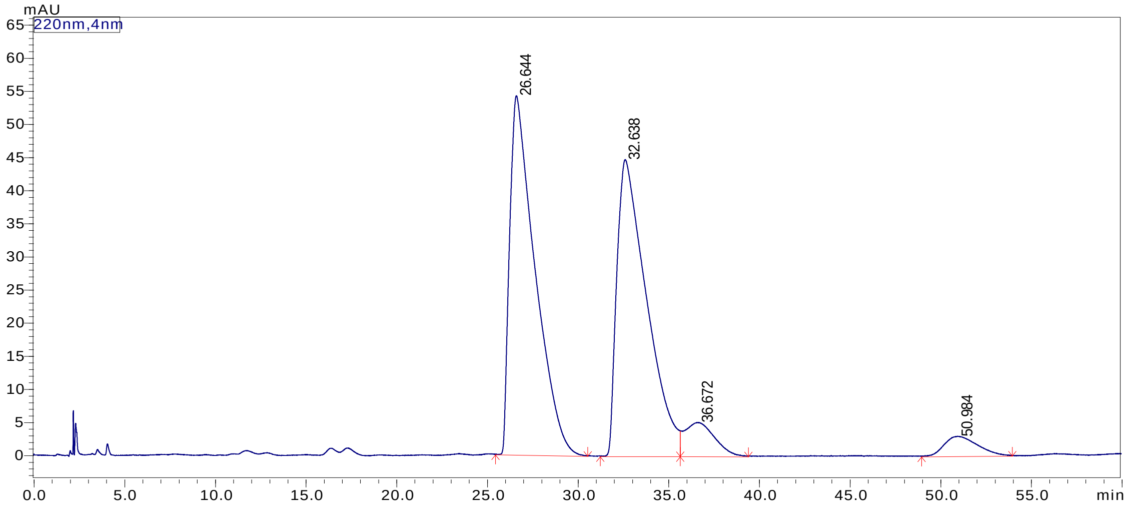

Peak	Ret. Time	Area%
1	26.644	46.525
2	32.638	45.022
3	36.672	5.202
4	50.984	3.251
Total		100.000

HPLC data of (1*R*,2*S*)-**9a**

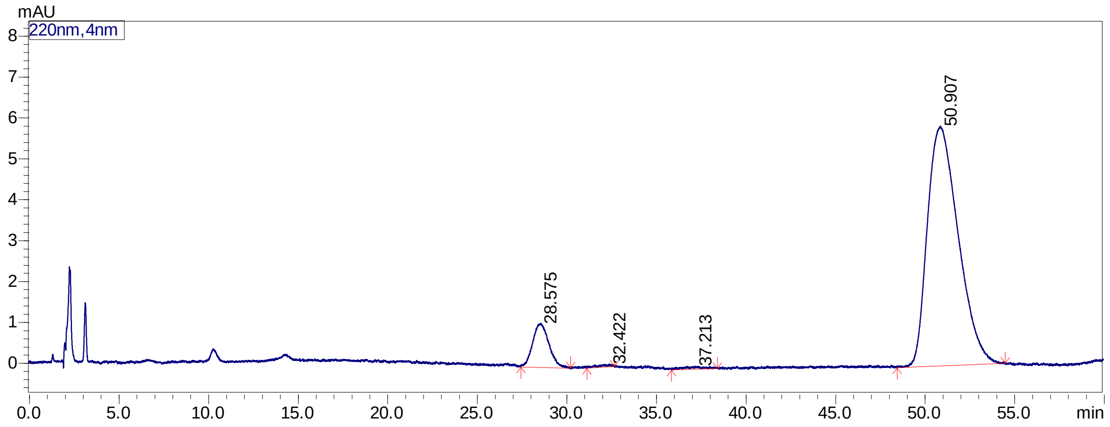

Peak	Ret. Time	Area%
1	28.575	8.427
2	32.422	0.131
3	37.213	0.273
4	50.907	91.169
Total		100.000

GC data of *rac*-**9b**

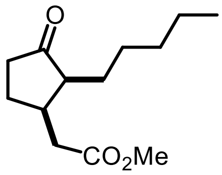

Gerät : RP-10 Scout II

Operator : Hi
Sample Name : LIS-SR-022-01
Vial # : 1
Injection Volume : 5 uL
Data File Name : LIS-SR-022-01-IG-ACN-04.lcd
Method File Name : Xu-CH3CN.lcm

Data Acquired: 11/8/2023 3:17:57 PM

<<Oven>>
Valve 1/L Position : 6: IG-3
Valve 2/R Position : 0

5 µl LIS-SR-022-01 (5 µl in 1 ml ACN)
150 mm Chiralpak IG-3, 4.6 mm i.D.
Acetonitril / Wasser-Gradient:
50 % - 10' - 70 % ACN
1.0 ml/min, 19.1 MPa, 298 K
UV 210 nm

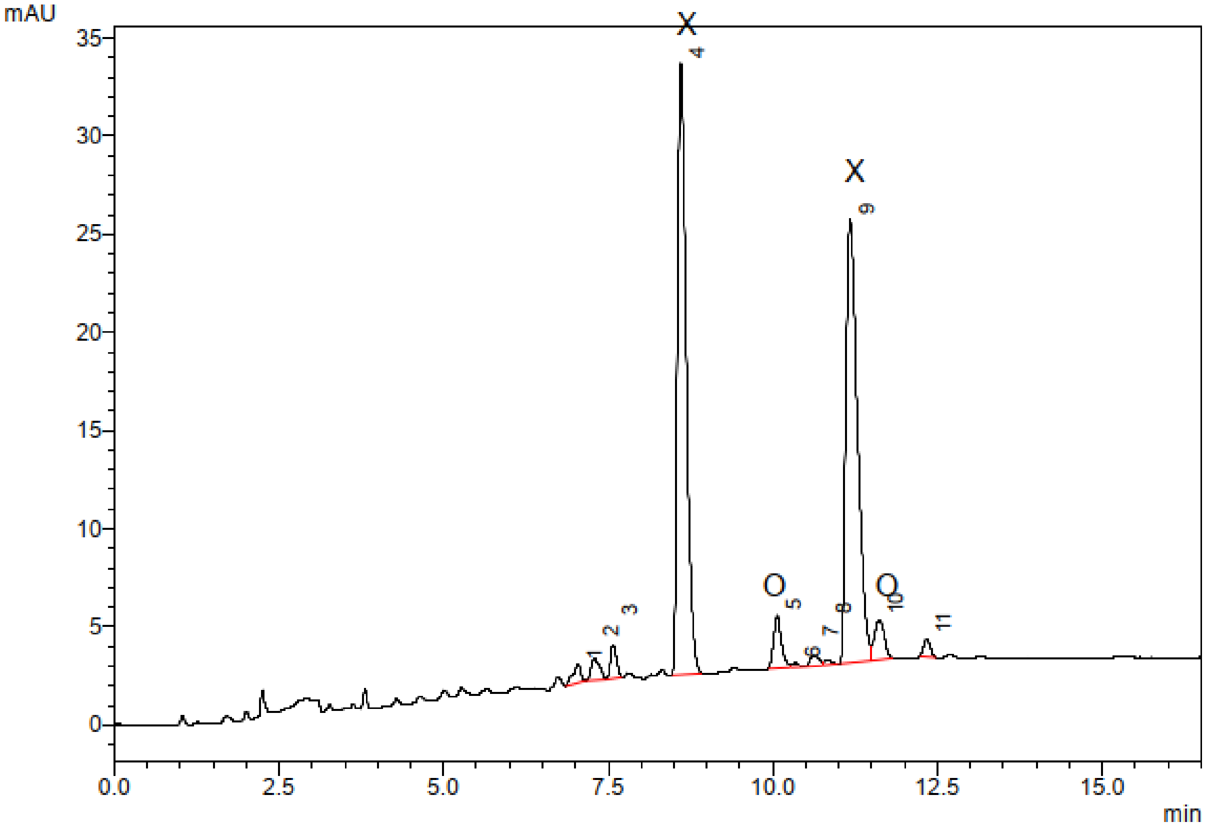

1 210nm,4nm

PDA Ch1 210nm

Peak #	Ret. Time	Area %	Name
1	7.04	1.27	
2	7.29	1.59	
3	7.58	1.73	
4	8.60	43.81	1st enantiomer 1st diastereomer
5	10.07	3.52	1st enantiomer 2nd diastereomer
6	10.35	0.26	
7	10.63	0.81	
8	10.83	0.32	
9	11.17	42.44	2nd enantiomer 1st diastereomer
10	11.62	3.26	2nd enantiomer 2nd diastereomer
11	12.34	0.99	
Total		100.00	

X: 1st enantiomer 1st diastereomer, 2nd enantiomer 1st diastereomer
O: 1st enantiomer 2nd diastereomer, 2nd enantiomer 2nd diastereomer

GC data of (1*R*,2*S*)-**9b** (0.1 mmol scale)

Gerät : RP-10 Scout II

Operator : Hi
Sample Name : LIS-SR-022-02
Vial # : 2
Injection Volume : 5 uL
Data File Name : LIS-SR-022-02-IG-ACN-05.lcd
Method File Name : Xu-CH3CN.lcm

Data Acquired: 11/8/2023 3:38:47 PM

<<Oven>>
Valve 1/L Position : 6: IG-3
Valve 2/R Position : 0

5 µl LIS-SR-022-02 (5 µl in 1 ml ACN)
150 mm Chiralpak IG-3, 4.6 mm i.D.
Acetonitril / Wasser-Gradient:
50 % - 10' - 70 % ACN
1.0 ml/min, 19.1 MPa, 298 K
UV 210 nm

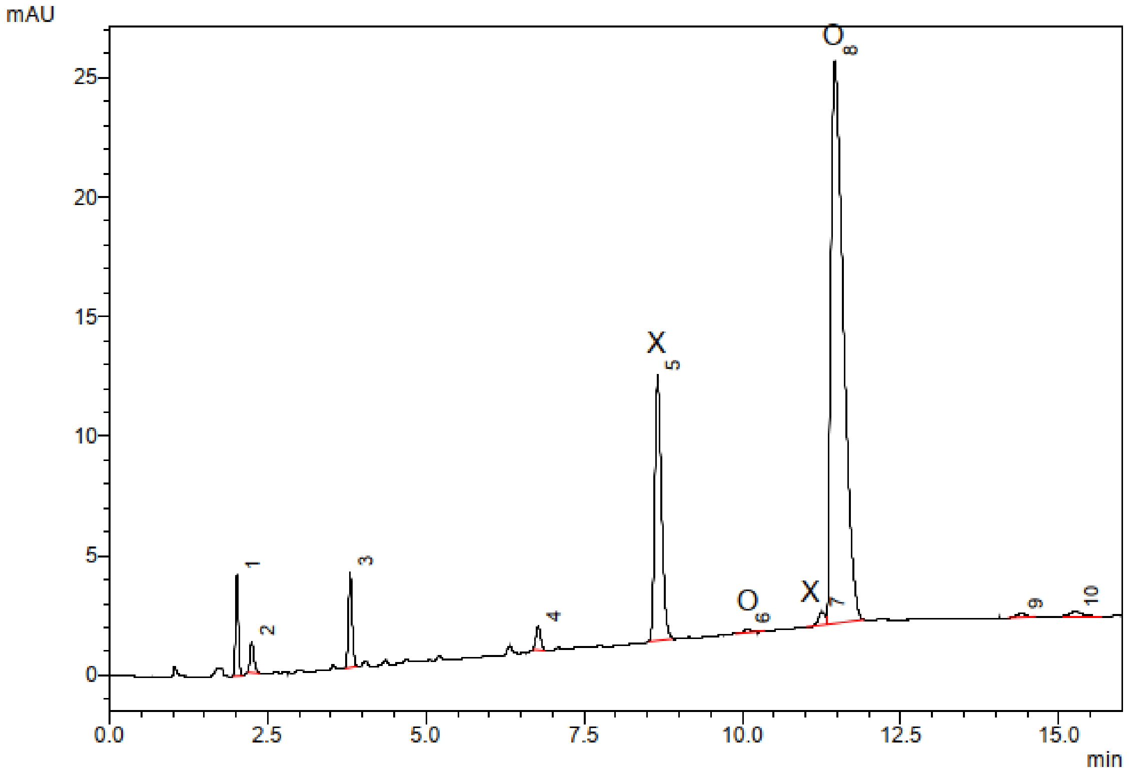

1 210nm,4nm

PDA Ch1 210nm

Peak #	Ret. Time	Area %	Name
1	2.02	2.90	
2	2.25	1.21	
3	3.80	3.55	
4	6.77	1.11	
5	8.66	18.66	1st enantiomer 1st diastereomer
6	10.08	0.24	1st enantiomer 2nd diastereomer
7	11.26	0.79	2nd enantiomer 1st diastereomer
8	11.46	70.58	2nd enantiomer 2nd diastereomer
9	14.38	0.39	
10	15.26	0.57	
Total		100.00	

X — = 91.9 % ee

O — = 99.3 % ee

GC data of (1*R*,2*S*)-**9b** (100 mmol scale)

Gerät : RP-10 Scout II

Operator : Hi
Sample Name : LIS-SR-113-03
Vial # : 2
Injection Volume : 2 uL
Data File Name : LIS-SR-113-03-01.lcd
Method File Name : Xu-CH3CN.lcm

Data Acquired: 9/27/2024 12:39:50 PM

<<Oven>>
Valve 1/L Position : 6: IG-3
Valve 2/R Position : 0

2 µL LIS-SR-113-03 (5 µl in 0.5 ml ACN)
150 mm Chiralpak IG-3 4.6mm i.D.
Acetonitril / Wassert-Gradient:
50 % B - 10' - 70 % ACN
1.0 mL/min, 19.0 MPa, 298 K
DAD, 210 nm

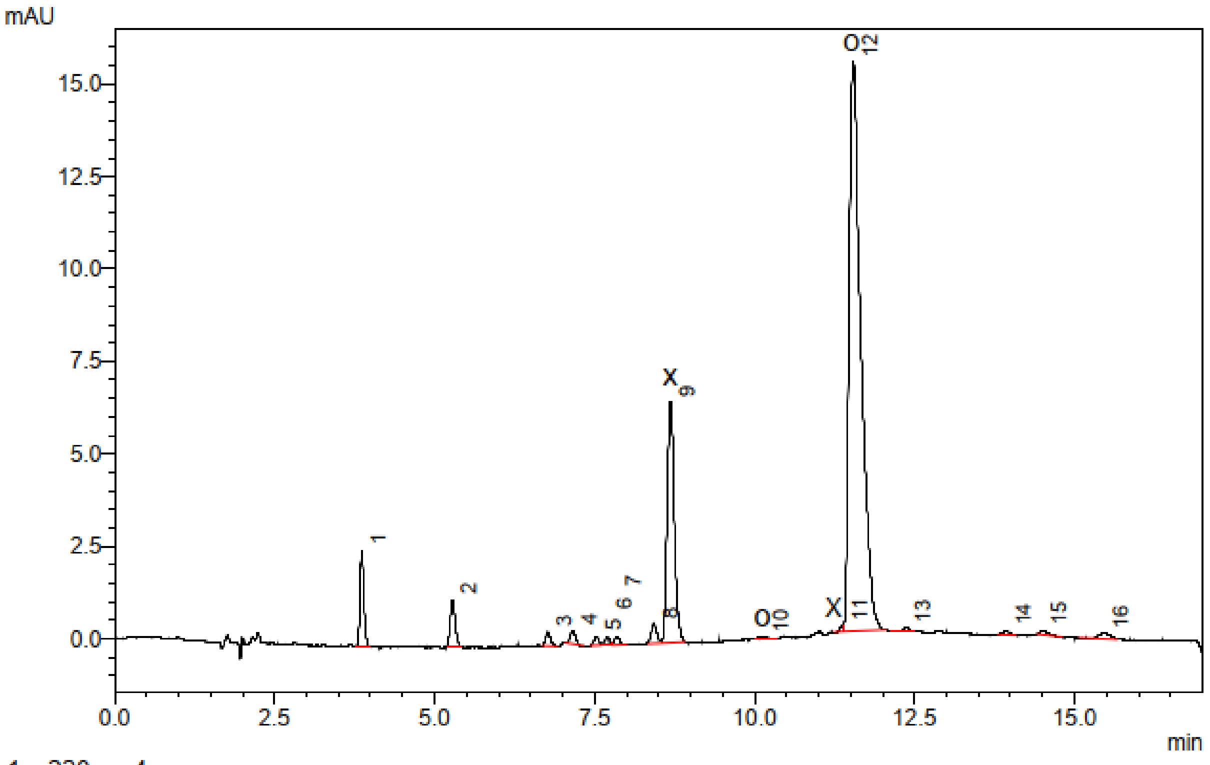

1 220nm,4nm

PDA Ch1 220nm

Peak #	Ret. Time	Area %	Name
1	3.86	3.76	
2	5.28	2.39	
3	6.77	0.67	
4	7.16	0.67	
5	7.53	0.47	
6	7.70	0.38	
7	7.85	0.44	
8	8.43	1.13	
9	8.68	17.80	1st enantiomer 1st diastereomer
10	10.14	0.09	1st enantiomer 2nd diastereomer
11	11.38	0.25	2nd enantiomer 1st diastereomer
12	11.54	70.43	2nd enantiomer 2nd diastereomer
13	12.37	0.18	
14	13.94	0.31	
15	14.50	0.39	
16	15.46	0.63	
Total		100.00	

X = 97.2 % ee
O = 99.7 % ee

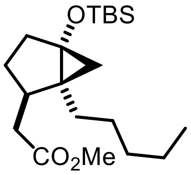

HPLC data of *rac*-**10**

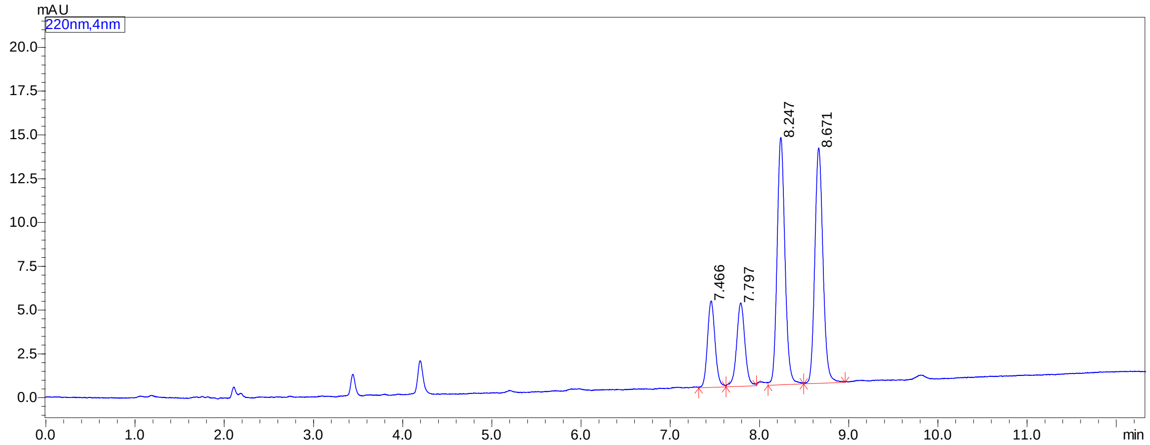

Peak	Ret. Time	Area%
1	7.466	12.496
2	7.797	13.115
3	8.247	37.414
4	8.671	36.975
Total		100.000

HPLC data of (1*S*,2*R*,5*R*)-**10**

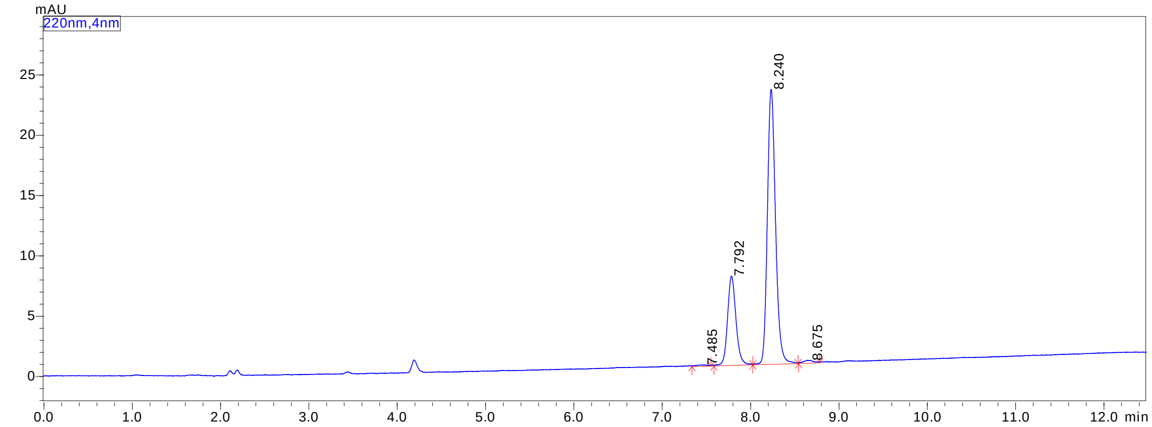

Peak	Ret. Time	Area%
1	7.485	0.148
2	7.792	24.673
3	8.240	74.619
4	8.675	0.560
Total		100.000

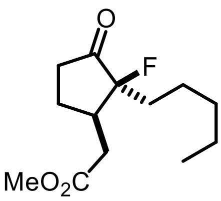

GC data of *rac*-**11**

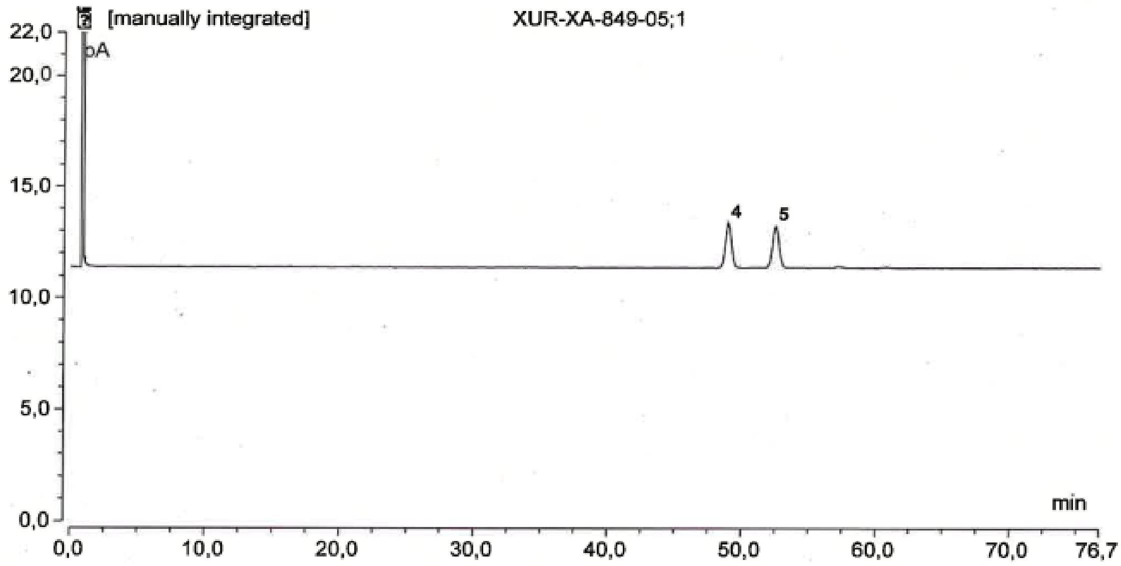

Sample:	**XUR-XA-849-05;1**	Instrument:	**GC_412**
Sequenz:	**12574 XUR-XA PH**	Measured:	**18.07.25 07:18**
Sequenz date:	**17.07.25**	Processing M.:	**XUR-XA-849**
		Report-File:	**XUR-XA-849**

Razemat
Zuordnung nach achiraler Messung

**No.**	**Ret.Time** min	**Rel.Area** %	**Peak Name**
4	48,96	50,08	.
5	52,47	49,92	.

Instrument parameters:

Column:	24,0 m	Cyclodextrin-H 0,25/0,125df G/632	
Temperature:	220 / 110 iso / 350		
Gas:	0,50 bar	Hydrogen	
Sample size:	0,2 µL	Split ratio:	60 : 1

GC data of (1*R*,2*R*)-**11**

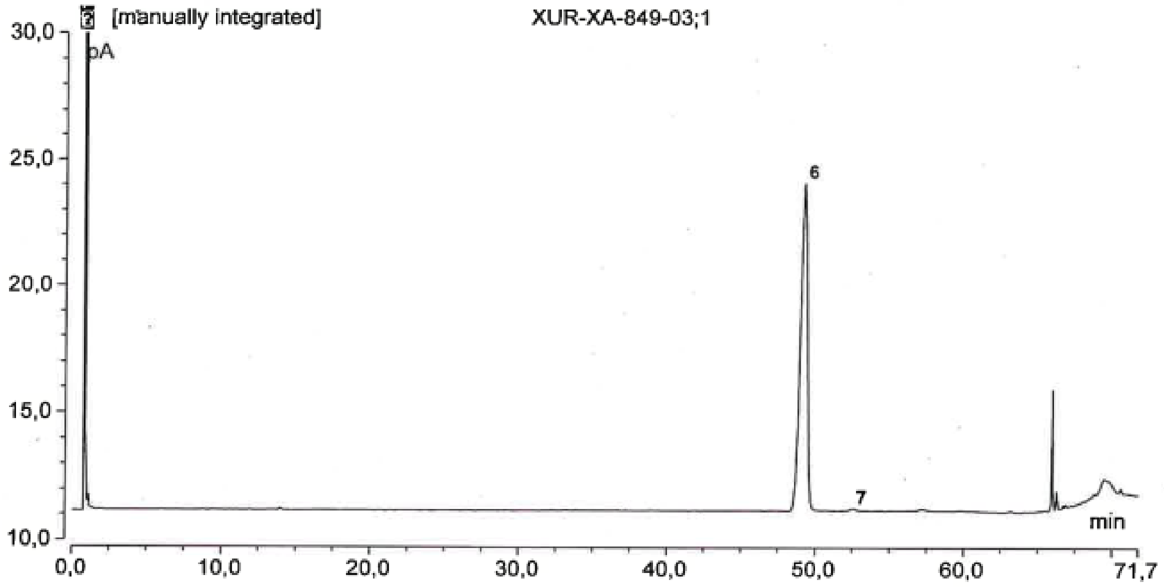

Sample:	**XUR-XA-849-03;1**	Instrument:	**GC_412**
Sequenz:	**12558 XUR-XA PH**	Measured:	**22.07.25 07:05**
Sequenz date:	**21.07.25**	Processing M.:	**XUR-XA-849**
		Report-File:	**XUR-XA-849**

Enantiomerenverhältnis
Zuordnung nach Razemat (XUR-XA-849-05) 25/12574

**No.**	**Ret.Time** min	**Rel.Area** %	**Peak Name**
6	49,17	99,37 .	
7	52,56	0,63 .	

Instrument parameters:

Column:	24,0 m	Cyclodextrin-H 0,25/0,125df G/632	
Temperature:	220 / 110, 60 min iso 8/min 180, 3 min iso / 350		
Gas:	0,50 bar	Hydrogen	
Sample size:	1,0 µL	Split ratio:	40 : 1

7. NMR Study

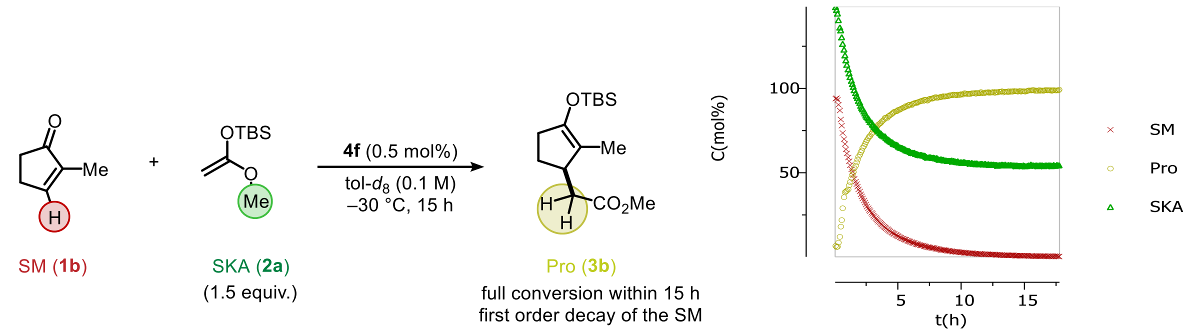

Scheme S1. NMR study.

A flame-dried Schlenk flask was charged with the corresponding catalyst **4f** (0.49 mg, 0.005 equiv.), dry toluene-d_8 (0.6 mL, 0.1 M), and the freshly distilled corresponding enone **1b** (5.89 µL, 0.06 mmol, 1.0 equiv.) under Ar. The mixture was cooled down to –78 °C in a dry ice bath and the corresponding SKA **2a** (19.6 µL, 1.5 equiv.) was added. The sample was placed inside to a precooled to –30 °C NMR machine. ^{1}H NMR was recorded every 30 min for 15 h, signals attributed to **1b**, **2a** and **3b** were observed. Selected spectra were stacked and presented in the following (Fig. S3).

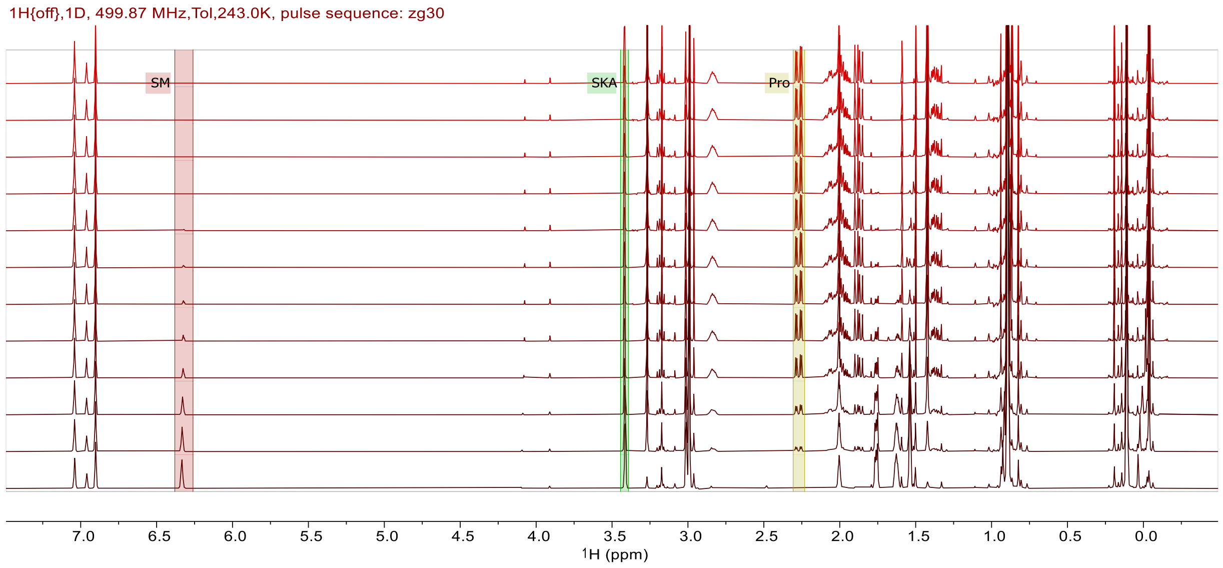

Figure S3. Stacked ^{1}H NMR, toluene-d_8, –50°C, SM **1b**, SKA **2a**, Pro **3b**.

8. Computational Studies

Computational details

Chiral ion-pair (R = C_5H_{11}, R = CH_3)

All calculations were run with ORCA 6.0.[7] For the chiral ion pair, an initial conformational sampling was conducted with CREST[8] at the GFN-FF,[16] GFN1-xTB[17] and GFN2-xTB level.[9] The exploration of the chemical space generated a vast number of conformers. Thus, we selected only some of them using an in-house code that performs iterative screening of molecular conformers by comparing root mean squared deviation (RMSD) values between each conformer and a reference conformer. For each iteration, the conformers wih the lowest energy and a high RMSD compared to the previous reference conformer was selected. After this, the so-selected conformers were reoptimized at PBE[10]-D3[11](BJ)[12]/def2-SVP(-f)[13]+CPCM(toluene)[14] (level for R = C_5H_{11}) and at PBE[10]-D3[11](BJ)[12]/def2-TZVP(-f)[13]+CPCM(toluene)[14] (level for R = CH_3). Electronic energies were refined with ωB97X[15]-D3[11](BJ)[12]def2-QZVP[13]+CPCM(toluene)[14] to identify the conformer lying lowest in energy.

Mechanistic studies for regioselectivity of cyclic versus linear ketones

Geometry optimizations of all intermediates and transition states of the catalytic cycle were computed at *ω*B97X-D3BJ/def2-TZVP(-f) + CPCM(toluene) level. Electronic energies were refined at *ω*B97X-D3BJ/def2-QZVP + CPCM(toluene) level.

Regioselectivity: linear versus cyclic ketones

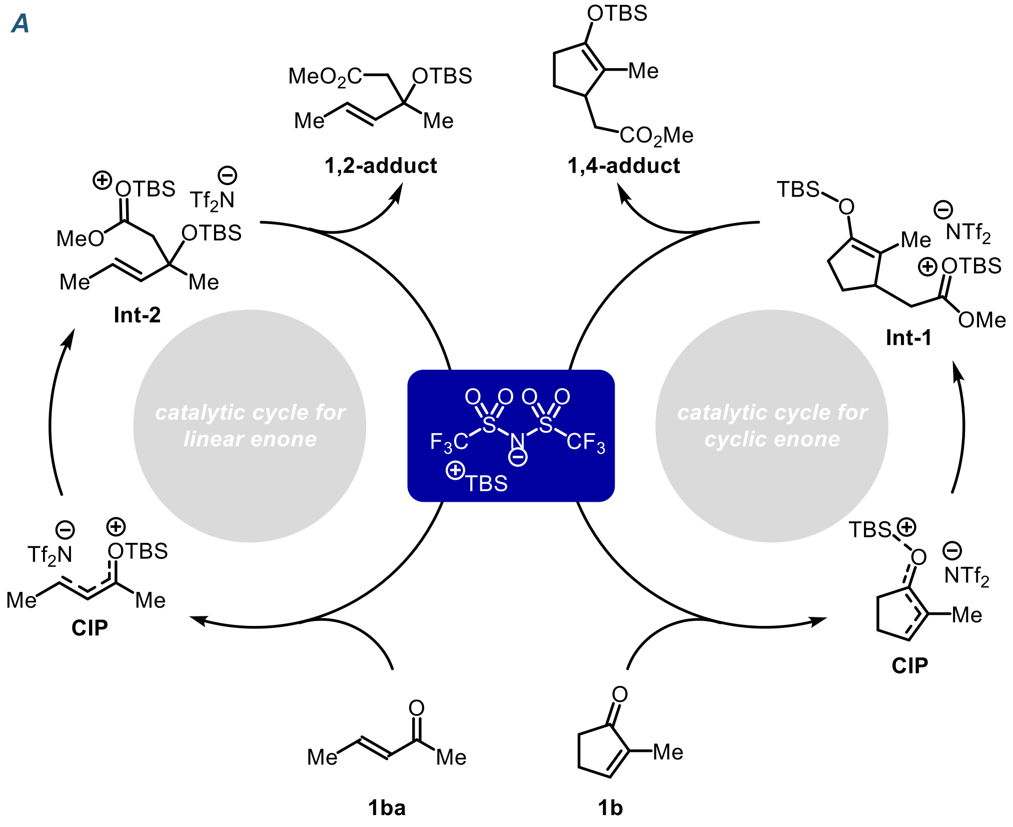

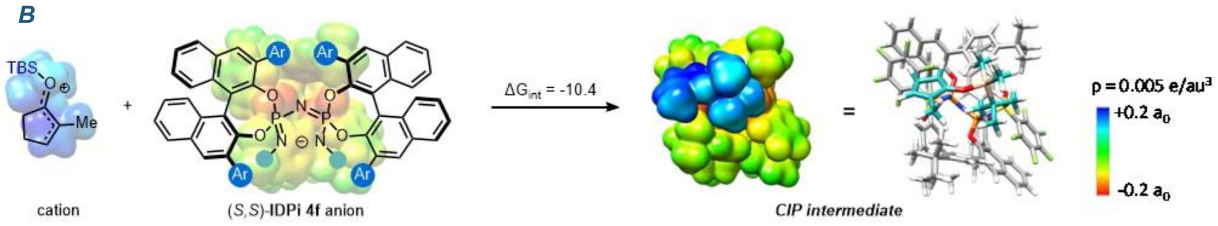

Figure S4. A) Catalytic cycle related to the formation of 1,2- and 1,4-cyclic ketones using Tf_2NH as precursor for the ion-pair. B) Molecular electrostatic potential of the chiral ion-pair and its components (isovalue = 0.005 a.u.). For clarity, the structure of the chiral-ion pair is also shown, with the carbon atoms of the cation highlighted in cyan and those of the anion in grey.

The proposed catalytic cycle relative to cyclic 1,2- and 1,4-enones is shown in Fig. S4A. Two kinds of substrate were investigated for such reactions: cyclic and linear. Experimentally, an inversion in the ratio between the 1,2- and 1,4- isomers was observed when using the linear and the cyclic variants. When dealing with the cyclic enones, the 1,4- and 1,2- isomers is 20:1, while the ratio is inverted with the linear substrates. The computational results closely replicate the experimentally observed 20:1 ratio between the 1,4- and 1,2-isomers of cycloenone, indicating that the formation of the 1,4-isomer is highly favored both kinetically and thermodynamically.

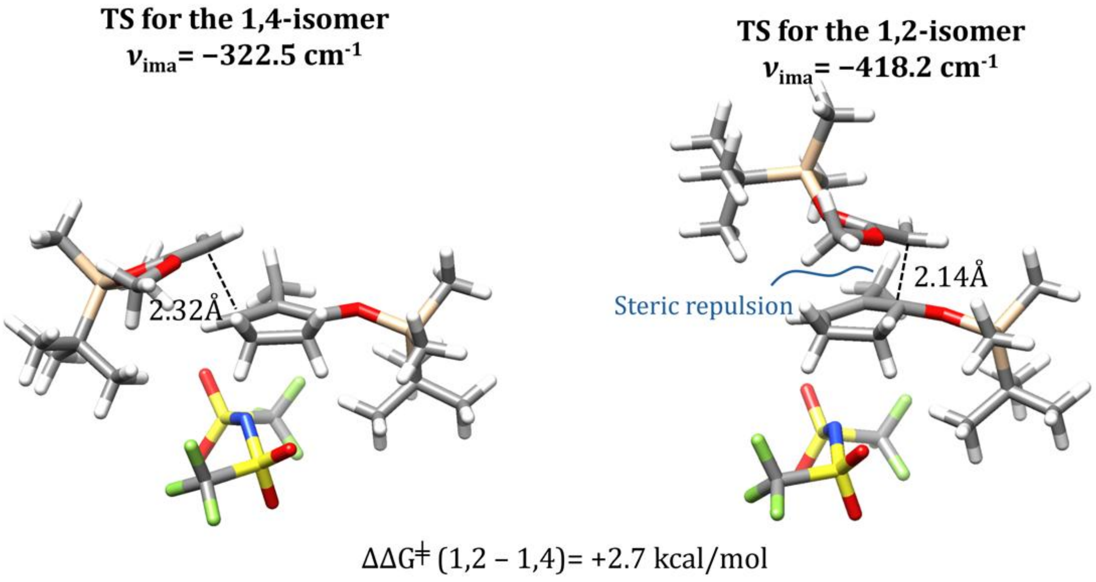

Figure S5. Transition states of the cyclic enones for the 1,4- and 1,2-isomers.

Figure S5 illustrates that, in cycloenone, steric repulsion between the ring and the nucleophile destabilizes the transition state leading to the 1,2-isomer, which is the primary factor governing the observed regioselectivity. In Fig. S4A the reaction for the linear variant is reported. The catalytic mechanism occurs in the same fashion as the cyclic variant.

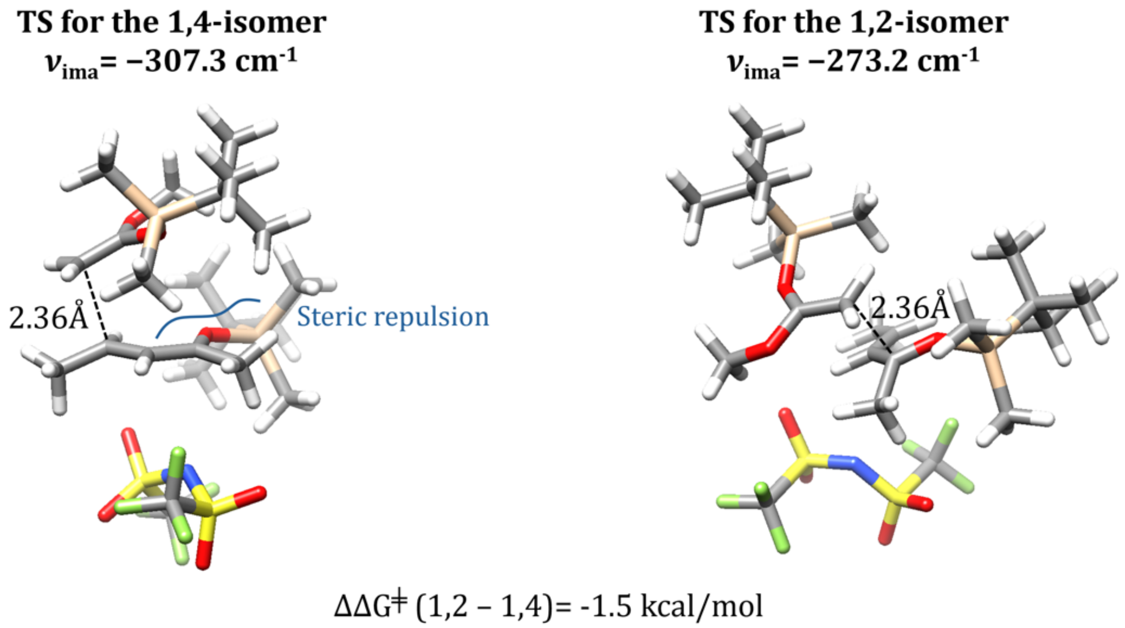

Figure S6. Transition states of the linear enones for the 1,4- and 1,2-isomers.

In the case of linear enones, the steric repulsion in the rate-determining transition state is markedly lower for the 1,2-attack compared to the 1,4-attack, thereby favoring the formation of the 1,2-isomer (Fig. S6).

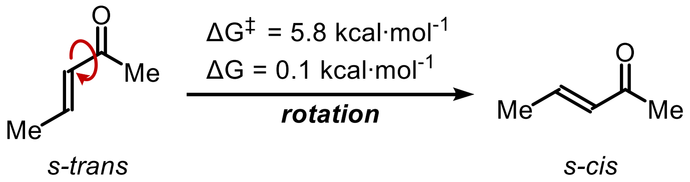

Scheme S2. Energetics of the rotational barrier for linear enone substrate.

The rotation of the linear enone substrate in solution unlocks reaction pathways that favor kinetically the 1,2-attack (Scheme S2), which are not accessible in the cyclic enones case for the rigid nature of the ring. Both **TS**s of the 1,2-isomer are more stable than the 1,4-isomer **TS**s. Moreover, for both the 1,2-isomer and 1,4-isomer, *s-cis* generates more stable **TS**s than *s-trans*.

CIP association process

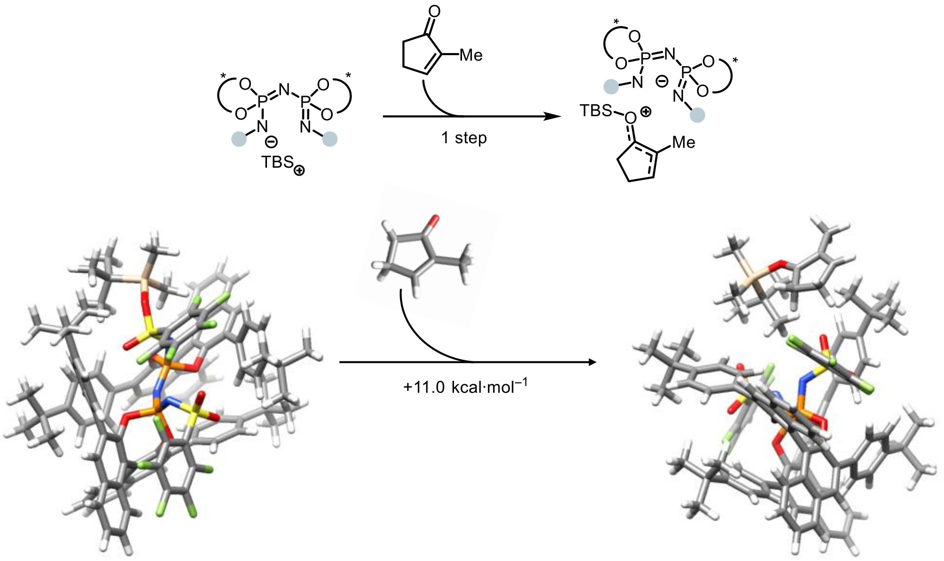

Figure S7. The chiral ion-pair formation of the enone substrate (R = CH_3) and IDPi catalyst **4f**.

Inversion of enantioselectivity

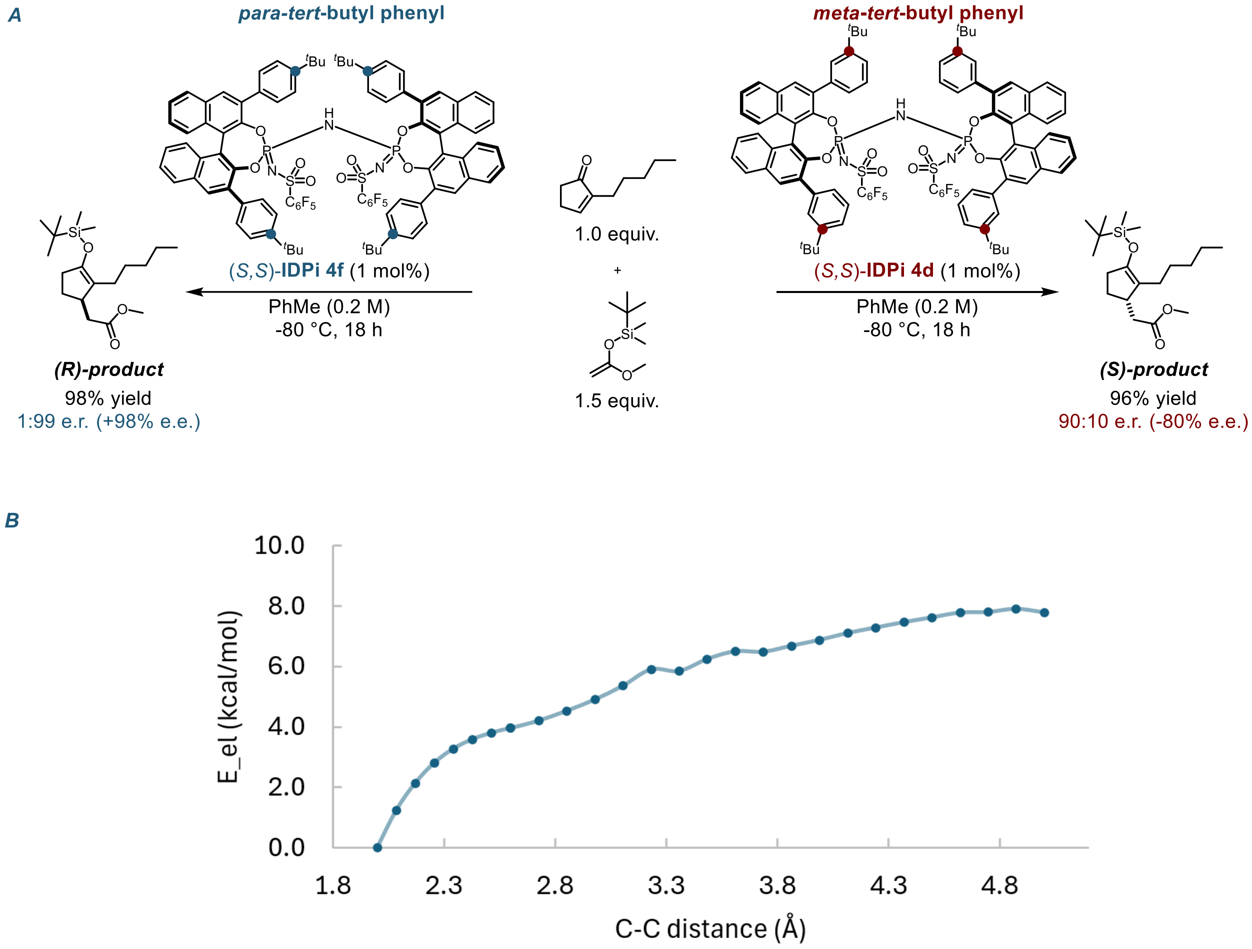

C

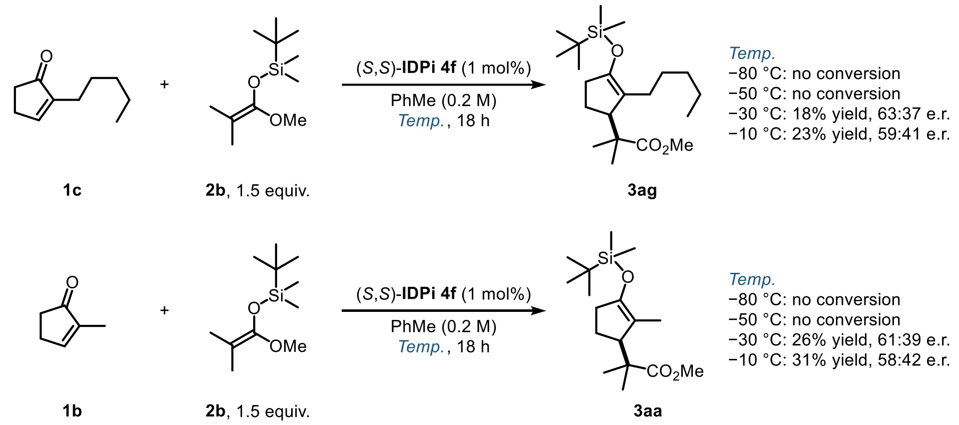

Figure S8: A) The experimentally observed enantioselectivity of the *para*-*tert*-butyl (**IDPi 4f**) and *meta*-*tert*-butyl (**IDPi 4d**) catalysts. B) Electronic energy (E_el) as a function of the forming C–C bond between the CIP associated with **IDPi 4d** and the substrate, obtained from a relaxed PES scan at the PBE-D3(BJ)/def2-SVP level of theory. C) Reactivity and enantioselectivity of **3ag** and **3aa** with sterically congested nucleophile **2b** under **IDPi 4f** catalysis.

To probe the origins of the experimentally observed enantioselectivity (Fig. S8A) computationally, several transition state searches were attempted. However, many did not lead to convergence as the C–C bond forming step was often found to be barrierless. This can be clearly seen in the relaxed potential energy surface (PES) scan (Fig. S8B). Note that although there are some apparent minima, e.g. at 3.36 Å, they are extremely shallow with walls lower than the available thermal energy ($RT_{-80\ °C}$ = 0.38 kcal/mol). Additionally, we examined a less reactive and more sterically congested nucleophile, β,β-dimethyl TBS-SKA (**2b**). Compared with the corresponding TBS-SKA (**2a**), **2b** shows markedly reduced reactivities and remarkable erosion of enantioselectivities (Fig. S8C). These results suggest that increased steric hindrance on the nucleophile diminishes both conversion and enantioselectivity. This trend is consistent with our mechanistic picture that high selectivity arises from a well-defined, tight ion-pair approach. When steric bulk disrupts effective preorganization and increases the accessible conformational ensemble, stereo-differentiation is eroded.

As expected, the primary attraction between their anionic form and organic cations is electrostatic. For catalyst **4f** and catalyst **4d**, the significant electrostatic interactions can be seen in the electrostatic potential maps (Fig. S10). To further probe the noncovalent intermolecular interactions in the CIPs, we employed the Atomic Decomposition of London Dispersion (ADLD) method,[18,19] using standard (B3LYP-D4) parameters.

The steric maps in Fig. S9 were made using Cavallo's web tool, keeping the substrate orientation constant so that the any differences in the maps reflect differences in the orientation of the catalyst. The following settings were used: bonds scaled by 1.17, sphere radius 8, distance from centre 0, mesh interval for integration 0.05, and hydrogen atoms were included.

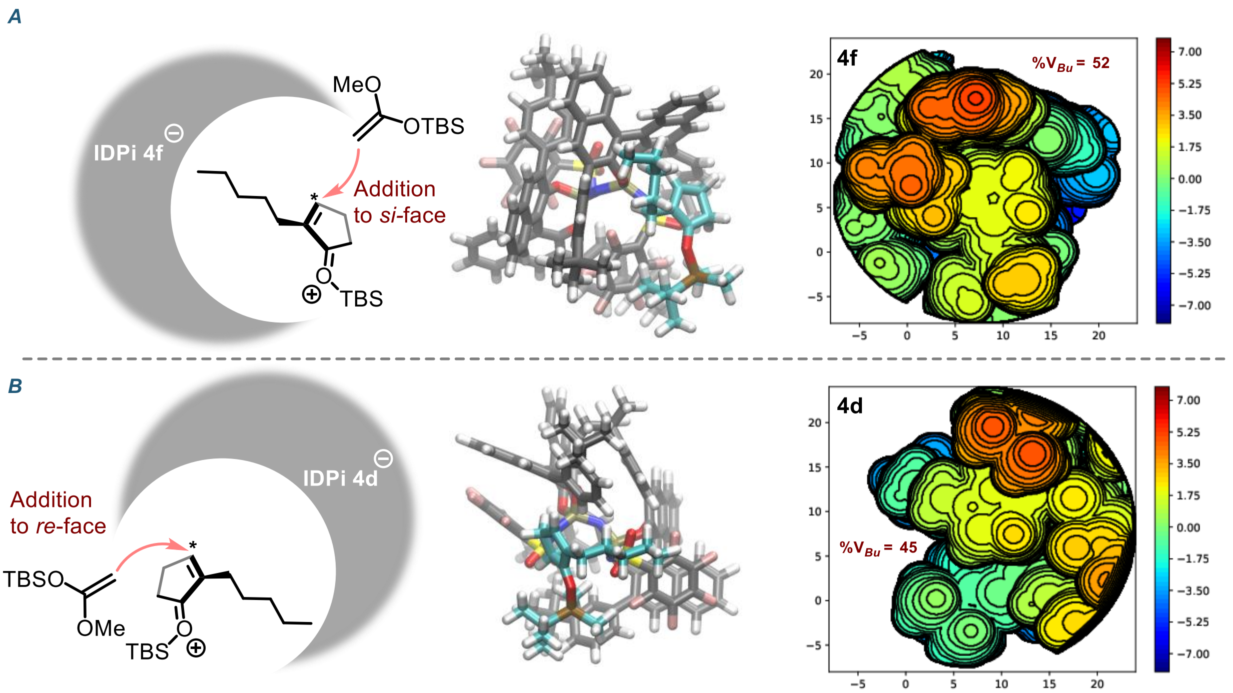

Figure S9: The CIPs of **IDPi 4f** (A) and **IDPi 4d** (B): schematic representation (left), optimized structure (center), and steric maps (right). The reactive carbon, marked by an asterisk (*), is placed at the origin of the steric maps. In the optimized structure, the ketone substrate is shown in cyan.

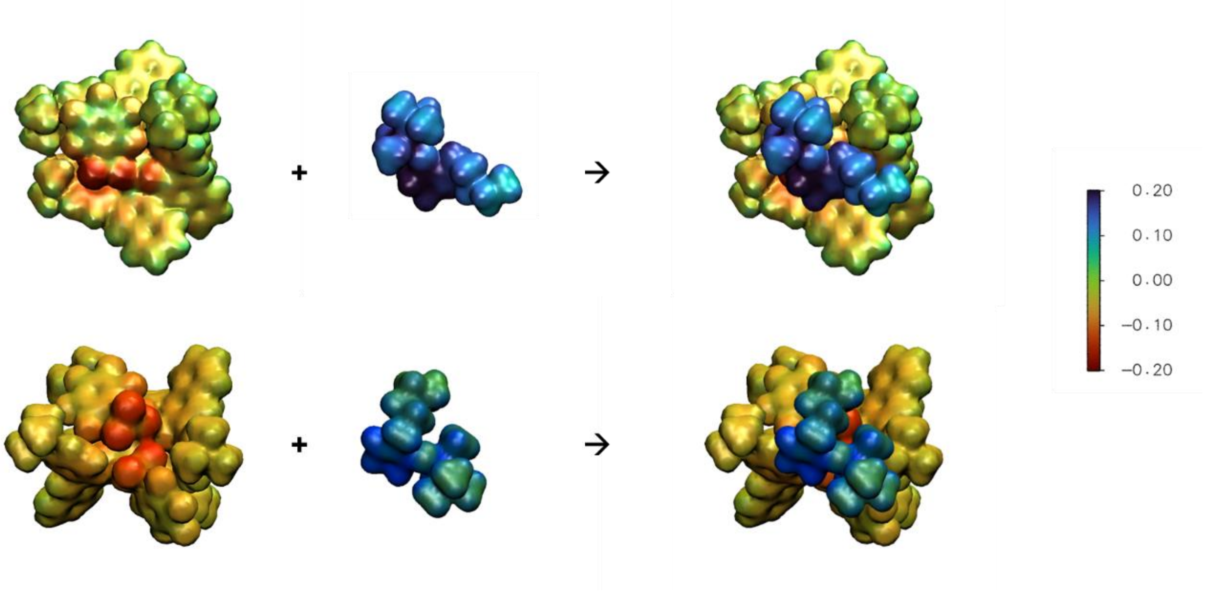

Figure S10: Electron density isosurface (0.01 a.u.) coloured with electrostatic potential for the chiral ion pairs of catalyst **4f** (top) and catalyst **4d** (bottom). Calculated with ωB97X-D4/def2-TZVP//PBE-D3(BJ)/def2-SVP.

Cartesian Coordinates and Electronic Energies

Cartesian coordinates (in Å) and electronic energies (in a. u.) of all stationary points discussed in the text.

Tf_2NH

C	-2.31247528401372	-2.00126200511446	-0.27629659328038
S	-0.49233853594101	-2.04210646641941	0.05829208336602
F	-2.67197543978380	-0.81605507176618	-0.74690870203962
F	-2.97388144437597	-2.25115373606294	0.85051849038355
F	-2.58504037838397	-2.94254812331401	-1.17191272857754
C	1.78551191876984	0.79427470739489	1.10702611781910
F	2.21378018405433	0.26697195369159	2.25086293673801
F	2.15447283754082	2.06759527862476	1.03739144482820
S	-0.06359925731582	0.73188165552981	1.05555163844752
F	2.30104835080753	0.12083866042708	0.08906270367564
O	-0.46145120082236	1.13976058924972	-0.24321897135040
O	-0.51028091263580	1.34225298307147	2.26034229818942
O	-0.23998792482850	-3.28684392635015	0.69912637303705
O	0.16204257242297	-1.60235732436549	-1.12065674126116
N	-0.34597339141080	-0.89018946067461	1.24019709352616
H	-0.44514072496473	-1.21929843218605	2.19502685734445

Cycloenone substrate

C	-1.51886110059097	0.80609812168272	0.02075042250618
C	-1.12629123344349	-0.67788518533797	0.02286231347651
C	0.37974395329759	-0.65627156341543	0.01162212653118
H	-1.51160118213271	-1.21238235318466	-0.85012691170916
H	-1.49825086996511	-1.20611991217592	0.90542056329164
C	0.90495255017869	0.57161979334358	0.00459223934351
C	-0.19943444880238	1.56352759818810	0.01008298486087
H	-2.09060716615472	1.09970185007661	0.90348848304557
H	-2.10110889383803	1.09407352360276	-0.85698563592277
C	2.33881290813425	0.98864603080955	-0.00680034468959
H	3.00249193313644	0.12259467268168	-0.00930244826344

H	2.56292563429839	1.60420243481131	0.86843101674330
H	2.55030612946863	1.59957406250673	-0.88839533875062
H	0.96513180207494	-1.57079299972283	0.00973536016048
O	-0.07108649616154	2.77103253423376	0.00648654827633

reagin

C	-1.65832888766229	0.64362374483985	-0.72830121010861
O	-0.20300781803628	0.91761341031371	1.07452542128846
C	-1.10945301461622	1.42715410196354	0.20274759838433
C	0.77445765947729	1.82815648058331	1.58710752888261
H	1.47214835034190	1.21660382679773	2.15720078648886
H	0.31820340435276	2.57469862749057	2.23837845517119
H	1.30480338336351	2.32815356284655	0.77194807880935
O	-1.39183964370020	2.71984246835509	0.42984187887639
Si	-2.02667733849125	3.85774786307563	-0.64749544609528
C	-3.86405817847998	3.58409987443161	-0.83358782871329
H	-4.08219199625471	2.61406808524891	-1.28509319828667
H	-4.29441998325313	4.35703620569587	-1.47667001002748
H	-4.36653785115996	3.62840787635686	0.13576695893959
C	-1.14489008035068	3.71837729460260	-2.28650304728884
C	-1.65815928528797	5.49457058144033	0.20632343390072
H	-1.45874223792318	2.83040334501357	-2.83768069578504
H	-0.06275919927471	3.66624258903733	-2.14357081506865
H	-1.36543443383107	4.59581169682571	-2.90128324406704
C	-2.24694429540149	5.48855733954362	1.62339274755584
C	-2.28465991722125	6.64115325801305	-0.59955713518526
C	-0.13936430785609	5.69870140916422	0.28769566076324
H	-3.37320976125842	6.55008077081791	-0.65218468392431
H	-1.89731822028866	6.67918800402642	-1.62213643928800
H	-2.05518446997671	7.60161902128256	-0.12375785308772
H	-3.33299216697549	5.35967820675895	1.60872227327659
H	-2.03250111232343	6.44112861358764	2.12216730589459
H	-1.81914486559027	4.68649300167125	2.23019862767682

H	0.34286631207567	4.88943194964259	0.84267516348084
H	0.08647266888063	6.64016283699218	0.80208203557587
H	0.31453713042760	5.74623506607025	-0.70619947749850
H	-1.32293030136081	-0.37885766492121	-0.82674829103673
H	-2.45303848998703	1.00592636674559	-1.36285675283389

reagout

C	-1.46429717944636	0.74846532832482	-0.85061344929859
O	-0.08079355301118	0.69264580704491	1.05274214582415
C	-0.73240083852567	1.51676949852396	0.21516037242425
C	0.64914503306804	1.34037425639146	2.10856760334168
H	1.10797849920758	0.53794338145459	2.68235282769402
H	-0.02840476956250	1.92098874433525	2.73535390672509
H	1.41224194132388	1.99849896537522	1.69194034912088
O	-0.71655436192691	2.71968222275996	0.32626172290903
H	-1.98150195907424	1.44200625410713	-1.50988193950701
H	-2.17970173020969	0.06812668683054	-0.38459153450944
H	-0.75364995314296	0.14680038635215	-1.42057048062407

$Tf_2N^-TBS^+$

C	3.31705773839401	-1.02855605752144	0.54547368517666
S	1.48097208770572	-1.22354508980525	0.49061306766889
F	3.86752028200468	-2.18872336141543	0.19272610902275
F	3.71855330889144	-0.70660009432396	1.77097602907452
F	3.70751006535929	-0.08265614367389	-0.30668314518758
C	0.66537601552923	2.03338094306230	-1.11090339081324
F	1.70076285424416	2.76636690926529	-0.71883262496736
F	-0.28588360349449	2.82624105357522	-1.59489462350735
S	0.00082904195718	1.14384171270104	0.37323505948571
F	1.05065952496816	1.18020324628205	-2.04700990398647
O	-1.09636581014633	0.34377504157418	-0.30204177691572
O	-0.46664201852413	2.16002378226046	1.25595038435970
O	1.14879458903929	-1.49310942848797	-0.87593592123298
O	1.15331592833609	-2.16979652313880	1.51058768006547

N	1.08387734318332	0.26116424180516	0.98076729399858
Si	-2.67583378664660	-0.08679670015710	0.39369666065716
C	-3.68305132472673	1.46483010420051	0.28229520427700
H	-4.69046005346262	1.28149397534429	0.66600681437439
H	-3.76557461372750	1.81059755233530	-0.75035847683359
H	-3.22703960213333	2.25591719866434	0.88117495713073
C	-2.32287737145855	-0.62073648141129	2.13378322152444
C	-3.22899912431986	-1.46072967212445	-0.74471355398811
H	-2.01203149036892	0.22755533703188	2.74750170568995
H	-1.54006207360332	-1.38173131196157	2.16642100393788
H	-3.23024042821425	-1.04425999825749	2.57388264852309
C	-4.66710363218736	-1.83301761705397	-0.34660248467466
C	-2.31463741229623	-2.68225460614264	-0.58146261068651
C	-3.20455943017864	-0.98539969497720	-2.20366115804613
H	-1.28010126547603	-2.45089946921531	-0.84469060052179
H	-2.65750226069342	-3.48647084488120	-1.24157253834907
H	-2.32855852082599	-3.06392113299666	0.44289418051309
H	-4.72765322451399	-2.16996797921638	0.69279139723923
H	-5.02112537049196	-2.65319737926016	-0.98009589455884
H	-5.35556611955130	-0.99288385841547	-0.47557579210533
H	-2.19317448696878	-0.72093838981290	-2.52281353527136
H	-3.85240218469065	-0.11825087150604	-2.35957710025333
H	-3.56175668071062	-1.78885583614511	-2.85689307261974

CIP of cycloenone

C	-2.37352748924642	1.85676830135790	0.92177228601634
S	-2.73846707538518	0.04698141034395	0.84635863365028
F	-2.19333600811405	2.36719373259777	-0.29603096826207
F	-1.27039855943861	2.09079270270652	1.64673227126320
F	-3.39812161484889	2.48663059790077	1.50226658574699
C	-1.81983019975311	-2.54376244720492	-1.51565227209527
F	-1.30853429869041	-3.40655215451812	-0.62359921051905
F	-1.48616011813917	-2.96492307666789	-2.74054005786899

S	-1.12323976202010	-0.85109856797617	-1.24813713476824
F	-3.14342950508363	-2.55869168737366	-1.40334362785126
O	-1.79613936714391	-0.01816688389630	-2.20064423804159
O	0.29616485130606	-1.06836110036949	-1.40379658756418
O	-2.84390124625475	-0.33359019074531	2.22686642627811
O	-3.88494908823851	-0.05575366187464	-0.00784575239925
N	-1.38991857272672	-0.56270598583348	0.27508822279670
C	2.64272414917018	-2.37366501965851	-0.02433870497343
C	1.87610927374534	-3.59942615545714	0.48296099476389
C	1.07378421604013	-3.07198222852005	1.62573698341368
C	1.22157763491770	-1.74629041521896	1.83448396516094
C	2.11569828264842	-1.26459916230971	0.82148419985165
C	0.60334598382931	-0.86822670653300	2.87138808365884
H	1.36283709227150	-0.52880302133038	3.58080989812895
H	-0.17666204727173	-1.40253794444051	3.41231070167384
H	0.15625432511895	0.00725589256948	2.40241445896007
O	2.40985431089270	-0.03716515396765	0.72775019717161
H	2.46674301214170	-2.16255217647009	-1.07679537410054
H	3.72315096046696	-2.45215283181715	0.13363381437190
H	2.53285498759753	-4.41070697394913	0.80706703682870
H	1.21313151877052	-4.00619267302983	-0.28479092031394
Si	3.41076995916527	0.91646570951189	-0.36009854395788
C	3.42892272609748	0.14648158154277	-2.04884323185889
H	4.07146817033912	-0.73482031421720	-2.09434697551971
H	3.82933607598574	0.88544712585047	-2.74961990246678
H	2.42263969988934	-0.12026185265712	-2.37619448742913
C	5.07621745489415	0.88161397882807	0.46359525947457
C	2.54541940550903	2.57174858076408	-0.30939046801011
H	5.02834076034999	1.28418527187061	1.47752341053189
H	5.78762241081086	1.48081593091592	-0.11148232110220
H	5.46104842000790	-0.14065466305300	0.51173573762677
C	1.15630968491372	2.43468669295799	-0.94792438102032

C	3.38823635313181	3.58075224699301	-1.10503053053141
C	2.40011636228499	3.05546765274947	1.13997405201874
H	4.38388898839565	3.71185345480997	-0.67032855290357
H	2.89236198613867	4.55719203344369	-1.09839632800956
H	3.50667101992923	3.27991098210091	-2.14996900409790
H	1.21639555526587	2.12395133891196	-1.99409920133108
H	0.63621942430488	3.39754193247320	-0.91235744298535
H	0.54186053343423	1.70642921043293	-0.41579354304173
H	1.89859639275149	4.02909746627750	1.15135783223765
H	3.37159690228768	3.17627033183821	1.62800732717919
H	1.79839363968099	2.36498078219769	1.73584424850542
H	0.42301542006957	-3.70651779195734	2.21813461611339

TS 1,2 cyclic

C	4.20339847209283	-1.64481541677521	2.47248709846464
O	4.82064842570052	-3.48203748494736	1.20634947242414
C	4.26962038828556	-3.01660973656833	2.31492832706131
C	4.82585479657381	-4.90665074066410	0.98265852195270
H	5.36304233403248	-5.41126505650520	1.78495749160102
H	5.34107843381546	-5.03903473749358	0.03490961281898
H	3.80560685916155	-5.28399258751788	0.91649048687769
O	3.73593343350432	-3.89905770466338	3.11142496114309
Si	3.85216925113350	-4.10962275157636	4.81890490798272
C	3.00383345631295	-2.72155430386647	5.71566049525359
H	3.54187870082378	-1.77756047611982	5.61180965742528
H	2.97099038792626	-2.96432019976946	6.78184482125883
H	1.98006923667398	-2.58326932647755	5.36652821794237
C	5.67783846356536	-4.13576462824582	5.17871556492686
C	2.98782843367111	-5.75305407323726	5.07351917062953
H	6.13304581280204	-3.18110223374592	4.89989501562954
H	6.18432778259267	-4.93311376124608	4.63021301542592
H	5.85185126076653	-4.28902707255994	6.24716084402168
C	1.52847842275351	-5.64540888333730	4.60911076196126

C	3.02972820774007	-6.09565550991005	6.57074553217343
C	3.69555090243304	-6.85892850760678	4.27964033452192
H	2.50320057048846	-5.35085854790194	7.17366853563465
H	4.05667842945128	-6.17148575720044	6.94117806448880
H	2.54422001045370	-7.06244434774780	6.74223129304428
H	0.98056543168637	-4.88020566507299	5.16568741622934
H	1.01691766945748	-6.60126453104930	4.76665374355790
H	1.46266860156401	-5.40293140362562	3.54508135865683
H	3.67007851511411	-6.65975626375653	3.20566078164566
H	3.19353025741749	-7.81718124632355	4.45251041717800
H	4.74002948334206	-6.97273824167999	4.58335416673792
H	4.86611099740502	-1.07059018011325	1.84120706095550
H	3.98760670964266	-1.25640800366383	3.45645056684552
C	-2.31235897075945	0.37204797852971	0.87621308086523
S	-2.33903836570868	-1.36846285593749	0.26023494551750
F	-2.32509076000648	1.24170531333695	-0.13398960326667
F	-1.21824876365282	0.59938272201467	1.61722542797650
F	-3.38643811612843	0.58964093567127	1.64149894005350
C	-1.16978543516229	-2.98642288293134	-2.76098127506232
F	-0.29501038825781	-3.85154857868857	-2.22832643992112
F	-1.00941953505586	-2.99164990847038	-4.08806845991937
S	-0.86328671866706	-1.28182738713964	-2.11443493376415
F	-2.40122360997770	-3.40709951202526	-2.47793111314536
O	-1.85137553799922	-0.45720232826959	-2.74515267887022
O	0.52684831434326	-1.06726942872933	-2.42543599460170
O	-2.20455169769179	-2.15282340424954	1.45767759783742
O	-3.54796509506262	-1.46060288916583	-0.50494907309678
N	-0.98468949995141	-1.46467259972739	-0.55079998335758
C	2.15332859381938	-2.02316372388159	0.42946806925768
C	1.31949959269430	-3.14726908594966	1.06713516755522
C	0.84323166021311	-2.54801422248878	2.35808560383929
C	1.41778115860759	-1.37325615607822	2.63524367686392

C	2.36620291811286	-1.04619266967739	1.55781880356817
C	1.15339949034040	-0.42897076819811	3.76119924504329
H	2.06997342498556	-0.19194242032569	4.30772654851164
H	0.42757945411266	-0.85078704477735	4.45695512017063
H	0.75664447324517	0.51173228124216	3.37311285467423
O	2.65281695215424	0.22169952788726	1.37429855777799
H	1.54691208027170	-1.50602179877381	-0.31643266493156
H	3.07130327589575	-2.35172526870860	-0.05085724640847
H	1.89808724231539	-4.05674564052184	1.25010727728510
H	0.47709141392440	-3.42001933640811	0.43071048228348
Si	3.45576156235224	1.13533814433749	0.16519785527746
C	3.88203804189626	0.09052471931309	-1.31425181371427
H	4.54421870245532	-0.73529075662817	-1.04213275184295
H	4.41882952962040	0.72250431611557	-2.02873626113838
H	2.99867566864701	-0.31117662972782	-1.81280626737272
C	5.00396645581910	1.75779556326435	1.00191191984231
C	2.24917984301256	2.51746257525728	-0.22054154251943
H	4.77323169378954	2.21626780219581	1.96630494503650
H	5.48712932264100	2.51107910280250	0.37347555017146
H	5.72523327678374	0.95344792859192	1.16537397022112
C	0.99774689757212	1.92844376690264	-0.88327223215660
C	2.91842876897444	3.51201803140511	-1.18036201439796
C	1.85321898039712	3.24011552634367	1.07418297805527
H	3.79926891179633	3.98180202700959	-0.73286057421518
H	2.21162714043916	4.30897048417887	-1.43665766989664
H	3.22365406834938	3.03087088114591	-2.11433740768764
H	1.22543800455423	1.46555180353215	-1.84515580695710
H	0.25937325090902	2.71908179299303	-1.05653666779585
H	0.52741778108755	1.16934727070953	-0.25495163611969
H	1.15903003411660	4.05609767547669	0.84405700893255
H	2.72083451894468	3.67395366321213	1.58032368848806
H	1.35298713349592	2.56320551312513	1.77149236800034

H	0.07852072300351	-3.01184766814213	2.96910371257911

TS 1,4 cyclic

C	3.61698882493305	-4.79550481125961	4.80769952061376
O	3.85019991289250	-6.75557334644812	3.58613126697327
C	3.12946324165874	-6.00818463015575	4.42609280022150
C	3.23899373415005	-7.94186685849650	3.04331841369993
H	2.98972328843458	-8.64124736350229	3.84091041045041
H	3.99255792690287	-8.37013078896936	2.38645289279294
H	2.34313936522223	-7.68645862723303	2.47686635090094
O	1.93043315271622	-6.47279369758653	4.70248091729329
Si	1.01029430161609	-6.41409123575710	6.15103400277403
C	0.68129722018560	-4.65441419148576	6.66153294442168
H	1.55418699434420	-4.19339477382077	7.12862918788260
H	-0.12454435513211	-4.65311709421594	7.40175017354404
H	0.35610717163551	-4.04076367914205	5.81986559278134
C	2.02930421194193	-7.30706795869227	7.42975492473984
C	-0.55616564969332	-7.31474255865033	5.65165945479958
H	2.99160544475239	-6.80481771358455	7.56408490600416
H	2.21834797031684	-8.34356287106706	7.14131970488323
H	1.51395379780909	-7.30601624608131	8.39415090539584
C	-1.27561226416330	-6.51301886551025	4.55755246864926
C	-1.46654532181144	-7.44215583451545	6.88198600293926
C	-0.21781311572196	-8.71485663956747	5.12266122481575
H	-1.75055869245910	-6.46424871053905	7.28049014203553
H	-0.98816703458012	-8.01294013790908	7.68401783588738
H	-2.38900726174006	-7.96539919372192	6.60700643988659
H	-1.56796118951154	-5.51949060116033	4.90401541904317
H	-2.18371247329574	-7.04036510165339	4.24498629938215
H	-0.64631124541993	-6.38432584414702	3.67291113827578
H	0.41420004677001	-8.66336352064461	4.23273846078322
H	-1.14005362826782	-9.23872634901513	4.84744697669300
H	0.29647529286880	-9.32107198241701	5.87430098101046

H	4.63058348950117	-4.53646178404296	4.53688582305764
H	3.12203023472529	-4.24234233214987	5.58962320127576
C	-1.42583833710264	-1.09430974875296	2.39979763137782
S	-1.36125449628803	-2.92263285479454	2.65967304792702
F	-1.74777123435234	-0.78975454581149	1.14354745713363
F	-0.23939469682183	-0.54031067325487	2.67970423715972
F	-2.33962430042328	-0.56485672094123	3.21849693887443
C	-0.78753730374999	-5.68584023230656	0.35211413417240
F	0.28270455067129	-6.24154478250982	0.94468477894114
F	-0.93372485603890	-6.24343777521781	-0.85250001586081
S	-0.54681341527529	-3.86224824847683	0.15992547315423
F	-1.86232472954832	-5.96471421332735	1.08639343105668
O	-1.76322558990037	-3.38957367788636	-0.43109170718837
O	0.68068150891283	-3.77034534731610	-0.58581495850420
O	-0.82075111816967	-3.04527335595050	3.98865186394518
O	-2.69565346613195	-3.38050714515237	2.40725990604122
N	-0.24871172715935	-3.39024996554739	1.63975227924532
C	3.75300235037513	-2.98192637321400	1.30316480656552
C	2.99459877401972	-4.19623131319097	1.84948154414844
C	2.41046474168795	-3.72133584208850	3.14362281517398
C	2.71133785221137	-2.37026657573358	3.37492568123672
C	3.46318388904663	-1.91498650513974	2.31354013978683
C	2.26195352937637	-1.55435890448321	4.54419081832987
H	2.90466915838365	-1.73786783581665	5.41120771471550
H	1.23767428784931	-1.81040950951039	4.81547639146947
H	2.30897458775360	-0.49045295414356	4.30920683042027
O	3.87098680828301	-0.67599738186241	2.21150890079692
H	3.38127004808611	-2.69663908735334	0.31798856522394
H	4.83206577277508	-3.13585521754770	1.22094603944132
H	3.63431907147150	-5.06815130262420	1.98404603573197
H	2.18393742048959	-4.48437136275945	1.18162776198530
Si	4.24669454662650	0.30737616486191	0.85342093630105

C	5.32853390893602	-0.61363394769776	-0.35497311799763
H	6.13166002422680	-1.14322815059111	0.16371524413780
H	5.79155825745514	0.11841171902756	-1.02358940947990
H	4.77735957199030	-1.32677538226166	-0.96961841281286
C	5.18997087714077	1.70428310451927	1.63928033330358
C	2.60918536204145	0.84585734103036	0.11103559060436
H	4.62647258695951	2.14524113030085	2.46427463850970
H	5.39303752493978	2.48787847798577	0.90426396709426
H	6.14755294823761	1.34663475901385	2.02688364377240
C	1.75086180780250	-0.37225001902488	-0.25538553209338
C	2.90396041442598	1.66108946469288	-1.15758044190862
C	1.85395005427266	1.72043228508456	1.12112815561966
H	3.52862728149458	2.53455062540346	-0.94551515548145
H	1.96527109137702	2.02282357049112	-1.59142730495365
H	3.40850396632021	1.05674296760791	-1.91687727868072
H	2.24753017246878	-1.02050191383000	-0.98239551510310
H	0.80848187072144	-0.04062292750909	-0.70464651646345
H	1.49255870190789	-0.97457325063250	0.61872825943699
H	0.87730646994343	2.00168925569880	0.71208119907972
H	2.40100659728228	2.64044279162653	1.34455938891950
H	1.67823325255825	1.19049391924657	2.06071147472048
H	1.54620023885879	-4.19846018918784	3.58322152706592

Intermediate 1,2 cyclic

C	4.01789562271195	-2.05475125625016	1.65537230610675
O	4.65260147636394	-4.21281979451047	0.96119678991542
C	4.22240984921239	-3.49634004288162	1.93538762626207
C	4.76823345740746	-5.65345899711269	1.14865898171401
H	5.45664469339247	-5.85750109488955	1.96637944932929
H	5.15374853691273	-6.02254419418905	0.20343583083249
H	3.77939209005937	-6.05797879321114	1.35777771239608
O	3.98210251225076	-4.05690413753071	3.04160896506562
Si	4.08516869261041	-3.70132247367321	4.77515102195436

C	3.62373404886102	-1.94188952480005	5.13706325525403
H	4.34752533679607	-1.23766284299136	4.72140580804328
H	3.64956517005539	-1.82737144483906	6.22575800370693
H	2.62361897455749	-1.66884003179975	4.79970552315233
C	5.88541973017416	-4.00633451299959	5.10844669641081
C	2.92399498730110	-4.97937686556078	5.48565127985301
H	6.50128215666845	-3.28134378939889	4.56914335971221
H	6.18740453333275	-5.01257885913613	4.81149005681355
H	6.08894144136433	-3.88776491273618	6.17642314640537
C	1.50061996596364	-4.75632102791855	4.95791483335437
C	2.93982837681924	-4.82132435067461	7.01543529214410
C	3.39862300017915	-6.39057610085214	5.11170686184639
H	2.56937504100568	-3.84071252564195	7.32619865252047
H	3.94339585525708	-4.95768800775406	7.43005045603643
H	2.29004574845990	-5.57803086116562	7.46681074762155
H	1.12165203162251	-3.76379969312997	5.21456717827649
H	0.82519185213644	-5.49511897080811	5.40173589427659
H	1.44946474838214	-4.86776279260979	3.87176714335679
H	3.41502233385745	-6.53694907967934	4.02835222066299
H	2.71328462249574	-7.13114665329213	5.53682382224113
H	4.39785331209442	-6.60012243403128	5.50276041696707
H	4.94098948262911	-1.65252975245873	1.23311967697766
H	3.78813773192848	-1.54516434420424	2.58552918868505
C	-2.91394405398895	1.42626537250172	0.16349444903961
S	-2.44932569010297	-0.28806079774663	0.68135894448285
F	-3.33781537400416	1.46615057666595	-1.09741285449095
F	-1.87497287240680	2.25842631779263	0.28897772356867
F	-3.90082230876610	1.86258456936625	0.95711420670912
C	-1.84703154559263	-3.12024698411436	-1.20832382783758
F	-1.67206158618496	-3.43582278078487	0.07988010218579
F	-1.12942784828998	-3.98243861132748	-1.94466824688171
S	-1.27809667645524	-1.36943101493584	-1.58487467852502

F	-3.12951831583073	-3.29082700709970	-1.51409832525345
O	-2.28102128007168	-0.86789029895930	-2.48067362860972
O	0.06377335910147	-1.53418647171958	-2.07539965894255
O	-2.03382407470852	-0.11322064398471	2.04702129529581
O	-3.61780806405703	-1.07697208562496	0.39776166793768
N	-1.17751085362031	-0.64427120513087	-0.18264648137255
C	3.15548402354165	-2.24278856449638	-0.79623000491897
C	2.43652085864132	-3.58571975939332	-1.02810896192627
C	1.50357591266177	-3.68226053357694	0.14683808919095
C	1.65479549335138	-2.69928682125460	1.02842393566467
C	2.83554312918346	-1.81454643621506	0.65424347375171
C	0.81035418703201	-2.39290535168506	2.22076023438067
H	1.40931445670963	-2.28500984904072	3.12777875944633
H	0.06893906171393	-3.17525472482090	2.38583583053296
H	0.28357150420791	-1.44995047898203	2.06199040297260
O	2.48046169723765	-0.45958689061232	0.76967941518088
H	2.71464269793206	-1.48517610808376	-1.44299530561624
H	4.22672137338317	-2.28675999423613	-0.99661797458148
H	3.13016005675023	-4.43067013852817	-1.07373087480789
H	1.87278927826701	-3.57975898191835	-1.96341503176119
Si	3.39581194726648	0.92715944108725	0.60620259917071
C	4.61259303537436	0.77903752272988	-0.81020456353694
H	5.32293652503688	-0.03687267644127	-0.65069310449461
H	5.18858896338448	1.70572652678931	-0.89057532810942
H	4.10634532259997	0.60828861824786	-1.76238790470479
C	4.36105211215323	1.22773447619847	2.19194219960371
C	2.13696189068033	2.29002667468518	0.32186173554013
H	3.72034129702134	1.10615110987698	3.06953399701989
H	4.75279917621419	2.24869776797315	2.20143283136120
H	5.21370161280275	0.55056061655539	2.28897760974462
C	1.35570849876555	1.99928544043519	-0.96692596535647
C	2.85387334524472	3.64063857473020	0.19105099201295

C	1.16914531203770	2.33693185169215	1.51244844417591
H	3.40178232013440	3.90295397500259	1.10099372159879
H	2.11928358384600	4.43336814473848	0.00869628689845
H	3.55943006595313	3.64677820890829	-0.64540921824325
H	2.01397041172951	1.98988373917762	-1.84109934387123
H	0.59914197824613	2.77525421133648	-1.12894428064306
H	0.84048029403911	1.03871528167283	-0.91438616840039
H	0.43959903538792	3.14143721739843	1.37110672035929
H	1.69701128459599	2.52988051380633	2.45189541581933
H	0.61393679895382	1.40148923856497	1.61393485281561
H	0.74127923003336	-4.44910761448854	0.22356859852948

intermediate 1,4 cyclic

C	3.54834040417504	-4.50900299108312	4.97342786376439
O	4.53921226801174	-6.60680630037255	4.57788364318277
C	3.51264567408524	-5.98838119081554	5.05480093913598
C	4.51073797726496	-8.06297553413348	4.52755104897878
H	4.41938436573286	-8.45630197506397	5.53818298709814
H	5.45974378166687	-8.34037584751633	4.07938081833962
H	3.67312360842054	-8.38148158557616	3.90942001739578
O	2.55632236659784	-6.67749310955537	5.49987835715027
Si	0.95377379449264	-6.47801892950013	6.25006788139110
C	0.64412589954888	-4.70331179664567	6.66504396289678
H	1.40616244091694	-4.30983678262847	7.34151033302205
H	-0.31213573327250	-4.67224385016313	7.19803905222686
H	0.54683309438342	-4.06031187399423	5.79184541924826
C	1.17561134932720	-7.50160304600731	7.78287546409661
C	-0.18768744000057	-7.23983354411977	4.97892867832232
H	1.86801836390738	-7.01350745936483	8.47347829143653
H	1.55309184121967	-8.49915816187828	7.55037727906998
H	0.21377120245601	-7.61129257376142	8.29230663744448
C	0.10397744780537	-6.71717218692411	3.56746967810039
C	-1.62425747069881	-6.85438441226554	5.37372410010651

C	-0.03523082294512	-8.76748282905387	5.00073810288890
H	-1.78498332926225	-5.77686693800481	5.28984298047962
H	-1.86728356349747	-7.16455460514100	6.39572377218707
H	-2.33202663749672	-7.34926363933591	4.70098991003639
H	-0.01725034271106	-5.63501872520379	3.50108959703634
H	-0.59963546000953	-7.16833540573241	2.86172425452212
H	1.11348809379845	-6.97588598680414	3.23490864794277
H	0.99022384248411	-9.07655410152078	4.77376019450107
H	-0.69060039659333	-9.20962308556082	4.24318282012368
H	-0.31167479662005	-9.19058219239017	5.96988007495814
H	4.58951356175714	-4.19273124301821	4.91106476235062
H	3.08984985169714	-4.09234526306046	5.86862210140796
C	-1.50136889523841	-1.10422606134698	2.84324176601623
S	-1.32532033123186	-2.94268994584961	2.78993577162528
F	-1.99295063270921	-0.63235890187633	1.69840767633172
F	-0.31909173033194	-0.52447437514670	3.07165810726653
F	-2.33341137127606	-0.77089861830068	3.83674062382861
C	-0.69785767757961	-5.23988919926335	-0.01014443842072
F	0.48153032811146	-5.75427541118492	0.36756740505698
F	-0.92533631845008	-5.59972813529146	-1.27664369722809
S	-0.67846690570954	-3.39745646921796	0.11683735491282
F	-1.65165407640781	-5.76880183196943	0.75929797167877
O	-1.99703910934735	-2.98881184430492	-0.26867880362960
O	0.43932561502884	-3.02168064803259	-0.70513542243612
O	-0.67777407690726	-3.24697511115433	4.04152302399564
O	-2.65365279701499	-3.43946130081315	2.57405996607560
N	-0.28117713030454	-3.17175805784531	1.63112262028973
C	3.26919074535218	-3.39994721423200	1.39079319411983
C	3.39417128924153	-4.55737838718907	2.39458082644346
C	2.78232229682069	-4.03431188089569	3.71157920436787
C	2.80612175345361	-2.53437981324595	3.55916396314322
C	3.03258786038610	-2.21119525418074	2.28349507006585

C	2.42638969638989	-1.60083224215531	4.66331887906184
H	3.10743740110737	-1.68126375921034	5.51801805674250
H	1.41470440036243	-1.81700033587849	5.02012736565118
H	2.44793357543842	-0.56907499397545	4.31056365040553
O	2.93179927905913	-0.95612281817950	1.80211817415157
H	2.42116879509060	-3.52250251257058	0.71384869015083
H	4.16789228448898	-3.28910177295823	0.78020971373295
H	4.45081969041527	-4.78894115531640	2.55283004590783
H	2.90259064888442	-5.46741133639010	2.04448706977504
Si	3.53922498714351	-0.19199883016121	0.43839911812635
C	3.48600323677893	-1.26605319700223	-1.08919699618921
H	4.30163652038216	-1.99182091574880	-1.10643096263335
H	3.59374633904620	-0.62578901997529	-1.97030487515303
H	2.53831225952090	-1.80067854548935	-1.17112290803997
C	5.30711541666315	0.29475637064172	0.81015132932615
C	2.40640085513706	1.29815879482947	0.24506583319277
H	5.36333405094860	0.97746344737218	1.66174794011011
H	5.76767872252872	0.78194271127564	-0.05389798717292
H	5.89724012719443	-0.59567581482506	1.04797980733187
C	1.01701805236787	0.80932763334380	-0.18856876268803
C	2.97669352873075	2.24347313113051	-0.82078741914814
C	2.28836042535024	2.04977431851540	1.57739664201082
H	3.95648001436343	2.63448409067199	-0.53034854423991
H	2.30518669796735	3.09887779483007	-0.95967541883460
H	3.08272946473708	1.74816698855792	-1.79058881976441
H	1.04843134303189	0.31882584984444	-1.16516211928003
H	0.32529472675791	1.65699827559399	-0.26098100484084
H	0.60081279990876	0.09733117673701	0.52835487947036
H	1.64173663405358	2.92689848940715	1.45494472440839
H	3.26221865544305	2.40229973450228	1.93137818395160
H	1.85342305278435	1.41578260023974	2.35327330988339
H	1.74200324539499	-4.35992053011836	3.80891255024608

product 1,2 cyclic

C	2.68818780796906	1.71980243103479	-0.16032151134929
H	2.76124964992526	2.04190484129799	0.88258932232846
H	3.49639724410676	2.20896385399427	-0.71761005029756
H	4.27726057130864	-1.35029338649904	0.18465316145112
H	0.96354731689277	1.01510500247369	2.46800375142571
H	-2.19749206545001	-0.16117776035067	1.84929130109456
Si	1.38586048147529	-0.61867061834410	0.65317670628262
C	4.14684870113828	-0.26904923879529	0.28738017092946
C	2.80350088563403	0.19504360327383	-0.29024036067370
C	1.35324026410577	-0.00540416389202	2.42527844869873
H	4.24478996893618	-0.01573137954553	1.34715428270411
H	-0.97988583688529	-1.43843576594951	1.80542914994607
H	2.36177368193453	-0.00293417005017	2.84861340072281
H	-1.97335421243195	0.70184955725591	-1.76797247355106
C	-1.81275922538348	-0.99271635245649	1.26093217847785
O	-1.12296164210293	2.73668263758012	-0.84493252662576
H	4.97293745796085	0.21655682323634	-0.24570385964701
C	1.53821288008329	-2.48588785007951	0.62617826640496
H	-2.86623332694269	-2.86443809841696	1.73929824773035
C	-2.15025487963685	0.61151539331113	-0.69672727965553
H	0.72645858237878	-0.63239218816742	3.06329450811309
C	-2.84708923075109	-2.09220494934805	0.96607488655386
O	0.03431560283935	-0.10004076495090	-0.16525566708658
C	2.73011368118034	-0.19021709687393	-1.77309799704531
C	-1.30935379114385	-0.54961002233981	-0.12608322509843
O	-2.04497856242780	2.19424020769967	1.12578729976472
C	-1.78637605110494	1.91147369856489	-0.02166667113459
H	-3.86173874553308	-1.68163681493204	0.90012958570248
H	3.55237584696205	0.28020956807325	-2.32586811962858
H	1.78904281794456	0.13850190288184	-2.22044735842472
H	-3.20885363926884	0.41046357903608	-0.52195316753070

H	2.81399503584636	-1.27221941428624	-1.91377159460233
C	-1.53921217752838	-1.79333490992253	-0.97601079750404
C	-2.39589122918961	-2.61352747814476	-0.37627786216574
H	-2.76562806245757	-3.53827812320366	-0.80792325385131
H	-1.21654130769552	-2.87516212134501	-2.79650221571464
C	-0.89689574069768	-1.94865160051819	-2.31636508495713
H	-1.15044114225943	-1.11193302456950	-2.97453648615012
H	0.19165238115824	-1.95893644148280	-2.22308044650806
H	1.63296521005204	-2.86831204468018	-0.39289853488543
H	0.66806395699852	-2.96181935088069	1.08432353359697
H	2.42433223976108	-2.79371820595813	1.18907979338707
H	1.73679927413750	2.07908224836556	-0.56134114946547
C	-0.69240161161071	3.97464411398751	-0.25539688035094
H	-0.16947429646108	4.50890718914858	-1.04582065517331
H	-1.55218412591777	4.54773212996283	0.09291182154357
H	-0.02194399284899	3.77826685860489	0.58195908501876

product 1,4 cyclic

C	-0.54313734216778	0.90791099904532	-1.66803130045867
C	-0.52435842107484	-0.54162107357370	-1.15519306372104
C	0.23542933097948	-0.50145085080309	0.19018445990416
C	0.13668400350267	0.94859287191355	0.60859618190030
C	-0.28354673462162	1.69774664957363	-0.41003921864913
C	0.56318671724583	1.43192355540819	1.95757415613911
H	1.58852719305039	1.11461081266670	2.17714352885725
H	-0.07599225299055	1.02665511631383	2.74867441358840
H	0.52079316849234	2.52057914004037	2.00845781410160
O	-0.37115191957263	3.05838616381143	-0.39556809845502
H	-1.49311515249068	1.16768456294886	-2.14176093304192
H	0.24749543809597	1.10678383168122	-2.40081542461835
H	-0.07866898255823	-1.24033146823371	-1.86548903464621
H	-1.55020685089076	-0.87177341141793	-0.96973214183721
Si	-1.76079007547717	3.92752288783132	-0.76742839775448

C	-3.24666206457100	2.94814985886382	-0.19381093321339
H	-3.37014436650079	2.03963314315271	-0.78905495681297
H	-4.16008947962672	3.54088525159439	-0.29478207745702
H	-3.14197987924769	2.65482078785783	0.85359100472951
C	-1.84240094265679	4.22564883202969	-2.61223933444607
C	-1.57635482374870	5.54818608223808	0.17350208270051
H	-1.88834590473682	3.28094232004723	-3.15920745807309
H	-0.96490884986507	4.77644916679267	-2.96043951407863
H	-2.73338632643330	4.80547453234423	-2.86904368360224
C	-1.60807122548953	5.27433389269676	1.68269594083631
C	-2.73257187085459	6.48695033943394	-0.19761138975678
C	-0.24230468578134	6.21110260836986	-0.19439235293430
H	-3.70573527538822	6.04499886010947	0.03759823967801
H	-2.72288537027707	6.73888037844575	-1.26177132553750
H	-2.65036403308785	7.42382602715289	0.36579515233204
H	-2.56067442168077	4.83530345703054	1.99237971392923
H	-1.47674573788436	6.21079501255317	2.23771118986718
H	-0.80773155543165	4.59227452333336	1.98160676749157
H	0.60481977049870	5.56823223334040	0.05837772766867
H	-0.12774848614672	7.15348912520249	0.35445345196318
H	-0.18682281934573	6.44094797684184	-1.26243571748122
C	-0.34221661372643	-1.47101677454585	1.23149174004485
H	1.29064870294965	-0.76385893366511	0.04140924560932
C	-0.32329033678963	-2.89097550157817	0.72779312796607
H	-1.37846939553662	-1.21027436523570	1.45226976040614
H	0.24222107127015	-1.42125196560167	2.15364553375299
O	-1.29777039271532	-3.53843465517194	0.42253086785352
O	0.93060520536993	-3.36393279525619	0.62695579335048
C	1.04608464778639	-4.69884851559628	0.10713905494804
H	2.11236017387231	-4.91410867477439	0.08634029749070
H	0.62468591591770	-4.75037036580197	-0.89732046968992
H	0.52523010543557	-5.40359927391005	0.75598829295596

Substrate linear

C	-7.37622857000472	5.91378797246807	-0.72456914907508
H	-6.58515537406154	5.17069730564982	-0.81103928541626
C	-7.02337173290989	6.93818144867249	0.32606435673983
H	-8.32032479260818	5.42885624796851	-0.45907243600861
H	-7.52939107622241	6.41390723508807	-1.68547657229616
O	-5.98097387839715	6.88132204521458	0.95247135221956
C	-8.02642916353233	8.01676292517235	0.54381531790082
C	-7.82775177097558	8.97909207588682	1.44234221385336
C	-8.77653574291796	10.09715826125901	1.72683222952744
H	-8.28640048614812	11.06063198274366	1.55637510157278
H	-9.07777018289002	10.07746857028401	2.77875196526026
H	-9.66930598883923	10.03858744469712	1.10197120670484
H	-8.93026158938578	7.99160019449675	-0.05895230345217
H	-6.90314219070474	8.95051573709940	2.01500865607037

CIP linear

C	-2.39332708289875	2.28420536113436	0.32998461297399
S	-1.38601943580136	0.81027578459537	0.80898315176993
F	-2.41418358407494	3.15216065720569	1.34644075015704
F	-3.64380050666963	1.94532614919693	0.02855916447613
F	-1.85080618187393	2.89524032888419	-0.73281505669107
C	-1.70416039807194	-2.72576660058821	-0.26121838022083
F	-1.47833881564060	-2.70292088977321	1.05255394257660
F	-0.53878029791970	-2.92109269652543	-0.89176225456335
S	-2.49592761871383	-1.15822554221746	-0.82993880988310
F	-2.51044766972637	-3.75307939788603	-0.53937930000771
O	-3.71505154141320	-1.06792568312072	-0.08332156029664
O	-2.55336876864144	-1.31351752225181	-2.25818505178700
O	-0.06390968463972	1.36544321258143	0.96933983300748
O	-2.04072834186792	0.26407145984594	1.95997824179232
N	-1.38734626841381	-0.07203941340891	-0.49854375030996
C	1.90248980452036	1.86904568096263	-1.01953084623393

O	2.37052864462474	0.73456458864385	-0.67887935928469
Si	3.48802074736787	0.21030700978132	0.57185103440594
C	3.14032218614289	1.12417766562249	2.15120442559063
H	3.56483440447874	0.54709832646973	2.97814401840164
H	2.06583007298297	1.21542626770071	2.32001608771906
H	3.59304226774606	2.11667399125930	2.16918149017000
C	5.16765455739808	0.57949377998242	-0.13746597026700
C	3.10705563623498	-1.61582050551271	0.69774991386402
H	5.30556926920603	1.64923384355562	-0.31201092750436
H	5.32433196011559	0.05157453150075	-1.08076012274749
H	5.93834164123428	0.25534485316050	0.56755215134429
C	1.73056735835258	-1.79747725642766	1.35056858169144
C	4.18777482315196	-2.26705191375856	1.57566287667728
C	3.11419929726712	-2.27201943709482	-0.68898604246915
H	4.23004055922804	-1.81910925410813	2.57302408232830
H	5.18059701216910	-2.18996051477473	1.12292819410073
H	3.96205764723898	-3.33107037622779	1.70335571688250
H	1.48363735908625	-2.86248729104435	1.40751387314123
H	0.94627561878470	-1.30367557532423	0.77461519141334
H	1.70819721544642	-1.39289848424727	2.36574813323696
H	4.07637770499621	-2.14691271983297	-1.19446237971193
H	2.33232661222526	-1.86145532816380	-1.33158249093850
H	2.92904948190845	-3.34671069209533	-0.58687222014858
C	2.30906220947365	3.10708646849941	-0.30402561496702
H	3.37416595055057	3.09642336203896	-0.07226733505953
H	2.05731682854127	3.99051312150815	-0.88682411107257
H	1.74902985918164	3.13216852216080	0.63531572705595
C	0.97839983147252	1.94895116658897	-2.10162156775213
H	0.64764914728158	2.93651150443704	-2.39773634629829
C	0.47448765026048	0.84300093009243	-2.68572699988307
H	0.80239299823455	-0.12829733816328	-2.32910905141075
C	-0.51694522105737	0.85948211220040	-3.78221687873178

H	-1.41013981020646	0.32241265534077	-3.44948389785986
H	-0.77791576300930	1.87230853580416	-4.08863536341909
H	-0.12243771026410	0.30073615719308	-4.63728991805733

TS 1,2 linear

C	-2.36918730279929	0.42714153375217	1.54688288297334
O	-1.81674586028839	-1.80018440492853	1.88093785471736
C	-2.43393526068025	-0.87664816746141	1.14851019869141
C	-1.73273423018032	-3.14535824327742	1.36262205917236
H	-2.73083631166171	-3.55692314767931	1.21542736516992
H	-1.19970221056791	-3.70413959105926	2.12778663471885
H	-1.17035140024891	-3.15871596213176	0.42978612788170
O	-2.97453935867624	-1.31568370208768	0.03611886147928
Si	-4.24609962304636	-0.73162884063787	-0.95044783336269
C	-3.98628095346884	-1.70488779364252	-2.51205546757283
H	-4.79932191905105	-1.52270859840238	-3.21980982379379
H	-3.94239146781104	-2.77629343360410	-2.30524566352970
H	-3.04821391142574	-1.41188388848966	-2.98839320519583
C	-4.09776200376858	1.10028992905782	-1.27444157205502
C	-5.85499972257634	-1.16367633629374	-0.08000211560003
H	-3.05690109302697	1.38545695770467	-1.43744165631883
H	-4.50571097843100	1.71110306287494	-0.46689596211523
H	-4.65785759025430	1.33309236872875	-2.18502590188080
C	-5.90781240553381	-0.52942565779483	1.31591608296275
C	-5.98612805549475	-2.68677821113554	0.04914596294714
C	-7.01237801982729	-0.61848564755747	-0.93122246481383
H	-6.00192728002232	-3.17512266509364	-0.92868477383636
H	-6.92022618155671	-2.93937761267086	0.56346109483376
H	-5.16148502341711	-3.11292143868139	0.62774903189739
H	-6.87295034512844	-0.75019553445690	1.78600935769519
H	-5.79985089152953	0.55810156641446	1.27546081992363
H	-5.12442403395325	-0.92216878875002	1.96932662776059
H	-6.97117790877200	0.47105838100077	-1.01846294538466

H	-7.97023771710398	-0.87872161854701	-0.46715531025858
H	-7.00866856709481	-1.04121698751321	-1.94044234891873
H	-2.03188665783720	0.63408121108358	2.55099562336915
H	-2.95692424487044	1.16772136947295	1.03067804885068
C	3.65837279477914	0.49052352537092	-2.17205540881172
S	3.79632349103835	-0.27557986560925	-0.49610231127656
F	2.61183810510804	1.32655878722931	-2.22547026838757
F	3.50079685419846	-0.43894012316089	-3.11606436172888
F	4.76516505202738	1.18883944506638	-2.43836854799774
C	2.54067582931777	-3.43593132328038	0.89443342528958
F	2.35861983409673	-4.75456812788592	0.77548287411601
F	1.65227835064573	-2.96871666821742	1.78439044053198
S	2.29685028182902	-2.62588356481073	-0.74715204686472
F	3.76591147567610	-3.21238630994805	1.36644199296887
O	0.90658077444963	-2.87750492675819	-1.03450392010293
O	3.29318069599185	-3.19990313343535	-1.60171255414281
O	5.01303866757548	-1.03111901611714	-0.53499209379934
O	3.69830029530802	0.85122434705432	0.39521190647529
N	2.45478922839506	-1.09901859240073	-0.39097314337949
C	0.50969658207926	0.55967127662574	1.67759095784584
C	-0.39765651006519	-0.15400055174596	-3.12346580001481
C	-0.48277968554461	0.66864242654061	-1.88268353978245
C	-0.38957112365324	0.16582369217263	-0.65095753113757
C	-0.32701451030078	1.01215361162420	0.52942746750193
O	-0.57279879314740	2.27489499254241	0.35389498542722
Si	-0.24539966023280	3.67545880260184	1.30900179857508
C	1.57084676771431	3.70591633363420	1.70980810541799
H	1.87753685323775	4.73388957766030	1.92329322055628
H	2.16565707606972	3.34308481233237	0.86813467475692
H	1.80908960111132	3.09554706635609	2.58244070658818
C	-1.32734745646761	3.60849893374274	2.82395808471479
C	-0.74925759607689	5.04015952362563	0.12459718311271

H	-1.05407357964108	2.78389390149816	3.48524226097552
H	-2.38178262735262	3.50615461213353	2.55858743451891
H	-1.20702819605553	4.53809157054986	3.38779736357941
C	-2.18818307846206	4.81461398704506	-0.35919014121213
C	0.20366759284941	5.04448779400818	-1.07915920521981
C	-0.66056432449283	6.38917000621633	0.85309968724497
H	0.18719028909407	4.08869752443575	-1.61011378712243
H	1.23505593642295	5.24362274668789	-0.77614566827530
H	-0.09360290136670	5.82635029580455	-1.78692376566040
H	-2.48096137798154	5.62071884200857	-1.04125355173239
H	-2.89945885312775	4.81005470091347	0.47201534301687
H	-2.28705232031856	3.86816183819993	-0.89666767389431
H	-0.91967451439049	7.20022500884633	0.16366280875388
H	0.34874599463211	6.58517017417260	1.22692075094603
H	-1.35277956347199	6.43943406785196	1.69840874613431
H	-0.58192793373542	1.74384723743205	-2.00472199763923
H	-0.23161466720047	-0.89264238430205	-0.49688044298421
H	0.48817642917401	0.13915602898995	-3.69523668056633
H	-1.26427775002564	0.03185848806994	-3.76546836373695
H	-0.33062165456166	-1.21785341074433	-2.89493674488875
H	0.29685850255901	1.11880542338681	2.58621672386414
H	1.55184870717748	0.72360118221870	1.38330253540067
H	0.37713771322099	-0.50568077912714	1.84938973043793

TS 1,4 linear

C	0.34623290013557	3.81928974664708	-1.19104911326866
O	0.99426623483851	4.07525827131768	1.02510546213301
C	0.02787808237651	3.85531323795918	0.12710098903958
C	0.60027922874279	4.27273917930116	2.39052127688576
H	1.52475166074911	4.46193458989350	2.93006298805871
H	0.10942783007345	3.38354154905553	2.78379884967085
H	-0.06475419122571	5.13352373038574	2.47197930310753
O	-1.16079958960332	3.58135435523016	0.62827958126104

Si	-2.69275187181438	4.06576995877802	0.03173995706986
C	-3.25292516818230	2.93657081894079	-1.33988365184264
H	-2.52938183740646	2.88954737539809	-2.15579562380587
H	-4.19116619817467	3.32260047781741	-1.74943944585521
H	-3.43350283614550	1.92107421158848	-0.98260386317174
C	-2.51813951098693	5.82132795282751	-0.56688288487900
C	-3.79753333016066	3.91746832548259	1.54378950112687
H	-1.95094545060308	5.87077887358352	-1.49859908297806
H	-2.01409362765785	6.44374737180353	0.17640348974941
H	-3.50861414718745	6.24690621087838	-0.75182247728023
C	-5.25289703837352	4.14768956349083	1.10972185931154
C	-3.40601265598196	4.96851551523868	2.59041605937073
C	-3.66293184888257	2.51690635613405	2.15514439064487
H	-3.51043410545767	5.98543131598027	2.20255639202831
H	-2.37435003058746	4.83508756540096	2.92558746486970
H	-4.05538267991350	4.87877886858617	3.46854261162850
H	-5.58599527666609	3.39082949723965	0.39447415696209
H	-5.39062129658691	5.13241251144232	0.65251788575611
H	-5.91365820579967	4.09365471324387	1.98201657072476
H	-4.31165552099544	2.42974700347606	3.03411878839348
H	-2.63636679195651	2.31711347179634	2.47335592516191
H	-3.95767127731584	1.73722638302003	1.44806025739853
H	1.32912585991261	4.14633459801241	-1.49773015496559
H	-0.43630210841612	3.73840111422041	-1.92822639669905
C	2.57270888439030	-3.69260189931554	-0.91200505643323
S	1.57341216271293	-2.33642001360619	-1.67817595204556
F	3.84986334182761	-3.54859143636397	-1.28180983923075
F	2.15001643838861	-4.89363960027217	-1.29643227577209
F	2.51159317330265	-3.62864659674439	0.42396624784414
C	-1.94291388216377	-2.33956489227128	-2.72263833540220
F	-1.15155459210725	-1.76445927647568	-3.62900858170919
F	-2.57619873666030	-1.36739106869922	-2.04731420690033

S	-0.98981799989601	-3.43210479941442	-1.56937211113036
F	-2.86403340733227	-3.06741200212490	-3.35828453167674
O	-0.55888049538939	-4.52135337995329	-2.39506256180475
O	-1.92075605898053	-3.67660540942549	-0.50130110868553
O	2.18274242477198	-1.14331037158326	-1.15205042496301
O	1.64555346805669	-2.58236391289517	-3.08986102849716
N	0.14793923381745	-2.48523614716534	-1.00950325387952
C	-1.77647464901766	-0.36179322421352	0.90755270808183
C	0.96122796577334	1.51406269833930	-2.75623701804542
C	0.68475145623091	1.48530042208061	-1.29029649074053
C	-0.47040961702392	0.89859997483164	-0.80118516784989
C	-0.58091204792107	0.42909551148597	0.49466183111837
O	0.38725796016615	0.62873412635248	1.35186291587514
Si	0.74764053075819	-0.16048596813220	2.84738931486592
C	0.38544787382239	-1.97801404823924	2.68396124084360
H	-0.65442953092119	-2.21338441940811	2.91694857615131
H	1.02040821859928	-2.53082518662641	3.38188532135964
H	0.60364578320117	-2.33498771140312	1.67550933349309
C	-0.28361289513248	0.64606628238883	4.17852035099733
C	2.57827338342236	0.17624045451178	3.08936283927755
H	-0.13678454616688	0.10128892049548	5.11578914996438
H	-1.35011107047161	0.62086096204852	3.94583845200005
H	0.00783656794768	1.68488921072974	4.34618254193447
C	3.39028385521625	-0.58493412708349	2.03419371873604
C	2.97240688377790	-0.31574459255082	4.49111831368305
C	2.87565802836536	1.67583865208044	2.98134513979938
H	2.44133768388659	0.22812560439408	5.27776126174390
H	4.04577317121390	-0.16029749132647	4.64628607210110
H	2.77363490552403	-1.38360815462639	4.62058868721860
H	3.12223868809218	-0.28563736342889	1.01917983420556
H	3.23871797952244	-1.66426279691298	2.11013113958760
H	4.45875327586022	-0.38693365262418	2.17758231804987

H	3.94951349614737	1.84946654665640	3.11483069570871
H	2.35134094842760	2.24577954077333	3.75316068014203
H	2.58977092798502	2.07568342275943	2.00553211751879
H	1.53252079573735	1.57098944869465	-0.62164441932363
H	-1.29324076131526	0.70065814184909	-1.47302312417587
H	0.04857975692555	1.66057994749499	-3.33568693770932
H	1.37047861218948	0.52974945407429	-3.00880722471202
H	1.70273667073997	2.26404038101047	-3.02613600789387
H	-1.54958069351698	-1.42333452528089	0.78026146392348
H	-2.62594541300419	-0.12536001830871	0.27101249611484
H	-2.03451004852679	-0.17793434574703	1.94952775563364

Intermediate 1,2 linear

C	-1.56695510865720	0.97130225130948	1.32129398809440
O	-0.99684503922584	-1.21016912764412	1.97814710308274
C	-1.72527238097405	-0.49936178575702	1.19849499242938
C	-1.05836956113282	-2.66825099310477	1.86652799512697
H	-1.99488328833980	-3.00129424414384	2.31156595785401
H	-0.19879828169959	-3.01986812623558	2.42723698057536
H	-0.98928929063554	-2.94580478330573	0.82009003442093
O	-2.49934638893597	-1.07421406784148	0.38705933872753
Si	-3.73836986395205	-0.68181149399872	-0.81534503110235
C	-3.15122396690624	-1.58178965832564	-2.31601753297548
H	-3.91270465854021	-1.51490832876943	-3.09858617466238
H	-2.95026387712582	-2.63225938444088	-2.10461251055889
H	-2.22906370352386	-1.13346472051209	-2.68978517092310
C	-3.86891239521517	1.14756166564583	-1.10610302685254
C	-5.26217495827012	-1.40415374870512	-0.00011975992171
H	-2.90509223634476	1.57958967251907	-1.37917007107349
H	-4.28911976404876	1.69972331578388	-0.26387667598266
H	-4.54411427638512	1.27708550345554	-1.95846531066184
C	-5.48061624943017	-0.76685929535410	1.37860810765265
C	-5.11177951846374	-2.92374352188853	0.15016138467896

C	-6.46452894773497	-1.09174017235869	-0.90575530105522
H	-4.99956711419154	-3.41542185308795	-0.81936075920644
H	-6.00318712690125	-3.33760430925110	0.63395315714741
H	-4.24584745943667	-3.18591743253253	0.76441171870146
H	-6.39916564873002	-1.16072269176688	1.82663024212891
H	-5.58426184589512	0.32003581083766	1.31464133384577
H	-4.65913704273062	-0.99390911086105	2.06445872238195
H	-6.62746164775835	-0.01495603771524	-1.00598887287151
H	-7.37291614731269	-1.52699990196239	-0.47582961764456
H	-6.33852959591319	-1.51331744674474	-1.90758604906848
H	-1.48920324006948	1.21611270060096	2.38015475612788
H	-2.43840876171302	1.45813732368111	0.89753475593027
C	3.69645694327857	-0.93211889220232	-1.04213443698771
S	3.20393599332763	-2.37522132022033	0.00567634905083
F	3.01704655095078	0.16511616085631	-0.69265710028870
F	3.48221068258454	-1.16430644756386	-2.33602209030117
F	4.99977672750552	-0.69621670914112	-0.85979188736070
C	0.92203895030607	-5.10751319766716	-0.72294856298386
F	0.22186207811087	-5.86156856440696	-1.57778133752463
F	0.29294288942200	-5.12754939713226	0.46227953501588
S	1.00330688263788	-3.38076760963403	-1.39079399071176
F	2.12610965920420	-5.64477929837376	-0.56817995406126
O	-0.38859876568036	-3.00943094976136	-1.47380170744431
O	1.76281331368706	-3.50093088006729	-2.60060016744940
O	4.00844231304937	-3.45660335475438	-0.48360719694162
O	3.39430975704418	-1.89585342352091	1.34665059906651
N	1.64028001756343	-2.51763211254924	-0.23205047876109
C	0.94162696964652	1.34723574730174	1.43694122961047
C	0.09642087257479	0.47242307658533	-3.19579221581565
C	-0.17605969912277	1.24895998484102	-1.94227138579666
C	-0.10392588979815	0.73500106954988	-0.72338240478192
C	-0.30173271349235	1.50669069968258	0.56287353215811

O	-0.57037212817115	2.85515720424160	0.25927058008241
Si	-0.47125750609700	4.23876368605860	1.18767051674916
C	1.31066050923465	4.74641787393320	1.44380267498729
H	1.36051316041061	5.77631577243602	1.80885287366210
H	1.86960630928656	4.69052325377277	0.50649666733260
H	1.80924337871781	4.10821023890009	2.17632978700790
C	-1.30363887767548	4.00624217498376	2.85201086617026
C	-1.39494378334788	5.51358351260142	0.15012123168171
H	-0.78244913480161	3.26837120387252	3.46722201009709
H	-2.34679142381360	3.69696307617890	2.74903033949067
H	-1.28624820073407	4.95371782226821	3.39836630123433
C	-2.81390510266474	5.01167731971903	-0.14688630144235
C	-0.64845701499019	5.73329151875729	-1.17267872045009
C	-1.47723094825147	6.84275518883026	0.91299943585365
H	-0.55577372989847	4.80298391733334	-1.73962179070561
H	0.35748552307028	6.12839645424844	-1.00543045858533
H	-1.18994779471219	6.45509317357732	-1.79561315423991
H	-3.35852565290561	5.75310668912722	-0.74348732012358
H	-3.38453252842359	4.84414463470846	0.77194011194085
H	-2.79359595440329	4.07563970238738	-0.71003211911598
H	-1.99107253616018	7.59380810733204	0.30175786534545
H	-0.48525083783730	7.23655437904416	1.15259306146658
H	-2.03588907795428	6.73933535393390	1.84759370733970
H	-0.42115252899309	2.30213282455289	-2.05077497156057
H	0.17473985001738	-0.31030660441481	-0.59902087241667
H	0.96099517152632	0.89023480864961	-3.72057326117177
H	-0.75252081371671	0.53200858371854	-3.88443295238293
H	0.29975482021203	-0.57812049605645	-2.98085055546253
H	0.79806945327709	1.82621928859293	2.40794940776482
H	1.79532082769973	1.80179499566823	0.93520322680436
H	1.16632264349444	0.29237974369477	1.60190783460925

Intermediate 1,4 linear

C	0.30379408248011	3.10019892387077	-0.91390554796975
O	0.67188451325287	3.71785146642230	1.31850853529454
C	-0.21538405269817	3.44262293693235	0.43097635716049
C	0.23834788319666	3.92343832149172	2.69369304506407
H	1.14909239135468	4.14894776315877	3.23816039962819
H	-0.21871598661294	3.00319589747942	3.05222715746479
H	-0.46182113804329	4.75544819484826	2.72961440554019
O	-1.43026210367681	3.42828567128026	0.77407866203071
Si	-2.99273312222509	3.52843170524024	-0.05813622423457
C	-3.21066216470497	2.17540659072431	-1.30201161100543
H	-2.45415718168621	2.18345693059986	-2.08671152332848
H	-4.18281736794117	2.34019189723563	-1.77885592982157
H	-3.22287874617123	1.18906855233925	-0.83960369341759
C	-2.92965694548365	5.21043600798392	-0.84231893516772
C	-4.15679560074565	3.37770219414573	1.39706116859203
H	-2.19063863199034	5.23782933154354	-1.64749307666635
H	-2.68198141689697	5.98500277367386	-0.11359241477282
H	-3.90430606876025	5.44849001819267	-1.27752121002876
C	-5.59335938702961	3.37197069217909	0.84866212205836
C	-3.97999615771530	4.56327506359976	2.35466781264296
C	-3.88622947685096	2.06377207719698	2.14389640166613
H	-4.19666379242339	5.51616089673494	1.86439649078390
H	-2.96517288409774	4.60824091502437	2.75864009388744
H	-4.66916645338760	4.45917661066445	3.19936585127086
H	-5.77414925109943	2.51360370832406	0.19643868218327
H	-5.81920437413108	4.28363995860568	0.28659924865279
H	-6.30326904755482	3.31212716345669	1.68015076232974
H	-4.58571367370408	1.96617656738259	2.98098808849759
H	-2.87148440757174	2.03083057509537	2.54987673786356
H	-4.01897238497735	1.19324750360353	1.49698958005222
H	1.13326202628624	3.77005717909334	-1.14633829101903
H	-0.48352796580517	3.22277822669967	-1.65669547681285

C	1.99654621751295	-4.44981781694968	-1.02413840823426
S	1.31129457551396	-2.84375757374615	-1.63387443371922
F	3.31173262326441	-4.48388338938301	-1.27037872401051
F	1.42426577679122	-5.49063201322543	-1.62585492503816
F	1.81353293994584	-4.56988364740751	0.29619039541542
C	-2.00038742370880	-2.06801692921955	-3.02883269774830
F	-1.02668022967544	-1.42616200675062	-3.67834374767707
F	-2.60413652557366	-1.18798091640079	-2.21267845942638
S	-1.35562408563438	-3.52135641917422	-2.07943005062439
F	-2.90326285310332	-2.48563312636456	-3.92087976206581
O	-0.94591527572550	-4.44443897761435	-3.09771575350417
O	-2.45409461606160	-3.86301631988033	-1.21872348602945
O	1.98244845540313	-1.87095369706217	-0.81330926617399
O	1.56707660567272	-2.84706323038110	-3.04646182697417
N	-0.20528868955986	-2.89999056903391	-1.19211384625250
C	-1.54289545945205	-0.74771173909787	1.13277363116407
C	1.32949778785404	1.27485696025984	-2.29145957871720
C	0.80642446767987	1.62329016680713	-0.89907139315113
C	-0.28691768820883	0.68769108217492	-0.47193219930592
C	-0.44524978035126	0.20370643143072	0.75821937694136
O	0.39662688419921	0.60296724037679	1.76244423817161
Si	1.09909008695985	-0.29419820492721	3.00615121955186
C	0.96115381601092	-2.13111302913618	2.71783288395704
H	-0.01452209792994	-2.52249492869548	3.01117747931665
H	1.72094021118521	-2.64474183436963	3.31385364686177
H	1.13664963824662	-2.37584983705651	1.66847396251243
C	0.21481538975919	0.20424480079205	4.57942338931387
C	2.91319424551564	0.21823837375585	3.05396042524083
H	0.57051230024531	-0.38912923423465	5.42609756697590
H	-0.86042939024229	0.03167399466567	4.47646120700990
H	0.37142341910990	1.25984408787920	4.81764747695379
C	3.63531819934759	-0.35760464439615	1.82860368558111

C	3.55370750591138	-0.34020696542362	4.33291889908605
C	3.05022621165684	1.74419661629928	3.05183850497819
H	3.09413559523395	0.08000864570781	5.23237060888443
H	4.62064706380815	-0.08945614247813	4.35616324477361
H	3.47180863124221	-1.42950356195547	4.38770162492778
H	3.18370467403214	-0.01840849572281	0.89337488002762
H	3.61050611248389	-1.44987708542843	1.82227585615437
H	4.68641120311793	-0.04515367339989	1.83186560530485
H	4.10892907582488	2.02584928161139	3.09915825484345
H	2.55102720771198	2.19518887703072	3.91530276158970
H	2.62847190171355	2.17850751472548	2.14269085751601
H	1.62432107452173	1.56998136536883	-0.17784283760514
H	-0.98552083036829	0.36374248859240	-1.23111258731688
H	0.52133794385032	1.31783760942917	-3.02785105467553
H	1.73213707776938	0.26176217501949	-2.28892788660987
H	2.11776833608742	1.96600953521615	-2.60165376888624
H	-1.15938419912860	-1.76352200783109	1.22399508274961
H	-2.31213587463738	-0.76332706981419	0.36216526368888
H	-1.99243034840745	-0.45456547540602	2.08505699583477

Product 1,2 linear

C	-1.56059370733024	0.25198104753155	1.68177761404001
O	-2.68284650970927	1.36992092355019	-0.07941053088527
C	-1.70115694993243	1.48491780553325	0.82384787932300
C	-2.83458837747865	2.48740486907623	-0.96991046486639
H	-3.64390213177985	2.21569644679777	-1.64432759563767
H	-1.91103723531227	2.65265208336916	-1.52550903207839
H	-3.08804071842843	3.38744493475362	-0.40879075333344
O	-1.02019246992157	2.47779411534651	0.93645694814011
H	-2.53454136446032	-0.00644957929991	2.10381683764139
H	-0.88339239062373	0.50885730899402	2.49546096240750
C	-0.57591533684151	-2.02544685078906	1.93821625987515
C	-2.98064055239496	-2.48850492464863	-2.11478241033896

C	-1.92227038072425	-1.85259255162709	-1.26176730833383
C	-2.06454033284224	-1.57922217822963	0.02588918496932
C	-1.00186697911701	-0.96920251606487	0.91325605519951
O	0.08178287255481	-0.55572139625451	0.10317825124957
Si	1.68852547276163	-0.28408013275595	0.43825669028014
C	2.59051198575247	-1.90293532771743	0.73308711816599
H	2.29390577779259	-2.65646174169846	-0.00089568861440
H	2.38829082467548	-2.29894078737649	1.73072076490809
H	3.67083027149596	-1.75297477558973	0.64691395573402
C	1.91676020413182	0.82620824296224	1.92767728877123
C	2.32867568703404	0.53601683474116	-1.13653104195624
H	1.38648454744745	1.77209807244153	1.80054422565195
H	2.98053006628804	1.03593882475052	2.07257718764091
H	1.54998255821016	0.34777027720844	2.83984054931671
C	2.28545959637929	-0.47275198599185	-2.29218552528017
C	3.77548205250115	1.00198999304697	-0.92473408300444
C	1.45496115913127	1.74604446627704	-1.49030498361313
H	3.84221686952588	1.76439341867821	-0.14371199486217
H	4.16717264558508	1.44044866763031	-1.85033321196987
H	4.43553614302602	0.17423138758952	-0.64782191524744
H	2.62638916816876	0.00232456899389	-3.22007218939311
H	1.27055834880160	-0.84395494296107	-2.45981777481798
H	2.93361546347759	-1.33301608861094	-2.10193978625856
H	1.84028672373819	2.23465935864183	-2.39368336108593
H	1.44213523729642	2.48650152577970	-0.68607693370470
H	0.42189613234726	1.44509795522765	-1.67943251369011
H	-0.98555402935302	-1.59058348832915	-1.74549212469252
H	-2.99539337069118	-1.82512291569740	0.53434957473247
H	-3.25900242320645	-1.82852971313513	-2.94262957953292
H	-3.87919417992538	-2.71100994324003	-1.53480366139860
H	-2.61624558871513	-3.42013630652053	-2.55904323926559
H	-1.44264943543803	-2.35722810844892	2.51460502893586

H	0.16214212222732	-1.62107874402877	2.63480195716994
H	-0.14990948812435	-2.88770873620569	1.42336135210896

Product 1,4 linear

C	3.38357771548045	-0.47055128041312	-0.78320882176606
O	2.47545188739388	1.66523769865426	-1.26882852938109
C	3.11321620623123	0.94301658350779	-0.33489935153605
C	2.12847713445250	3.00453338247037	-0.87907951514475
H	1.59730683424482	3.42837665543061	-1.72863347126586
H	3.02833346605130	3.58033204971704	-0.66097784071449
H	1.48606465589734	2.98365217417065	0.00176544804507
O	3.41675905135118	1.38532218893583	0.74896991583670
H	4.46538925056708	-0.62854337150054	-0.75261747666718
H	3.04051553495384	-0.60642962621599	-1.81057839147659
C	0.90574542786756	-0.30530728274862	2.46613871192203
C	3.02903973409305	-2.90183751407656	-0.25990172521383
C	2.68317013037492	-1.47010677502248	0.15520253026519
C	1.19358663578214	-1.24918672279035	0.13061833369409
C	0.44232705192983	-0.74139593353623	1.10889566711754
O	-0.90995972783477	-0.56481504291178	0.98665309400322
Si	-1.82146717119374	-0.30120834692375	-0.39719102867456
C	-1.97128023137477	-1.87288504263269	-1.40652523076466
H	-2.84239949876898	-1.81748469317555	-2.06555833729223
H	-2.09596027896184	-2.74056845106630	-0.75357603076011
H	-1.09258203136173	-2.04068745693729	-2.03331082223986
C	-1.04867719296095	1.06349874508743	-1.41272175528919
C	-3.50337124782859	0.20579418388722	0.28590135314788
H	-0.03224302241671	0.80389871047940	-1.71457464820423
H	-1.00636398157251	1.99864231542398	-0.84870963299906
H	-1.63882756569548	1.23616070556518	-2.31726639625986
C	-4.42625412418979	0.61134894296458	-0.87155031297457
C	-3.34020571233023	1.39521021590298	1.24105787753094
C	-4.12477106382409	-0.97429113830298	1.04483203447994

H	-2.69111615204794	1.14366977987686	2.08325455618489
H	-4.31704188555107	1.69156478257808	1.64170123782873
H	-2.91377728276249	2.26496884367740	0.73240237311800
H	-5.41196087057002	0.89282029688430	-0.48273118501855
H	-4.57473851470515	-0.20688332787603	-1.58242705093461
H	-4.02980051394463	1.47002264922000	-1.42106358316919
H	-5.09612648472871	-0.68410323867223	1.46292519057786
H	-3.48636645663314	-1.29766537398178	1.87173554742744
H	-4.28896497309798	-1.83320290775049	0.38769123765967
H	3.06834521358744	-1.29555471801715	1.16045571907009
H	0.70487308061254	-1.52641877703306	-0.79980826579152
H	4.11099568300247	-3.06063783451951	-0.25546232823589
H	2.65897431990560	-3.10995139693278	-1.26874344065267
H	2.57036011783420	-3.62058809813692	0.42292328039324
H	0.69869488405799	0.76068733144601	2.59335955067143
H	1.96901492938874	-0.47075018819768	2.61960606102717
H	0.34101839479527	-0.84546445270805	3.23092375002555

CIP (with **IDPi 4f**)

H	-4.65783932863665	3.47991404368659	3.07659325679776
C	-4.30250637360496	4.24165929505669	2.36871006301298
C	-4.48389938913985	5.58881773430880	2.64532913373922
C	-4.00508995192628	6.58043416337848	1.74836370683592
C	-3.33597328663935	6.20524891556588	0.59441957875406
C	-3.13653808336201	4.82950222794401	0.27784460443869
C	-3.64190900298558	3.82269394736342	1.17701534587891
C	-3.44054894827693	2.43588525963068	0.85127255922081
C	-2.64455476094554	2.11637597731908	-0.25325871553259
C	-2.20636516122583	3.09385436282150	-1.20363055907466
C	-2.47318885902132	4.42887263035850	-0.91401409022772
H	-4.98981576167526	5.89105545537115	3.57457256857864
H	-4.15250061814055	7.64592287660326	1.97967701891881
H	-2.94022754077003	6.96556027431058	-0.09632757372523

H	-2.14594316145528	5.20190424211162	-1.62586178506615
H	-7.96371204690328	-0.72847625479307	3.19340960106123
C	-7.53616715492683	0.09382760719596	2.59851723669224
C	-8.36223061353338	1.00840558979826	1.96443898648540
C	-7.79492189479742	2.05527221073707	1.19054852972186
C	-6.41742252596463	2.18437900482046	1.07994154679085
C	-5.53779920905105	1.27023332294014	1.73257320730791
C	-6.11543079545625	0.18557526506788	2.49086453929861
C	-5.26538945307289	-0.77849749548828	3.09943371970405
C	-3.87875745161123	-0.75335773353351	2.95323428576912
C	-3.33293557383912	0.32272286523571	2.17979309589193
C	-4.10196851337741	1.34713136016310	1.62272521738264
H	-9.45563861800441	0.92183710699506	2.05345468169256
H	-8.45150748965436	2.77144842028311	0.67362550057184
H	-5.99159952245133	2.99657169555679	0.47588257776525
H	-5.72255153650491	-1.57460568296883	3.70680456828065
O	-1.96549868707824	0.35138326586762	1.95841026680939
O	-2.38309809305345	0.77556312907018	-0.52357733685126
P	-1.38251028920117	-0.09244056787453	0.47721019612287
N	0.07811006860013	0.48054618313004	0.39558248208238
N	-1.78774325748815	-1.59137388991922	0.02939578430381
H	4.12670077427969	1.48397226472732	-3.63800680776567
C	3.48341214469177	2.37284024128144	-3.70495723358633
C	3.29206182355363	3.01290312818224	-4.92147939955802
C	2.44139311334944	4.14687204973545	-5.01334136795150
C	1.78568862459191	4.61600810948687	-3.88551659232264
C	1.95529125057736	3.98001185086007	-2.62096791383118
C	2.82954809975575	2.83695543335688	-2.52709305344960
C	2.97668637506963	2.18288309073210	-1.25531339961188
C	2.16147840117472	2.58885694942414	-0.19880087092080
C	1.34297965057427	3.76292605632103	-0.24067792667862
C	1.27995376049600	4.44061294006819	-1.45685349459878

H	3.79961389127209	2.63672306461713	-5.82270211781629
H	2.29998376489179	4.64860932942881	-5.98259156436074
H	1.11113473693114	5.48362506730123	-3.95150793751115
H	0.65242497718137	5.34176258507887	-1.53055388426729
H	8.53943470184379	0.02360520843758	-0.99247824458011
C	7.78955748263985	0.81725531697142	-1.13483174469499
C	8.18024090207665	2.11415582547115	-1.42631177705007
C	7.19972755610376	3.12952300575077	-1.58105799595472
C	5.84972683694912	2.83261575285489	-1.46259403870018
C	5.41382294786285	1.50421504330684	-1.17962336065239
C	6.41026001060865	0.48163426111506	-0.99457399323822
C	5.99957318543957	-0.83306768499516	-0.65440878193762
C	4.66758122252914	-1.17483040890946	-0.41723834187072
C	3.69917452062095	-0.12874096500220	-0.56013279654973
C	4.02670326445176	1.15361497297105	-1.01323360185112
H	9.24776948039963	2.36279929814457	-1.52399297131161
H	7.51273027294053	4.16416851317295	-1.78730815623377
H	5.10268176719690	3.63047948492645	-1.56992167542720
H	6.77279861150429	-1.60417521463653	-0.52117741119543
O	2.35804998522468	-0.41122879891018	-0.31474082029982
O	2.22089565301560	1.84854008812828	0.97367442675536
P	1.56411316920704	0.32258120417214	0.94093582486626
N	1.95344394296811	-0.43122808445845	2.28058250361957
C	0.70611671568329	4.30742453472457	0.98483447915752
C	0.89858390013943	5.66943806753346	1.31110436879522
C	-0.04149216515250	3.52050926969019	1.88219962328077
C	0.39942594100347	6.20088413124522	2.50448023522126
C	-0.54549311868435	4.06306515369733	3.07019500219727
C	-0.31816037352155	5.40626629396382	3.42584093591426
H	1.49458603492685	6.30581027704443	0.63870534979300
H	-0.23676383819028	2.46627769139782	1.66248115386403
H	0.60618981858572	7.25681394273657	2.73656774717856

H	-1.09838656992499	3.39105002390904	3.73819538556331
C	4.34510217937349	-2.58388704153253	-0.06923579717722
C	3.46896523604804	-2.95341635808550	0.97136406186695
C	5.02851087892951	-3.62122724614961	-0.75030281615837
C	3.32797451375991	-4.30099424619371	1.34117465269812
C	4.89324749968406	-4.95604575628374	-0.36140911851418
C	4.05485859247202	-5.33153501934947	0.71212916955475
H	2.92528052374648	-2.18906338006841	1.54464321870860
H	5.67817322767732	-3.37097210275382	-1.60284662959219
H	2.64144893258878	-4.52861498893448	2.16690761270357
H	5.45756305186183	-5.72441676108347	-0.91182560703537
C	-3.00816948437183	-1.81338219462637	3.50686151822052
C	-1.73107637222158	-1.53463073679444	4.03786883542533
C	-3.43431100821831	-3.16166289242816	3.48506754900460
C	-0.89087155010806	-2.56883166592183	4.46418572515206
C	-2.58796207536544	-4.18670304058051	3.91152115230685
C	-1.28308180277585	-3.91812188236942	4.37885366585072
H	-1.35188763271793	-0.50602498074256	4.09464185787481
H	-4.43037275430105	-3.41309429583235	3.08878830442740
H	0.11163359096058	-2.29295022782115	4.81917368735299
H	-2.94301275108120	-5.22548569329274	3.83910744098009
C	-1.63997263842535	2.68691066816718	-2.51536601416088
C	-2.25626347900898	3.14021780090586	-3.70365378134580
C	-0.53749808268827	1.82312649812889	-2.63602627130890
C	-1.82702840017115	2.68284756585106	-4.95481468741626
C	-0.10099221143437	1.38627387489865	-3.89315917706158
C	-0.75605065156084	1.76994974994765	-5.07806723854896
H	-3.11642130692511	3.82278726380437	-3.63725706336602
H	-0.01327119421032	1.48113542394800	-1.73490216039012
H	-2.35965632753572	3.03091612933919	-5.85350033562608
H	0.76360609865182	0.71412193159543	-3.92783946920017
S	-0.93524260814227	-2.93665066973286	0.29300997406497

S	1.86034488020057	0.14574594848956	3.80219754848183
O	-1.88134334935751	-4.07474994724757	0.12453467659503
O	-0.07287222330320	-2.89399232521605	1.49514350815217
O	0.56364731415114	0.80994044901431	4.07894953636310
O	2.34403051460985	-0.90379215434297	4.71774358118859
C	-0.75242585730710	5.99199641562751	4.77960737391396
C	-1.55347989769853	7.29373777086617	4.56256295089577
C	0.51797953299221	6.30231971932073	5.60690601077609
H	1.16859784266803	7.03577479067143	5.09024004989271
H	0.24187367286798	6.72508238367664	6.59557385513133
H	1.11556206240913	5.38518994152660	5.76874743516989
H	-1.87297159320618	7.71649340493819	5.53763645655889
H	-2.45645083593350	7.10169075208360	3.95104881528408
H	-0.95291124411675	8.06837091824671	4.04560619319853
C	-0.38816797038512	1.18447240874936	-6.45149481987060
C	-1.57376718218876	0.31221700568608	-6.93021950476944
C	0.86896666439737	0.29969464709993	-6.38500796000907
H	1.11230887814738	-0.08463256650669	-7.39637974362819
H	1.74605749806642	0.86451476647277	-6.01228616046842
H	0.72671859691565	-0.57375487807400	-5.71858882338634
H	-1.35311040290193	-0.14177635751250	-7.91934319608756
H	-2.50518999041358	0.90572759736227	-7.02678981089551
H	-1.76923518281245	-0.50854471989286	-6.20974157634169
C	4.01501983993501	-6.79721531640602	1.17920445105300
C	3.60799619902713	-7.72797049106138	0.01533418594786
C	3.01364020272315	-7.00354763714525	2.32517182559070
H	1.98792324094703	-6.72475744910439	2.01862353567977
H	2.99950295655157	-8.07012398419355	2.62875938224207
H	3.27475200596934	-6.40395139965150	3.21990872939634
H	3.63276214336472	-8.78656919978419	0.34750205112686
H	4.28945413036414	-7.63047852948262	-0.85269205313624
H	2.58364903693210	-7.49541889121363	-0.33323599275801

C	-0.33973200067885	-5.07812146762394	4.72523355985009
C	1.09730039229184	-4.58679744655614	4.97379637360275
C	-0.32485528097952	-6.07394624788337	3.54327888993969
H	-1.31955821722927	-6.53305043757808	3.37704647814135
H	-0.03188184345403	-5.56393146940839	2.60409872258497
H	0.39048671182960	-6.89731231521334	3.74316041830565
H	1.76493597044441	-5.45352757053375	5.15460901383879
H	1.16447614434246	-3.92368036261109	5.85866114871438
H	1.49175308792419	-4.01918223614463	4.10776437426597
C	-0.85676629285415	-5.79048873190179	5.99515455733038
H	-0.20271276905613	-6.64932856940318	6.25499588350750
H	-0.87299421496953	-5.09628218488288	6.85961362988932
H	-1.88614201458139	-6.17812526782317	5.85338361194705
C	5.42612042902617	-7.18689251291443	1.67759018411467
H	5.74585024611551	-6.53052326151863	2.51196818807539
H	5.43125238054172	-8.23564247735050	2.04133420855364
H	6.18329304730083	-7.10304202683243	0.87250564776524
C	-1.62531736590294	5.00578953680858	5.57543841715751
H	-2.54245463786033	4.72999256313284	5.01643780132011
H	-1.07500195865517	4.07492926221016	5.81763090848548
H	-1.93651563854026	5.46851569936666	6.53399892619249
C	-0.13662792893791	2.32094023074905	-7.46565282977207
H	-1.02975357320119	2.96309233517714	-7.59882570002135
H	0.69893375235766	2.96686773550781	-7.12957203918393
H	0.12507177221674	1.90192816488451	-8.45930664110863
C	0.27197361535786	-3.14502309410367	-1.08225276154885
C	0.67007048377352	-2.13973060419651	-1.97518484460819
C	0.96644284914807	-4.37219515388642	-1.12783286461466
C	1.76930997046687	-2.31230444248752	-2.83143600652271
C	1.99790813378002	-4.59250726420756	-2.05171105010783
C	2.44095632361780	-3.53911093081627	-2.86792187400742
C	3.11533535599014	1.48236877482433	3.73647052302306

C	4.33152827979292	1.25774673283178	3.06216288160926
C	2.82380971247386	2.78514136196988	4.17796311747334
C	5.17698591100897	2.32298875770354	2.71954788608113
C	3.66562817648810	3.85985865474045	3.84347748841438
C	4.84420888551645	3.62848685823238	3.11715121922932
F	1.73491424196274	3.06507442514533	4.89649339493146
F	3.36551444255833	5.10229221294287	4.23884906062399
F	5.64640634562243	4.64720369010828	2.80406589327556
F	6.29635808807467	2.10312185095126	2.02267493242828
F	4.68194089506837	0.03962107687567	2.64213774373282
F	0.63309063144415	-5.38358362611779	-0.31706456348908
F	2.58346599186833	-5.78854712403801	-2.15176640041249
F	3.46323205386184	-3.72467125437773	-3.70138735168985
F	2.16075424829566	-1.31347396065108	-3.63541505177474
F	0.00380337185161	-0.98801404966387	-2.08342252355913
C	-4.53847011554128	-1.63379271590896	-0.66305012183556
C	-4.82719708968476	-2.92907652442332	0.00202449584654
C	-4.18927765508860	-1.76366433374680	-1.99835761218761
C	-4.61620714528877	-3.98033566798788	-1.09426198722409
C	-4.14078305514529	-3.16614999142131	-2.25868732865530
H	-5.83363390075181	-2.93551724759682	0.47260272728787
H	-5.53943756158067	-4.53368454609444	-1.37389412085610
H	-4.09262320638242	-3.06884572186322	0.82733283254849
H	-3.84616405682310	-4.72095120440968	-0.80105368529569
H	-4.57882511303182	-0.67572327512101	-0.12559545502467
O	-3.70177021252808	-3.63220774419132	-3.38096336295025
C	-3.78115858313201	-0.70021527912323	-2.97337362163721
H	-2.97420781622014	-0.10541262506244	-2.50130426044420
H	-3.34029980357241	-1.19255077434280	-3.86321162205680
C	-4.91635441594243	0.24270714345504	-3.42509783305675
H	-4.45699179007415	0.98881102401916	-4.10719000329483
H	-5.65598708134749	-0.32413160268110	-4.03405975428235

C	-5.64964407260112	0.95893226814352	-2.28643240760998
H	-6.16803715550842	0.20718475242720	-1.65255746940992
H	-4.91048223328545	1.45596546928857	-1.62015714978937
C	-6.68579584403177	1.99368666604263	-2.74769137274446
H	-7.36671958701942	1.52366313615971	-3.49255295441592
H	-7.32728389911640	2.25682449555733	-1.87971074355081
C	-6.08281410072063	3.27433468203120	-3.32868686632676
H	-6.87062812259313	3.99419299467898	-3.62951076289839
H	-5.43328422491446	3.77952304017664	-2.58266561111502
H	-5.46076244518501	3.07268898866054	-4.22483044143107
Si	-3.03403420795423	-5.17008596145004	-3.97550431966968
C	-4.51727647913201	-6.10641376366570	-4.64168698030879
H	-5.06987982958949	-5.51771013784835	-5.40011859656749
H	-4.18463568274270	-7.05225654052848	-5.11682374135856
H	-5.21816928834875	-6.37300437863593	-3.82487970233818
C	-2.18346911541640	-6.04405424177361	-2.55327658614092
C	-1.85195767036179	-4.52056027014082	-5.31924157877386
H	-2.88376357002259	-6.71053176398513	-2.01127225609411
H	-1.35930192430820	-6.67585631268348	-2.94439324215336
H	-1.75143188572361	-5.33904118106141	-1.81294285729294
C	-1.18330784795326	-5.73028267152119	-6.00311012578631
C	-0.78217175415247	-3.64319091423485	-4.64244550242642
C	-2.63628739555603	-3.69240290883054	-6.35400464388154
H	-0.47562390818224	-5.38020057844531	-6.78567890513318
H	-1.92108014062047	-6.39301110788197	-6.50193825949833
H	-0.59766801134436	-6.34504071568329	-5.28848534278324
H	-0.20008808886687	-4.21098878249399	-3.88922421255011
H	-0.06026808093980	-3.26150744247561	-5.39716484265587
H	-1.22072673772182	-2.76190261666964	-4.13194663693699
H	-3.16048714507227	-2.83595811951893	-5.88343055359126
H	-3.39086864904753	-4.30085680748357	-6.89405696669578
H	-1.94134428121377	-3.27635422366585	-7.11548214607526

CIP (with **IDPi 4d**)

H	5.47035693629042	-3.50660571542240	0.51415647406708
C	4.82439204238543	-4.38979368113075	0.60737401526715
C	5.33317165846171	-5.56010155816188	1.15321411425832
C	4.51194653541795	-6.70912874618906	1.29493167798719
C	3.18346290893521	-6.65703865391593	0.90445160597767
C	2.62557386419777	-5.46591567544223	0.35221169126867
C	3.46575432727796	-4.30615957386414	0.17884266907034
C	2.88497613287747	-3.10057927733841	-0.36307334183224
C	1.50361555518864	-3.06120217798139	-0.57796501701444
C	0.66073985730876	-4.21559955561269	-0.45905017713134
C	1.25220570552410	-5.39290094124421	-0.00728677393597
H	6.38171087737003	-5.59359360509691	1.48543246782897
H	4.92782796896665	-7.63228213807596	1.72558181467957
H	2.52904810259013	-7.53454364963390	1.02454133245169
H	0.62387960252527	-6.28839090309352	0.11516235372606
H	7.70235491007705	-0.24449587264488	-2.46383132874556
C	6.97740570638245	-1.07321773375182	-2.43782923760266
C	7.15157557318279	-2.18948599096543	-3.24193638950463
C	6.20101610555942	-3.24407352095926	-3.20324274676441
C	5.10599455416182	-3.17703097220830	-2.35299748174901
C	4.89776343791327	-2.04350984615110	-1.51328315094260
C	5.84895758928192	-0.96274210868457	-1.57195527767147
C	5.63791280044027	0.19168616033709	-0.77146161592911
C	4.47653130727165	0.38028225296500	-0.02348923969354
C	3.51304840501084	-0.68247774284229	-0.04070307795120
C	3.75297771042708	-1.92151839809437	-0.64494747039494
H	8.02017650905688	-2.25781488038236	-3.91409852174640
H	6.33113061825221	-4.12080491591240	-3.85538094369503
H	4.37128776143271	-3.99384076555049	-2.33336127219345
H	6.41909899260178	0.96649828524344	-0.75106826662960
O	2.31442984900163	-0.48108710771422	0.62810048123328

O	0.93655742988332	-1.89495547349965	-1.03699040642309
P	0.89717534162867	-0.45569135145106	-0.20425439071698
N	-0.25834425015943	-0.52060044544527	0.85970254507755
N	0.99076645686074	0.60112899725315	-1.41884979297027
H	-5.27897113699968	-3.71296408679982	1.94107784610620
C	-4.42464120214794	-4.39118174559275	2.06519497471371
C	-4.56450186992238	-5.73707614360078	1.76030546983608
C	-3.46459874156748	-6.62518503809518	1.88701587413916
C	-2.23483237797494	-6.14153891294244	2.30365697442538
C	-2.05494046152441	-4.76119383645412	2.61452946531536
C	-3.17756697050279	-3.86234236374928	2.51044975118169
C	-2.97037521578823	-2.46052746900119	2.78095283629020
C	-1.68043286060032	-2.02284430611000	3.08843510999706
C	-0.56644001241216	-2.90465258922765	3.25125701390431
C	-0.78516334106762	-4.25714774333949	3.00523486272918
H	-5.53480732579481	-6.11608242646726	1.40536896775415
H	-3.58718902188064	-7.69136238266708	1.64365364098024
H	-1.36770547105729	-6.81468805932766	2.39248804387412
H	0.05359939477509	-4.96100078358094	3.11671598929911
H	-8.45437279055337	-0.22723662081520	3.64213222983721
C	-7.60922958595743	-0.89246684844482	3.87844682409100
C	-7.71605048350352	-1.83946376764816	4.88481775015133
C	-6.60752495817117	-2.67222846612217	5.19280253219492
C	-5.42256866450272	-2.56243487624296	4.47951275254988
C	-5.28672142723409	-1.61235527647013	3.42393076679398
C	-6.40081351819275	-0.74552060031477	3.13452112995829
C	-6.26558857220059	0.24352123479291	2.12296970844156
C	-5.06897692028250	0.45911641876787	1.44416717343486
C	-3.96747550014935	-0.40178498490147	1.75852984137137
C	-4.07635393976492	-1.47182962982779	2.65596601958991
H	-8.65211221513248	-1.93919799233429	5.45488031791040
H	-6.68609483787641	-3.40736704076399	6.00800551803300

H	-4.56816863508362	-3.20594624546371	4.73111913272822
H	-7.12578858321794	0.89098024817096	1.89354328234187
O	-2.78705521619464	-0.23833615254507	1.04241174540188
O	-1.46094481689390	-0.64992945693174	3.20349456753894
P	-1.35679843274320	0.17062414542958	1.77189912677404
N	-1.37161272823778	1.70887303136627	2.20577659208686
C	0.77433784704964	-2.42171354166158	3.66867589538036
C	1.92319819569835	-2.87883551922349	2.99727875122250
C	0.92707611594858	-1.56971727234371	4.78538289638330
C	3.19346319023443	-2.46739112822050	3.41956110050975
C	2.19604561043685	-1.18814937555117	5.25665875537294
C	3.32836133197444	-1.63169393977838	4.53508291692633
H	1.81487868045647	-3.54232776499853	2.13072439132633
H	0.02298262848806	-1.22743828267227	5.30362784250004
H	4.08470345249990	-2.81292806920070	2.87400687656097
C	-4.99338146650117	1.50348897351851	0.38769124594364
C	-4.01450740138732	2.51627687587984	0.40554139832764
C	-5.94605871047227	1.49137312786939	-0.65087924518575
C	-4.01212789670616	3.49096873909429	-0.59571793404561
C	-5.95305492773032	2.46045676412306	-1.67474620366168
C	-4.97063689700821	3.47012965790041	-1.62136880731170
H	-3.25463795746447	2.54474486893664	1.19574070793500
H	-6.68115894665852	0.67230677124474	-0.66922018994774
H	-3.23237590139223	4.26597147394299	-0.57050559377793
C	4.29659593227156	1.61565924677259	0.78180116112109
C	3.72240579371490	1.58575186221031	2.06925754385684
C	4.79099298035629	2.84760096603813	0.28855987627521
C	3.66786027849151	2.76091440068243	2.82791306266479
C	4.74277831307489	4.03560660857134	1.03832101003698
C	4.16686683747917	3.96753829939155	2.32547977665738
H	3.32632675456227	0.64847859796028	2.48262638522265
H	5.22144644044975	2.86077667483393	-0.72213236451694

H	3.21953328093068	2.73126361204705	3.83094452968471
C	-0.77322133972861	-4.20327352020259	-0.84860498908707
C	-1.28201974781883	-5.26684493940403	-1.62260304789952
C	-1.65753506420359	-3.18764396882673	-0.43074465875207
C	-2.64398301578746	-5.35954047646459	-1.97436337918582
C	-3.01188233869189	-3.26882353365290	-0.76270107432421
C	-3.50567736863032	-4.34245835393794	-1.51916548902178
H	-0.57824082150360	-6.03760081517249	-1.97122427901939
H	-1.29290262713105	-2.35408354082495	0.17822077113074
H	-3.69634410495803	-2.47743936835432	-0.42207954239607
S	-0.27809210274256	1.16446532091733	-2.23023164957022
S	-0.35980613317918	2.86063988129545	1.73641769328679
O	0.25962731802433	1.78557588699718	-3.48072063205504
O	-1.19592359694783	1.98494945454750	-1.40770579154259
O	0.86215122126046	2.34972582745182	1.05547127636004
O	-1.08820556169886	3.98554821527536	1.10479018623530
C	-1.21673493318867	-0.28223360364688	-2.82247630866059
C	-0.54417923935743	-1.26363628347689	-3.57729439405001
C	-2.61413150274117	-0.38118866073555	-2.67743010231882
C	-1.22936271932348	-2.35834976306356	-4.12064601870651
C	-3.31039119870803	-1.46190182009416	-3.24416631824381
C	-2.62396098405936	-2.43264257042092	-3.98317456191932
C	0.26105363622425	3.58173166954126	3.30236654445046
C	1.02985203896194	4.75446680428971	3.17288809775206
C	0.11437134030732	3.02863519117919	4.58582085118546
C	1.62572890333224	5.37058021323975	4.28051059235819
C	0.68424443435924	3.65583226806356	5.71057637366819
C	1.46069243752932	4.81362765169628	5.55864889940319
F	-0.54699263141940	1.89090799568015	4.80542634926171
F	0.53289833102175	3.12176196954341	6.92662100428455
F	2.03379319301341	5.38119761440012	6.61942167409287
F	2.39456206127806	6.45698768235759	4.11831797358954

F	1.24540895373910	5.31632861431480	1.97774702210749
F	-3.33204685148749	0.51777721472199	-2.01687188389287
F	-4.63153485575297	-1.57659124154985	-3.07136224175859
F	-3.29349301104818	-3.44867076640453	-4.53127128757010
F	-0.57631985676993	-3.30454276415081	-4.80212627972732
F	0.77069208161869	-1.17903086765741	-3.80456406654046
C	0.36261153975506	5.18940790477388	-2.21996797762165
C	1.15043108891067	4.41890728516706	-1.23486031733117
C	0.69070201053745	4.90407233879194	-3.52641968807686
C	2.18284652194586	3.66500517251141	-2.07344998222201
C	1.74774834433723	3.92332102113214	-3.47959286072970
H	0.46644850847750	3.70915918619562	-0.71551401939467
H	2.23875628667940	2.58509755953843	-1.82893778529336
H	1.56780079805592	5.03085415554116	-0.41152854622059
H	3.21006260294329	4.07139732941871	-1.94092673092610
H	-0.44869270421955	5.87375127832080	-1.92544780589781
O	2.26572032403629	3.43668721414244	-4.55017678712287
C	0.02479047949229	5.37040543268259	-4.78655409288328
H	-0.50805834568837	6.32145814938143	-4.57781558712512
H	0.80465647433195	5.59290514713870	-5.54531495397163
C	-0.96448520910800	4.33559895227349	-5.36649489738258
H	-1.38363386109298	4.75246146260912	-6.30907408177591
H	-0.39914260378663	3.42423542590424	-5.65015678457222
C	-2.09461477071075	3.95422803216976	-4.40775781089749
H	-1.65780274248689	3.47909410900892	-3.50549029301171
H	-2.60899067085554	4.87637896486465	-4.05397733218465
C	-3.14238049392041	2.99870542788678	-4.99990541990160
H	-3.83483875071684	2.71576430192244	-4.17949174278060
H	-3.76104160275655	3.53637650931188	-5.75366037753941
C	-2.56792072539523	1.72613028183646	-5.63002282404432
H	-3.37111013385098	0.99995937396763	-5.87162445250738
H	-2.02183935370622	1.93592350303121	-6.57265800055475

H	-1.85151244783525	1.23326808103574	-4.94398391698873
Si	3.20537616310786	1.97350199170501	-4.96967171572367
C	2.08105871645616	1.14626726651922	-6.21375368735423
H	1.74262279591639	1.84847730113028	-7.00026245904484
H	2.59552520953338	0.29094988620226	-6.69690407193342
H	1.19693362908436	0.76422933055177	-5.66670791627481
C	3.56896135877777	0.89263908801444	-3.49915107193514
C	4.78723615369600	2.75716579871971	-5.70271572110952
H	2.64532825057987	0.57267301941721	-2.97705954118306
H	4.08096371514358	-0.02054168236245	-3.86923666924928
H	4.24154016730580	1.36638921820391	-2.76005969272237
C	5.73027104455557	1.60714788244891	-6.11213064088139
C	5.46412184725983	3.62824321487521	-4.62661295903665
C	4.43680890962183	3.61687637711860	-6.93075386904443
H	6.66399145328865	2.01624119986923	-6.55736416058741
H	5.27016694790429	0.94269618982726	-6.87327532229485
H	6.02461655918965	0.97710894056105	-5.24696972295530
H	6.41084654949142	4.06154448082650	-5.01765967463240
H	4.82065202621464	4.47732724834930	-4.31548602022845
H	5.72123298691621	3.04612830377005	-3.71705026555252
H	5.35419843274696	4.08481881142650	-7.35103666804338
H	3.73129222916522	4.43351759878574	-6.67379871558278
H	3.97446769291128	3.01333285030132	-7.73855429631299
C	-6.97458520275260	2.34524205227349	-2.82115240636865
H	-8.66498429385457	3.09102892217609	-1.62705420815901
H	-9.14386854621913	2.13142019668287	-3.06818115739006
H	-8.51096702301602	1.30680615300555	-1.61355551786727
H	-7.71485610700507	3.45904572047712	-4.54264134529043
H	-5.96809227841770	3.69024390276173	-4.25280105937789
H	-7.16817331280316	4.50838586097030	-3.19761591289591
H	-6.65746001181458	0.16962643280182	-3.03589022258484
H	-7.35833828785610	0.96178007208170	-4.48762086793261

H	-5.61755640606287	1.16334332358730	-4.08908805892777
C	-6.94797434863731	3.57221113648136	-3.74965177031466
C	-6.63482916342007	1.08738412440298	-3.65482763967602
C	-8.40210264901667	2.20953012118929	-2.24639519976565
H	-4.93778279492315	4.24984090431875	-2.39474833683972
C	5.30434588097740	5.37384220522855	0.52344339274791
H	6.31754028880188	6.22741615320457	-1.21007991520808
H	6.77095395174135	4.52571218514622	-0.89322573738811
H	5.18477870868744	4.91406319706104	-1.63227820891768
H	3.40376992359572	6.13178402670829	-0.28333578011971
H	4.57657654653439	7.41274711592205	0.16074109339607
H	3.65963213309461	6.54387441901304	1.43477342485835
H	6.83187467426239	6.82352960049277	1.14369586740869
H	5.99631738502931	6.03214296390183	2.51507659164142
H	7.22121453903054	5.12339840365455	1.57748458881422
C	6.40028098414904	5.86438062738605	1.49747479358847
C	4.17178997907008	6.42409292222122	0.46060788655532
C	5.92728539038969	5.24479196415405	-0.87731595639973
H	4.10192825342335	4.87378234687273	2.94592666926562
C	-3.13421881724228	-6.55442717032275	-2.81148381597646
H	-4.90952358434260	-7.25075344601676	-3.86901175914343
H	-5.28665128103694	-6.34635726269260	-2.37508294231265
H	-4.74393683451347	-5.46611474157807	-3.84334629258701
H	-2.27079222298644	-5.76129213984848	-4.67362398621599
H	-2.63741856730909	-7.52234214798026	-4.71718380297571
H	-1.20638044838038	-6.92386018876598	-3.83044943372778
H	-3.62914357383645	-7.75721709360405	-1.03783742782627
H	-3.35926958549364	-8.72309539191313	-2.53110128760230
H	-1.96690601122934	-8.02046836653689	-1.64469688669499
C	-3.01472507169332	-7.83744167377829	-1.95731306705519
C	-2.26152049036414	-6.69566850092905	-4.07958048751579
C	-4.60108032149929	-6.38753789877012	-3.24540472970290

H	-4.57472073213095	-4.36718086907156	-1.76681420501816
C	2.37893308496259	-0.32480358962874	6.51825683934660
H	3.48834520280854	-0.40971271542237	8.40567728178161
H	2.96573110472035	-2.01614691109576	7.79733662723225
H	4.35568971398355	-1.17655053701190	7.04197461087031
H	2.28670905975977	1.53612352489230	5.37614188887805
H	3.95245183021718	0.93342046583556	5.60839888512210
H	3.08071075266234	1.71055887100444	6.97434222523419
H	0.31696144769270	0.45636513662359	6.63959148196945
H	0.59142841880984	-1.05154183062851	7.57475394035575
H	1.23000684511840	0.51292822691612	8.16932321764742
C	3.35470088397744	-1.02398150354820	7.49132776345857
C	1.05020000897120	-0.09298371412411	7.25894754743882
C	2.96388837703462	1.04059150401781	6.09769651899240
H	4.33682985029621	-1.33121020194124	4.85783696799968

9. ECD Experiments for Determination of the Absolute Configuration

Method:
The ECD-spectra of **3b** (1 mg/mL) was recorded in hexanes (HPLC grade) at 20 °C and compared with the corresponding TD-DFT calculated ECD spectra of the possible conformers. After a correction of the σ-value of 0.3 eV, the ECD characteristics of the calculated spectrum (blue curve) was in good agreement with the experimental spectrum (red curve), thus allowing the assignment of the absolute configuration of **3b**, and the absolute configuration of **3b** could be assigned to be *R* using (*S*,*S*)-IDPi **4f** as catalyst (Fig. S11).

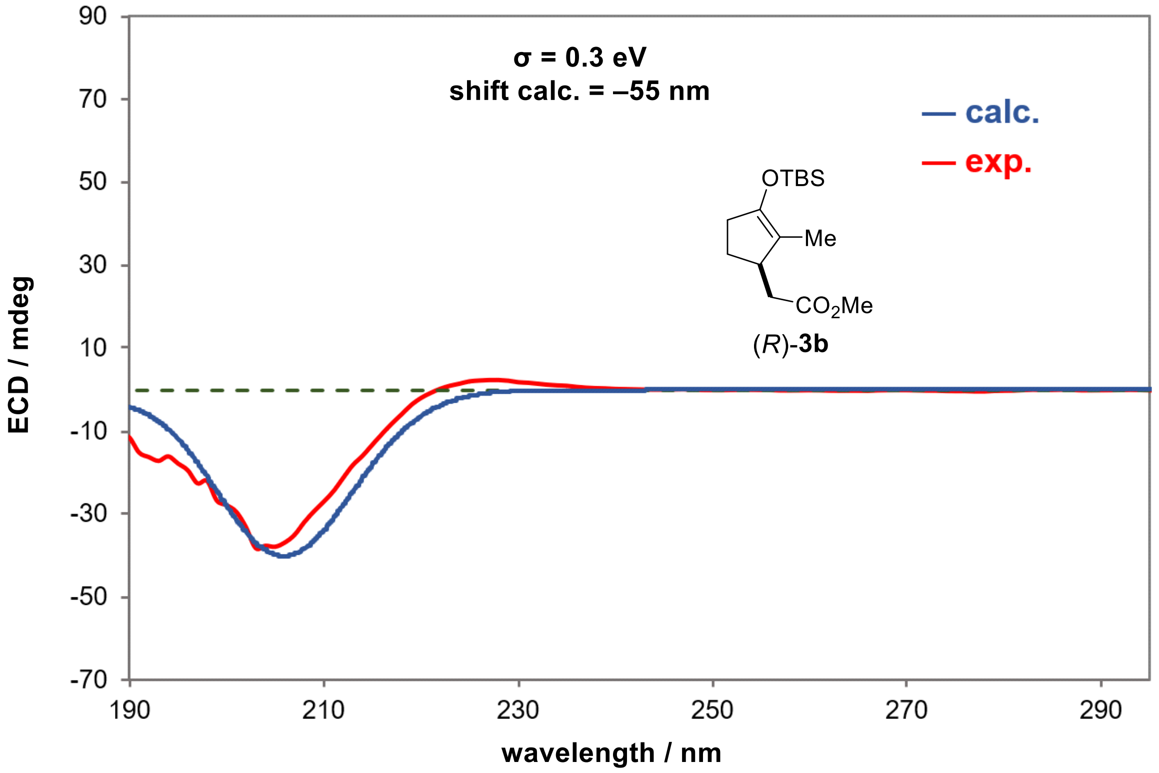

Figure S11. Comparison between the experimental ECD-spectrum (red curve) and the calculated spectrum (blue curve).

ECD spectrum calculation

Density Functional Theory (DFT) calculations were performed on the Max-Planck-Institut für Kohlenforschung computer cluster using the ORCA program package (Version 5.0-Stable).[20] Structural optimizations and frequency calculations to identify all of the stationary points as minima (zero imaginary frequencies) and to obtain thermal and entropic correction were performed with the PBE functional[21,22] with D3 dispersion correction[11] and Becke-Johnson damping (BJ)[12] along with RI approximation, utilizing the def2/J auxiliary basis set[23] and the def2-SVP basis set[13] on all atoms. The libint2 library was used for the computation of 2-el integrals.[24] Tight SCF convergence and geometry optimization criterions were chosen.

The ECD spectrum was computed by time-dependent density functional theory (TD-DFT, NROOTS = 25) at apfd/6-311+g(2d,p) level, solvent effects of hexane were taken into account using the conductor-like polarized continuum model (CPCM).[14] The ECD spectrum was created using Multiwfn[25] with a half-width at full-height. Starting from the initial guess structures obtained in the first step, molecular dynamics (MD) simulations with xTB 6.3[26] employing GFN2-xTB method and the following data processing with Molclus[27] were performed to sample all the possible conformers of the compound.

Calculated coordinates:

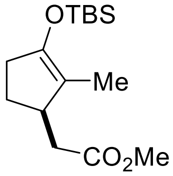

C	2.34676900	-1.44028900	1.48404400
C	0.86777100	-1.01359600	1.59906000
C	0.31006800	-1.38030100	0.24332900
C	1.26446900	-1.66726200	-0.66239000
C	1.04305300	-1.95353700	-2.11188500
H	1.38254200	-1.10439600	-2.73191500
H	1.60198400	-2.84649000	-2.44362100
H	-0.02518700	-2.11488000	-2.31709900
C	2.64027900	-1.57231400	-0.03923900
C	3.51065500	-0.42527800	-0.62939300
C	2.84387200	0.91961100	-0.49366500
O	2.07952800	1.40225700	-1.29596500
O	3.15740600	1.51824000	0.67614400
C	2.52523900	2.77128300	0.94339900
H	3.00701800	3.17300600	1.84368600
H	1.44824000	2.63163200	1.12262700
H	2.64923900	3.46335500	0.09704000
H	3.66958400	-0.60412700	-1.70256800
H	4.48689100	-0.40924600	-0.12223300
H	3.21531100	-2.49593900	-0.23625800

O	-1.01573800	-1.42017200	-0.01016400
Si	-2.36759900	-0.50096000	0.42756000
C	-2.40743300	-0.22107000	2.29032800
H	-2.36434200	-1.18359600	2.82537600
H	-1.56955700	0.39995600	2.64065400
H	-3.34503300	0.28225500	2.57906000
C	-3.81859100	-1.56781000	-0.09587300
H	-3.79658800	-2.53056800	0.43978300
H	-4.77866600	-1.07482800	0.12658500
H	-3.78346900	-1.78175500	-1.17549400
C	-2.31287100	1.15201900	-0.52934500
C	-3.60694300	1.94573700	-0.26067700
H	-3.73115200	2.18720700	0.80857200
H	-4.50632800	1.39838900	-0.58883600
H	-3.58955100	2.90428900	-0.81064900
C	-2.18203700	0.86217600	-2.03801000
H	-1.26496200	0.29528000	-2.26224600
H	-3.04054700	0.28863200	-2.42523900
H	-2.13512500	1.80832800	-2.60698000
C	-1.09403000	1.97684600	-0.07335400
H	-0.14701800	1.46843200	-0.30585100
H	-1.12240100	2.19816100	1.00725500
H	-1.06650400	2.94618400	-0.60369300
H	0.75760700	0.07290300	1.77558500
H	0.33416500	-1.51863800	2.42114200
H	2.48011300	-2.42366600	1.95989300
H	3.02892200	-0.73633700	1.98149500

10. Crystallographic Data of 7

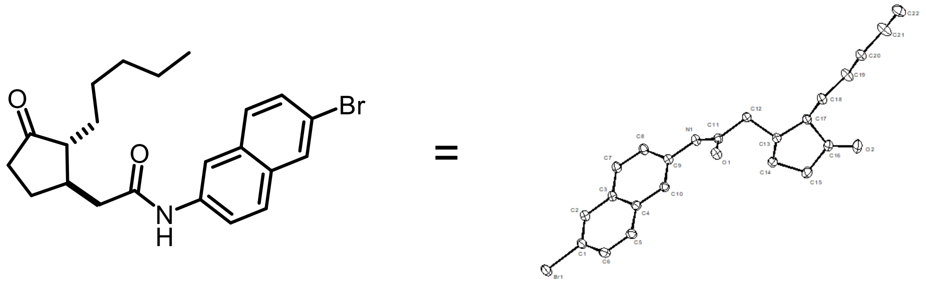

Crystal data and structure refinement.

Identification code	15916	
Empirical formula	$C_{22}H_{26}BrNO_2$	
Color	colourless	
Formula weight	416.361 g·mol^{-1}	
Temperature	100(2) K	
Wavelength	0.71073 Å	
Crystal system	Monoclinic	
Space group	$P2_1$, (no. 4)	
Unit cell dimensions	a = 10.9454(4) Å	α= 90°.
	b = 8.6061(3) Å	β= 111.648(2)°.
	c = 11.2666(4) Å	γ = 90°.
Volume	986.43(6) Å^3	
Z	2	
Density (calculated)	1.402 Mg·m^{-3}	
Absorption coefficient	2.099 mm^{-1}	
F(000)	431.832 e	
Crystal size	0.4 x 0.171 x 0.05 mm^3	
θ range for data collection	2.22 to 33.26°.	
Index ranges	$-16 \le h \le 16, -13\le k \le 13, -17 \le l \le 17$	
Reflections collected	296159	
Independent reflections	7571 [R_{int} = 0.0859]	
Reflections with $I>2\sigma(I)$	7182	
Completeness to θ = 25.2417°	99.74%	
Absorption correction	Numerical	
Max. and min. transmission	0.9637 and 0.5924	
Refinement method	Full-matrix least-squares on F^2	
Data / restraints / parameters	7571 / 1 / 469	
Goodness-of-fit on F^2	1.0690	
Final R indices [$I>2\sigma(I)$]	R_1 = 0.0290	wR^2 = 0.0737
R indices (all data)	R_1 = 0.0317	wR^2 = 0.0756
Absolute structure parameter	-0.0124(15)	
Largest diff. peak and hole	0.5295 and -0.2387 e·Å^{-3}	

Bond lengths [Å] and angles [°].

Br(1)-C(1)	1.8888(12)	O(1)-C(11)	1.2330(18)
O(2)-C(16)	1.2084(18)	N(1)-H(1)	0.97(3)
N(1)-C(9)	1.4069(17)	N(1)-C(11)	1.3547(17)
C(1)-C(2)	1.3702(19)	C(1)-C(6)	1.413(2)
C(2)-H(2)	1.08(2)	C(2)-C(3)	1.4180(18)
C(3)-C(4)	1.4198(17)	C(3)-C(7)	1.4187(19)
C(4)-C(5)	1.4221(18)	C(4)-C(10)	1.4195(16)
C(5)-H(5)	1.09(3)	C(5)-C(6)	1.375(2)
C(6)-H(6)	1.07(3)	C(7)-H(7)	1.07(2)
C(7)-C(8)	1.3679(19)	C(8)-H(8)	1.07(2)
C(8)-C(9)	1.4217(18)	C(9)-C(10)	1.3780(18)
C(10)-H(10)	1.07(2)	C(11)-C(12)	1.5129(18)
C(12)-H(12a)	1.08(3)	C(12)-H(12b)	1.10(3)
C(12)-C(13)	1.5313(18)	C(13)-H(13)	1.07(3)
C(13)-C(14)	1.5356(18)	C(13)-C(17)	1.5387(18)
C(14)-H(14a)	1.07(2)	C(14)-H(14b)	1.10(2)
C(14)-C(15)	1.5281(19)	C(15)-H(15a)	1.10(3)
C(15)-H(15b)	1.11(3)	C(15)-C(16)	1.526(2)
C(16)-C(17)	1.5258(19)	C(17)-H(17)	1.12(3)
C(17)-C(18)	1.5220(18)	C(18)-H(18a)	1.10(3)
C(18)-H(18b)	1.08(3)	C(18)-C(19)	1.520(2)
C(19)-H(19a)	1.08(3)	C(19)-H(19b)	1.08(3)
C(19)-C(20)	1.521(2)	C(20)-H(20a)	1.09(3)
C(20)-H(20b)	1.09(2)	C(20)-C(21)	1.517(2)
C(21)-H(21a)	1.07(3)	C(21)-H(21b)	1.10(3)
C(21)-C(22)	1.519(2)	C(22)-H(22a)	1.11(3)
C(22)-H(22b)	1.07(3)	C(22)-H(22c)	1.05(4)
C(9)-N(1)-H(1)	115.8(14)	C(11)-N(1)-H(1)	116.8(14)
C(11)-N(1)-C(9)	127.35(12)	C(2)-C(1)-Br(1)	119.08(11)

C(6)-C(1)-Br(1)	119.18(10)	C(6)-C(1)-C(2)	121.74(12)
H(2)-C(2)-C(1)	120.3(13)	C(3)-C(2)-C(1)	119.39(12)
C(3)-C(2)-H(2)	120.3(13)	C(4)-C(3)-C(2)	119.92(11)
C(7)-C(3)-C(2)	121.46(12)	C(7)-C(3)-C(4)	118.62(11)
C(5)-C(4)-C(3)	118.53(11)	C(10)-C(4)-C(3)	119.99(11)
C(10)-C(4)-C(5)	121.48(11)	H(5)-C(5)-C(4)	118.2(14)
C(6)-C(5)-C(4)	121.02(12)	C(6)-C(5)-H(5)	120.8(14)
C(5)-C(6)-C(1)	119.27(12)	H(6)-C(6)-C(1)	119.8(15)
H(6)-C(6)-C(5)	120.9(15)	H(7)-C(7)-C(3)	118.5(16)
C(8)-C(7)-C(3)	120.86(12)	C(8)-C(7)-H(7)	120.5(16)
H(8)-C(8)-C(7)	119.4(14)	C(9)-C(8)-C(7)	120.35(12)
C(9)-C(8)-H(8)	120.2(13)	C(8)-C(9)-N(1)	115.43(11)
C(10)-C(9)-N(1)	124.19(11)	C(10)-C(9)-C(8)	120.35(11)
C(9)-C(10)-C(4)	119.79(11)	H(10)-C(10)-C(4)	118.2(14)
H(10)-C(10)-C(9)	122.0(14)	N(1)-C(11)-O(1)	123.29(11)
C(12)-C(11)-O(1)	121.59(12)	C(12)-C(11)-N(1)	115.10(13)
H(12a)-C(12)-C(11)	112.5(14)	H(12b)-C(12)-C(11)	108.4(14)
H(12b)-C(12)-H(12a)	107(2)	C(13)-C(12)-C(11)	109.33(10)
C(13)-C(12)-H(12a)	109.8(14)	C(13)-C(12)-H(12b)	109.6(14)
H(13)-C(13)-C(12)	110.0(13)	C(14)-C(13)-C(12)	112.67(10)
C(14)-C(13)-H(13)	108.6(14)	C(17)-C(13)-C(12)	114.19(10)
C(17)-C(13)-H(13)	107.6(12)	C(17)-C(13)-C(14)	103.45(10)
H(14a)-C(14)-C(13)	112.0(15)	H(14b)-C(14)-C(13)	110.4(13)
H(14b)-C(14)-H(14a)	107(2)	C(15)-C(14)-C(13)	104.29(11)
C(15)-C(14)-H(14a)	112.6(15)	C(15)-C(14)-H(14b)	110.7(12)
H(15a)-C(15)-C(14)	111.0(16)	H(15b)-C(15)-C(14)	115.9(15)
H(15b)-C(15)-H(15a)	107(3)	C(16)-C(15)-C(14)	104.96(11)
C(16)-C(15)-H(15a)	108.0(17)	C(16)-C(15)-H(15b)	110.0(17)
C(15)-C(16)-O(2)	124.58(14)	C(17)-C(16)-O(2)	126.51(14)
C(17)-C(16)-C(15)	108.82(11)	C(16)-C(17)-C(13)	101.50(10)

H(17)-C(17)-C(13)	106.0(14)	H(17)-C(17)-C(16)	105.7(15)
C(18)-C(17)-C(13)	116.17(11)	C(18)-C(17)-C(16)	115.65(11)
C(18)-C(17)-H(17)	110.7(14)	H(18a)-C(18)-C(17)	108.9(14)
H(18b)-C(18)-C(17)	108.3(13)	H(18b)-C(18)-H(18a)	103(2)
C(19)-C(18)-C(17)	115.30(11)	C(19)-C(18)-H(18a)	112.2(13)
C(19)-C(18)-H(18b)	108.3(14)	H(19a)-C(19)-C(18)	109.1(16)
H(19b)-C(19)-C(18)	105.7(17)	H(19b)-C(19)-H(19a)	107(3)
C(20)-C(19)-C(18)	112.75(12)	C(20)-C(19)-H(19a)	111.6(19)
C(20)-C(19)-H(19b)	110.2(16)	H(20a)-C(20)-C(19)	108.9(16)
H(20b)-C(20)-C(19)	108.0(14)	H(20b)-C(20)-H(20a)	107(2)
C(21)-C(20)-C(19)	114.48(13)	C(21)-C(20)-H(20a)	108.2(16)
C(21)-C(20)-H(20b)	110.1(15)	H(21a)-C(21)-C(20)	109.2(19)
H(21b)-C(21)-C(20)	107.6(19)	H(21b)-C(21)-H(21a)	107(3)
C(22)-C(21)-C(20)	113.73(14)	C(22)-C(21)-H(21a)	109(2)
C(22)-C(21)-H(21b)	110.3(18)	H(22a)-C(22)-C(21)	109.0(18)
H(22b)-C(22)-C(21)	116(2)	H(22b)-C(22)-H(22a)	106(3)
H(22c)-C(22)-C(21)	108.5(19)	H(22c)-C(22)-H(22a)	108(3)
H(22c)-C(22)-H(22b)	109(3)		

11. ^{1}H , ^{13}C, ^{31}P and ^{19}F NMR Spectra of Substrates and Products

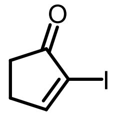

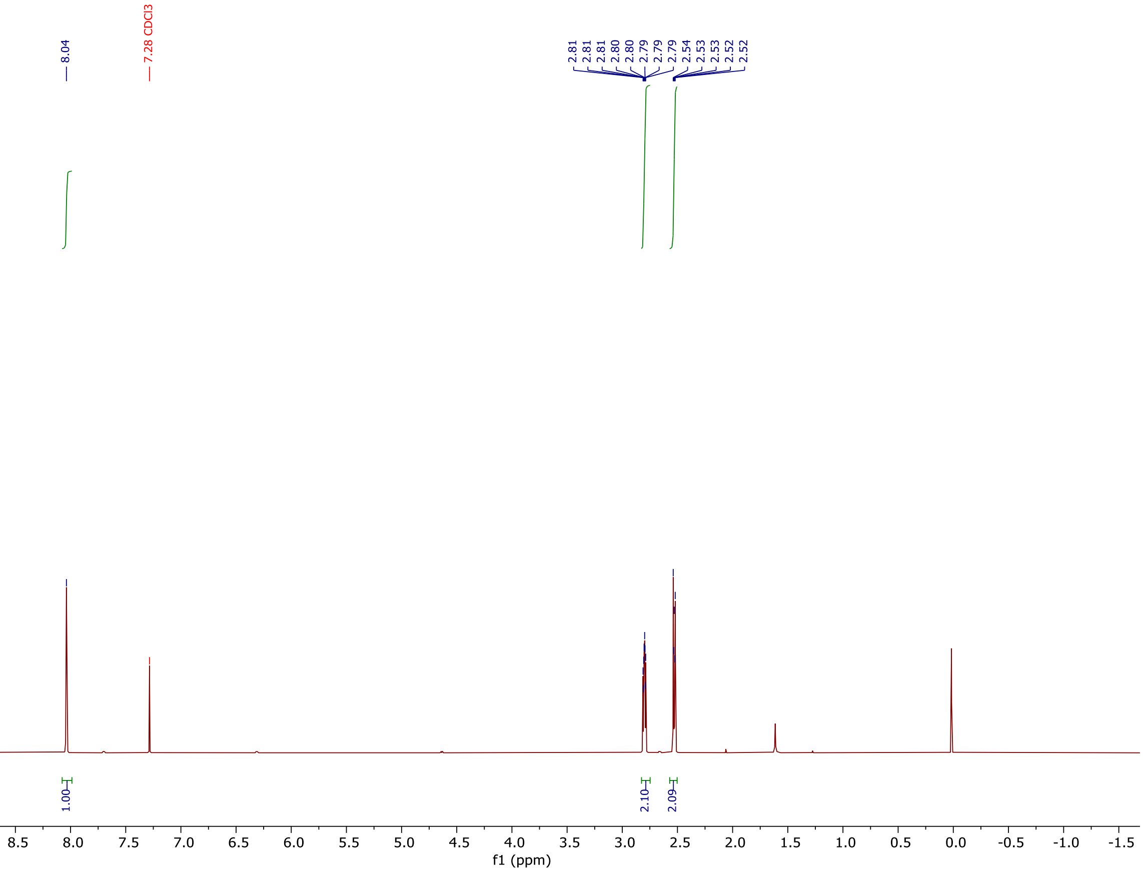

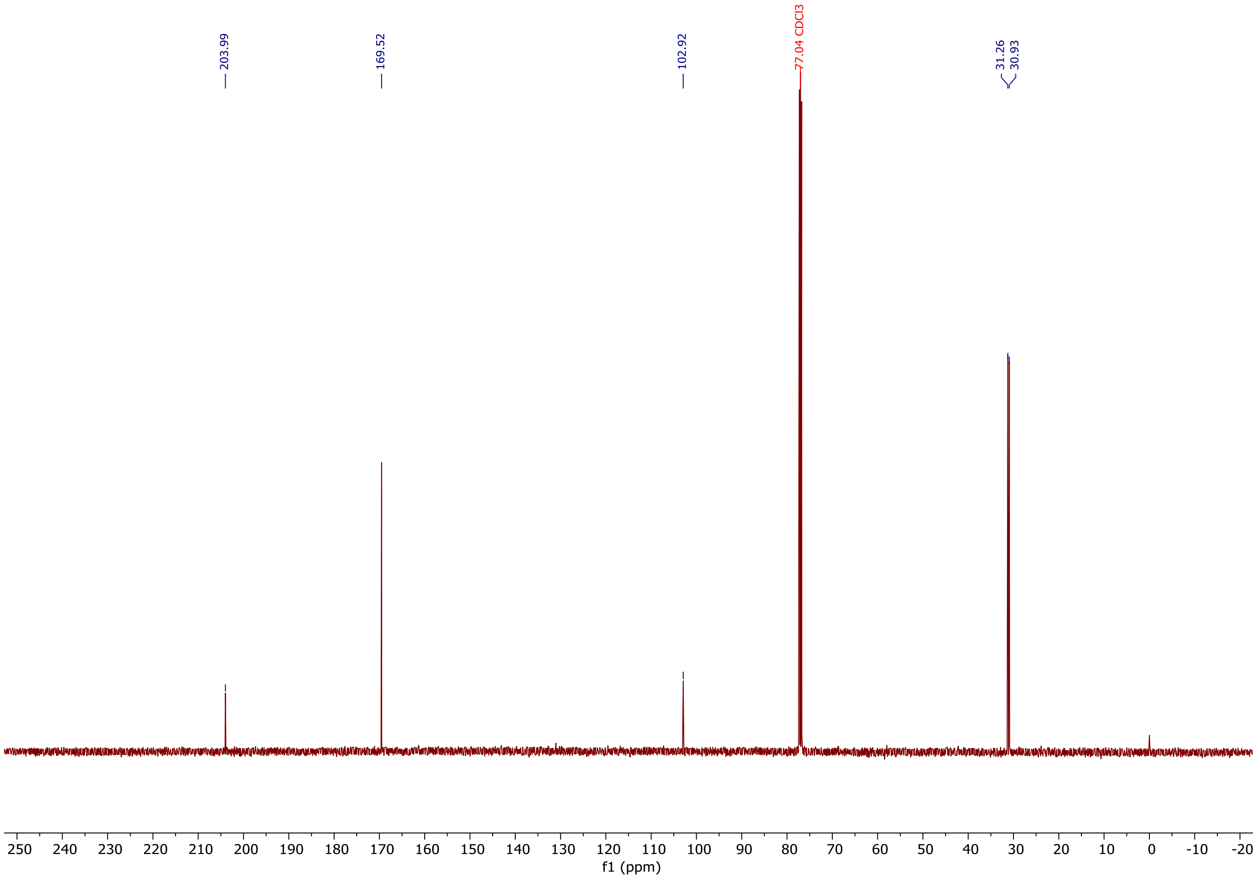

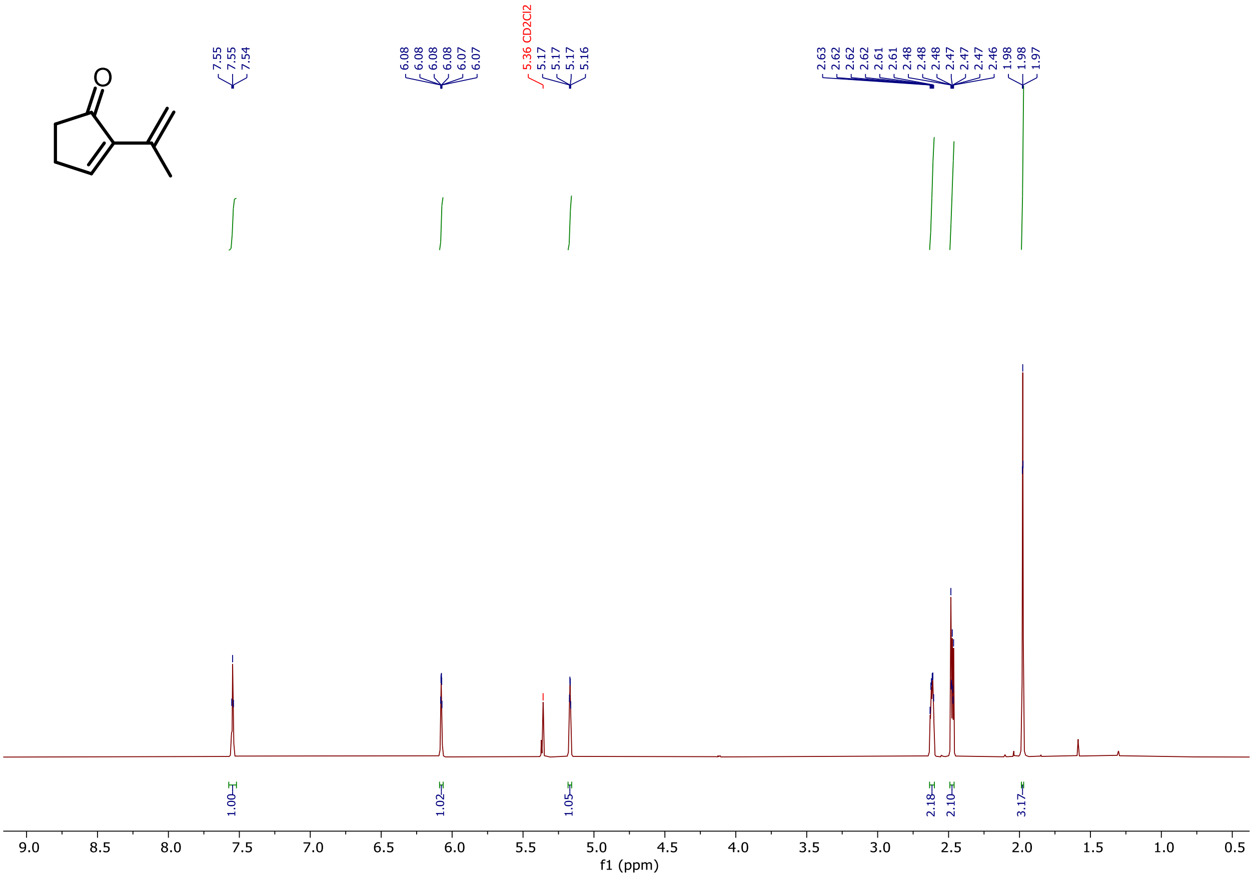
7.55
7.55
7.54
6.08
6.08
6.08
6.08
6.07
6.07
5.36 CD2Cl2
5.17
5.17
5.17
5.16
2.63
2.62
2.62
2.62
2.61
2.61
2.48
2.48
2.47
2.47
2.47
2.46
1.98
1.98
1.97
1.00
1.02
1.05
2.18
2.10
3.17
9.0 8.5 8.0 7.5 7.0 6.5 6.0 5.5 5.0 4.5 4.0 3.5 3.0 2.5 2.0 1.5 1.0 0.5
f1 (ppm)

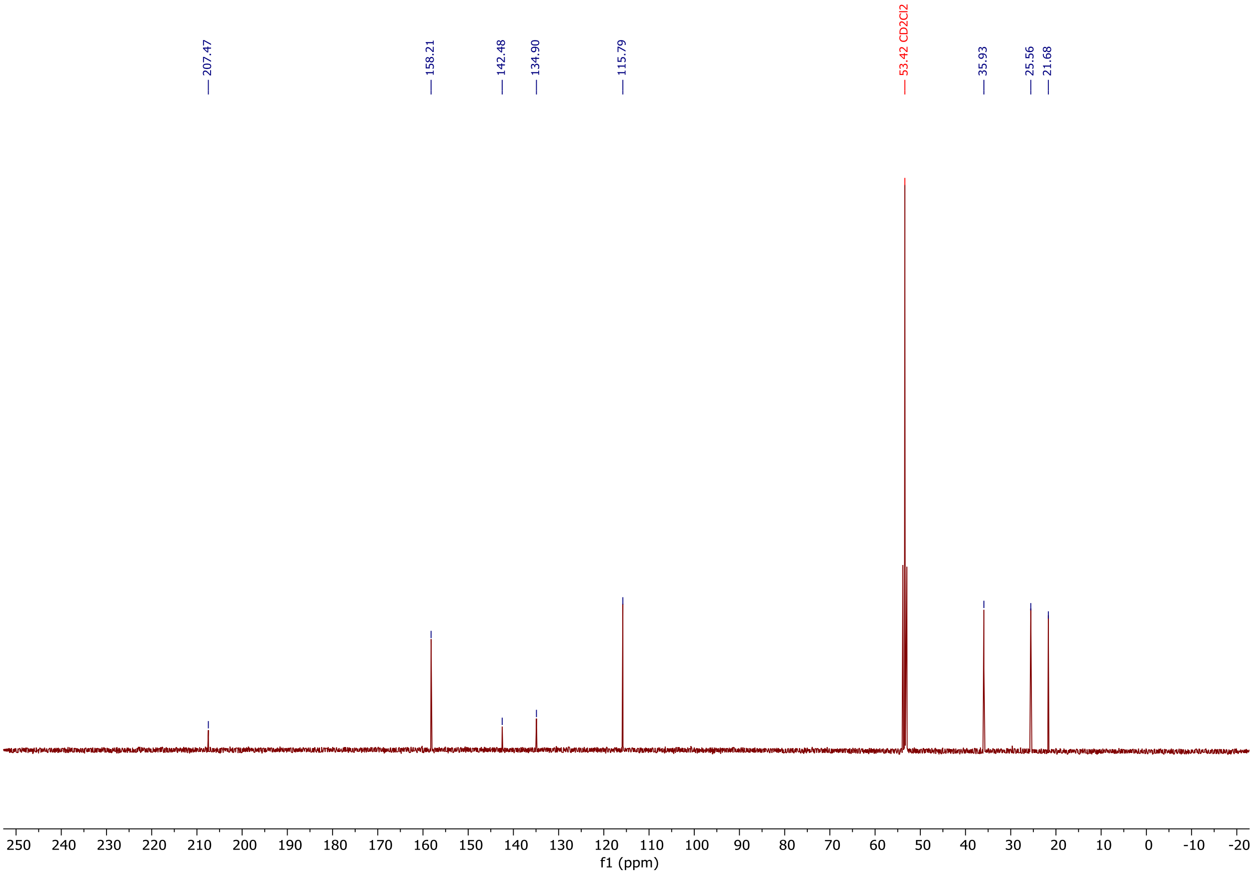
207.47
158.21
142.48
134.90
115.79
53.42 CD2Cl2
35.93
25.56
21.68
250 240 230 220 210 200 190 180 170 160 150 140 130 120 110 100 90 80 70 60 50 40 30 20 10 0 -10 -20
f1 (ppm)

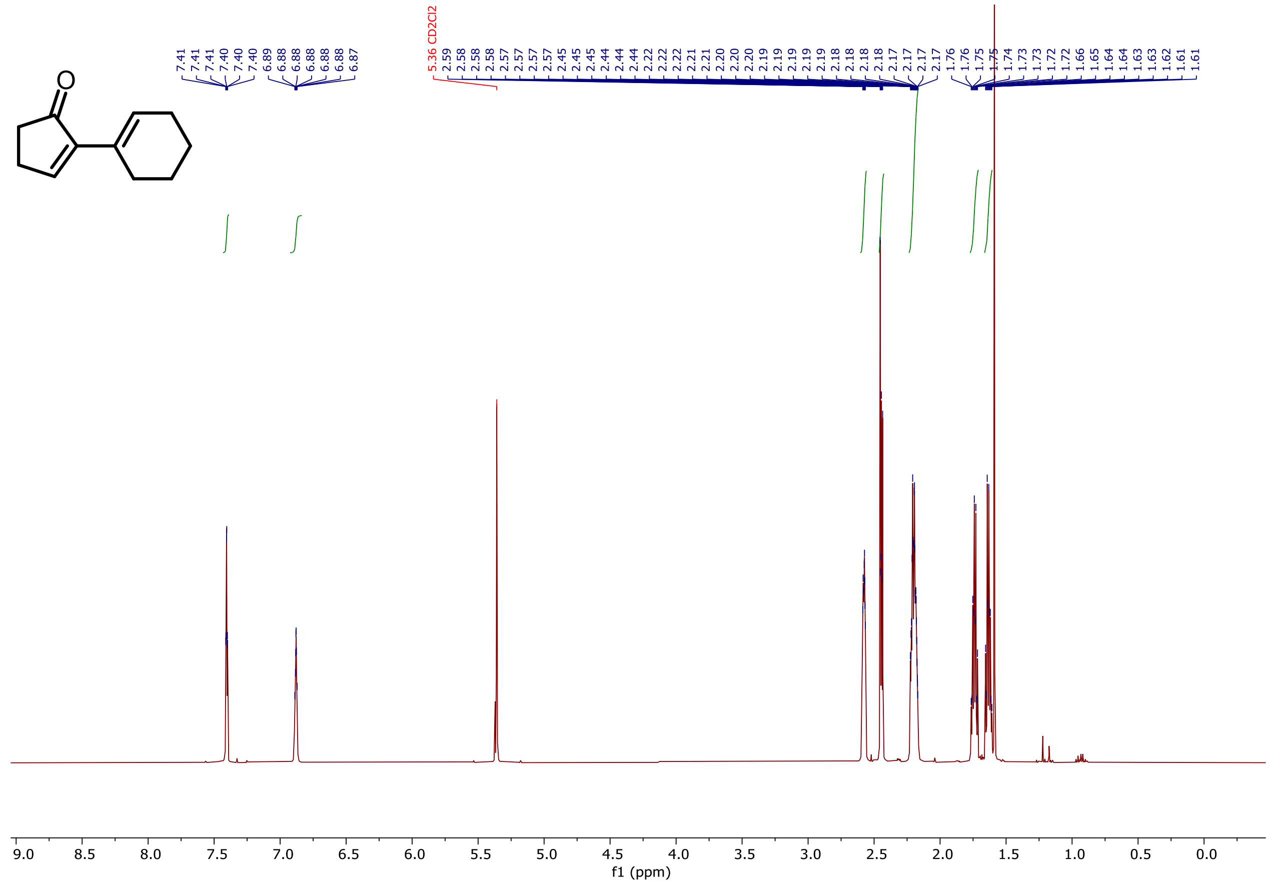
7.41
7.41
7.41
7.40
7.40
7.40
6.89
6.88
6.88
6.88
6.88
6.88
6.87
5.36 CD2Cl2
2.59
2.58
2.58
2.58
2.57
2.57
2.57
2.57
2.45
2.45
2.45
2.44
2.44
2.44
2.22
2.22
2.22
2.21
2.21
2.20
2.20
2.20
2.19
2.19
2.19
2.19
2.19
2.18
2.18
2.18
2.17
2.17
2.17
2.17
1.76
1.75
1.75
1.74
1.73
1.73
1.72
1.72
1.66
1.65
1.64
1.64
1.63
1.62
1.61
1.61
9.0
8.5
8.0
7.5
7.0
6.5
6.0
5.5
5.0
4.5
4.0
3.5
3.0
2.5
2.0
1.5
1.0
0.5
0.0
f1 (ppm)

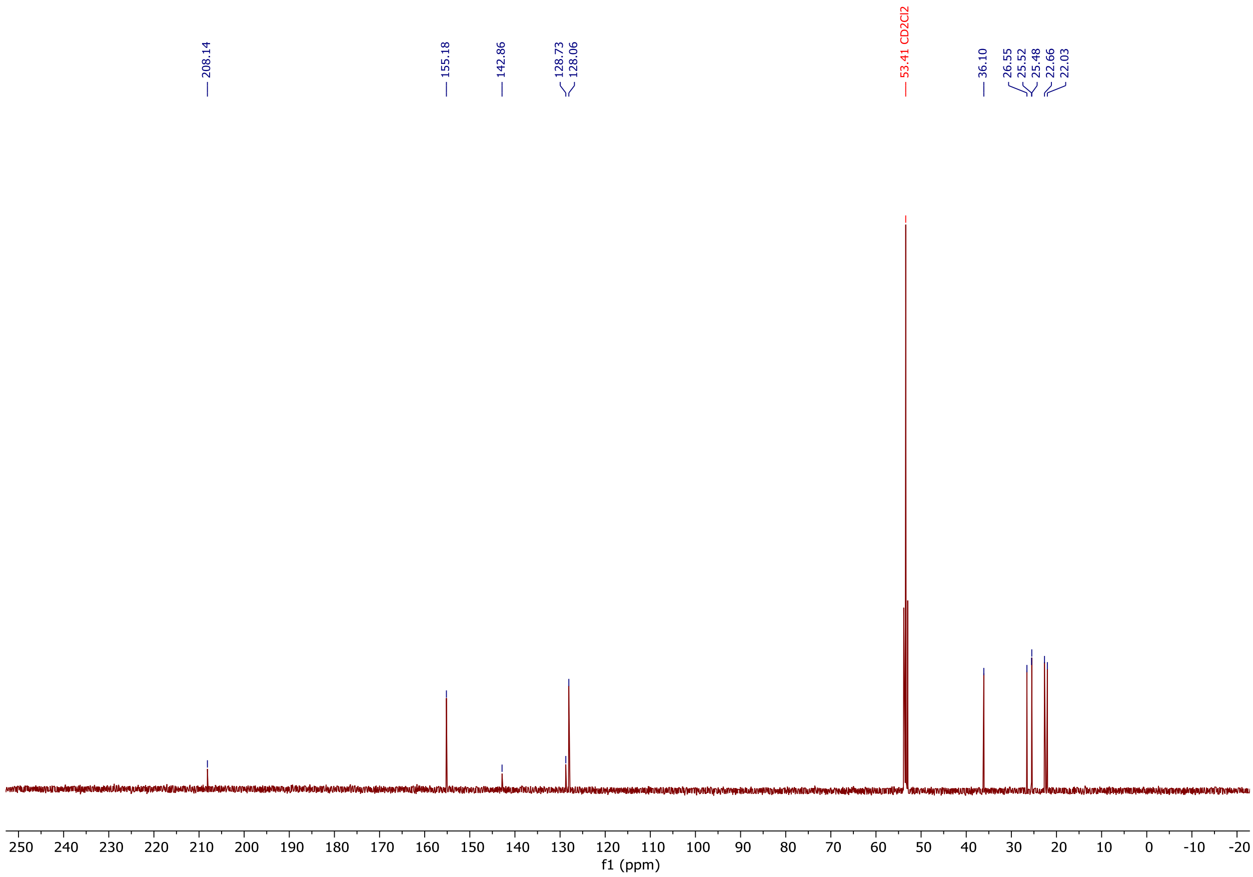
208.14
155.18
142.86
128.73
128.06
53.41 CD2Cl2
36.10
26.55
25.52
25.48
22.66
22.03
250
240
230
220
210
200
190
180
170
160
150
140
130
120
110
100
90
80
70
60
50
40
30
20
10
0
-10
-20
f1 (ppm)

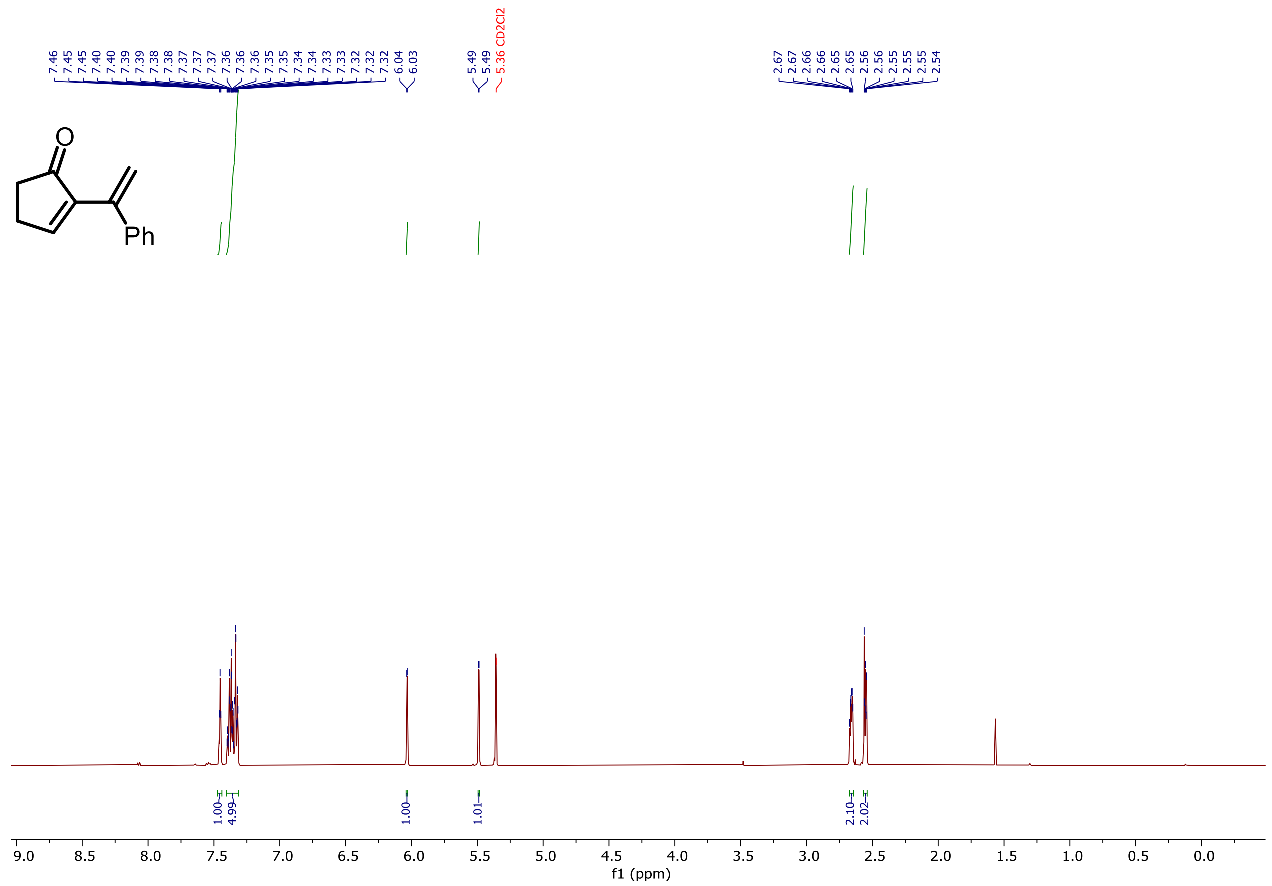
7.46 7.45 7.45 7.40 7.40 7.39 7.39 7.38 7.38 7.37 7.37 7.36 7.36 7.36 7.35 7.35 7.34 7.34 7.33 7.33 7.32 7.32 7.32 6.04 6.03
5.49 5.49 5.36 CD2Cl2
2.67 2.67 2.66 2.66 2.65 2.65 2.56 2.56 2.55 2.55 2.55 2.54
O
Ph
1.00 4.99 1.00 1.01 2.10 2.02
9.0 8.5 8.0 7.5 7.0 6.5 6.0 5.5 5.0 4.5 4.0 3.5 3.0 2.5 2.0 1.5 1.0 0.5 0.0
f1 (ppm)

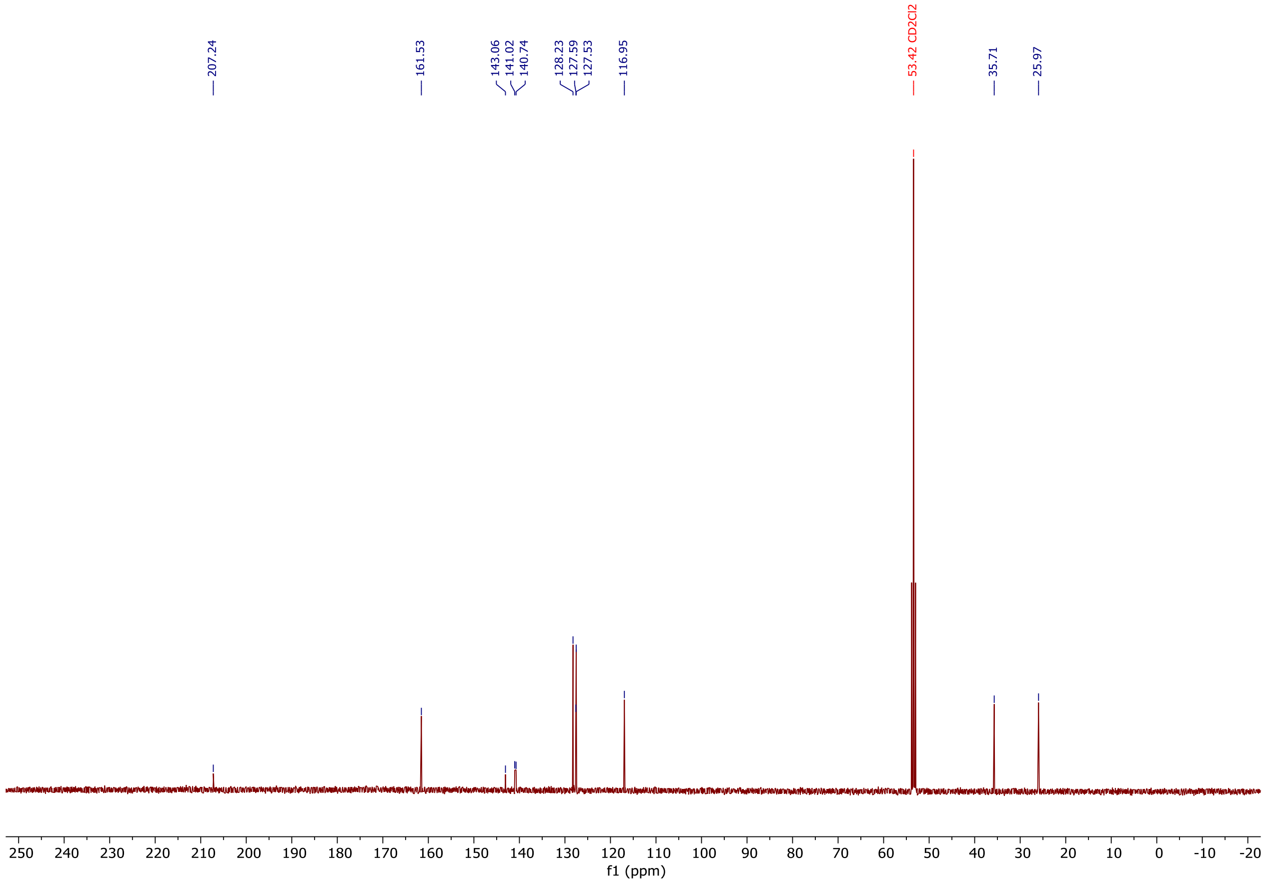
207.24
161.53
143.06 141.02 140.74
128.23 127.59 127.53
116.95
53.42 CD2Cl2
35.71
25.97
250 240 230 220 210 200 190 180 170 160 150 140 130 120 110 100 90 80 70 60 50 40 30 20 10 0 -10 -20
f1 (ppm)

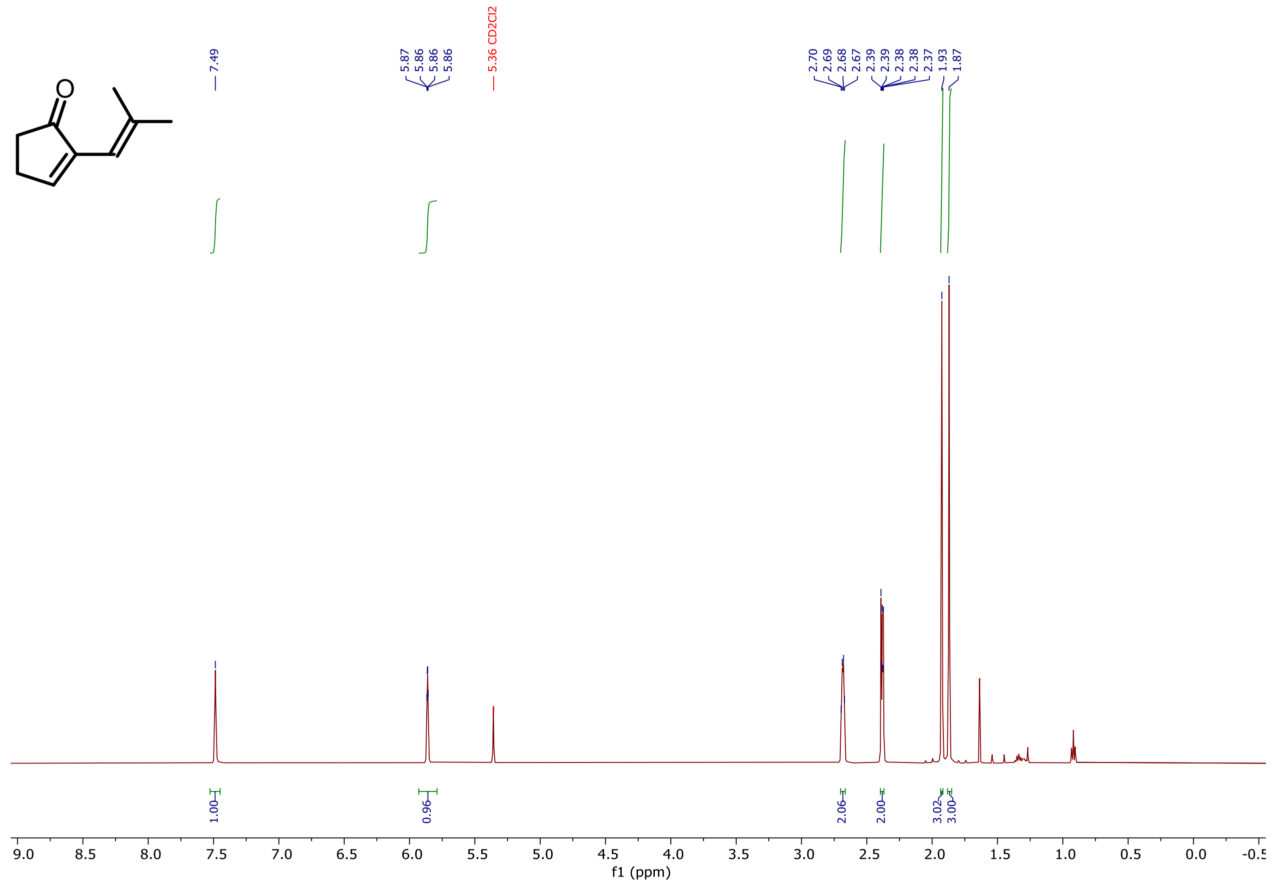

7.49
5.87
5.86
5.86
5.86
5.36 CD2Cl2
2.70
2.69
2.68
2.67
2.39
2.39
2.38
2.38
2.37
1.93
1.87
1.00
0.96
2.06
2.00
3.02
3.00
9.0 8.5 8.0 7.5 7.0 6.5 6.0 5.5 5.0 4.5 4.0 3.5 3.0 2.5 2.0 1.5 1.0 0.5 0.0 -0.5
f1 (ppm)

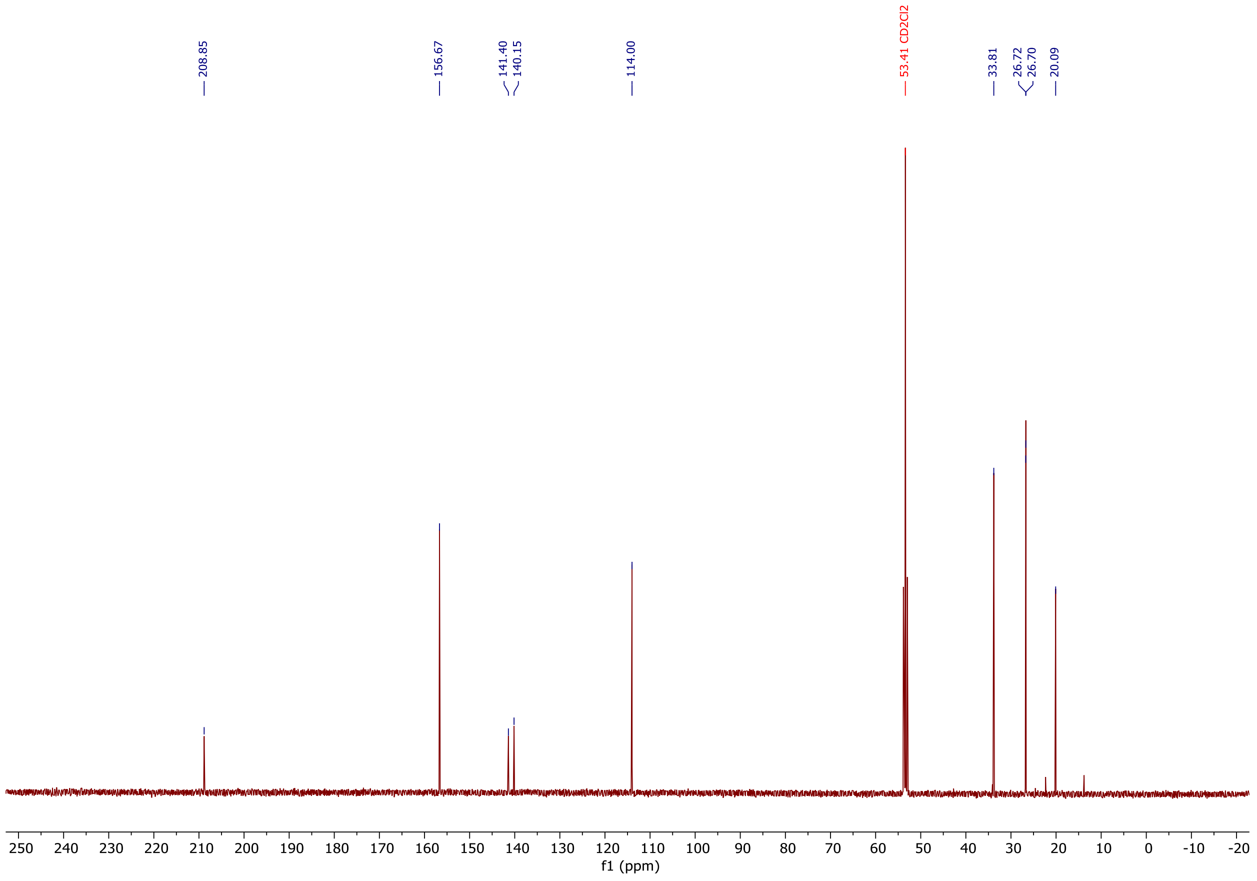

208.85
156.67
141.40
140.15
114.00
53.41 CD2Cl2
33.81
26.72
26.70
20.09
250 240 230 220 210 200 190 180 170 160 150 140 130 120 110 100 90 80 70 60 50 40 30 20 10 0 -10 -20
f1 (ppm)

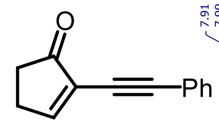

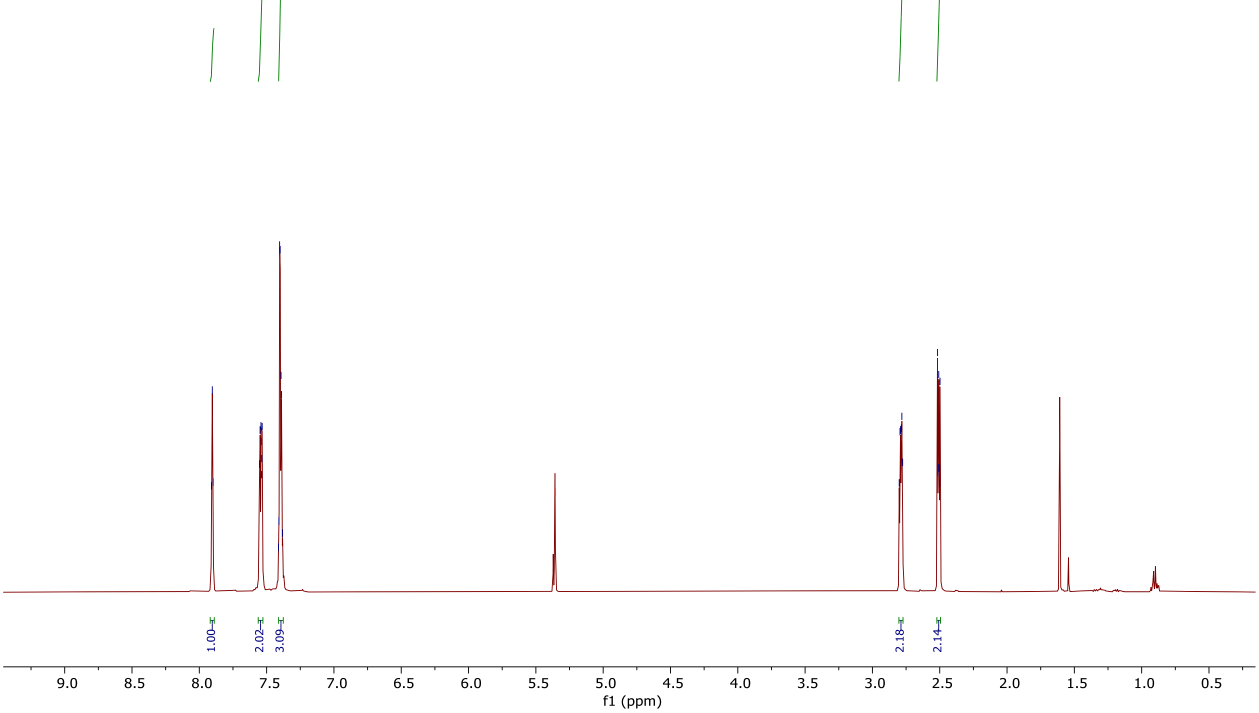
7.91
7.90
7.90
7.55
7.55
7.54
7.54
7.54
7.54
7.53
7.41
7.40
7.40
7.39
7.39
7.38
5.36 CD2Cl2
2.80
2.79
2.79
2.79
2.78
2.78
2.52
2.51
2.51
2.51
2.50
2.50
2.50
1.00
2.02
3.09
2.18
2.14
f1 (ppm)

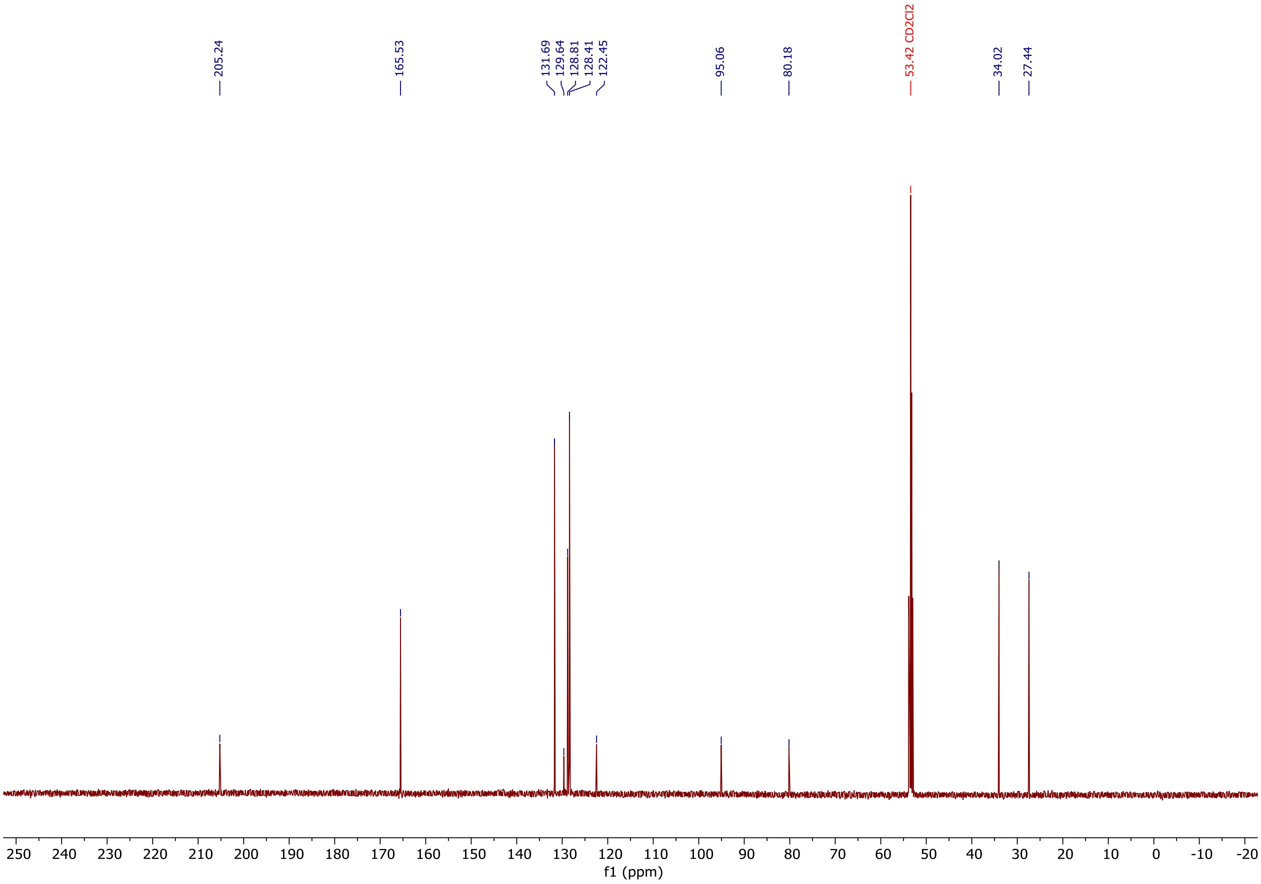
205.24
165.53
131.69
129.64
128.81
128.41
122.45
95.06
80.18
53.42 CD2Cl2
34.02
27.44
f1 (ppm)

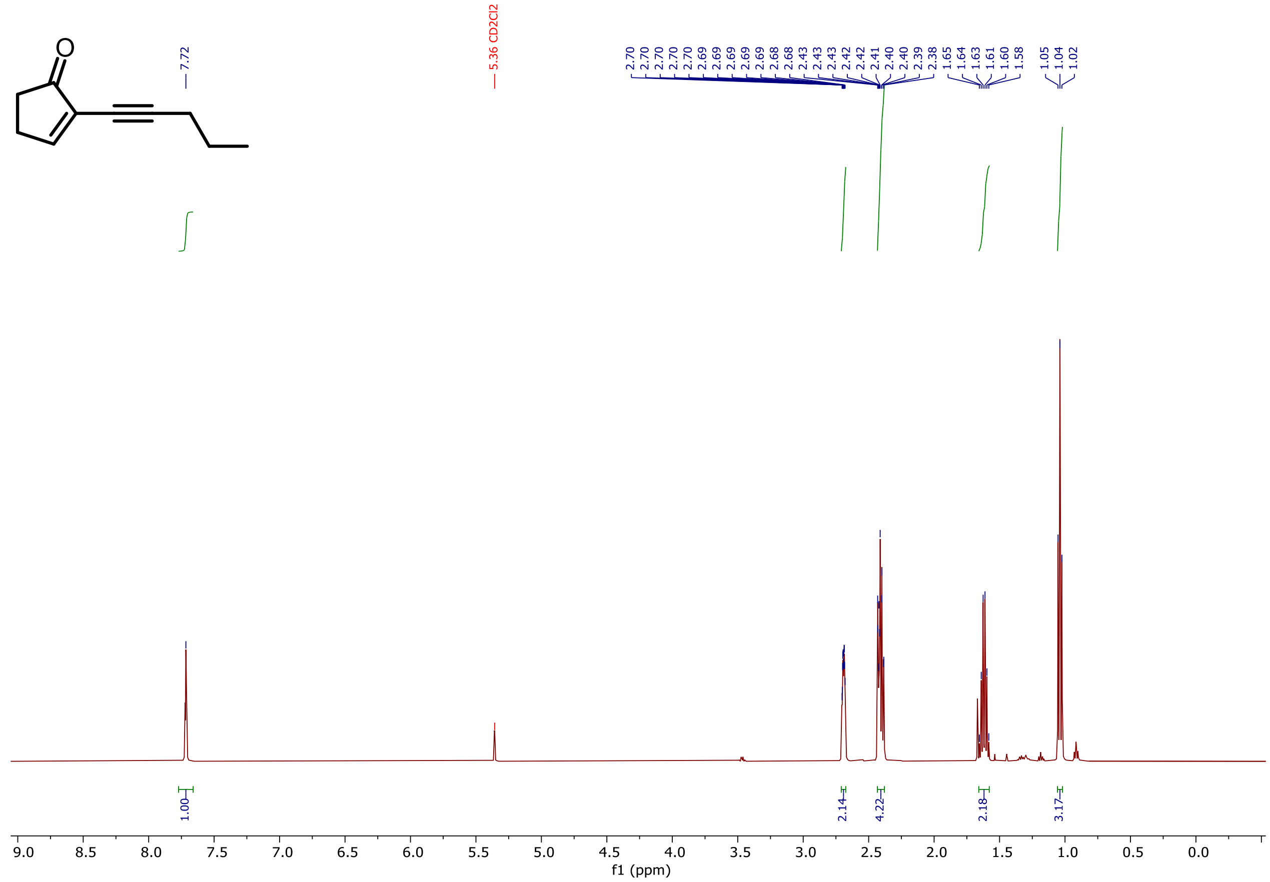

7.72
5.36 CD2Cl2
2.70 2.70 2.70 2.70 2.70 2.69 2.69 2.69 2.69 2.69 2.68 2.43 2.43 2.43 2.42 2.42 2.41 2.40 2.40 2.39 2.38 1.65 1.64 1.63 1.61 1.60 1.58 1.05 1.04 1.02
1.00
2.14
4.22
2.18
3.17
9.0 8.5 8.0 7.5 7.0 6.5 6.0 5.5 5.0 4.5 4.0 3.5 3.0 2.5 2.0 1.5 1.0 0.5 0.0
f1 (ppm)

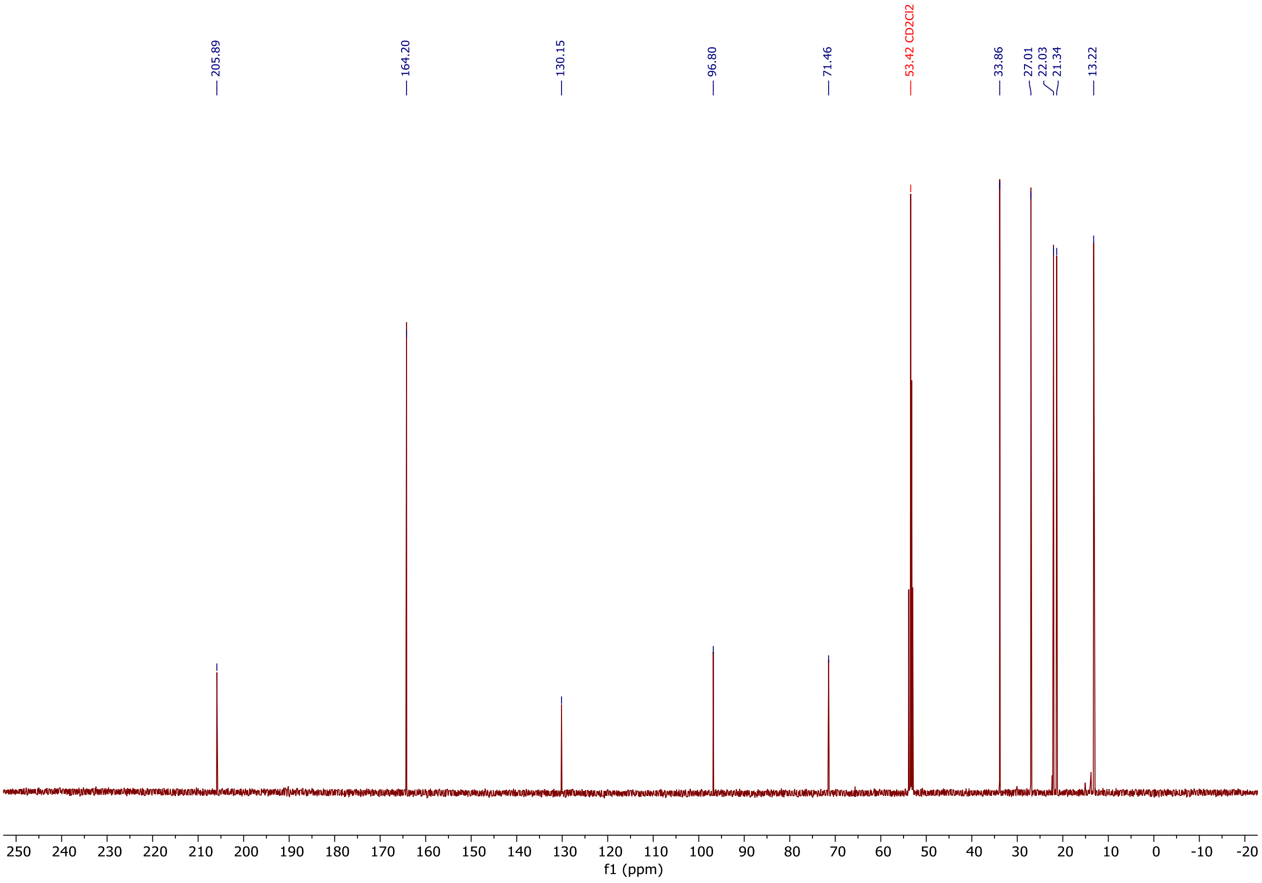

205.89
164.20
130.15
96.80
71.46
53.42 CD2Cl2
33.86
27.01
22.03
21.34
13.22
250 240 230 220 210 200 190 180 170 160 150 140 130 120 110 100 90 80 70 60 50 40 30 20 10 0 -10 -20
f1 (ppm)

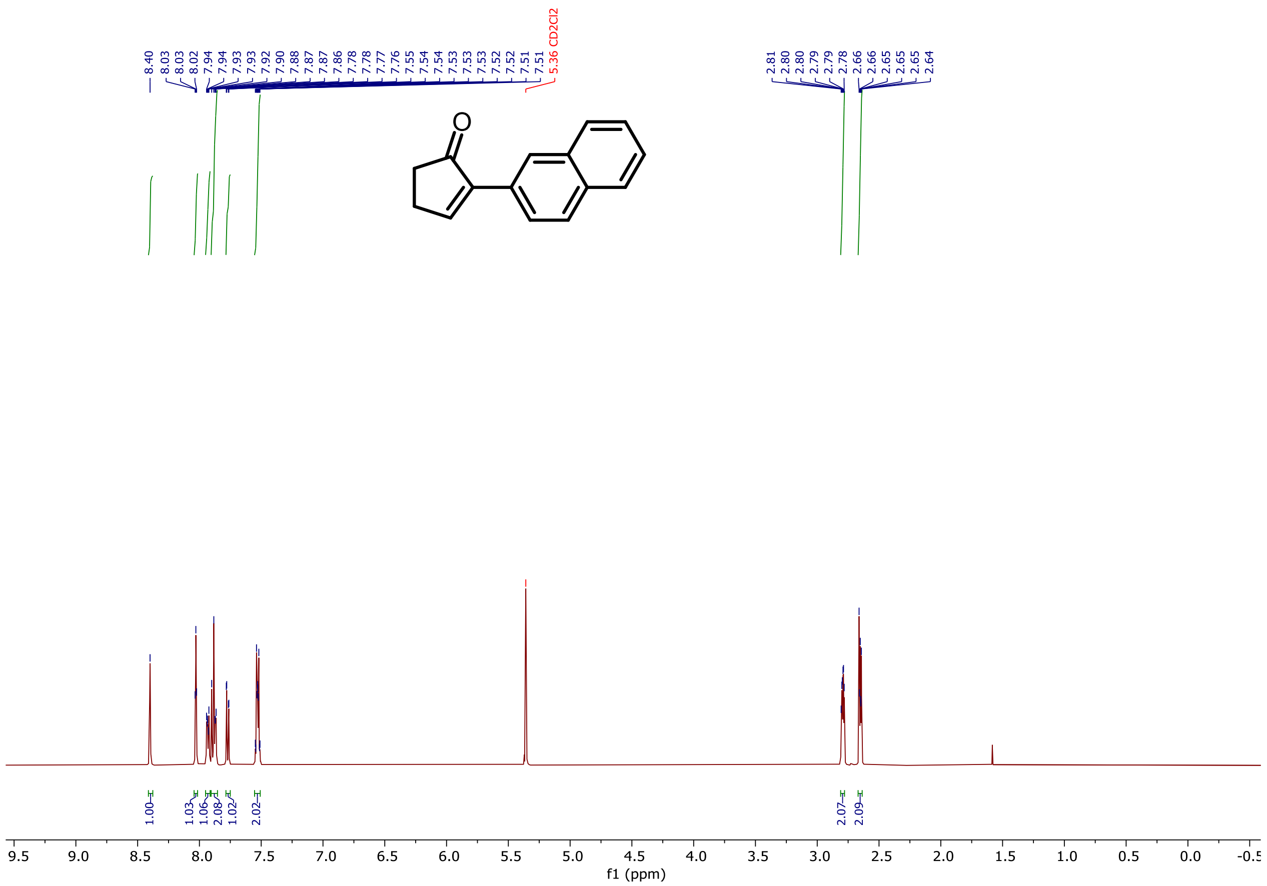
8.40
8.03
8.03
8.02
7.94
7.94
7.93
7.93
7.92
7.90
7.88
7.87
7.87
7.86
7.78
7.78
7.77
7.76
7.55
7.54
7.54
7.53
7.53
7.53
7.52
7.52
7.51
7.51
5.36 CD2Cl2
2.81
2.80
2.80
2.79
2.79
2.78
2.66
2.66
2.65
2.65
2.65
2.64
O
1.00
1.03
1.06
2.08
1.02
2.02
2.07
2.09
9.5 9.0 8.5 8.0 7.5 7.0 6.5 6.0 5.5 5.0 4.5 4.0 3.5 3.0 2.5 2.0 1.5 1.0 0.5 0.0 -0.5
f1 (ppm)

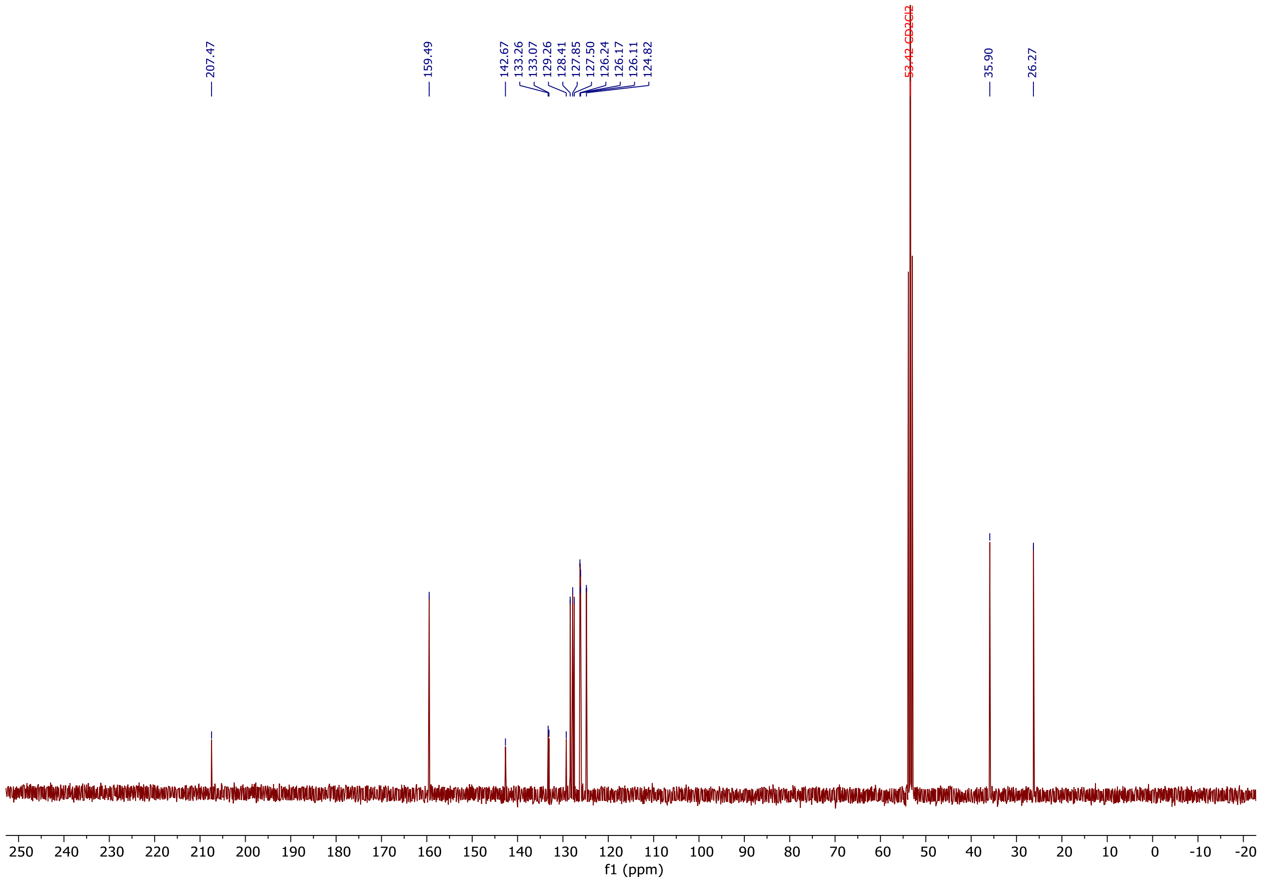
207.47
159.49
142.67
133.26
133.07
129.26
128.41
127.85
127.50
126.24
126.17
126.11
124.82
53.42 CD2Cl2
35.90
26.27
250 240 230 220 210 200 190 180 170 160 150 140 130 120 110 100 90 80 70 60 50 40 30 20 10 0 -10 -20
f1 (ppm)

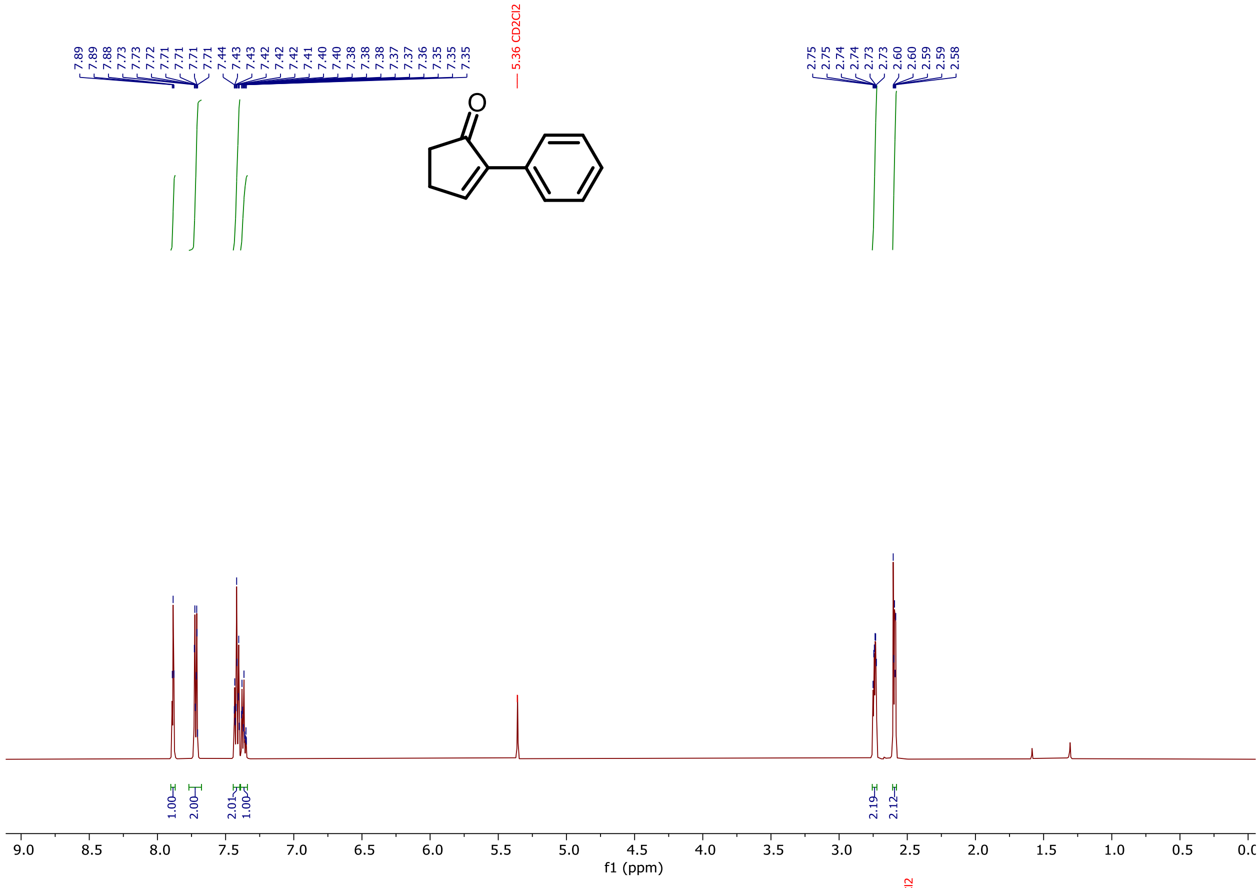

7.89
7.89
7.88
7.73
7.73
7.72
7.71
7.71
7.71
7.71
7.44
7.43
7.43
7.42
7.42
7.42
7.41
7.40
7.40
7.38
7.38
7.38
7.37
7.37
7.36
7.35
7.35
7.35
5.36 CD2Cl2
2.75
2.75
2.74
2.74
2.73
2.73
2.60
2.60
2.59
2.59
2.58
O
1.00
2.00
2.01
1.00
2.19
2.12
f1 (ppm)

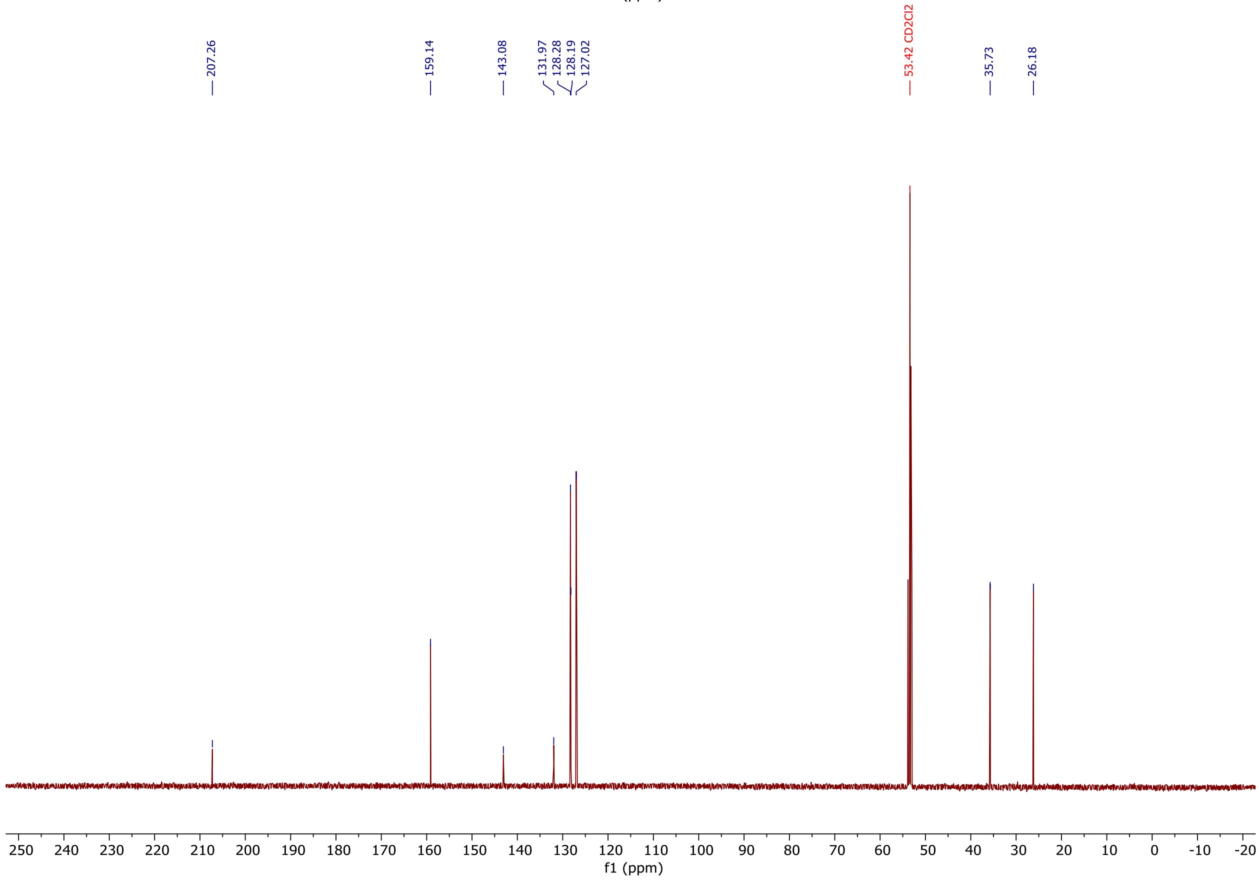

207.26
159.14
143.08
131.97
128.28
128.19
127.02
53.42 CD2Cl2
35.73
26.18
f1 (ppm)

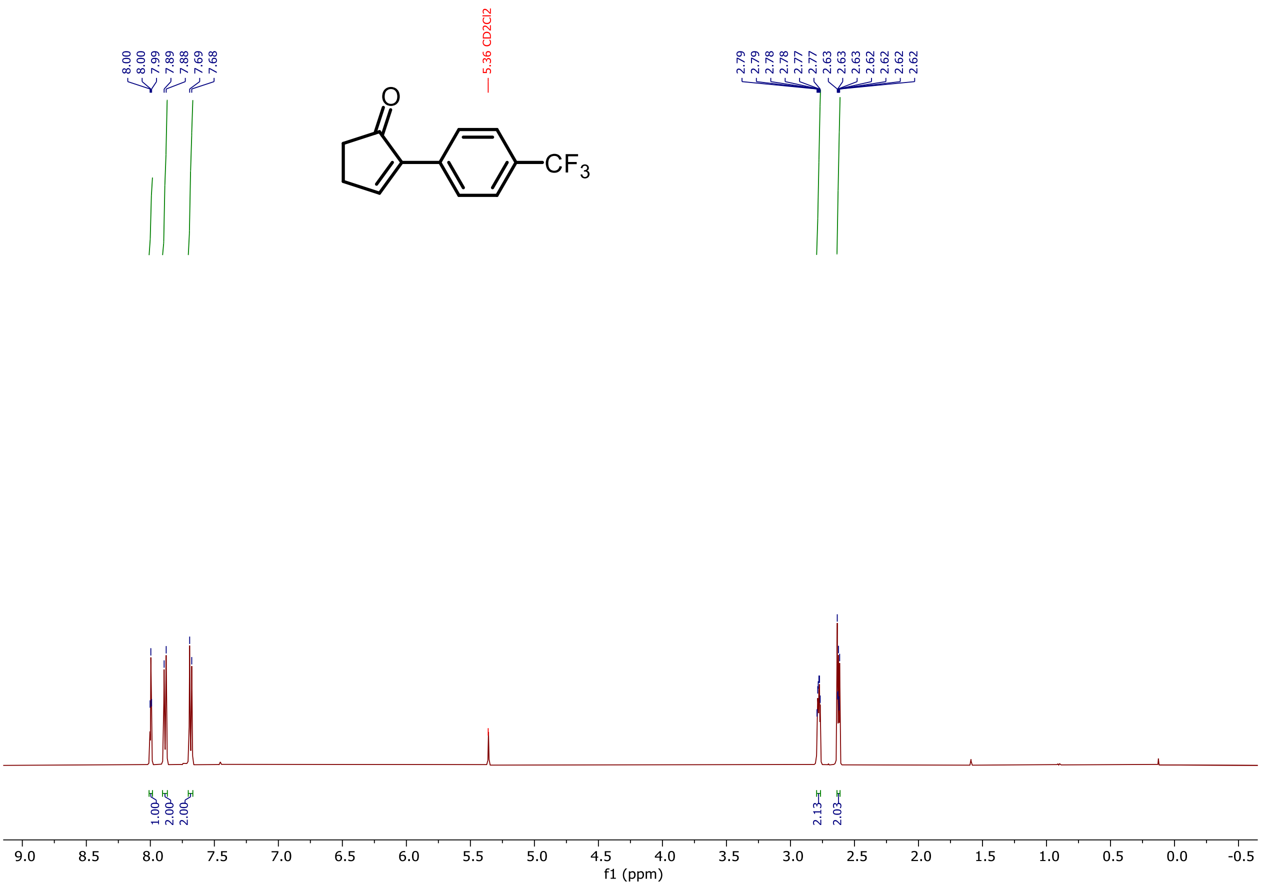
8.00
8.00
7.99
7.89
7.88
7.69
7.68
5.36 CD2Cl2
2.79
2.79
2.78
2.78
2.77
2.77
2.63
2.63
2.63
2.62
2.62
2.62
2.62
O
CF3
1.00
2.00
2.00
2.13
2.03
9.0 8.5 8.0 7.5 7.0 6.5 6.0 5.5 5.0 4.5 4.0 3.5 3.0 2.5 2.0 1.5 1.0 0.5 0.0 -0.5
f1 (ppm)

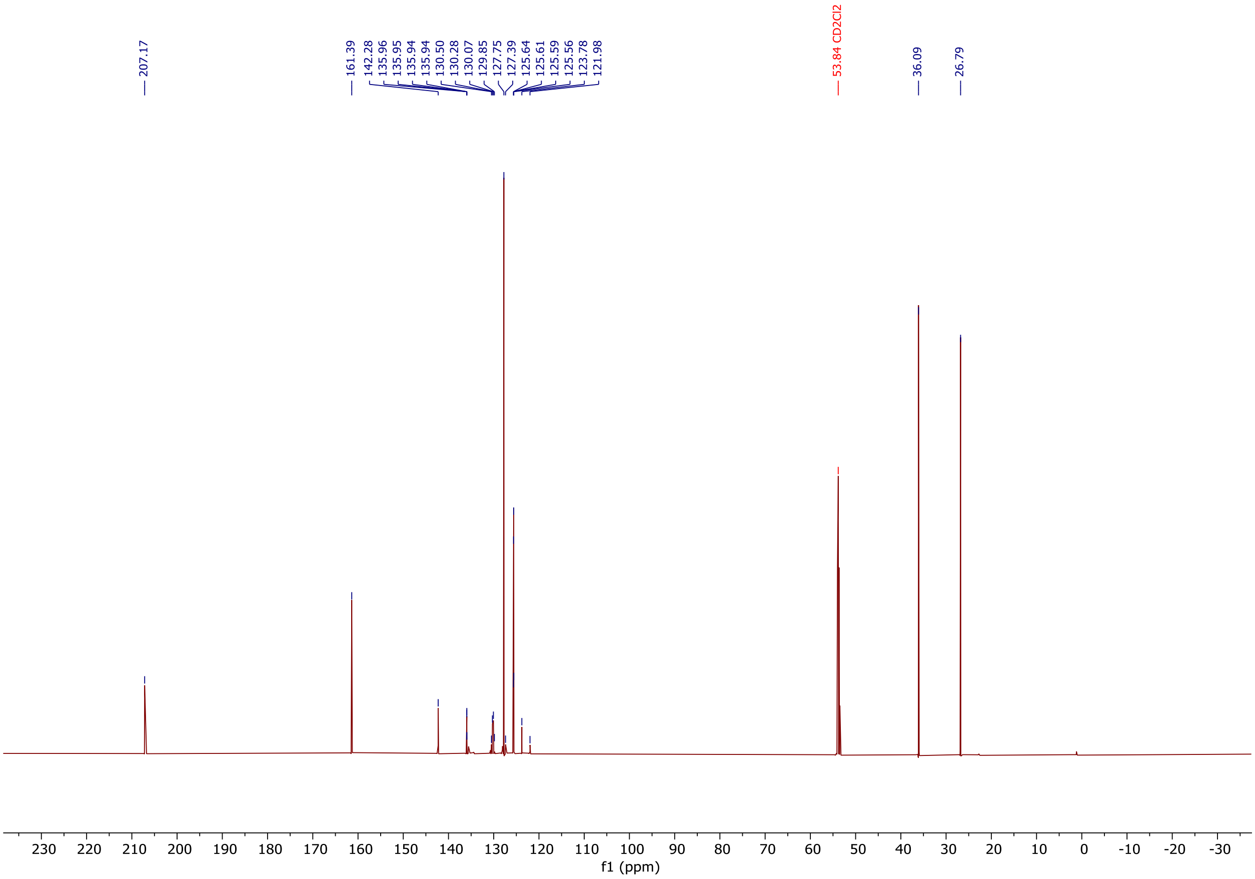
207.17
161.39
142.28
135.96
135.95
135.94
135.94
130.50
130.28
130.07
129.85
127.75
127.39
125.64
125.61
125.59
125.56
123.78
121.98
53.84 CD2Cl2
36.09
26.79
230 220 210 200 190 180 170 160 150 140 130 120 110 100 90 80 70 60 50 40 30 20 10 0 -10 -20 -30
f1 (ppm)

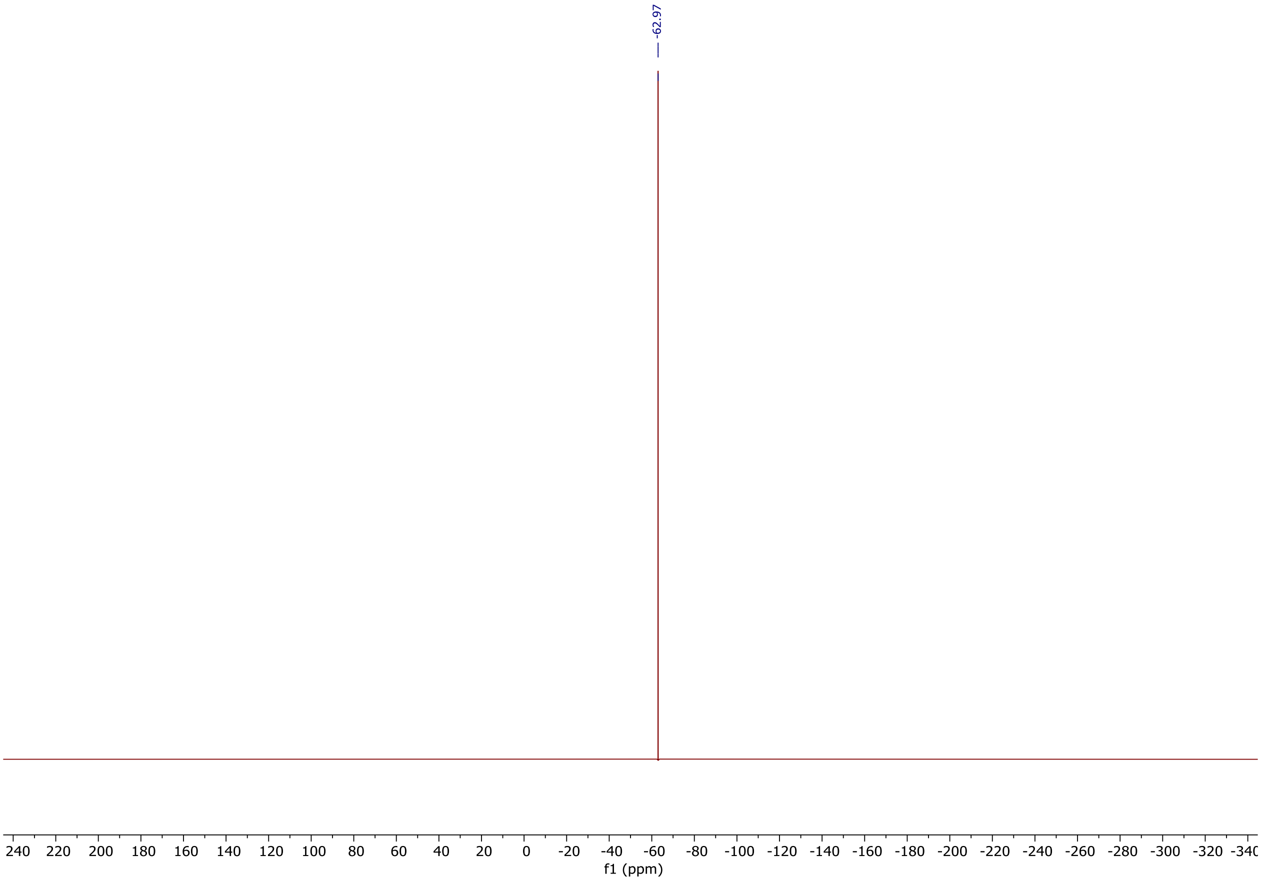
-62.97
240 220 200 180 160 140 120 100 80 60 40 20 0 -20 -40 -60 -80 -100 -120 -140 -160 -180 -200 -220 -240 -260 -280 -300 -320 -340
f1 (ppm)

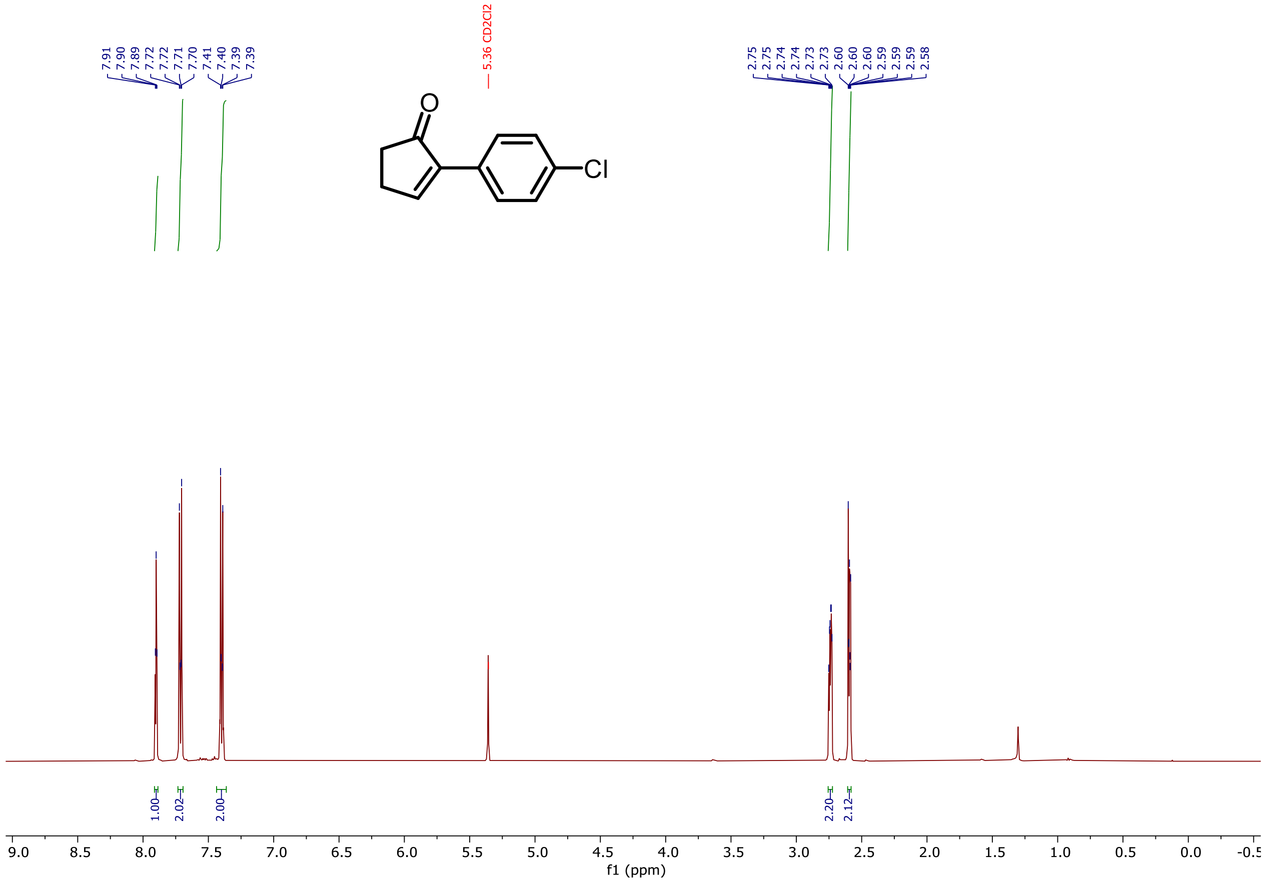

7.91
7.90
7.89
7.72
7.72
7.71
7.70
7.41
7.40
7.39
7.39
5.36 CD2Cl2
2.75
2.75
2.74
2.74
2.73
2.73
2.60
2.60
2.60
2.59
2.59
2.59
2.58
1.00
2.02
2.00
2.20
2.12
O
Cl
f1 (ppm)

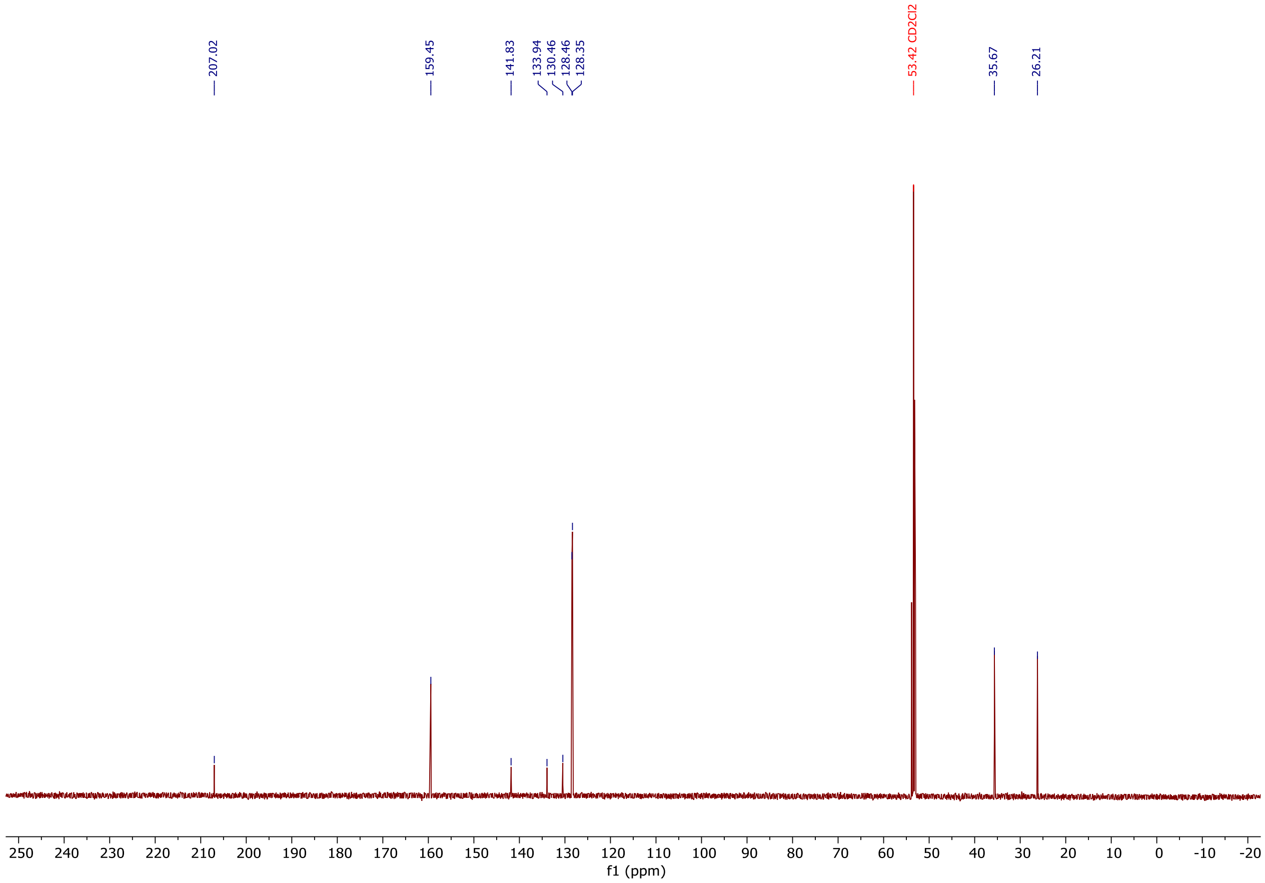

207.02
159.45
141.83
133.94
130.46
128.46
128.35
53.42 CD2Cl2
35.67
26.21
f1 (ppm)

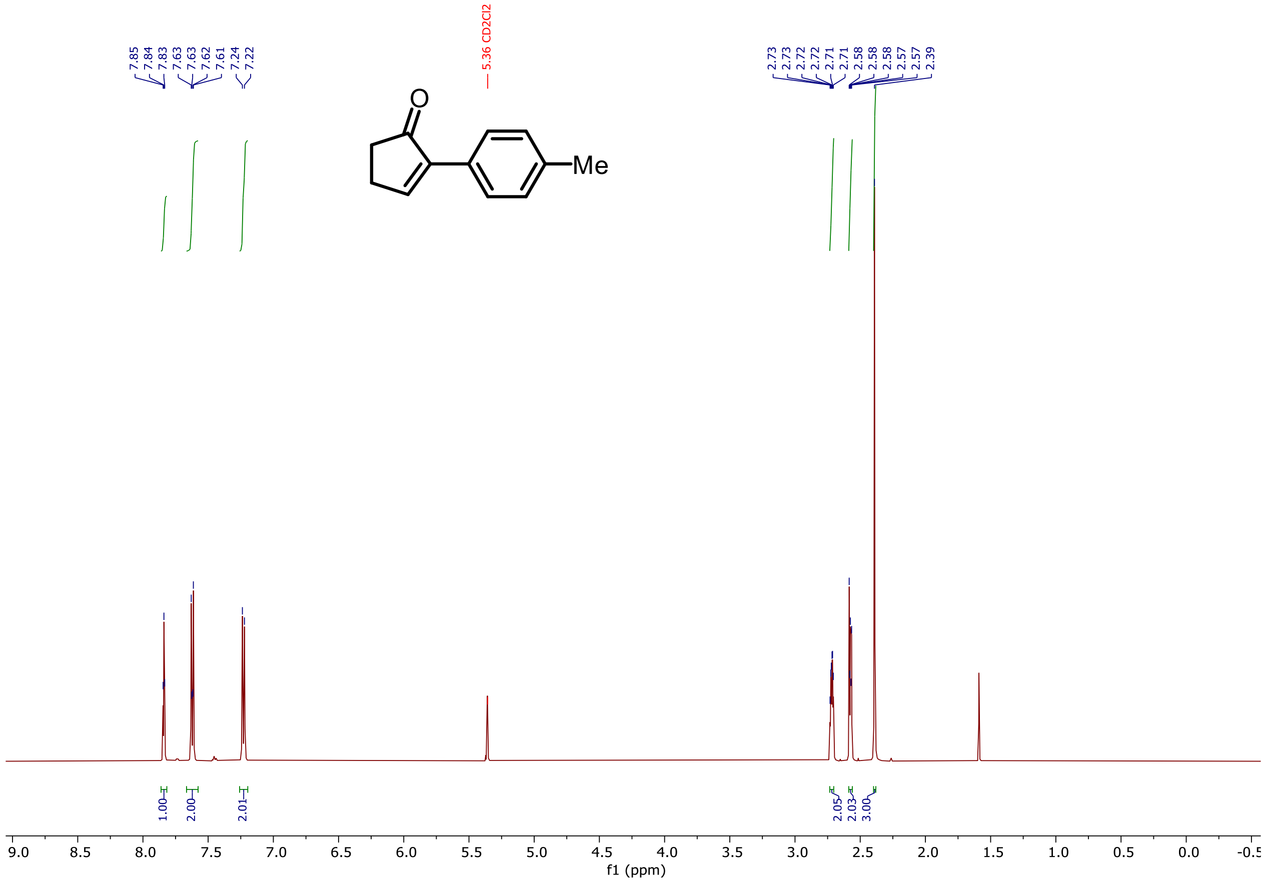

7.85
7.84
7.83
7.63
7.63
7.62
7.61
7.24
7.22
5.36 CD2Cl2
2.73
2.73
2.72
2.72
2.71
2.71
2.58
2.58
2.58
2.57
2.57
2.39
O
Me
1.00
2.00
2.01
2.05
2.03
3.00
9.0 8.5 8.0 7.5 7.0 6.5 6.0 5.5 5.0 4.5 4.0 3.5 3.0 2.5 2.0 1.5 1.0 0.5 0.0 -0.5
f1 (ppm)

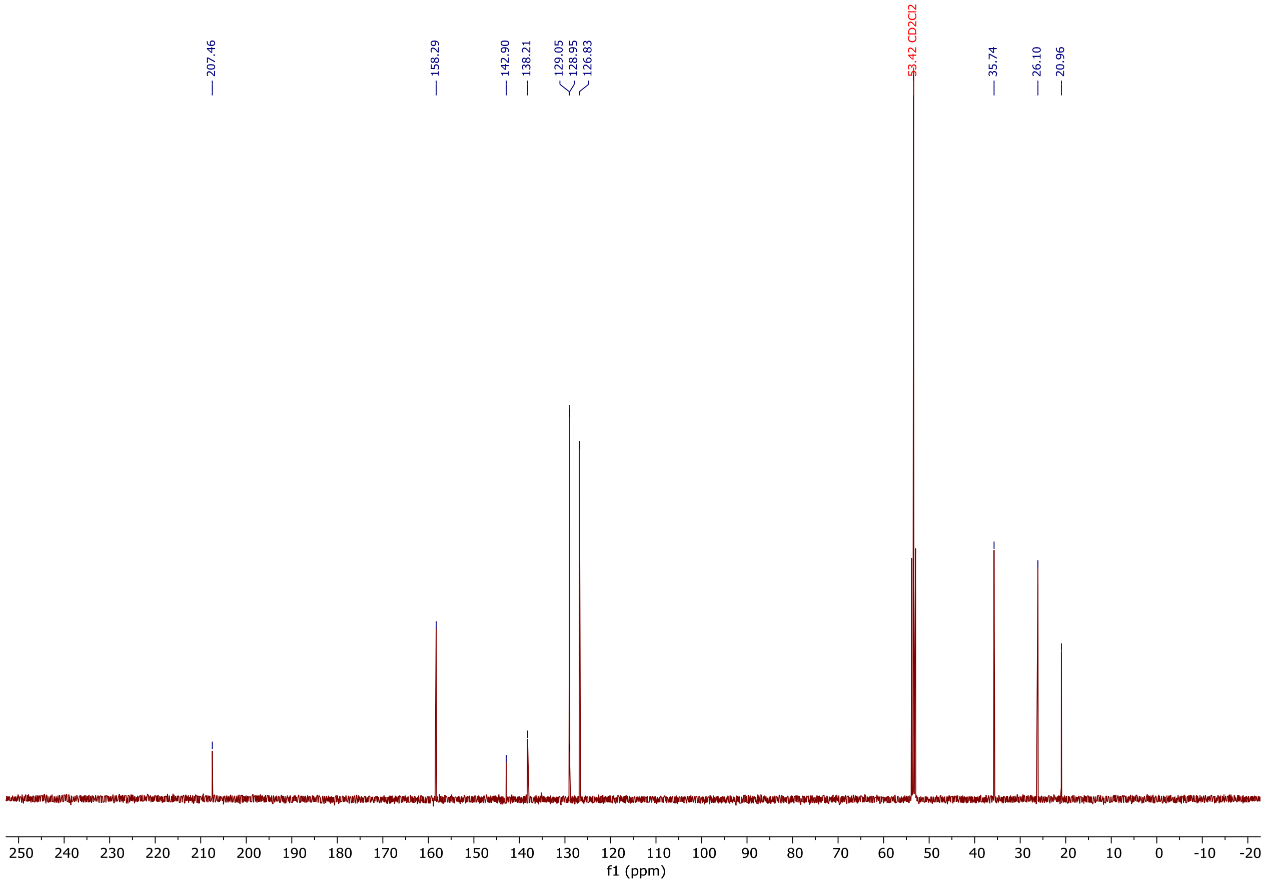

207.46
158.29
142.90
138.21
129.05
128.95
126.83
53.42 CD2Cl2
35.74
26.10
20.96
250 240 230 220 210 200 190 180 170 160 150 140 130 120 110 100 90 80 70 60 50 40 30 20 10 0 -10 -20
f1 (ppm)

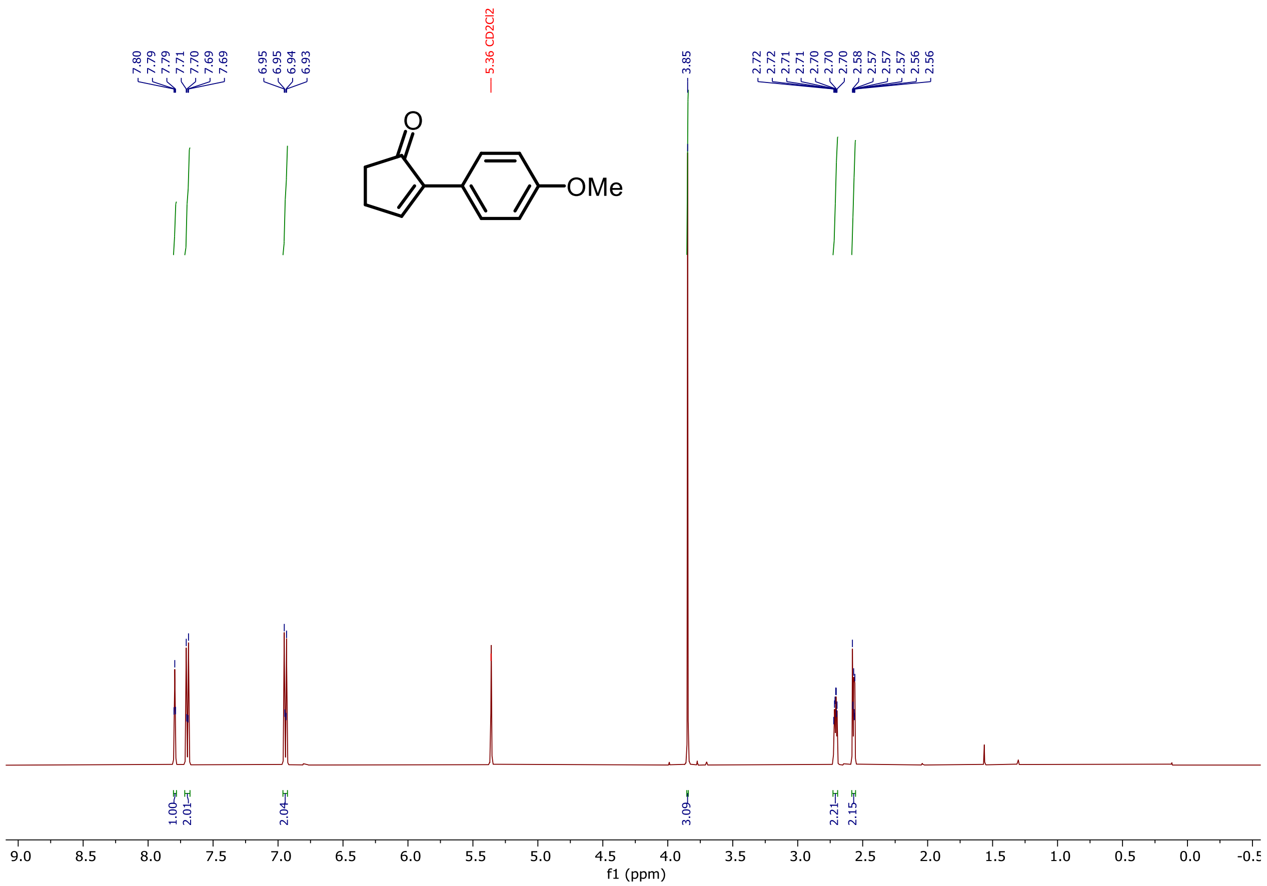

7.80 7.79 7.79 7.71 7.70 7.69 7.69
6.95 6.95 6.94 6.93
5.36 CD2Cl2
3.85
2.72 2.72 2.71 2.71 2.70 2.70 2.70 2.58 2.57 2.57 2.57 2.56 2.56
O
OMe
1.00 2.01 2.04 3.09 2.21 2.15
9.0 8.5 8.0 7.5 7.0 6.5 6.0 5.5 5.0 4.5 4.0 3.5 3.0 2.5 2.0 1.5 1.0 0.5 0.0
f1 (ppm)

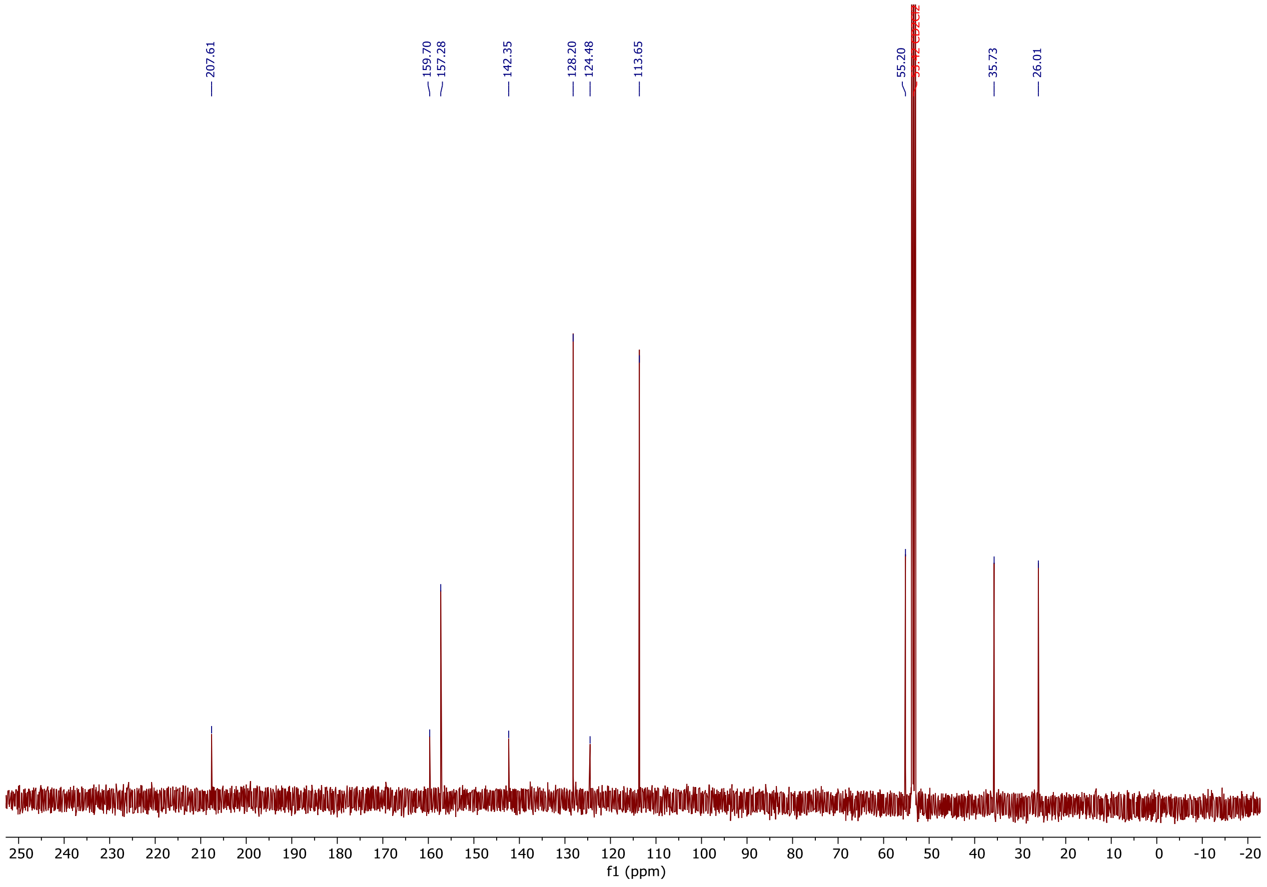

207.61
159.70 157.28
142.35
128.20 124.48
113.65
55.20
35.73
26.01
250 240 230 220 210 200 190 180 170 160 150 140 130 120 110 100 90 80 70 60 50 40 30 20 10 0 -10 -20
f1 (ppm)

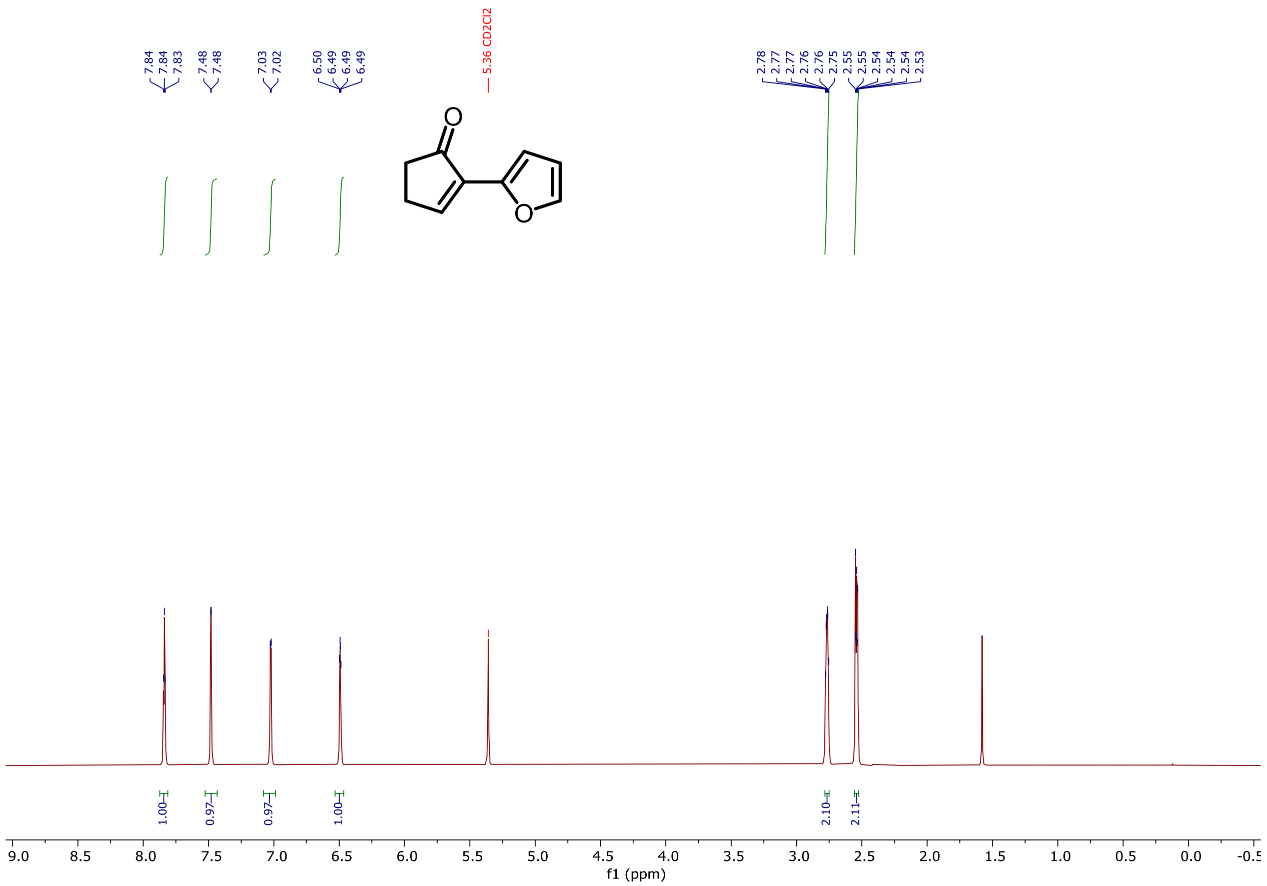
7.84
7.84
7.83
7.48
7.48
7.03
7.02
6.50
6.49
6.49
6.49
5.36 CD2Cl2
2.78
2.77
2.77
2.76
2.76
2.75
2.55
2.55
2.54
2.54
2.54
2.53
O
O
1.00
0.97
0.97
1.00
2.10
2.11
f1 (ppm)

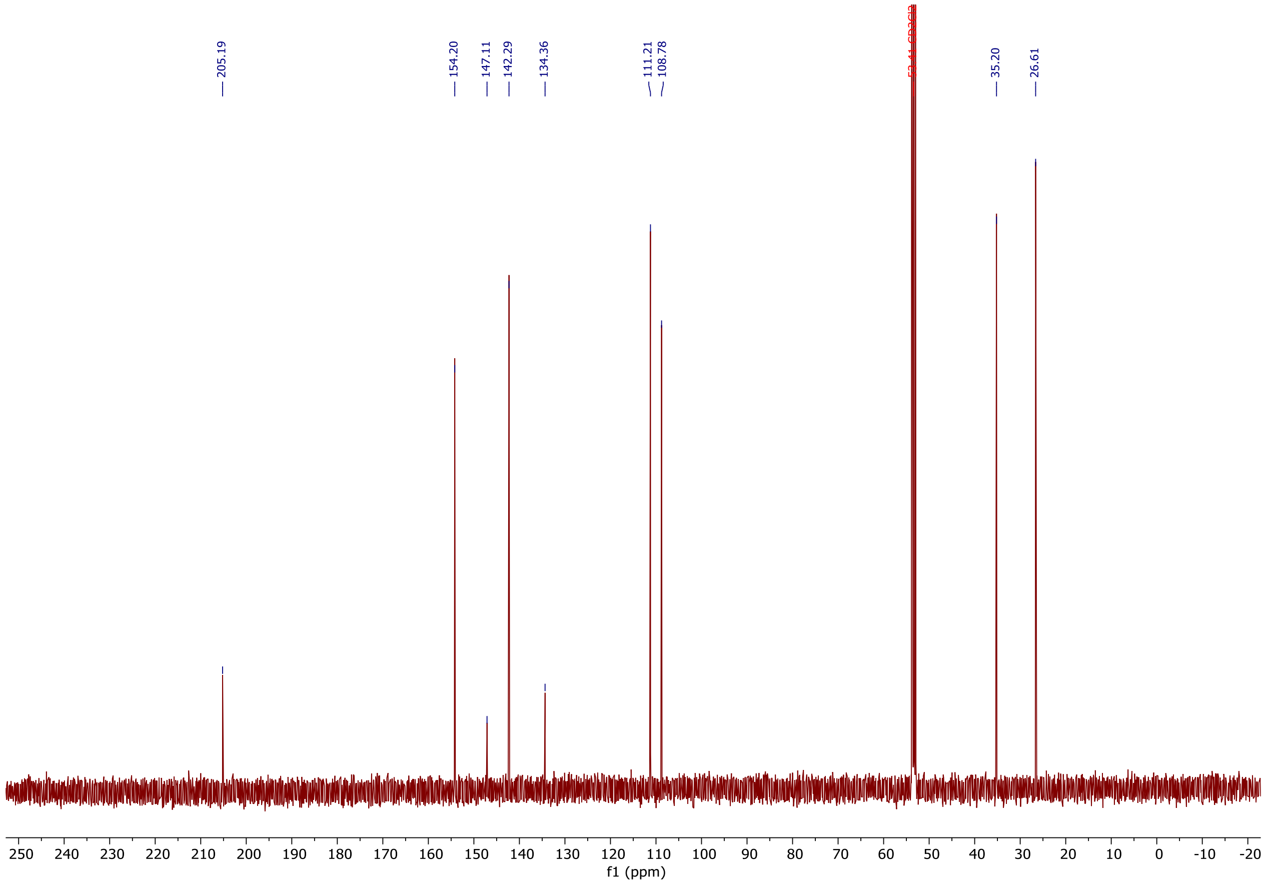
205.19
154.20
147.11
142.29
134.36
111.21
108.78
35.20
26.61
f1 (ppm)

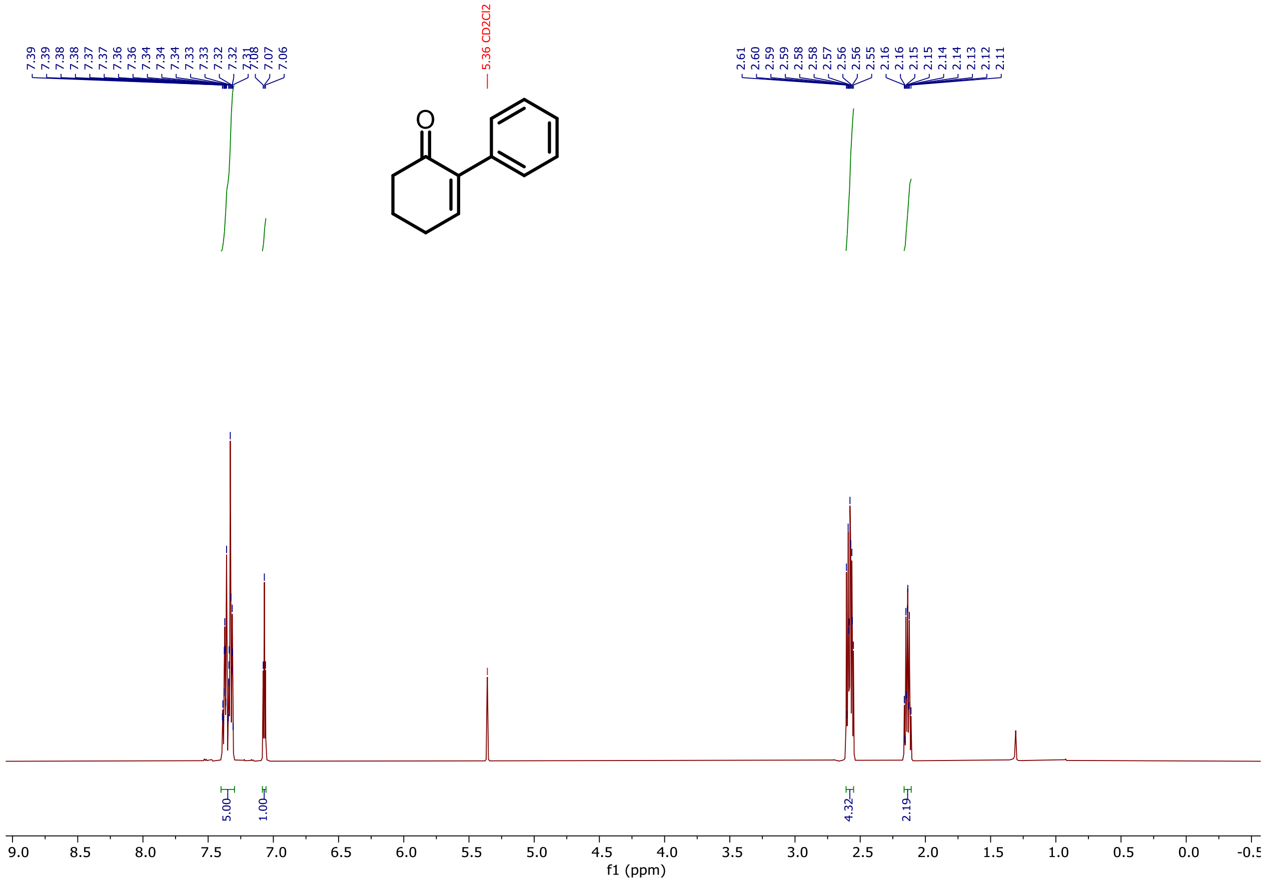
7.39
7.39
7.38
7.38
7.37
7.37
7.36
7.36
7.34
7.34
7.34
7.33
7.33
7.32
7.32
7.31
7.08
7.07
7.06
5.36 CD2Cl2
2.61
2.60
2.59
2.59
2.58
2.58
2.57
2.56
2.56
2.55
2.16
2.16
2.15
2.15
2.14
2.14
2.13
2.12
2.11
O
5.00
1.00
4.32
2.19
9.0 8.5 8.0 7.5 7.0 6.5 6.0 5.5 5.0 4.5 4.0 3.5 3.0 2.5 2.0 1.5 1.0 0.5 0.0 -0.5
f1 (ppm)

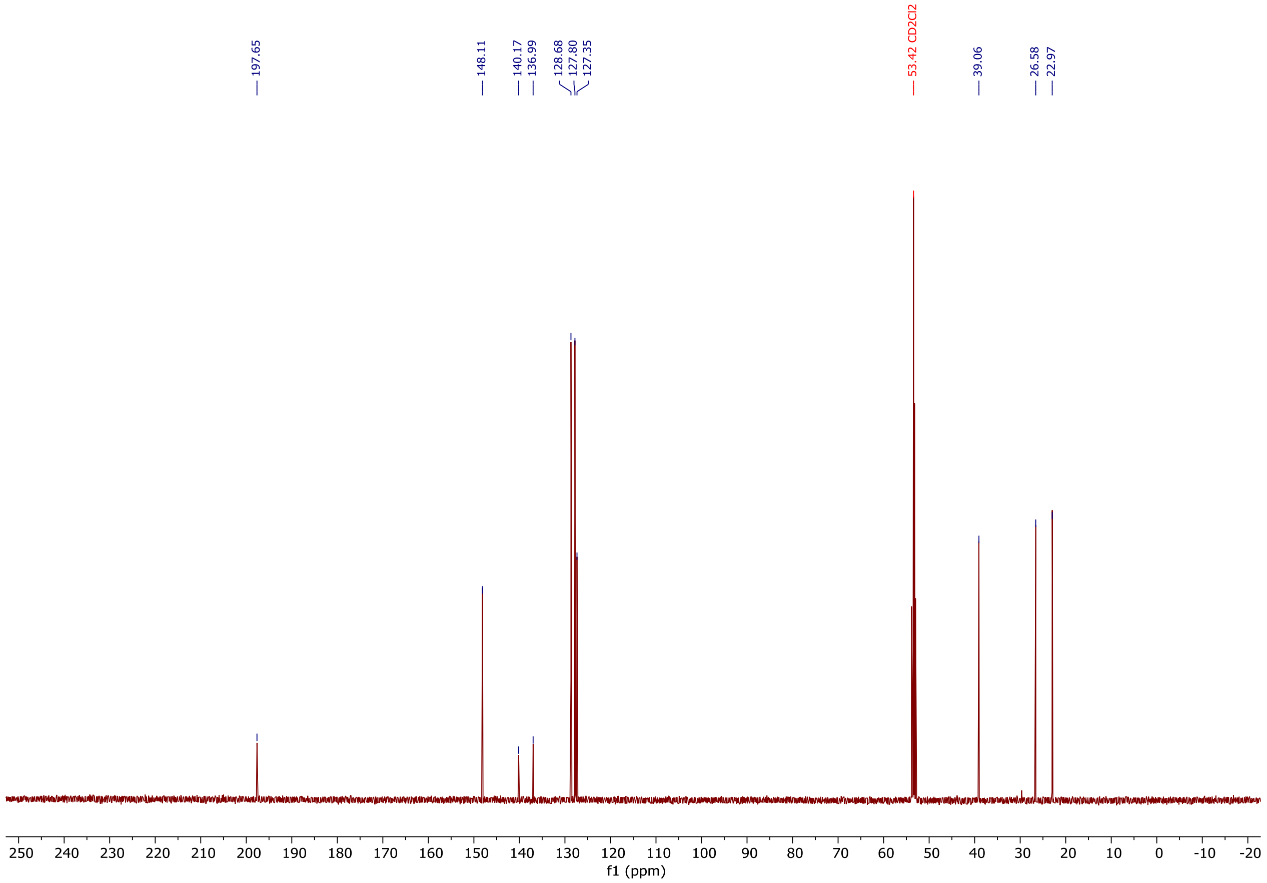
197.65
148.11
140.17
136.99
128.68
127.80
127.35
53.42 CD2Cl2
39.06
26.58
22.97
250 240 230 220 210 200 190 180 170 160 150 140 130 120 110 100 90 80 70 60 50 40 30 20 10 0 -10 -20
f1 (ppm)

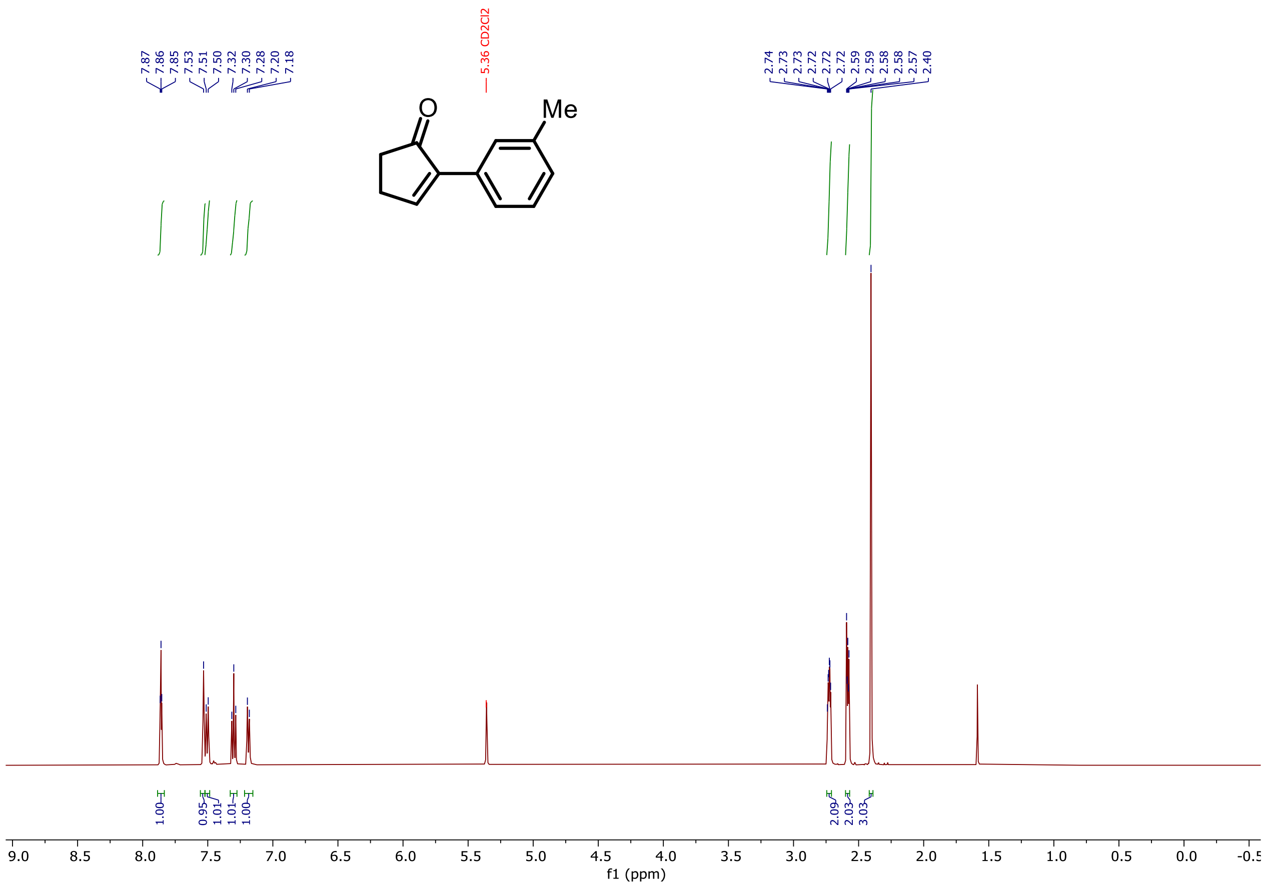

7.87
7.86
7.85
7.53
7.51
7.50
7.32
7.30
7.28
7.20
7.18
5.36 CD2Cl2
2.74
2.73
2.73
2.72
2.72
2.72
2.72
2.59
2.59
2.58
2.58
2.57
2.40
O
Me
1.00
0.95
1.01
1.01
1.00
2.09
2.03
3.03
9.0 8.5 8.0 7.5 7.0 6.5 6.0 5.5 5.0 4.5 4.0 3.5 3.0 2.5 2.0 1.5 1.0 0.5 0.0 -0.5
f1 (ppm)

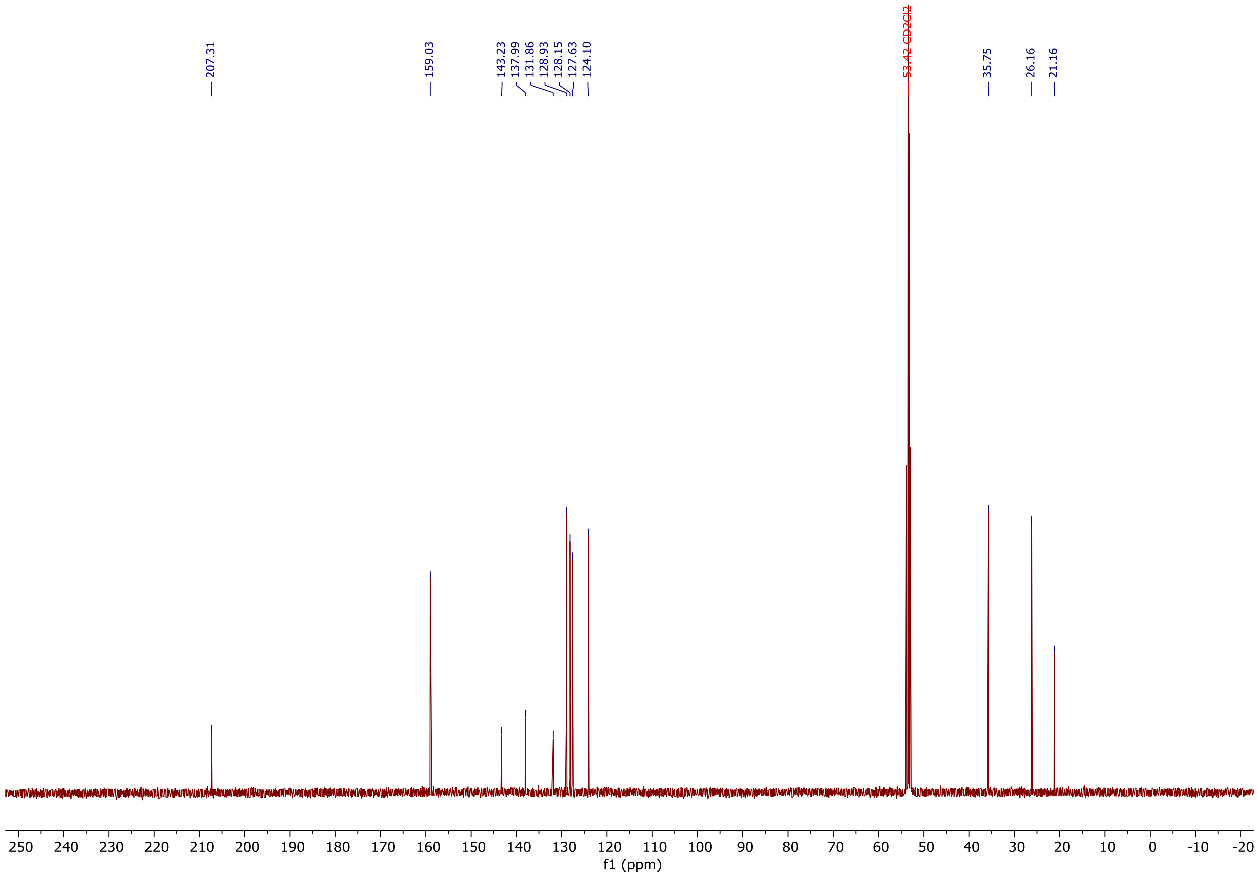

207.31
159.03
143.23
137.99
131.86
128.93
128.15
127.63
124.10
53.42 CD2Cl2
35.75
26.16
21.16
250 240 230 220 210 200 190 180 170 160 150 140 130 120 110 100 90 80 70 60 50 40 30 20 10 0 -10 -20
f1 (ppm)

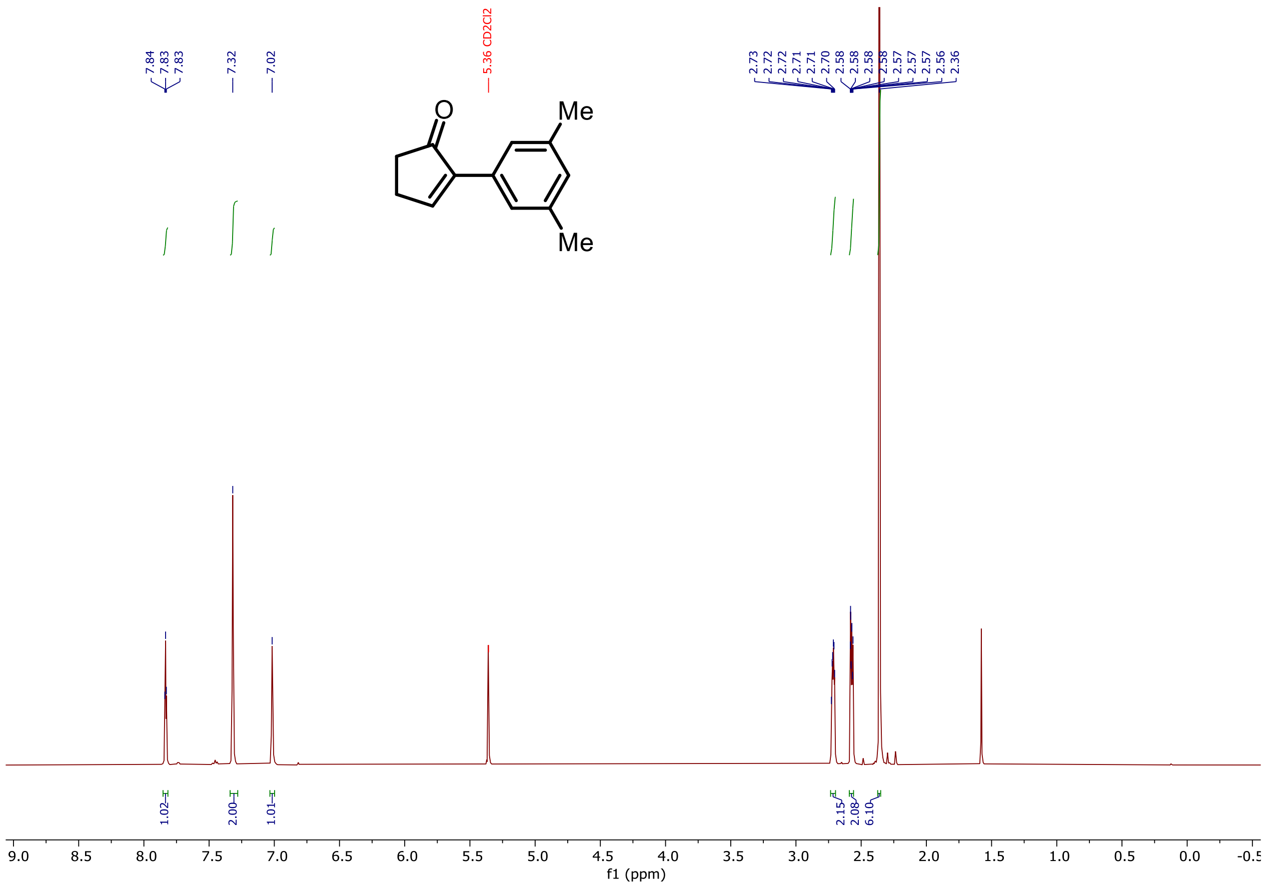
7.84
7.83
7.83
7.32
7.02
5.36 CD2Cl2
2.73
2.72
2.72
2.71
2.71
2.70
2.58
2.58
2.58
2.58
2.57
2.57
2.57
2.56
2.36
O
Me
Me
1.02
2.00
1.01
2.15
2.08
6.10
f1 (ppm)

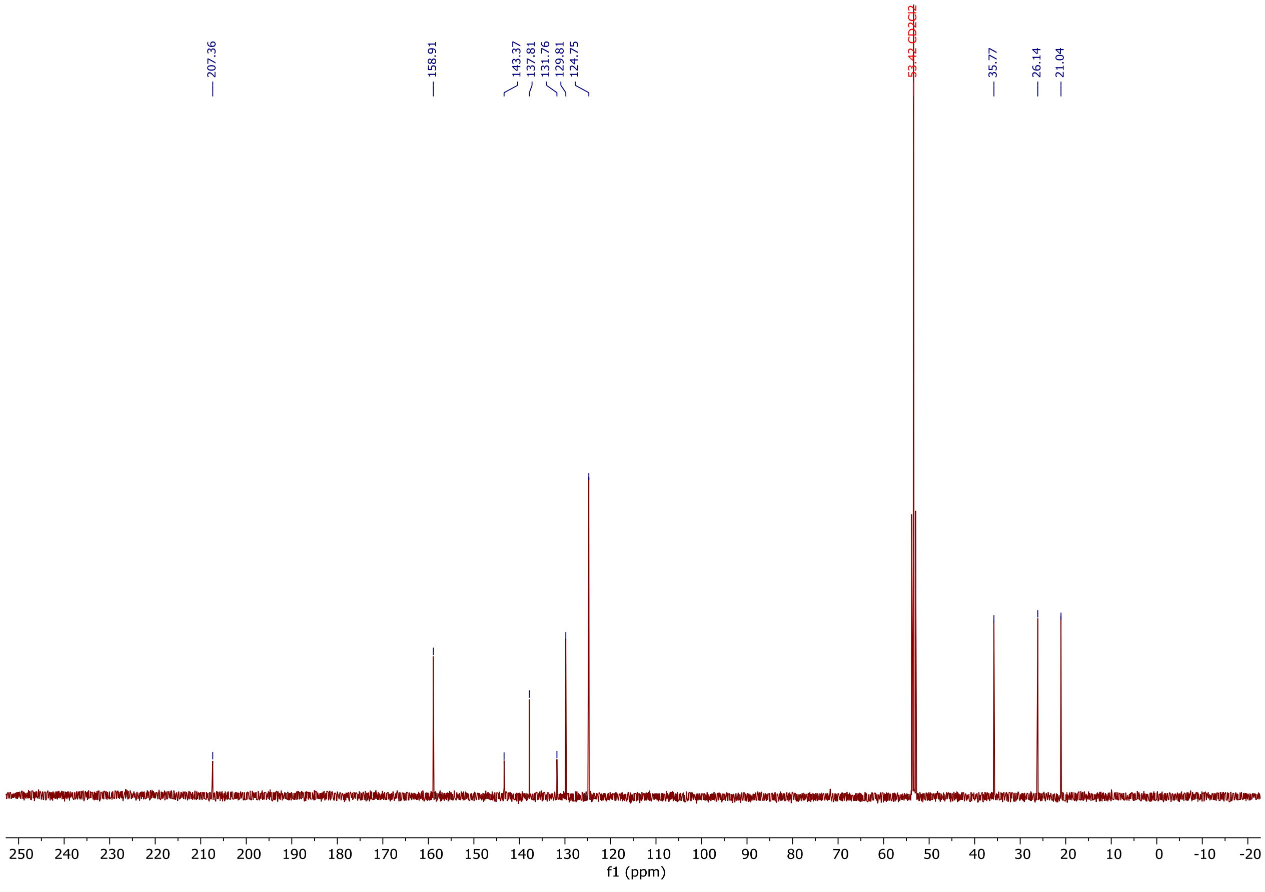
207.36
158.91
143.37
137.81
131.76
129.81
124.75
53.42 CD2Cl2
35.77
26.14
21.04
f1 (ppm)

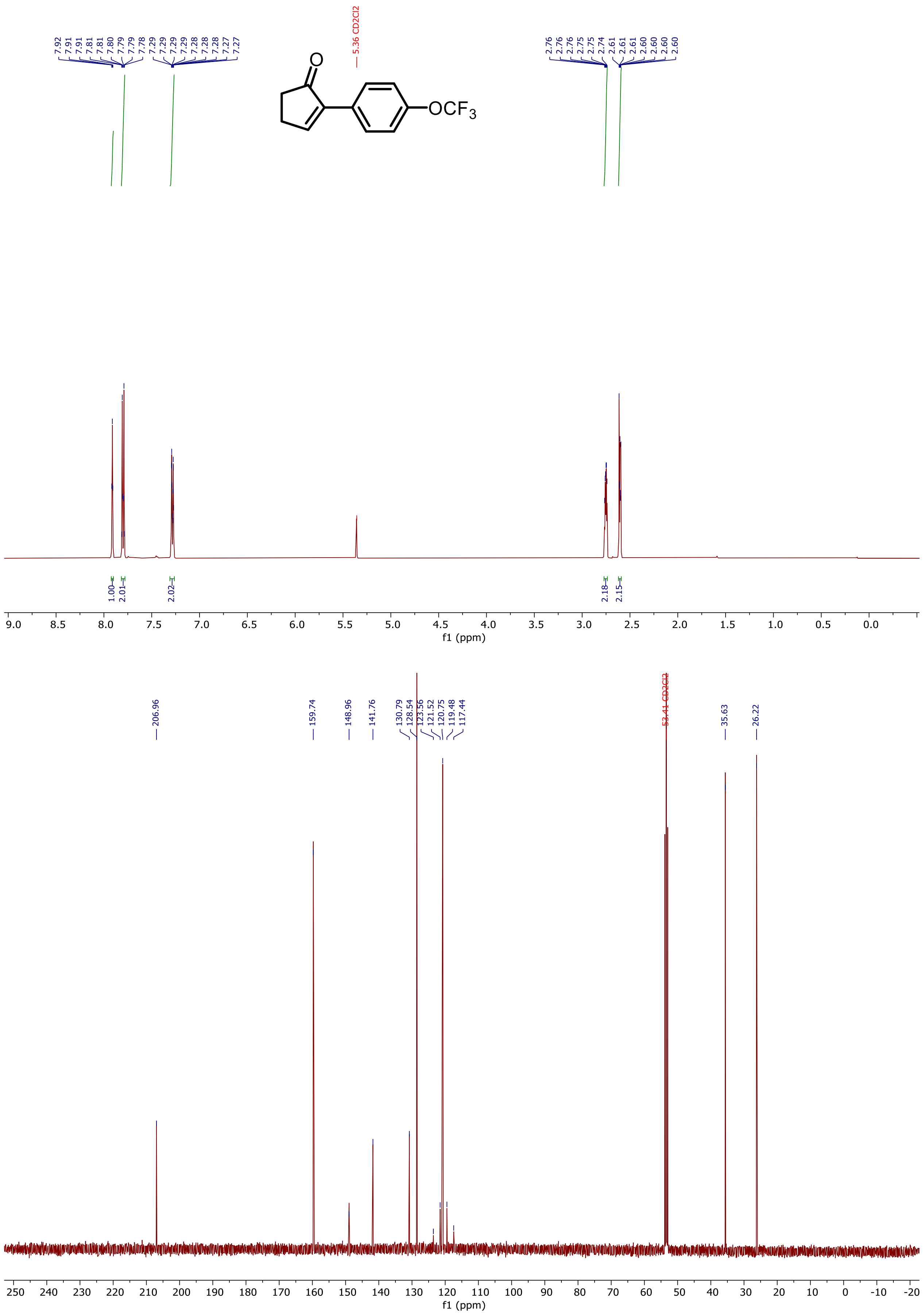

OCF3
5.36 CD2Cl2
f1 (ppm)
53.41 CD2Cl2
f1 (ppm)

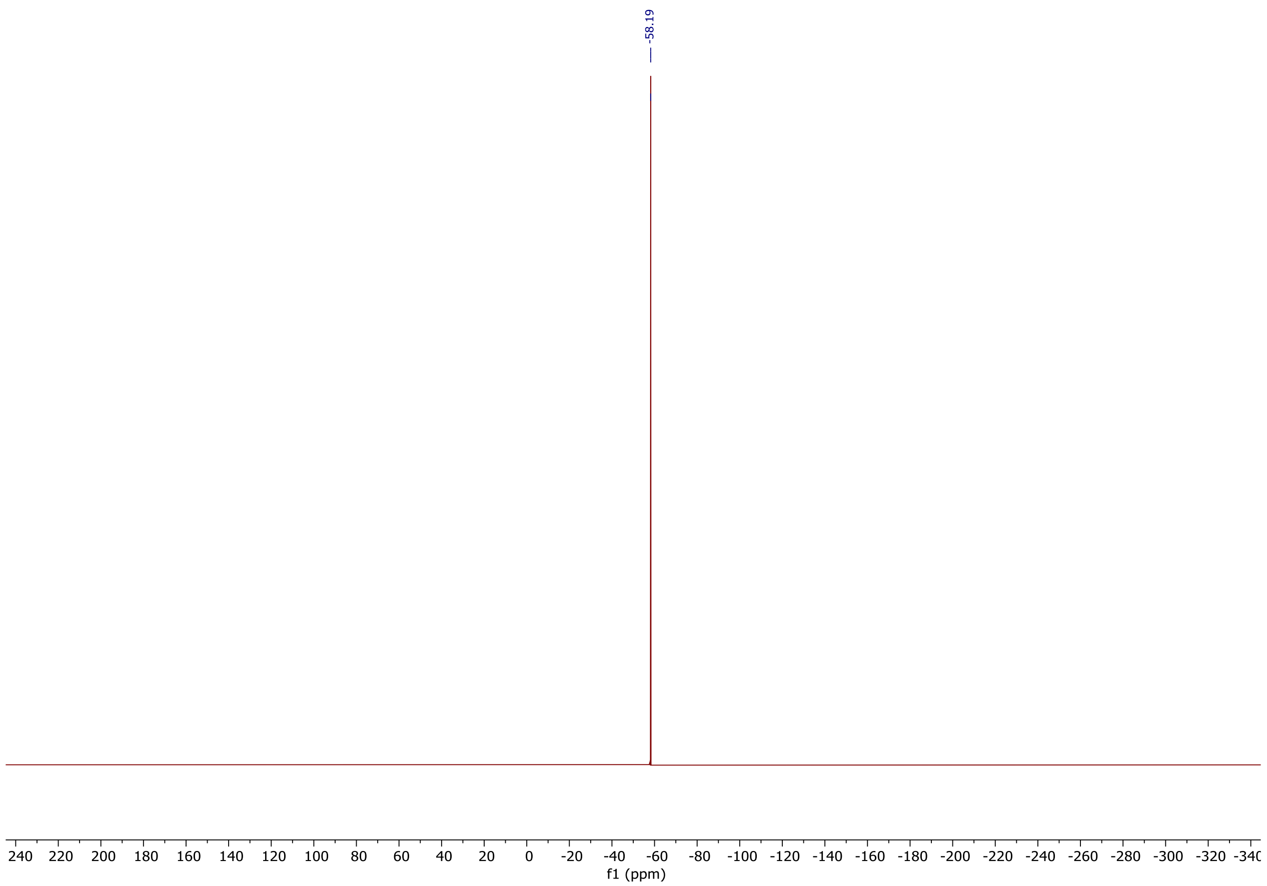
-58.19
240 220 200 180 160 140 120 100 80 60 40 20 0 -20 -40 -60 -80 -100 -120 -140 -160 -180 -200 -220 -240 -260 -280 -300 -320 -340
f1 (ppm)

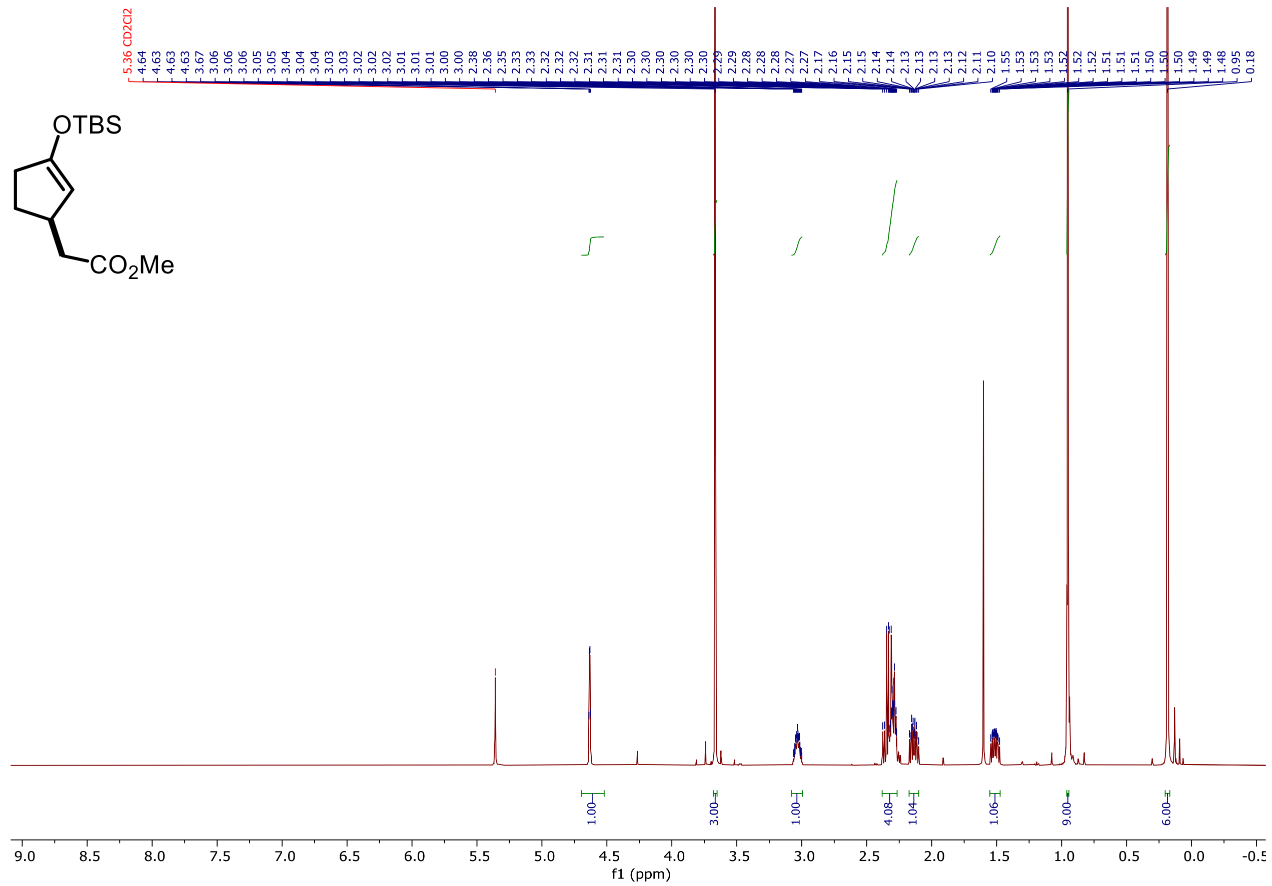
OTBS
CO2Me
f1 (ppm)

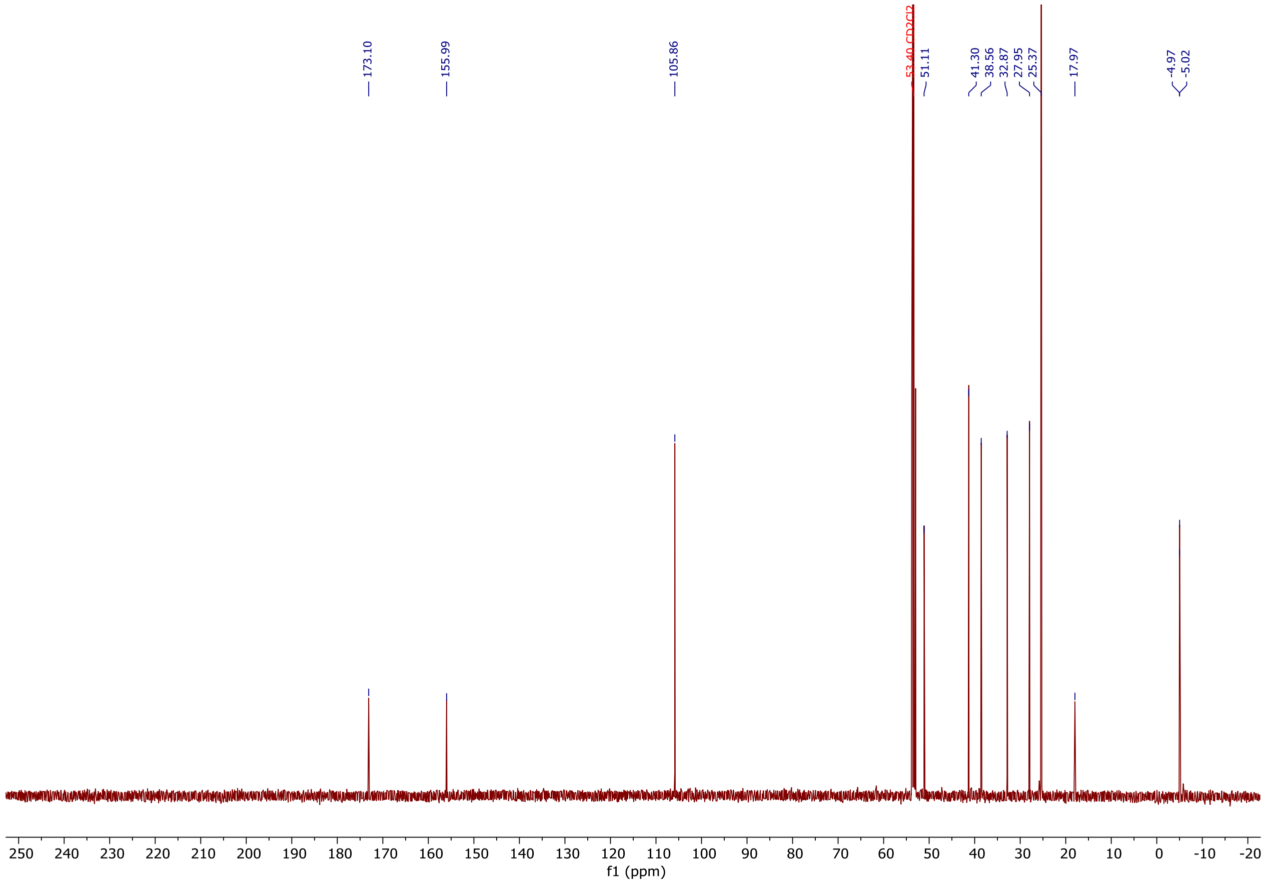
f1 (ppm)

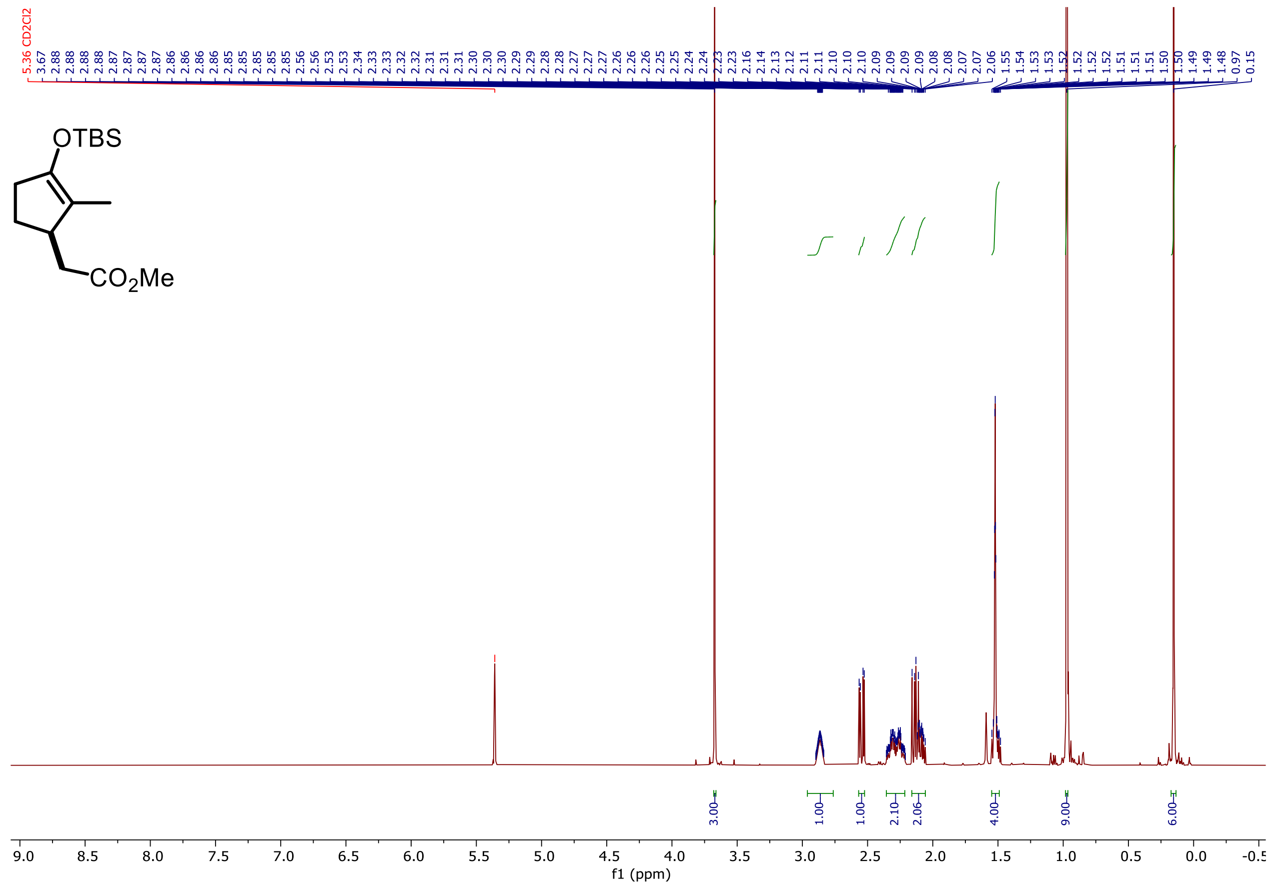
OTBS
CO2Me
f1 (ppm)

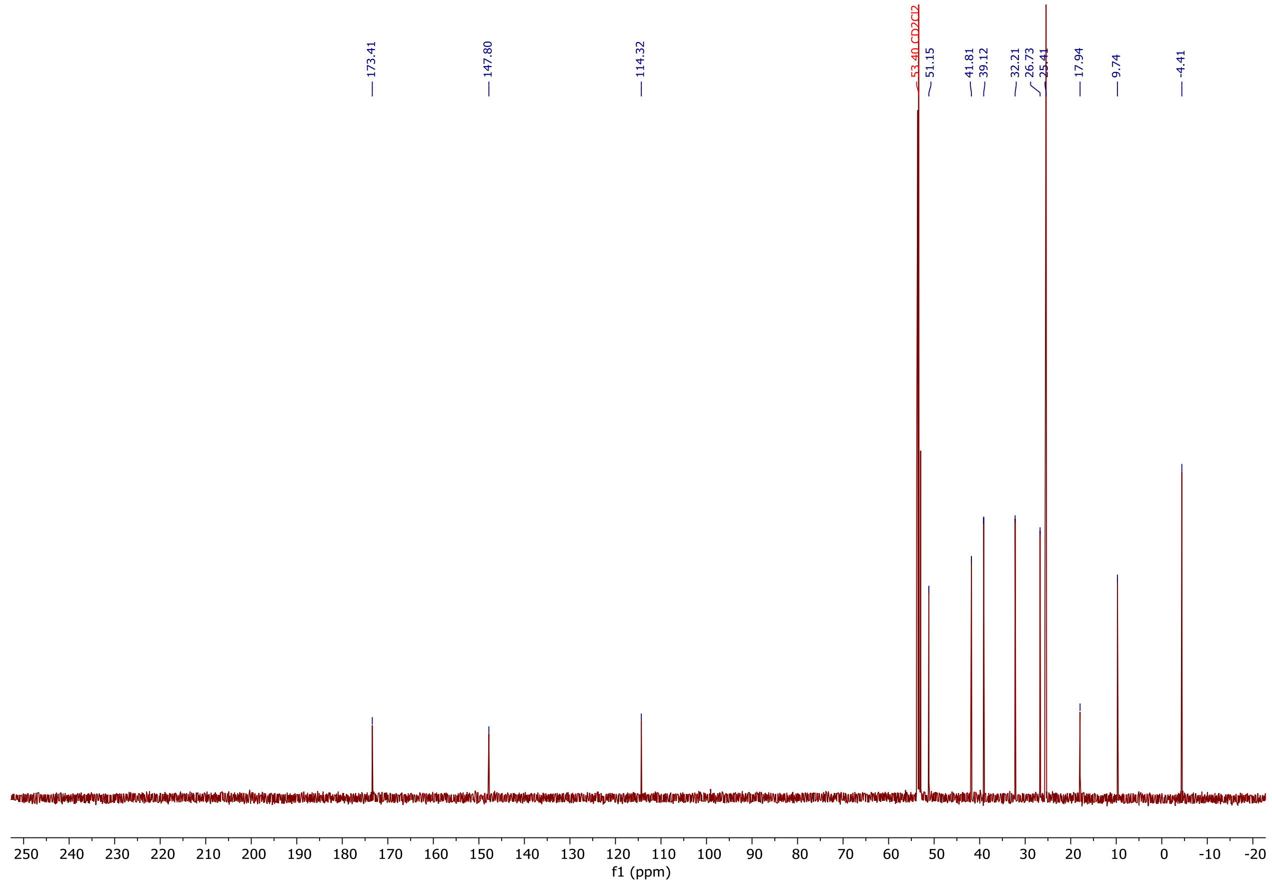
f1 (ppm)

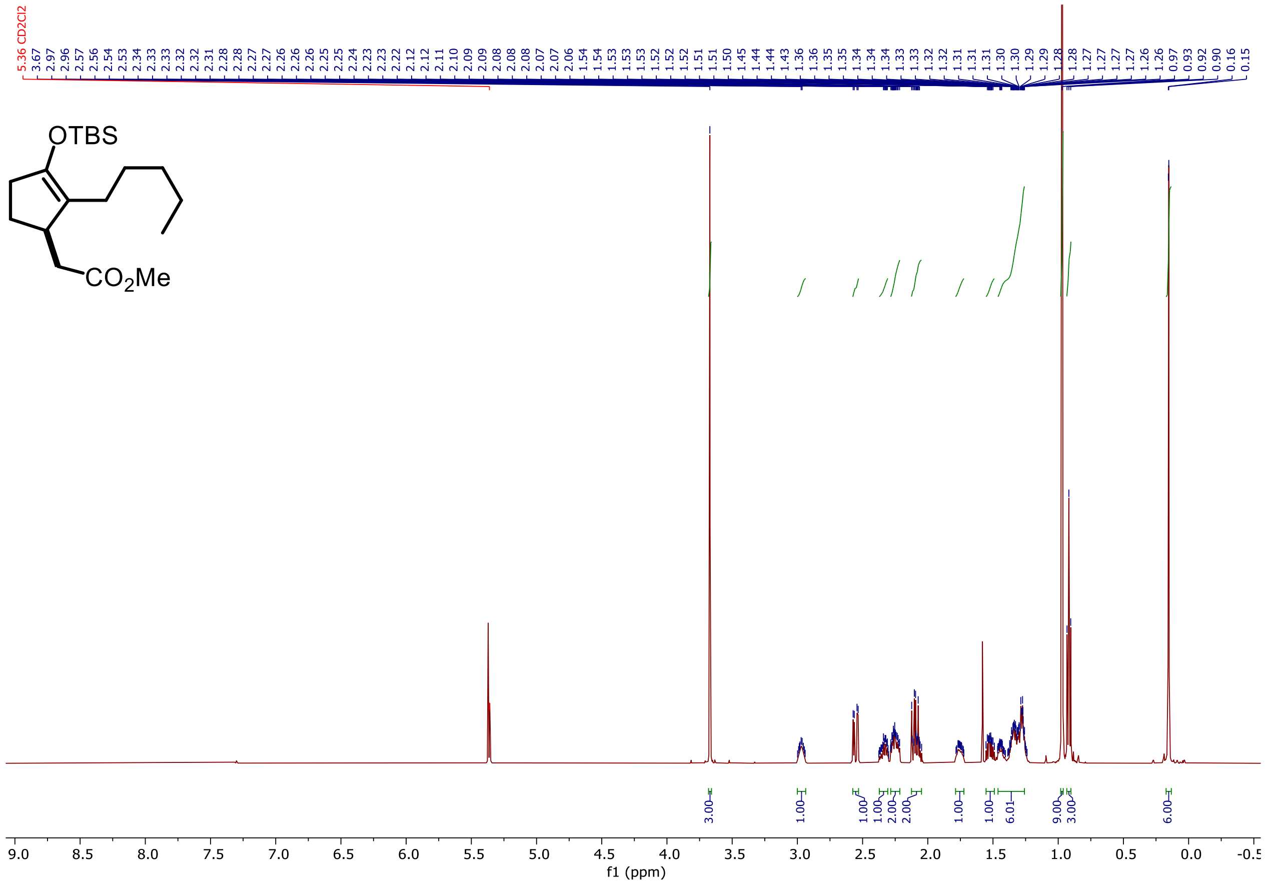

5.36 CD2Cl2
3.67
2.97
2.96
2.57
2.56
2.54
2.53
2.34
2.33
2.33
2.32
2.31
2.28
2.28
2.27
2.27
2.26
2.26
2.25
2.25
2.24
2.23
2.23
2.22
2.12
2.12
2.11
2.10
2.09
2.09
2.08
2.08
2.08
2.07
2.07
2.06
1.54
1.54
1.53
1.53
1.53
1.52
1.52
1.52
1.51
1.51
1.50
1.45
1.44
1.44
1.43
1.36
1.36
1.35
1.35
1.34
1.34
1.34
1.33
1.33
1.33
1.32
1.32
1.31
1.31
1.30
1.30
1.29
1.29
1.28
1.28
1.27
1.27
1.27
1.27
1.26
0.97
0.93
0.92
0.90
0.16
0.15
OTBS
CO2Me
3.00
1.00
1.00
1.00
2.00
2.00
1.00
1.00
6.01
9.00
3.00
6.00
9.0 8.5 8.0 7.5 7.0 6.5 6.0 5.5 5.0 4.5 4.0 3.5 3.0 2.5 2.0 1.5 1.0 0.5 0.0 -0.5
f1 (ppm)

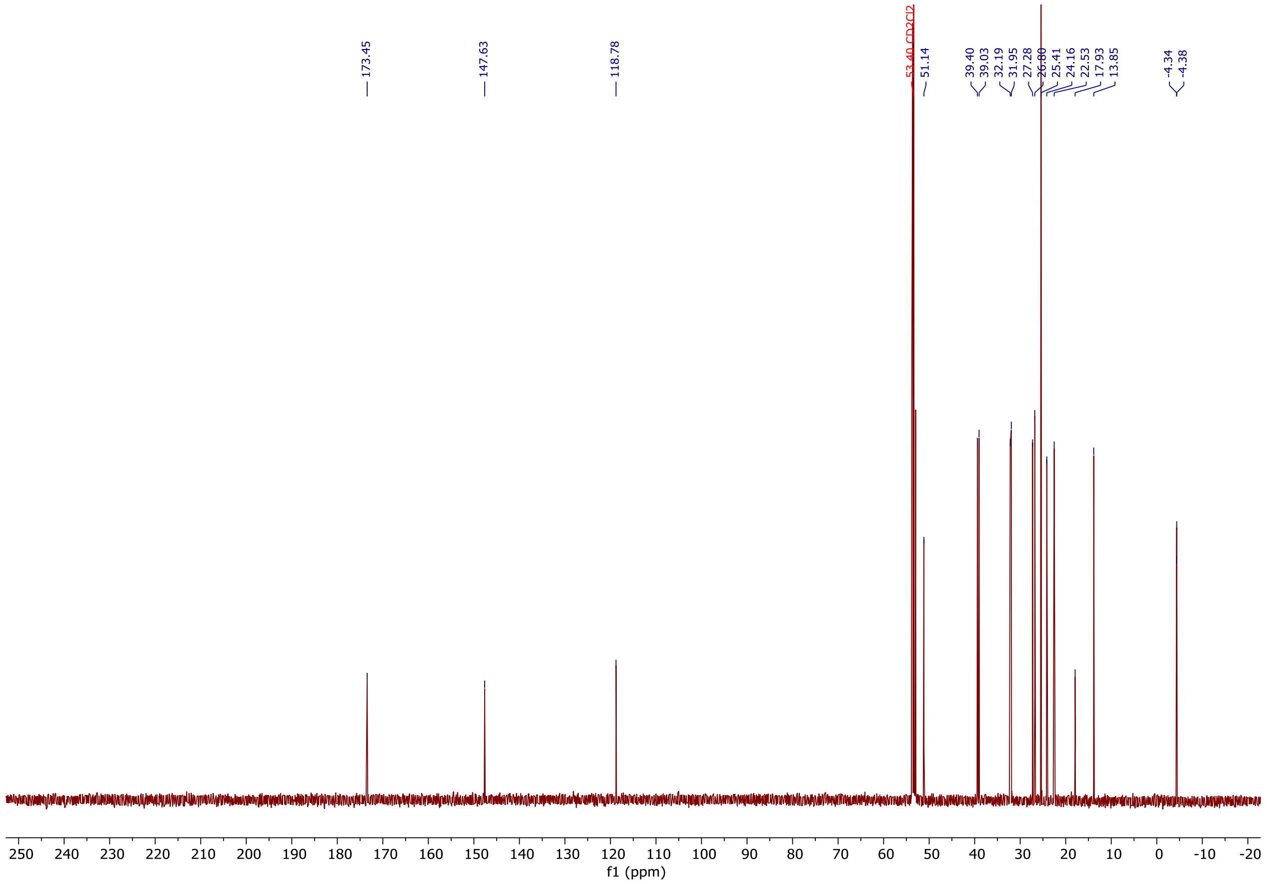

173.45
147.63
118.78
53.40 CD2Cl2
51.14
39.40
39.03
32.19
31.95
27.28
26.00
25.41
24.16
22.53
17.93
13.85
-4.34
-4.38
250 240 230 220 210 200 190 180 170 160 150 140 130 120 110 100 90 80 70 60 50 40 30 20 10 0 -10 -20
f1 (ppm)

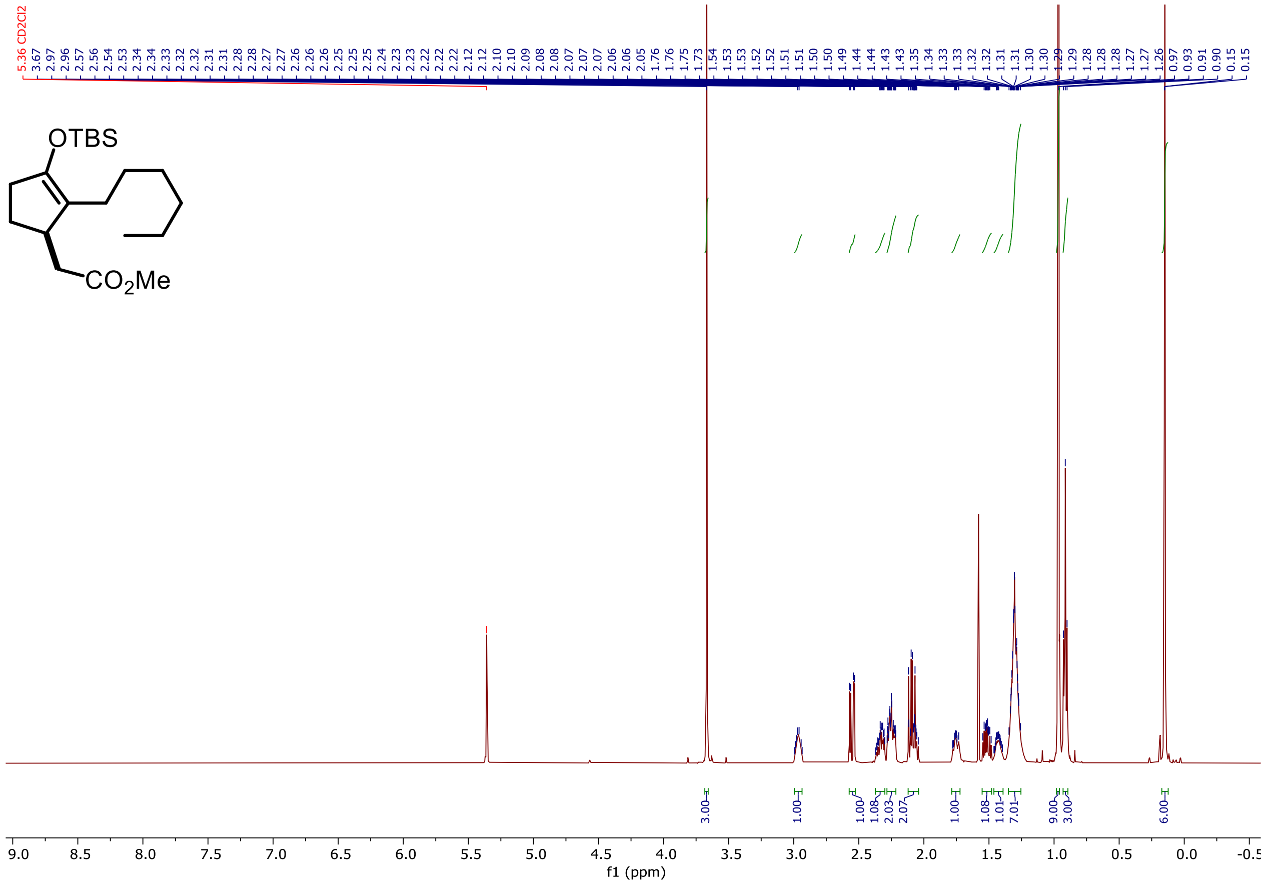
OTBS
CO2Me
f1 (ppm)

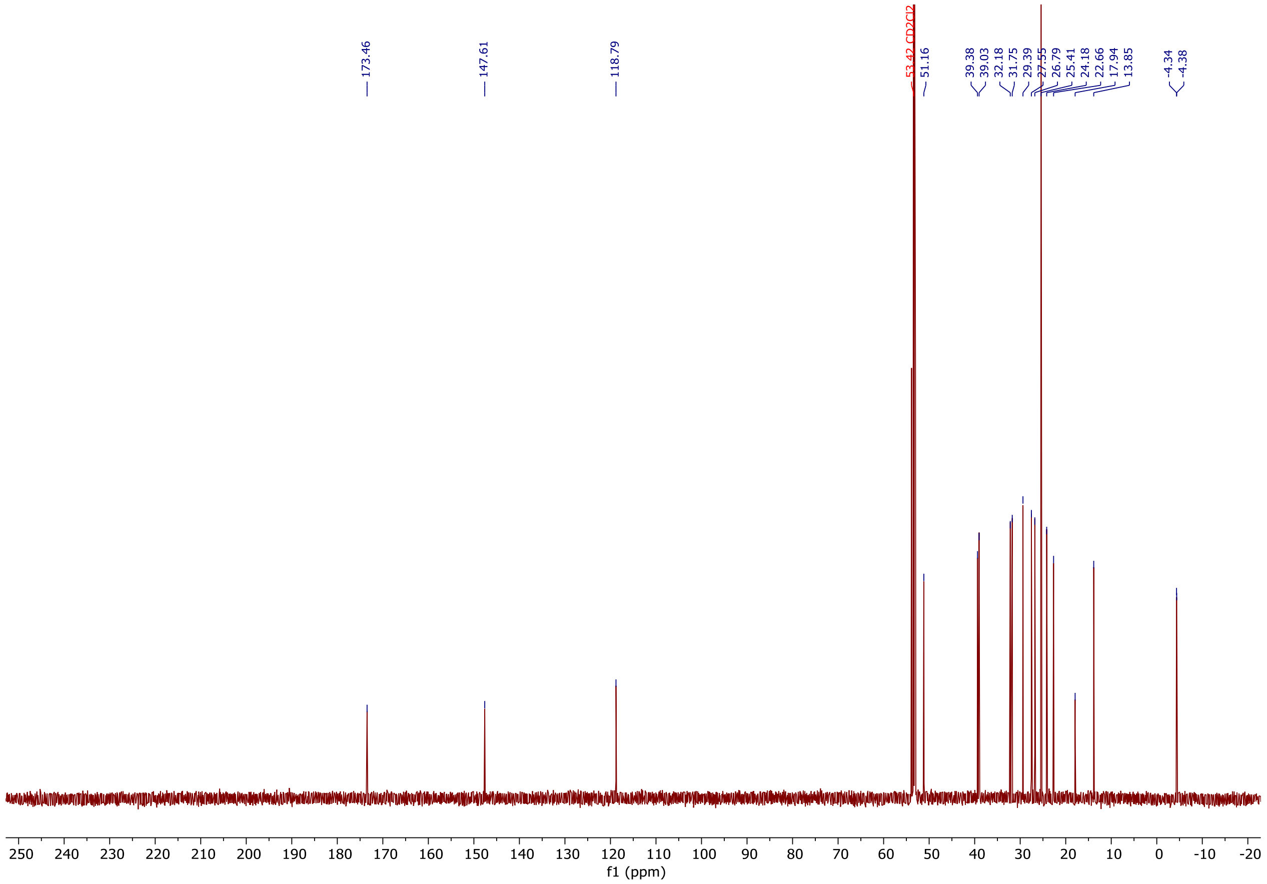
f1 (ppm)

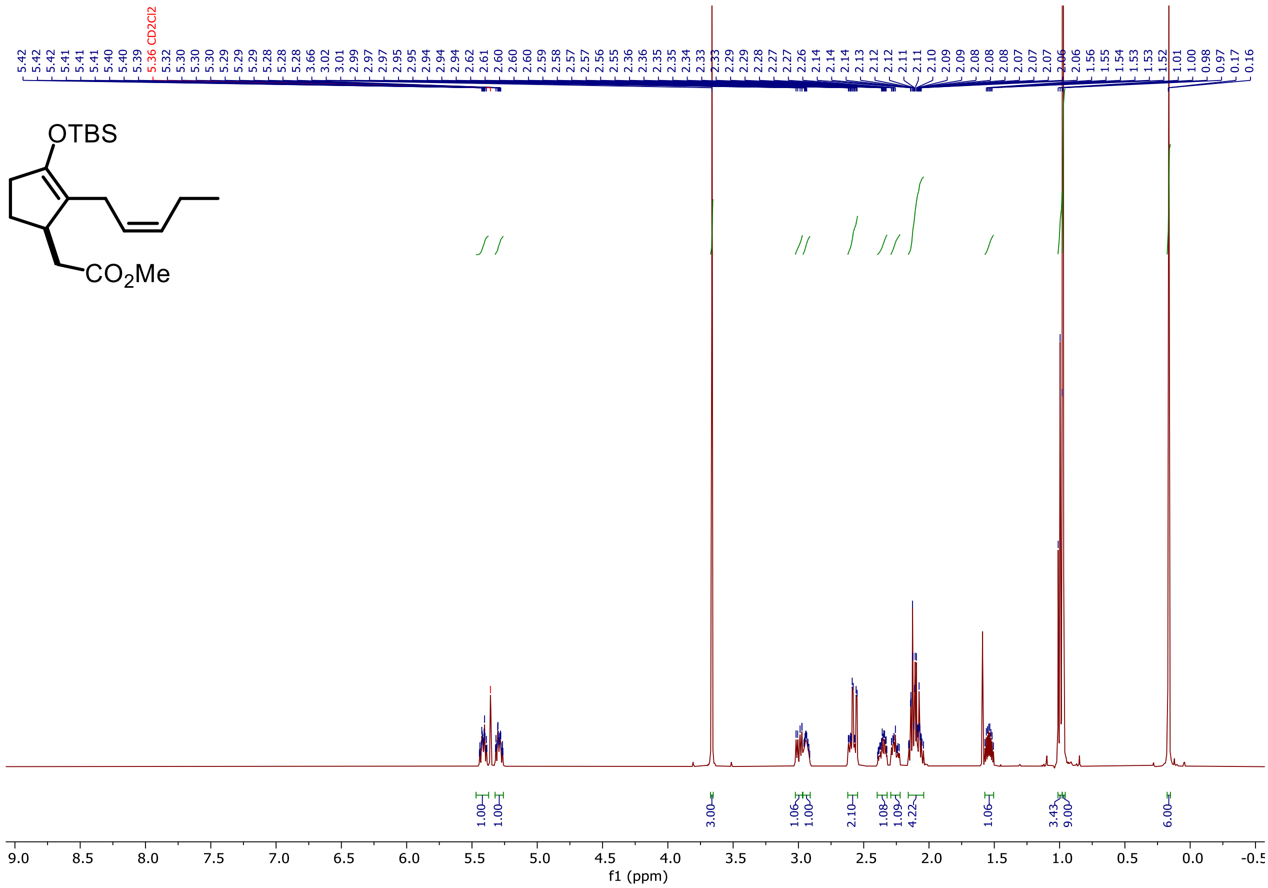
OTBS
CO_2Me
5.42 5.42 5.41 5.41 5.41 5.40 5.40 5.39 5.36 CD2Cl2 5.32 5.30 5.30 5.30 5.29 5.29 5.29 5.29 5.28 5.28 5.28 3.66 3.02 3.01 2.99 2.97 2.97 2.95 2.95 2.94 2.94 2.94 2.62 2.61 2.60 2.60 2.60 2.59 2.58 2.57 2.57 2.56 2.55 2.36 2.36 2.35 2.35 2.34 2.33 2.33 2.29 2.29 2.28 2.27 2.27 2.26 2.14 2.14 2.14 2.13 2.12 2.12 2.11 2.11 2.10 2.09 2.09 2.08 2.08 2.08 2.07 2.07 2.07 2.06 2.06 1.56 1.55 1.54 1.53 1.53 1.52 1.01 1.00 0.98 0.97 0.17 0.16
1.00 1.00 3.00 1.06 1.00 2.10 1.08 1.09 4.22 1.06 3.43 9.00 6.00
9.0 8.5 8.0 7.5 7.0 6.5 6.0 5.5 5.0 4.5 4.0 3.5 3.0 2.5 2.0 1.5 1.0 0.5 0.0 -0.5
f1 (ppm)

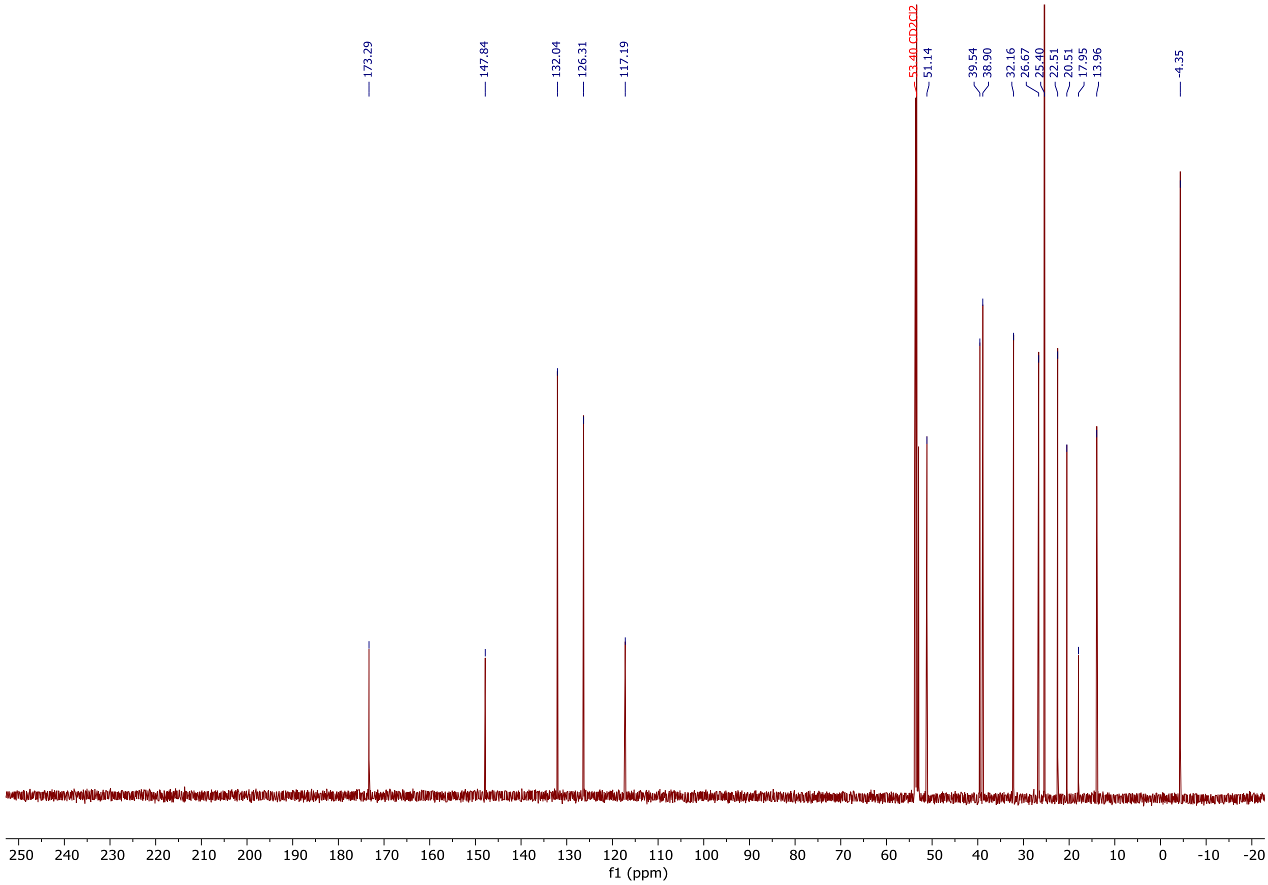
173.29 147.84 132.04 126.31 117.19 53.40 CD2Cl2 51.14 39.54 38.90 32.16 26.67 25.40 22.51 20.51 17.95 13.96 -4.35
250 240 230 220 210 200 190 180 170 160 150 140 130 120 110 100 90 80 70 60 50 40 30 20 10 0 -10 -20
f1 (ppm)

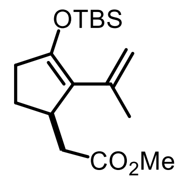

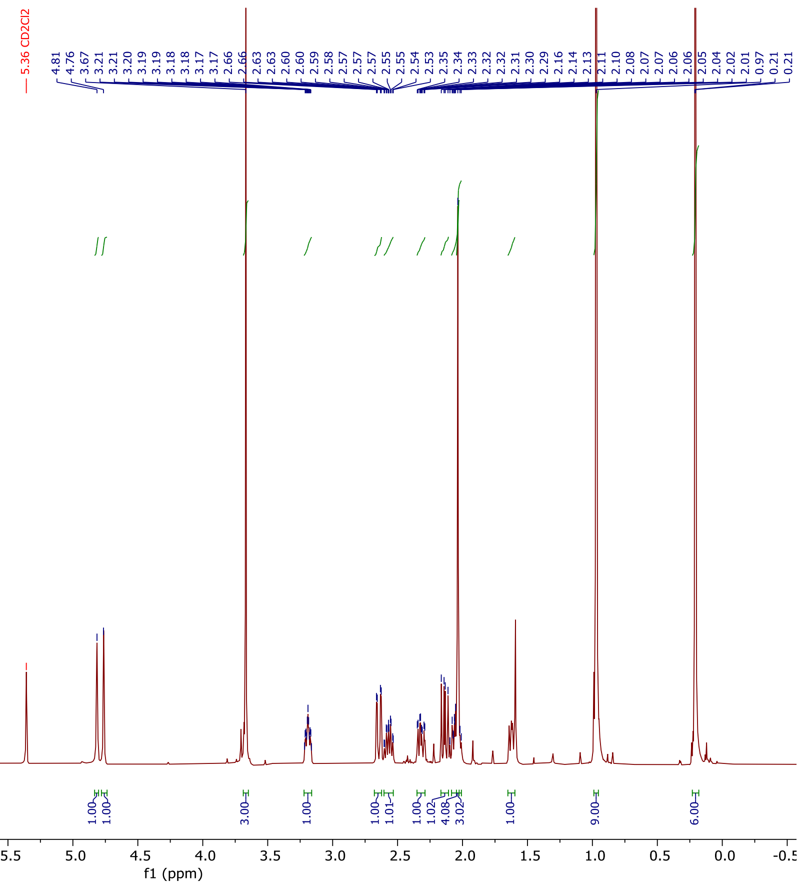

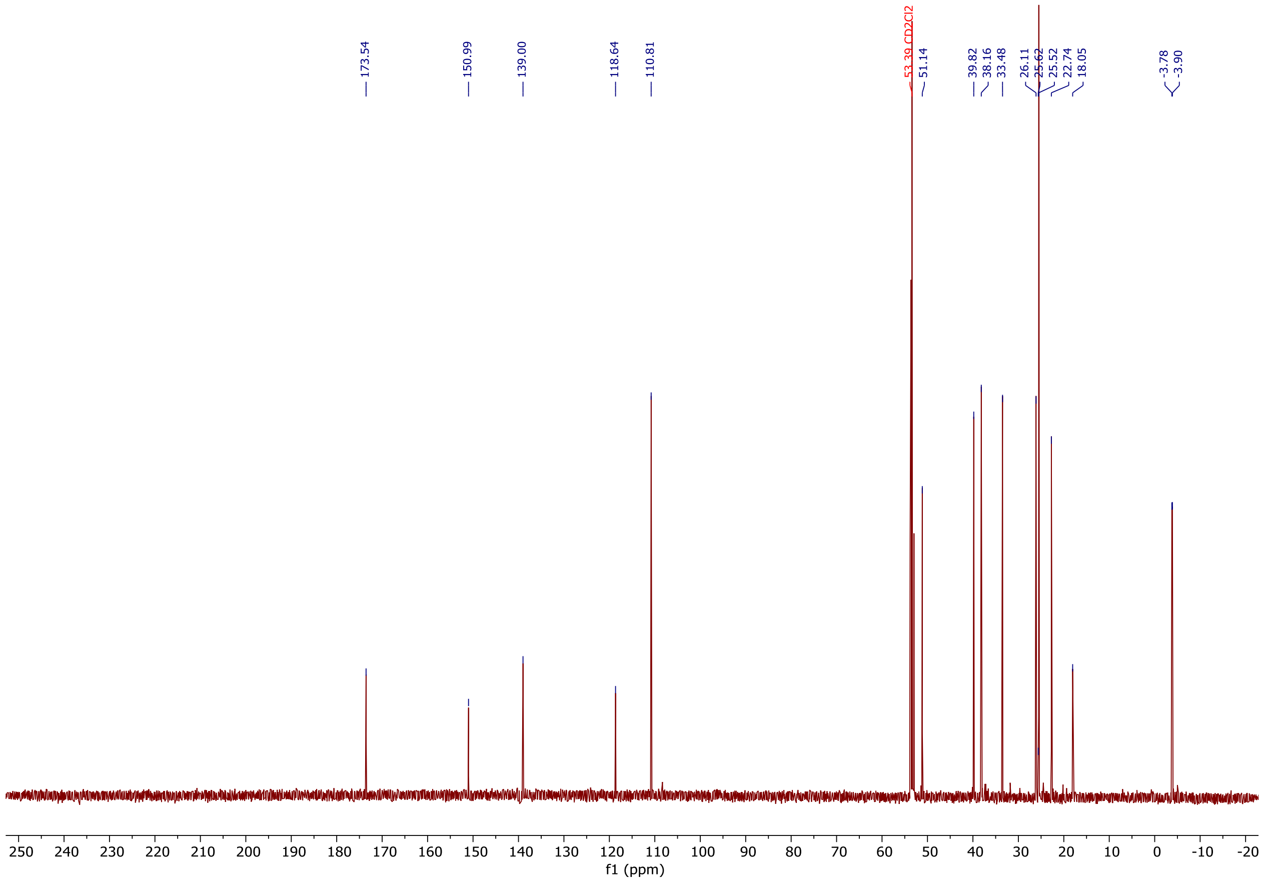

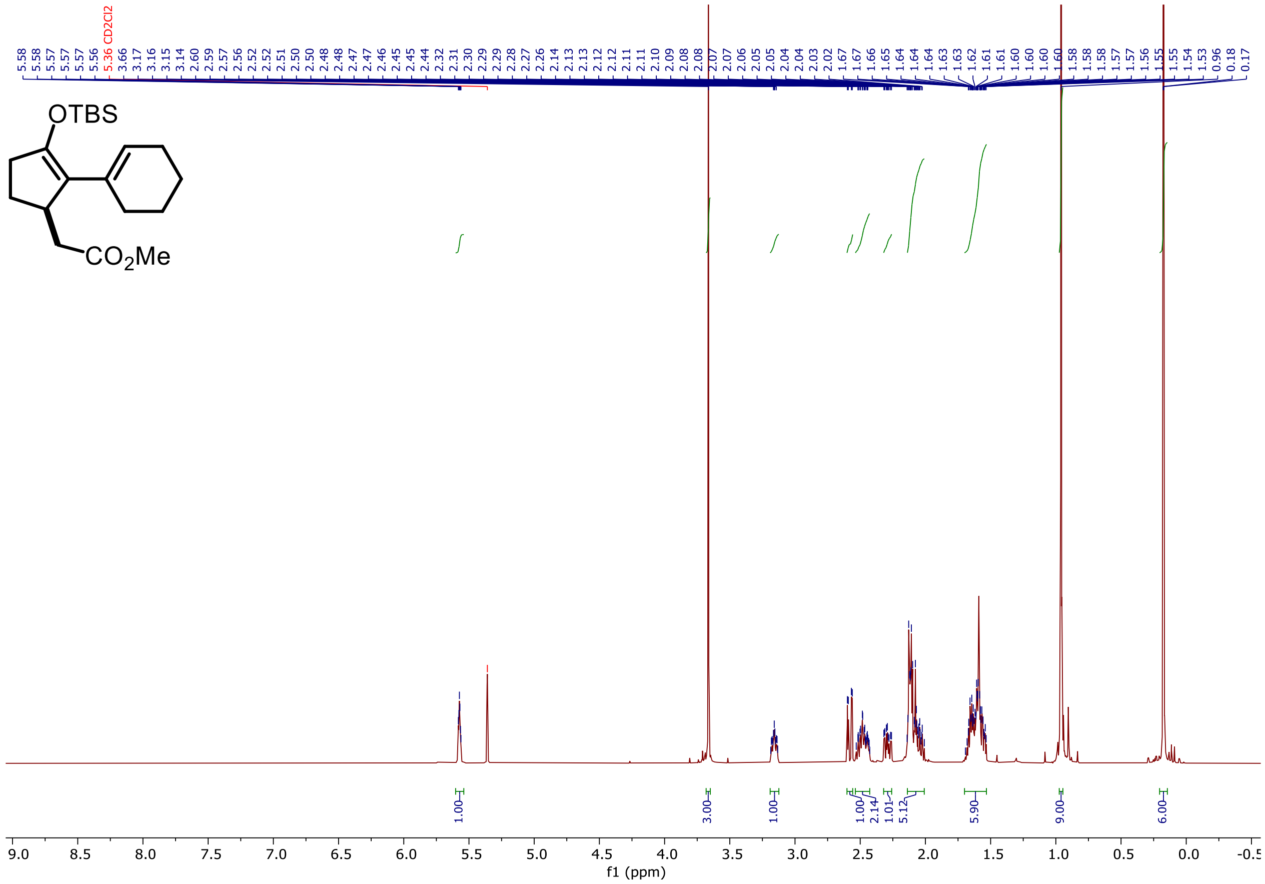

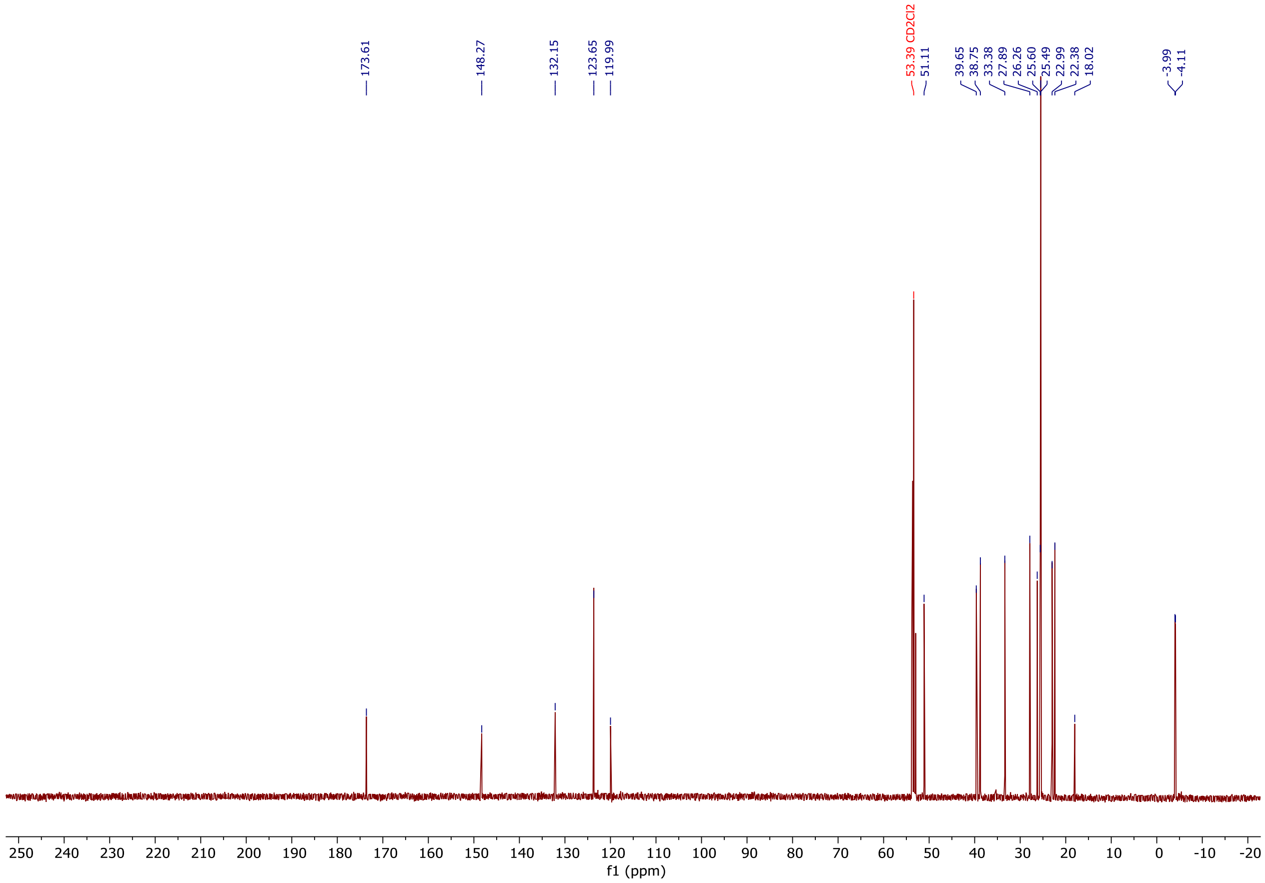

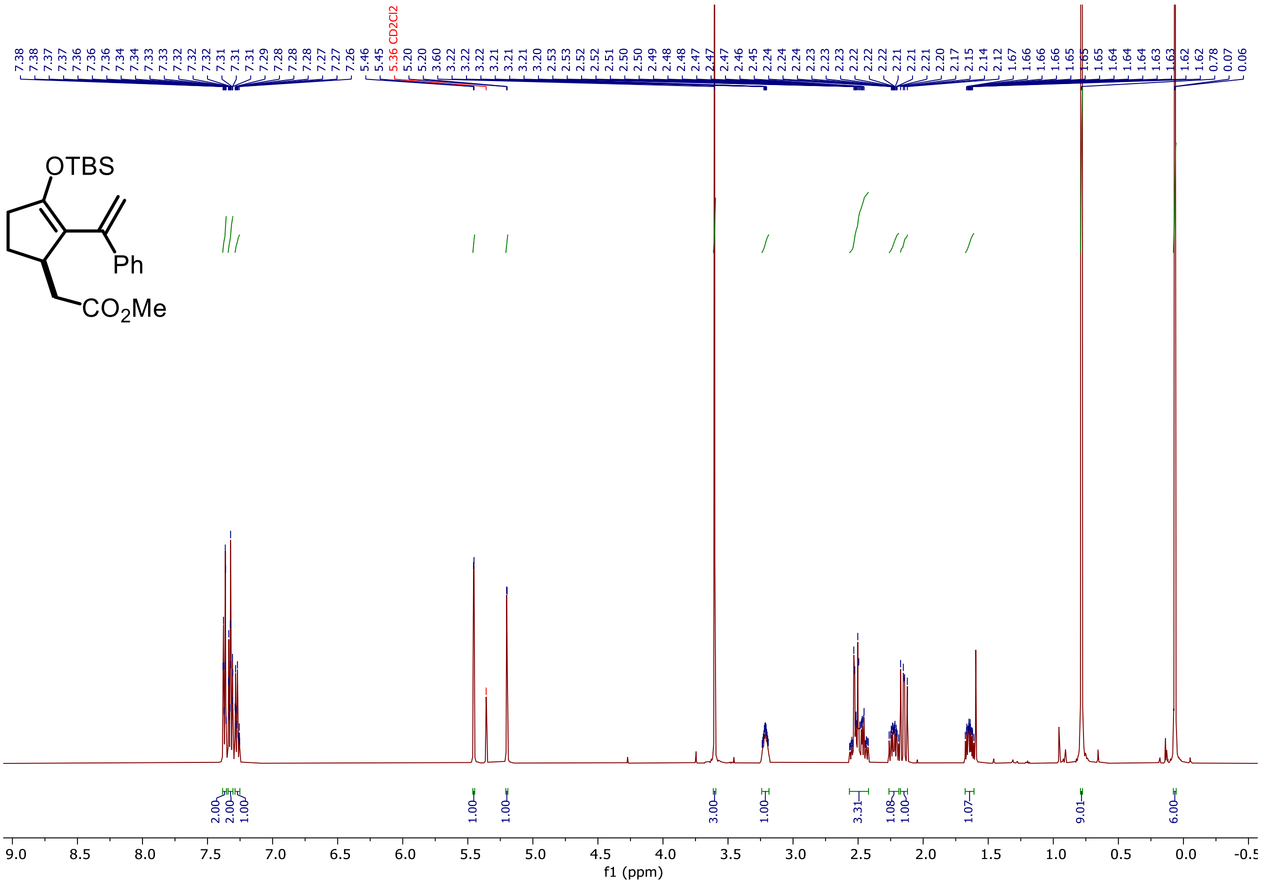
OTBS
Ph
CO2Me
7.38 7.38 7.37 7.36 7.36 7.34 7.34 7.33 7.33 7.32 7.32 7.31 7.31 7.31 7.29 7.28 7.28 7.27 7.27 7.26 5.46 5.45 5.36 CD2Cl2 5.20 5.20 3.60 3.22 3.22 3.22 3.21 3.21 3.21 3.20 2.53 2.53 2.52 2.52 2.51 2.50 2.50 2.49 2.48 2.48 2.47 2.47 2.47 2.46 2.45 2.24 2.24 2.24 2.24 2.23 2.23 2.23 2.22 2.22 2.22 2.22 2.21 2.21 2.21 2.20 2.17 2.15 2.14 2.12 1.67 1.66 1.66 1.66 1.65 1.65 1.65 1.64 1.64 1.64 1.63 1.63 1.62 1.62 0.78 0.07 0.06
2.00 2.00 1.00 1.00 1.00 3.00 1.00 3.31 1.08 1.00 1.07 9.01 6.00
9.0 8.5 8.0 7.5 7.0 6.5 6.0 5.5 5.0 4.5 4.0 3.5 3.0 2.5 2.0 1.5 1.0 0.5 0.0 -0.5
f1 (ppm)

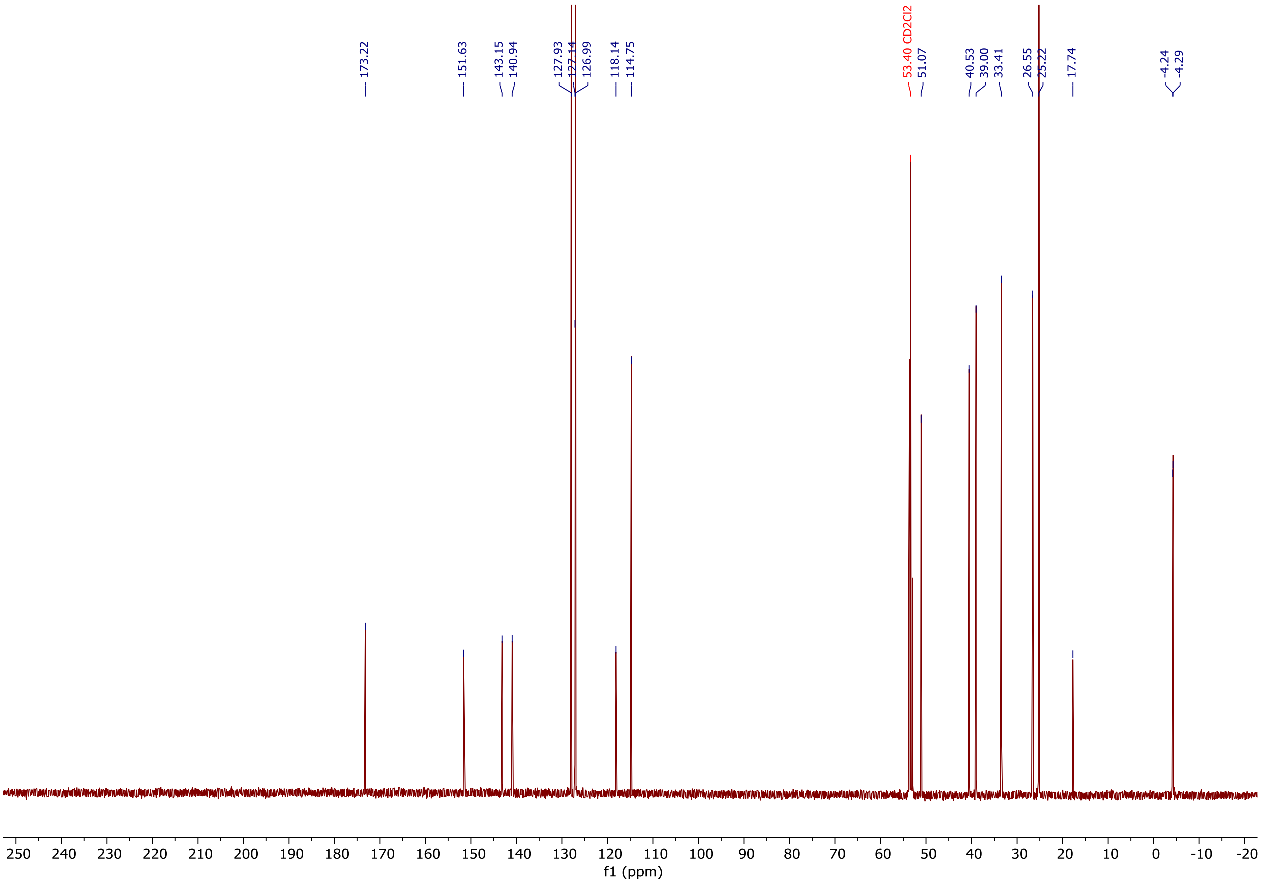
173.22 151.63 143.15 140.94 127.93 127.14 126.99 118.14 114.75 53.40 CD2Cl2 51.07 40.53 39.00 33.41 26.55 25.22 17.74 -4.24 -4.29
250 240 230 220 210 200 190 180 170 160 150 140 130 120 110 100 90 80 70 60 50 40 30 20 10 0 -10 -20
f1 (ppm)

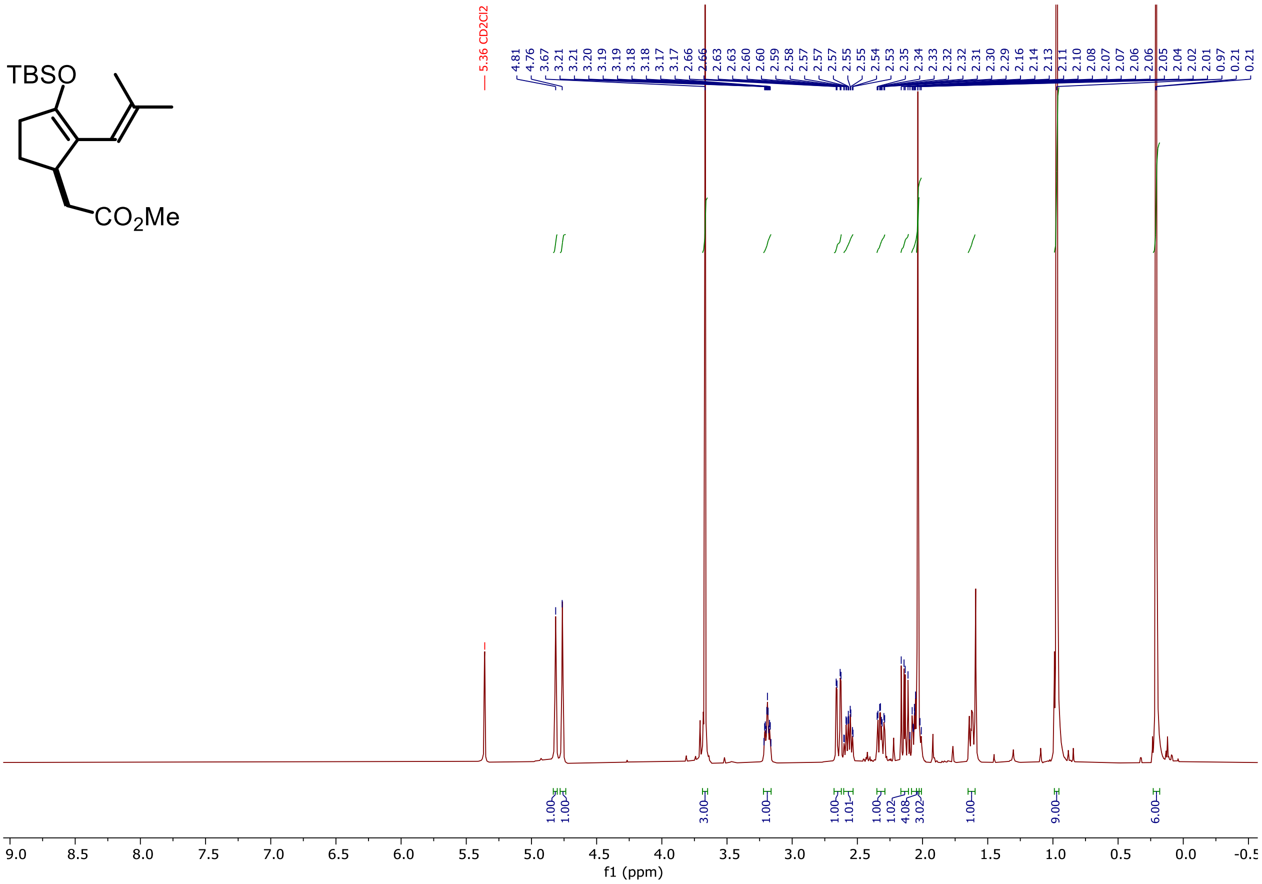
TBSO
CO_2Me
5.36 CD2Cl2
4.81
4.76
3.67
3.21
3.21
3.20
3.19
3.19
3.18
3.18
3.17
3.17
2.66
2.66
2.63
2.63
2.60
2.60
2.59
2.58
2.58
2.57
2.57
2.57
2.55
2.55
2.54
2.53
2.35
2.34
2.33
2.32
2.32
2.31
2.30
2.29
2.16
2.14
2.13
2.11
2.10
2.08
2.07
2.07
2.06
2.05
2.04
2.02
2.01
0.97
0.21
0.21
1.00
1.00
3.00
1.00
1.00
1.01
1.00
1.02
4.08
3.02
1.00
9.00
6.00
9.0
8.5
8.0
7.5
7.0
6.5
6.0
5.5
5.0
4.5
4.0
3.5
3.0
2.5
2.0
1.5
1.0
0.5
0.0
-0.5
f1 (ppm)

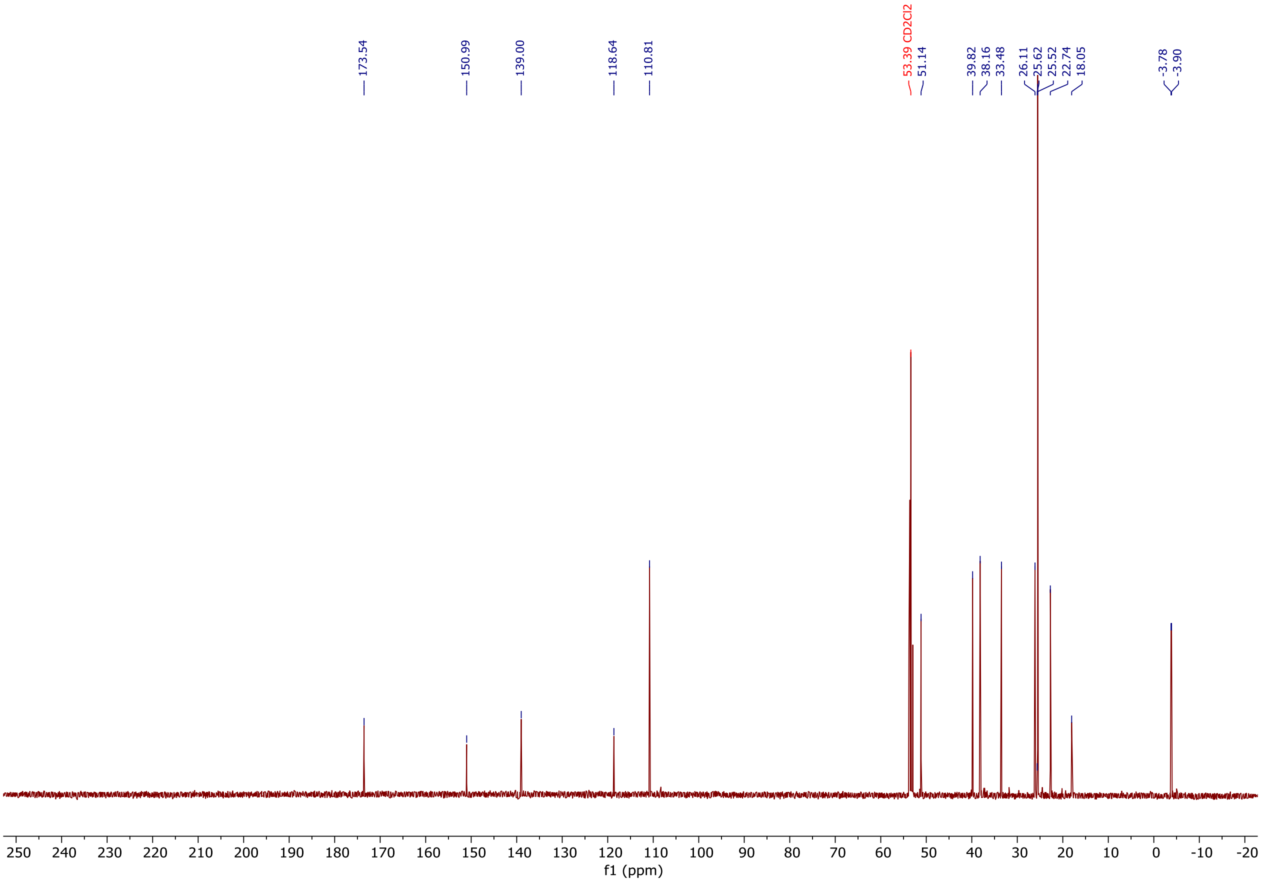
173.54
150.99
139.00
118.64
110.81
53.39 CD2Cl2
51.14
39.82
38.16
33.48
26.11
25.62
25.52
22.74
18.05
-3.78
-3.90
250
240
230
220
210
200
190
180
170
160
150
140
130
120
110
100
90
80
70
60
50
40
30
20
10
0
-10
-20
f1 (ppm)

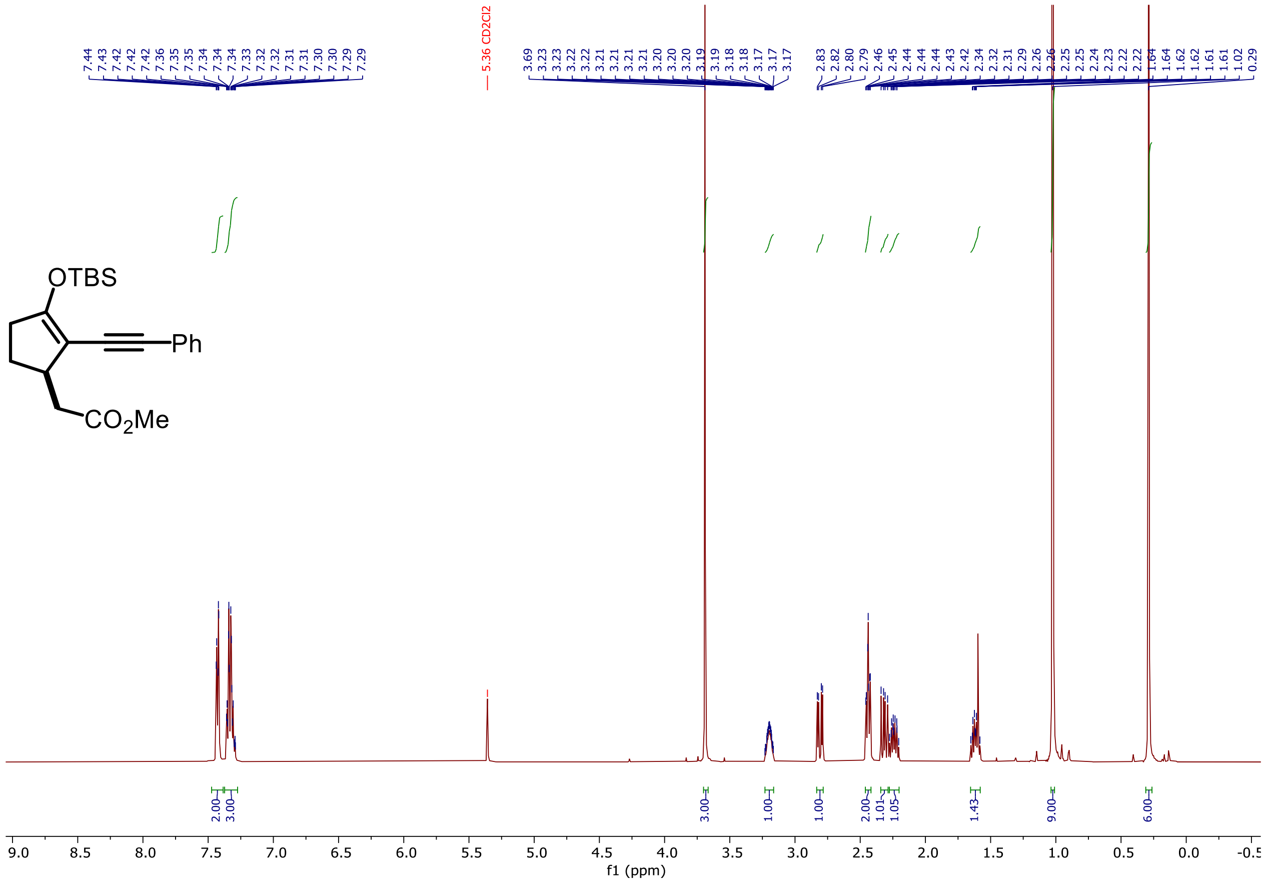

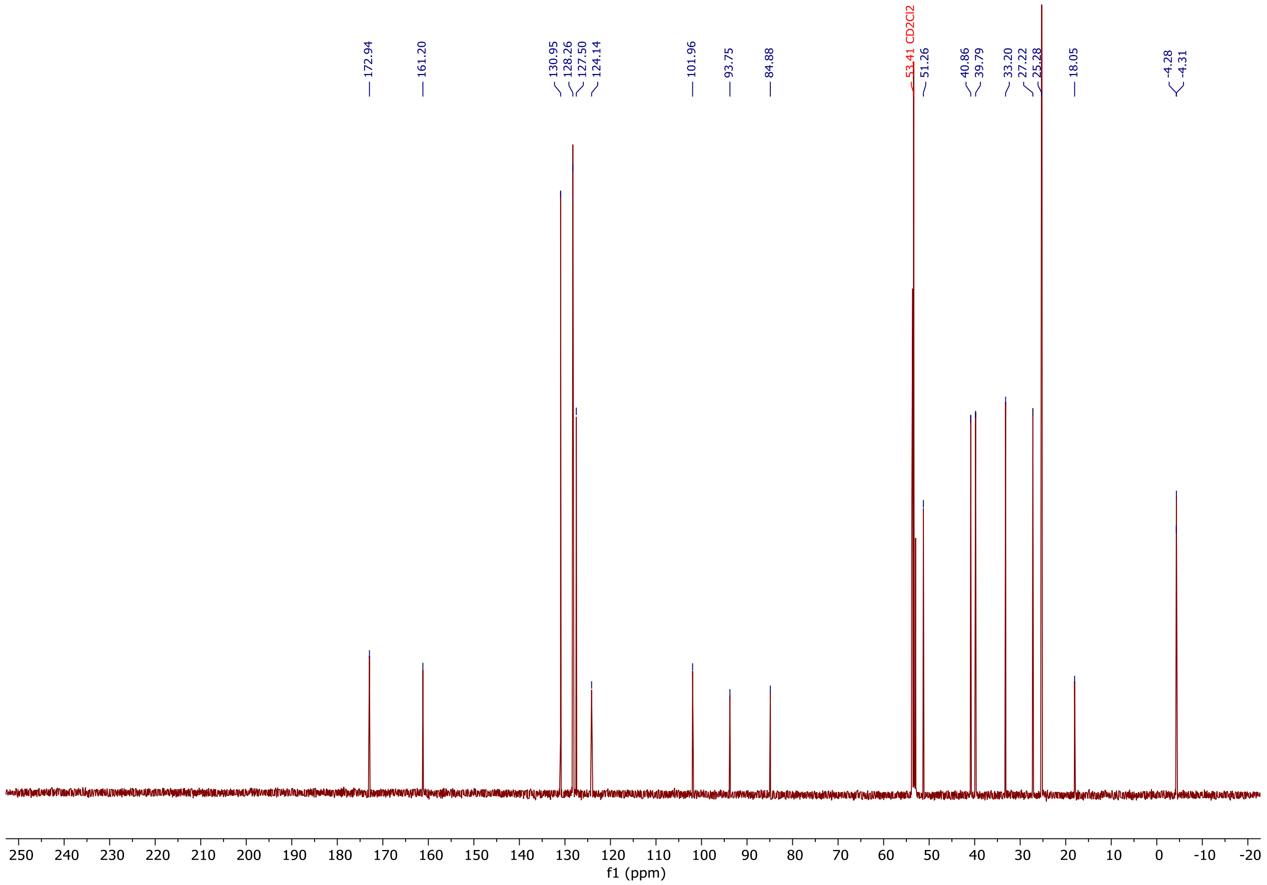

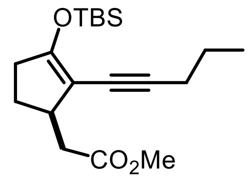
OTBS
CO_2Me

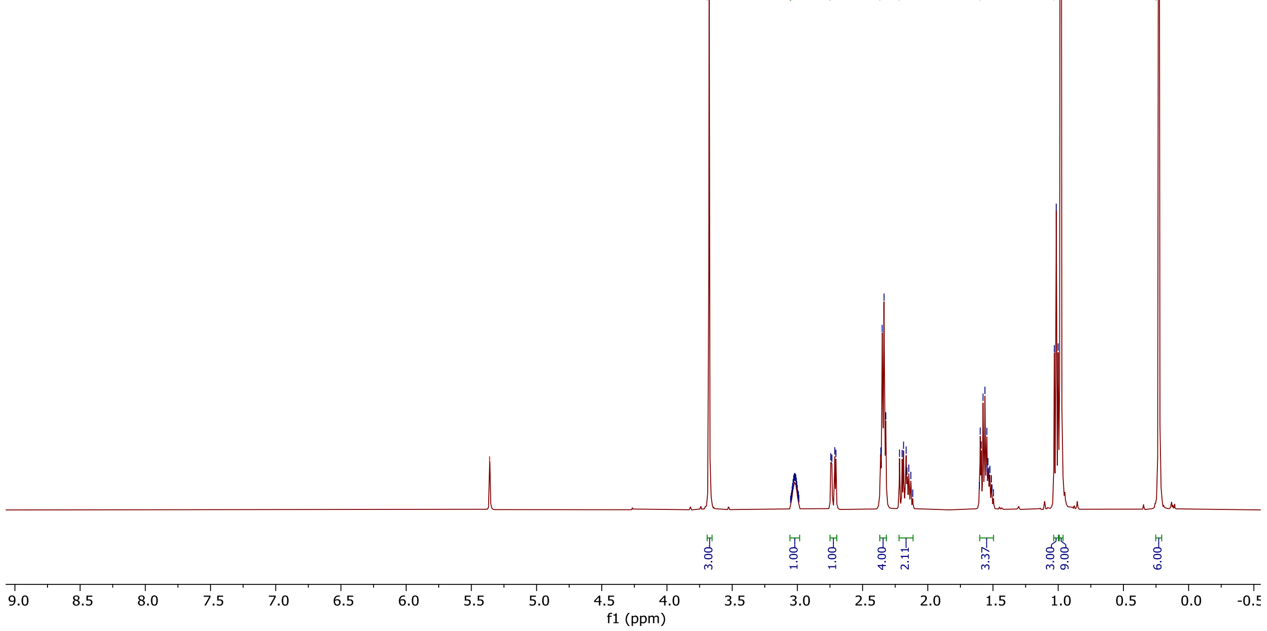
5.36 CD2Cl2
3.68
3.05
3.05
3.04
3.04
3.03
3.03
3.02
3.02
3.02
3.01
3.01
3.00
3.00
3.00
2.99
2.99
2.74
2.74
2.71
2.70
2.36
2.35
2.34
2.32
2.22
2.20
2.19
2.17
2.16
2.15
2.13
1.60
1.59
1.58
1.56
1.55
1.55
1.54
1.53
1.53
1.52
1.03
1.02
1.00
0.98
0.23
3.00
1.00
1.00
4.00
2.11
3.37
3.00
9.00
6.00
f1 (ppm)

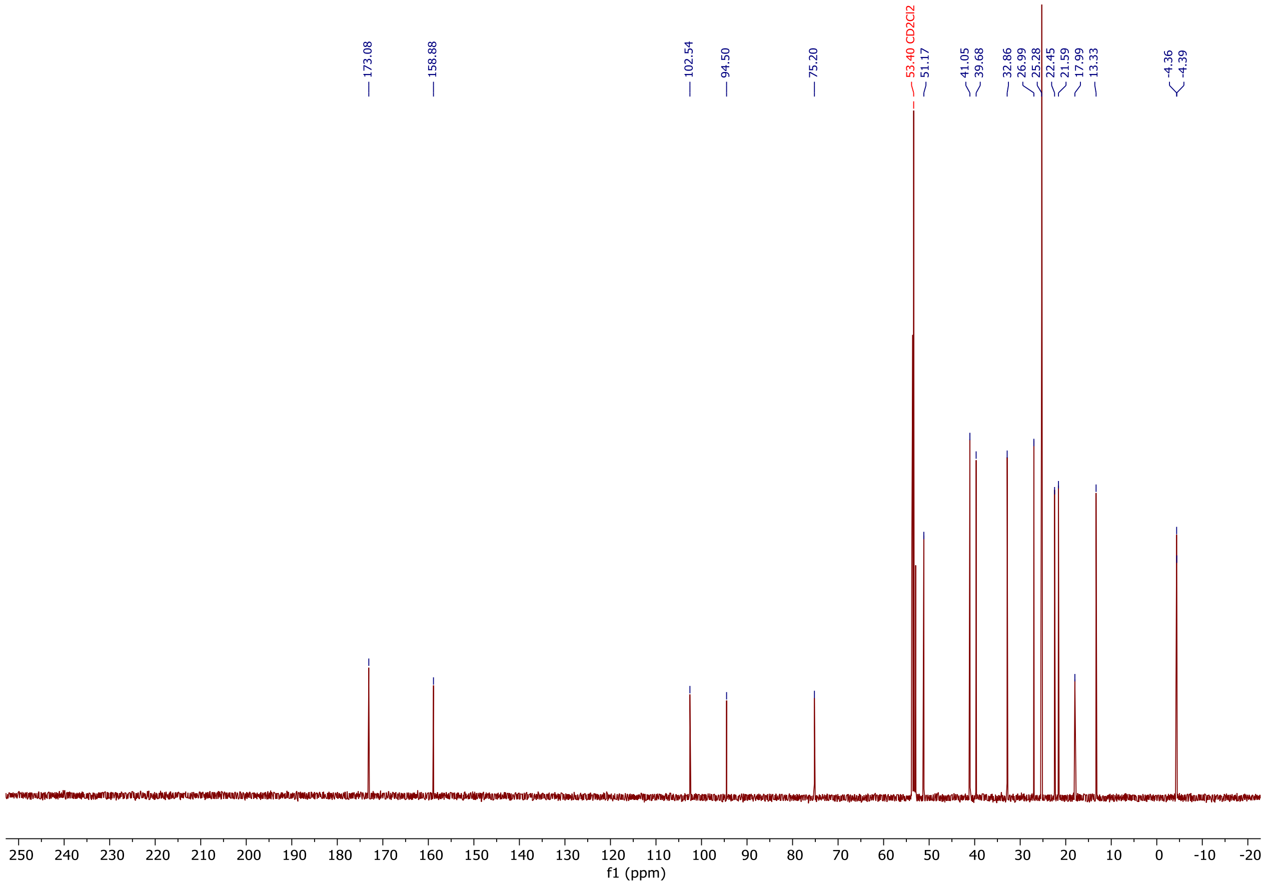
173.08
158.88
102.54
94.50
75.20
53.40 CD2Cl2
51.17
41.05
39.68
32.86
26.99
25.28
22.45
21.59
17.99
13.33
-4.36
-4.39
f1 (ppm)

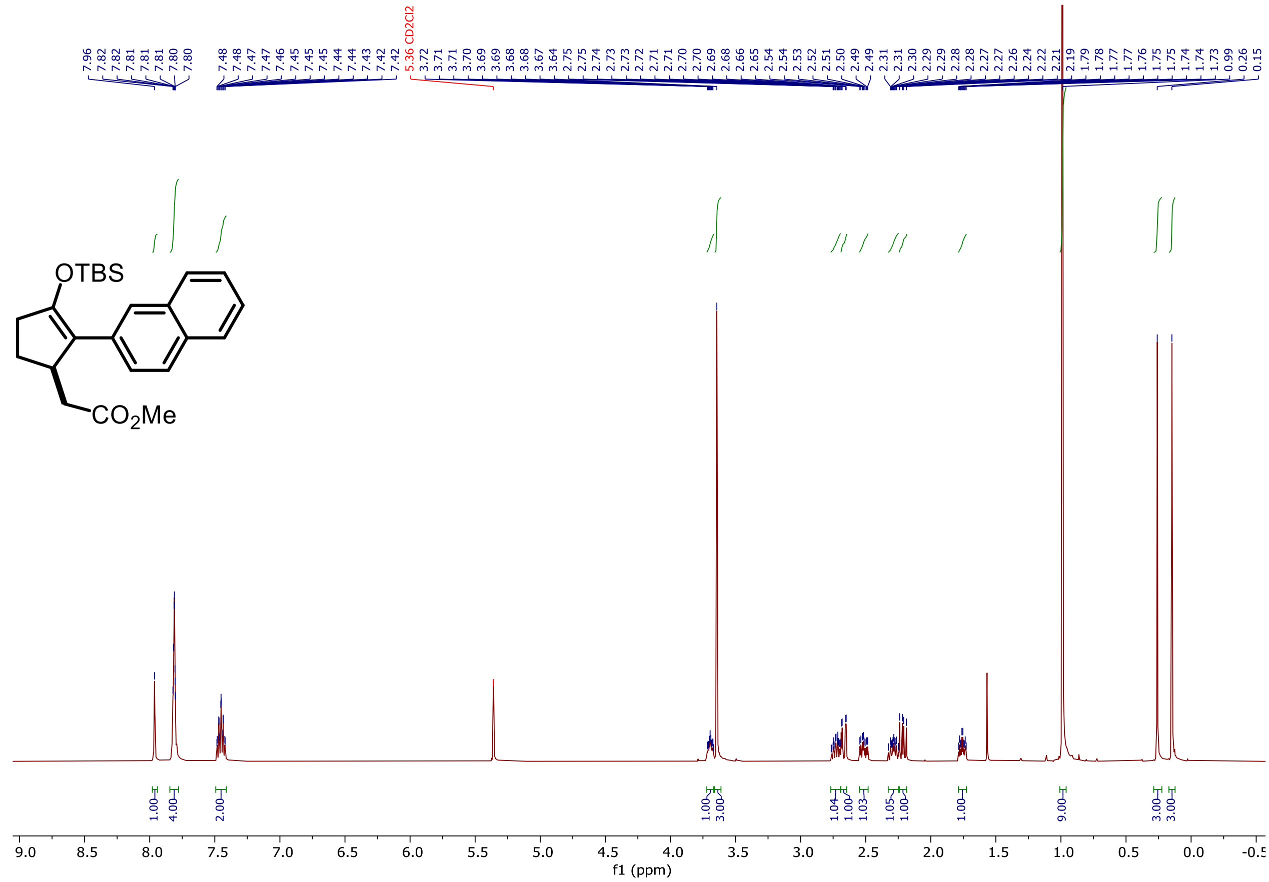
7.96
7.82
7.82
7.81
7.81
7.81
7.80
7.80
7.48
7.48
7.47
7.47
7.46
7.45
7.45
7.45
7.44
7.44
7.43
7.42
7.42
5.36 CD2Cl2
3.72
3.71
3.71
3.70
3.69
3.69
3.68
3.68
3.67
3.64
2.75
2.75
2.74
2.73
2.73
2.72
2.71
2.71
2.70
2.70
2.69
2.68
2.66
2.65
2.54
2.54
2.53
2.52
2.51
2.50
2.49
2.49
2.31
2.31
2.30
2.29
2.29
2.28
2.28
2.27
2.27
2.26
2.24
2.22
2.21
2.19
1.79
1.78
1.77
1.77
1.76
1.75
1.75
1.74
1.74
1.73
0.99
0.26
0.15
1.00
4.00
2.00
1.00
3.00
1.04
1.00
1.03
1.05
1.00
1.00
9.00
3.00
3.00
f1 (ppm)
OTBS
CO2Me

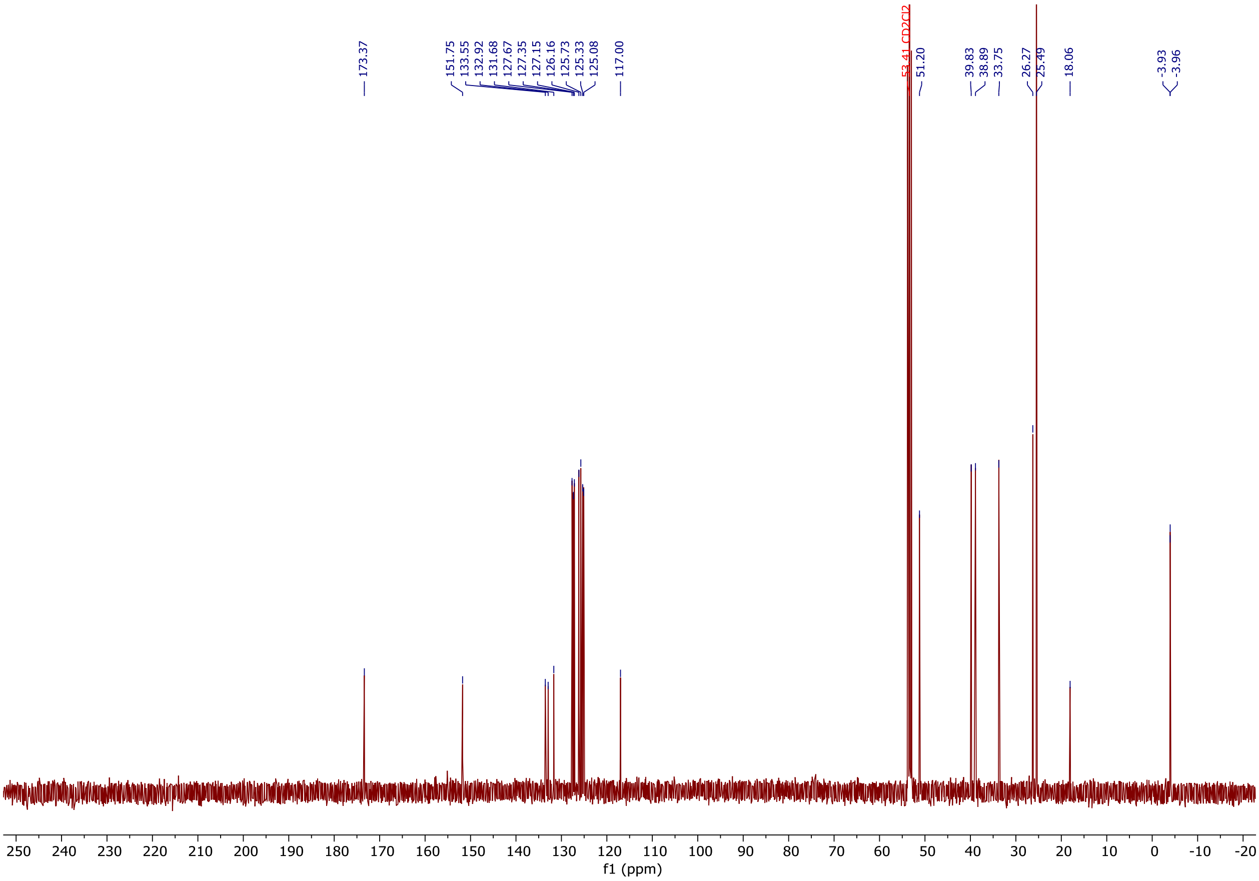
173.37
151.75
133.55
132.92
131.68
127.67
127.35
127.15
126.16
125.73
125.33
125.08
117.00
53.41 CD2Cl2
51.20
39.83
38.89
33.75
26.27
25.49
18.06
-3.93
-3.96
f1 (ppm)

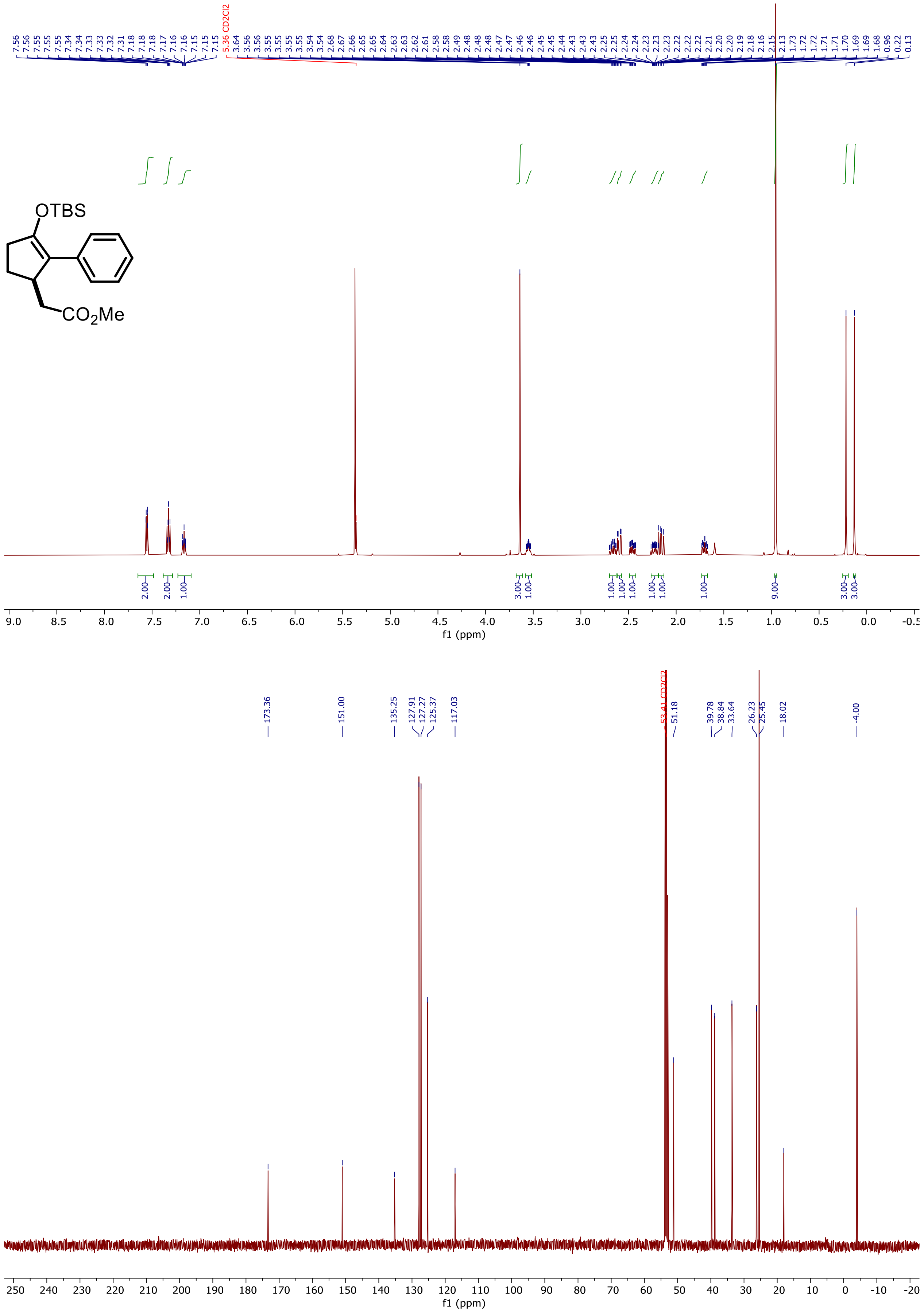
OTBS
CO2Me
f1 (ppm)
f1 (ppm)

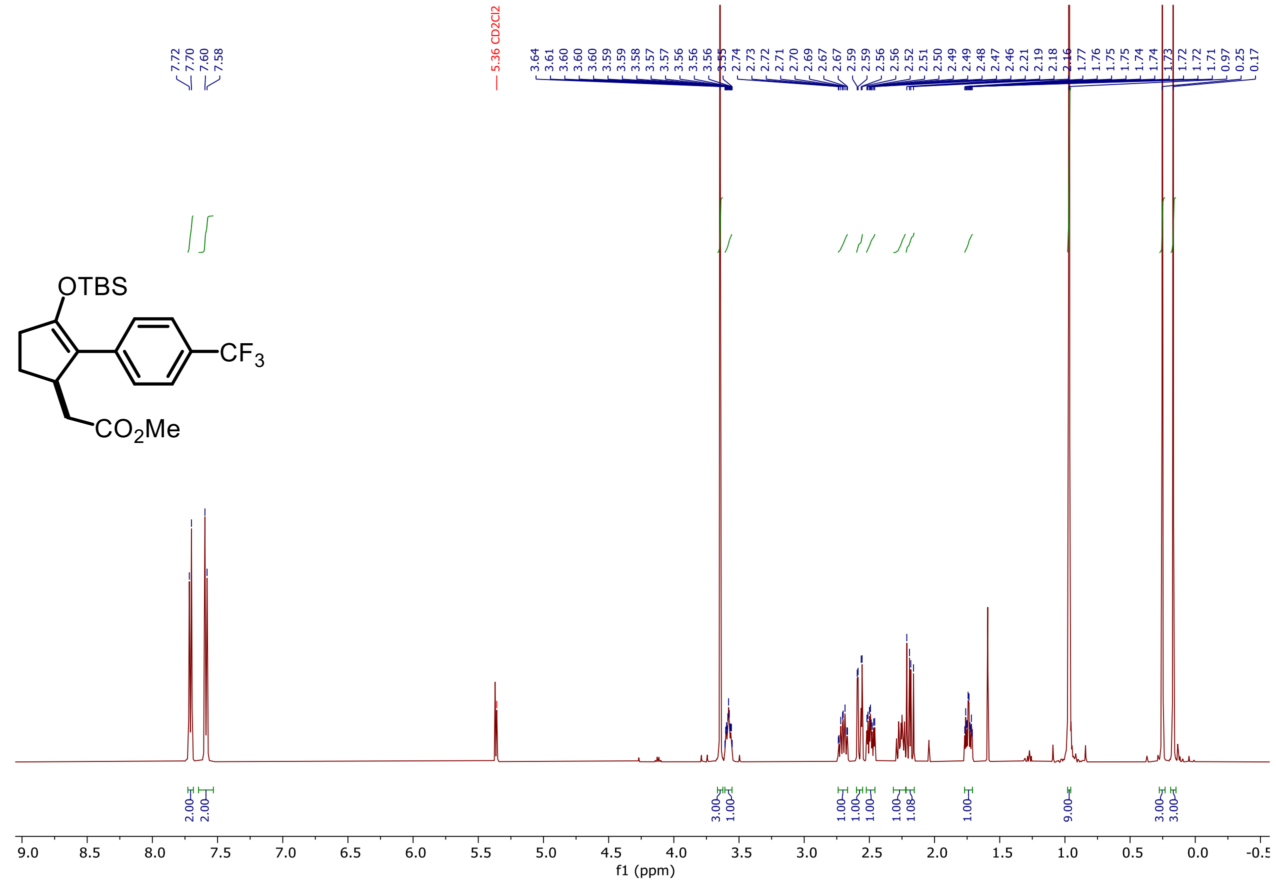

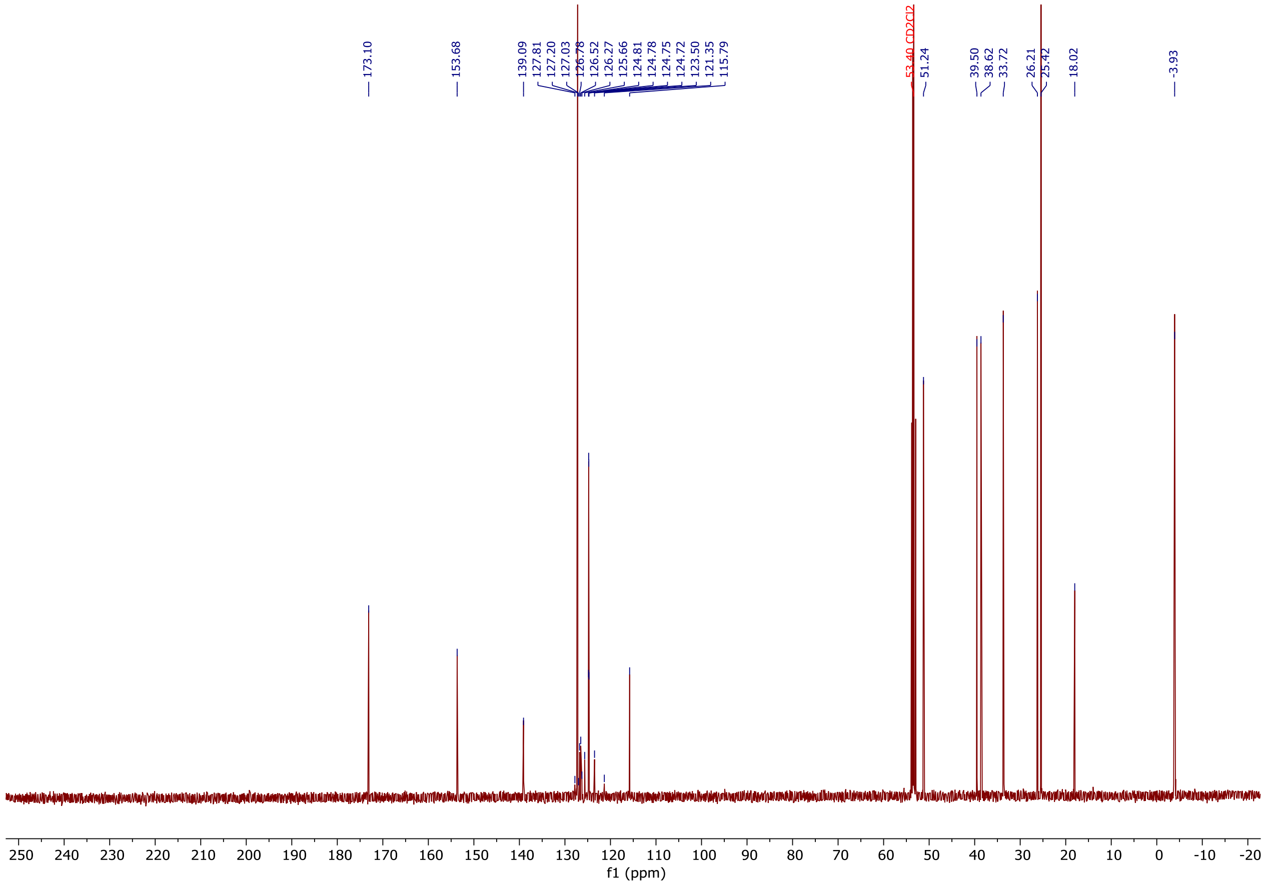

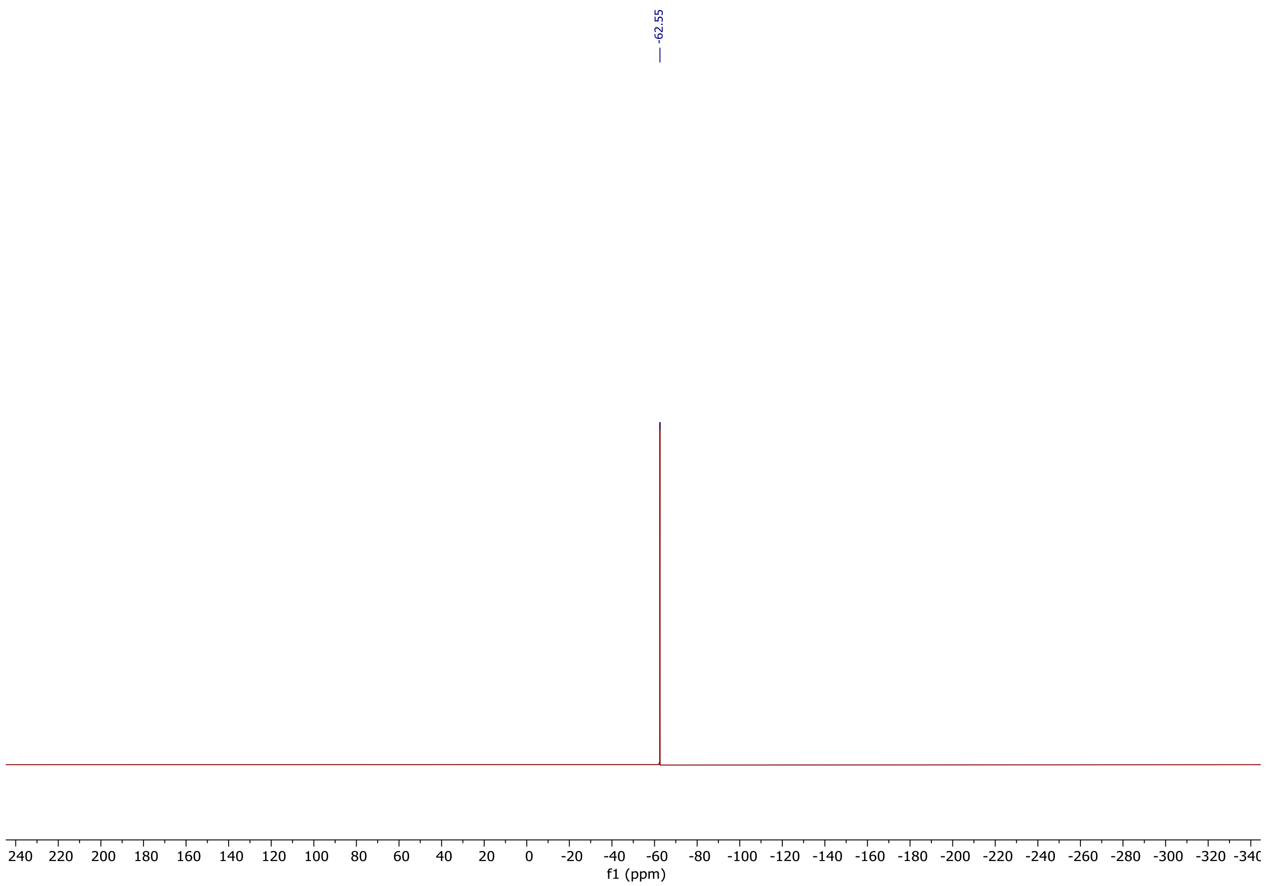
-62.55
240 220 200 180 160 140 120 100 80 60 40 20 0 -20 -40 -60 -80 -100 -120 -140 -160 -180 -200 -220 -240 -260 -280 -300 -320 -340
f1 (ppm)

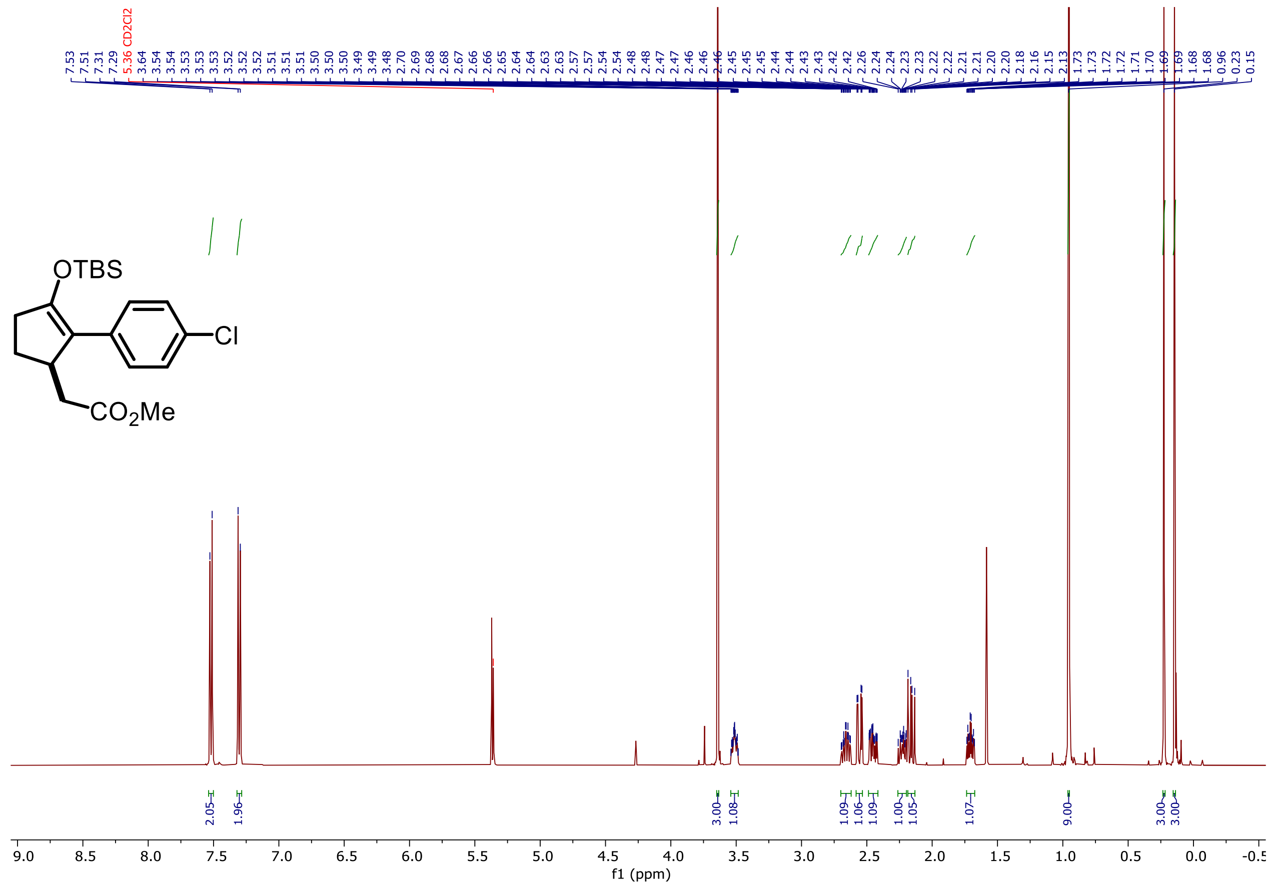
7.53
7.51
7.31
7.29
5.36 CD2Cl2
3.64
3.54
3.54
3.53
3.53
3.53
3.52
3.52
3.52
3.51
3.51
3.50
3.50
3.50
3.49
3.49
3.48
2.70
2.69
2.68
2.68
2.67
2.66
2.66
2.65
2.64
2.64
2.63
2.63
2.57
2.57
2.54
2.54
2.48
2.48
2.47
2.47
2.46
2.46
2.46
2.45
2.45
2.45
2.44
2.44
2.43
2.43
2.42
2.42
2.42
2.26
2.24
2.24
2.23
2.23
2.22
2.22
2.22
2.21
2.21
2.20
2.20
2.18
2.16
2.15
2.13
1.73
1.73
1.72
1.72
1.71
1.70
1.69
1.68
1.68
0.96
0.23
0.15
OTBS
Cl
CO2Me
2.05
1.96
3.00
1.08
1.09
1.06
1.09
1.00
1.05
1.07
9.00
3.00
3.00
f1 (ppm)

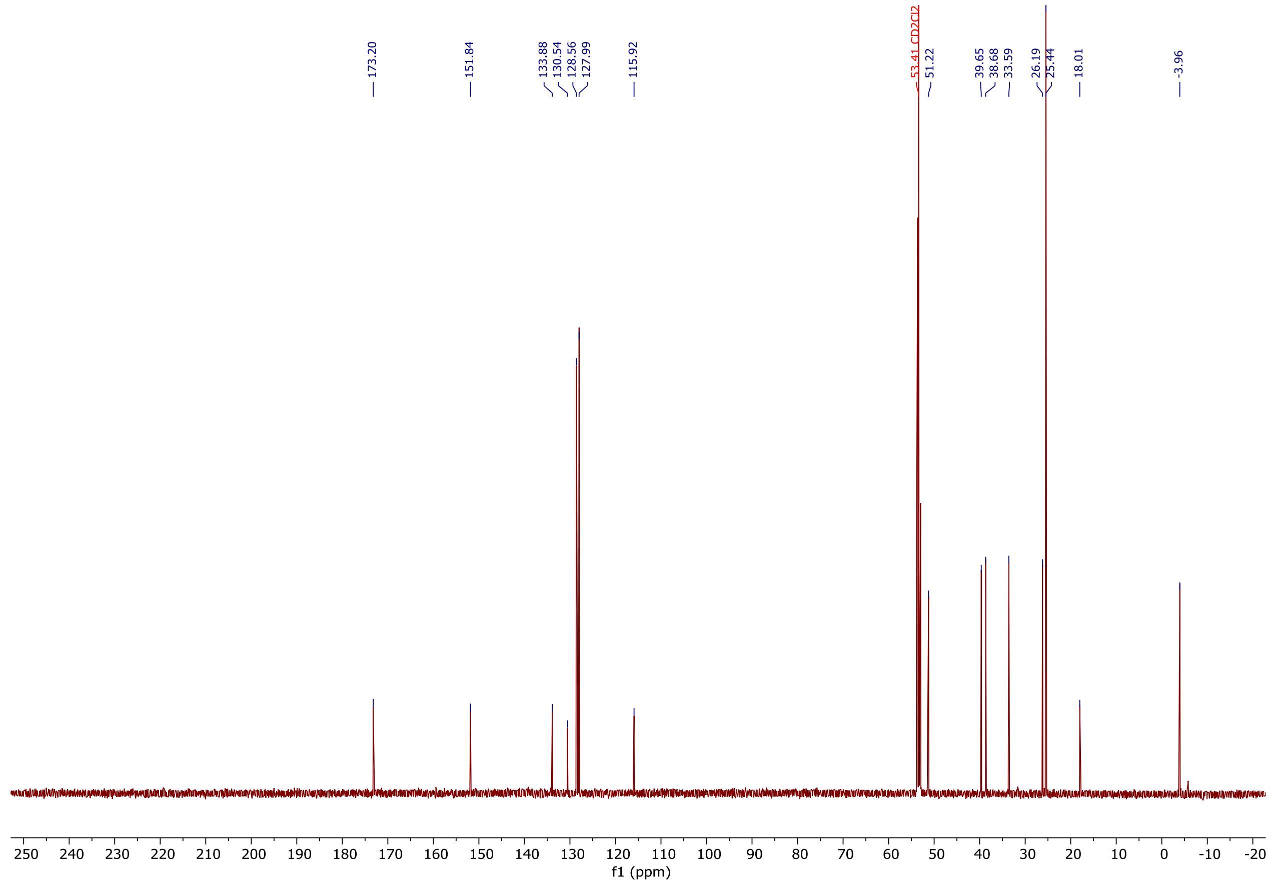
173.20
151.84
133.88
130.54
128.56
127.99
115.92
53.41 CD2Cl2
51.22
39.65
38.68
33.59
26.19
25.44
18.01
-3.96
f1 (ppm)

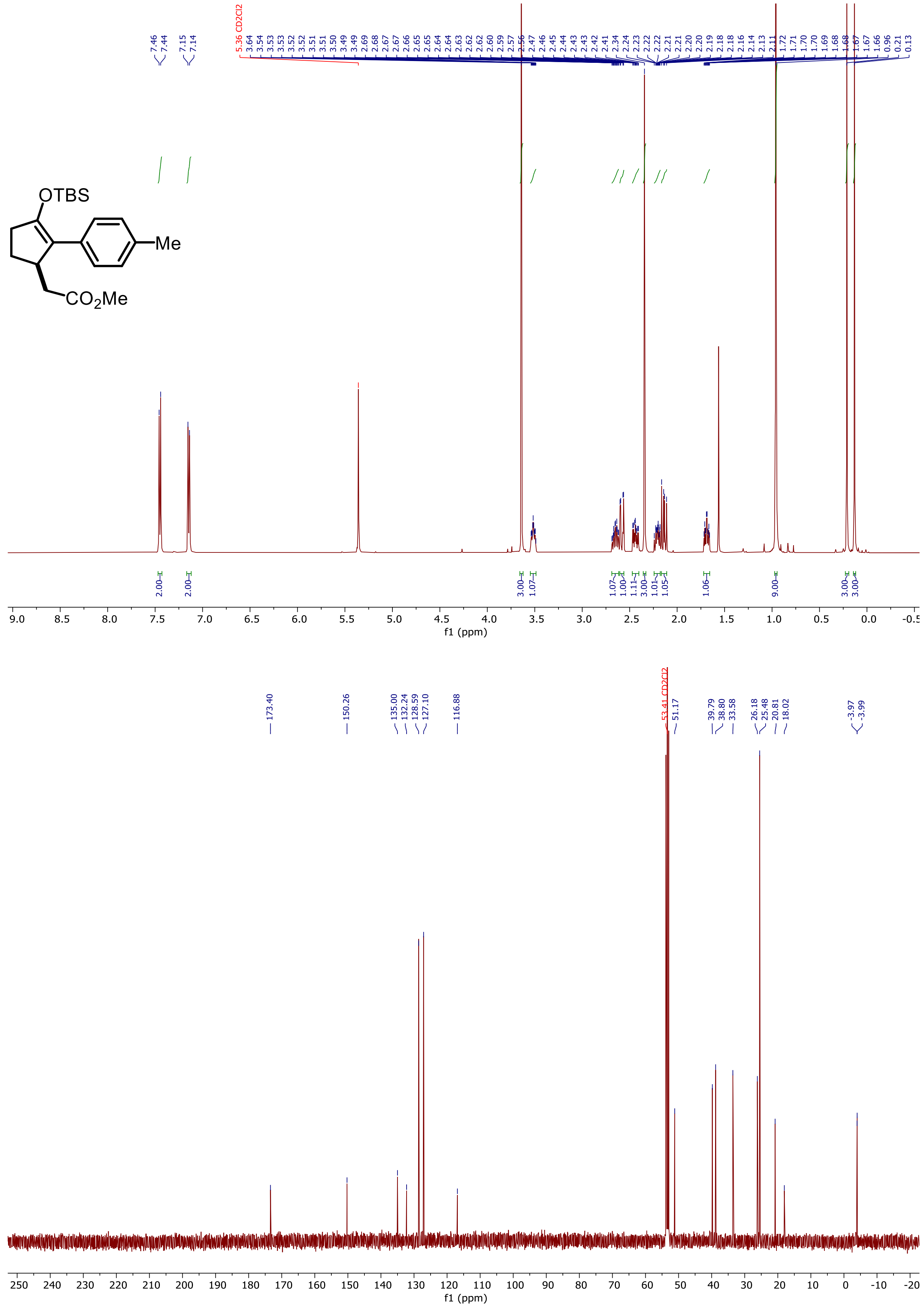

7.46
7.44
7.15
7.14
5.36 CD2Cl2
3.64 3.54 3.53 3.53 3.52 3.52 3.51 3.51 3.50 3.49 3.49 2.69 2.68 2.67 2.67 2.66 2.65 2.65 2.64 2.64 2.63 2.62 2.62 2.60 2.59 2.57 2.56 2.47 2.46 2.45 2.44 2.43 2.43 2.42 2.41 2.34 2.24 2.23 2.22 2.22 2.21 2.21 2.20 2.20 2.19 2.18 2.18 2.16 2.14 2.13 2.11 1.72 1.71 1.70 1.70 1.69 1.68 1.68 1.67 1.67 1.66 0.96 0.21 0.13
OTBS
Me
CO_2Me
2.00
2.00
3.00
1.07
1.07
1.00
1.11
3.00
1.01
1.05
1.06
9.00
3.00
3.00
f1 (ppm)
173.40
150.26
135.00
132.24
128.59
127.10
116.88
53.41 CD2Cl2
51.17
39.79
38.80
33.58
26.18
25.48
20.81
18.02
-3.97
-3.99
f1 (ppm)

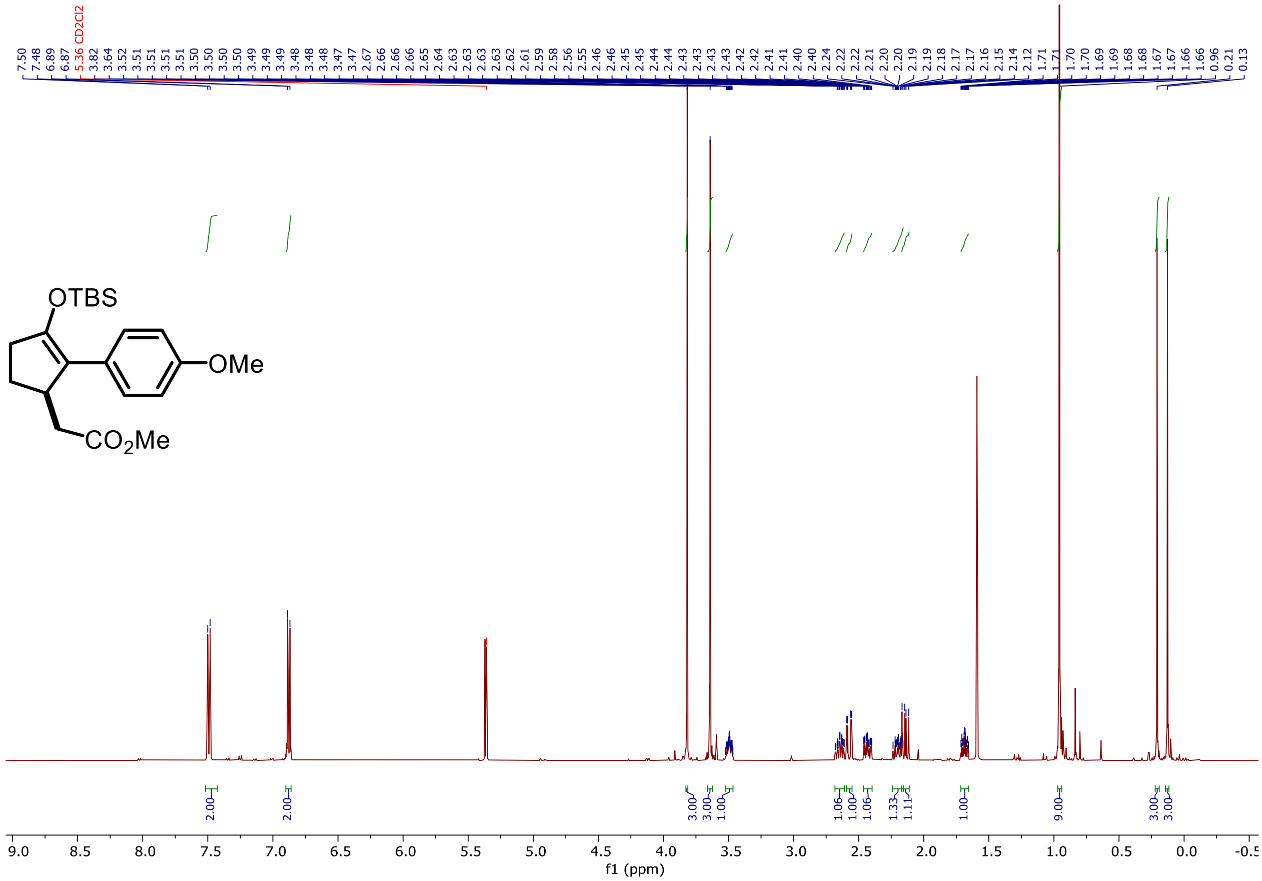
OTBS
OMe
CO2Me
f1 (ppm)

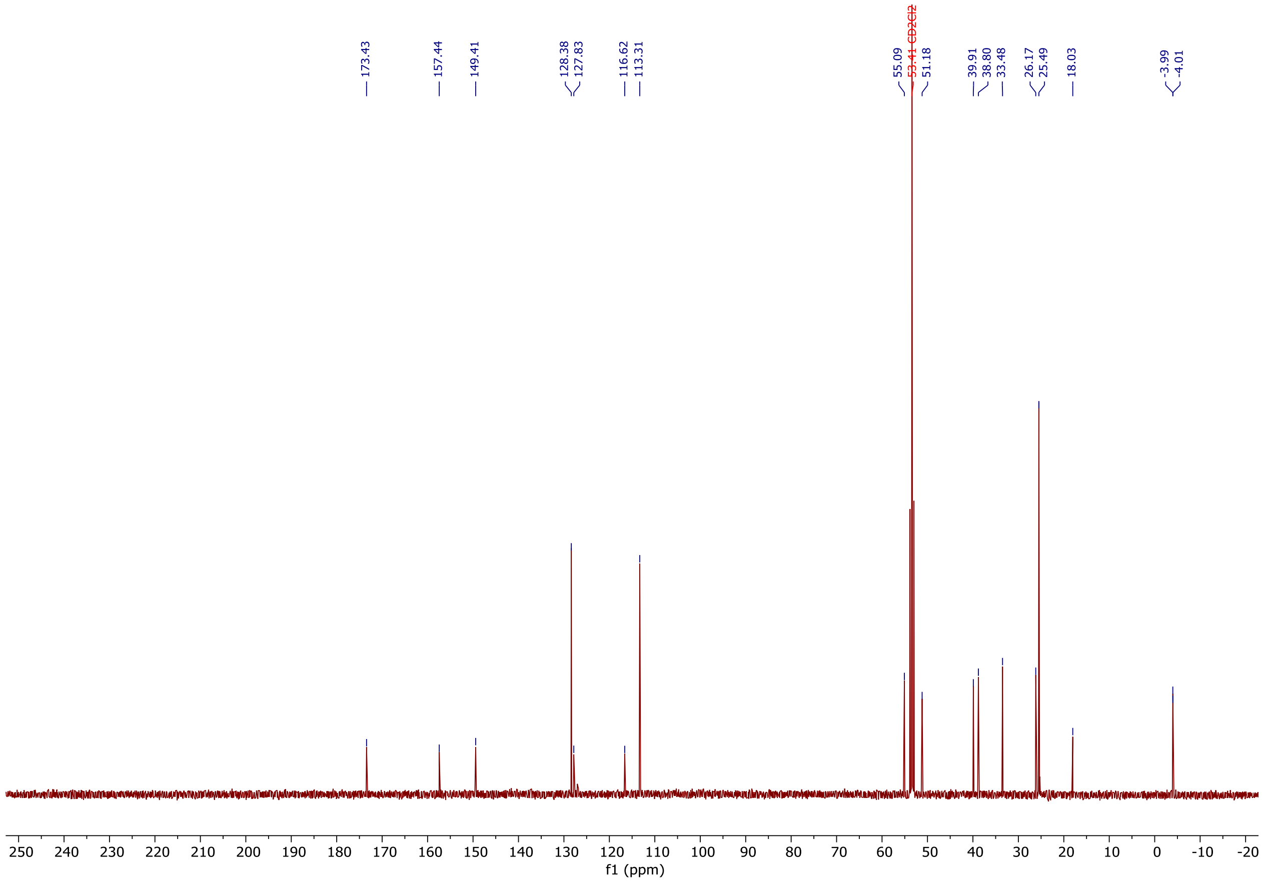
f1 (ppm)

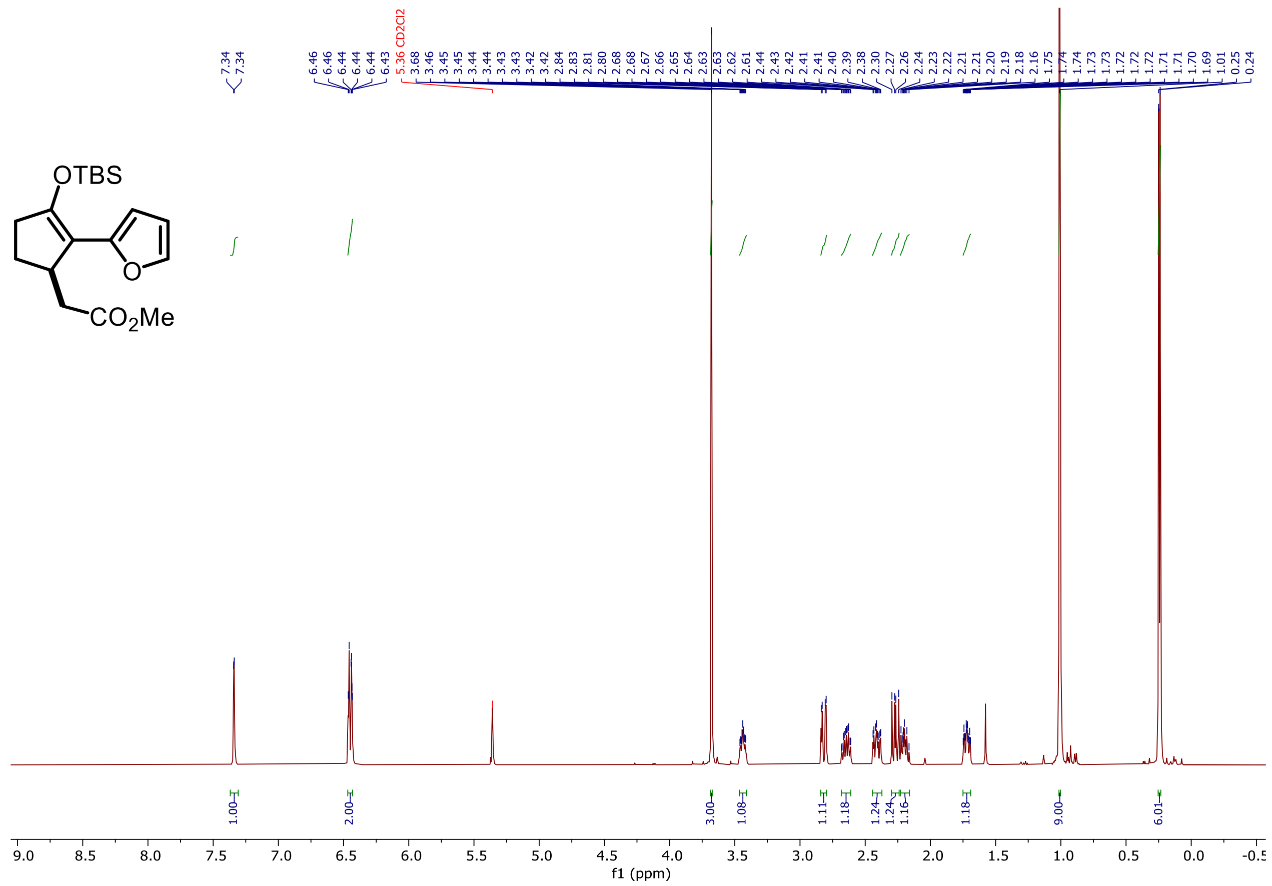

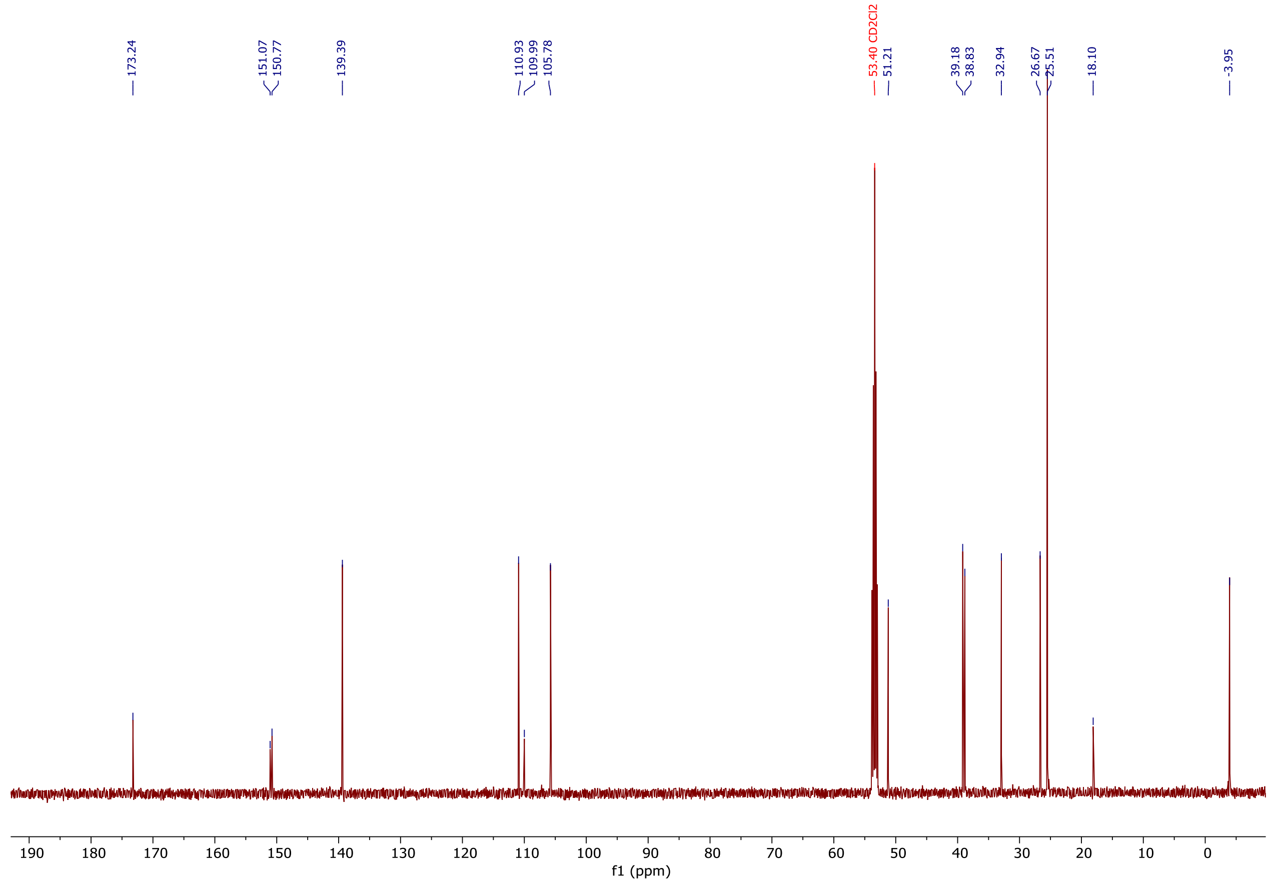

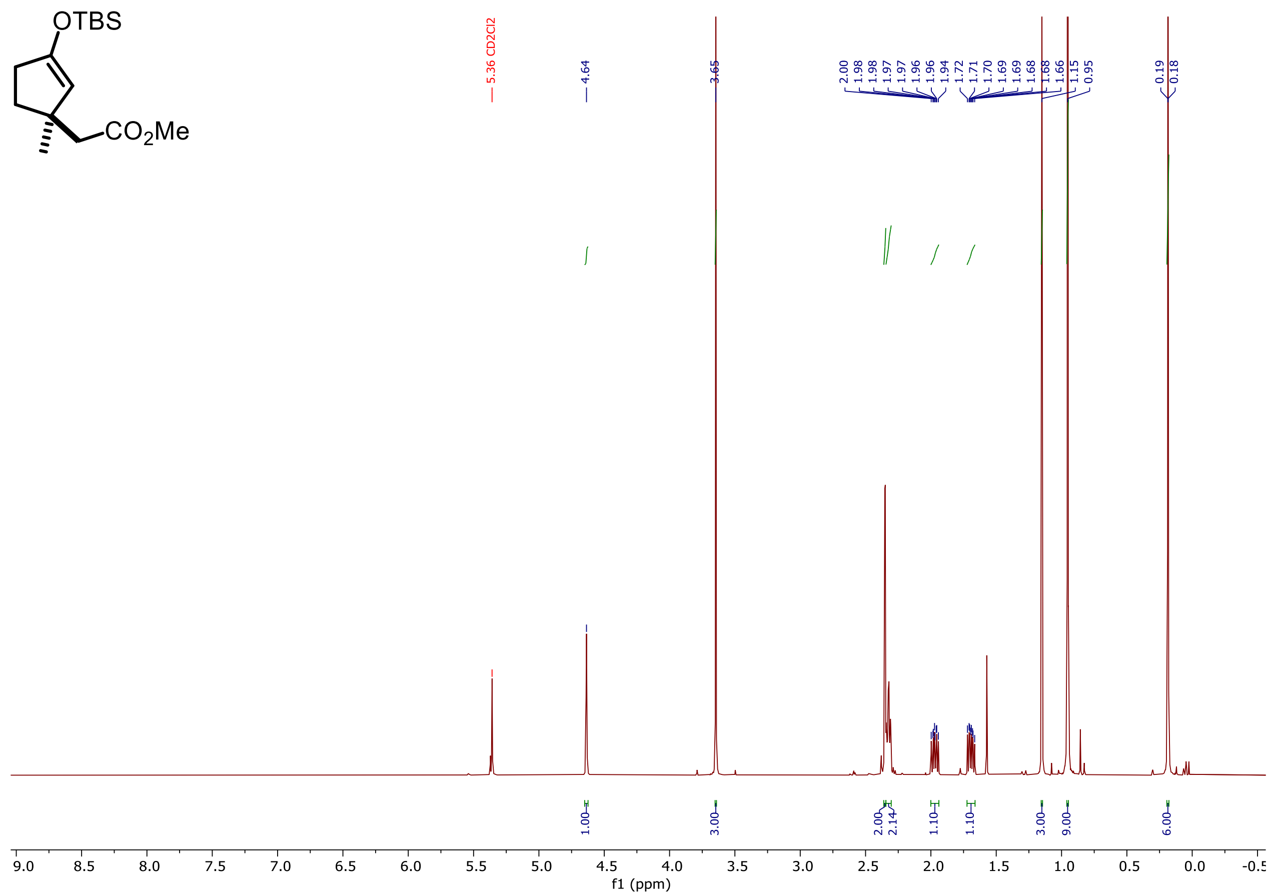
OTBS
CO2Me
5.36 CD2Cl2
4.64
3.65
2.00
1.98
1.98
1.97
1.97
1.96
1.96
1.94
1.72
1.71
1.70
1.69
1.69
1.68
1.68
1.66
1.15
0.95
0.19
0.18
1.00
3.00
2.00
2.14
1.10
1.10
3.00
9.00
6.00
f1 (ppm)

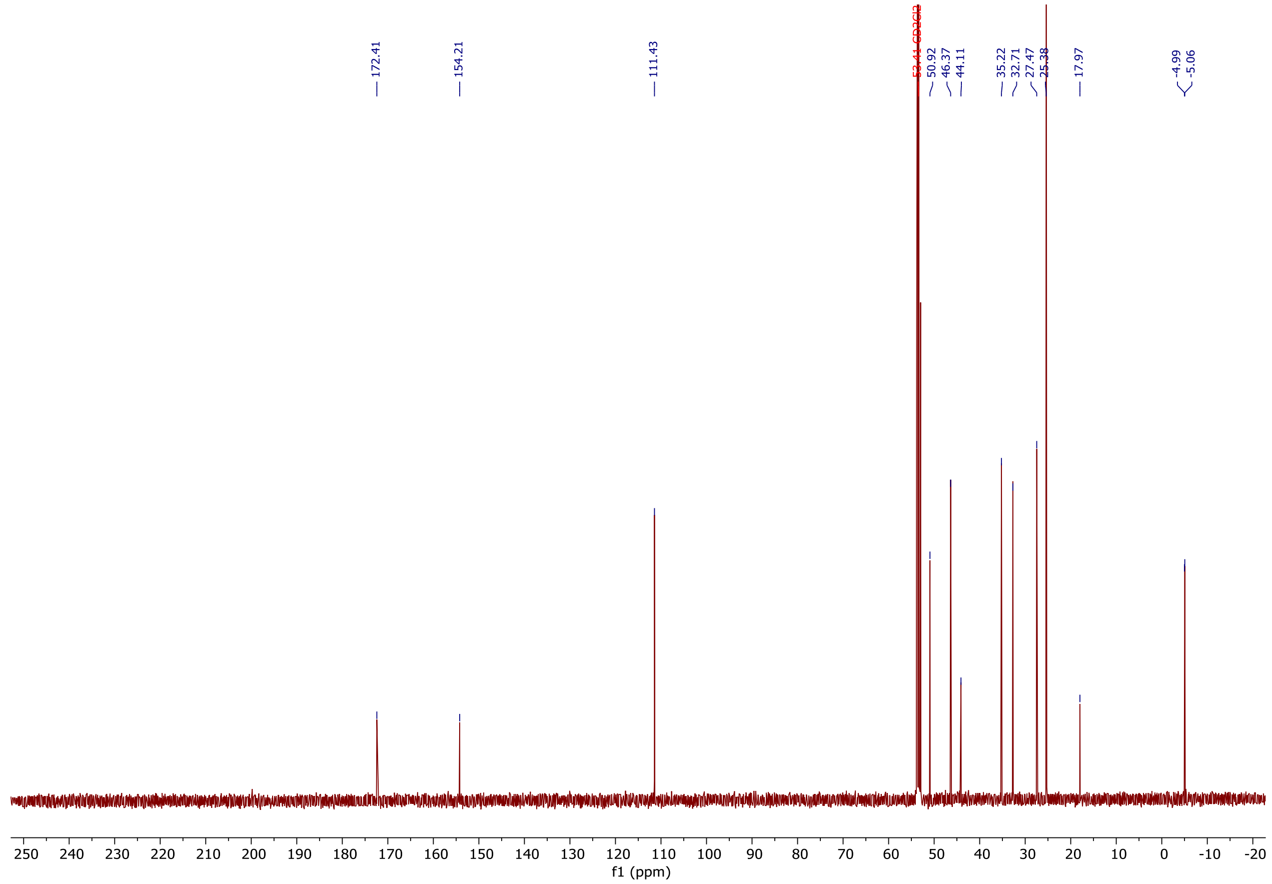
172.41
154.21
111.43
53.41 CD2Cl2
50.92
46.37
44.11
35.22
32.71
27.47
25.38
17.97
-4.99
-5.06
f1 (ppm)

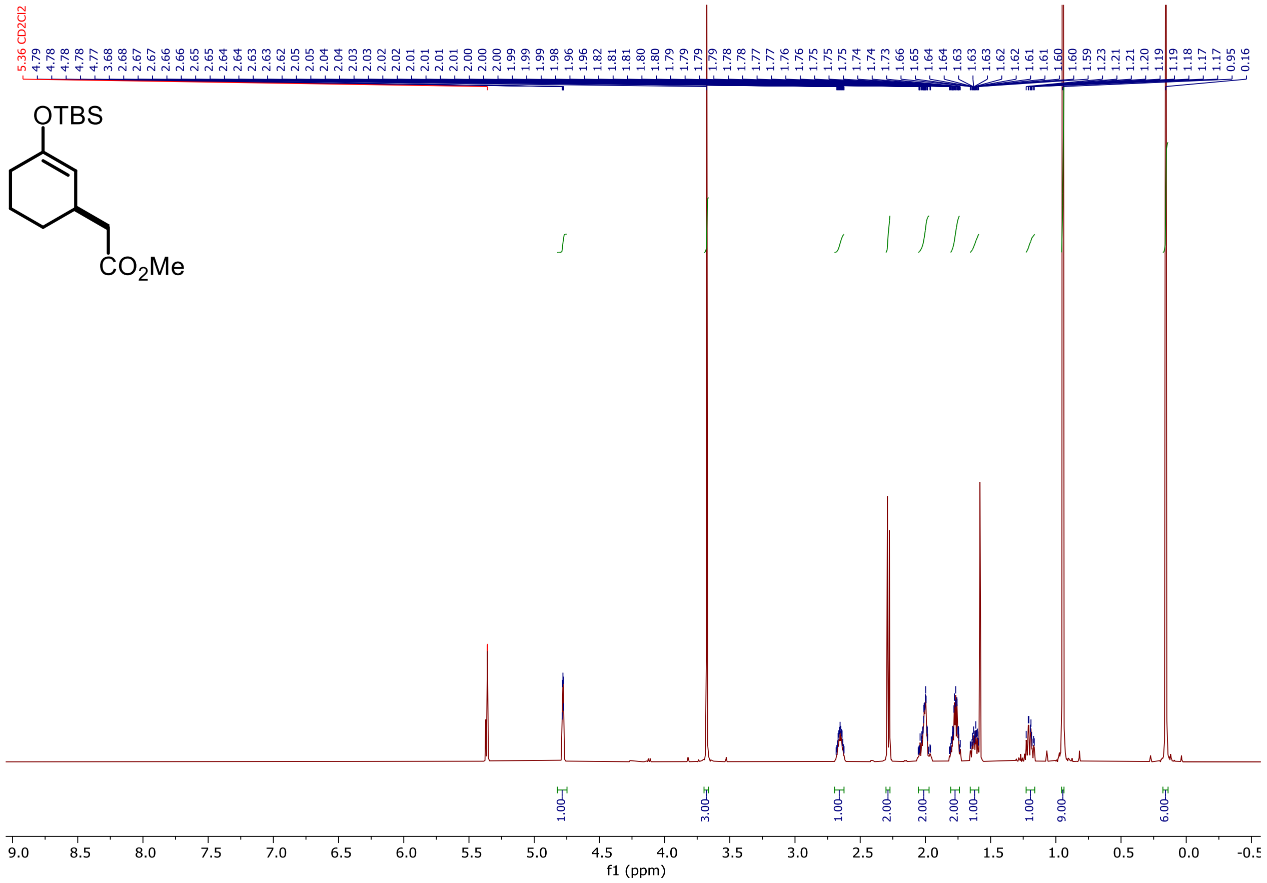

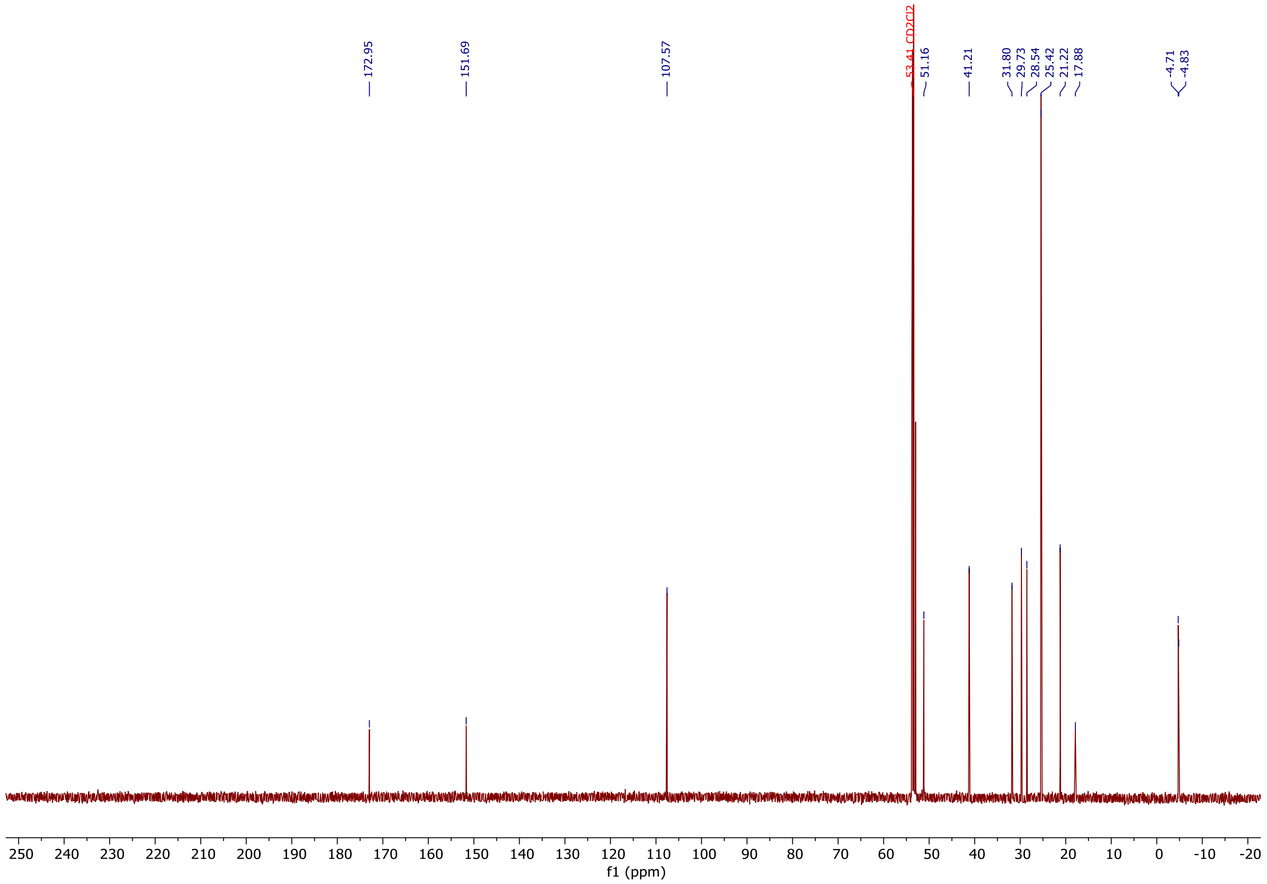

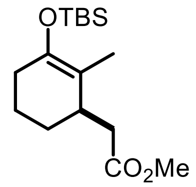
OTBS
CO2Me

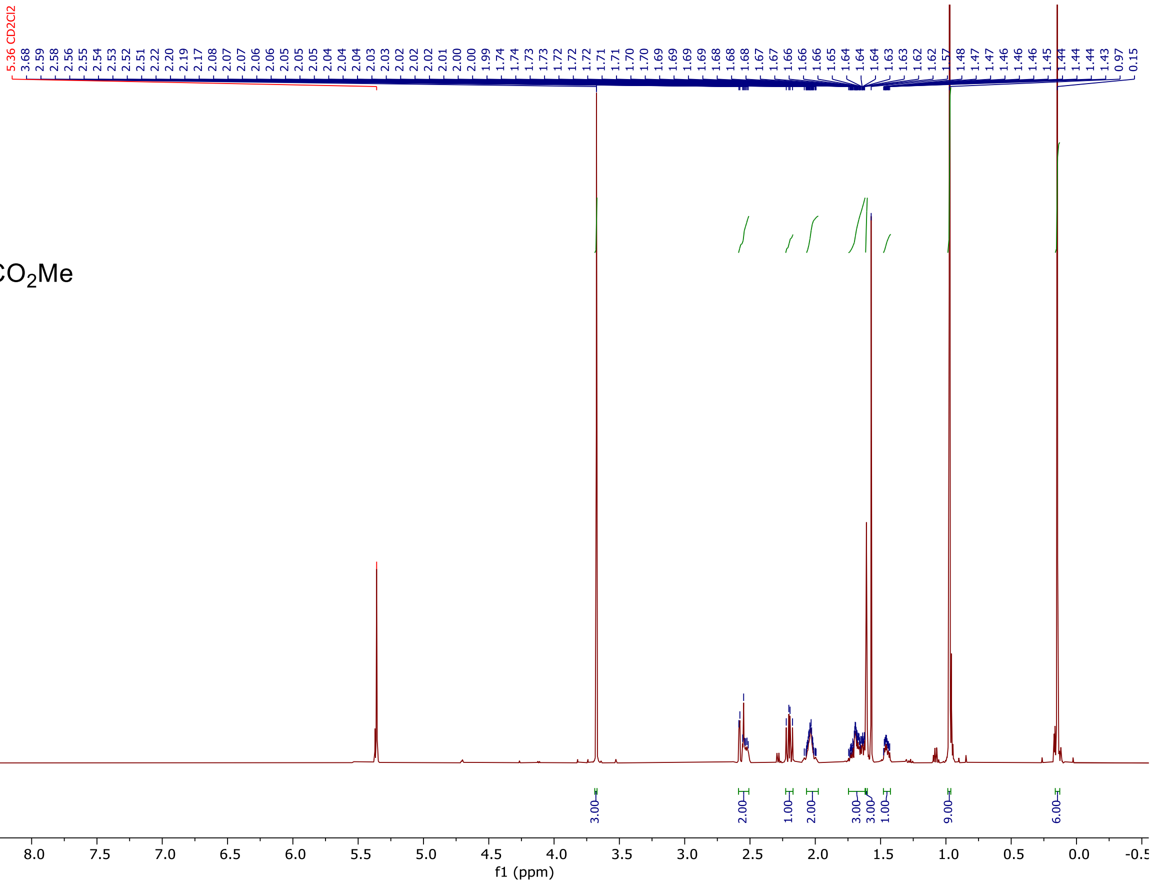
5.36 CD2Cl2
3.68
2.59
2.58
2.56
2.55
2.54
2.53
2.52
2.51
2.22
2.20
2.19
2.17
2.08
2.07
2.07
2.06
2.06
2.05
2.05
2.05
2.04
2.04
2.04
2.03
2.03
2.02
2.02
2.02
2.02
2.01
2.00
2.00
1.99
1.74
1.74
1.73
1.73
1.72
1.72
1.72
1.71
1.71
1.70
1.70
1.69
1.69
1.69
1.69
1.68
1.68
1.68
1.67
1.67
1.66
1.66
1.66
1.65
1.64
1.64
1.64
1.63
1.63
1.62
1.57
1.48
1.47
1.47
1.46
1.46
1.46
1.45
1.44
1.44
1.44
1.43
0.97
0.15
3.00
2.00
1.00
2.00
3.00
3.00
1.00
9.00
6.00
9.0
8.5
8.0
7.5
7.0
6.5
6.0
5.5
5.0
4.5
4.0
3.5
3.0
2.5
2.0
1.5
1.0
0.5
0.0
-0.5
f1 (ppm)

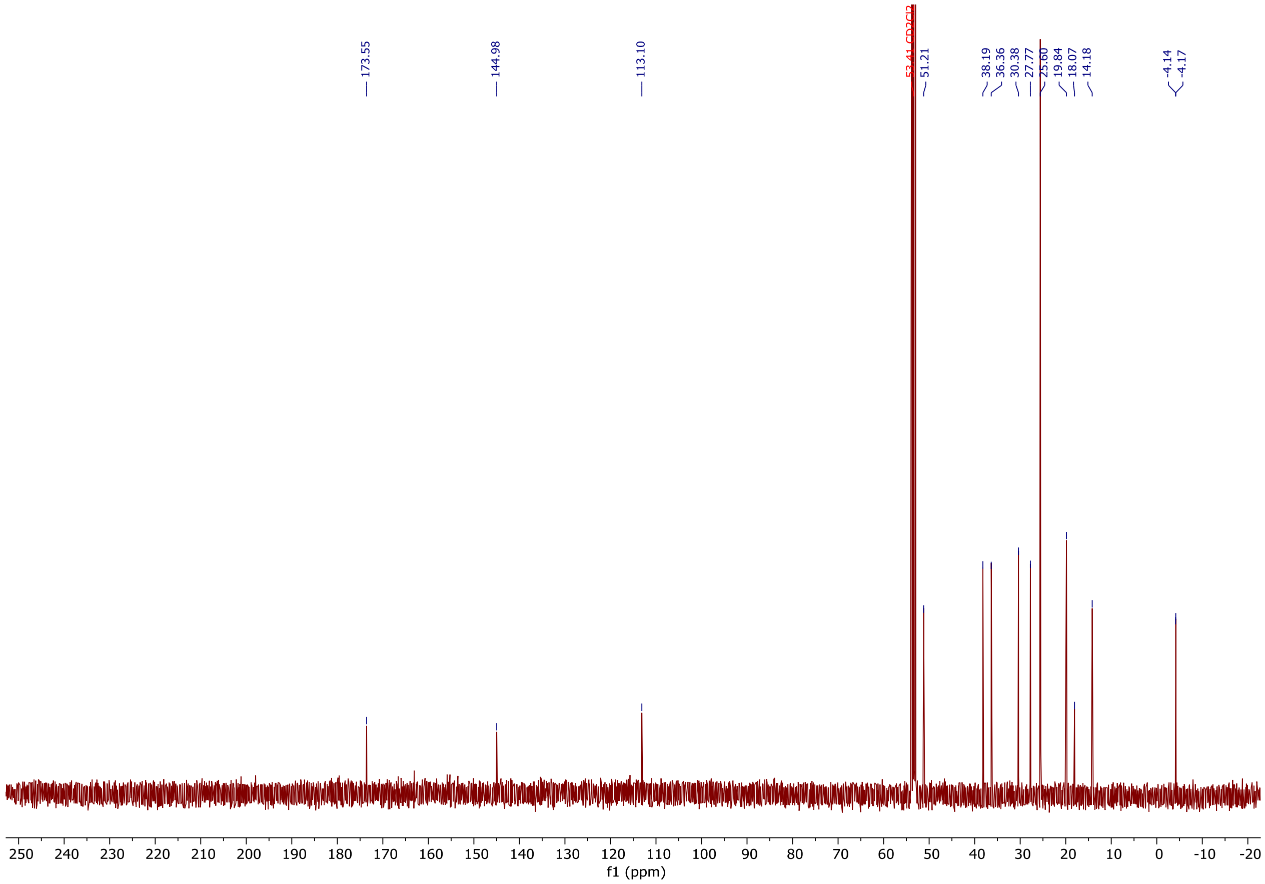
173.55
144.98
113.10
53.41 CD2Cl2
51.21
38.19
36.36
30.38
27.77
25.60
19.84
18.07
14.18
-4.14
-4.17
250
240
230
220
210
200
190
180
170
160
150
140
130
120
110
100
90
80
70
60
50
40
30
20
10
0
-10
-20
f1 (ppm)

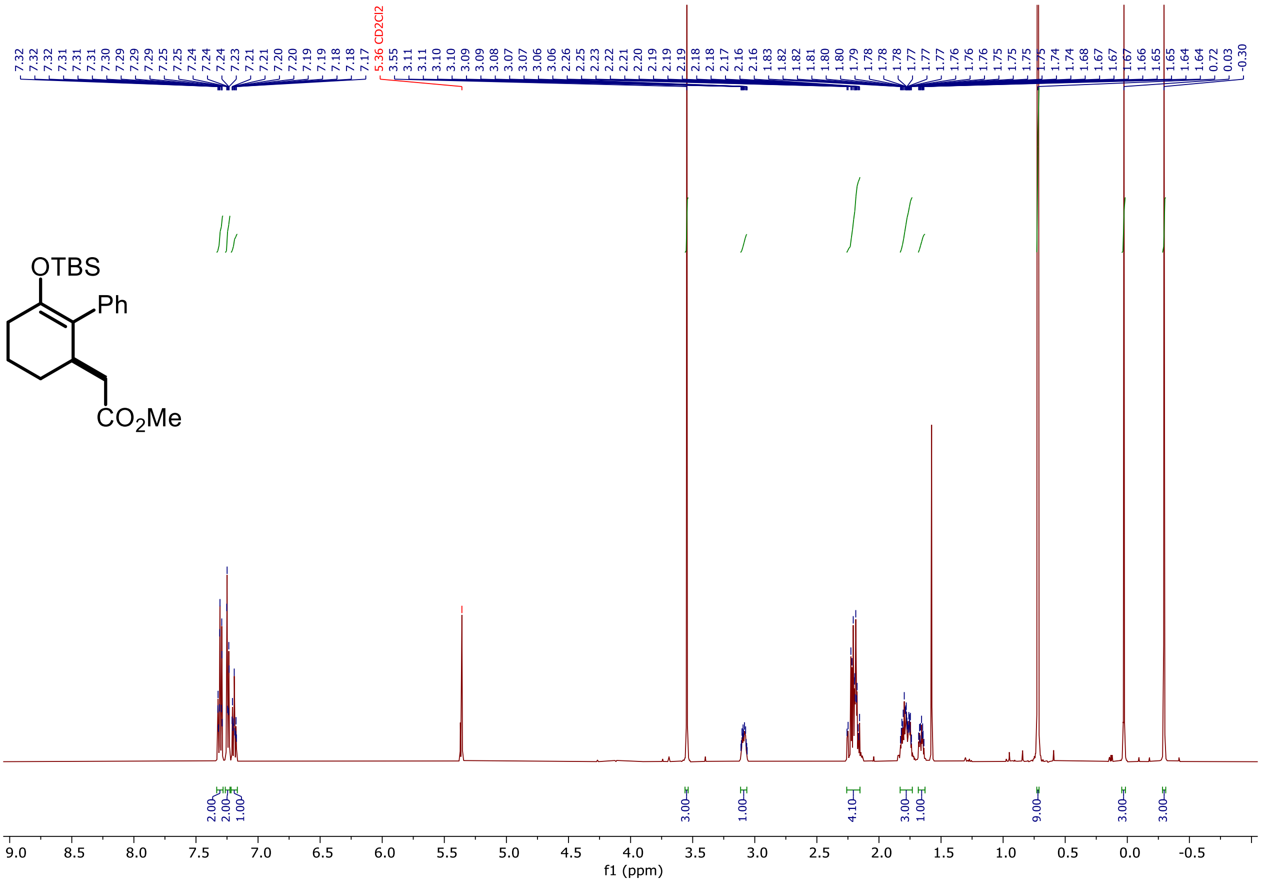
OTBS
Ph
CO_2Me
7.32 7.32 7.31 7.31 7.31 7.30 7.29 7.29 7.29 7.25 7.25 7.24 7.24 7.23 7.21 7.21 7.20 7.19 7.19 7.18 7.17
5.36 CD2Cl2
3.55 3.11 3.11 3.10 3.10 3.09 3.09 3.08 3.07 3.07 3.06 3.06 2.26 2.25 2.23 2.22 2.21 2.20 2.19 2.19 2.19 2.18 2.18 2.17 2.16 2.16 1.83 1.82 1.82 1.81 1.80 1.80 1.79 1.78 1.78 1.78 1.77 1.77 1.77 1.76 1.76 1.75 1.75 1.75 1.75 1.74 1.74 1.68 1.67 1.67 1.67 1.66 1.65 1.65 1.64 1.64 0.72 0.03 -0.30
2.00 2.00 1.00 3.00 1.00 4.10 3.00 1.00 9.00 3.00 3.00
9.0 8.5 8.0 7.5 7.0 6.5 6.0 5.5 5.0 4.5 4.0 3.5 3.0 2.5 2.0 1.5 1.0 0.5 0.0 -0.5
f1 (ppm)

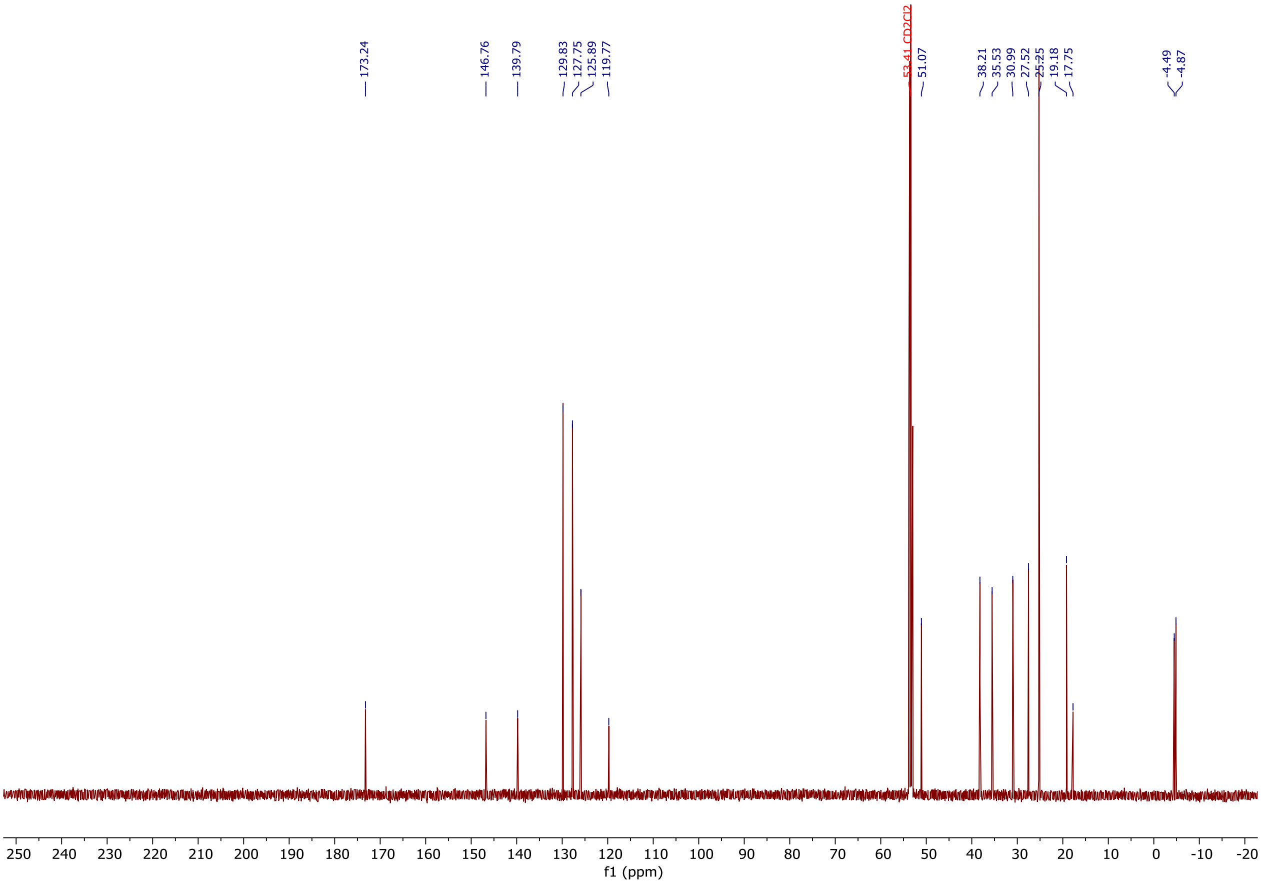
173.24
146.76
139.79
129.83 127.75 125.89 119.77
53.41 CD2Cl2
51.07
38.21 35.53 30.99 27.52 25.25 19.18 17.75
-4.49 -4.87
250 240 230 220 210 200 190 180 170 160 150 140 130 120 110 100 90 80 70 60 50 40 30 20 10 0 -10 -20
f1 (ppm)

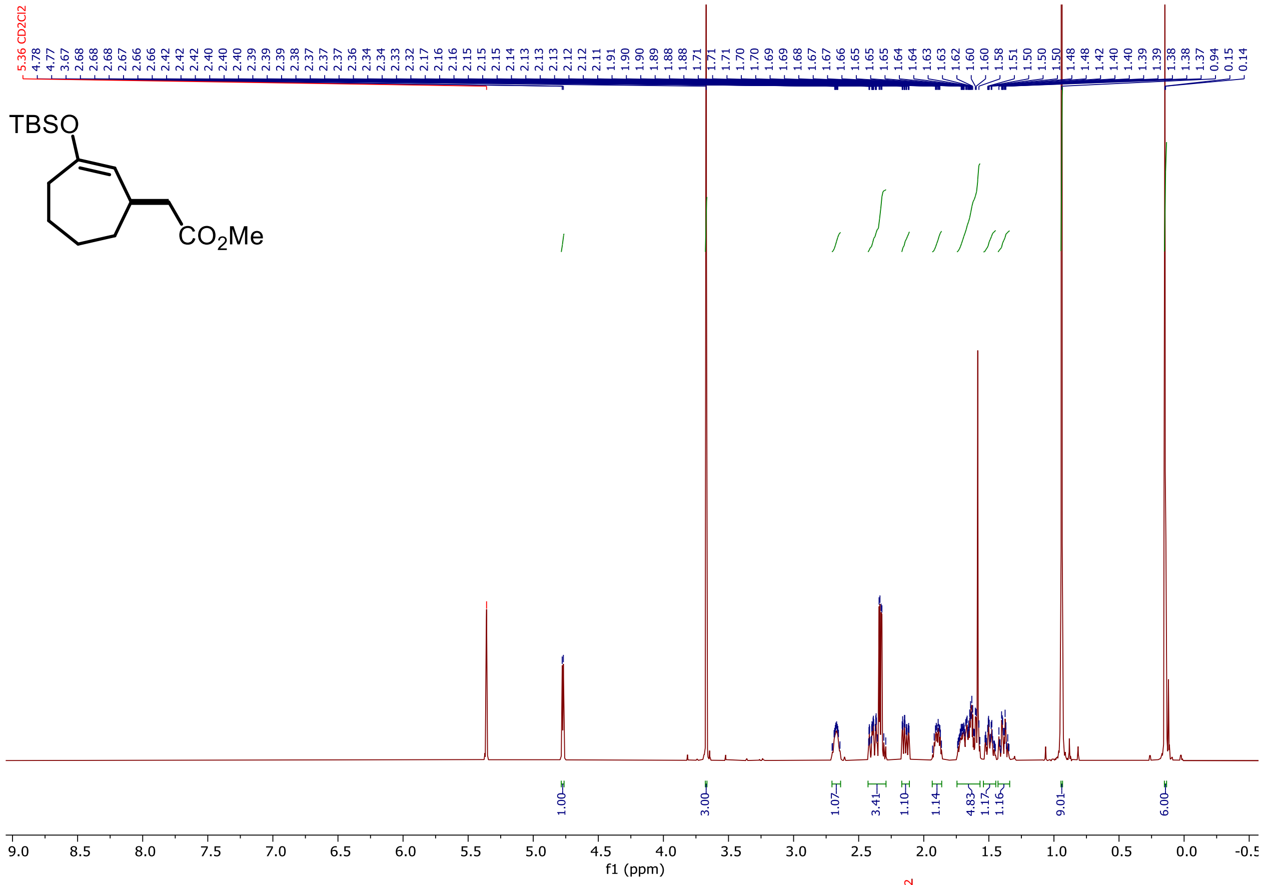
TBSO
CO2Me
1.00
3.00
1.07
3.41
1.10
1.14
4.83
1.17
1.16
9.01
6.00
f1 (ppm)

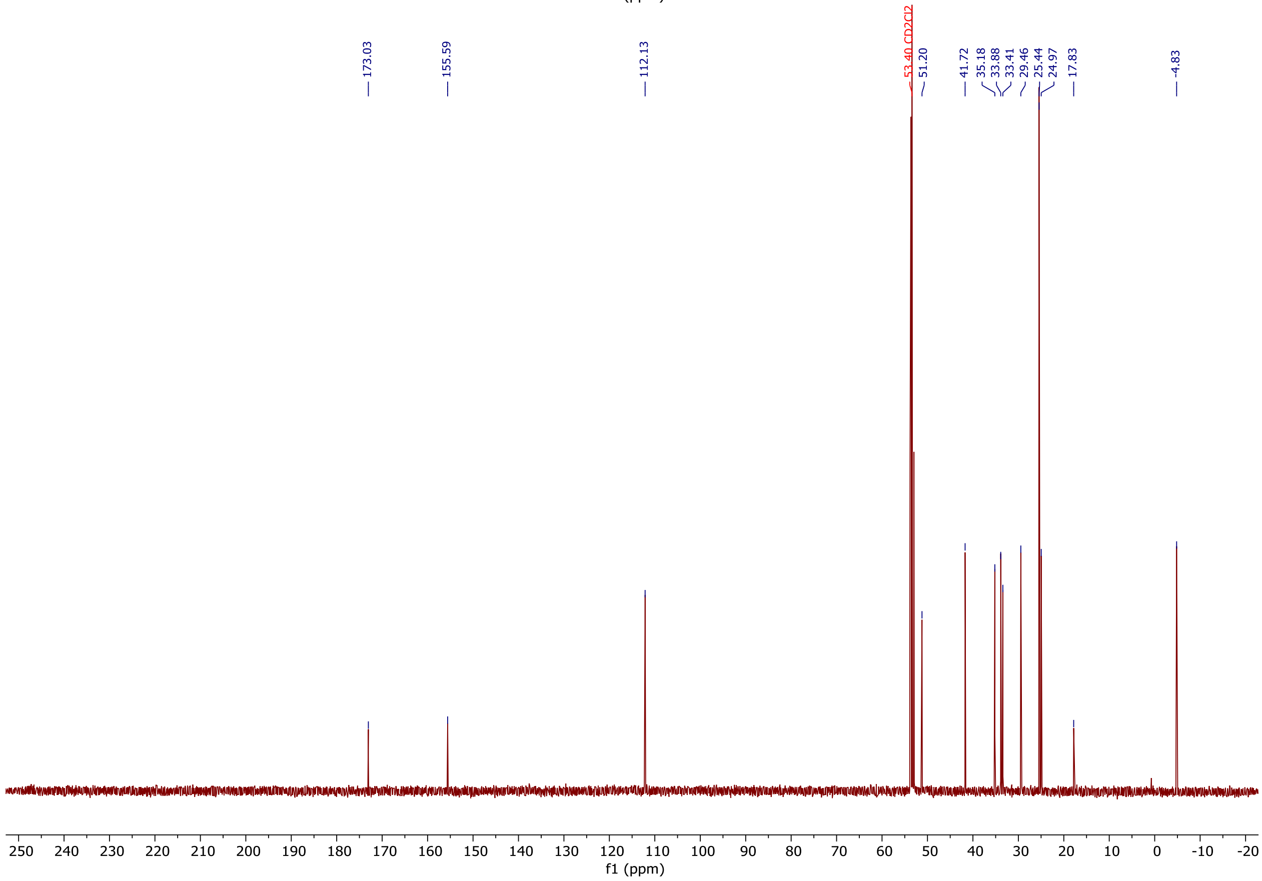
173.03
155.59
112.13
53.40 CD2Cl2
51.20
41.72
35.18
33.88
33.41
29.46
25.44
24.97
17.83
-4.83
f1 (ppm)

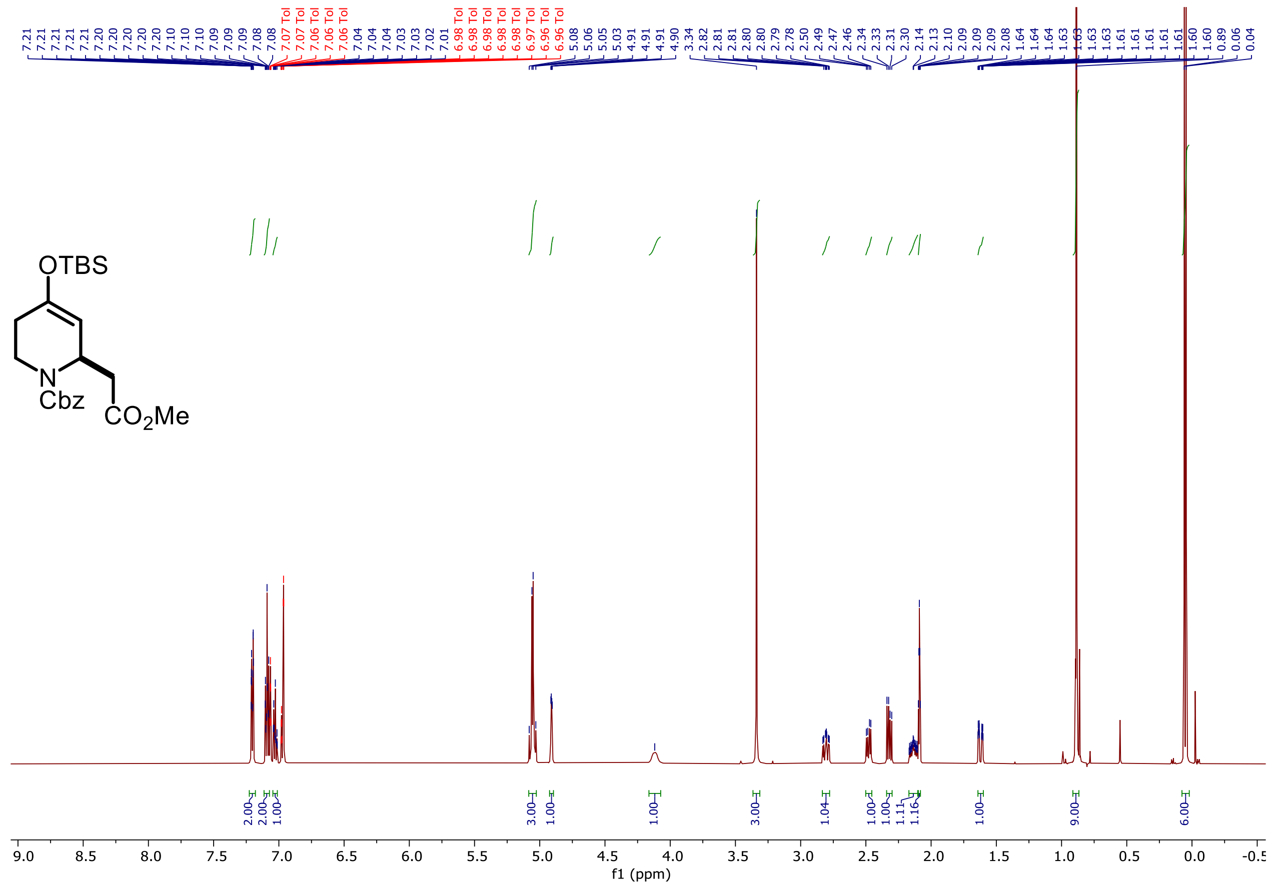

7.21
7.21
7.21
7.21
7.21
7.20
7.20
7.20
7.20
7.10
7.10
7.09
7.09
7.09
7.08
7.07 Tol
7.07 Tol
7.06 Tol
7.06 Tol
7.06 Tol
7.04
7.04
7.03
7.02
7.01
6.98 Tol
6.98 Tol
6.98 Tol
6.98 Tol
6.98 Tol
6.97 Tol
6.96 Tol
6.96 Tol
5.08
5.06
5.05
5.03
4.91
4.91
4.91
4.90
3.34
2.82
2.81
2.81
2.80
2.80
2.79
2.78
2.50
2.49
2.47
2.46
2.34
2.33
2.31
2.30
2.14
2.13
2.10
2.09
2.09
2.09
2.08
1.64
1.64
1.64
1.63
1.63
1.63
1.63
1.61
1.61
1.61
1.61
1.60
1.60
0.89
0.06
0.04
2.00
2.00
1.00
3.00
1.00
1.00
3.00
1.04
1.00
1.00
1.11
1.16
1.00
9.00
6.00
9.0 8.5 8.0 7.5 7.0 6.5 6.0 5.5 5.0 4.5 4.0 3.5 3.0 2.5 2.0 1.5 1.0 0.5 0.0 -0.5
f1 (ppm)
OTBS
N
Cbz
CO2Me

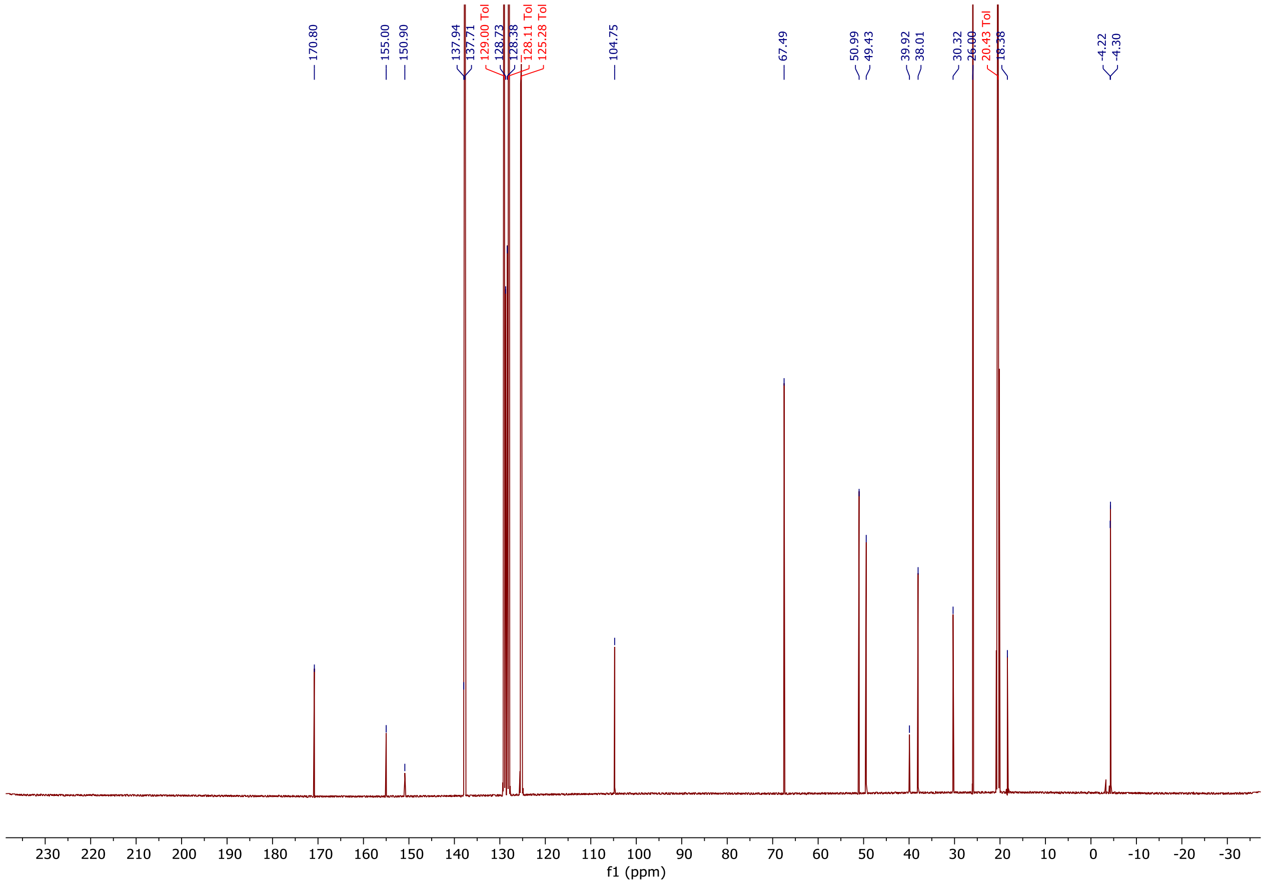

170.80
155.00
150.90
137.94
137.71
129.00 Tol
128.73
128.38
128.11 Tol
125.28 Tol
104.75
67.49
50.99
49.43
39.92
38.01
30.32
26.00
20.43 Tol
18.38
-4.22
-4.30
230 220 210 200 190 180 170 160 150 140 130 120 110 100 90 80 70 60 50 40 30 20 10 0 -10 -20 -30
f1 (ppm)

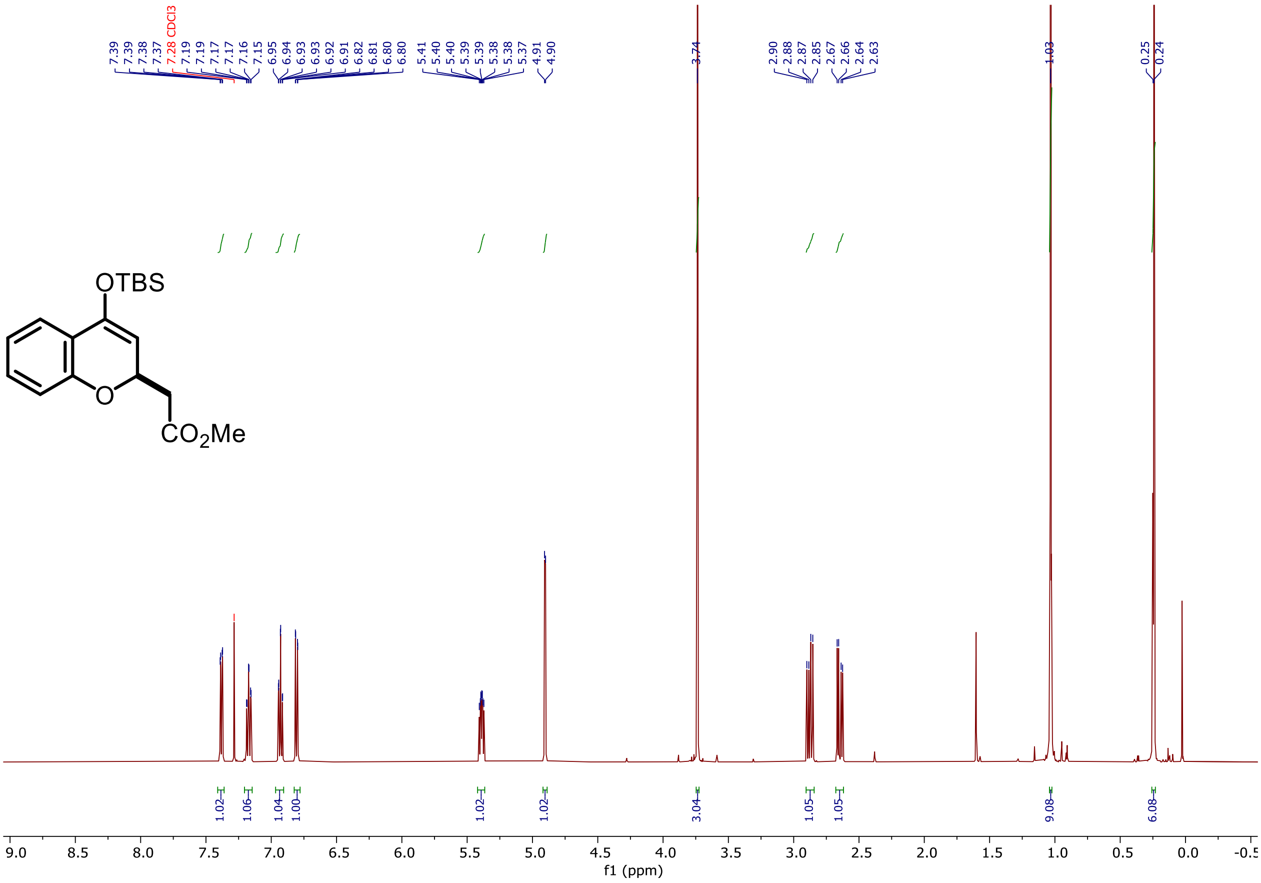

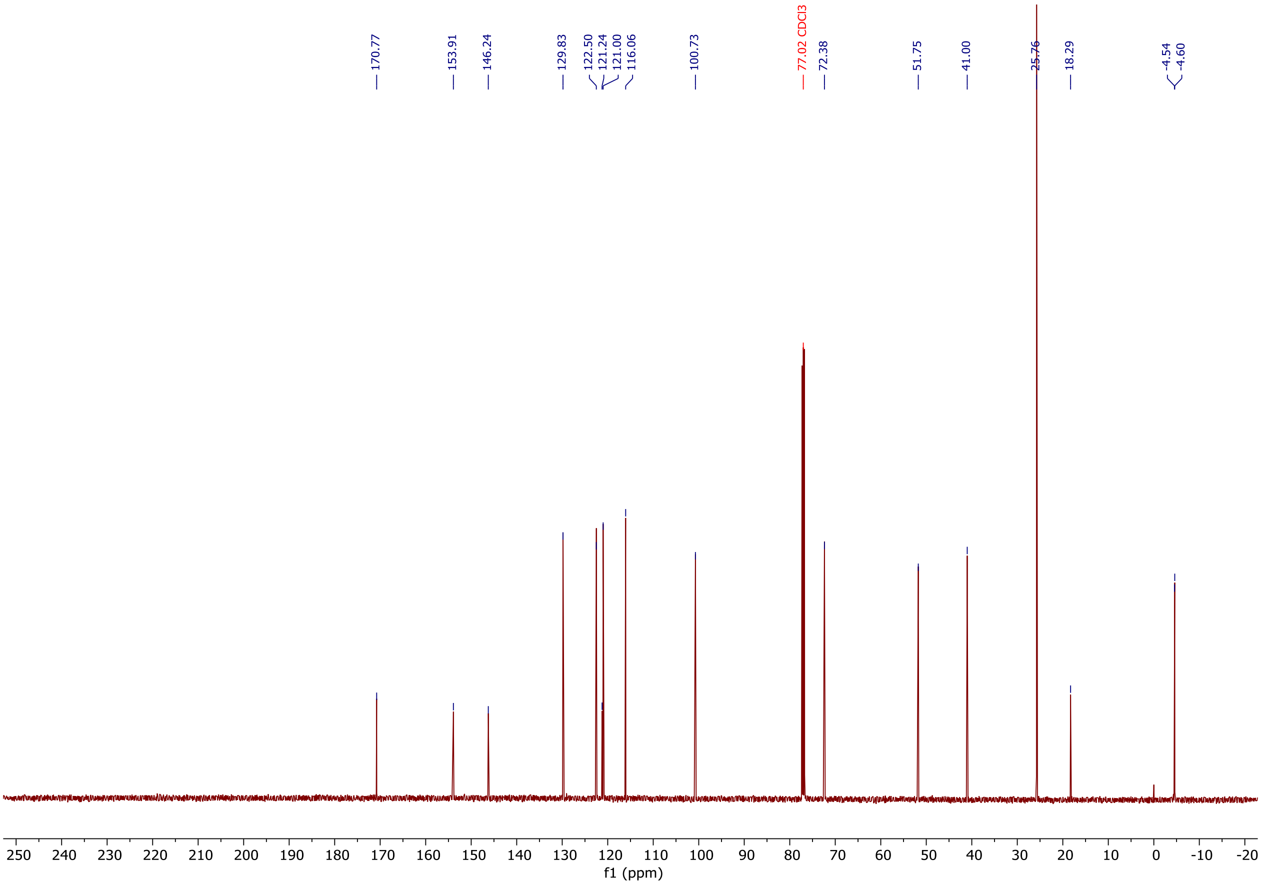

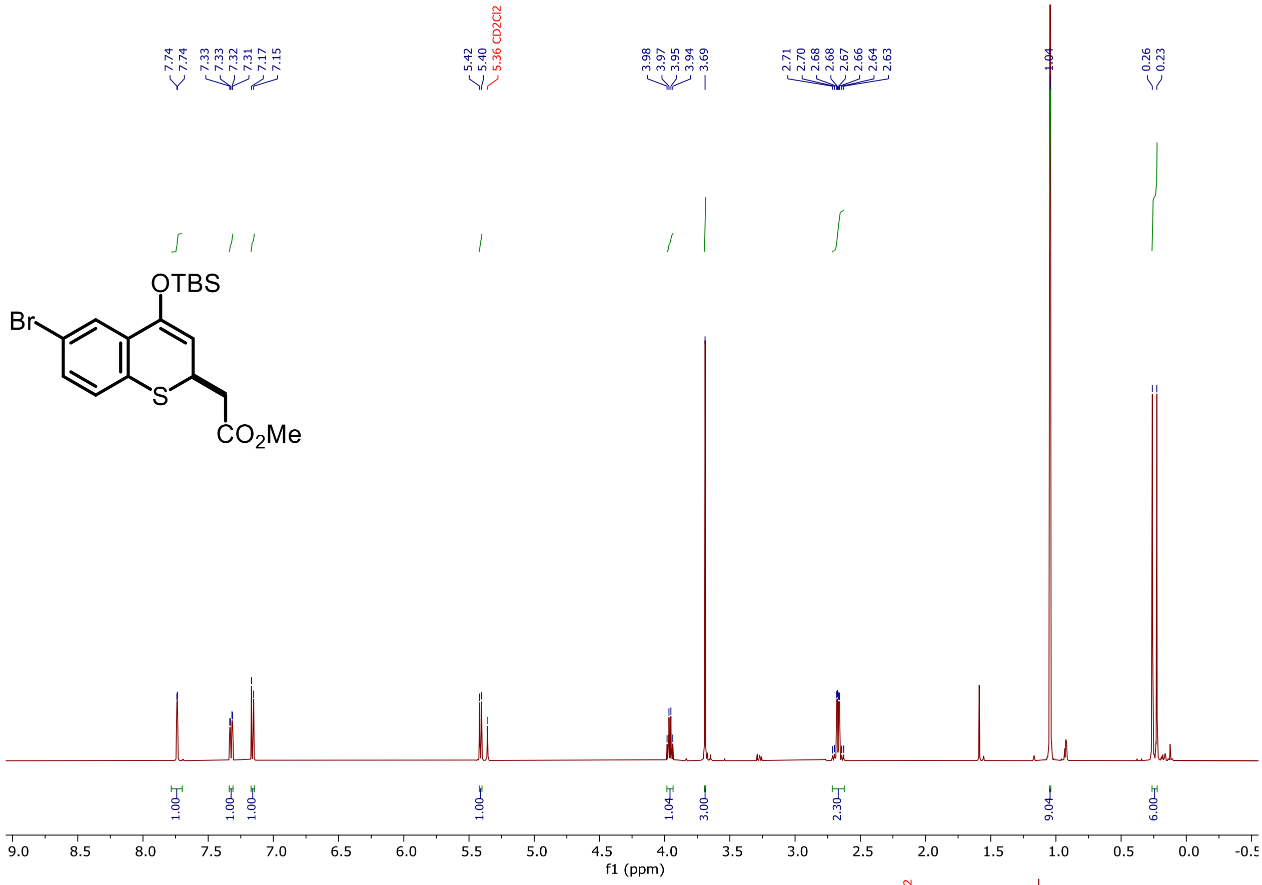
7.74
7.74
7.33
7.33
7.32
7.31
7.17
7.15
5.42
5.40
5.36 CD2Cl2
3.98
3.97
3.95
3.94
3.69
2.71
2.70
2.68
2.68
2.67
2.66
2.64
2.63
1.04
0.26
0.23
OTBS
Br
S
CO2Me
1.00
1.00
1.00
1.00
1.04
3.00
2.30
9.04
6.00
f1 (ppm)

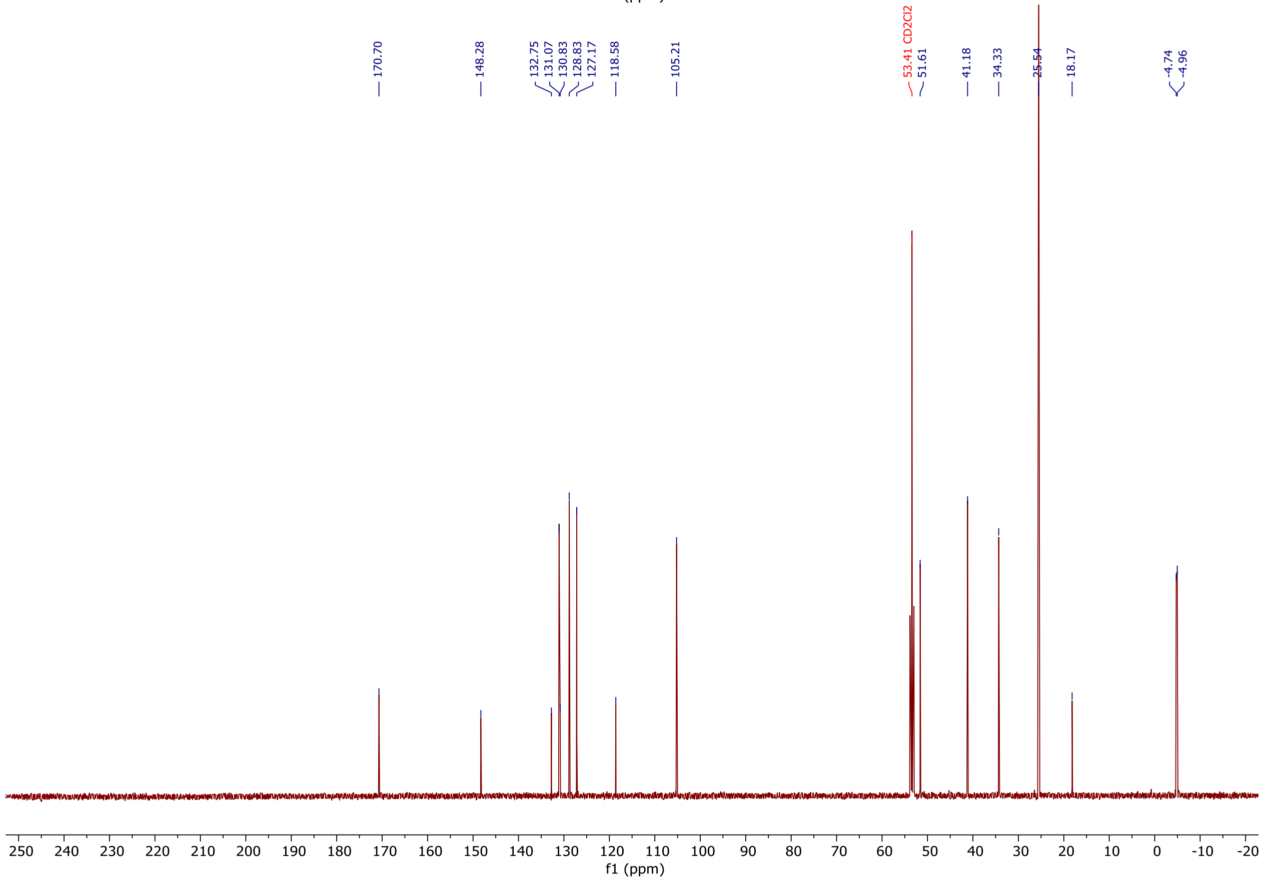
170.70
148.28
132.75
131.07
130.83
128.83
127.17
118.58
105.21
53.41 CD2Cl2
51.61
41.18
34.33
25.54
18.17
-4.74
-4.96
f1 (ppm)

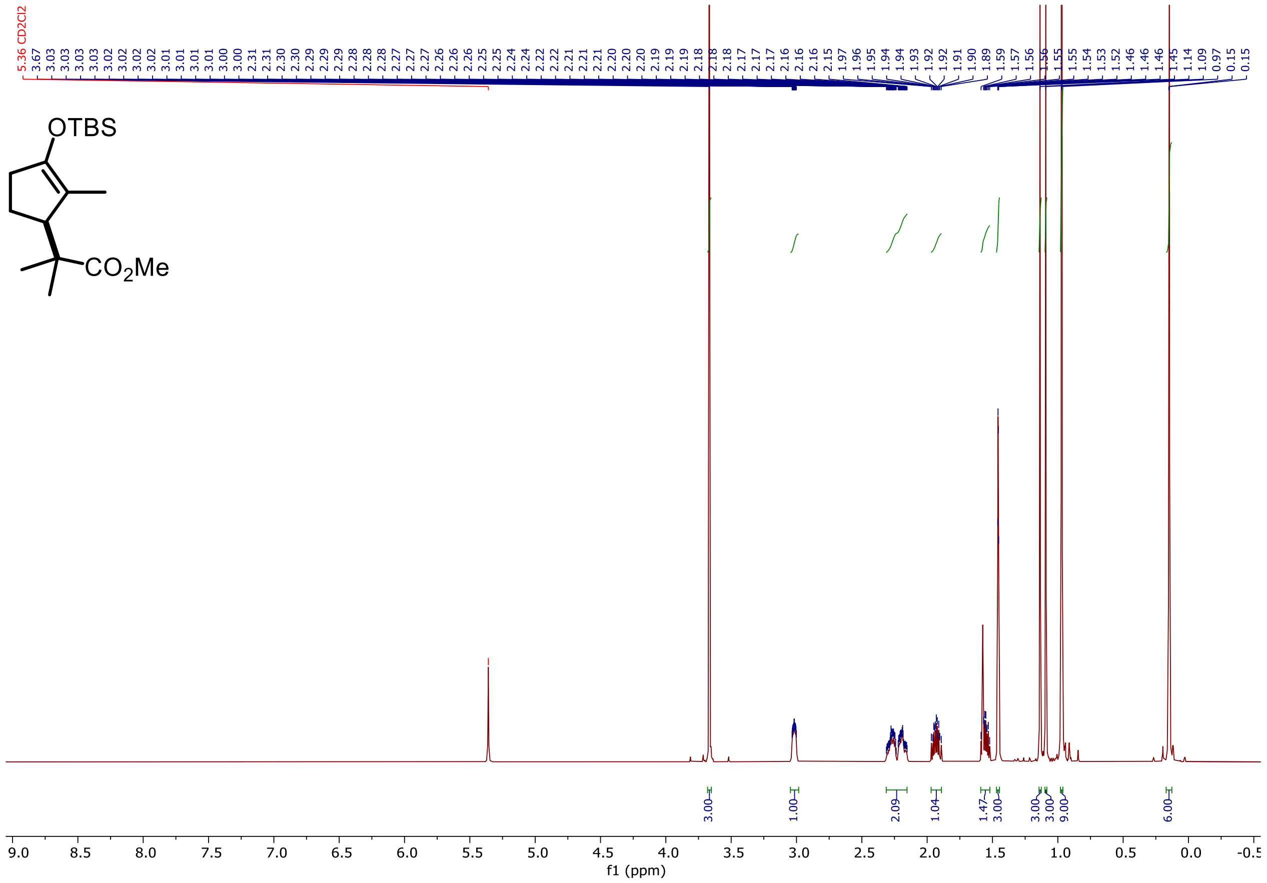
OTBS
CO_2Me
f1 (ppm)

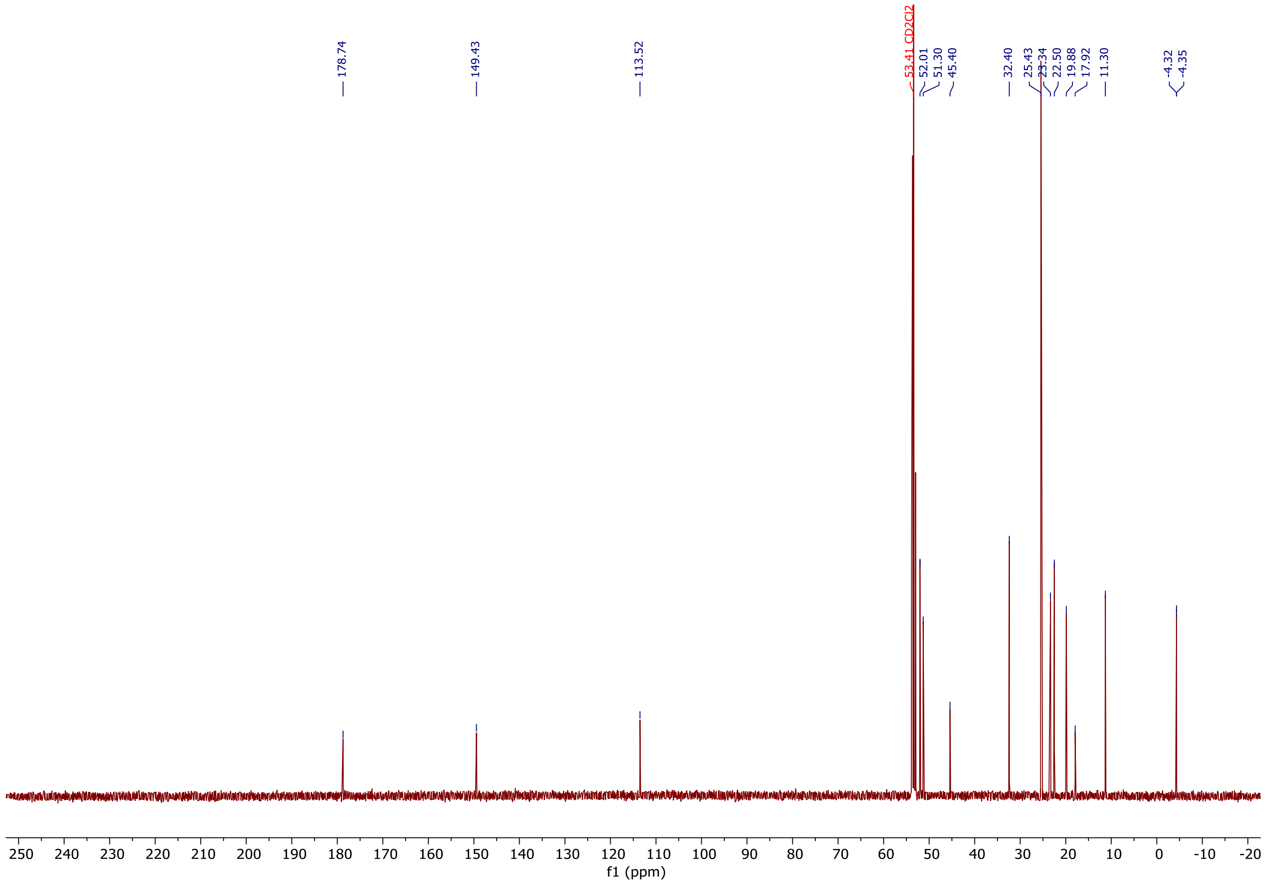
f1 (ppm)

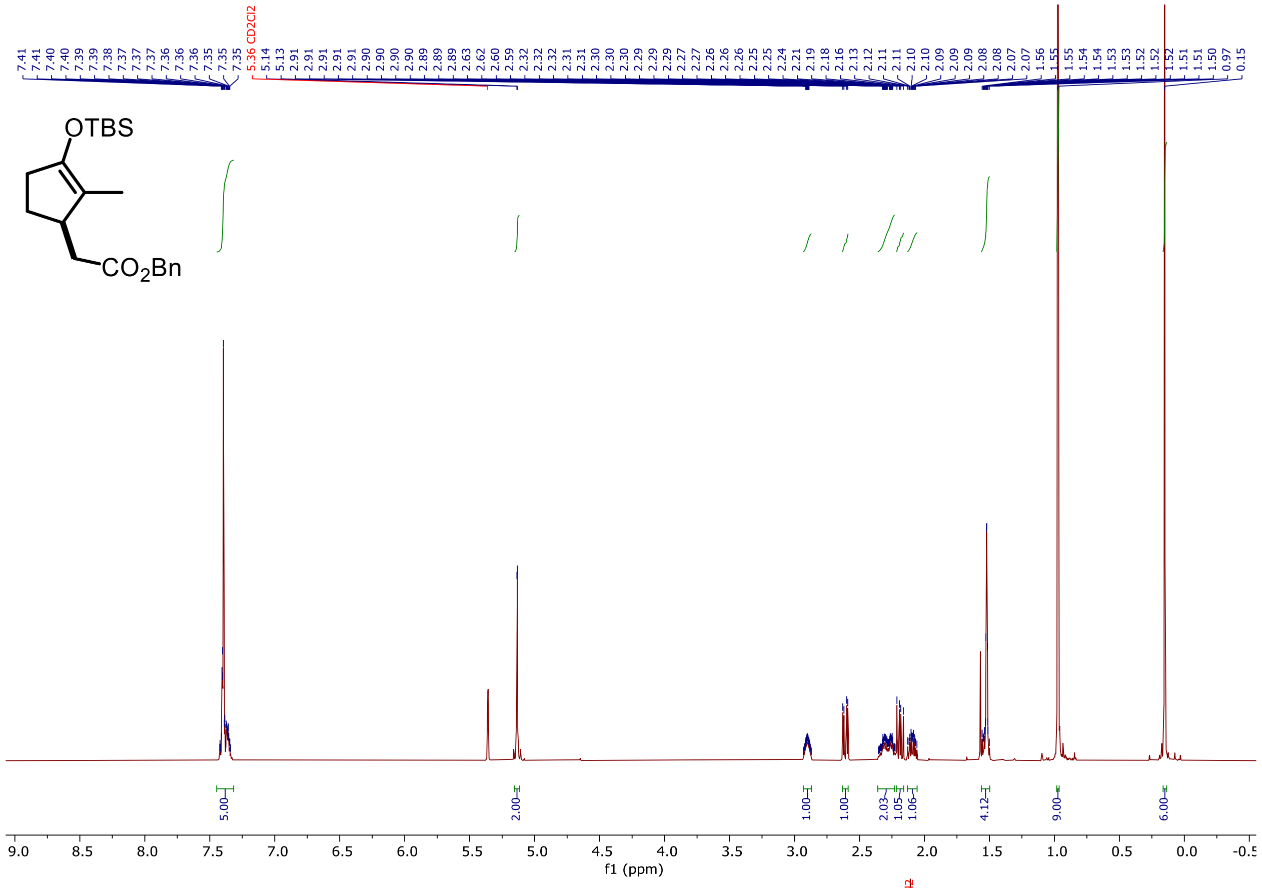
OTBS
CO2Bn
7.41 7.41 7.40 7.39 7.39 7.38 7.37 7.37 7.36 7.36 7.35 7.35 7.35
5.36 CD2Cl2
5.14 5.13
2.91 2.91 2.91 2.91 2.91 2.90 2.90 2.90 2.89 2.89 2.89 2.63 2.62 2.60 2.59
2.32 2.32 2.32 2.31 2.31 2.30 2.30 2.30 2.29 2.29 2.29 2.27 2.26 2.26 2.25 2.25 2.24 2.21 2.19 2.18 2.16 2.13 2.12 2.11 2.11 2.10 2.10 2.09 2.09 2.08 2.08 2.07 2.07
1.56 1.55 1.55 1.54 1.54 1.53 1.53 1.52 1.52 1.52 1.51 1.51 1.50 0.97 0.15
5.00 2.00 1.00 1.00 2.03 1.05 1.06 4.12 9.00 6.00
f1 (ppm)

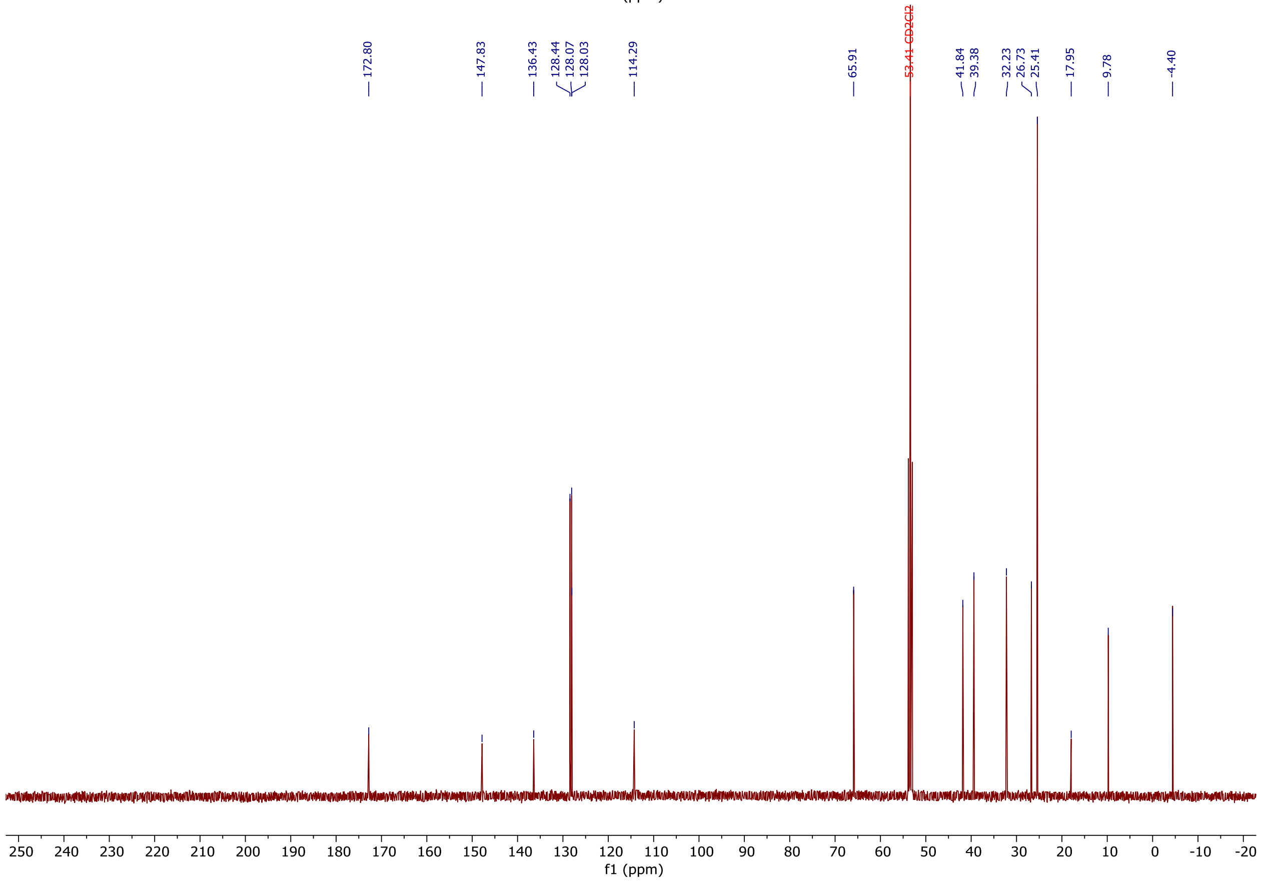
172.80
147.83
136.43
128.44
128.07
128.03
114.29
65.91
53.41 CD2Cl2
41.84
39.38
32.23
26.73
25.41
17.95
9.78
-4.40
f1 (ppm)

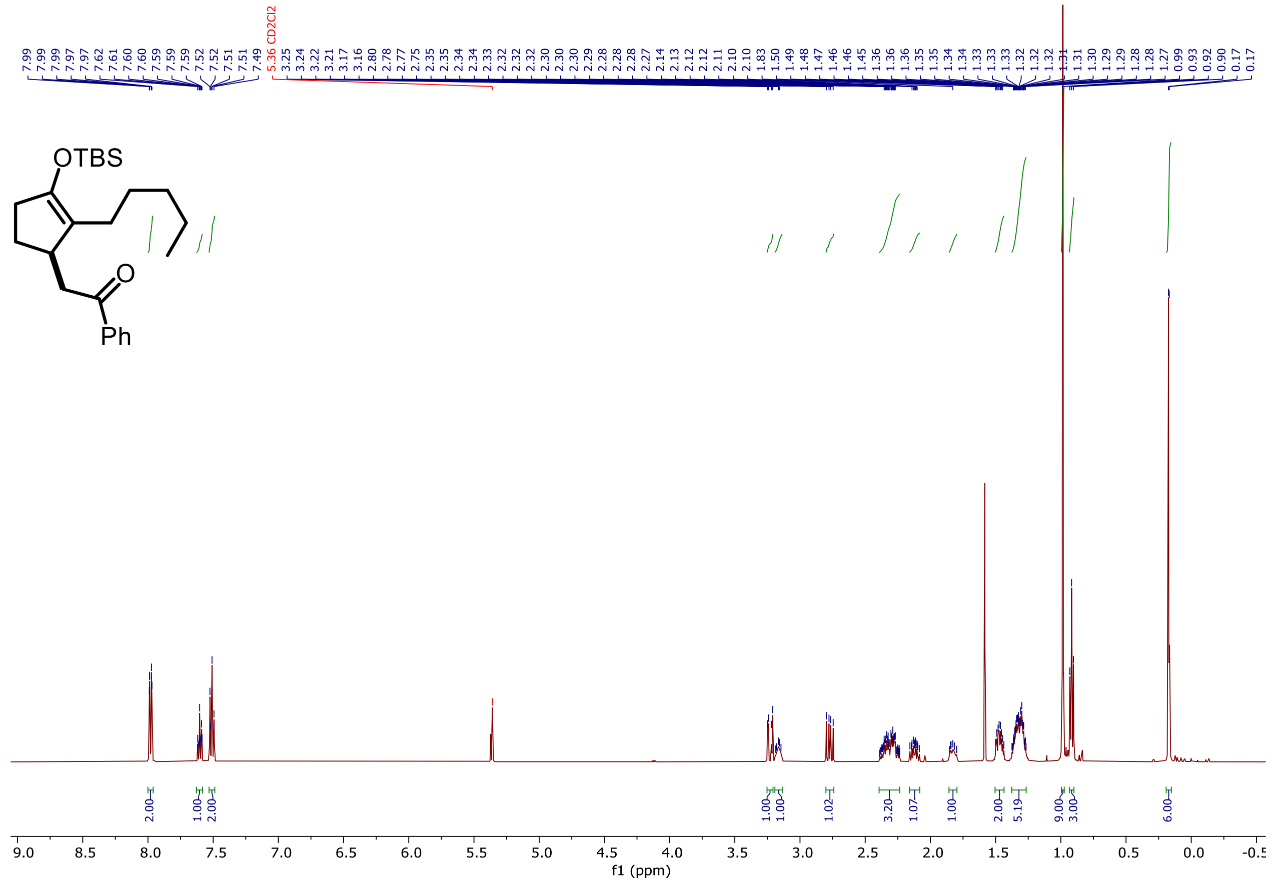
OTBS
O
Ph
5.36 CD2Cl2
f1 (ppm)

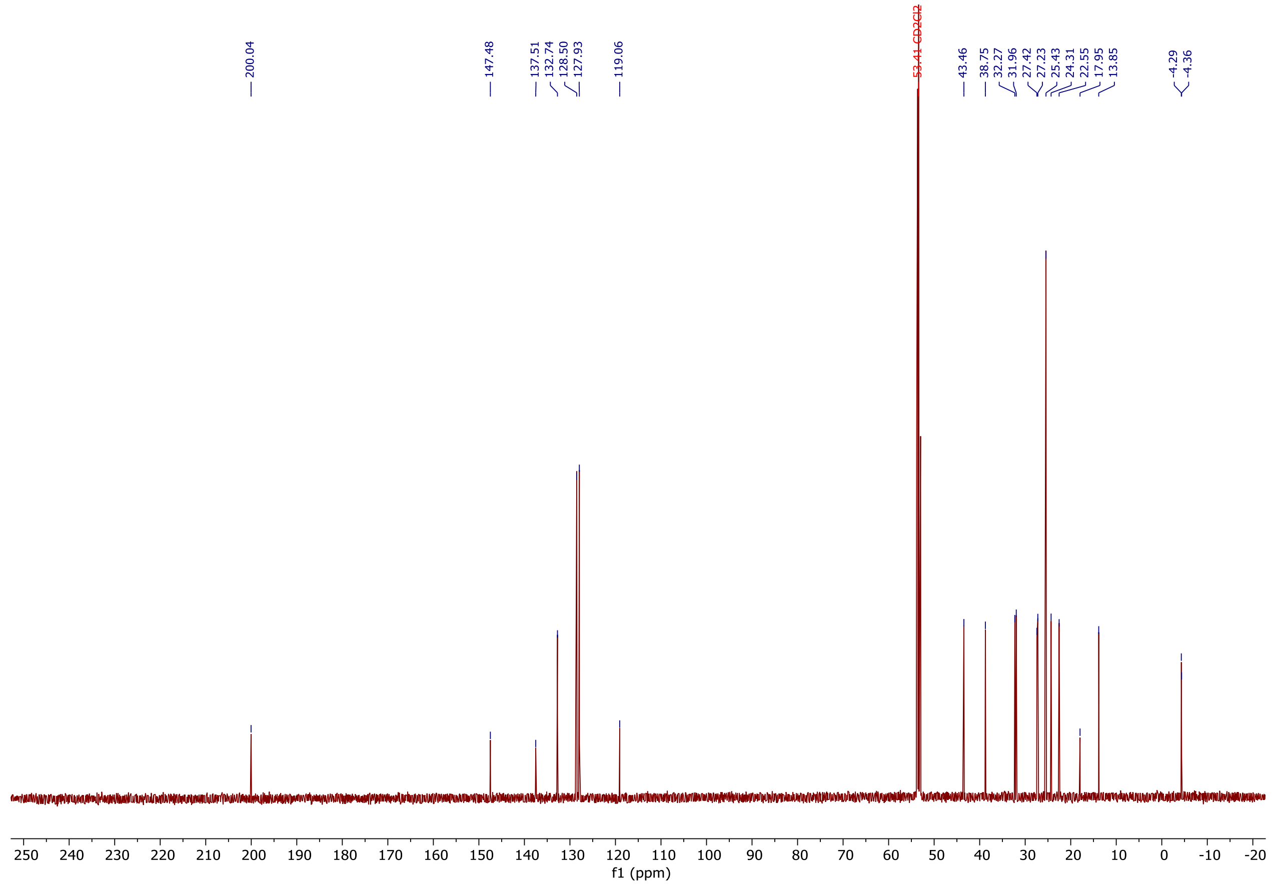
200.04
147.48
137.51
132.74
128.50
127.93
119.06
53.41 CD2Cl2
43.46
38.75
32.27
31.96
27.42
27.23
25.43
24.31
22.55
17.95
13.85
-4.29
-4.36
f1 (ppm)

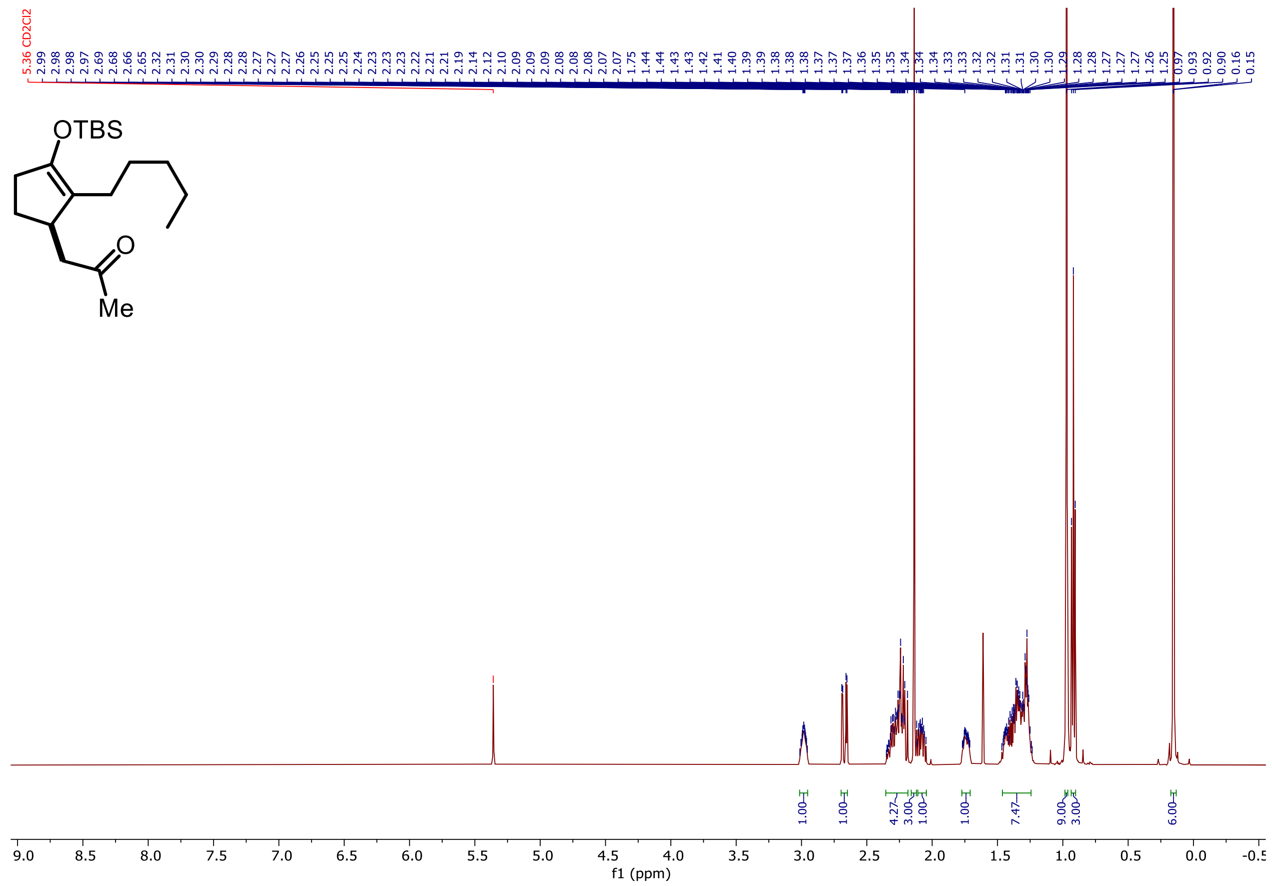

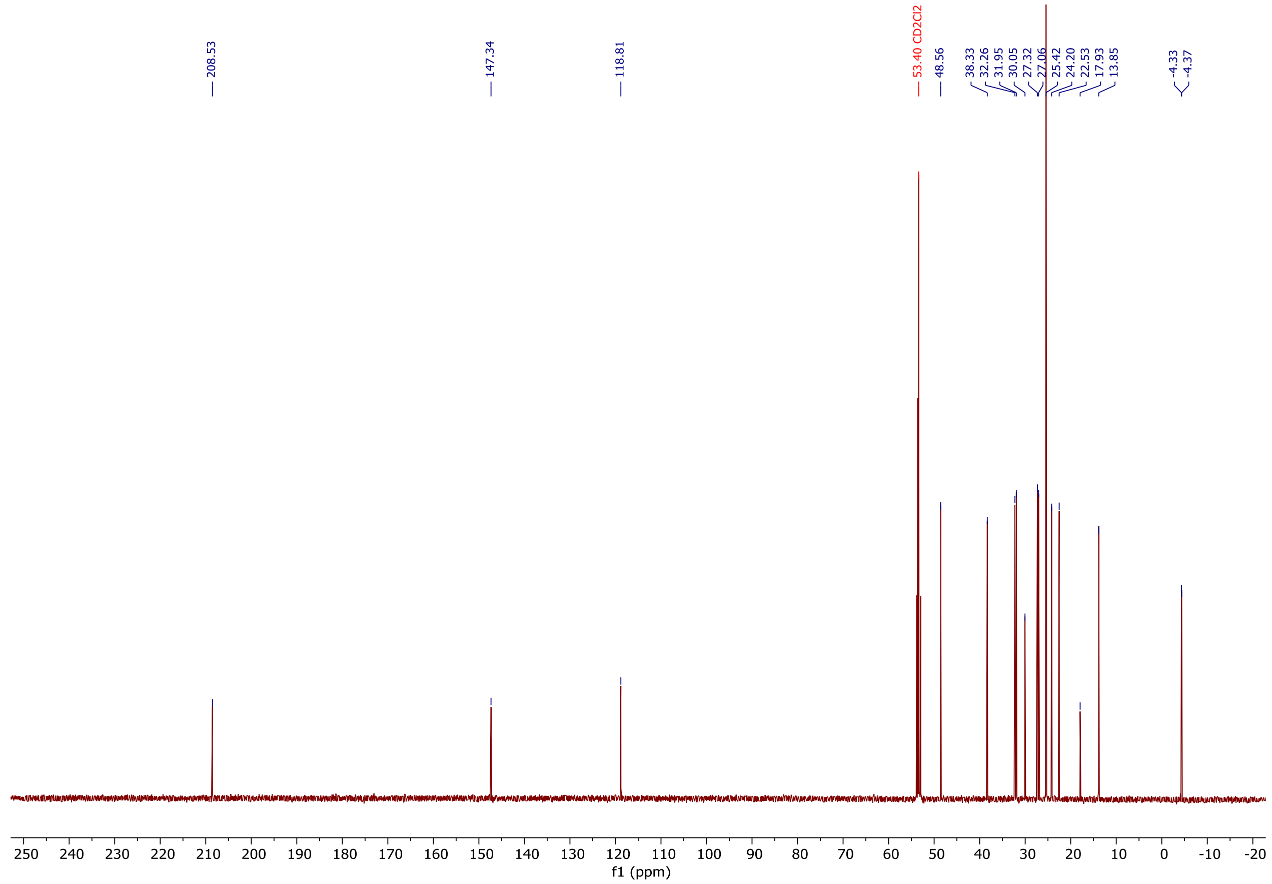

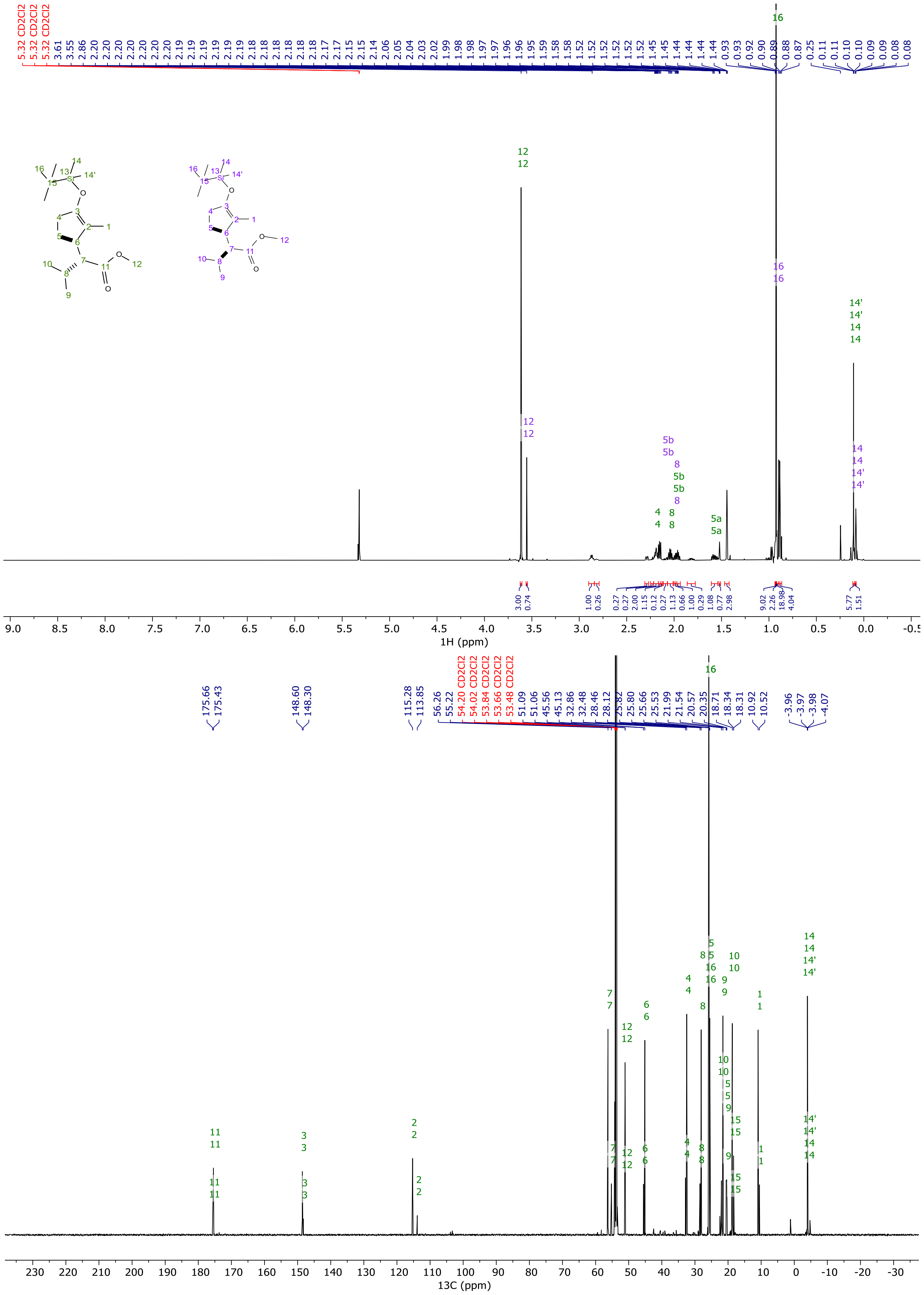

HSQC spectra of 3ae

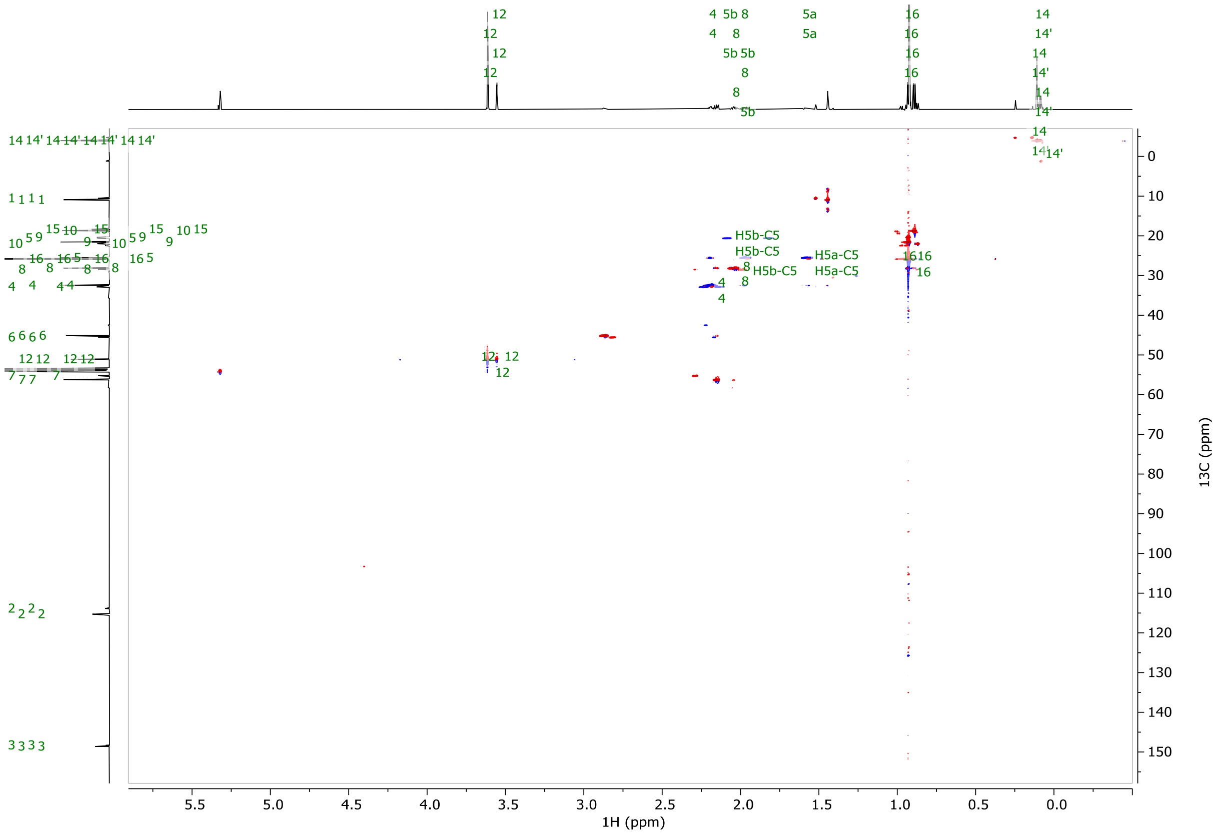

HMBC spectra of 3ae

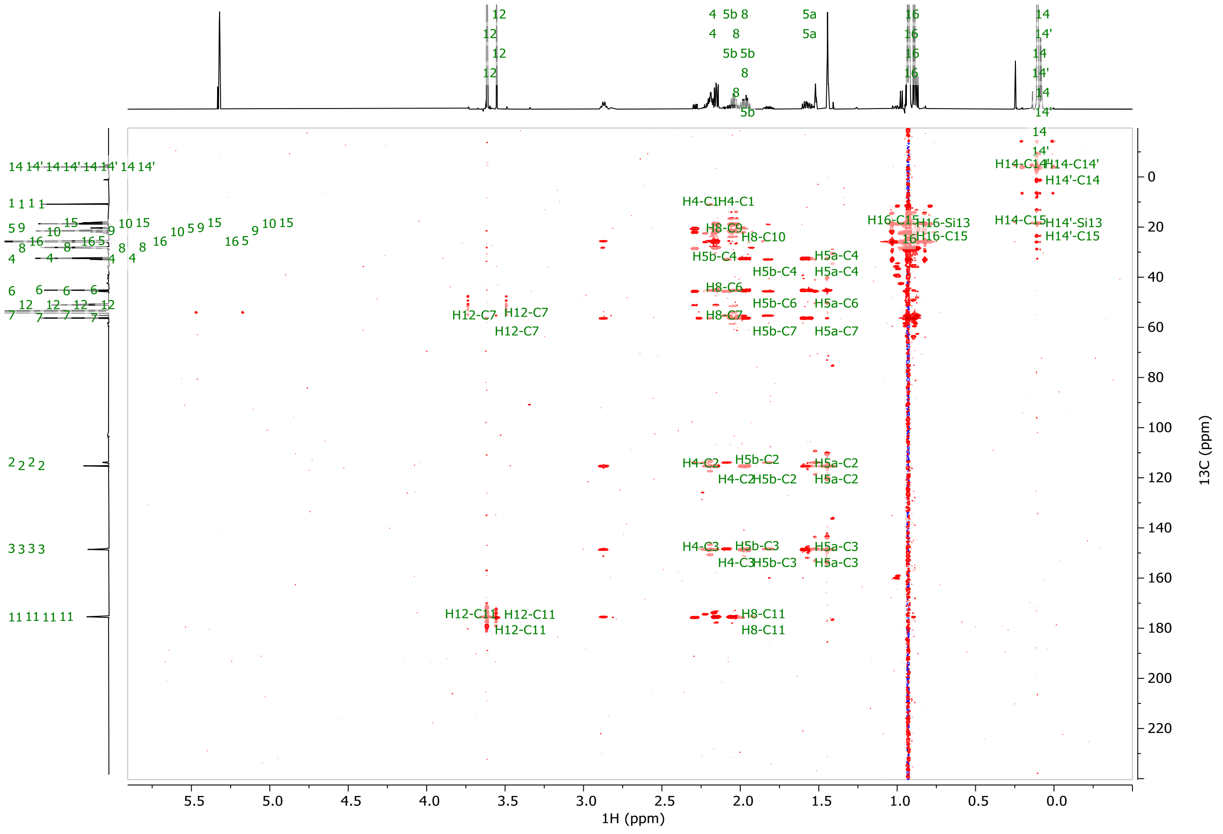

NOESY spectra of 3ae

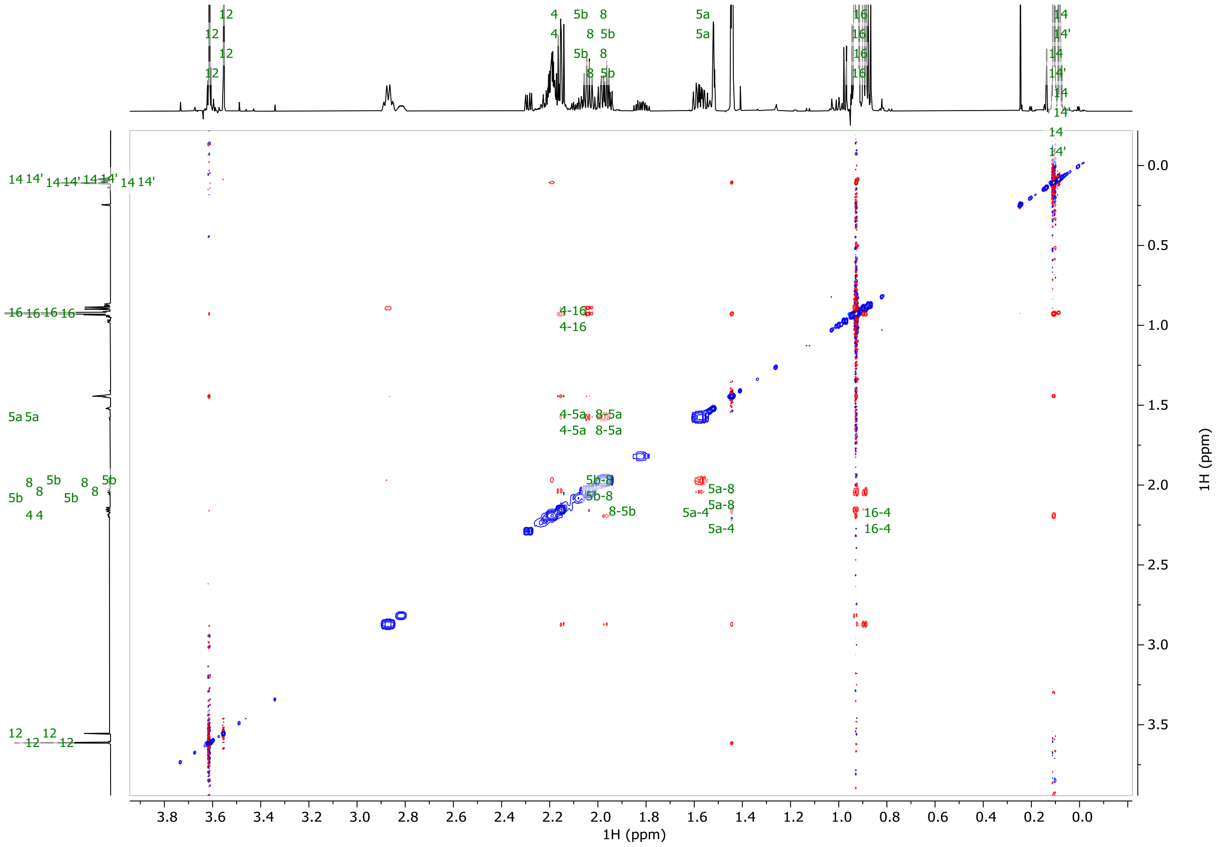

COSY spectra of 3ae

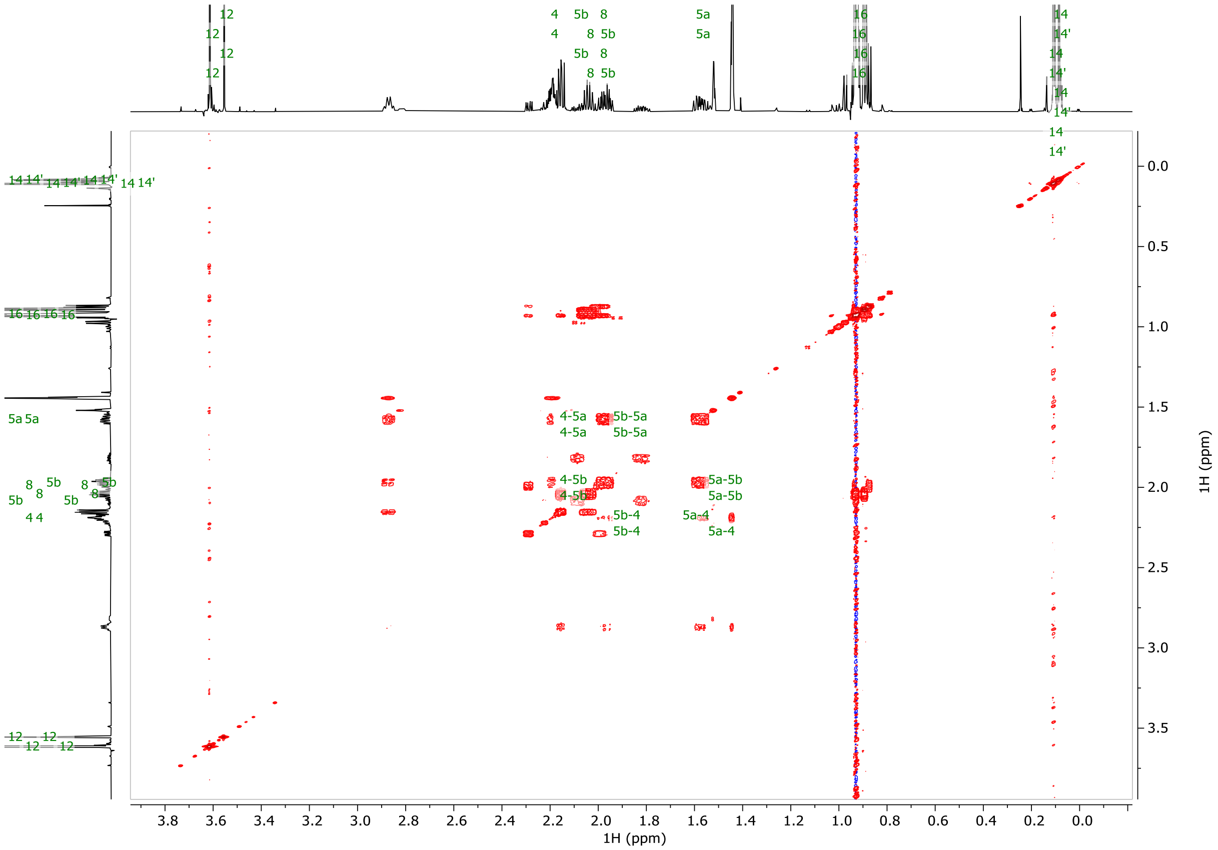

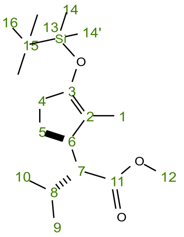

Atom	δ (ppm)	Min..Max (ppm)	J	COSY	HSQC	HMBC	NOESY
1 C	10.92	10.92..10.92			1	4	
H3	1.44	1.44..1.45		4, 6	1	2, 3, 4, 6, 13	6, 7, 8, 12, 14, 14'
2 C	115.28	115.27..115.28				1, 4, 5a, 5b, 6, 7	
3 C	148.60	148.59..148.60				1, 4, 5a, 5b, 6	
4 C	32.48	32.48..32.49			4	1, 5a, 5b	
H2	2.19	2.17..2.21		1, 5a, 5b	4	1, 2, 3, 6, 13	5a, 16
5 C	25.53	25.53..25.53			5a, 5b	6, 7	
Ha	1.57	1.54..1.61	12.7(5b), 8.8(?), 7.3(?), 6.3(?)	4, 5b, 6	5	2, 3, 4, 6, 7	4, 7, 8
Hb	1.97	1.94..2.00	12.7(5a)	4, 5a, 6	5	2, 3, 4, 6, 7	6, 10
6 C	45.13	45.12..45.13			6	1, 4, 5a, 5b, 7, 8	
H	2.87	2.83..2.91	8.2(7)	1, 5a, 5b, 7	6	2, 3, 5, 7, 8	1, 5b, 9, 10
7 C	56.26	56.26..56.27			7	5a, 5b, 6, 8, 9, 10, 12	
H	2.15	2.14..2.17	8.2(6), 6.2(8)	6, 8, 9, 10	7	2, 5, 6, 8, 9, 10, 11	1, 5a, 9, 10, 12
8 C	28.12	28.12..28.12			8	6, 7, 9, 10	
H	2.04	2.01..2.07	6.9(9), 6.7(10), 6.2(7)	7, 9, 10	8	6, 7, 9, 10, 11	1, 5a, 9, 10
9 C	21.54	21.54..21.55			9	7, 8, 10	
H3	0.93	0.91..0.95	6.9(8)	7, 8	9	7, 8, 10	6, 7, 8
10 C	18.71	18.70..18.71			10	7, 8, 9	
H3	0.89	0.88..0.90	6.7(8)	7, 8	10	7, 8, 9	5b, 6, 7, 8
11 C	175.43	175.43..175.44				7, 8, 12	
12 C	51.09	51.09..51.10			12		
H3	3.61	3.61..3.61			12	7, 11	1, 7
13 Si	19.45	19.45..19.46				1, 4, 14, 14', 16	
14 C	-3.97	-3.97..-3.97			14	14'	
H3	0.11	0.11..0.11			14	13, 14', 15	1
14' C	-3.98	-3.98..-3.97			14'	14	
H3	0.10	0.10..0.10			14'	13, 14, 15	1
15 C	18.34	18.33..18.34				14, 14', 16	
16 C	25.82	25.81..25.82			16	16	
H3	0.93	0.93..0.93			16	13, 15, 16	4

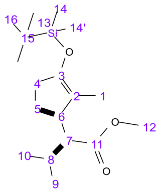

Atom	δ (ppm)	Min..Max (ppm)	J	COSY	HSQC	HMBC	NOESY
1 C	10.52	10.52..10.53			1	6	
H3	1.52	1.51..1.53		4a, 4b, 6	1	2, 3, 4, 6, 13	6, 7, 12
2 C	113.85	113.85..113.86				1, 4b, 5a, 5b, 7	
3 C	148.30	148.30..148.31				1, 5a, 5b	
4 C	32.86	32.86..32.87			4a, 4b	1, 5a, 5b	
Ha	2.13	2.14..2.17		1, 4b, 5a, 5b	4		5a
Hb	2.23	2.27..2.30		1, 4a, 5a, 5b	4	2	
5 C	20.57	20.57..20.58			5a, 5b	7	
Ha	1.82	1.78..1.86	13.1(5b), 10.0(?), 9.3(?), 5.5(?)	4a, 4b, 5b, 6	5	2, 3, 4, 6, 7	4a, 6, 8
Hb	2.08	2.05..2.11	13.1(5a)	4a, 4b, 5a, 6	5	2, 3, 4, 6, 7	8
6 C	45.56	45.55..45.56			6	1, 5a, 5b, 7	
H	2.82	2.79..2.84	4.5(7)	1, 5a, 5b, 7	6	1, 7, 9, 11	1, 5a, 7, 9
7 C	55.22	55.22..55.23			7	5a, 5b, 6, 8, 9, 10, 12	
H	2.29	2.27..2.30	4.5(6), 10.5(8)	6, 8, 9, 10	7	2, 5, 6, 8, 9, 10, 11	1, 6, 9, 10, 12
8 C	28.46	28.46..28.47			8	7, 9, 10	
H	1.99	1.96..2.01	10.5(7), 6.7(9), 6.6(10)	7, 9, 10	8	7, 9, 10	5a, 5b, 9, 10

Atom	δ (ppm)	Min..Max (ppm)	J	COSY	HSQC	HMBC	NOESY
9 C	20.35	20.34..20.35			9	6, 7, 8, 10	
H3	0.93	0.91..0.95	6.7(8)	7, 8	9	7, 8, 10	6, 7, 8
10 C	21.99	21.99..22.00			10	7, 8, 9	
H3	0.87	0.87..0.88	6.6(8)	7, 8	10	7, 8, 9	7, 8
11 C	175.66	175.66..175.66				6, 7, 12	
12 C	51.06	51.06..51.07			12		
H3	3.55	3.55..3.55			12	7, 11	1, 7
13 Si	19.15	19.14..19.15				1, 14, 14'	
14 C	-3.96	-3.96..-3.95			14	14'	
H3	0.09	0.09..0.09			14	13, 14', 15	
14' C	-4.07	-4.08..-4.07			14'	14	
H3	0.08	0.08..0.08			14'	13, 14, 15	
15 C	18.31	18.31..18.32				14, 14', 16	
16 C	25.80	25.80..25.81			16	16	
H3	0.92	0.92..0.92			16	15, 16	

^3J(H7/H6) = 8.2 Hz
^3J(H7/H8) = 6.2 Hz

NOE:
H6 - H5b
H7 - H5a

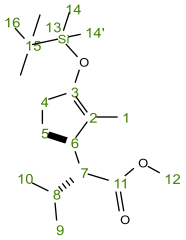

4 : 1

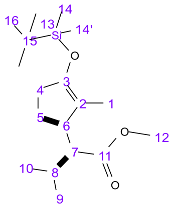

^3J(H7/H6) = 4.5 Hz
^3J(H7/H8) = 10.5 Hz

NOE:
H6 - H7
H1 - H7 strong

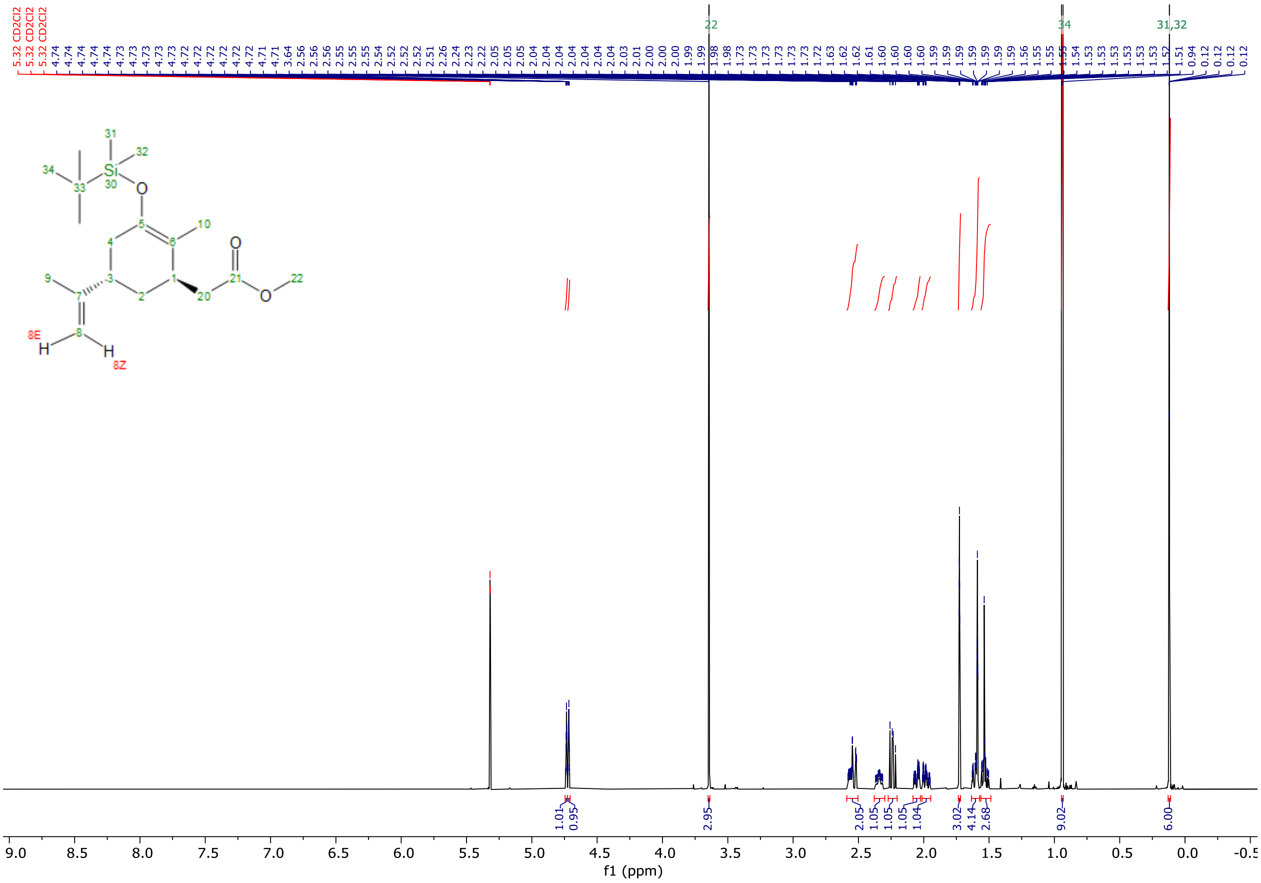
f1 (ppm)

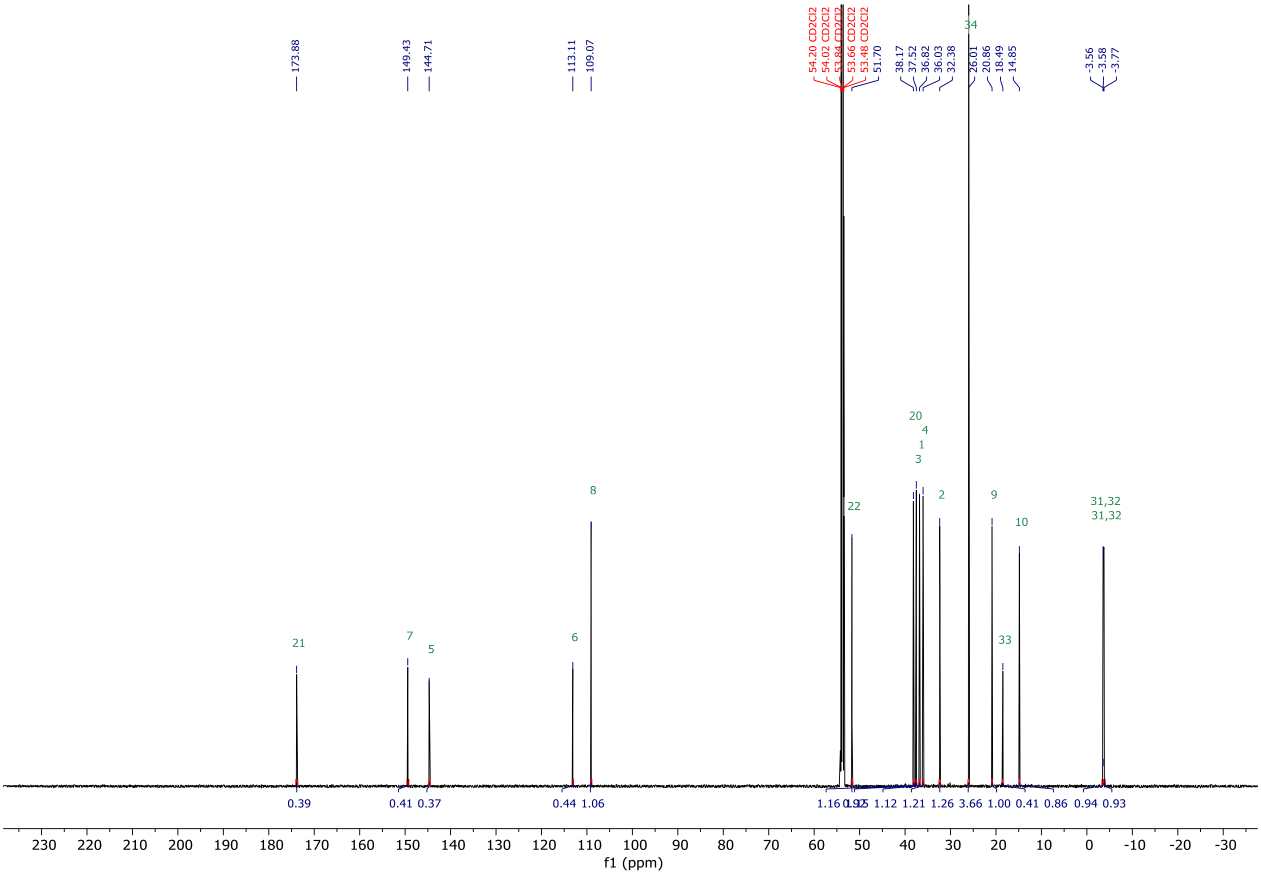
f1 (ppm)

NOESY spectra of 3af

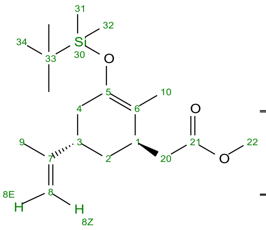

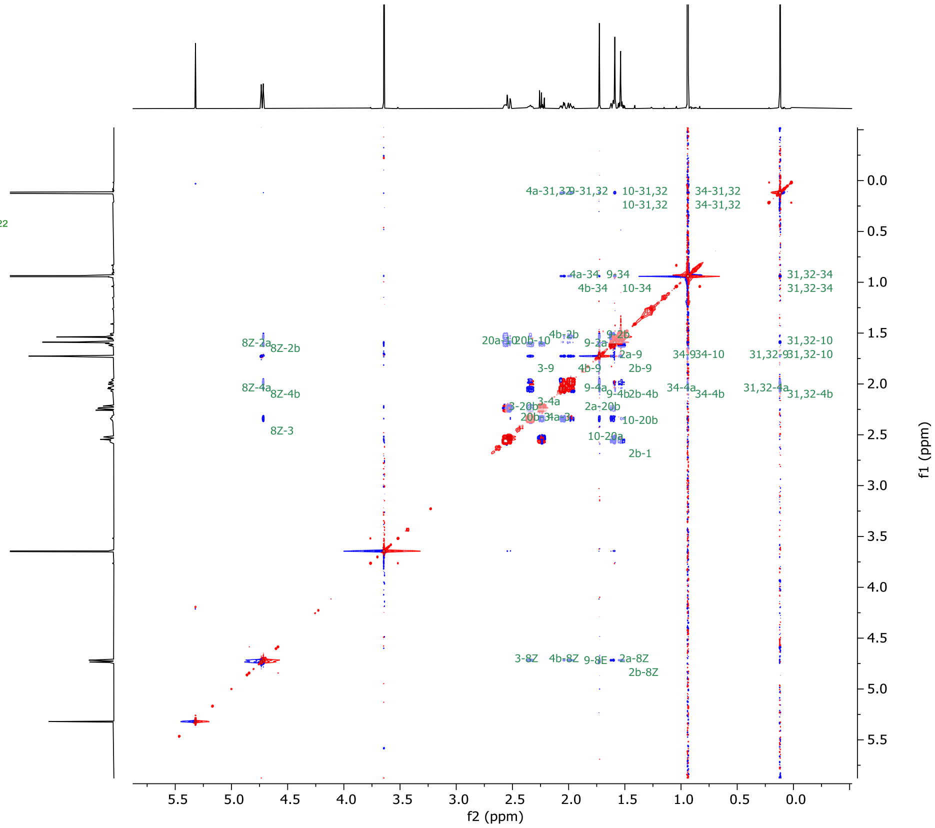

- in NOESY : H2b - H4b
H3 - H2a, H4a, H20b
H1 - H2a, H2b, H10

R-XA-821-01.15.1.2rr — XUR-XA-821-01 (12204) — CD2Cl2; 298.0 K; 8 mg; 5 mm — 1H (noesygpphpp) — AV600neo (cryoBBO) — 13.04.24 1

HMBC spectra of 3af

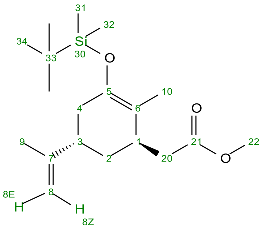

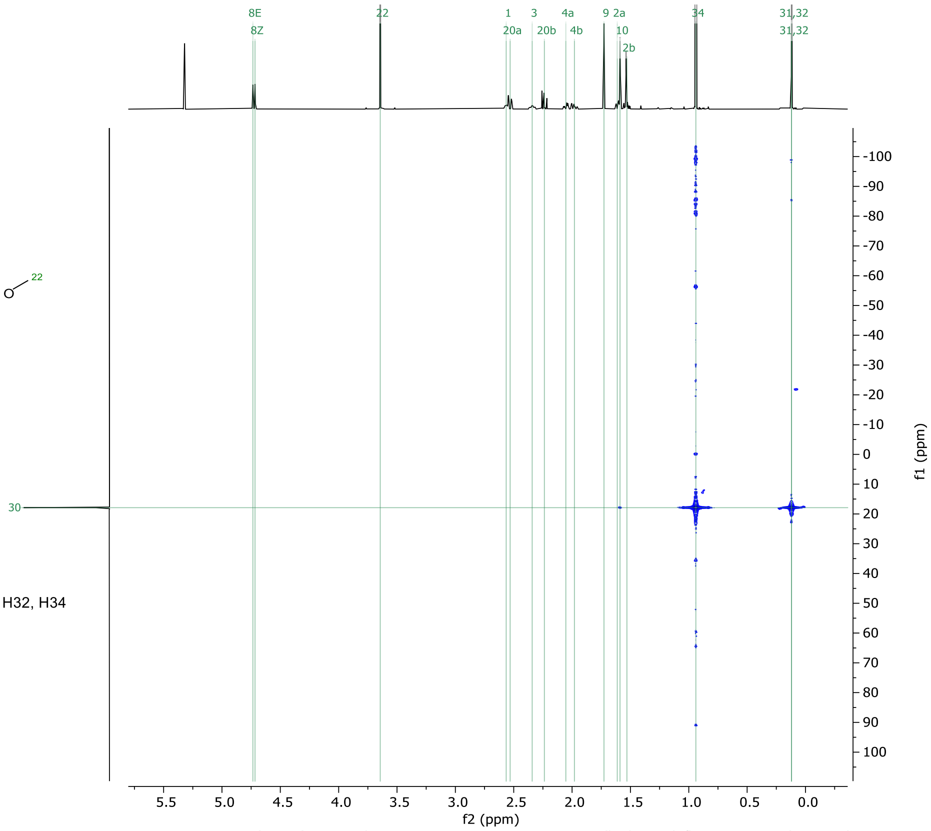

- in 29Si HMBC:
δ29Si = 17.90 ppm
Si30 - ((H10)), H31, H32, H34

XA-821-01.17.1.2rr — XUR-XA-821-01 (12204) — CD2Cl2; 298.0 K; 8 mg; 5 mm — 1H-29Si (hmbcgpndqf) — AV600neo (cryoBBO) — 13.04.24

HMBC spectra of 3af

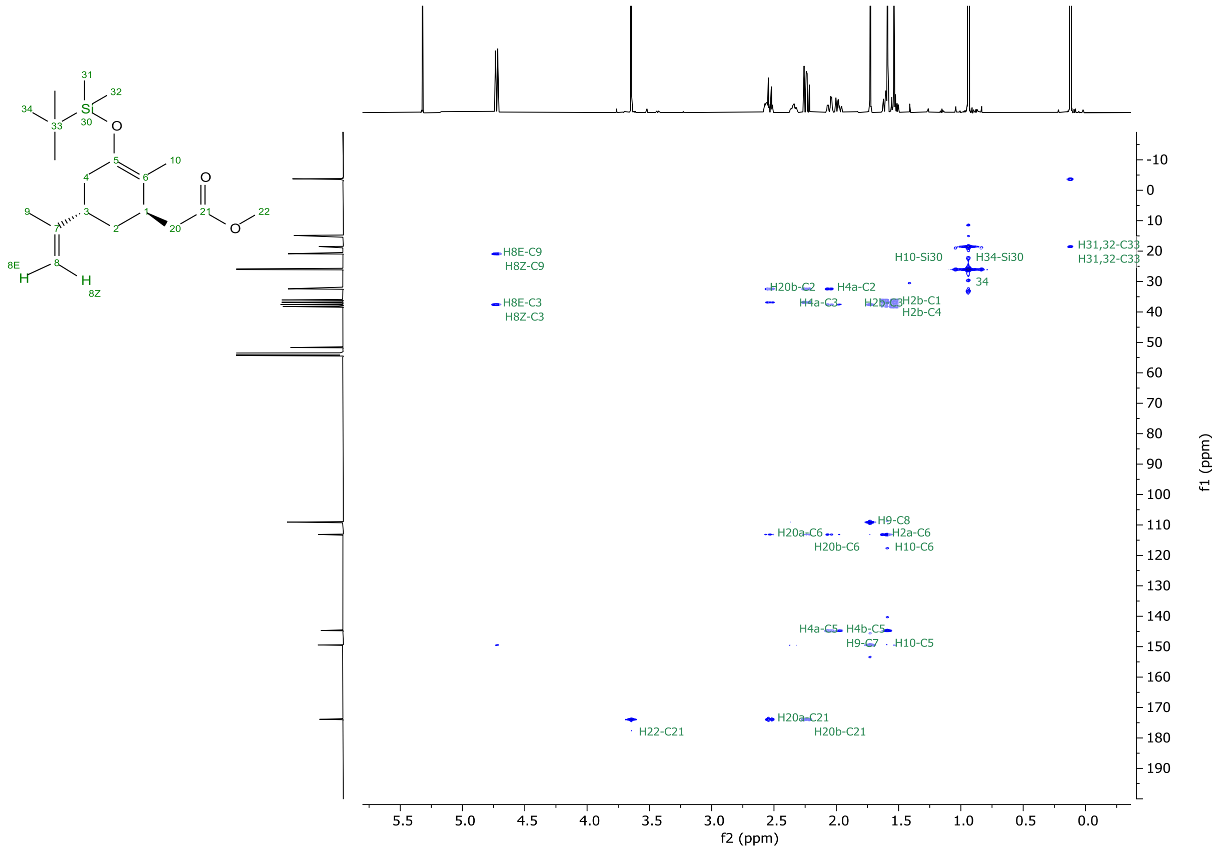

R-XA-821-01.13.1.2rr — XUR-XA-821-01 (12204) — CD2Cl2; 298.0 K; 8 mg; 5 mm — 1H-13C (hmbcetgpl3nd) — AV600neo (cryoBBO) — 13.04.24 1

HSQC spectra of 3af

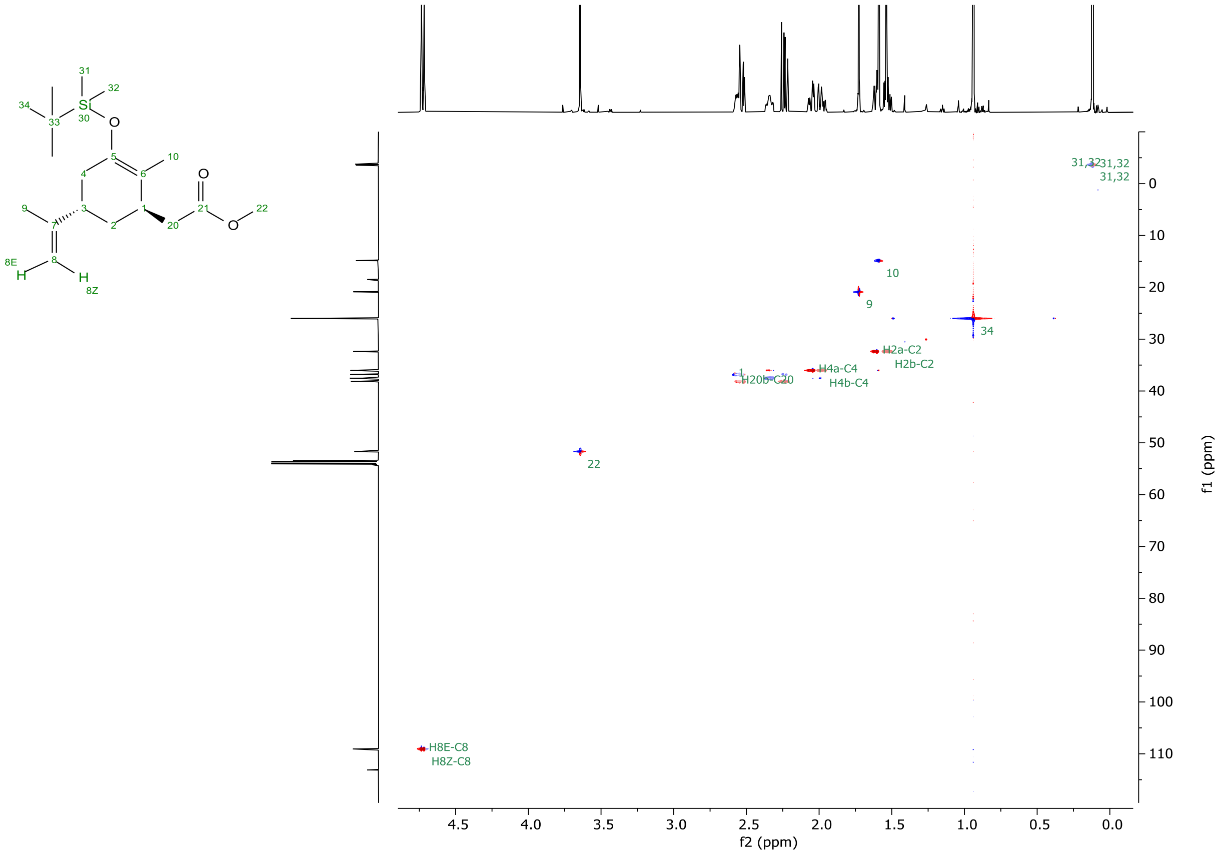

-821-01.12.1.2rr — XUR-XA-821-01 (12204) — CD2Cl2; 298.0 K; 8 mg; 5 mm — 1H-13C (hsqcedetgpsisp2.3) — AV600neo (cryoBBO) — 14.04

COSY spectra of 3af

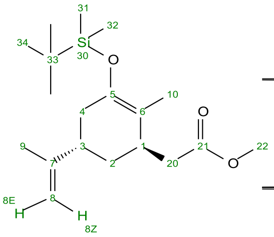

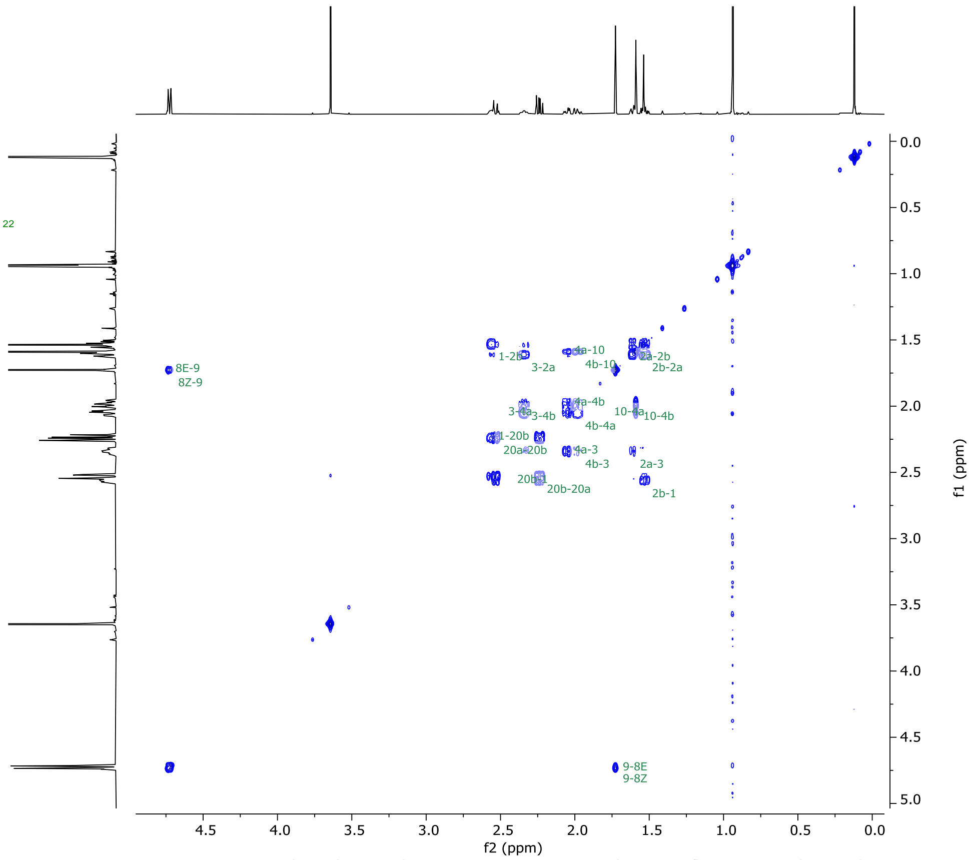

JR-XA-821-01.14.1.2rr — XUR-XA-821-01 (12204) — CD2Cl2; 298.0 K; 8 mg; 5 mm — 1H (cosygpppqf) — AV600neo (cryoBBO) — 14.04.24 10

Result in NMR:

Regarding the results in NOESY and the JHH coupling constances in 1H data the relative stereochemistry of the proposed compound can be confirmed as followed.

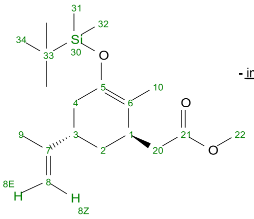

- in 1H: J H3,H2b = 11.45 Hz trans, aa
J H3,H4b = 10.98 Hz trans, aa
J H1,H2a/ H2b = ~ 5.30 Hz /4.34 Hz ~gauche !?

H3 axial up,
H2b axial down,
H4b axial down
H1 down

Mol Formula: $C_{19}H_{34}O_3Si$
Av Mass: 338.56
Mol Composition: C:67.40% H:10.12% O:14.18% Si:8.30%
Molecule DBE: 4

Atom	δ (ppm)	J	COSY	HSQC	HMBC	NOESY
1 C	36.82			1	2a, 2b, 10, 20a, 20b	
H	2.56	10.30(20b), 5.30(?), 4.34(?)	2b, 20b	1		2a, 2b, 10
2 C	32.38			2a, 2b	4a, 20a, 20b	
Ha	1.61	13.19(2b), 3.06(?), 1.65(?), 1.65(?)	2b, 3	2	1, 3, 4, 6, 20	1, 3, 8Z, 9, 20b
Hb	1.53	13.19(2a), 11.45(3), 5.38(?), 1.38(?)	1, 2a	2	1, 3, 4, 20	1, 4b, 8Z, 9
3 C	37.52			3	2a, 2b, 4a, 8E, 8Z, 9	
H	2.34	10.98(4b), 11.45(2b), 5.54(?), 2.75(?)	2a, 4a, 4b	3		2a, 4a, 8Z, 9, 20b
4 C	36.03			4a, 4b	2a, 2b	
Ha	2.05	16.48(4b), 5.55(?), 1.55(?), 1.55(?), 0.78(?)	3, 4b, 10	4	2, 3, 5	3, 8Z, 9, 31, 32, 34
Hb	1.98	16.48(4a), 10.98(3), 3.78(?), 1.80(?), 1.59(?)	3, 4a, 10	4	5	2b, 8Z, 9, 31, 32, 34
5 C	144.71				4a, 4b, 10	
6 C	113.11				2a, 10, 20a, 20b	
7 C	149.43				9	
8 C	109.07			8E, 8Z	9	
8E H	4.74	1.75(?), 1.75(?), 1.75(?), 1.43(?), 0.53(?)	9	8	3, 9	9
8Z H	4.72	1.96(?), 1.96(?), 0.83(?), 0.83(?)	9	8	3, 9	2a, 2b, 3, 4a, 4b
9 C	20.86			9	8E, 8Z	
H3	1.73	1.38(?), 0.84(?), 0.39(?)	8E, 8Z	9	3, 7, 8	2a, 2b, 3, 4a, 4b, 8E, 31, 32, 34

Atom	δ (ppm)	J	COSY	HSQC	HMBC	NOESY
10 C	14.85			10		
H3	1.59	2.24(?), 1.57(?), 0.64(?)	4a, 4b	10	1, 5, 6, 30	1, 20a, 20b, 31, 32, 34
20 C	38.17			20a, 20b	2a, 2b	
Ha	2.53	14.90(20b), 3.72(?), 1.24(?)	20b	20	1, 2, 6, 21	10
Hb	2.24	10.30(1), 14.90(20a)	1, 20a	20	1, 2, 6, 21	2a, 3, 10
21 C	173.88				20a, 20b, 22	
22 C	51.70			22		
H3	3.64			22	21	
30 Si	17.90				10, 31, 32, 34	
31 C	-3.77, -3.56			31	32	
H3	0.12, 0.12			31	30, 32, 33	4a, 4b, 9, 10, 34
32 C	-3.77, -3.56			32	31	
H3	0.12, 0.12			32	30, 31, 33	4a, 4b, 9, 10, 34
33 C	18.49				31, 32	
34 C	26.01			34	34	
H3	0.94			34	30, 34	4a, 4b, 9, 10, 31, 32

P-ID: PP
Measured on: 14/04/2024
CHIFFRE: XUR-XA-821-01
ELNA#: 12204
Client: Ruigang Xu
Group: List
Spectroscopist: Philipps
Analysed on: 15/04/2024
Analysed by: Philipps
Amount: 8.0 mg
Solvent: CD2Cl2
Reference: 1H+13C on solvent,other nuclei w/ xiref
Temperature: 298 K
Spectrometer: av600neo
Probe: cryoBBO
Experiments: 1H-zg30, 13C-zgpg30, 1H-13C-hsqcedetgpsisp2.3, 1H-13C-hmbcetgpl3nd, 1H-1H-cosygpppqf, 1H-1H-noesygpphpp,29Si-ineptrd, 1H-29Si-hmbcgpndqf, 1H-seldigpzs

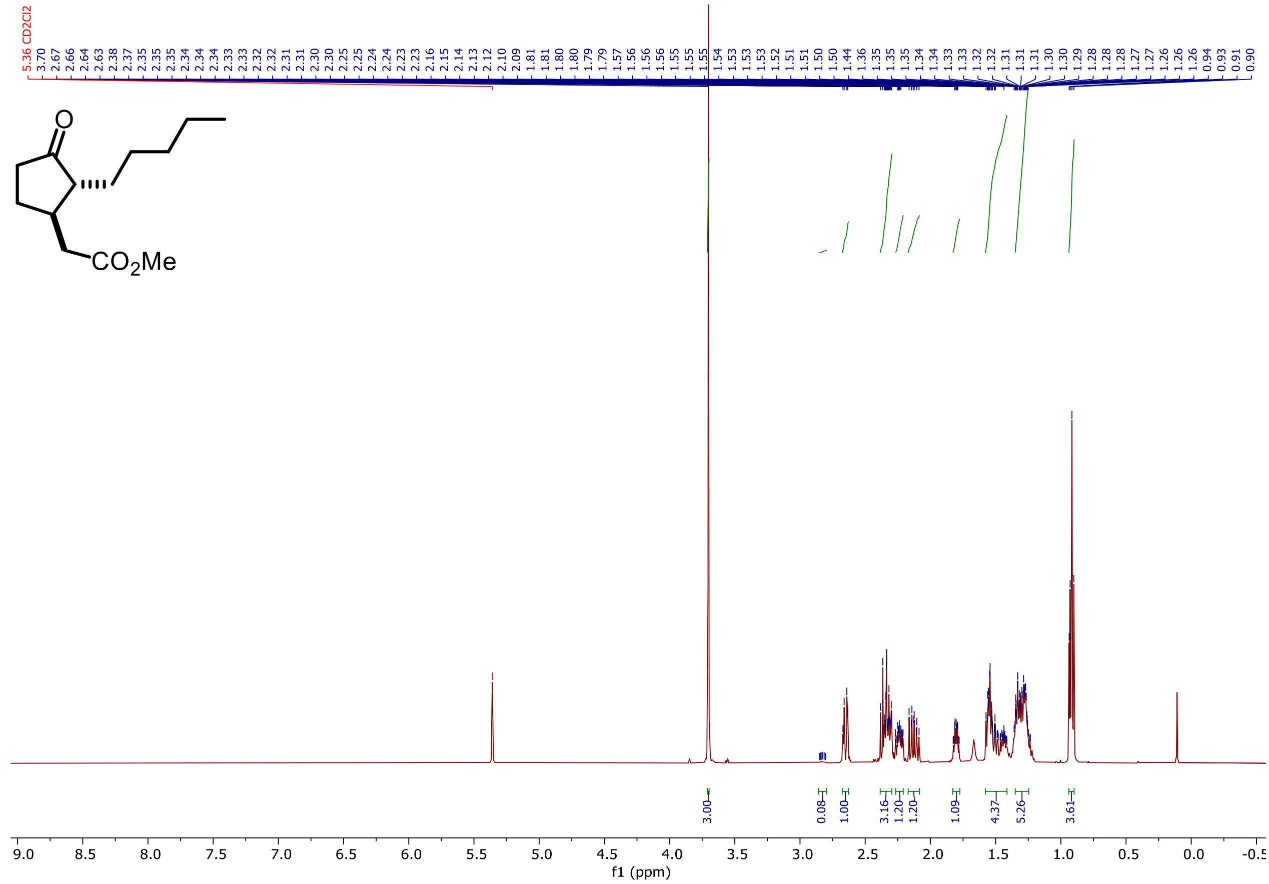

5.36 CD2Cl2
3.00
0.08
1.00
3.16
1.20
1.20
1.09
4.37
5.26
3.61
f1 (ppm)

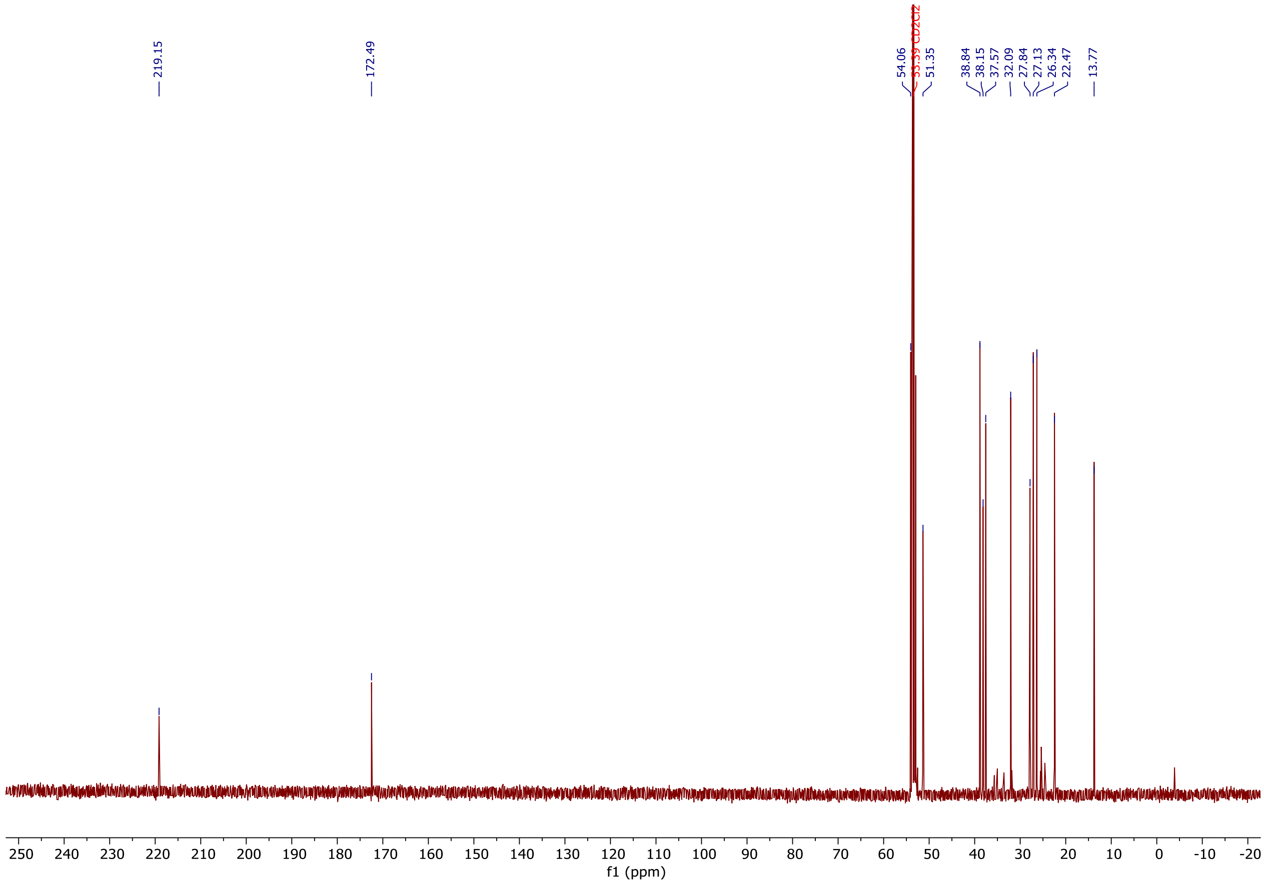

219.15
172.49
54.06
53.59 CD2Cl2
51.35
38.84
38.15
37.57
32.09
27.84
27.13
26.34
22.47
13.77
f1 (ppm)

HSQC spectra of 6a

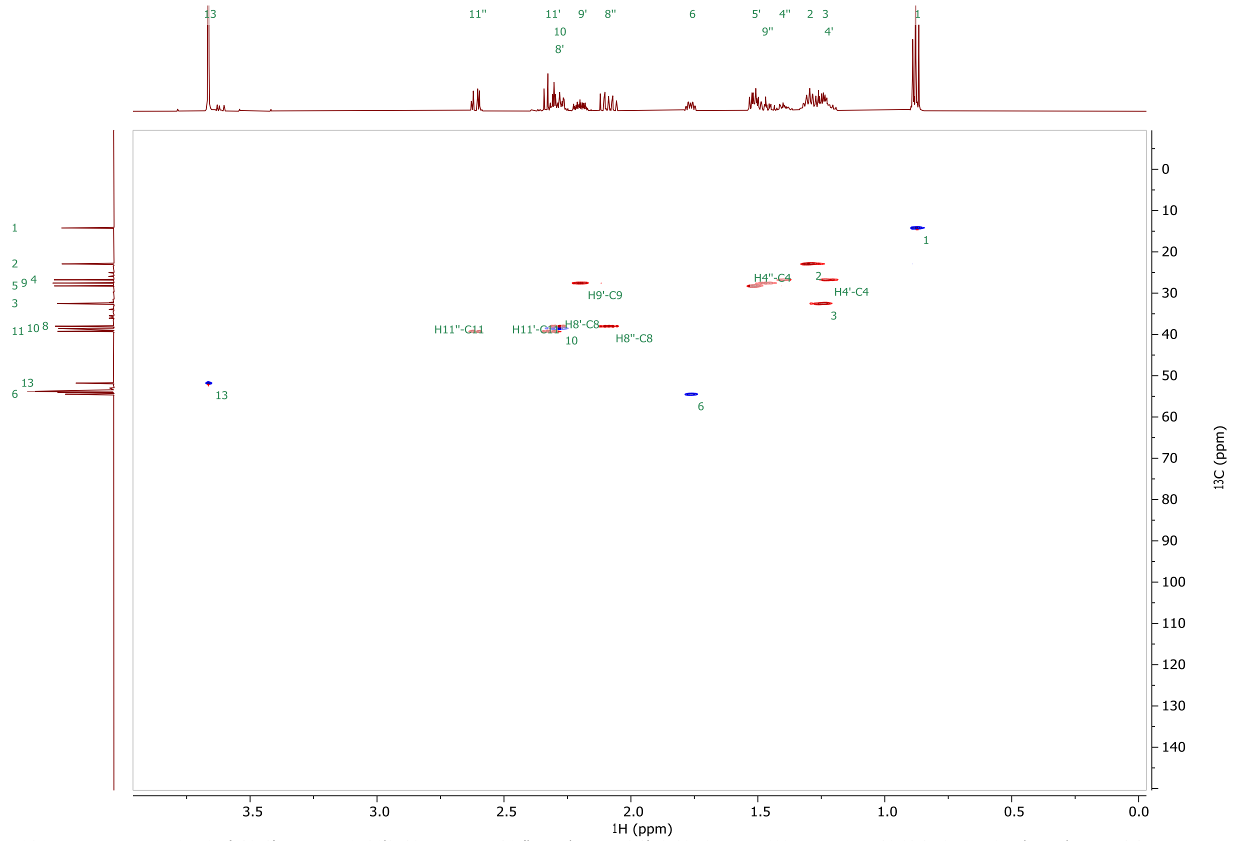

HSQC-EDITED — LIS-SR-057-01 (13913) — 5 mm, CD2Cl2, 20 mg — 1H-13C (hsqcedetgpsisp2.3) @ 297.9 K — AV600a, cryoTCI — 20.02.25 07:07:19 — hsqcedetgpsisp2.3

HMBC spectra of 6a

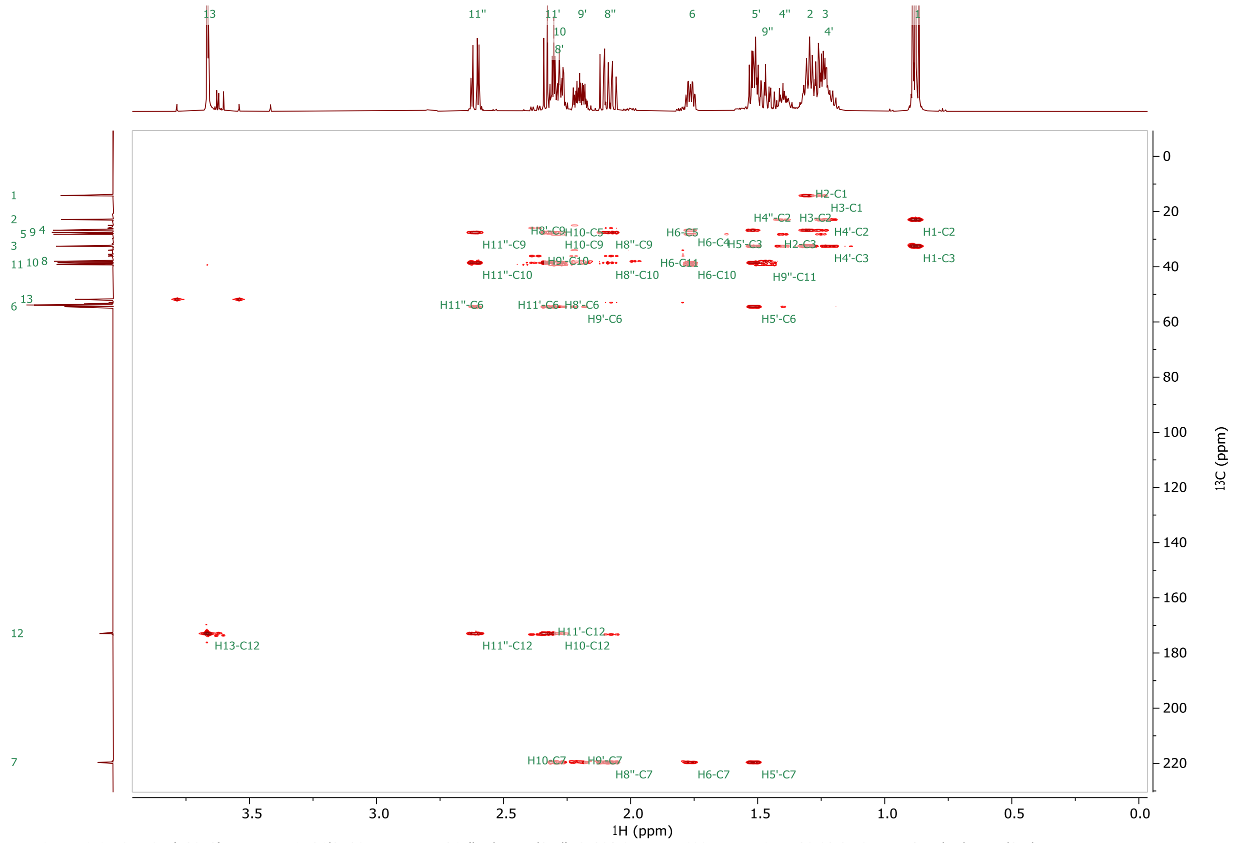

HMBC — LIS-SR-057-01 (13913) — 5 mm, CD2Cl2, 20 mg — 1H-13C (hmbcetgpl3nd) @ 298.0 K — AV600a, cryoTCI — 20.02.25 07:15:58 — hmbcetgpl3nd

NOESY spectra of 6a

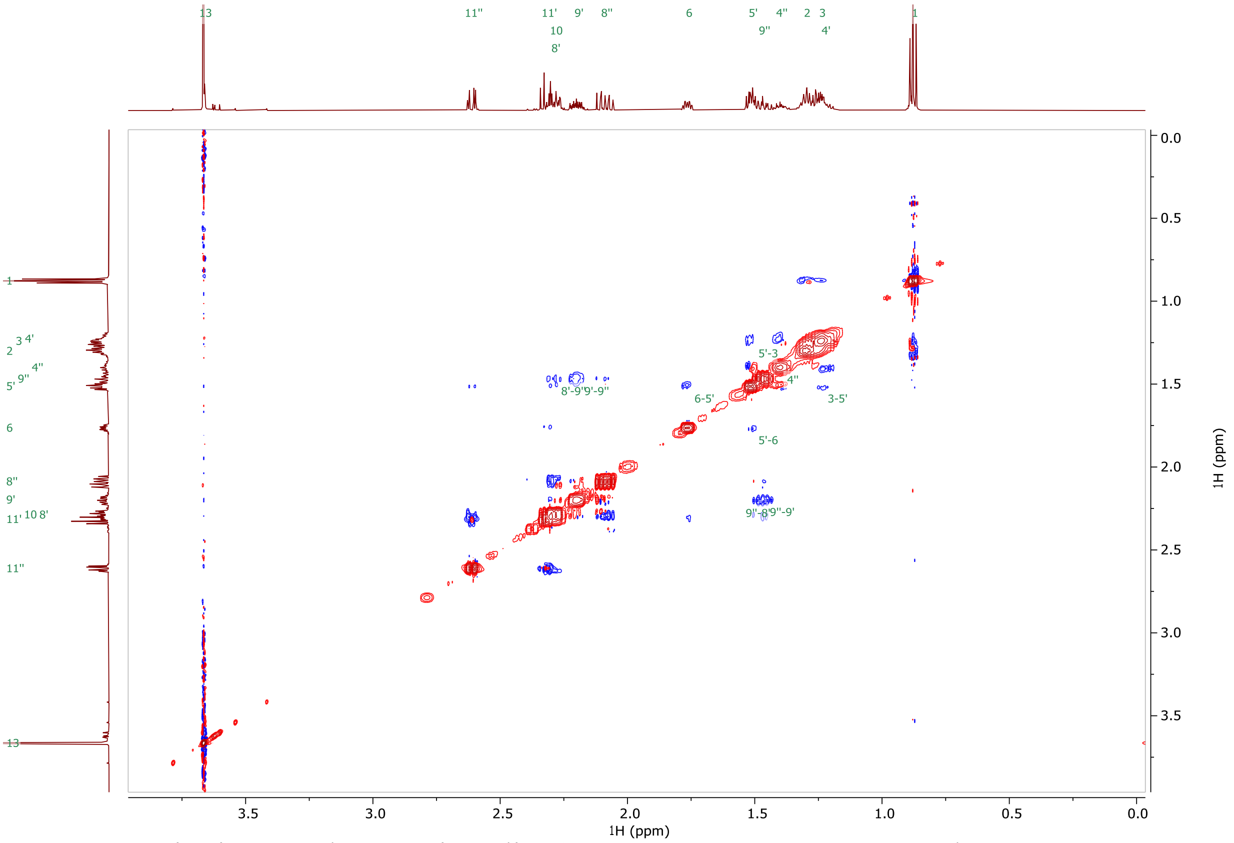

NOESY — LIS-SR-057-01 (13913) — 5 mm, CD2Cl2, 20 mg — 1H (noesygpph) @ 298.0 K — AV600a, cryoTCI — 20.02.25 07:54:14 — noesygpph

COSY spectra of 6a

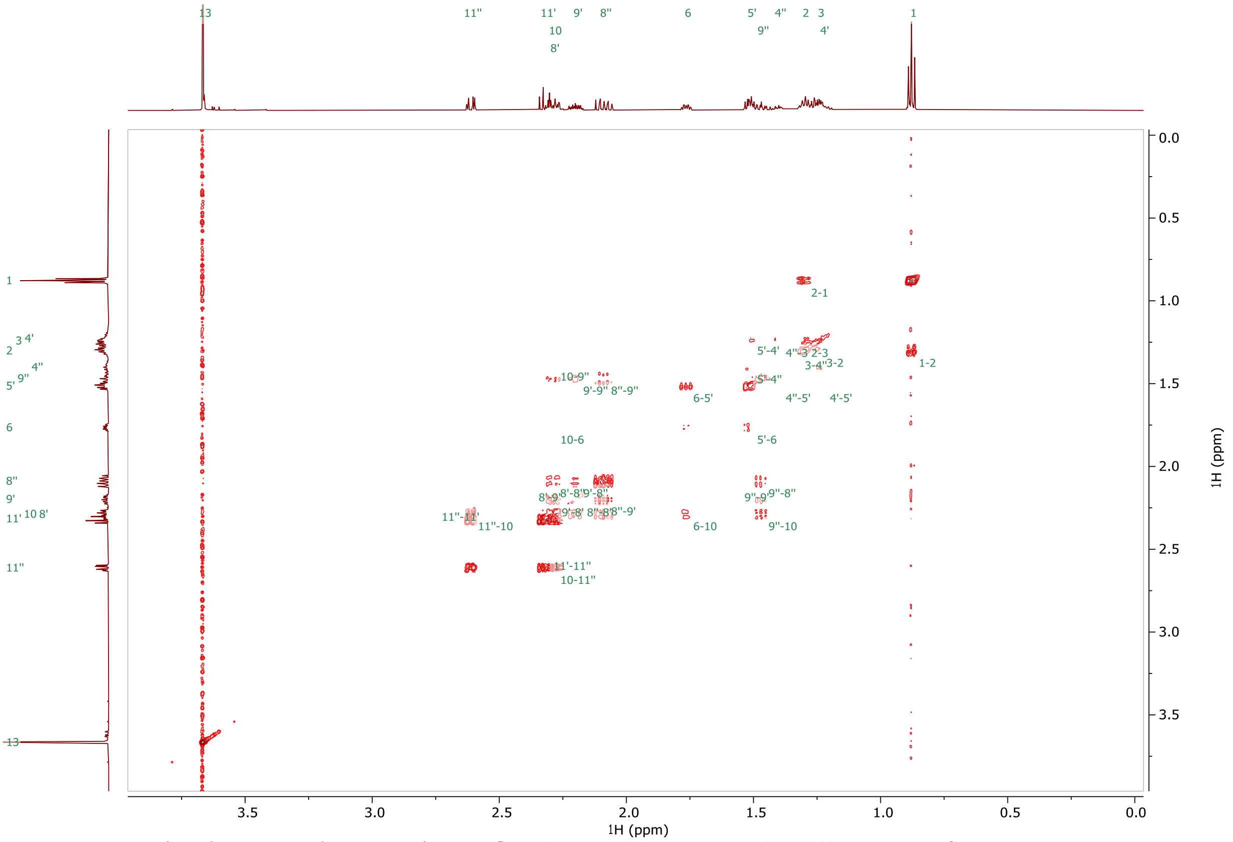

COSY — LIS-SR-057-01 (13913) — 5 mm, CD2Cl2, 5 mg — 1H (cosygpppqf) @ 298.0 K — AV600a, cryoTCI — 20.02.25 09:38:07 — cosygpppqf

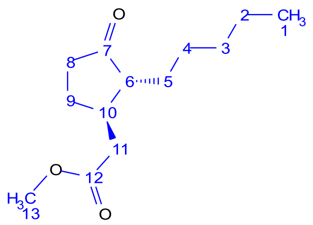

Atom	δ (ppm)	Predicted Shift	J	COSY	HSQC	HMBC	NOESY
1 C	14.211	14.10			1	2, 3	
H3	0.878	0.89	7.20(2)	2	1	2, 3	
2 C	22.909	22.52			2	1, 3, 4', 4"	
H2	1.302	1.30	7.20(1)	1, 3	2	1, 3, 4	
3 C	32.524	31.83			3	1, 2, 4', 4", 5'	
H2	1.242	1.28, 1.31		2, 4"	3	1, 2, 4	5'
4 C	26.759	27.31			4', 4"	2, 3, 5', 6	
H'	1.228	1.26, 1.38		5'	4	2, 3	
H"	1.401	1.26, 1.38		3, 5'	4	2, 3, 5	4"
5 C	28.237	28.19			5', 5"	4", 6, 10	
H'	1.514	1.49, 1.72		4', 4", 6	5	3, 4, 6, 7, 10	3, 6
H"		1.49, 1.72			5		
6 C	54.483	54.50			6	5', 8', 9', 11', 11"	
H	1.765	2.25		5', 10	6	4, 5, 7, 10, 11	5'
7 C	219.627	218.57				5', 6, 8', 8", 9', 10	
8 C	38.016	37.76			8', 8"		
H'	2.289	2.43, 2.57		8", 9'	8	6, 7, 9, 10	9"
H"	2.089	2.43, 2.57		8', 9', 9"	8	7, 9, 10	
9 C	27.568	28.14			9', 9"	8', 8", 10, 11', 11"	
H'	2.198	1.59, 2.04		8', 8", 9"	9	6, 7, 10	9"
H"	1.469	1.59, 2.04		8", 9', 10	9	11	8', 9'
10 C	38.551	36.46			10	5', 6, 8', 8", 9', 11', 11"	
H	2.288	2.34		6, 9", 11"	10	5, 7, 9, 11, 12	
11 C	39.258	38.74			11', 11"	6, 9", 10	
H'	2.316	2.22, 2.47		11"	11	6, 9, 10, 12	
H"	2.612	2.22, 2.47		10, 11'	11	6, 9, 10, 12	
12 C	172.933	173.35				10, 11', 11", 13	
13 C	51.799	51.73			13		
H3	3.666	3.67			13	12	

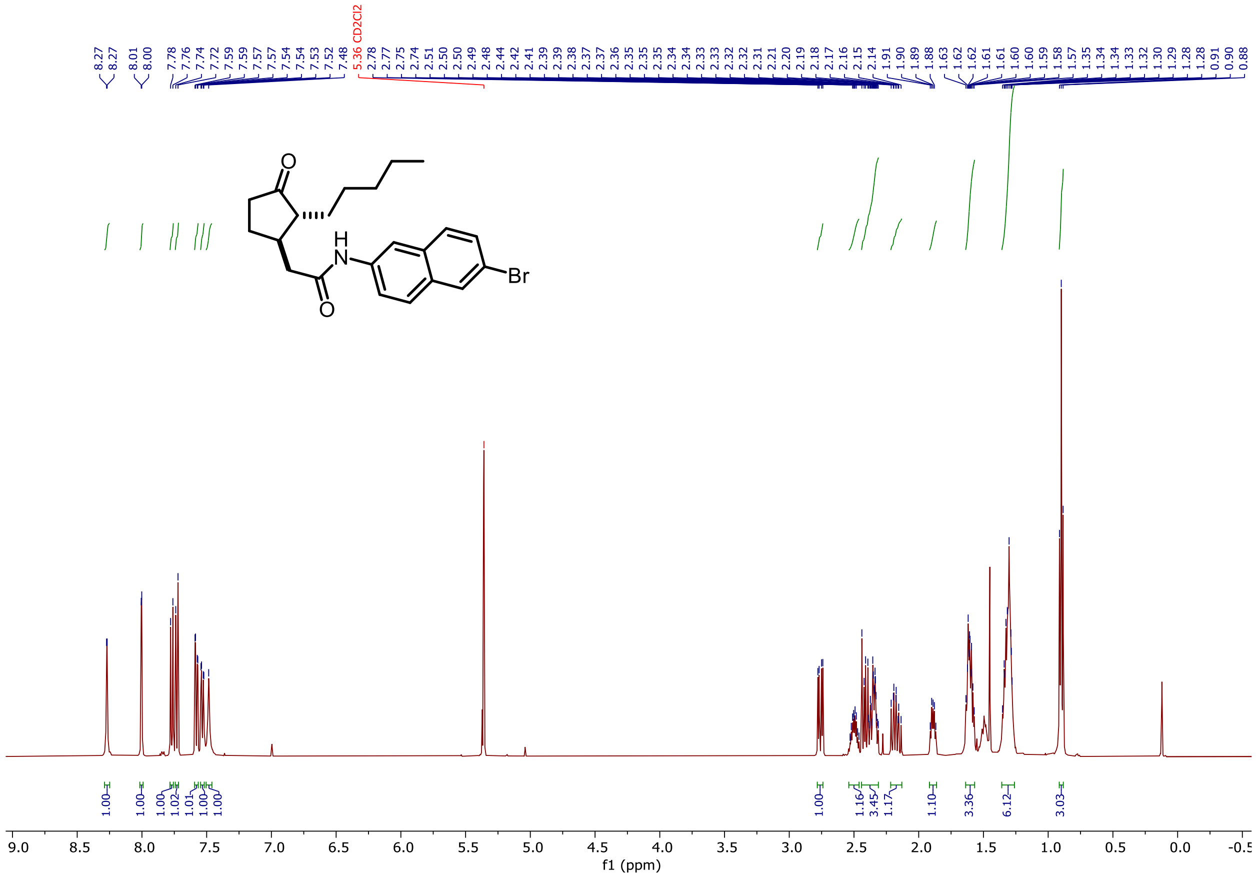

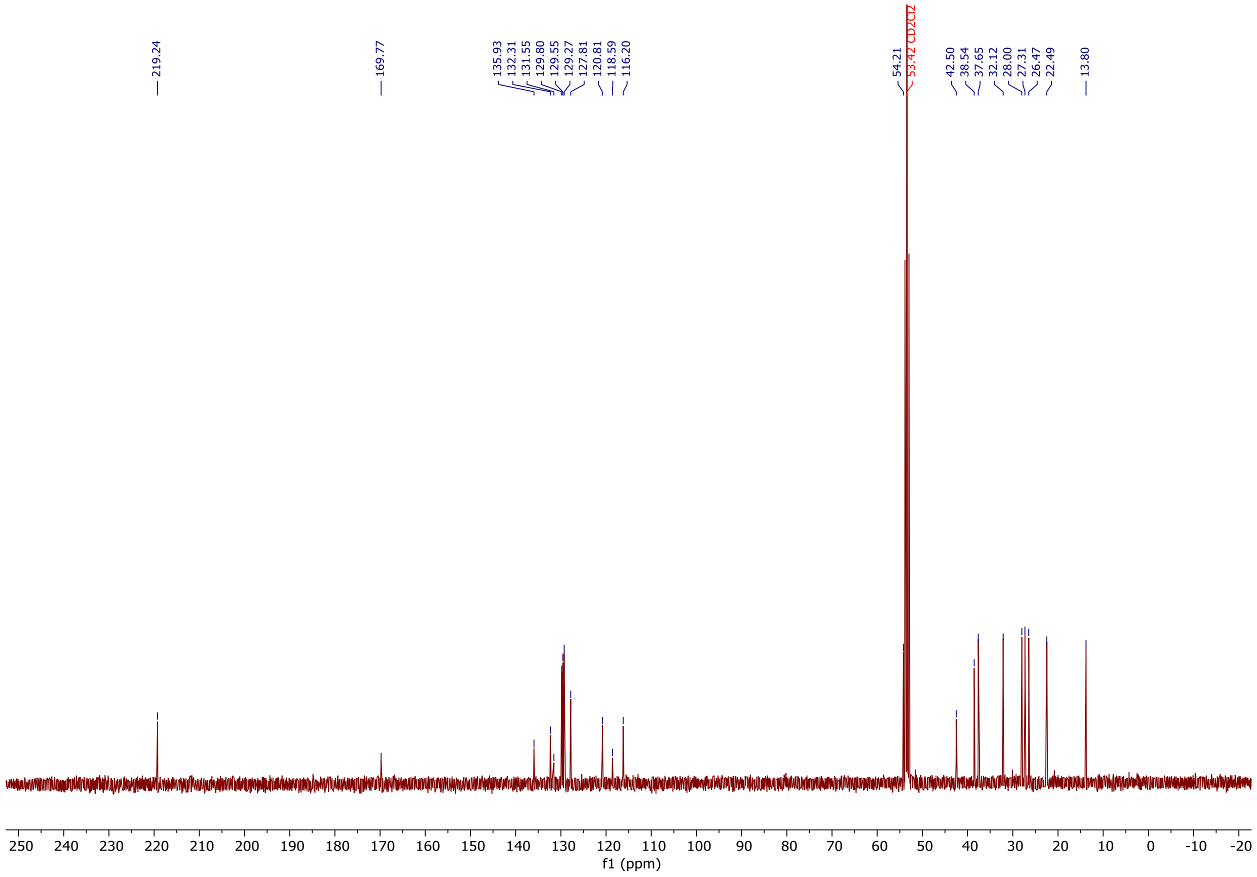

HSQC spectra of 7

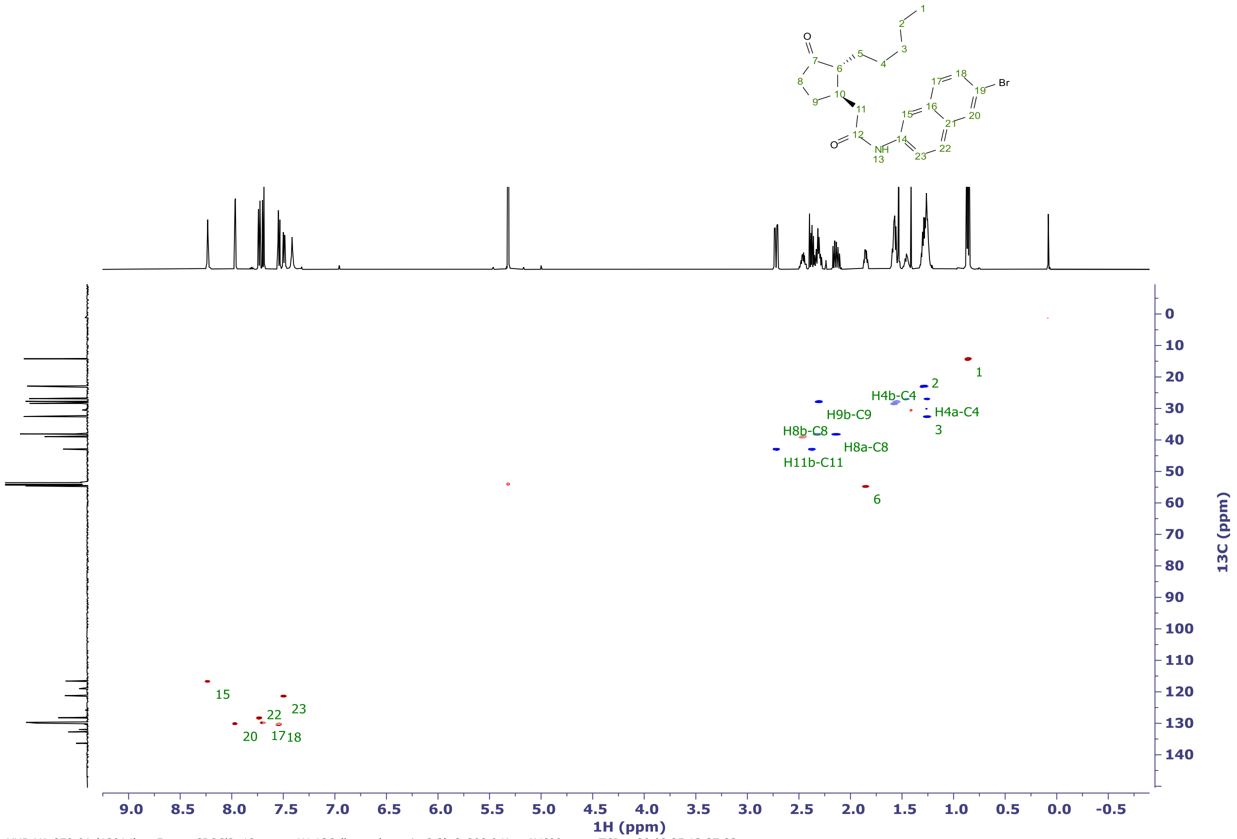

XUR-XA-878-01 (13914) — 5 mm, CD2Cl2, 12 mg — 1H-13C (hsqcedetgpsisp2.3) @ 298.0 K — AV600a, cryoTCI — 20.02.25 13:37:33

HMBC spectra of 7

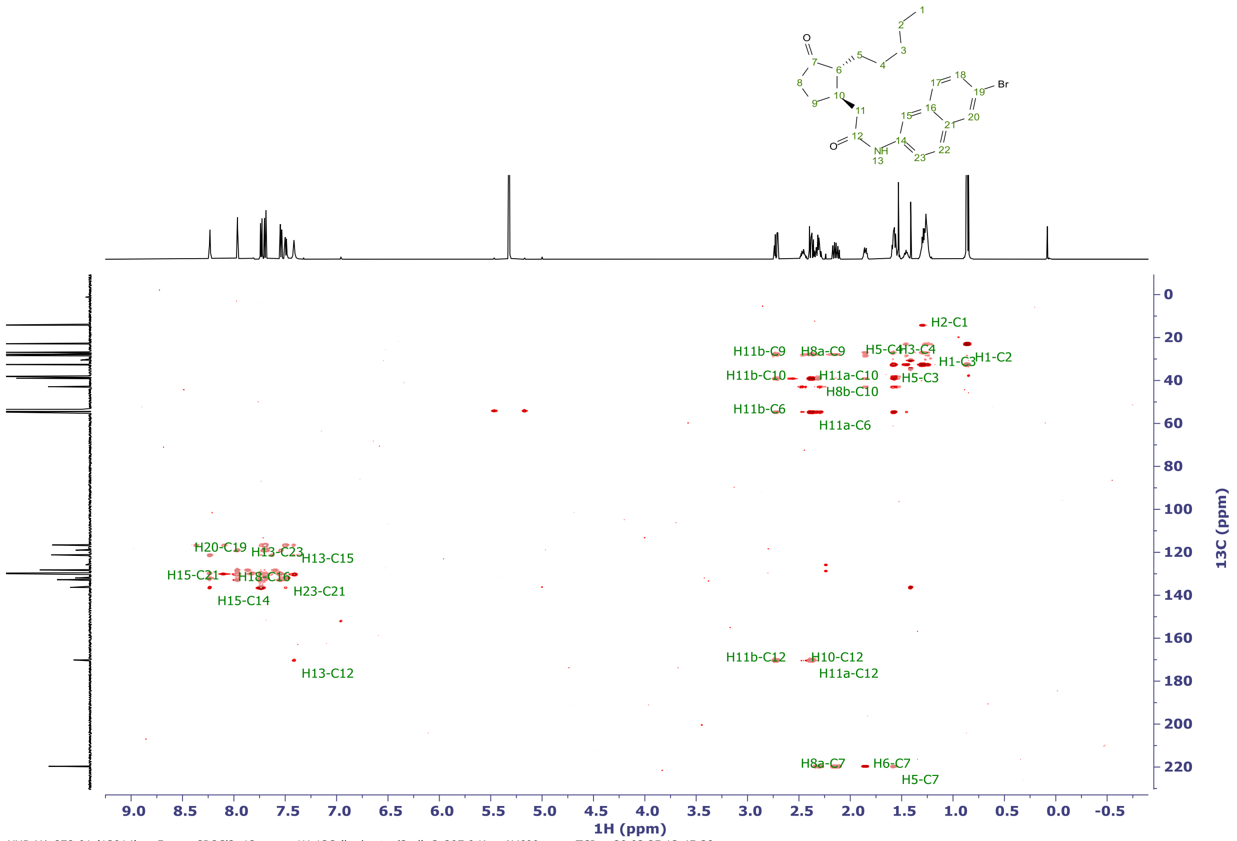

XUR-XA-878-01 (13914) — 5 mm, CD2Cl2, 12 mg — 1H-13C (hmbcetgpl3nd) @ 297.9 K — AV600a, cryoTCI — 20.02.25 13:45:38

COSY spectra of 7

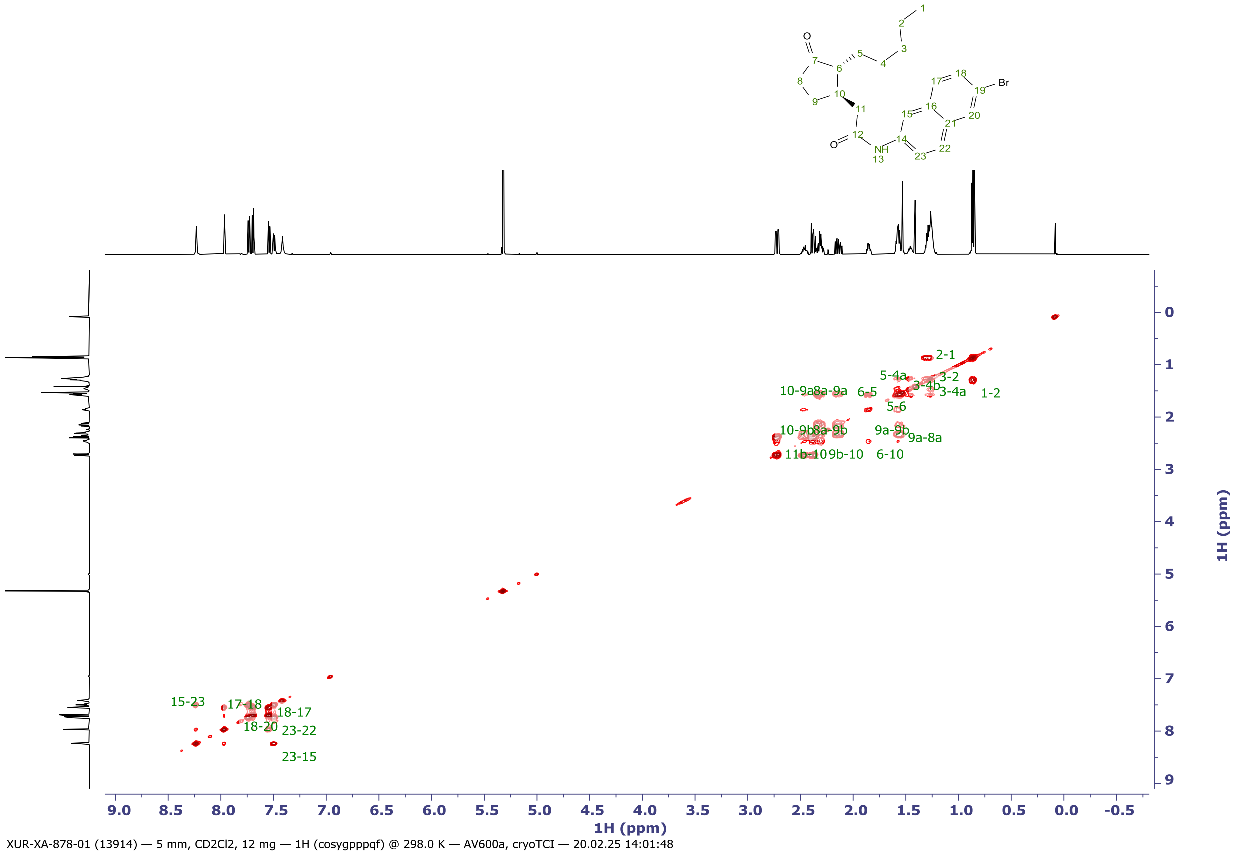

NOESY spectra of 7

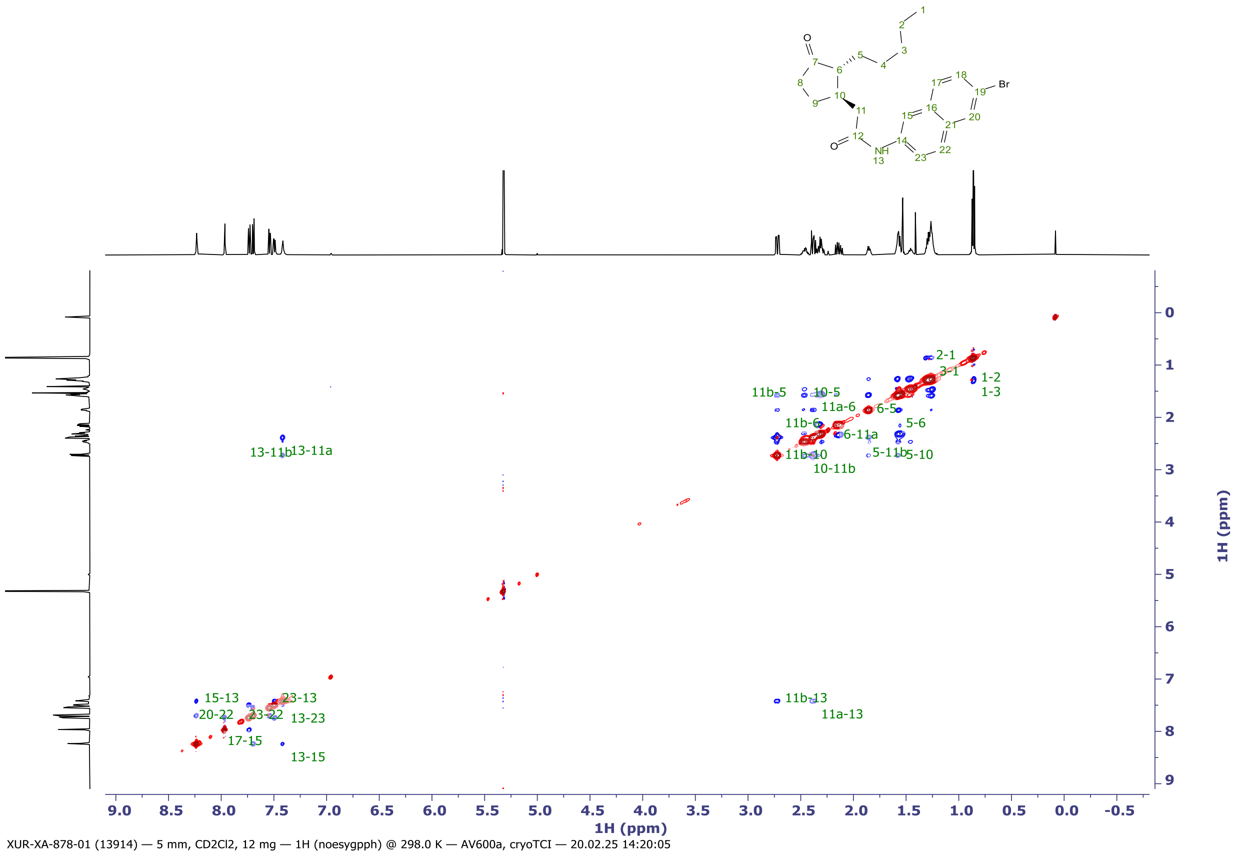

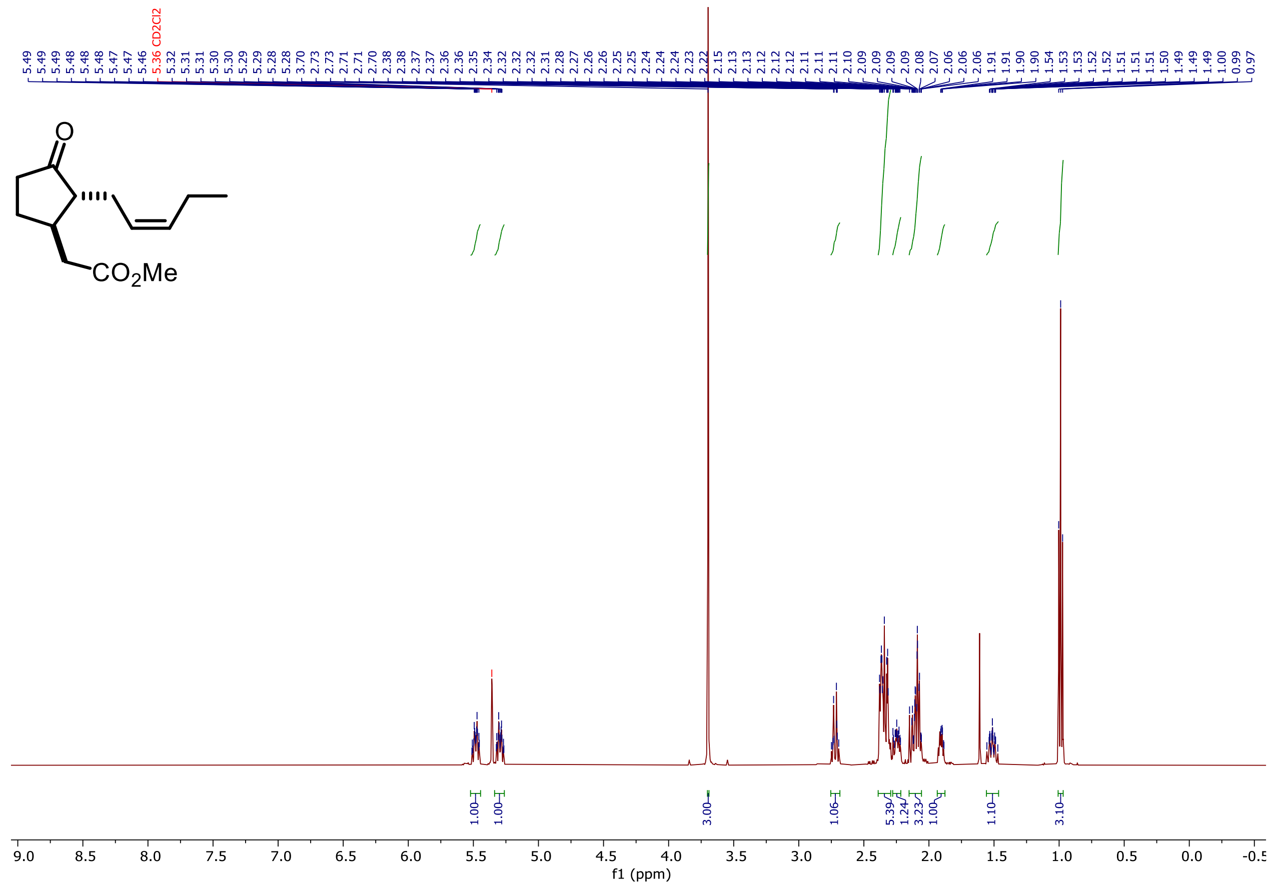
5.49 5.49 5.49 5.48 5.48 5.48 5.47 5.47 5.46 5.36 CD2Cl2 5.32 5.31 5.31 5.30 5.30 5.29 5.29 5.29 5.28 5.28 3.70 2.73 2.73 2.73 2.71 2.71 2.70 2.38 2.38 2.37 2.37 2.36 2.36 2.35 2.34 2.32 2.32 2.32 2.31 2.28 2.27 2.26 2.26 2.25 2.25 2.24 2.24 2.24 2.23 2.22 2.15 2.13 2.13 2.12 2.12 2.12 2.11 2.11 2.11 2.10 2.09 2.09 2.09 2.09 2.08 2.07 2.06 2.06 2.06 1.91 1.91 1.90 1.90 1.54 1.53 1.53 1.52 1.52 1.51 1.51 1.51 1.50 1.49 1.49 1.00 0.99 0.97
O
CO2Me
1.00 1.00 3.00 1.06 5.39 1.24 3.23 1.00 1.10 3.10
9.0 8.5 8.0 7.5 7.0 6.5 6.0 5.5 5.0 4.5 4.0 3.5 3.0 2.5 2.0 1.5 1.0 0.5 0.0 -0.5
f1 (ppm)

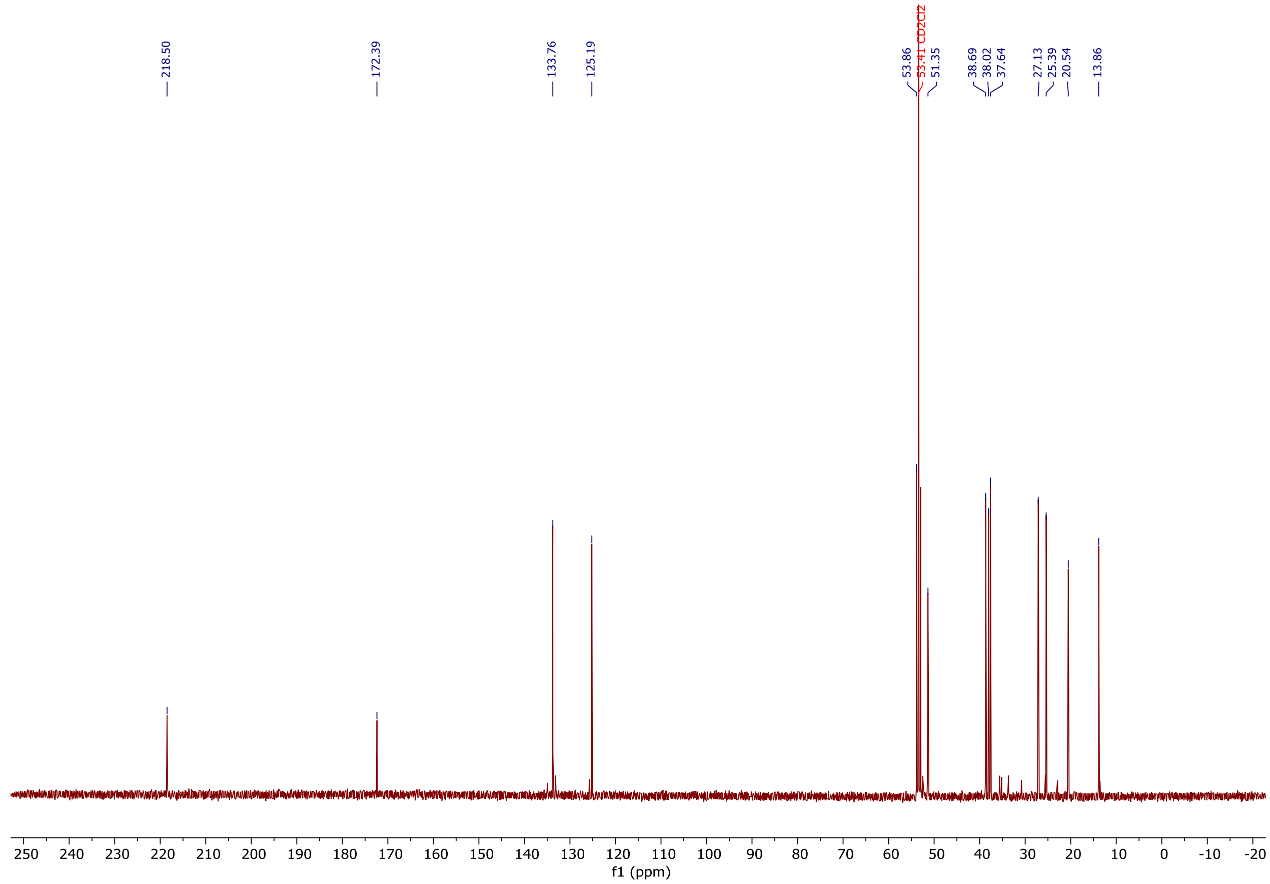
218.50 172.39 133.76 125.19 53.86 53.41 CD2Cl2 51.35 38.69 38.02 37.64 27.13 25.39 20.54 13.86
250 240 230 220 210 200 190 180 170 160 150 140 130 120 110 100 90 80 70 60 50 40 30 20 10 0 -10 -20
f1 (ppm)

HSQC spectra of 6c

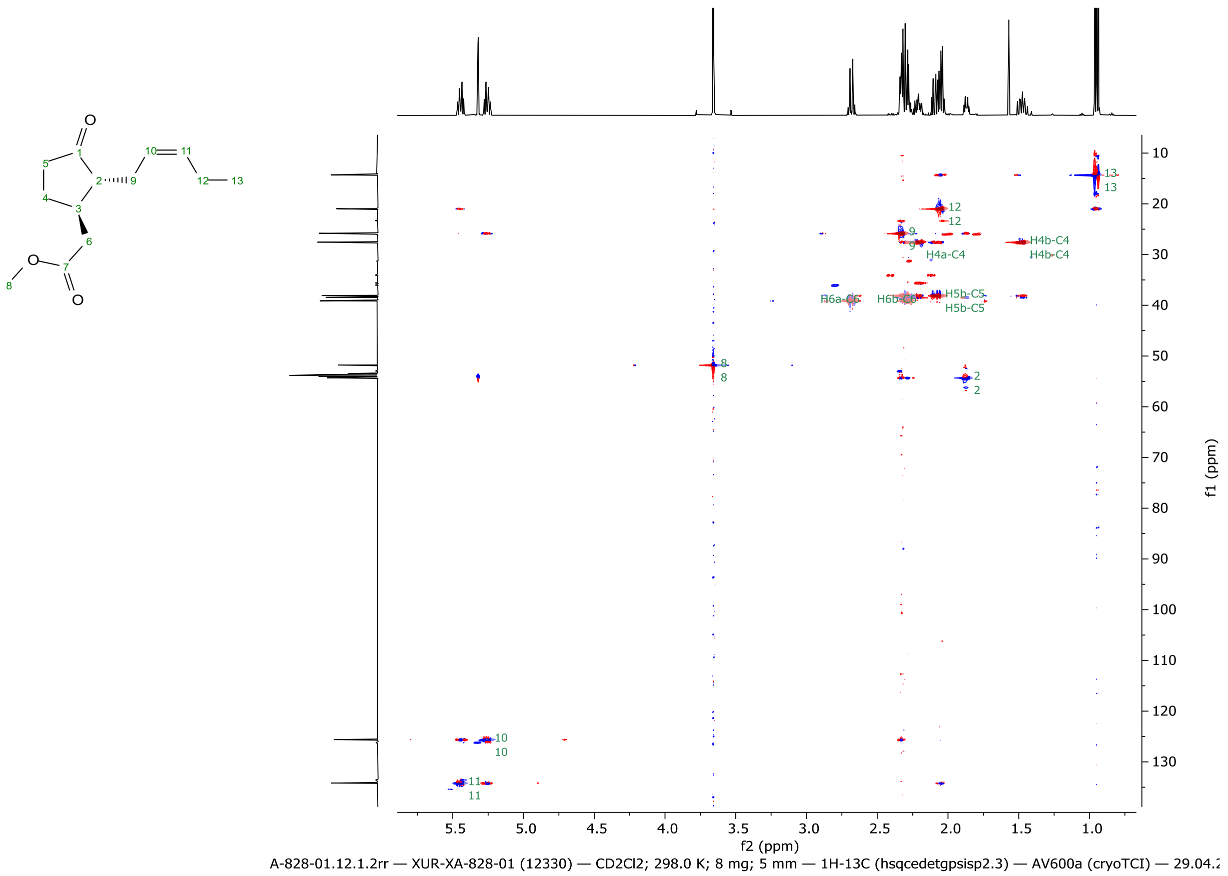

HMBC spectra of 6c

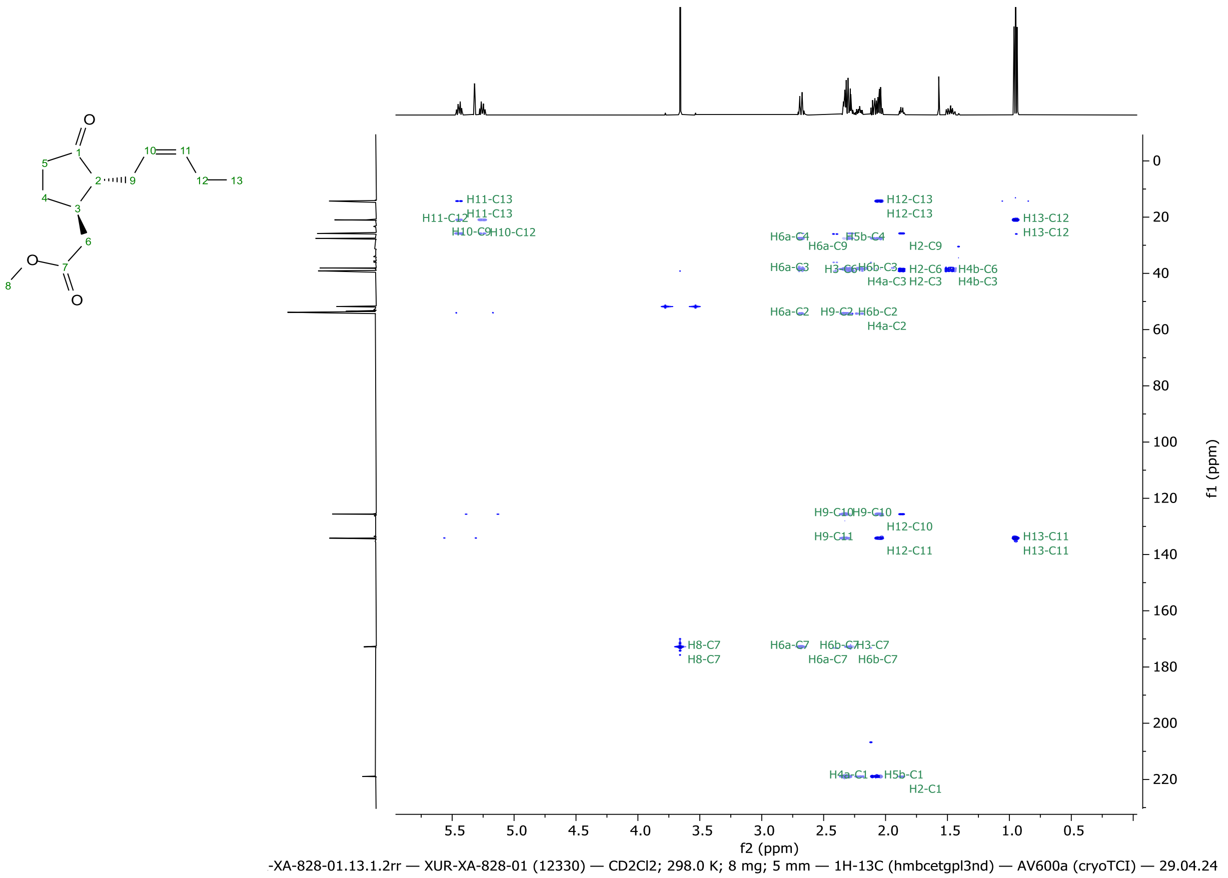

COSY spectra of 6c

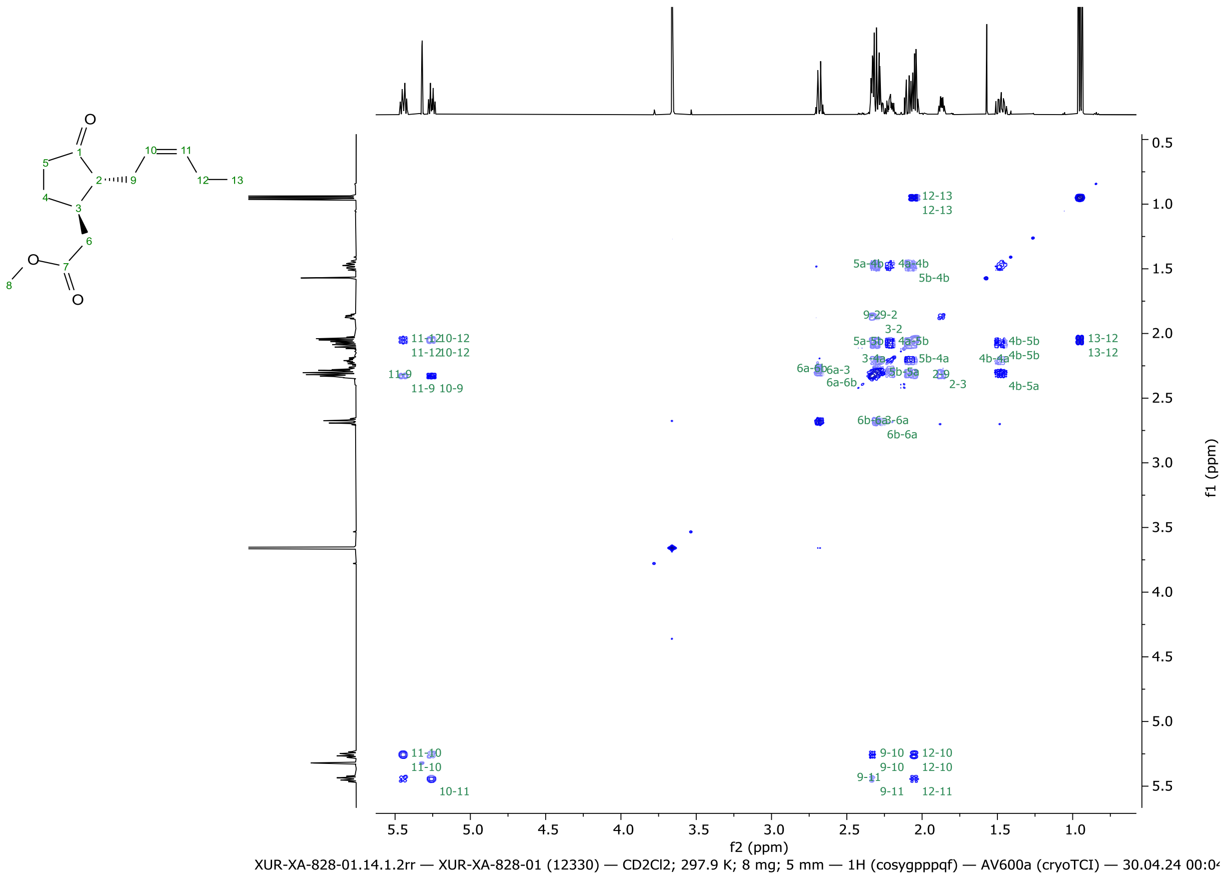

NOESY spectra of 6c

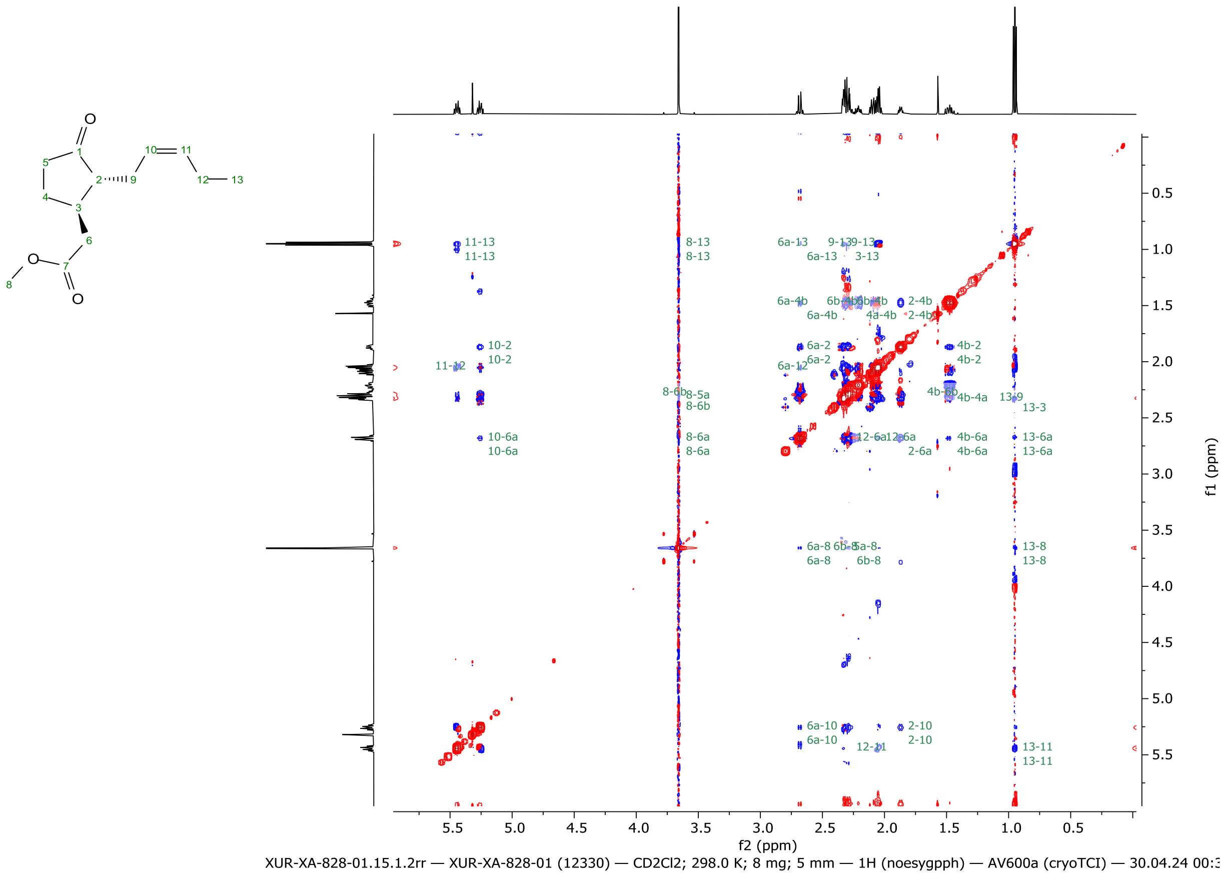

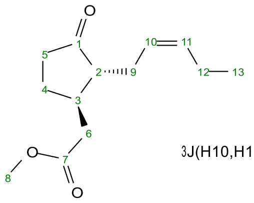

^3J(H10,H11) = 10.8 Hz, cis

major isomer

- δC6 = 39.11 ppm is shielded, in comparison to deshielded δC6 = 34.07 ppm for the major C2,C3 cis compound in XUR-XA-829-01!

- in NOESY: H2 - H4b, H6a, H10
  H3 - H13

Atom	δ (ppm)	J	COSY	HSQC	HMBC	NOESY
1 C	218.93				2, 4a, 5a, 5b, 9	
2 C	54.28			2	4a, 6a, 6b, 9	
H	1.87	10.45(?), 5.66(?), 5.65(?), 1.51(?)	3, 9	2	1, 3, 6, 9	4b, 6a, 10
3 C	38.44			3	2, 4a, 4b, 5a, 6a, 6b	
H	2.30		2, 4a, 6a	3	4, 6, 7	13
4 C	27.56			4a, 4b	3, 5a, 5b, 6a, 6b	
Ha	2.21		3, 4b, 5a, 5b	4	1, 2, 3	4b
Hb	1.47		4a, 5a, 5b	4	3, 6	2, 4a, 6a, 6b
5 C	38.07			5a, 5b		
Ha	2.31		4a, 4b, 5b	5	1, 3, 4	8
Hb	2.07		4a, 4b, 5a	5	1, 4	
6 C	39.11			6a, 6b	2, 3, 4b	
Ha	2.68		3, 6b	6	2, 3, 4, 7, 9	2, 4b, 8, 10, 12, 13
Hb	2.28		6a	6	2, 3, 4, 7, 9	4b, 8
7 C	172.82				3, 6a, 6b, 8	
8 C	51.78			8		
H3	3.66			8	7	5a, 6a, 6b, 13
9 C	25.81			9	2, 6a, 6b, 10, 11	
H2	2.33		2, 10, 11	9	1, 2, 10, 11	13
10 C	125.61			10	9, 12	
H	5.26	10.80(11), 7.48(?), 7.48(?), 1.64(?), 1.64(?)	9, 11, 12	10	9, 12	2, 6a
11 C	134.18			11	9, 12, 13	
H	5.44	10.80(10), 7.24(?), 7.24(?), 1.62(?), 1.62(?)	9, 10, 12	11	9, 12, 13	12, 13
12 C	20.96			12	10, 11, 13	
H2	2.05	7.54(13)	10, 11, 13	12	10, 11, 13	6a, 11
13 C	14.29			13	11, 12	
H3	0.95	7.54(12)	12	13	11, 12	3, 6a, 8, 9, 11

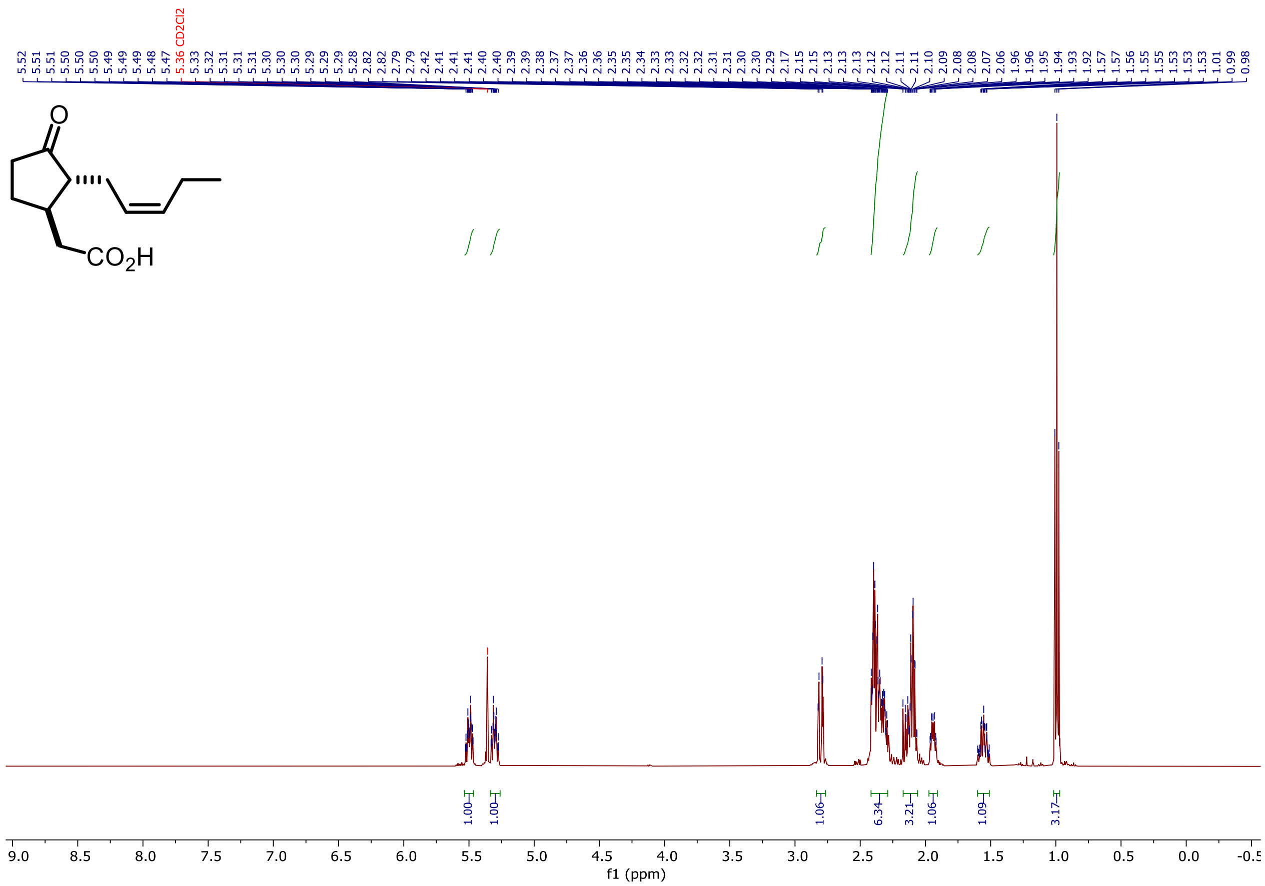
5.52 5.51 5.51 5.50 5.50 5.50 5.49 5.49 5.49 5.48 5.47 5.36 CD2Cl2 5.33 5.32 5.31 5.31 5.31 5.30 5.30 5.29 5.29 5.29 5.28 2.82 2.82 2.79 2.79 2.42 2.41 2.41 2.40 2.40 2.39 2.39 2.38 2.37 2.37 2.36 2.36 2.35 2.35 2.34 2.33 2.33 2.32 2.32 2.31 2.31 2.30 2.30 2.29 2.17 2.15 2.15 2.13 2.13 2.13 2.12 2.12 2.11 2.11 2.10 2.09 2.08 2.08 2.07 2.06 1.96 1.96 1.95 1.94 1.93 1.92 1.57 1.57 1.56 1.55 1.55 1.53 1.53 1.53 1.01 0.99 0.98
O
CO_2H
1.00
1.00
1.06
6.34
3.21
1.06
1.09
3.17
9.0 8.5 8.0 7.5 7.0 6.5 6.0 5.5 5.0 4.5 4.0 3.5 3.0 2.5 2.0 1.5 1.0 0.5 0.0 -0.5
f1 (ppm)

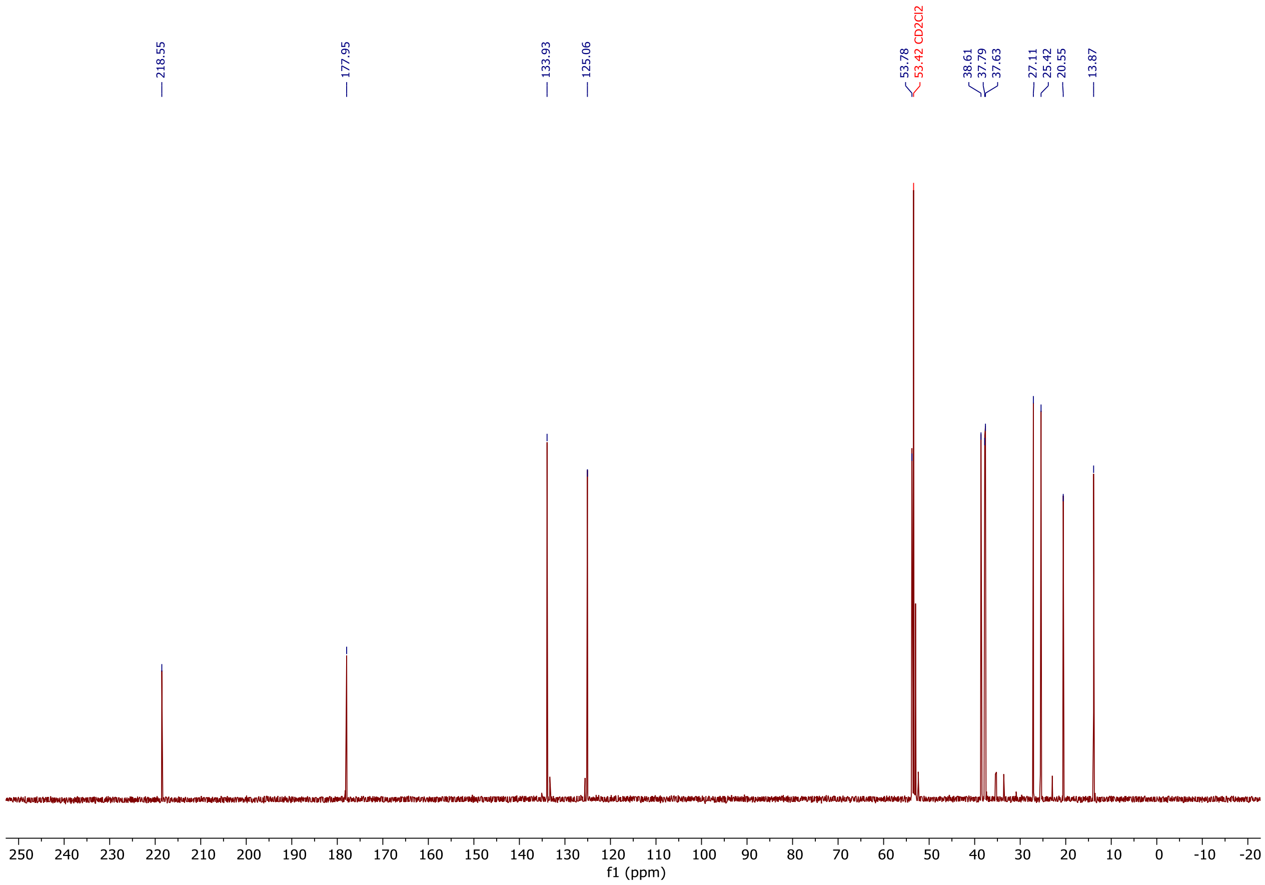
218.55
177.95
133.93
125.06
53.78
53.42 CD2Cl2
38.61
37.79
37.63
27.11
25.42
20.55
13.87
250 240 230 220 210 200 190 180 170 160 150 140 130 120 110 100 90 80 70 60 50 40 30 20 10 0 -10 -20
f1 (ppm)

HSQC spectra of 8

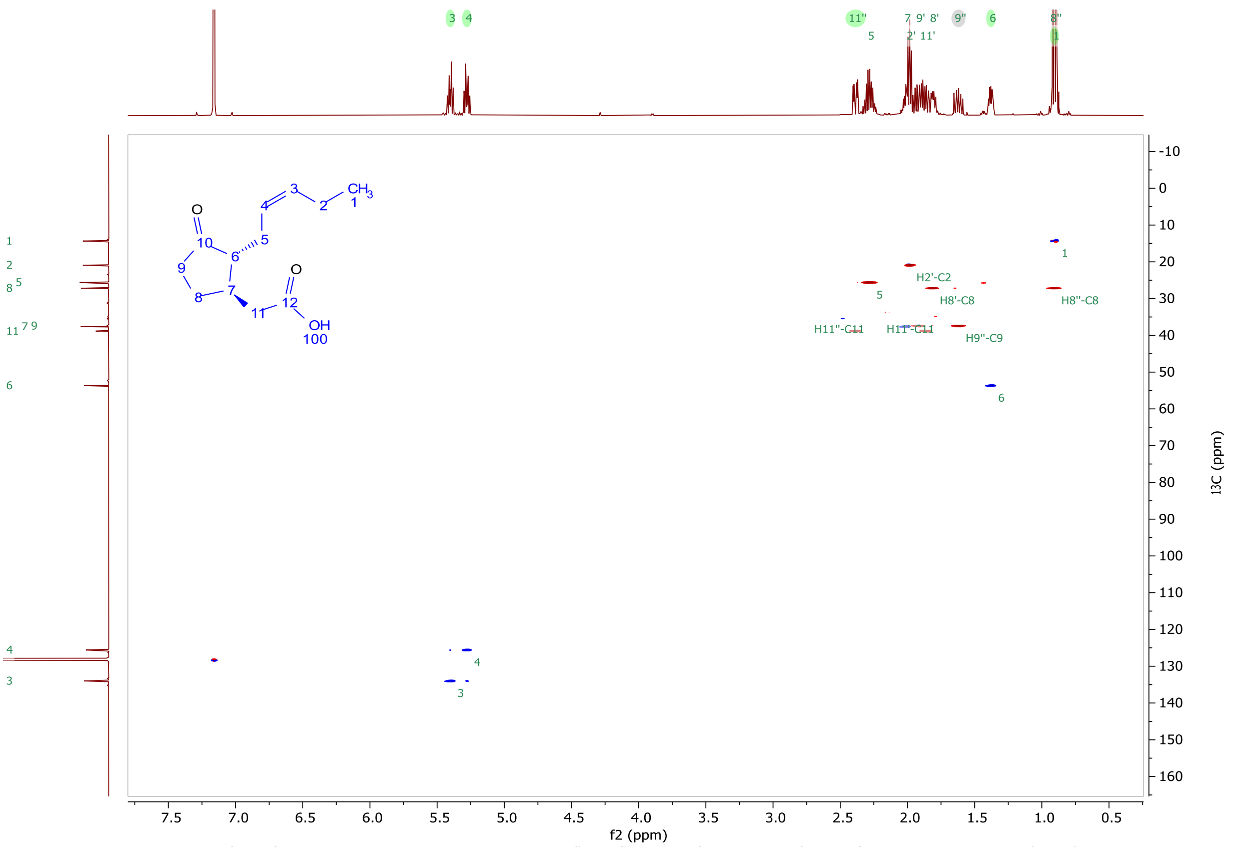

HSQC-EDITED — XUR-XA-827-02 (13956) — C6D6; 298.0 K; 30 mg; 5 mm — 1H-13C (hsqcedetgpsisp2.3) — AV600neo (cryoBBO) — 27.02.25 03:28:23 — hsqcedetgpsisp2.3

COSY spectra of 8

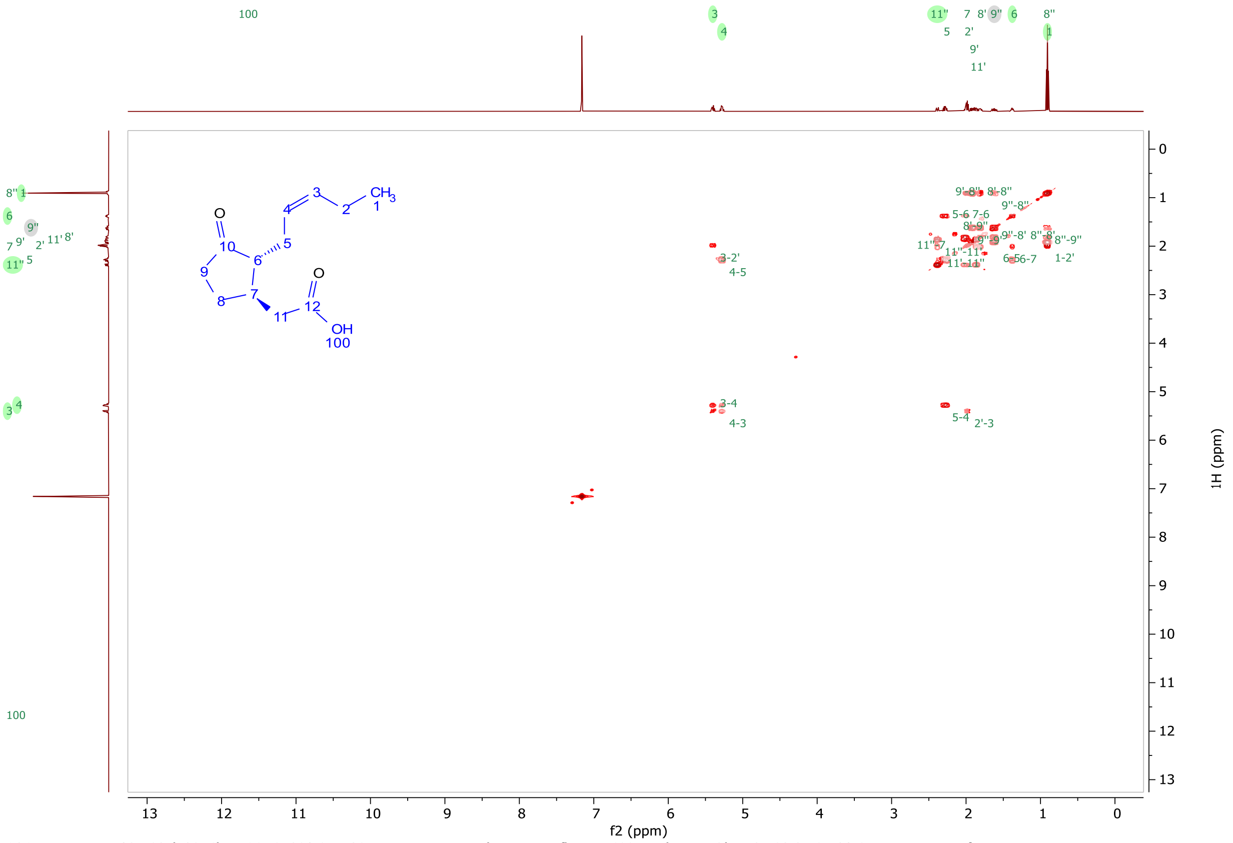

COSY — XUR-XA-827-02 (13956) — C6D6; 298.0 K; 30 mg; 5 mm — 1H (cosygpppqf) — AV600neo (cryoBBO) — 27.02.25 04:38:07 — cosygpppqf

HMBC spectra of 8

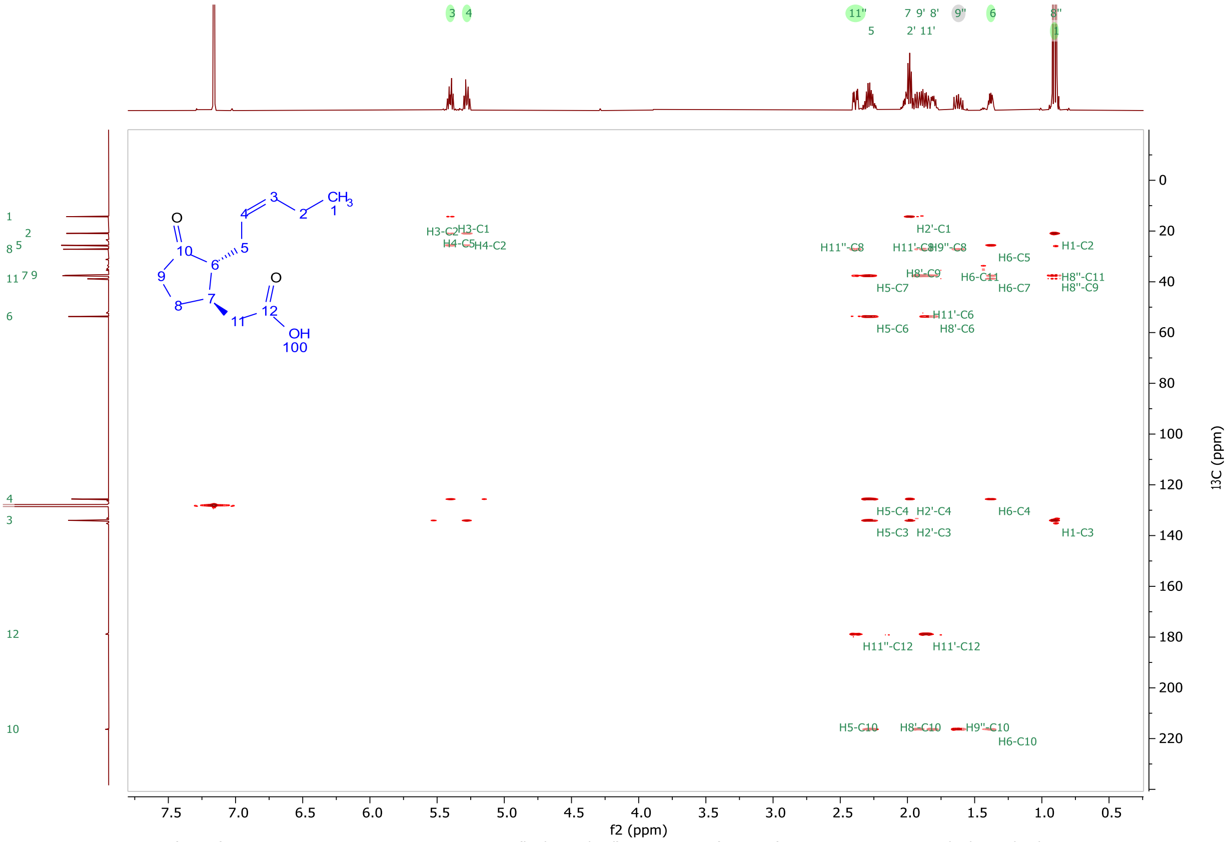

HMBC — XUR-XA-827-02 (13956) — C6D6; 298.0 K; 30 mg; 5 mm — 1H-13C (hmbcetgpl3nd) — AV600neo (cryoBBO) — 27.02.25 04:12:57 — hmbcetgpl3nd

NOESY spectra of 8

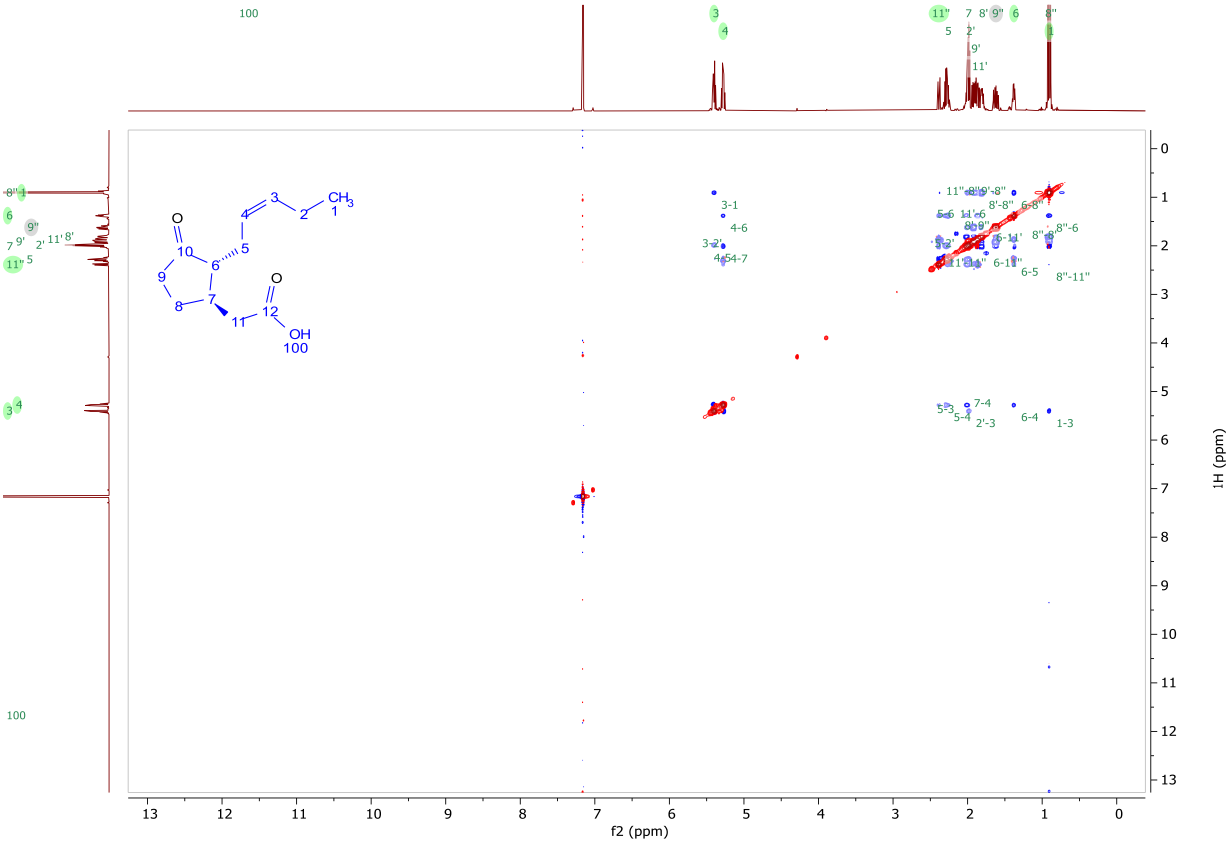

NOESY — XUR-XA-827-02 (13956) — C6D6; 298.0 K; 30 mg; 5 mm — 1H (noesygpphpp) — AV600neo (cryoBBO) — 27.02.25 06:38:56 — noesygpphpp

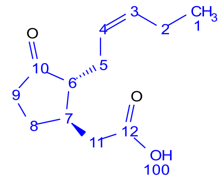

The following NOEs were important to confirm the orientation.
NOE: H6-H11',H11" and H7 - H5

Atom	δ (ppm)	Predicted Shift	J	COSY	HSQC	HMBC	NOESY
1 C	14.336	14.10			1	2', 3	
H3	0.907	0.96	7.50(2'), 7.50(2")	2', 2"	1	2, 3	3
2 C	20.926	22.16			2', 2"	1, 3, 4	
H'	1.987	2.06	7.50(1)	1, 3	2	1, 3, 4	3, 5
H"		2.06	7.50(1)	1	2		
3 C	134.016	134.44			3	1, 2', 5	
H	5.402	5.39	10.80(4)	2', 4	3	1, 2, 5	1, 2', 5
4 C	125.588	128.14			4	2', 5, 6	
H	5.278	5.36	10.80(3)	3, 5	4	2, 5	5, 6, 7
5 C	25.634	27.05			5	3, 4, 6	
H2	2.285	2.15, 2.40		4, 6	5	3, 4, 6, 7, 10	2', 3, 4, 6, 7
6 C	53.677	53.80			6	5, 8', 11'	
H	1.379	2.28		5, 7	6	4, 5, 7, 10, 11	4, 5, 7, 8", 11', 11"
7 C	37.629	38.74			7	5, 6, 9'	
H	2.013	1.89		6, 8", 11', 11"	7		4, 5, 6, 9", 11"
8 C	27.192	27.83			8', 8"	9", 11', 11"	
H'	1.813	1.57, 2.15		8", 9"	8	6, 9, 10	8", 9"
H"	0.909	1.57, 2.15		7, 8', 9', 9"	8	9, 11	6, 8', 9', 11"
9 C	37.433	38.32			9', 9"	8', 8"	
H'	1.916	2.39, 2.71		8", 9"	9	7, 10	8", 9"
H"	1.620	2.39, 2.71		8', 8", 9'	9	8, 10	7, 8', 9'
10 C	216.339	219.08				5, 6, 8', 9', 9"	
11 C	38.842	40.18			11', 11"	6, 8"	
H'	1.867	2.25, 2.46		7, 11"	11	6, 8, 12	6, 11"
H"	2.387	2.25, 2.46		7, 11'	11	8, 12	6, 7, 8", 11'
12 C	178.881	178.53				11', 11"	
100 O							
H	11.674	10.98					

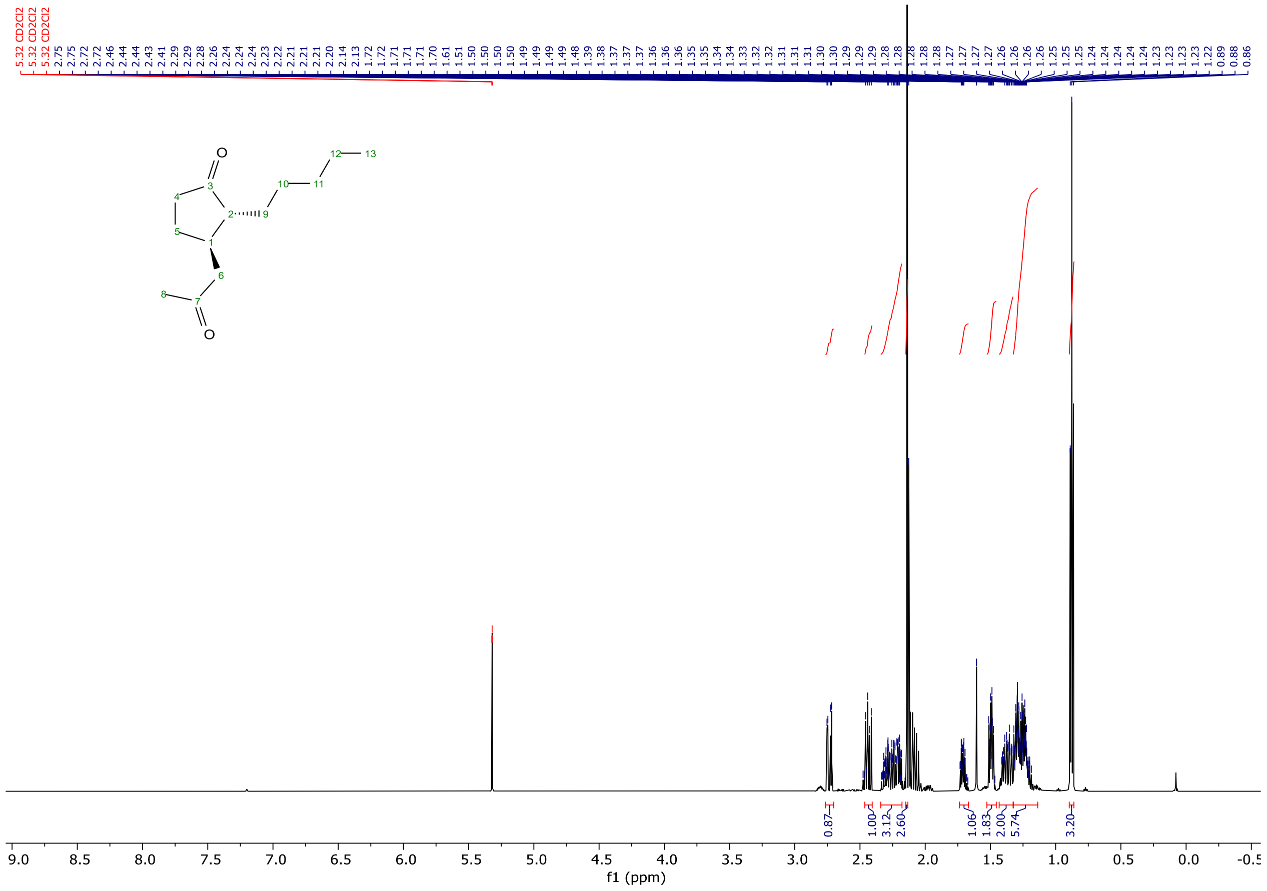

5.32 CD2Cl2
0.87
1.00
3.12
2.60
1.06
1.83
2.00
5.74
3.20
f1 (ppm)

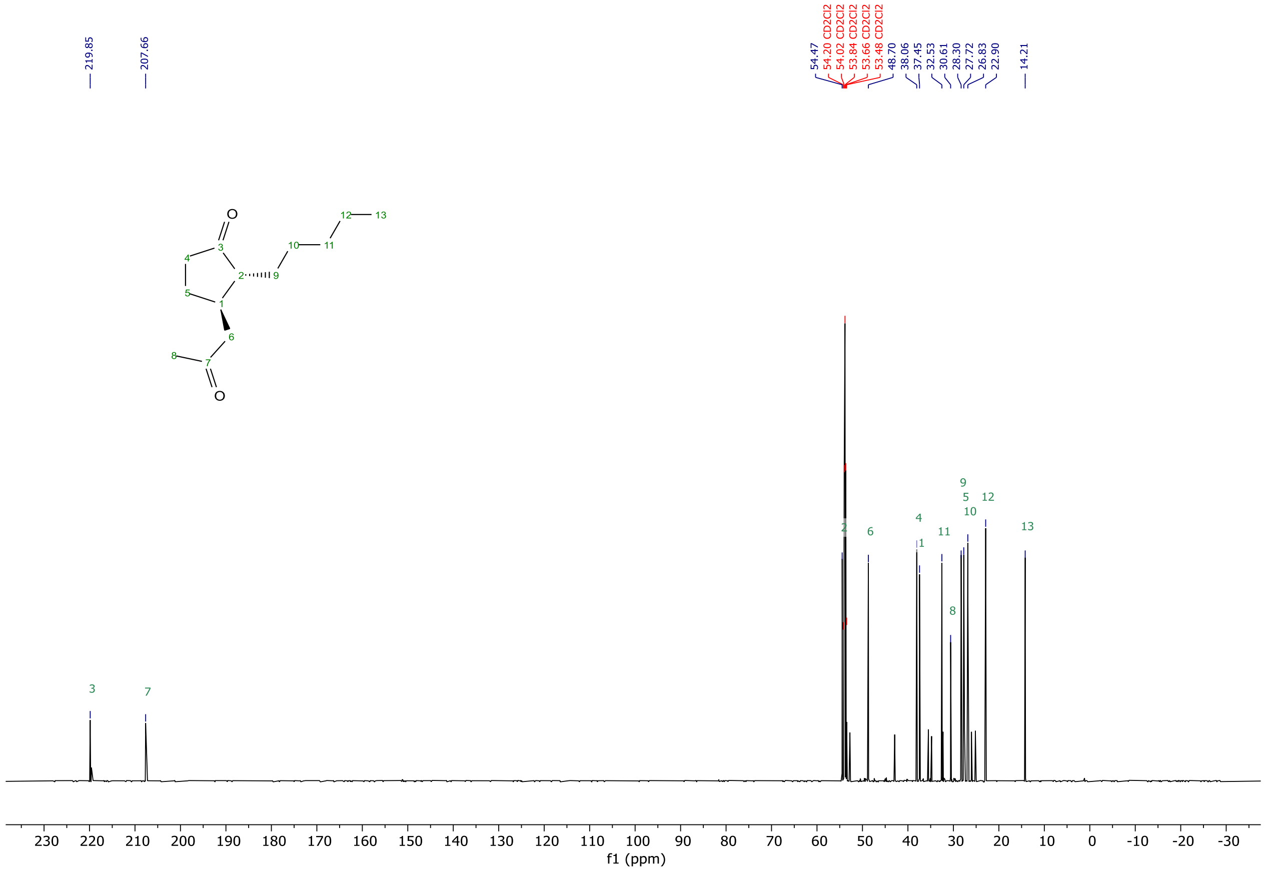

219.85
207.66
54.47
54.20 CD2Cl2
54.02 CD2Cl2
53.84 CD2Cl2
53.66 CD2Cl2
53.48 CD2Cl2
48.70
38.06
37.45
32.53
30.61
28.30
27.72
26.83
22.90
14.21
f1 (ppm)

HSQC spectra of 6b

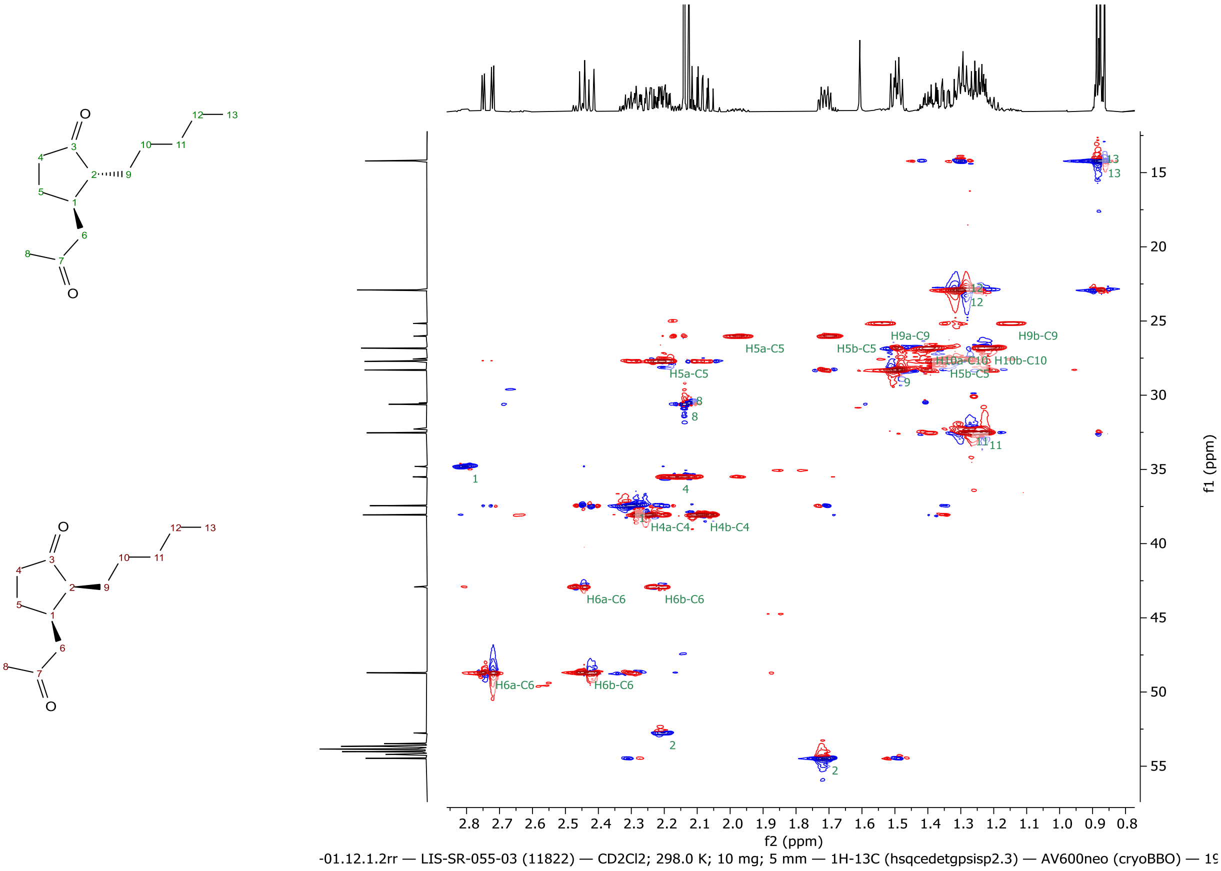

HMBC spectra of 6b

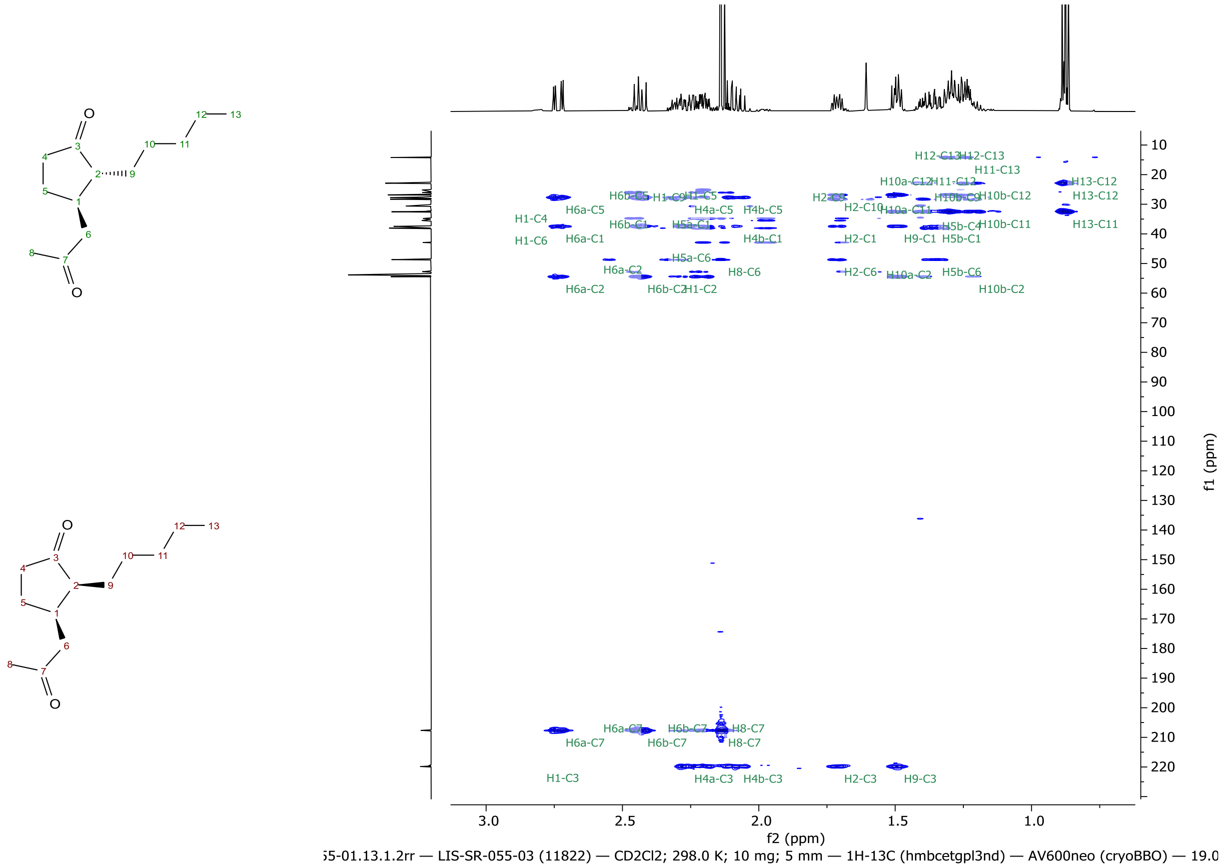

COSY spectra of 6b

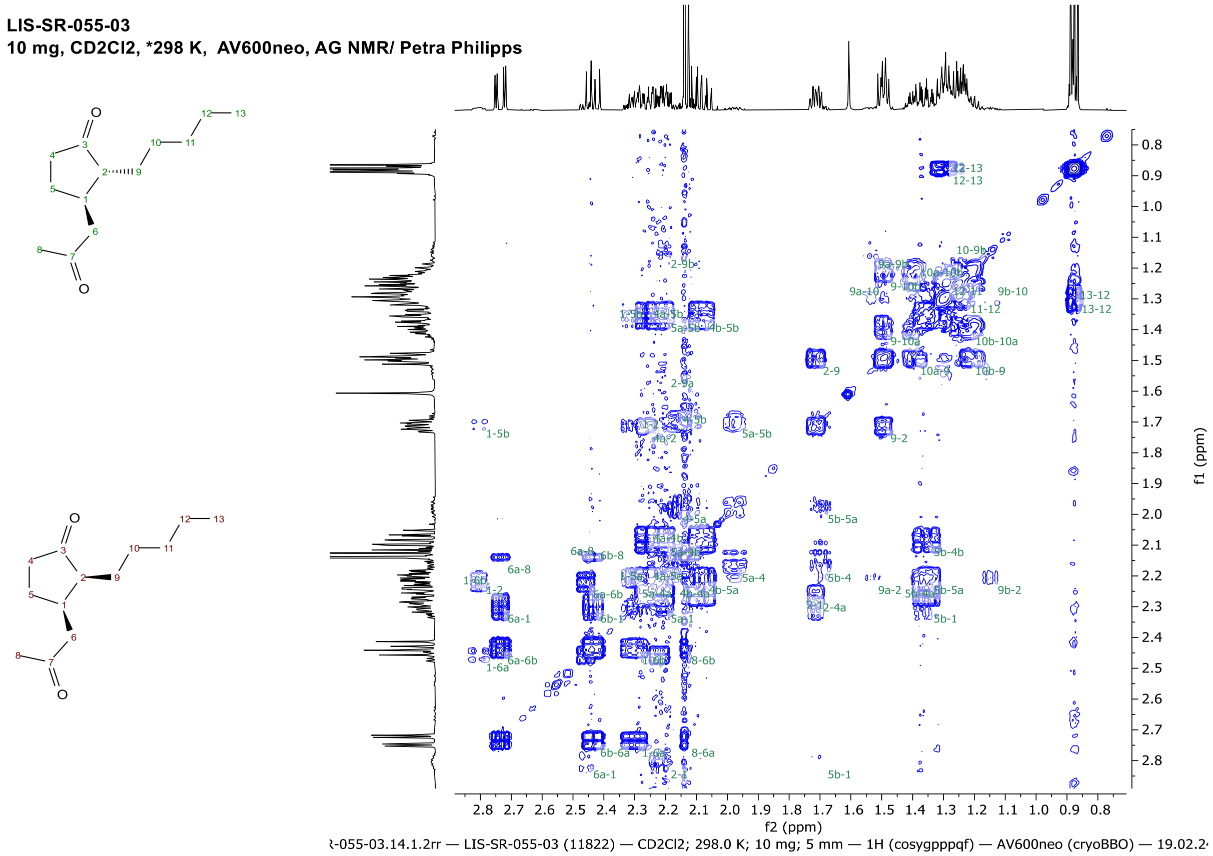

NOESY spectra of 6b

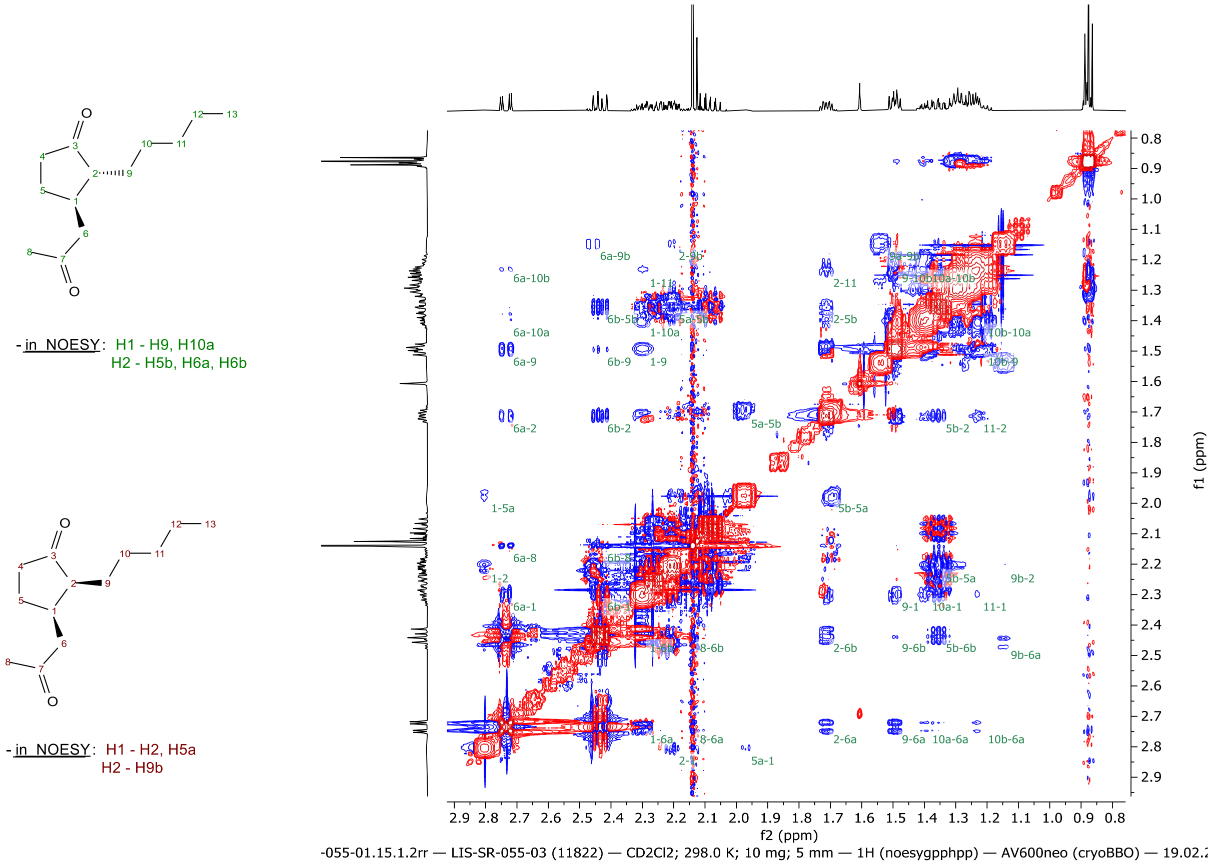

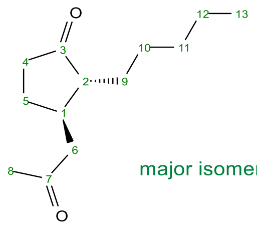

major isomer, trans

ratio ~ 4.5 trans : 1 cis

- δC6 = 48.70 ppm, downfield !

- in NOESY: H1 - H9, H10a
H2 - H5b, H6a, H6b

Atom	δ (ppm)	J	COSY	HSQC	HMBC	NOESY
1 C	37.45			1	2, 4a, 4b, 5a, 5b, 6a, 6b, 9	
H	2.30	4.14(6a), 9.21(6b)	2, 5a, 5b, 6a, 6b	1	2, 5, 6, 9	6a, 6b, 9, 10a, 11
2 C	54.47			2	1, 5a, 6a, 6b, 9, 10a, 10b	
H	1.71	10.59(?), 5.35(?), 5.18(?), 1.39(?)	1, 4a, 9	2	1, 3, 6, 9, 10	5b, 6a, 6b, 11
3 C	219.85				2, 4a, 4b, 9	
4 C	38.06			4a, 4b	5a, 5b	
Ha	2.26		2, 4b, 5a, 5b	4	1, 3, 5	
Hb	2.08	18.49(?), 10.95(?), 8.79(?)	4a, 5a, 5b	4	1, 3, 5	
5 C	27.72			5a, 5b	1, 4a, 4b, 6a, 6b	
Ha	2.21	12.53(?), 8.67(?), 6.16(?), 2.35(?)	1, 4a, 4b, 5b	5	1, 2, 4, 6	5b
Hb	1.35		1, 4a, 4b, 5a	5	1, 4, 6	2, 5a, 6b
6 C	48.70			6a, 6b	1, 2, 5a, 5b, 8	
Ha	2.74	4.14(1), 16.92(6b)	1, 6b, 8	6	1, 2, 5, 7	1, 2, 8, 9, 10a, 10b
Hb	2.44	9.21(1), 16.92(6a)	1, 6a, 8	6	1, 2, 5, 7	1, 2, 5b, 8, 9
7 C	207.66				6a, 6b, 8	
8 C	30.61			8		
H3	2.14		6a, 6b	8	6, 7	6a, 6b
9 C	28.30			9	1, 2, 10a, 10b, 11	
H2	1.49	9.36(?), 6.69(?), 5.53(?)	2, 10a, 10b	9	1, 2, 3, 10, 11	1, 6a, 6b, 10b
10 C	26.83			10a, 10b	2, 9, 11, 12	
Ha	1.40		9, 10b	10	2, 9, 11, 12	1, 6a, 10b
Hb	1.22		9, 10a	10	2, 9, 11, 12	6a, 9, 10a
11 C	32.53			11	9, 10a, 10b, 11, 12, 13	
H2	1.24		12	11	9, 10, 11, 12, 13	1, 2
12 C	22.90			12	10a, 10b, 11, 13	
H2	1.29	7.18(13)	11, 13	12	10, 11, 13	
13 C	14.21			13	11, 12	
H3	0.88	7.18(12)	12	13	11, 12	

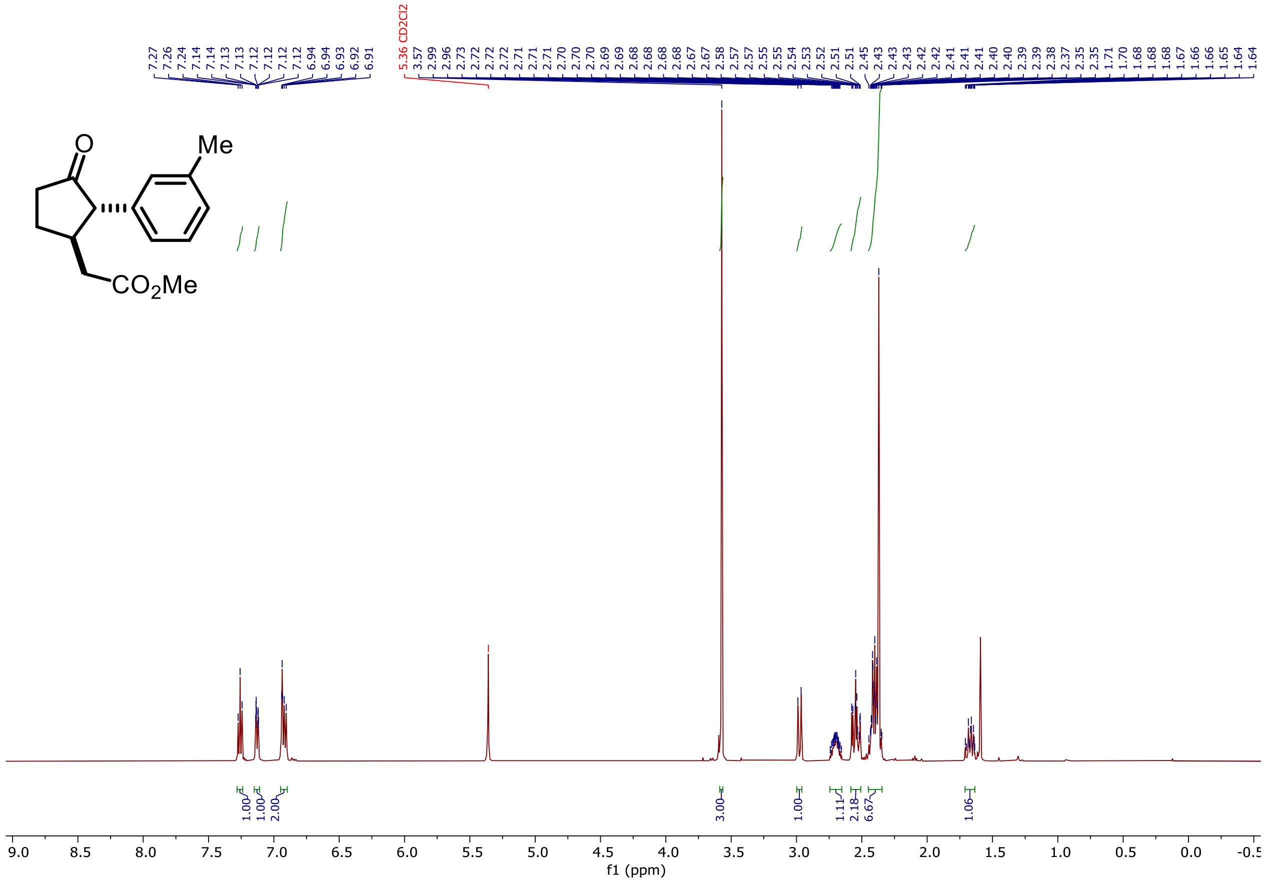

O
Me
CO2Me
7.27 7.26 7.24 7.14 7.14 7.13 7.13 7.12 7.12 7.12 7.12 6.94 6.94 6.93 6.92 6.91
5.36 CD2Cl2
3.57 2.99 2.96 2.73 2.72 2.72 2.72 2.71 2.71 2.71 2.70 2.70 2.70 2.69 2.69 2.68 2.68 2.68 2.68 2.67 2.67 2.58 2.57 2.57 2.55 2.55 2.54 2.53 2.52 2.51 2.51 2.45 2.43 2.43 2.43 2.42 2.42 2.42 2.41 2.41 2.41 2.40 2.40 2.39 2.39 2.38 2.37 2.35 2.35 1.71 1.70 1.68 1.68 1.68 1.67 1.66 1.66 1.65 1.64 1.64
1.00 1.00 2.00 3.00 1.00 1.11 2.18 6.67 1.06
9.0 8.5 8.0 7.5 7.0 6.5 6.0 5.5 5.0 4.5 4.0 3.5 3.0 2.5 2.0 1.5 1.0 0.5 0.0 -0.5
f1 (ppm)

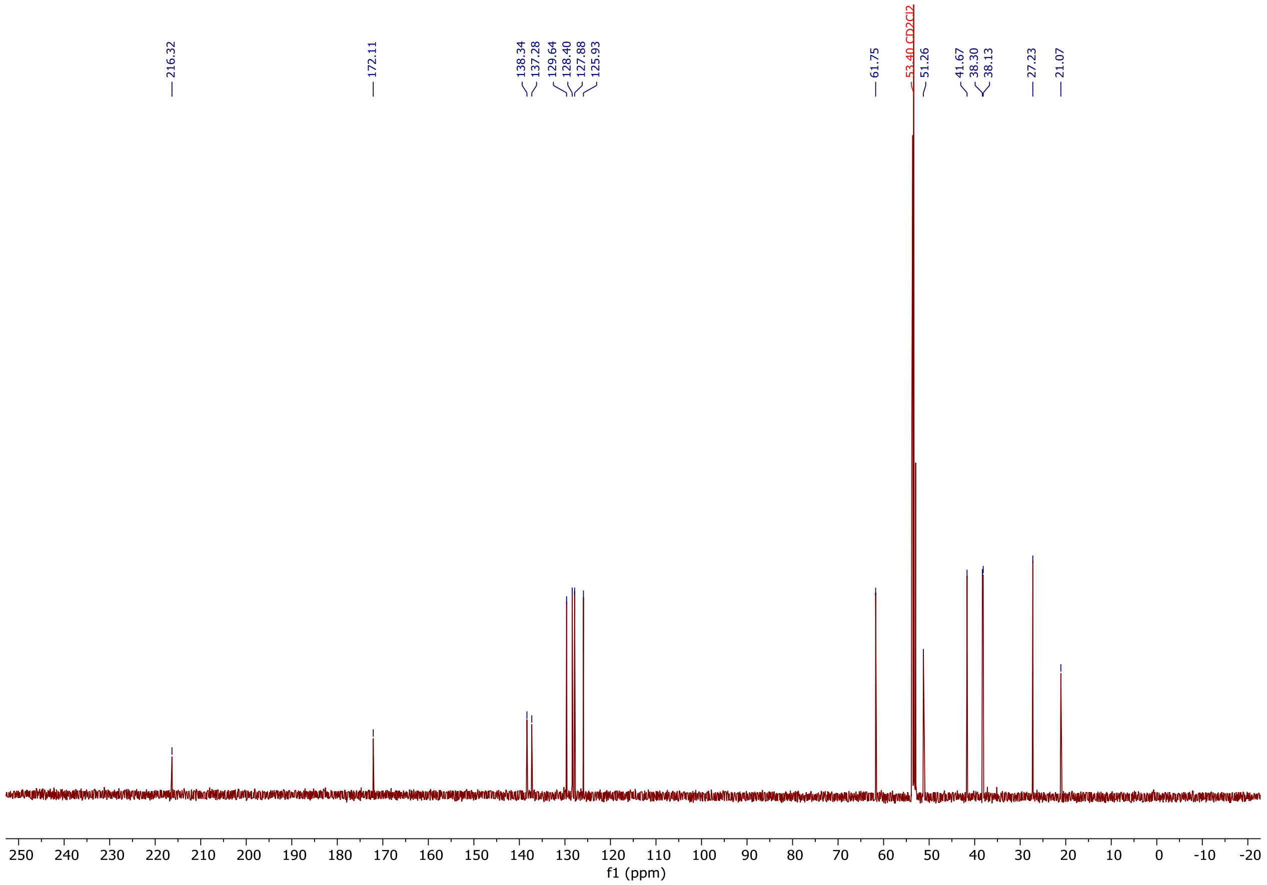

216.32
172.11
138.34 137.28 129.64 128.40 127.88 125.93
61.75
53.40 CD2Cl2
51.26
41.67 38.30 38.13
27.23
21.07
250 240 230 220 210 200 190 180 170 160 150 140 130 120 110 100 90 80 70 60 50 40 30 20 10 0 -10 -20
f1 (ppm)

HSQC spectra of 6d

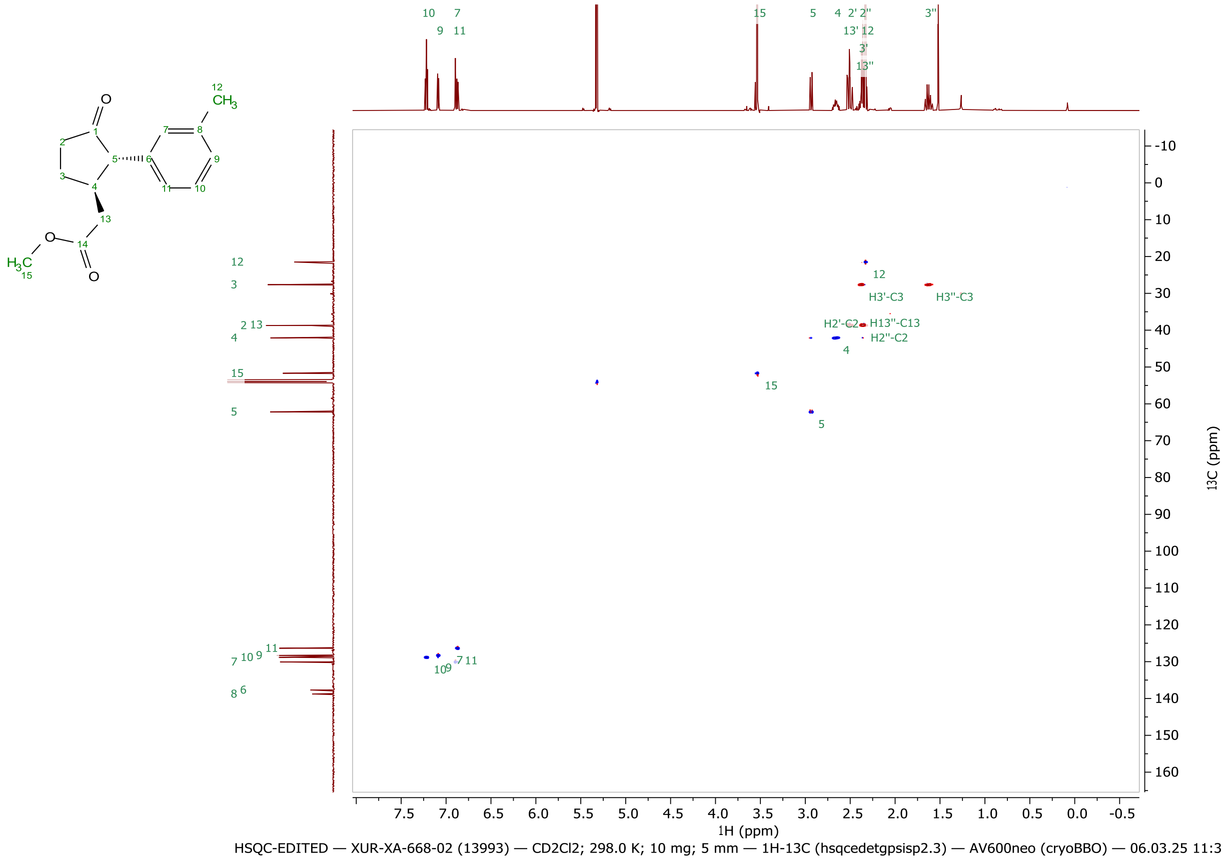

HMBC spectra of 6d

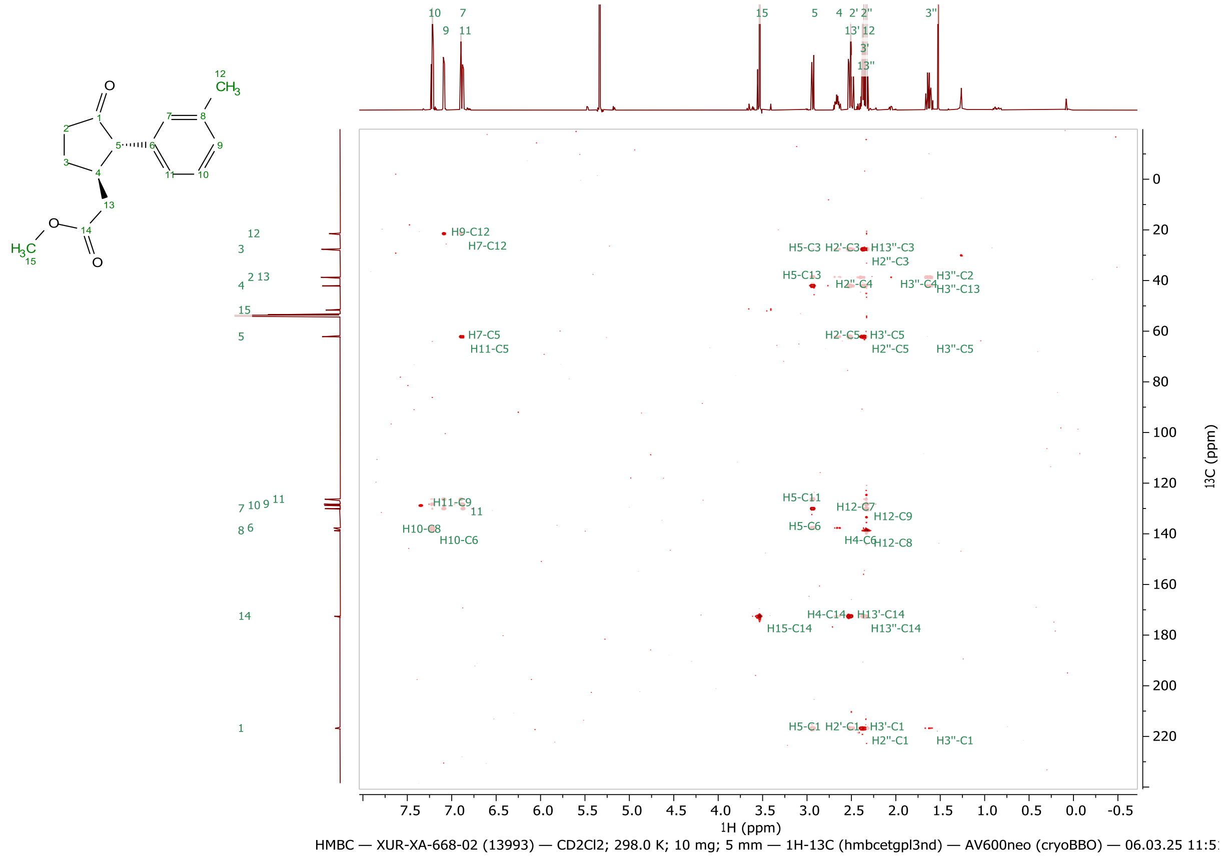

COSY spectra of 6d

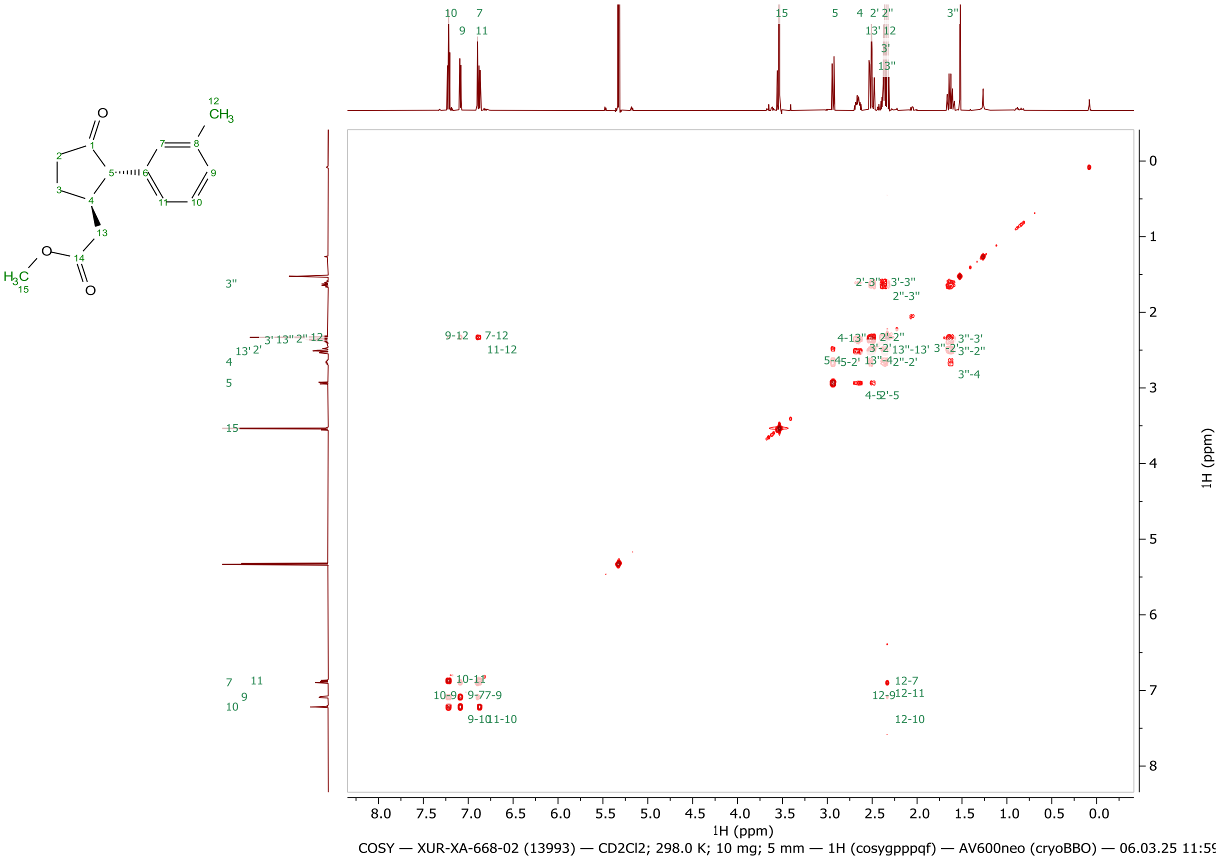

NOESY spectra of 6d

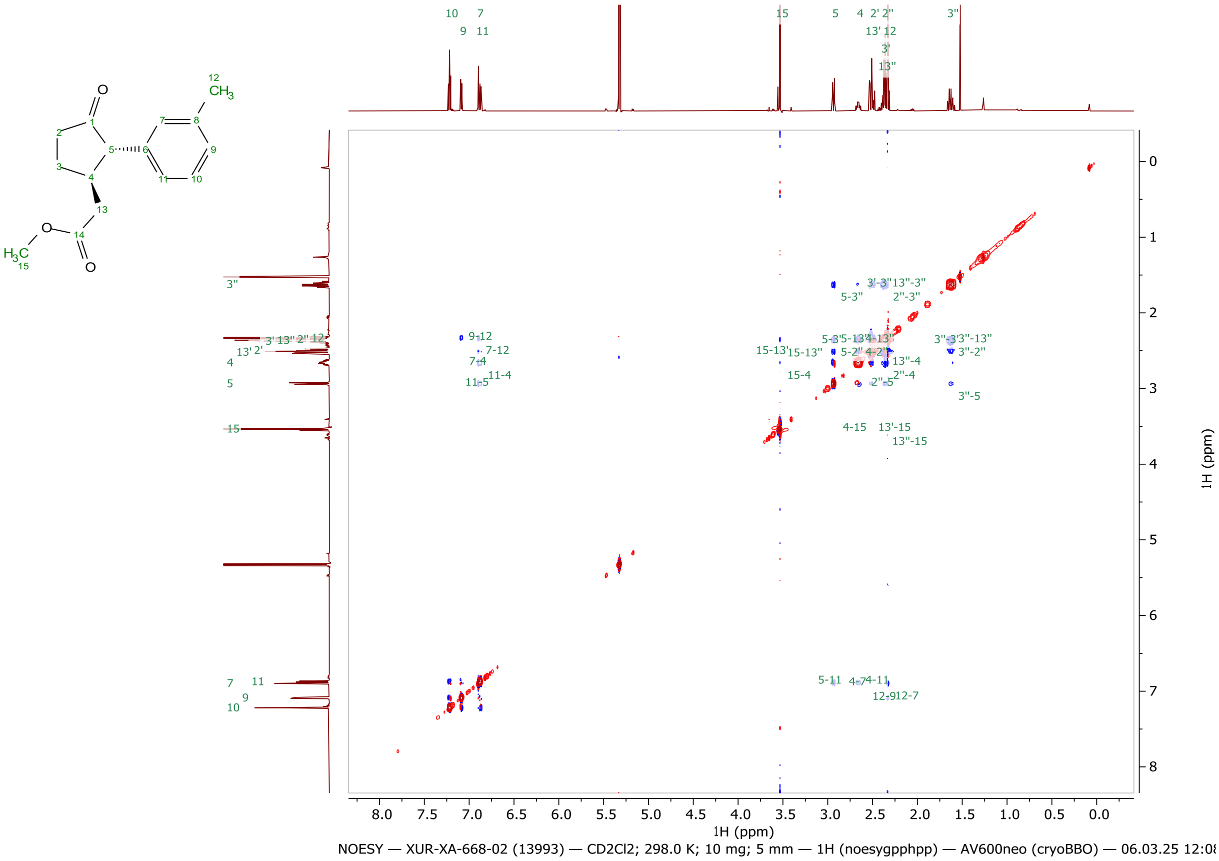

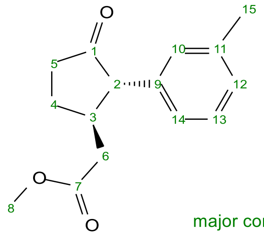

major compound, trans

- major trans isomer, δ C6 = 38.53 ppm !

- due to aromatic ring current caused by the phenyl ring, the proton signals of H6ab get shielded, δ H6ab = 2.51, 2.36 ppm, the signal of H3 deshielded, δ H3 = 2.66 ppm! (consistent to cis isomer in -660-01!)

- <u>in NOESY</u>:

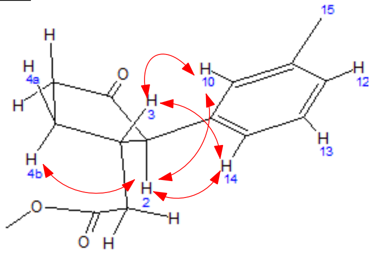

Atom	δ (ppm)	J	COSY	HSQC	HMBC	NOESY/EXSY
1 C	216.82				2, 4a, 5a, 5b	
2 C	62.18			2	5a, 5b, 6a, 6b, 14	
H	2.94	12.37(?), 1.30(?)	3, 5a, 6a	2	1, 3, 6, 9, 10, 14	4b, 5a, 5b, 6a, 6b, 10, 14
3 C	42.09			3	2, 4b, 6a, 6b	
H	2.66		2, 5a, 5b, 6a	3		10, 14
4 C	27.65			4a, 4b	5a, 5b, 6a, 6b	
Ha	2.37			4	1, 5	
Hb	1.63		5a, 5b, 6a, 6b	4	3, 5	2
5 C	38.73			5a, 5b	4a, 4b	
Ha	2.51		2, 3, 4b, 5b	5	1, 2, 4	2, 10, 14
Hb	2.36		3, 4b, 5a	5	1, 2, 4	2, 10, 14
6 C	38.53			6a, 6b	2	
Ha	2.51		2, 3, 4b	6	2, 3, 4, 7	2, 10, 14
Hb	2.36		4b	6	2, 3, 4, 7	2, 8, 10, 14
7 C	172.55				6a, 6b, 8	
8 C	51.70			8		
H3	3.53			8	7	6b, 15
9 C	137.68				2, 13	
10 C	130.06			10	2, 12, 14, 15	
H	6.90		12, 15	10	12, 14, 15	2, 3, 5a, 5b, 6a, 6b, 15
11 C	138.76				13, 15	
12 C	128.30			12	10, 13, 14, 15	
H	7.09	7.78(?)	10, 13, 15	12	10, 13, 14, 15	15
13 C	128.82			13	12, 14	
H	7.22	7.52(?), 7.52(?)	12, 14, 15	13	9, 11, 12	
14 C	126.35			14	2, 10, 12	
H	6.87	7.71(?)	13, 15	14	2, 10, 12, 13	2, 3, 5a, 5b, 6a, 6b
15 C	21.49			15	10, 12	
H3	2.33	0.76(?), 0.67(?), 0.67(?)	10, 12, 13, 14	15	10, 11, 12	8, 10, 12

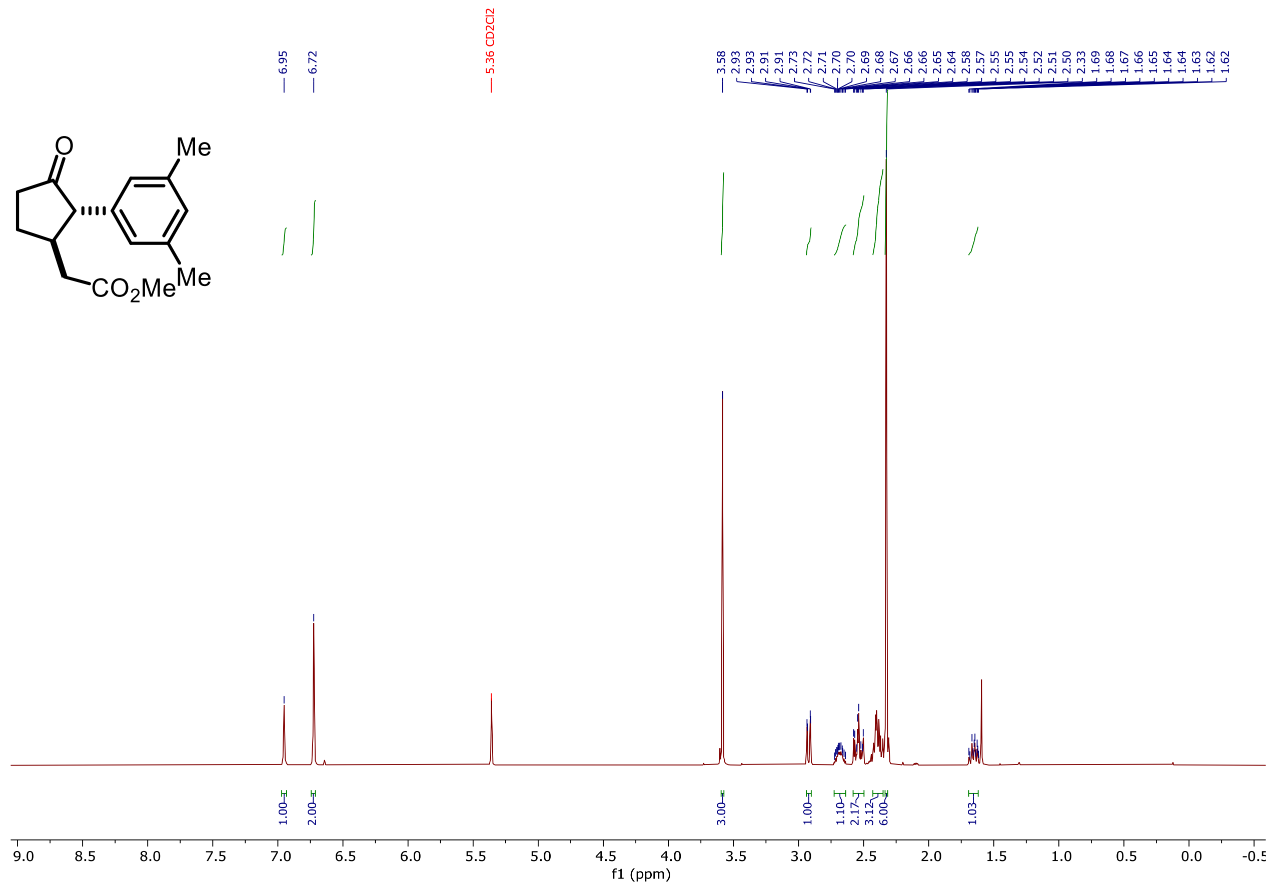

O
Me
Me
CO_2Me
6.95
6.72
5.36 CD2Cl2
3.58
2.93
2.93
2.91
2.91
2.73
2.72
2.71
2.70
2.70
2.69
2.68
2.67
2.66
2.66
2.65
2.64
2.58
2.57
2.55
2.55
2.54
2.52
2.51
2.50
2.33
1.69
1.68
1.67
1.66
1.65
1.64
1.64
1.63
1.62
1.62
1.00
2.00
3.00
1.00
1.10
2.17
3.12
6.00
1.03
f1 (ppm)

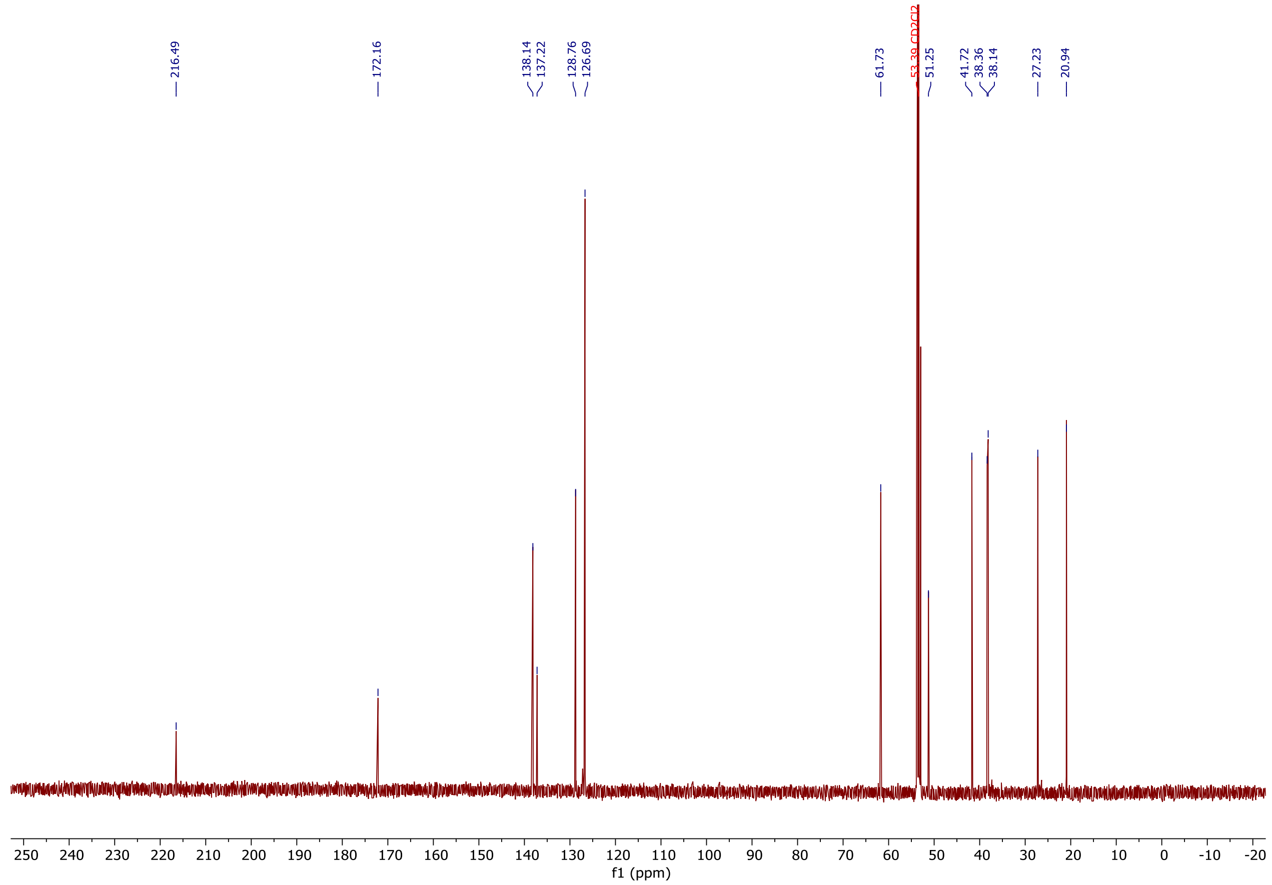

216.49
172.16
138.14
137.22
128.76
126.69
61.73
53.39 CD2Cl2
51.25
41.72
38.36
38.14
27.23
20.94
f1 (ppm)

HSQC spectra of 6e

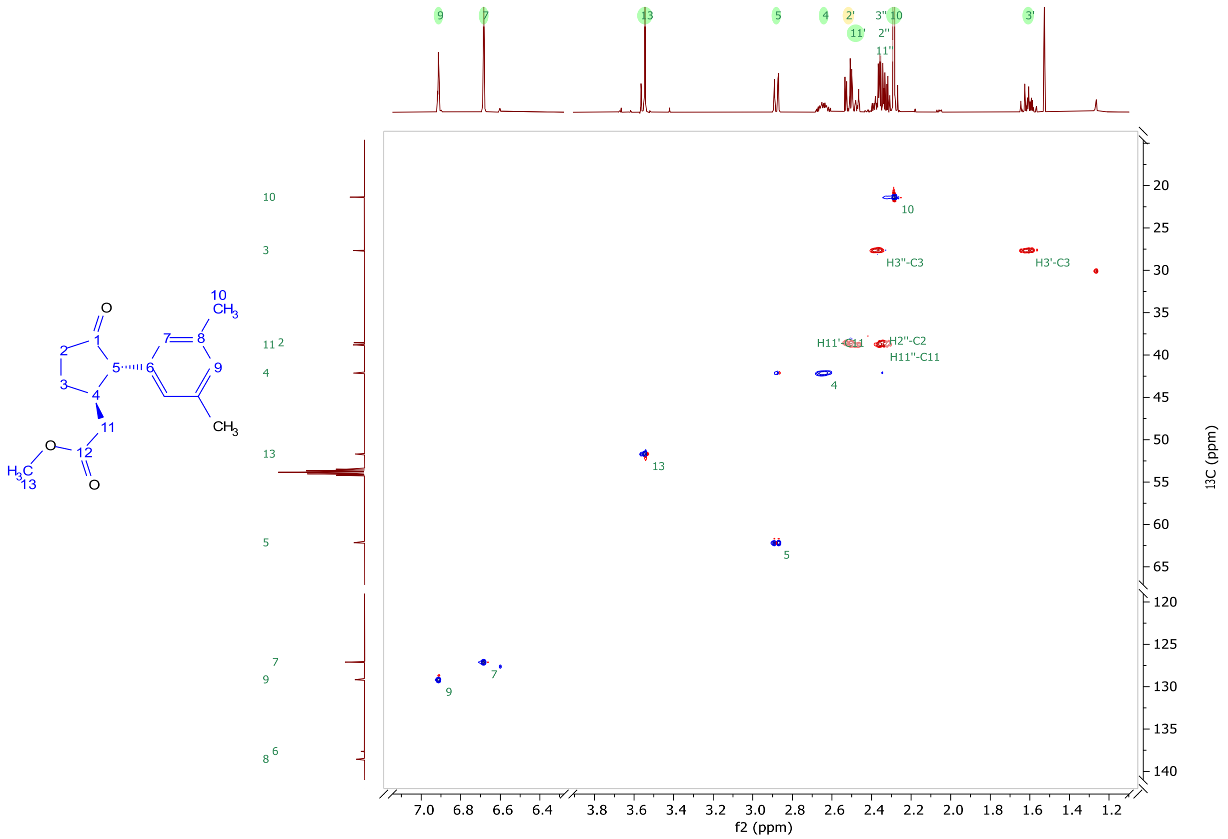

COSY spectra of 6e

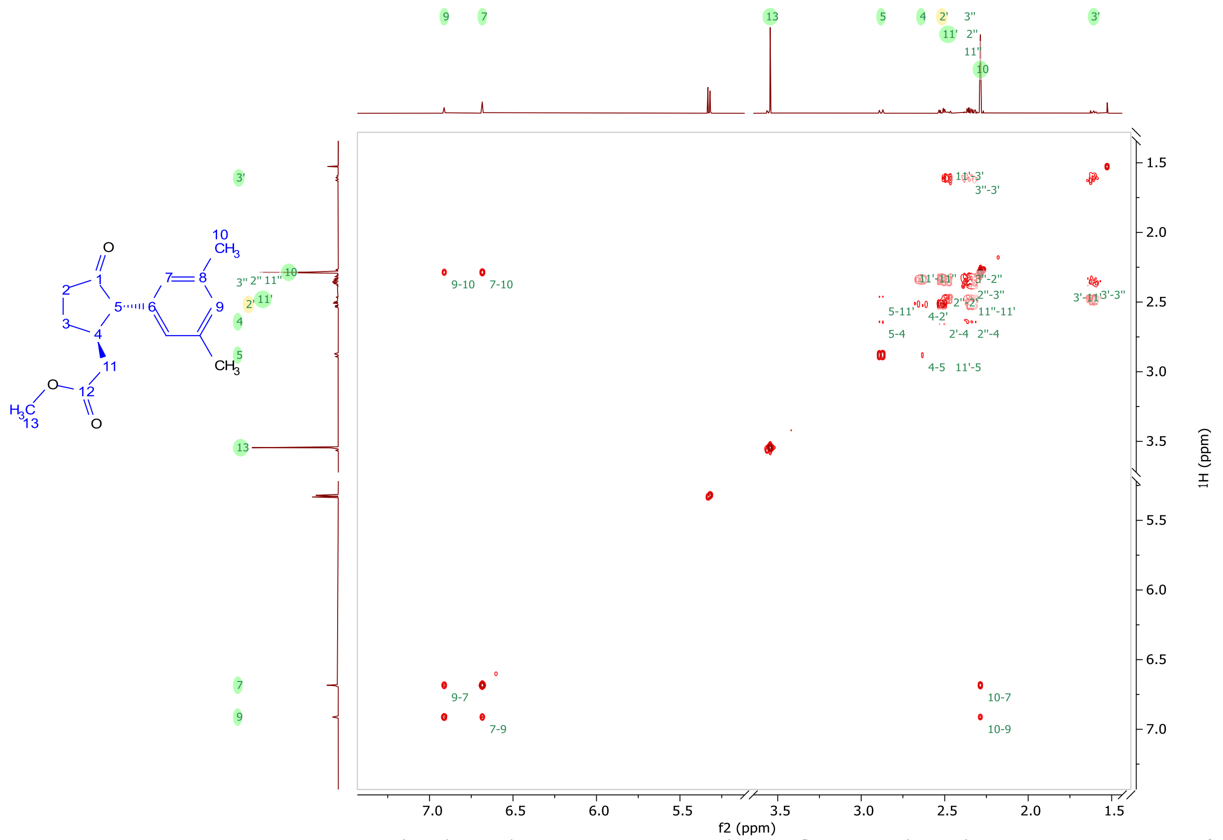

HMBC spectra of 6e

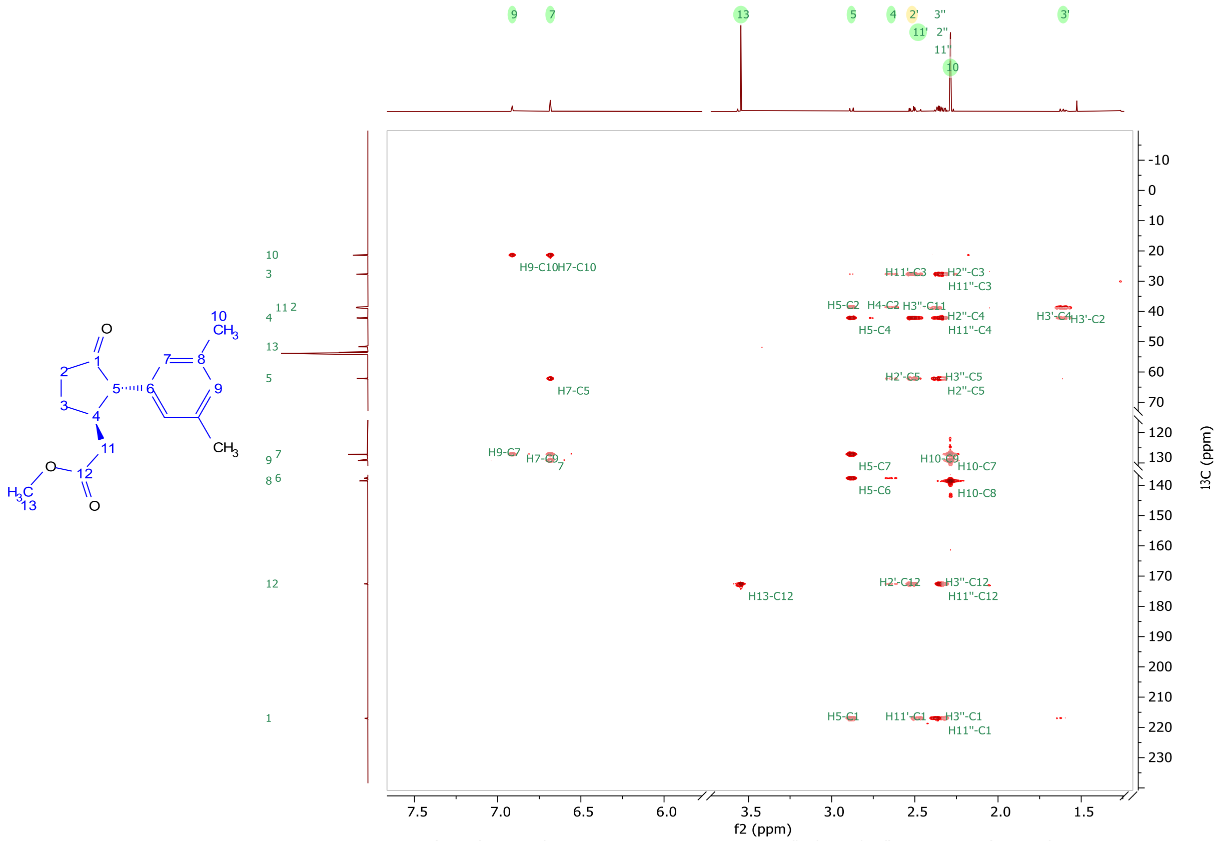

HMBC — XUR-XA-660-02 (13992) — CD2Cl2; 298.0 K; 10 mg; 5 mm — 1H-13C (hmbcetgpl3nd) — AV600neo (cryoBBO) — 06.03.25 09:21:05 —

NOESY spectra of 6e

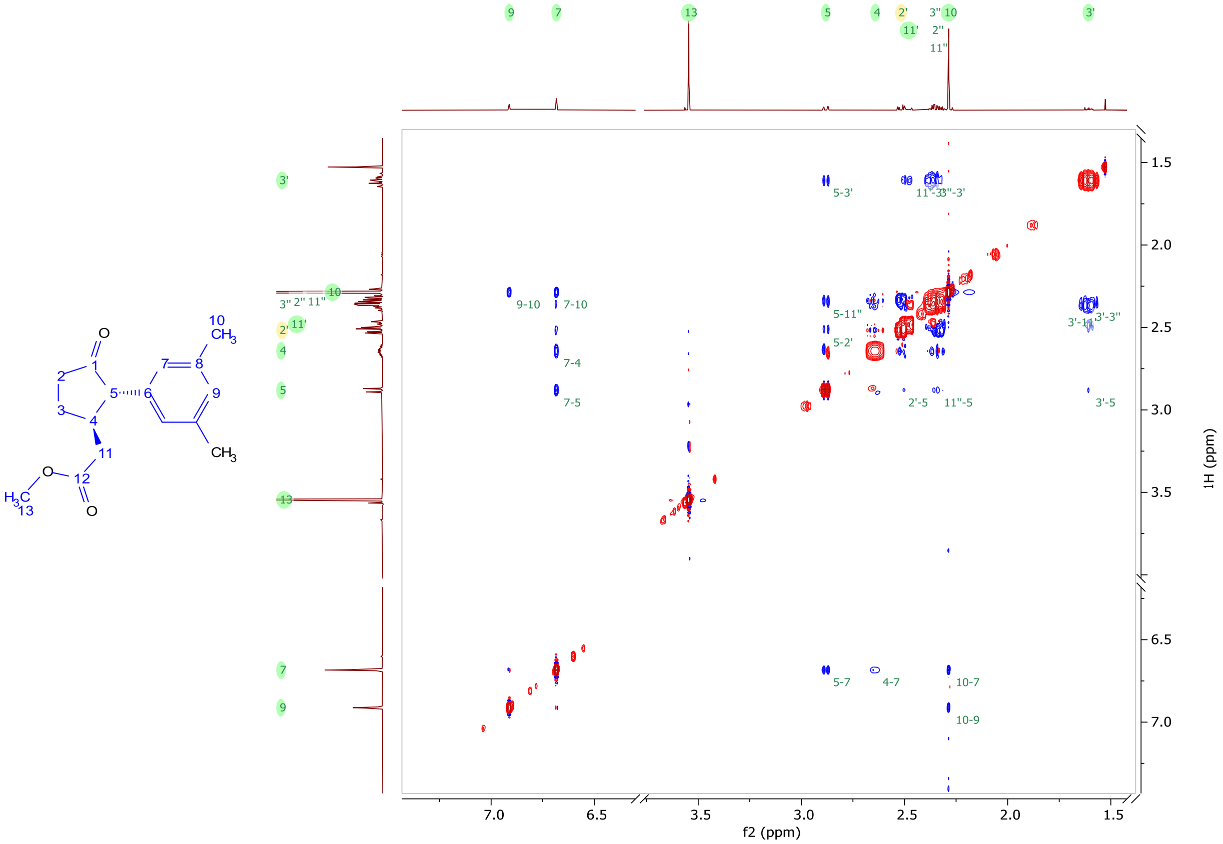

NOESY — XUR-XA-660-02 (13992) — CD2Cl2; 298.0 K; 10 mg; 5 mm — 1H (noesygpphpp) — AV600neo (cryoBBO) — 06.03.25 09:47:46 — n

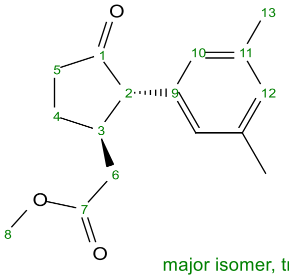

major isomer, trans

- major trans isomer, δ C6 = 38.54 ppm !

- due to aromatic ring current caused by the phenyl ring, the proton signals of H6ab get shielded, δ H6ab = 2.52, 2.34 ppm, the signal of H3 deshielded, δ H3 = 2.65 ppm!
(in comparison to the cis isomer !)

- in NOESY:

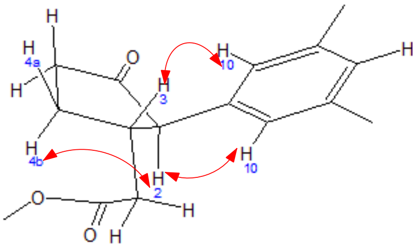

Atom	δ (ppm)	J	COSY	HSQC	HMBC	NOESY/EXSY
1 C	216.99				2, 4a, 4b, 5a, 5b	
2 C	62.15			2	4a, 4b, 6a, 6b, 10	
H	2.88	12.29(?), 1.34(?)	3, 5a, 5b, 6a, 10	2	1, 3, 4, 6, 9, 10	4b, 5a, 5b, 6a, 10
3 C	42.13			3	2, 3, 4b, 5a, 5b, 6a, 6b	
H	2.65		2, 4a, 5a, 6a, 6b	3	3, 4, 6	10
4 C	27.65			4a, 4b	2, 3, 5a, 5b, 6a, 6b	
Ha	2.36		3	4	1, 2, 5	8
Hb	1.61		5a, 5b, 6a	4	1, 2, 3, 5	2, 5b
5 C	38.79			5a, 5b	4a, 4b	
Ha	2.47		2, 3, 4b, 5b	5	1, 3, 4	2
Hb	2.35		2, 4b, 5a	5	1, 3, 4	2, 4b, 10
6 C	38.54			6a, 6b	2, 3	
Ha	2.52	15.48(?), 4.56(?)	2, 3, 4b, 6b, 8	6	2, 3, 4, 7	2, 8, 10
Hb	2.34		3, 6a	6	2, 3, 4, 7	8
7 C	172.60				6a, 6b, 8	
8 C	51.69			8		
H3	3.55		6a	8	7	4a, 6a, 6b, 10, 13
9 C	137.63				2	
10 C	127.11			10	2, 10, 12, 13	
H	6.69	1.38(?), 0.79(?), 0.79(?), 0.68(?)	2, 12, 13	10	2, 10, 11, 12, 13	2, 3, 5b, 6a, 8, 13
11 C	138.56				10, 13	
12 C	129.17			12	10, 13	
H	6.91	1.51(?), 1.51(?), 0.76(?), 0.74(?), 0.74(?), 0.74(?)	10, 13	12	10, 13	13
13 C	21.37			13	10, 12, 13	
H3	2.29	0.65(?), 0.65(?), 0.61(?)	10, 12	13	10, 11, 12, 13	8, 10, 12

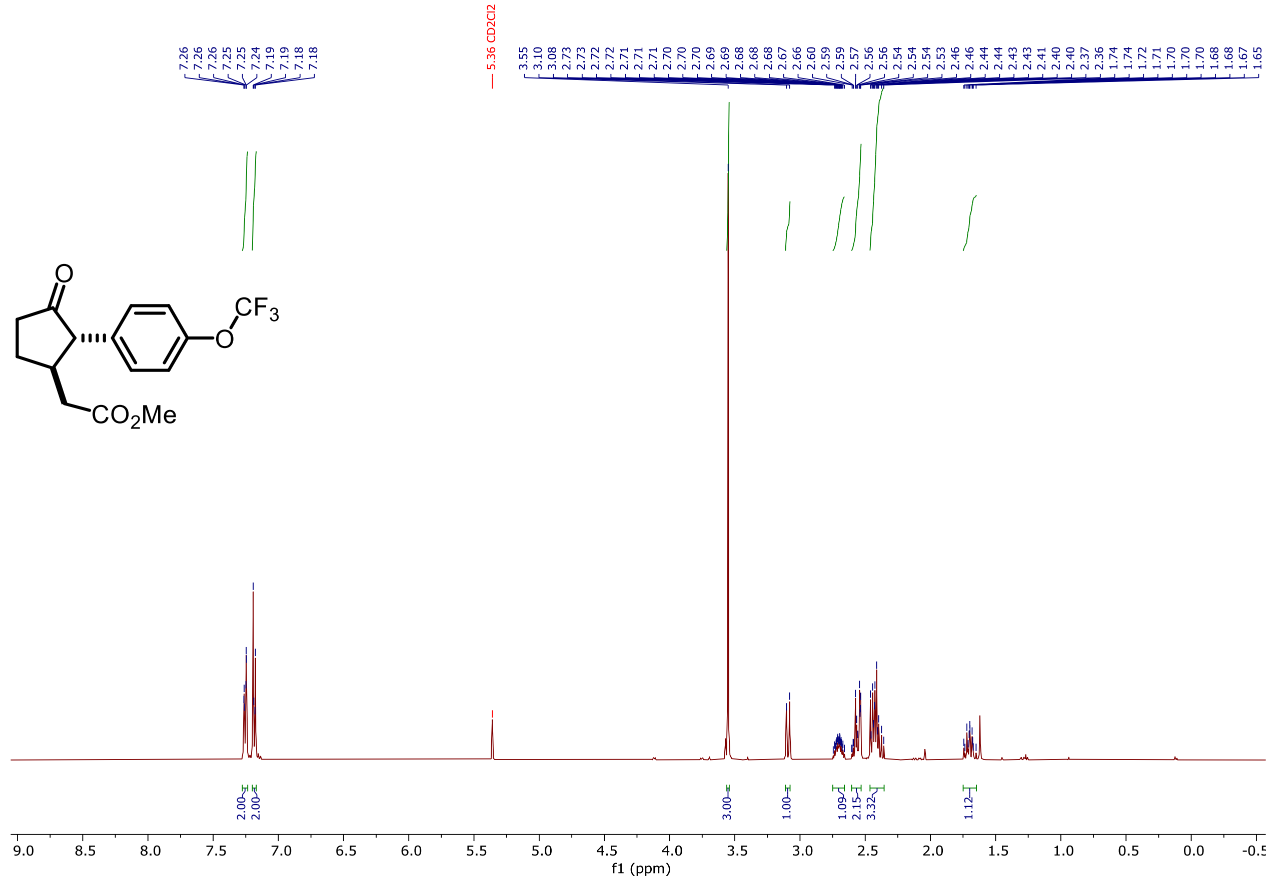

O
CF3
O
CO2Me
7.26
7.26
7.26
7.25
7.25
7.24
7.19
7.19
7.18
7.18
5.36 CD2Cl2
3.55
3.10
3.08
2.73
2.73
2.72
2.72
2.71
2.71
2.71
2.70
2.70
2.70
2.69
2.69
2.68
2.68
2.68
2.67
2.66
2.60
2.59
2.59
2.57
2.56
2.56
2.54
2.54
2.54
2.54
2.53
2.46
2.46
2.44
2.44
2.43
2.43
2.41
2.40
2.40
2.37
2.36
1.74
1.72
1.71
1.71
1.70
1.70
1.68
1.68
1.67
1.65
2.00
2.00
3.00
1.00
1.09
2.15
3.32
1.12
f1 (ppm)

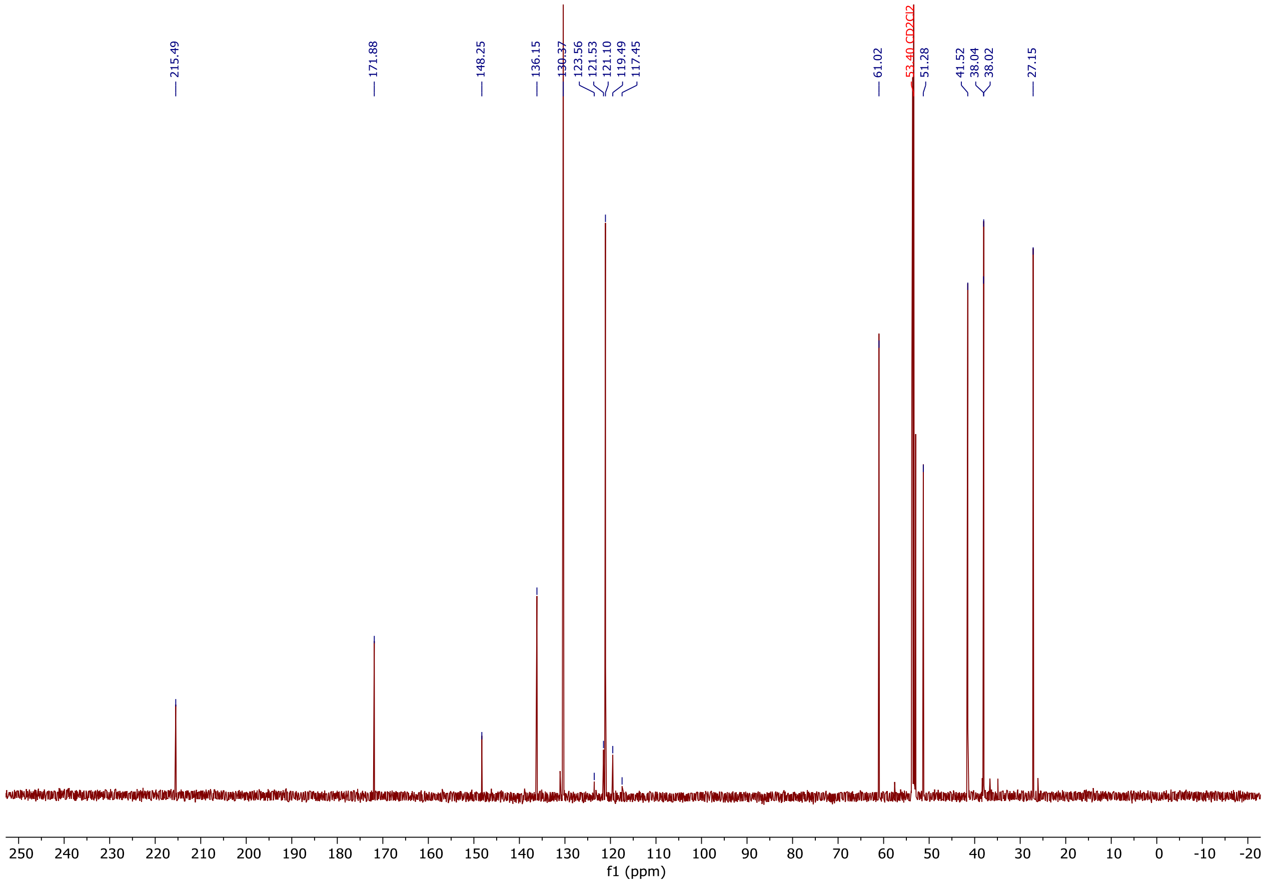

215.49
171.88
148.25
136.15
130.37
123.56
121.53
121.10
119.49
117.45
61.02
53.40 CD2Cl2
51.28
41.52
38.04
38.02
27.15
f1 (ppm)

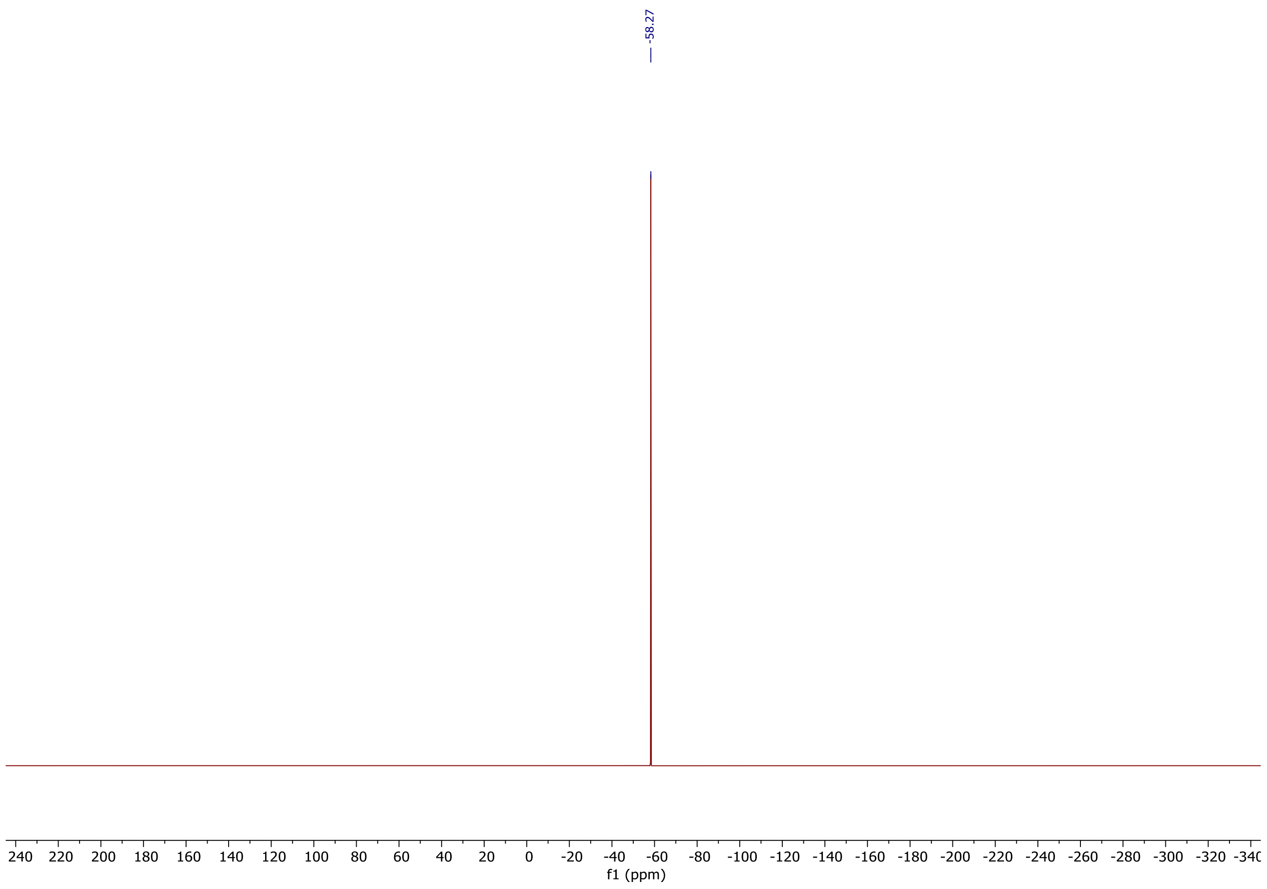

-58.27
240 220 200 180 160 140 120 100 80 60 40 20 0 -20 -40 -60 -80 -100 -120 -140 -160 -180 -200 -220 -240 -260 -280 -300 -320 -340
f1 (ppm)

HSQC spectra of 6f

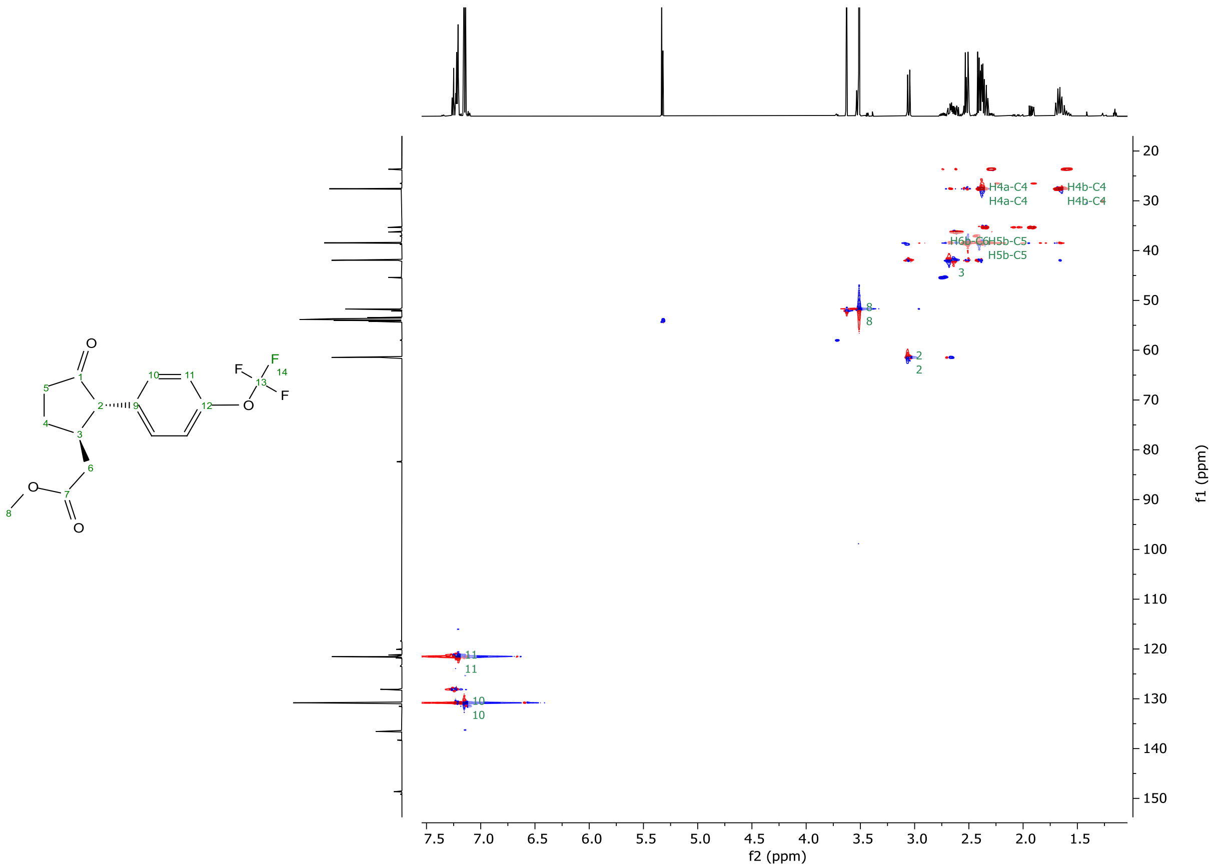

HMBC spectra of 6f

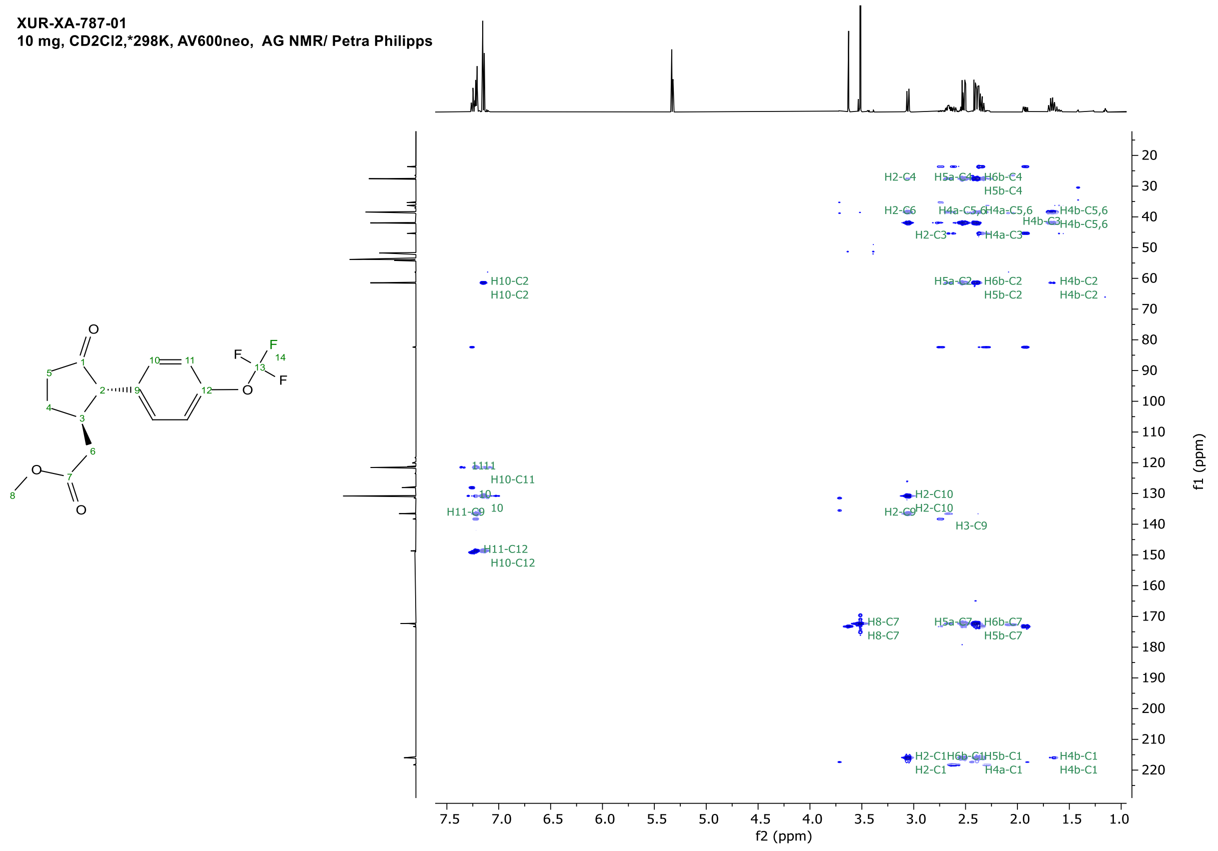

COSY spectra of 6f

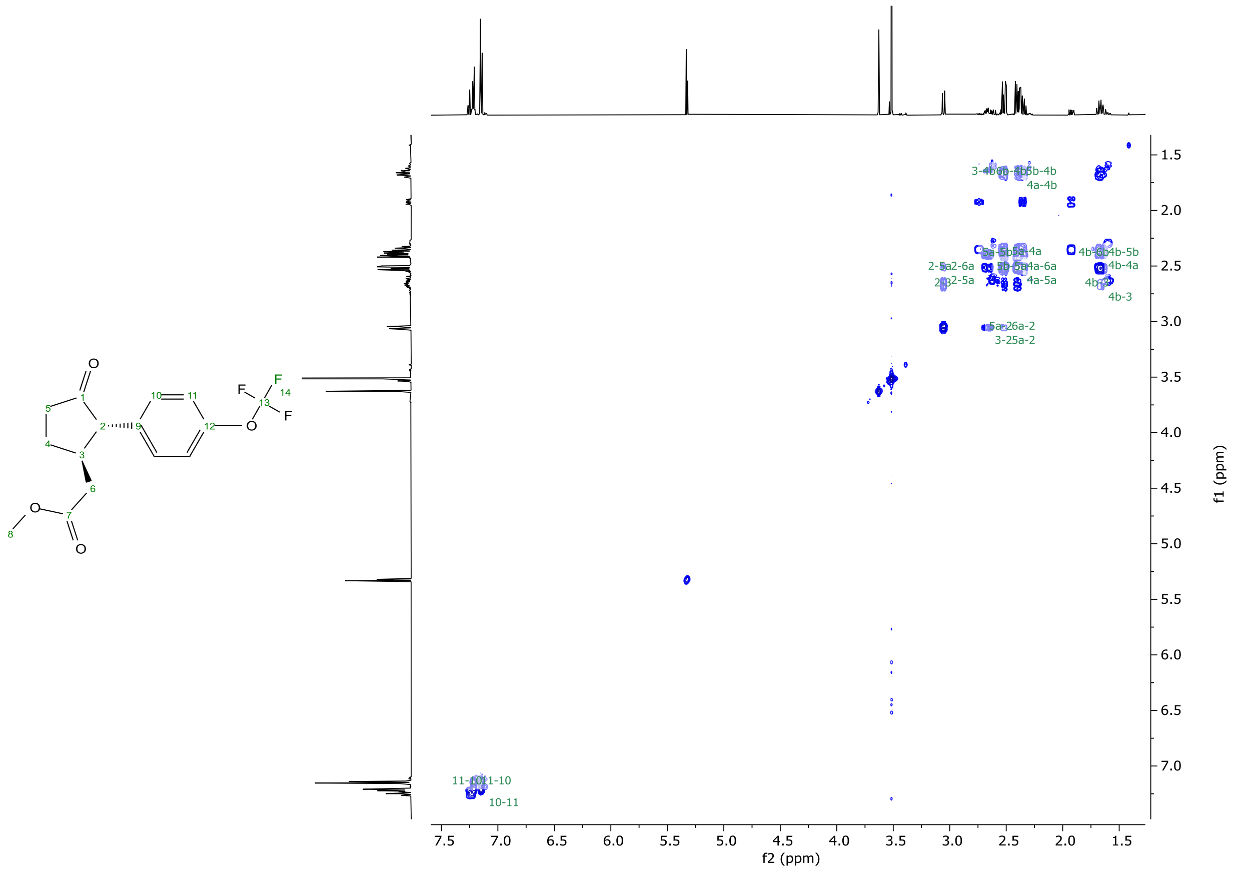

NOESY spectra of 6f

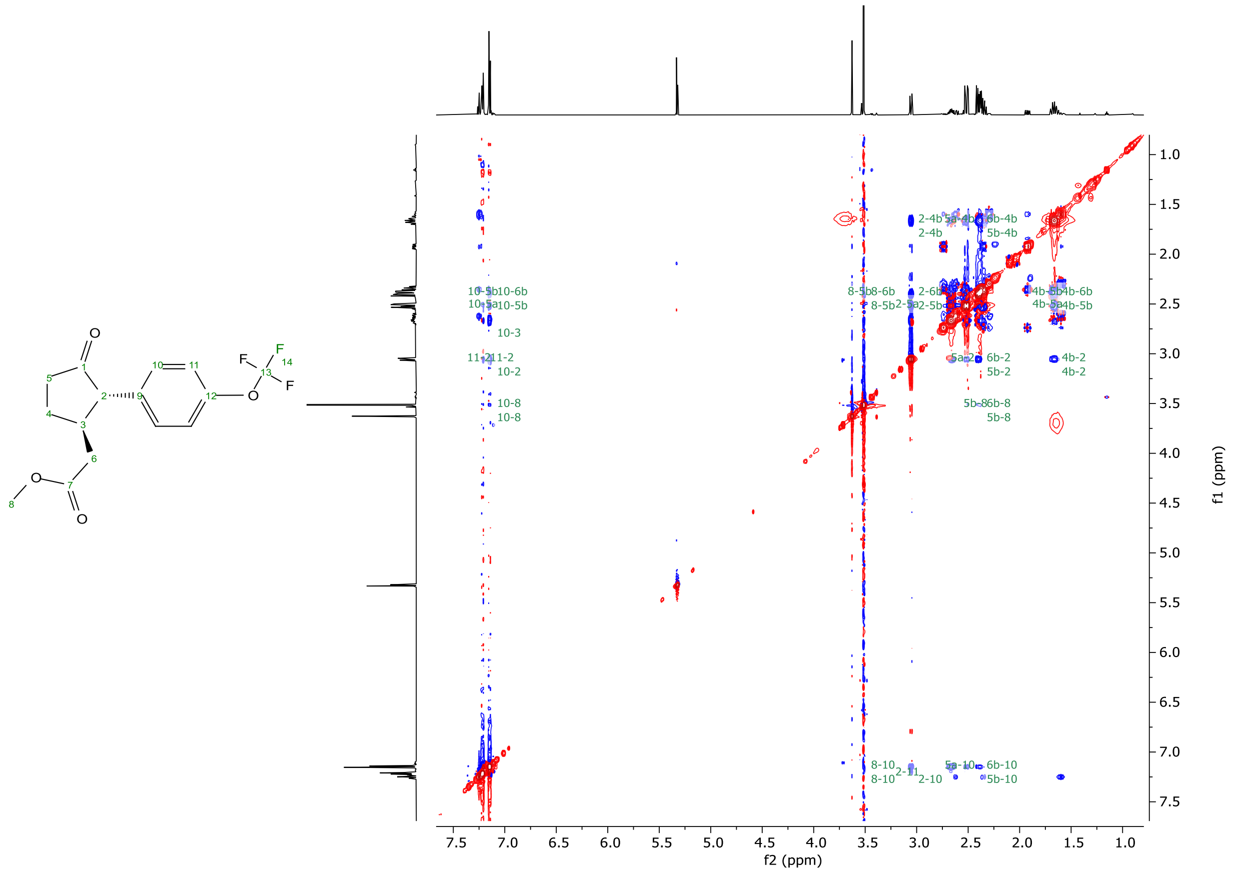

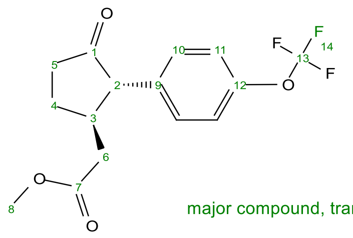

major compound, trans

- major trans isomer, δ C6 = 38.43, 38.47 ppm !

- in NOESY :

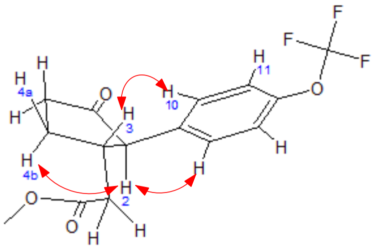

Atom	δ (ppm)	J	COSY	HSQC	HMBC	NOESY
1 C	215.99				2, 4a, 4b, 5a, 5b, 6a, 6b	
2 C	61.45			2	3, 4b, 5a, 5b, 6a, 6b, 10	
H	3.06	12.35(?), 1.22(?)	3, 5a, 6a	2	1, 3, 4, 6, 9, 10	4b, 5a, 5b, 6b, 10, 11
3 C	41.94			3	2, 4a, 4b, 5a, 5b, 6a, 6b	
H	2.67		2, 4b, 5a, 5b, 6a, 6b	3	2, 4, 6, 7, 9	10, 11
4 C	27.58			4a, 4b	2, 3, 5a, 5b, 6a, 6b	
Ha	2.38		4b, 5a, 6a	4	1, 3, 5, 6	
Hb	1.66		3, 4a, 5a, 5b, 6a, 6b	4	1, 2, 3, 5, 6	2, 5a, 5b, 6a, 6b
5 C	38.43, 38.47			5a, 5b	4a, 4b, 6b	
Ha	2.52		2, 3, 4a, 4b, 5b	5	1, 2, 3, 4, 7	2, 4b, 10
Hb	2.39		3, 4b, 5a	5	1, 2, 3, 4, 6, 7	2, 4b, 8, 10
6 C	38.43, 38.47			6a, 6b	2, 3, 4a, 4b, 5b	
Ha	2.52		2, 3, 4a, 4b, 6b	6	1, 2, 3, 4, 7	4b
Hb	2.39		3, 4b, 6a	6	1, 2, 3, 4, 5, 7	2, 4b, 8, 10
7 C	172.32				3, 5a, 5b, 6a, 6b, 8	
8 C	51.73			8		
H3	3.51			8	7	5b, 6b, 10
9 C	136.57				2, 3, 11	
10 C	130.80			10	2, 10, 11	
H	7.15		11	10	2, 10, 11, 12	2, 3, 5a, 5b, 6b, 8
11 C	121.53			11	10, 11	
H	7.22		10	11	9, 10, 11, 12	2, 3
12 C	148.66	1.88(14)			10, 11	
13 C	120.92	257.70(14)				
14 F	-58.30	257.70(13), 1.88(12)				

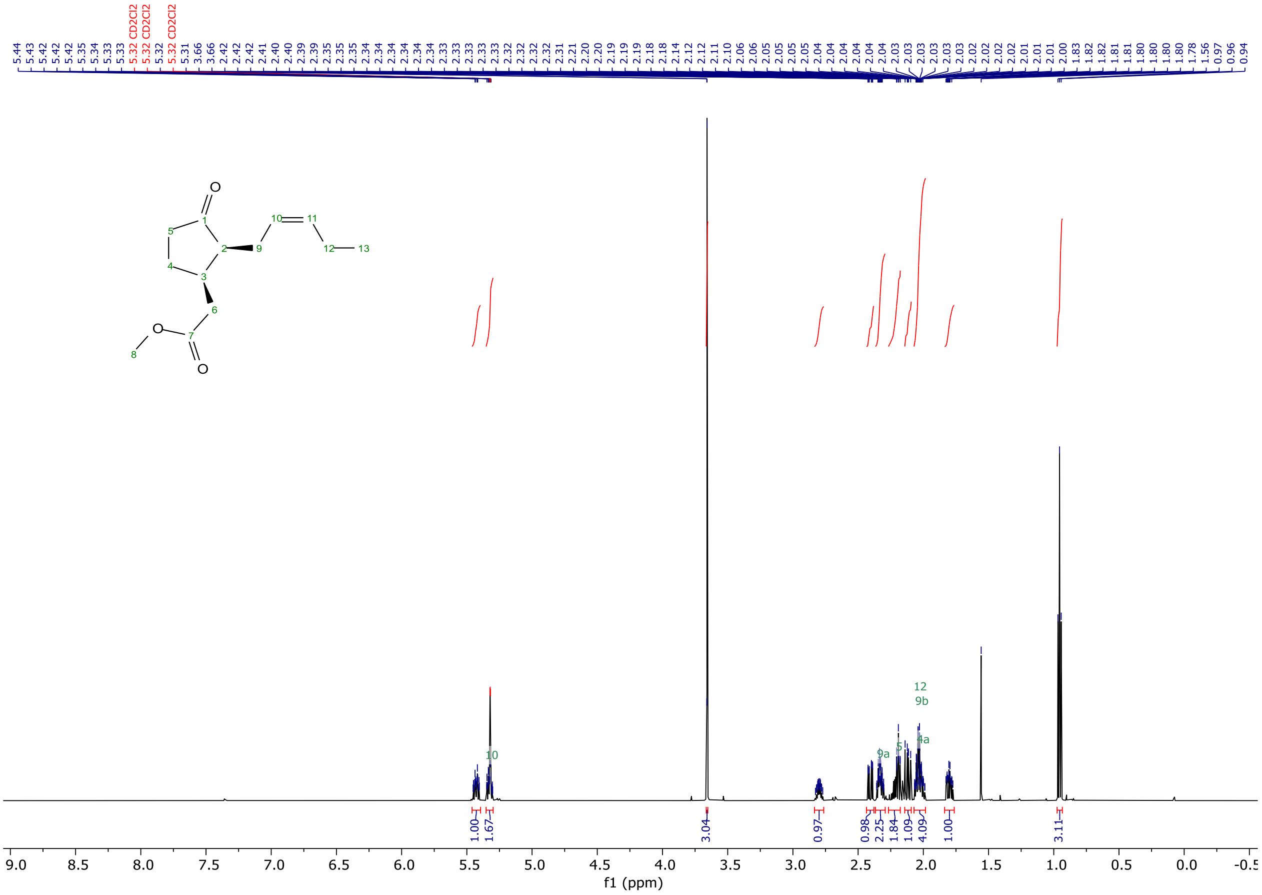

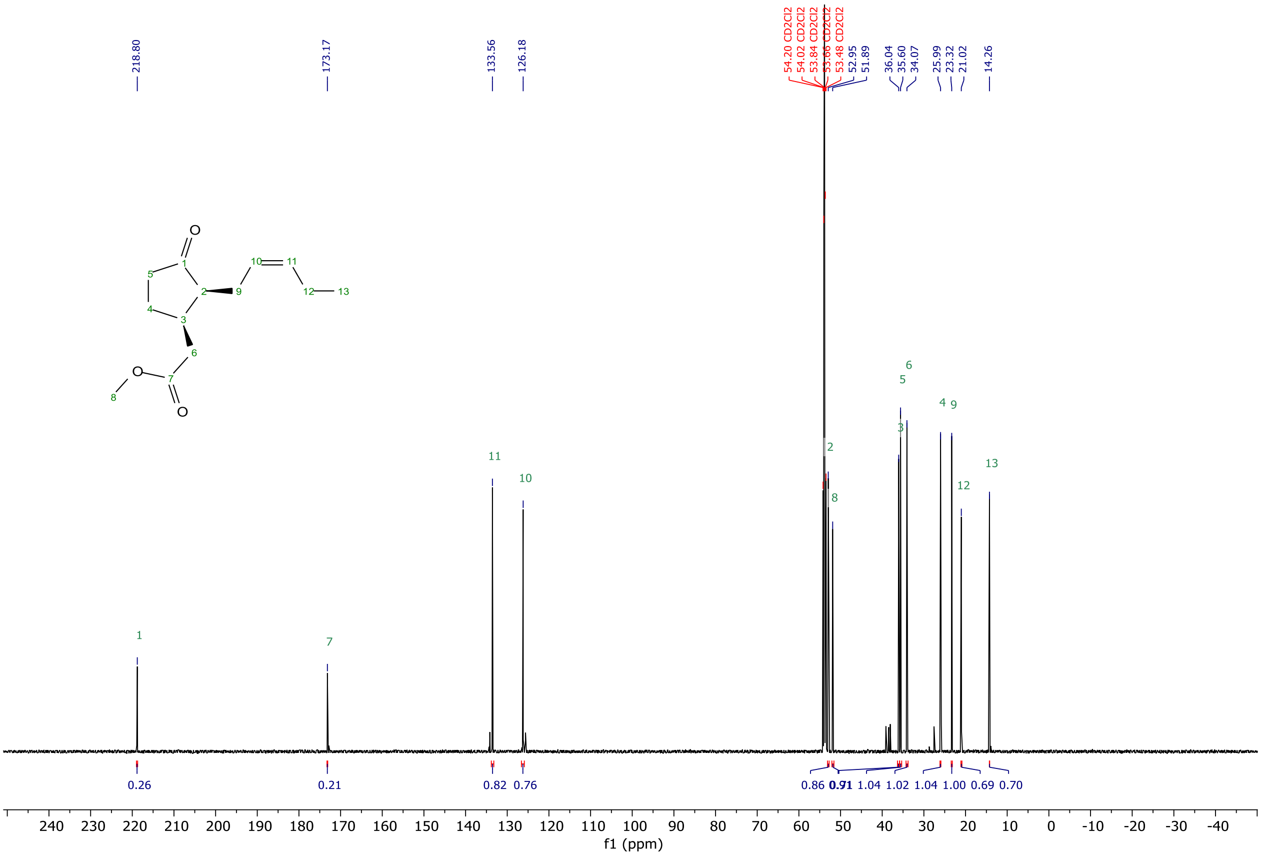

XUR-XA-829-01.11.1.1r — XUR-XA-829-01 (12329) — CD2Cl2; 298.0 K; 5 mg; 5 mm — 13C{1H} (zgdc30) — AV600a (cryoTCI) — 30.04.24 03:29:51

HSQC spectra of 9a

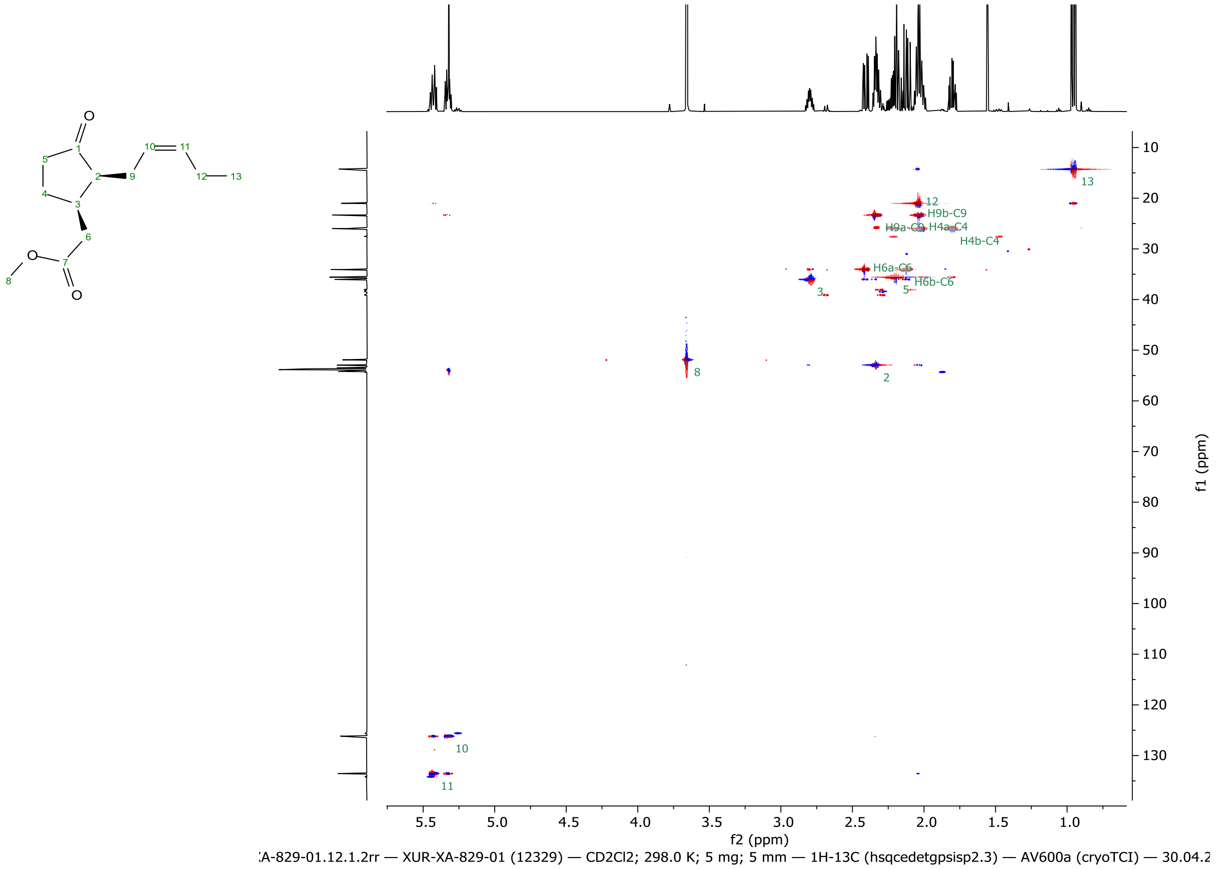

HMBC spectra of 9a

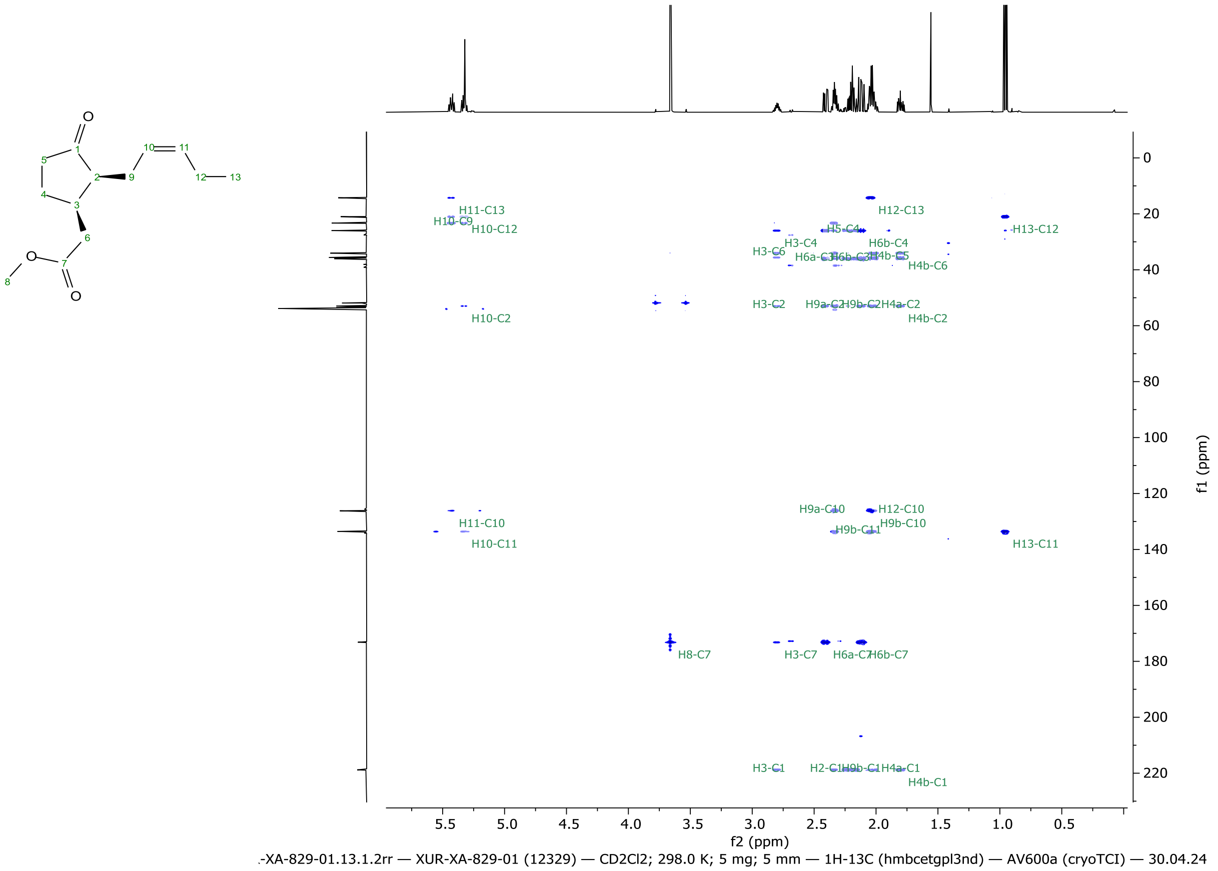

COSY spectra of 9a

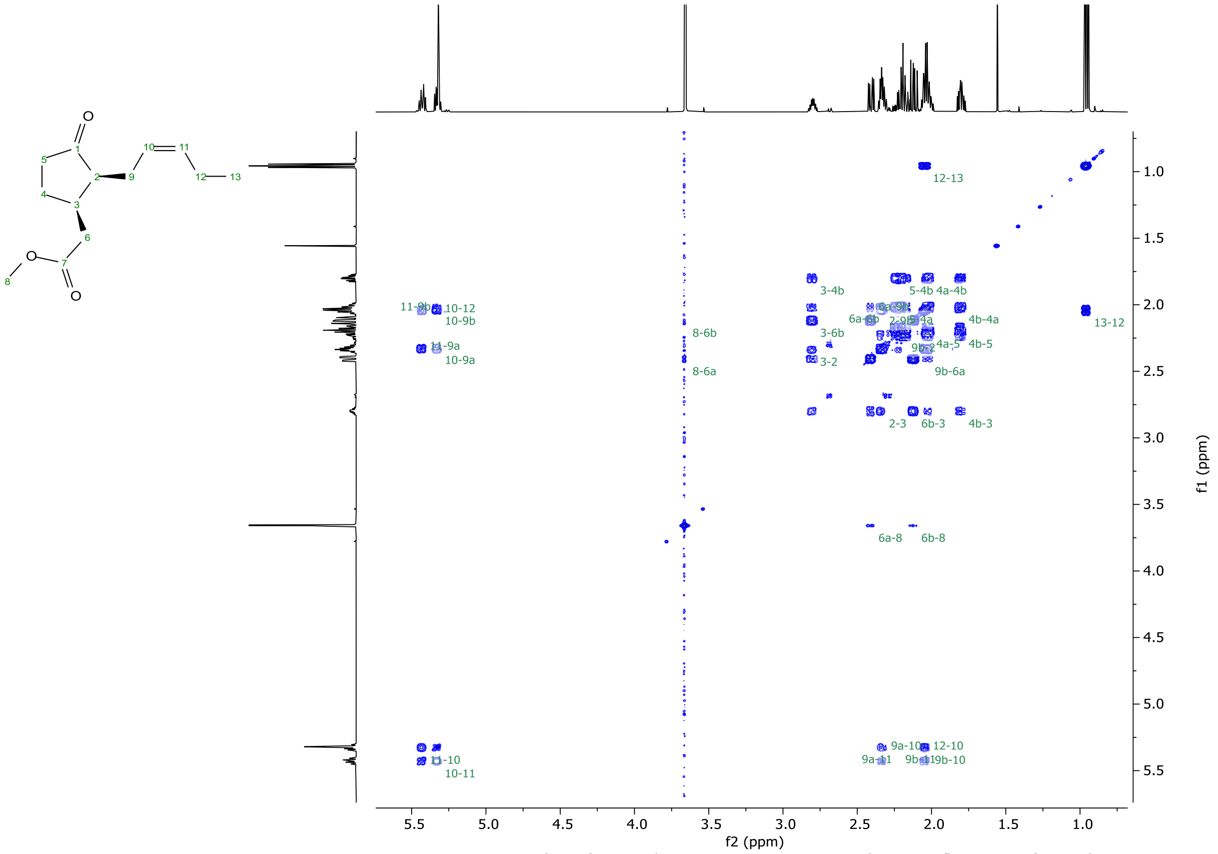

NOESY spectra of 9a

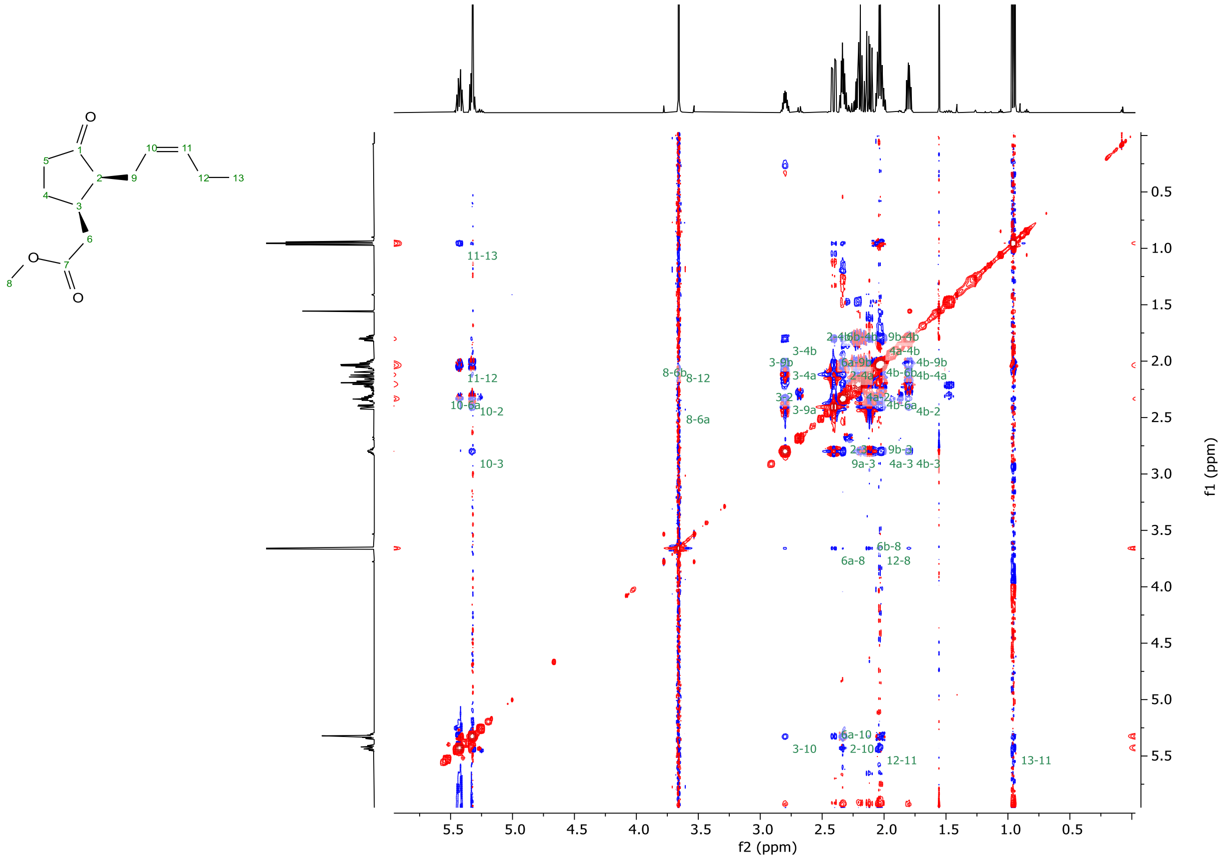

XUR-XA-829-01
5 mg, CD2Cl2, *298K, AV600a, AG NMR/ Petra Philipps

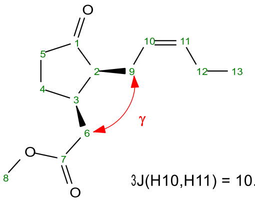

^3J(H10,H11) = 10.5 Hz, cis

relative stereochemistry on C2,C3, cis

- in comparison to C2,C3 trans compound in XUR-XA-828-01, C6 = 34.07 ppm is strongly deshielded due to γ interaction with C9!

- in NOESY: H2 - H3, H4a, H4b, H10
  H3 - H2, H4a, H4b, H9a, H9b, H10

Atom	δ (ppm)	J	COSY	HSQC	HMBC	NOESY
1 C	218.80				2, 3, 4a, 4b, 5, 9b	
2 C	52.95			2	3, 4a, 4b, 6a, 6b, 9a, 9b, 10	
H	2.34		3, 9b	2	1, 6, 9, 10, 11	3, 4a, 4b, 10
3 C	36.04			3	4a, 4b, 5, 6a, 6b, 9a, 9b	
H	2.80	10.15(6b)	2, 4b, 6b	3	1, 2, 4, 5, 6, 7	2, 4a, 4b, 9a, 9b, 10
4 C	25.99			4a, 4b	3, 5, 6a, 6b	
Ha	2.02		4b, 5	4	1, 2, 3, 5, 6	2, 3, 4b
Hb	1.80	13.31(?), 8.59(?), 5.26(?), 4.70(?)	3, 4a, 5	4	1, 2, 3, 5, 6	2, 3, 4a, 6a, 6b, 9b
5 C	35.60			5	3, 4a, 4b	
H2	2.20		4a, 4b	5	1, 3, 4	
6 C	34.07			6a, 6b	2, 3, 4a, 4b, 9b	
Ha	2.41	15.65(6b), 5.49(?), 0.72(?)	6b, 8, 9b	6	2, 3, 4, 7	4b, 8, 9b, 10
Hb	2.12	10.15(3), 15.65(6a)	3, 6a, 8	6	2, 3, 4, 7	4b, 8
7 C	173.17				3, 6a, 6b, 8	
8 C	51.89			8		
H3	3.66		6a, 6b	8	7	6a, 6b, 12
9 C	23.32			9a, 9b	2, 10, 11	
Ha	2.32		10, 11	9	2, 3, 10, 11	3
Hb	2.03		2, 6a, 10, 11	9	1, 2, 3, 6, 10, 11	3, 4b, 6a
10 C	126.18			10	2, 9a, 9b, 11, 12	
H	5.33	10.48(11)	9a, 9b, 11, 12	10	2, 9, 11, 12	2, 3, 6a
11 C	133.56			11	2, 9a, 9b, 10, 12, 13	
H	5.43	10.48(10), 7.20(?), 7.20(?), 1.63(?), 1.63(?	9a, 9b, 10, 12	11	9, 10, 12, 13	12, 13
12 C	21.02			12	10, 11, 13	
H2	2.04	7.54(13)	10, 11, 13	12	10, 11, 13	8, 11
13 C	14.26			13	11, 12	
H3	0.96	7.54(12)	12	13	11, 12	11

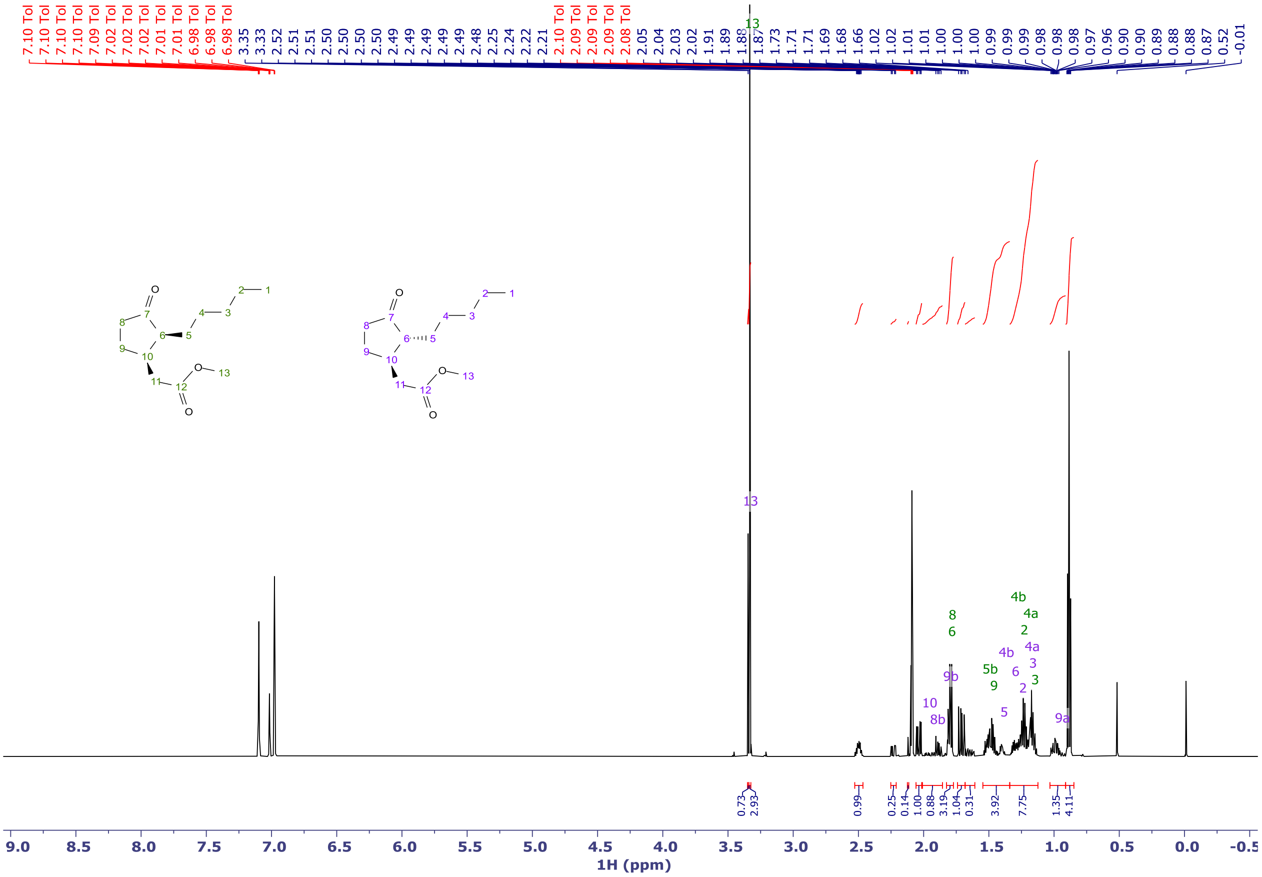
1H (ppm)

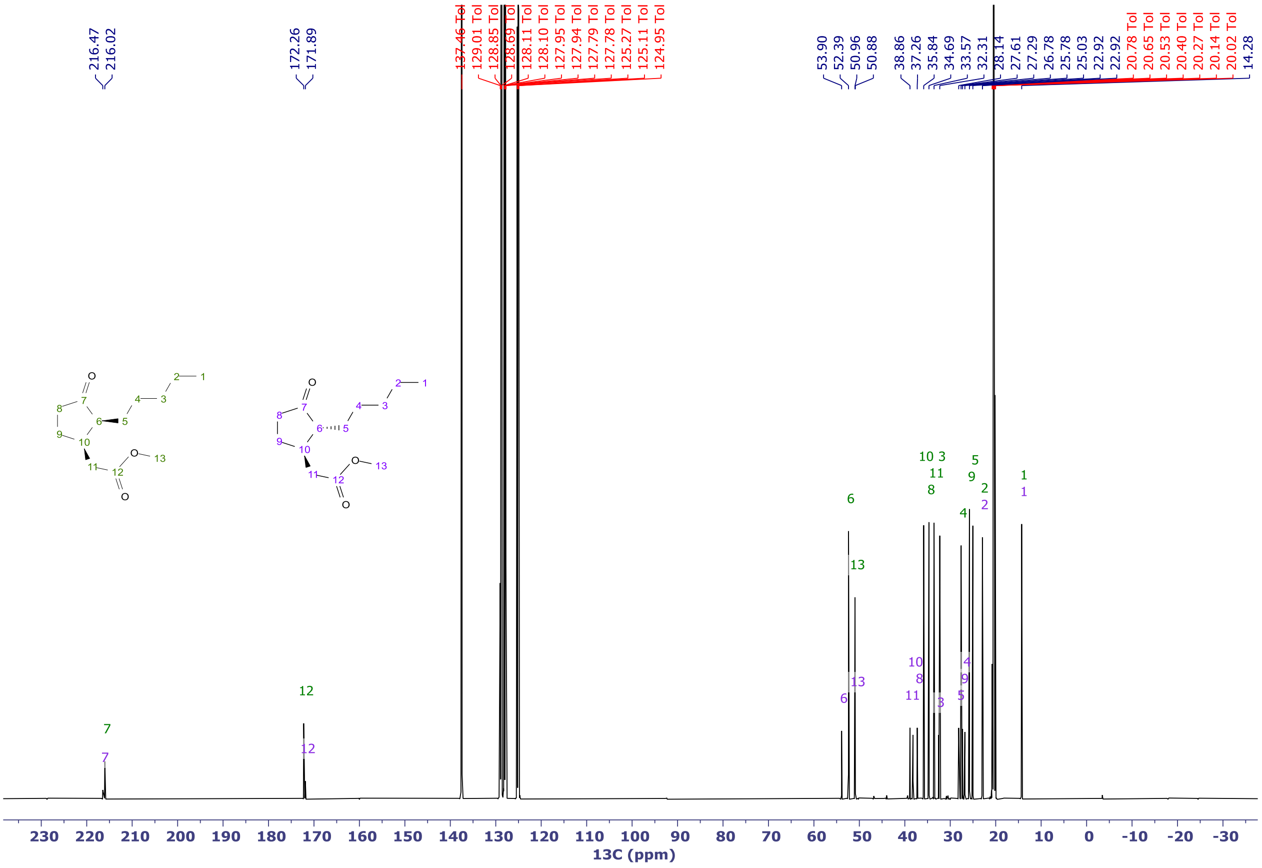
13C (ppm)

HMBC spectra of 9b

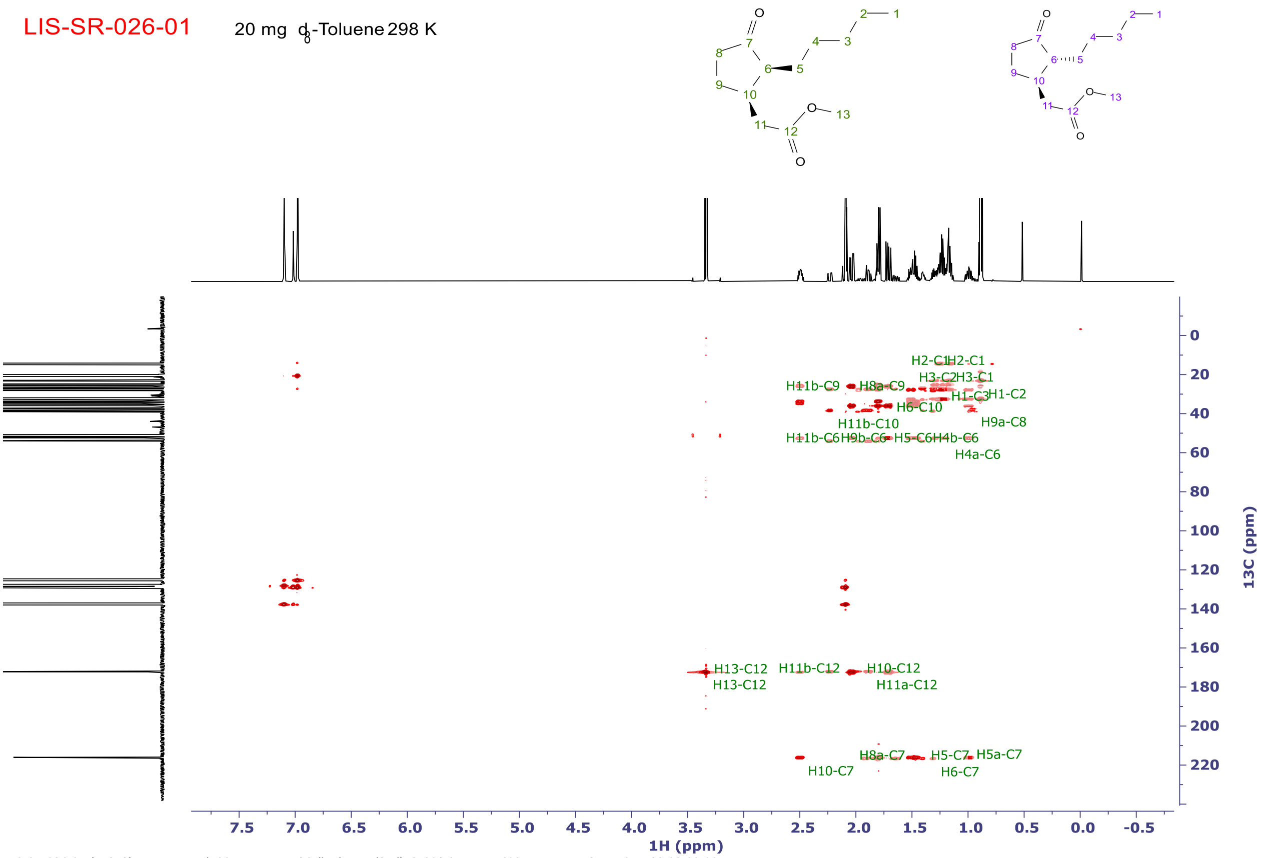

LIS-SR-026-01 (11352) — 5 mm, Tol, 20 mg — 1H-13C (hmbcetgpl3nd) @ 298.0 K — AV600neo, cryoBBO — 16.11.23 00:09:00

HSQC spectra of 9b

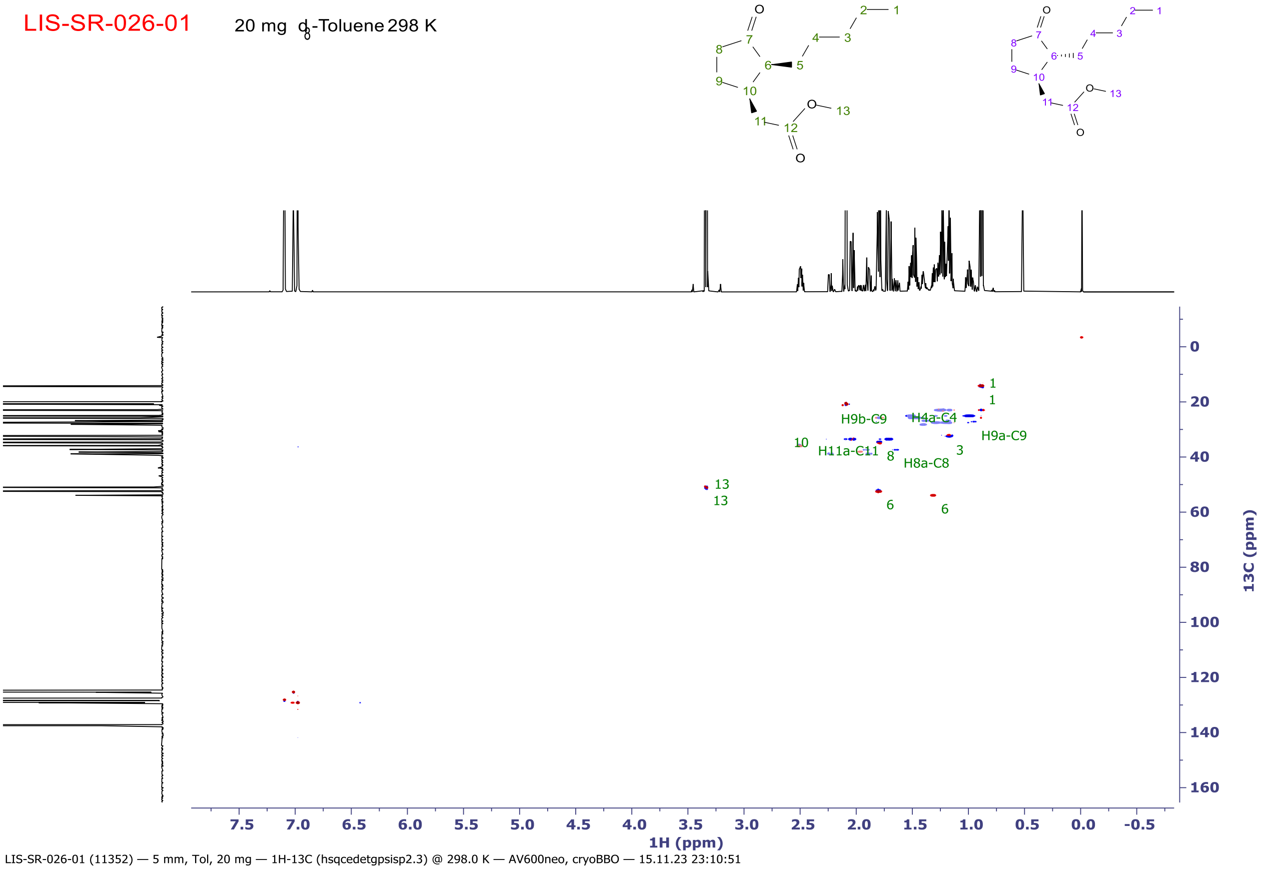

LIS-SR-026-01 (11352) — 5 mm, Tol, 20 mg — 1H-13C (hsqcedetgpsisp2.3) @ 298.0 K — AV600neo, cryoBBO — 15.11.23 23:10:51

COSY spectra of 9b

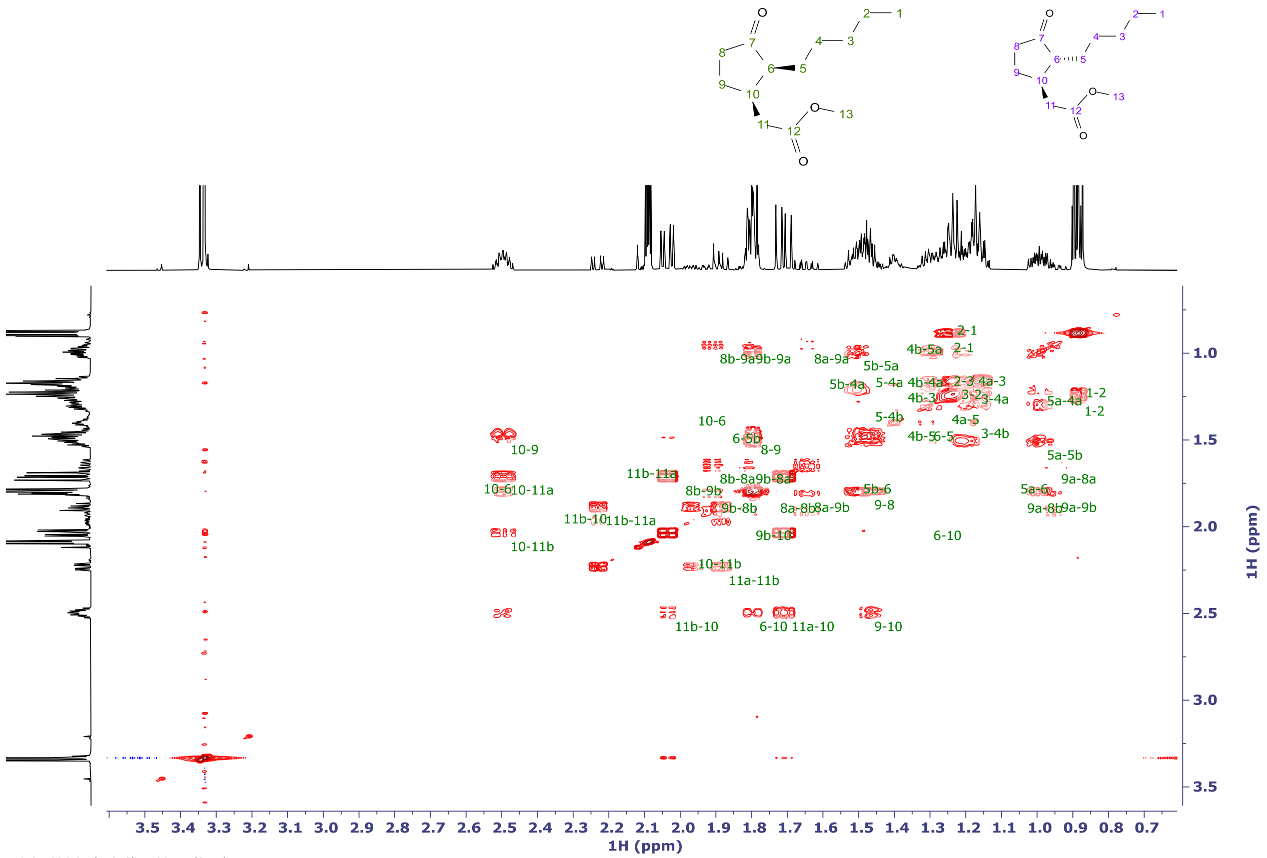

LIS-SR-026-01 (11352) — 20mg d8-Toluene

NOESY spectra of 9b

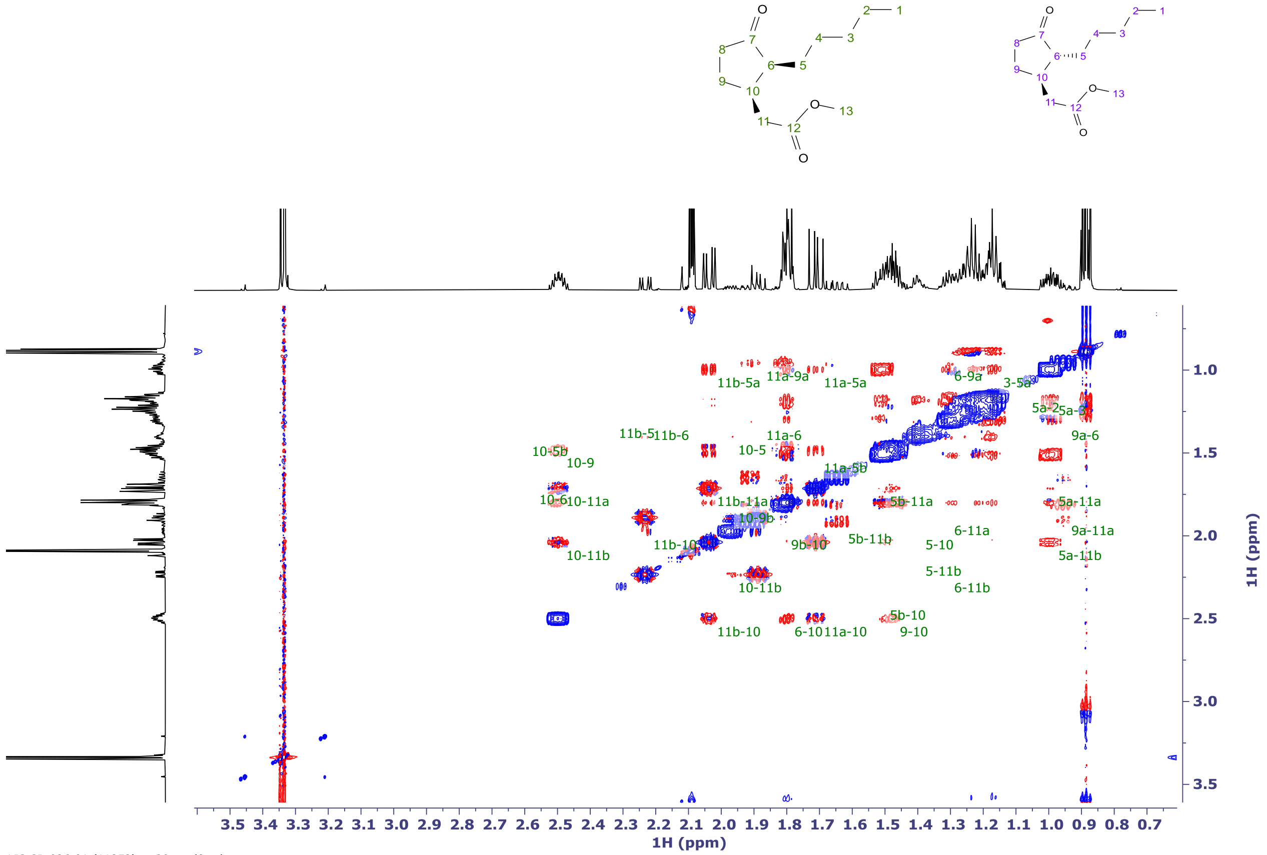

LIS-SR-026-01 (11352) — 20mg d8-toluene

cis-configured product
ca. 80 %

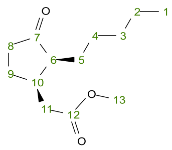

important NOEs:

H10 - H6
H11b - H5a,b

Atom	δ (ppm)	Min..Max (ppm)	J	COSY	HSQC	HMBC	NOESY
1 C	14.28	14.28..14.29			1	2, 3	
H3	0.88	0.86..0.91	7.20(?)	2	1	2, 3	
2 C	22.92	22.92..22.93			2	1, 3, 4a, 4b	
H2	1.25	1.24..1.25		1, 3	2	1, 3, 4	5a
3 C	32.31	32.30..32.31			3	1, 2, 4a, 4b, 5a, 5b	
H2	1.16	1.16..1.17		2, 4a, 4b	3	1, 2, 4, 5	5a
4 C	27.61	27.61..27.62			4a, 4b	2, 3, 5a, 5b, 6	
Ha	1.20	1.19..1.20		3, 4b, 5a, 5b	4	2, 3, 5, 6	
Hb	1.29	1.29..1.30		3, 4a, 5a, 5b	4	2, 3, 5, 6	
5 C	25.03	25.02..25.03			5a, 5b	3, 4a, 4b, 6, 10	
Ha	0.99	0.96..1.03		4a, 4b, 5b, 6	5	3, 4, 6, 7, 10	2, 3, 11a, 11b
Hb	1.51	1.51..1.51		4a, 4b, 5a, 6	5	3, 4, 6, 7, 10	10, 11a, 11b
6 C	52.39	52.38..52.39			6	4a, 4b, 5a, 5b, 8, 9, 10, 11a, 11b	
H	1.80	1.79..1.80		5a, 5b, 10	6	4, 5, 7, 9, 11	10
7 C	216.02	216.01..216.02				5a, 5b, 6, 8, 9, 10	
8 C	34.69	34.68..34.69			8	10	
H2	1.80	1.79..1.80		9	8	6, 7, 10	
9 C	25.78	25.77..25.78			9	6, 10, 11a, 11b	
H2	1.48	1.47..1.48		8, 10	9	6, 7, 10, 11	10
10 C	35.84	35.84..35.85			10	5a, 5b, 8, 9, 11a, 11b	
H	2.50	2.47..2.53	10.00(?), 7.40(?), 5.60(?), 4.20(?)	6, 9, 11a, 11b	10	5, 6, 7, 8, 9, 11, 12	5b, 6, 9, 11a, 11b
11 C	33.57	33.56..33.57			11a, 11b	6, 9, 10	
Ha	1.71	1.69..1.74	15.50(?), 10.10(?)	10, 11b	11	6, 9, 10, 12	5a, 5b, 10, 11b
Hb	2.04	2.00..2.07	15.50(?), 5.40(?)	10, 11a	11	6, 9, 10, 12	5a, 5b, 10, 11a
12 C	172.26	172.25..172.26				10, 11a, 11b, 13	
13 C	50.96	50.95..50.96			13		
H3	3.33	3.33..3.33			13	12	

P-ID CW00914
Measured on:17/11/2023
CHIFFRE:IS-SR-026-01
ELNA#:11352
Client: Ruigang Xu
Group: List
Spectroscopist: Wirtz
Analysed on:22/11/2023
Analysed by: Wirtz
Amount:20.0 mg
Solvent: Tol
Reference: 1H on solvent,other nuclei w/ xiref
Temperature:298 K
Spectrometer:av600a
Probe: cryoTCI
Experiments:1H-zg30, 13C-zgpg30, 1H-13C-hsqcedetgpsisp2.3
1H-13C-hmbcetgpl3nd, 1H-1H-cosygpppqf, 1H-1H-noesygpph,
1H-seldigpzs, 1H-selnogpzs.2

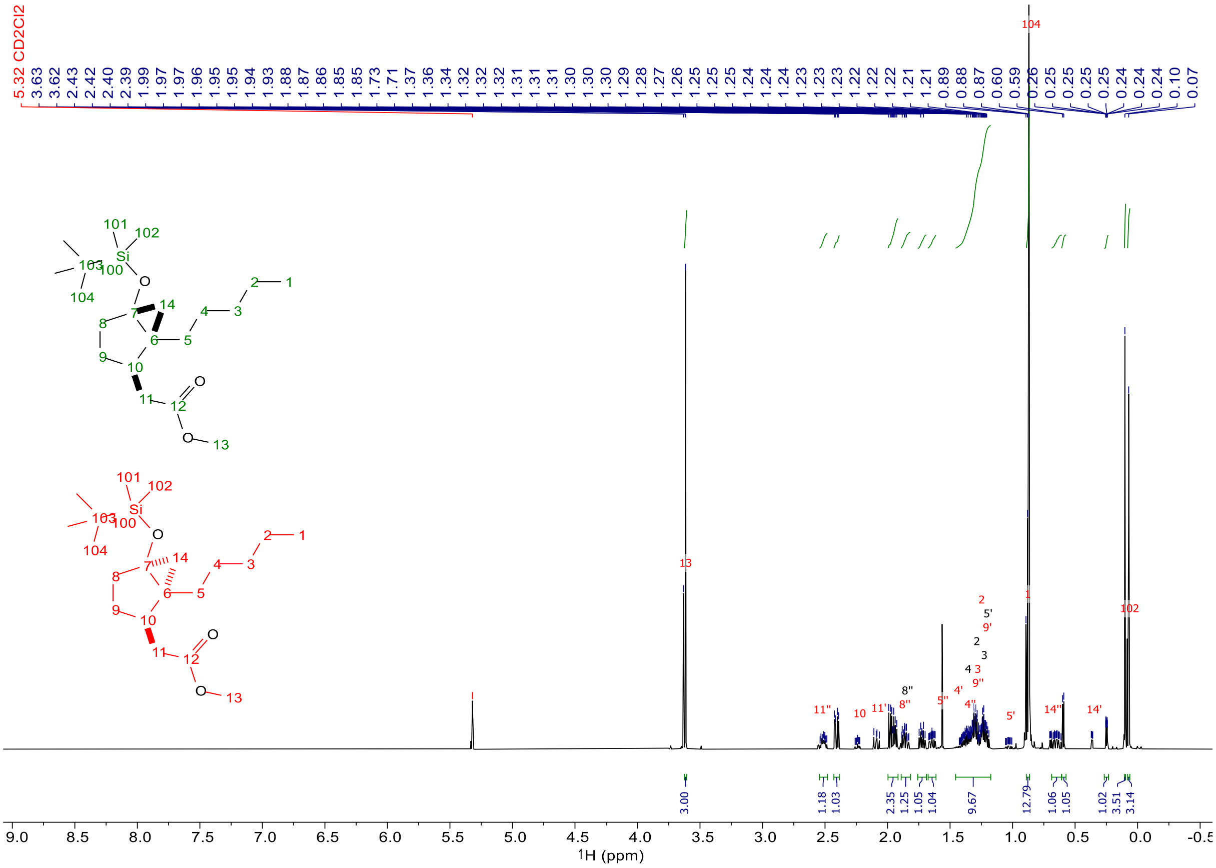
5.32 CD2Cl2
1H (ppm)

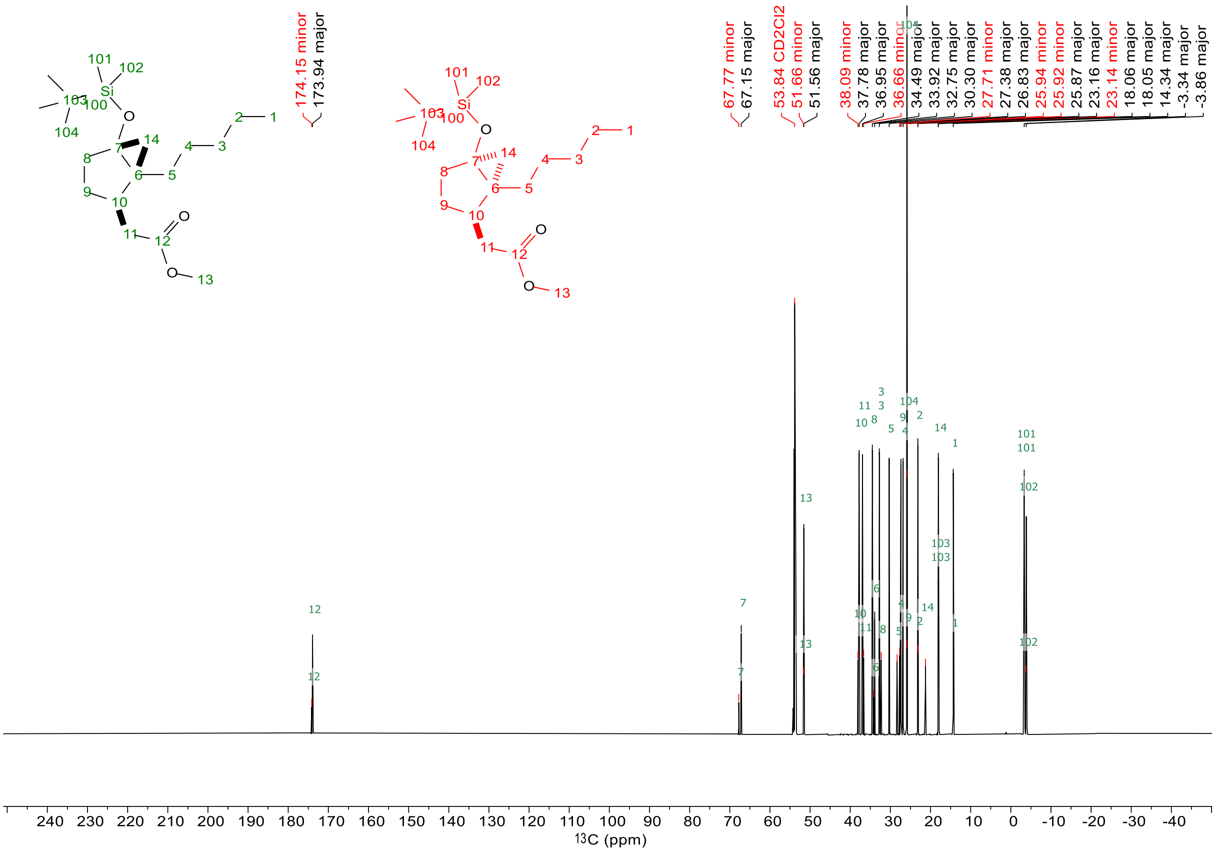
174.15 minor
173.94 major
67.77 minor
67.15 major
53.84 CD2Cl2
51.66 minor
51.56 major
38.09 minor
37.78 major
36.95 major
36.66 minor
34.49 major
33.92 major
32.75 major
30.30 major
27.71 minor
27.38 major
26.83 major
25.94 minor
25.92 minor
25.87 major
23.16 major
23.14 minor
18.06 major
18.05 major
14.34 major
-3.34 major
-3.86 major
13C (ppm)

NOESY spectra of 10

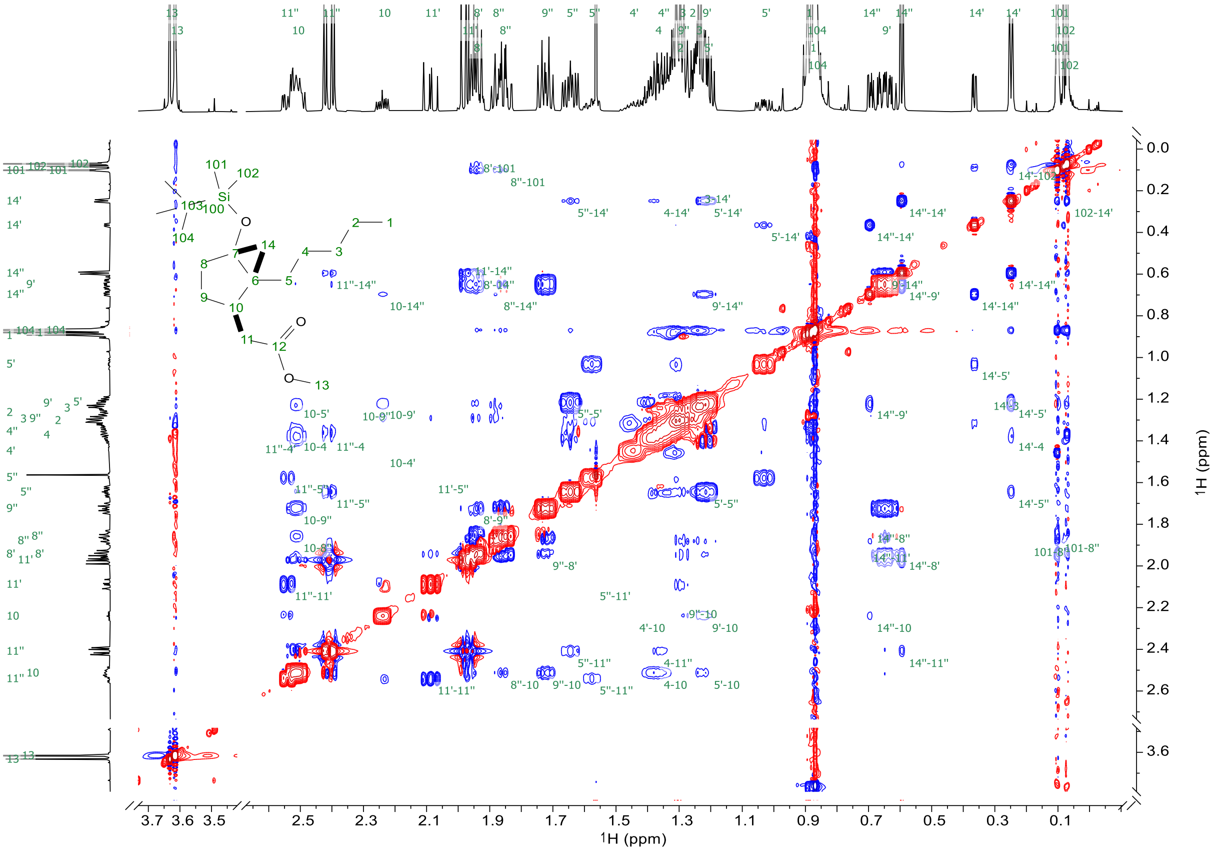

HMBC spectra of 10

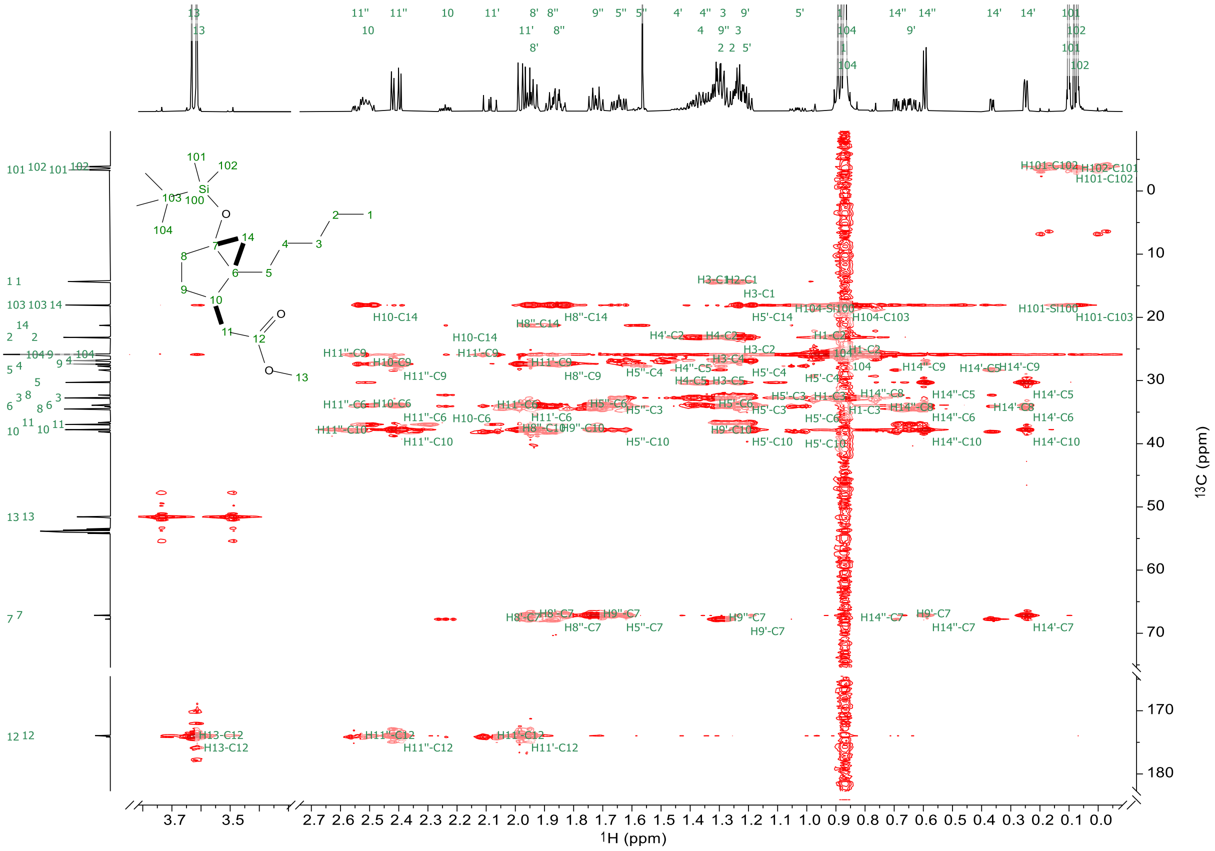

HMBC spectra of 10

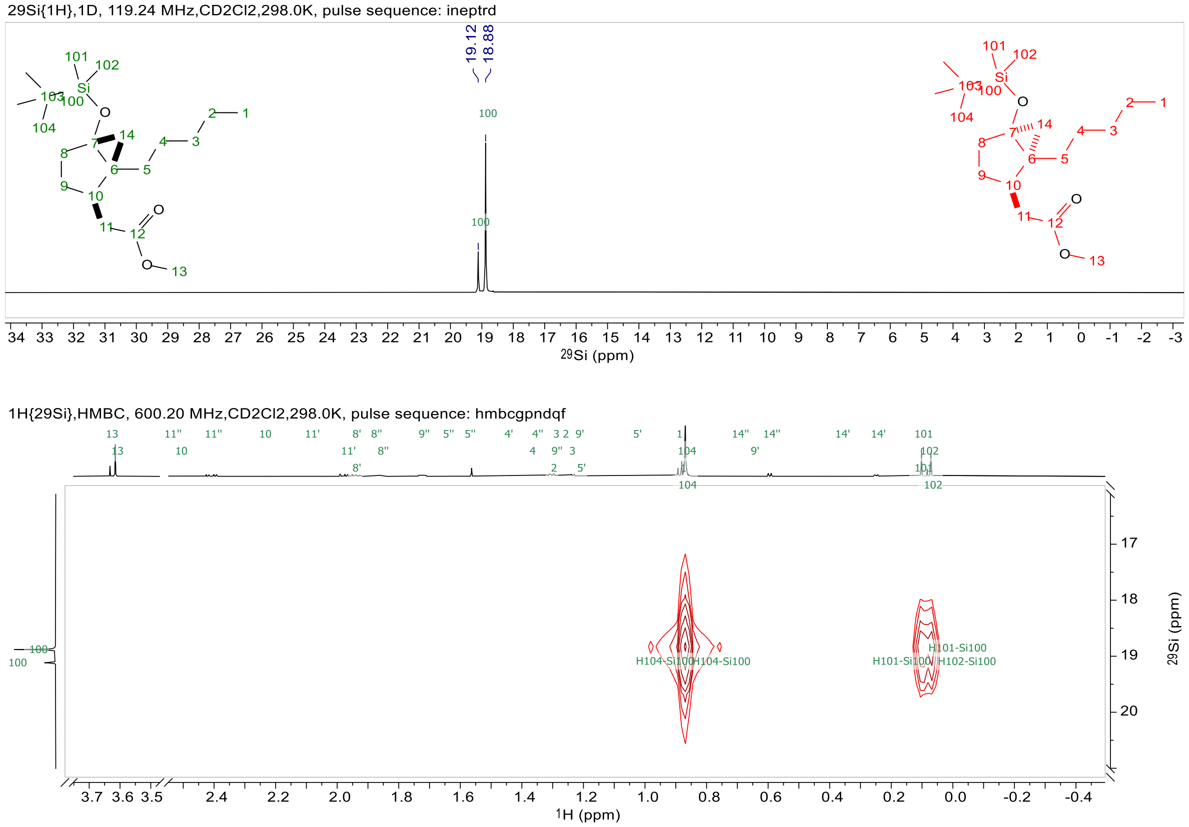

HSQC spectra of 10

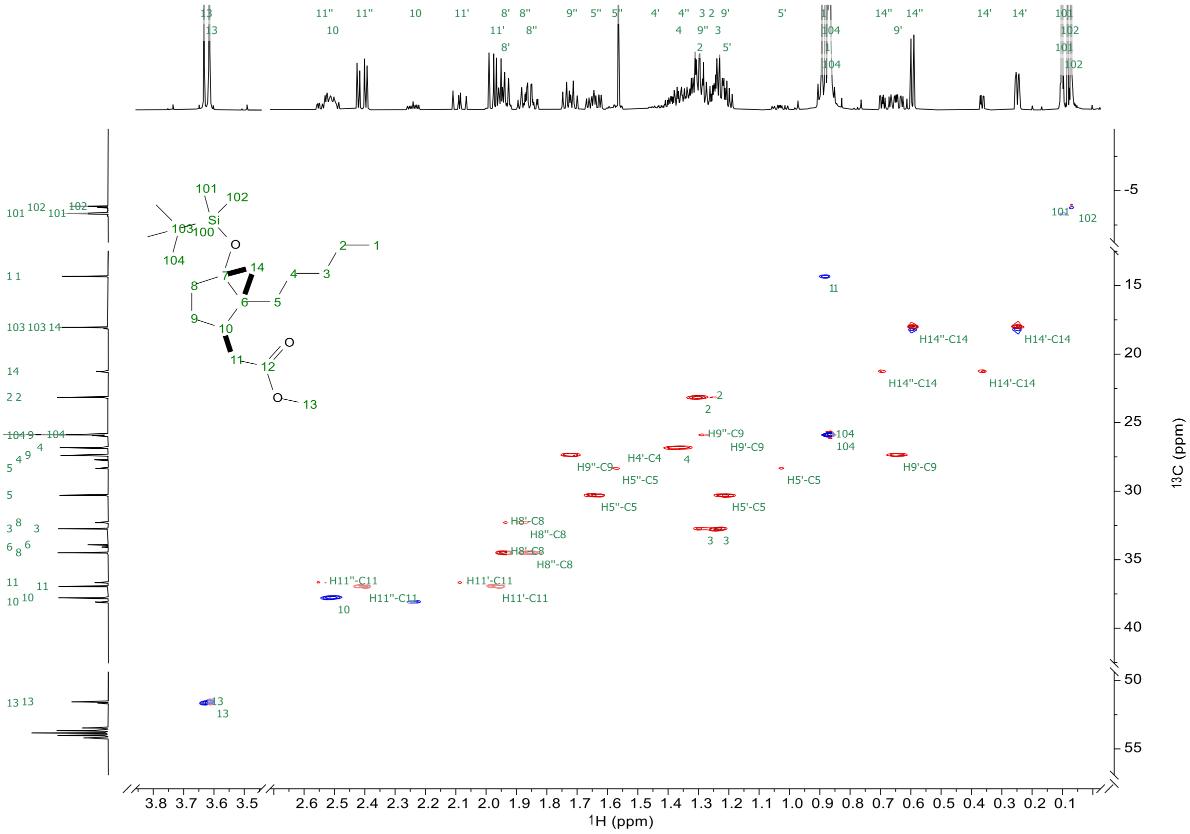

COSY spectra of 10

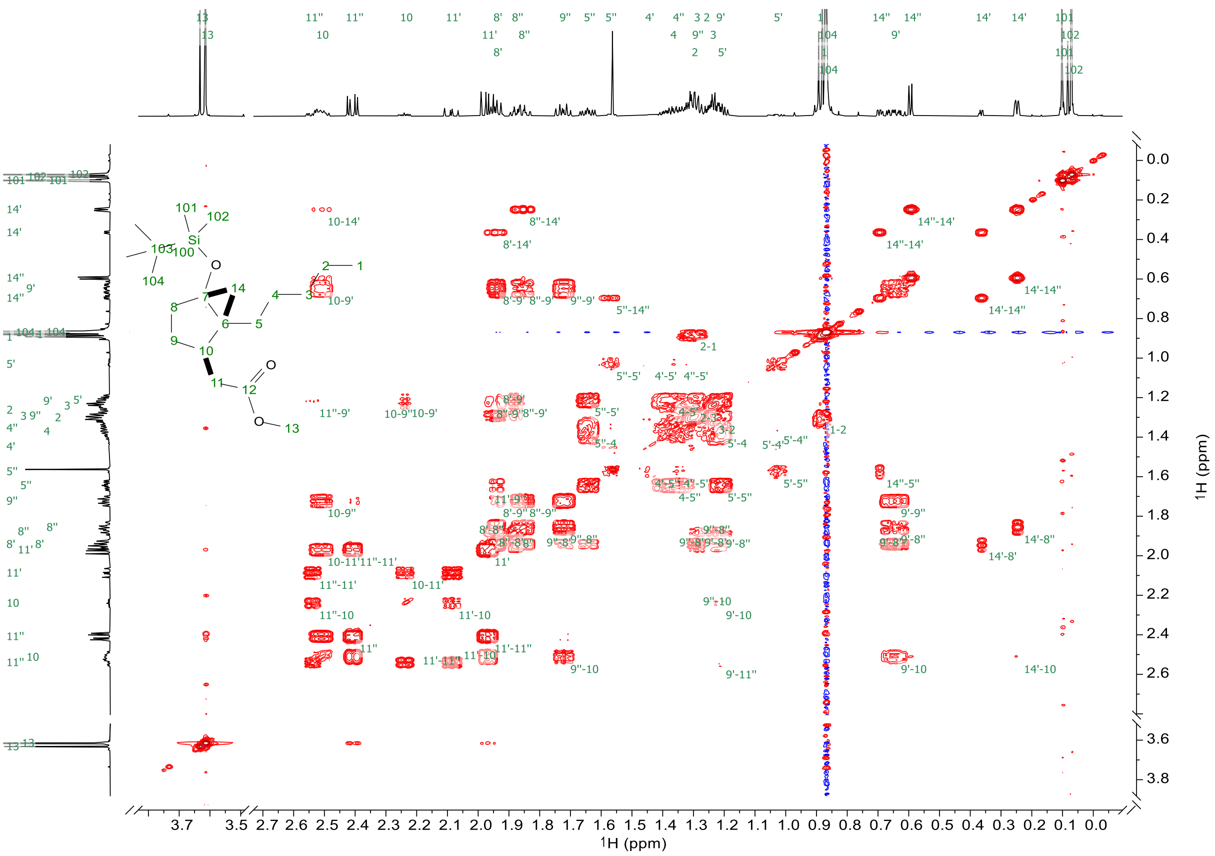

NOE analysis of 10

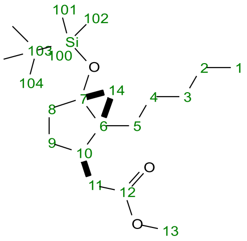

Excerpt of a Chem3D model showing important NOEs for relative stereochemistry assignment

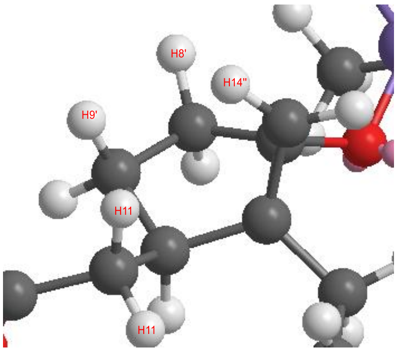

Atom	δ (ppm)	J	COSY	HSQC	HMBC	NOESY
1 C	14.337			1	2, 3	
H3	0.881		2	1	2, 3	
2 C	23.160			2	1, 3, 4	
H2	1.302		1, 3	2	1, 3, 4	
3 C	32.748			3	1, 2, 5', 5"	
H2	1.242		2	3	1, 2, 4, 5	14'
4 C	26.826			4	2, 3, 5', 5"	
H2	1.371		5', 5"	4	2, 5	10, 11", 14'
5 C	30.297			5', 5"	3, 4, 14', 14"	
H'	1.213		4, 5"	5	3, 4, 6, 10, 14	5", 10, 14'
H"	1.645		4, 5'	5	3, 4, 6, 7, 10	5', 11", 14'
6 C	33.917				5', 5", 9", 10, 11', 11", 14', 14"	
7 C	67.154				5", 8', 8", 9', 9", 14', 14"	
8 C	34.495			8', 8"	9', 14', 14"	
H'	1.942	11.80(8"), 7.80(9")	9', 9"	8	7	9", 14", 101
H"	1.857	11.60(9"), 11.80(8'), 8.20(9'), 1.70(14')	9', 9", 14'	8	7, 9, 10, 14	10, 101
9 C	27.376			9', 9"	8", 10, 11', 11"	
H'	0.649	8.20(8"), 13.10(9"), 7.30(10)	8', 8", 9", 10	9	7, 8, 10, 11	14"
H"	1.724	11.60(8"), 10.50(10), 7.80(8'), 13.10(9')	8', 8", 9', 10, 11'	9	6, 7, 10	8', 10

Atom	δ (ppm)	J	COSY	HSQC	HMBC	NOESY
10 C	37.781			10	5', 5", 8", 9', 9", 11', 11", 14', 14"	
H	2.513	5.20(11'), 9.20(11"), 10.50(9"), 7.30(9'), 0.80(14')	9', 9", 11', 14'	10	6, 9, 14	4, 5', 8", 9"
11 C	36.947			11', 11"	9'	
H'	1.970	5.20(10), 14.70(11")	9", 10, 11', 11"	11	6, 9, 10, 12	14"
H"	2.409	9.20(10), 14.70(11')	11', 11"	11	6, 9, 10, 12	4, 5", 14"
12 C	173.942				11', 11", 13	
13 C	51.563			13		
H3	3.615			13	12	
14 C	18.052			14', 14"	5', 8", 10	
H'	0.249	1.70(8"), 0.80(10), 5.70(14")	8", 10, 14"	14	5, 6, 7, 8, 10	3, 4, 5', 5", 14", 102
H"	0.595	5.70(14')	14'	14	5, 6, 7, 8, 10	8', 9', 11', 11", 14'
100 Si	18.881				101, 102, 104	
101 C	-3.338			101	102	
H3	0.102			101	100, 102, 103	8', 8"
102 C	-3.855			102	101	
H3	0.071			102	100, 101, 103	14'
103 C	18.060				101, 102, 104	
104 C	25.874			104	104	
H3	0.869			104	100, 103, 104	

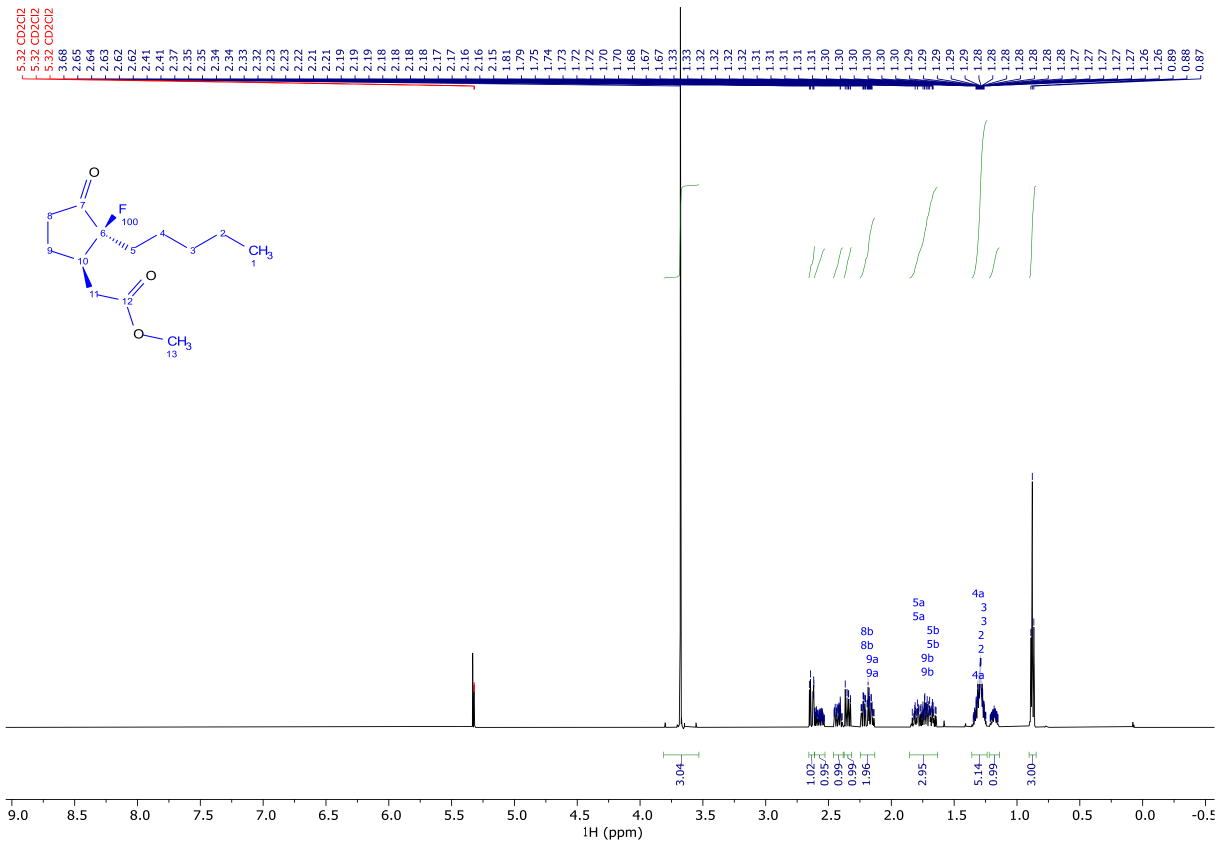

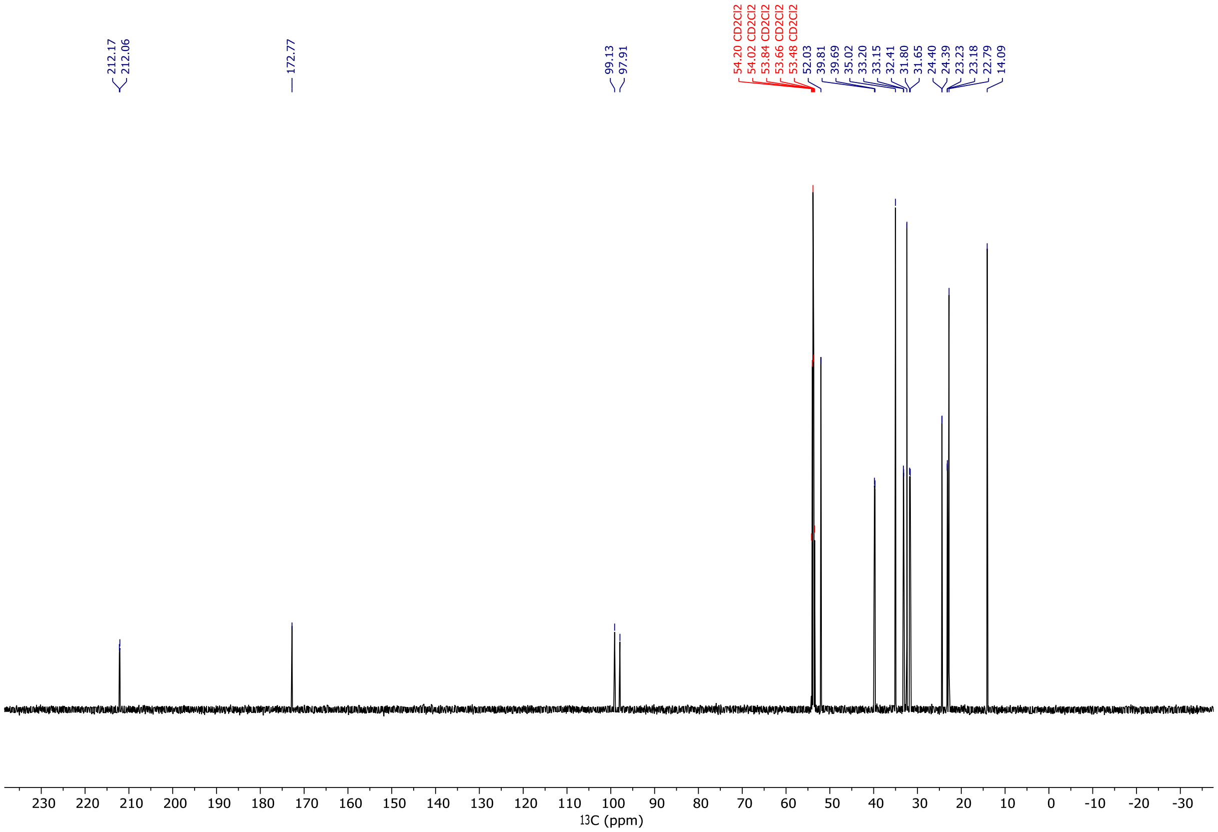

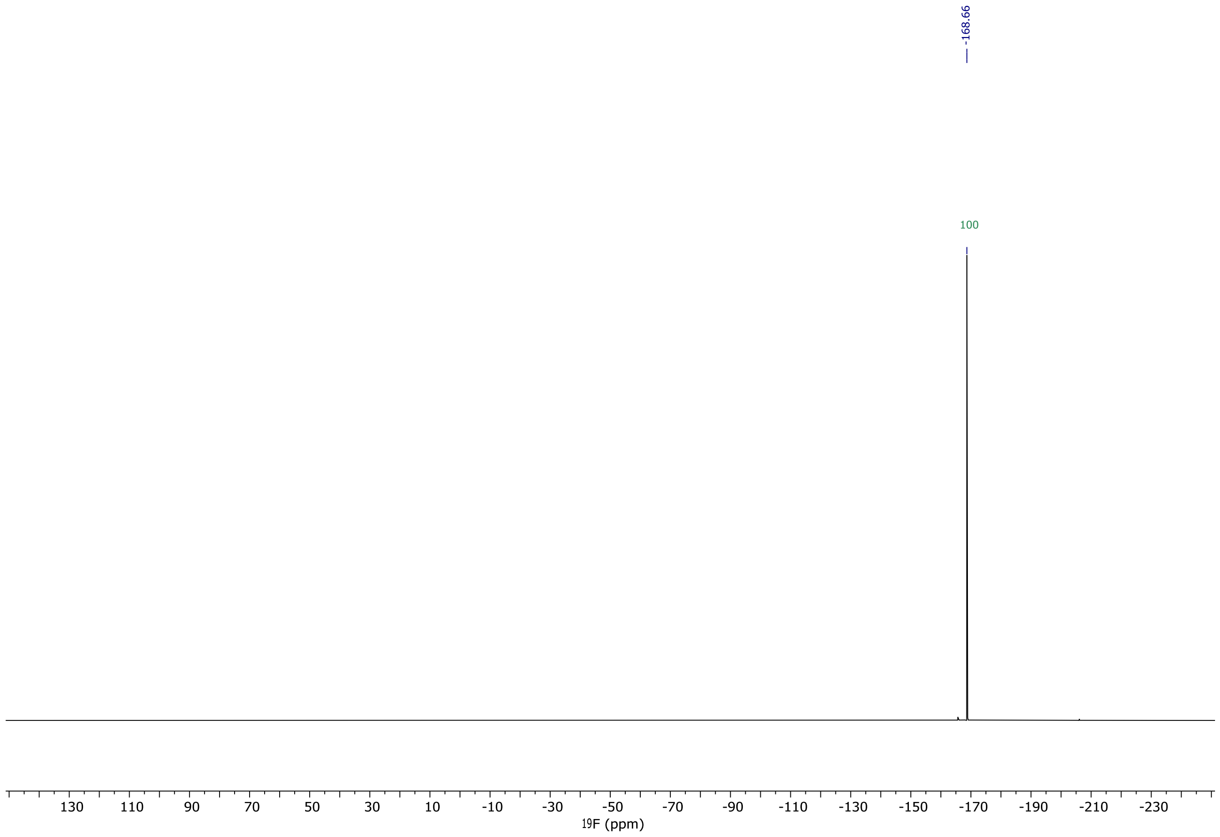

NOESY spectra of 11

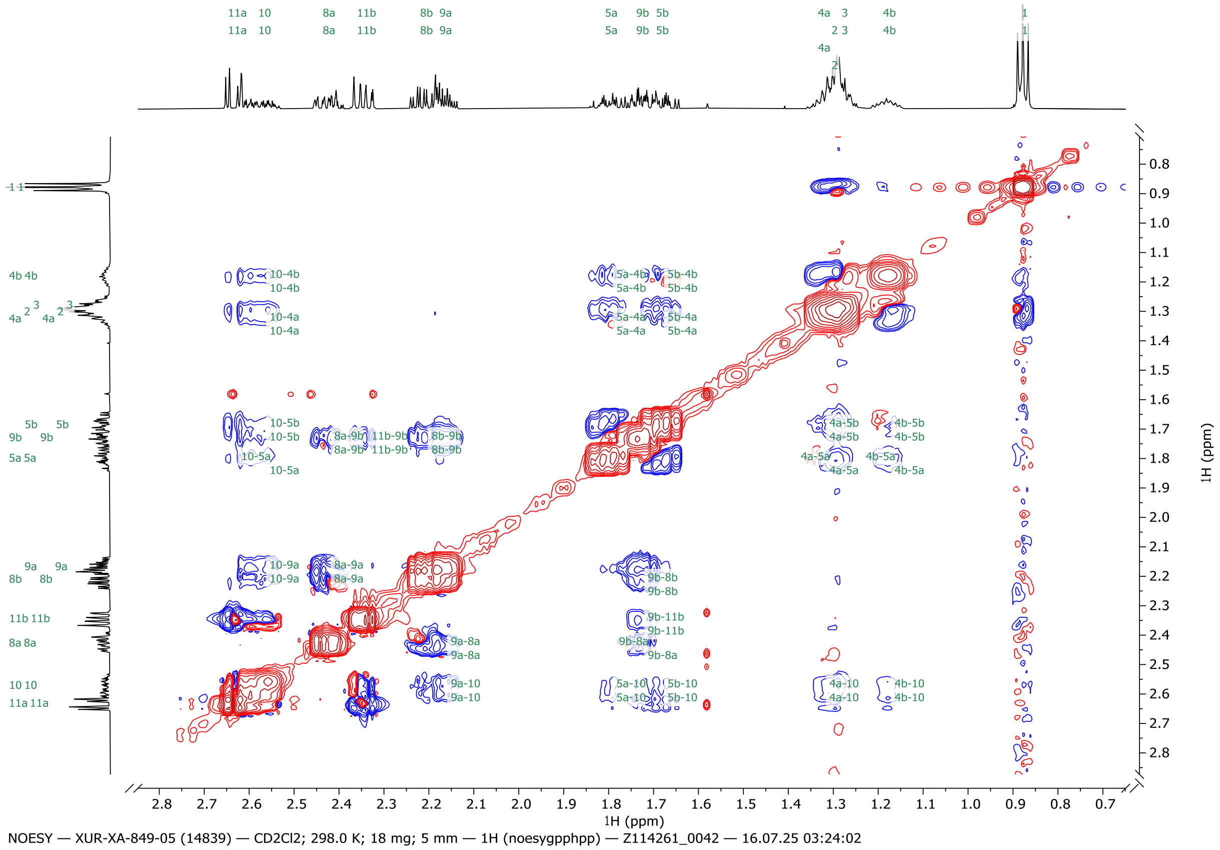

HMBC spectra of 11

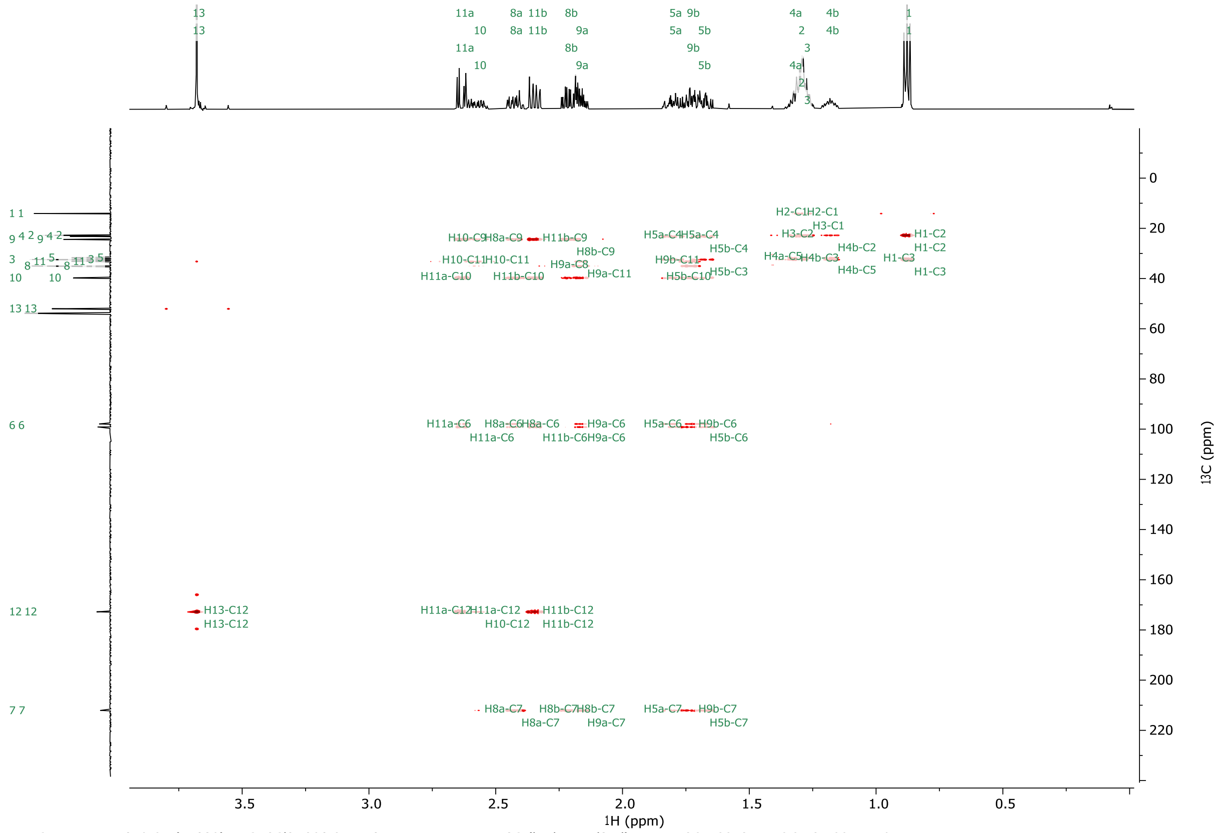

HMBC — XUR-XA-849-05 (14839) — CD2Cl2; 298.0 K; 18 mg; 5 mm — 1H-13C (hmbcetgpl3nd) — Z114261_0042 — 16.07.25 00:11:19

HSQC spectra of 11

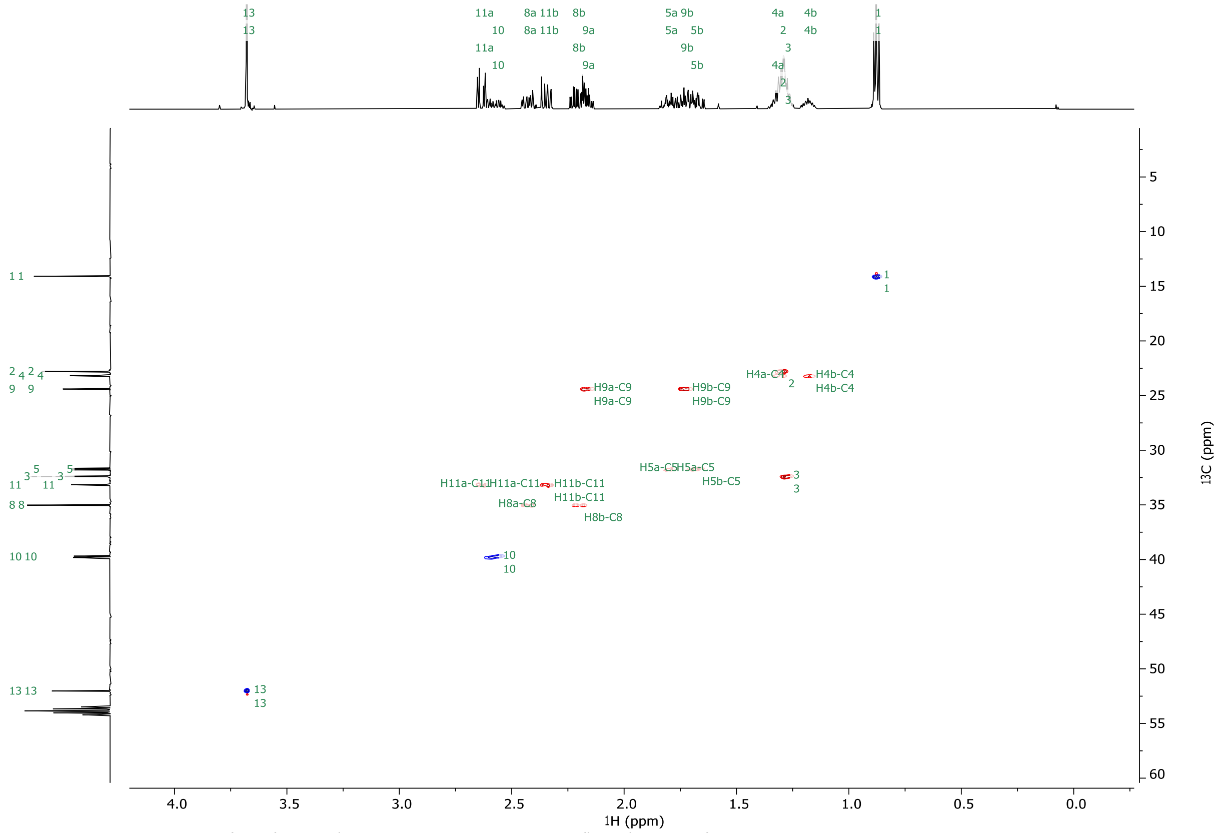

HSQC-EDITED — XUR-XA-849-05 (14839) — CD2Cl2; 298.0 K; 18 mg; 5 mm — 1H-13C (hsqcedetgpsisp2.3) — Z114261_0042 — 15.07.25 22:26:57

COSY spectra of 11

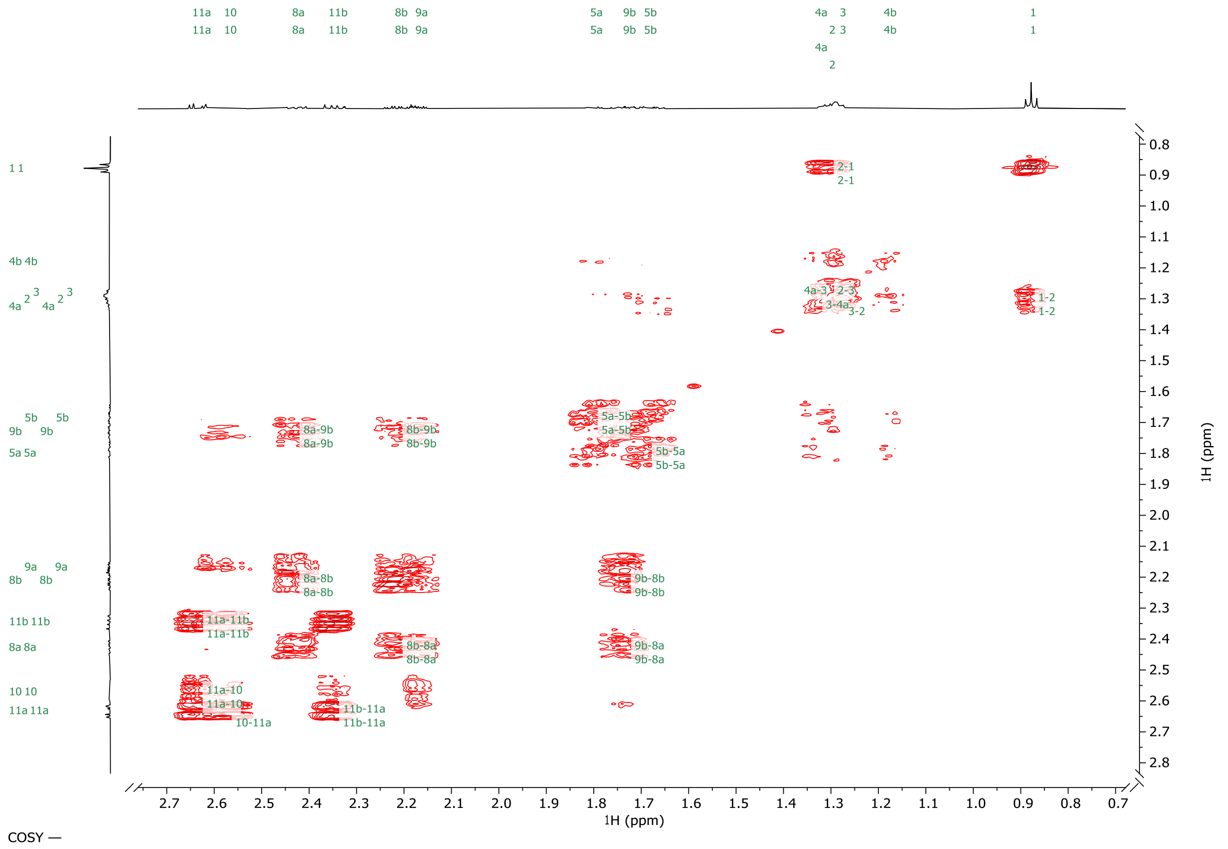

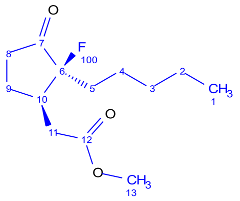

Atom	δ (ppm)	Predicted Shift	J	COSY	HSQC	HMBC	NOESY
1 C	14.088	14.10			1	2, 3	
H3	0.878	0.89		2	1	2, 3	
2 C	22.789	22.64			2	1, 3, 4a, 4b	
H2	1.301	1.30, 1.31		1, 3	2	1, 3, 4	
3 C	32.409	31.48			3	1, 2, 4a, 4b, 5a, 5b	
H2	1.279	1.28, 1.32		2, 4a	3	1, 2, 5	
4 C	23.206	22.20	7.8(100)		4a, 4b	2, 5a, 5b	
Ha	1.325	1.36, 1.49		3	4	2, 3, 5	5a, 5b, 10
Hb	1.179	1.36, 1.49			4	2, 3, 5	5a, 5b, 10
5 C	31.727	32.28	23.4(100)		5a, 5b	3, 4a, 4b	
Ha	1.799	1.82, 2.07		5b	5	3, 4, 6, 7, 10	4a, 4b, 10
Hb	1.685	1.82, 2.07		5a	5	3, 4, 6, 7, 10	4a, 4b, 10
6 C	98.519	102.91	183.7(100)			5a, 5b, 8a, 9a, 9b, 11a, 11b	
7 C	212.115	211.84	16.6(100)			5a, 5b, 8a, 8b, 9a, 9b	
8 C	35.021	37.80			8a, 8b	9a, 9b	
Ha	2.427	2.42, 2.55		8b, 9b	8	6, 7, 9, 10	9a, 9b
Hb	2.210	2.42, 2.55		8a, 9b	8	7, 9, 10	9b
9 C	24.399	28.57	1.5(100)		9a, 9b	8a, 8b, 10, 11a, 11b	
Ha	2.167	1.84, 2.09			9	6, 7, 8, 10, 11	8a, 10
Hb	1.729	1.84, 2.09		8a, 8b	9	6, 7, 8, 10, 11	8a, 8b, 11b
10 C	39.749	42.67	19.0(100)		10	5a, 5b, 8a, 8b, 9a, 9b, 11a, 11b	
H	2.570	2.30	8.9(11b), 5.0(11a)	11a	10	9, 11, 12	4a, 4b, 5a, 5b, 9a
11 C	33.179	35.50	7.7(100)		11a, 11b	9a, 9b, 10	
Ha	2.631	2.43, 2.68	5.0(10), 16.0(11b)	10, 11b	11	6, 9, 10, 12	
Hb	2.344	2.43, 2.68	8.9(10), 16.0(11a)	11a	11	6, 9, 10, 12	9b
12 C	172.769	172.94				10, 11a, 11b, 13	
13 C	52.029	51.79			13		
H3	3.680	3.63			13	12	
100 F	-168.661		16.6(7), 183.7(6), 19.0(10), 7.7(11), 23.4(5), 1.5(9), 7.8(4)				

remarks:

-The evaluation supports the specified structure.
The following 2D correlations support the proposed structure:

NOE: H_{10} - H_5,H_4
HOESY: F_{100} - H_{13}, H_{11a}+H_{11b}

A comparison was also made with NMR analysis 12542, which also supports the proposed structure.

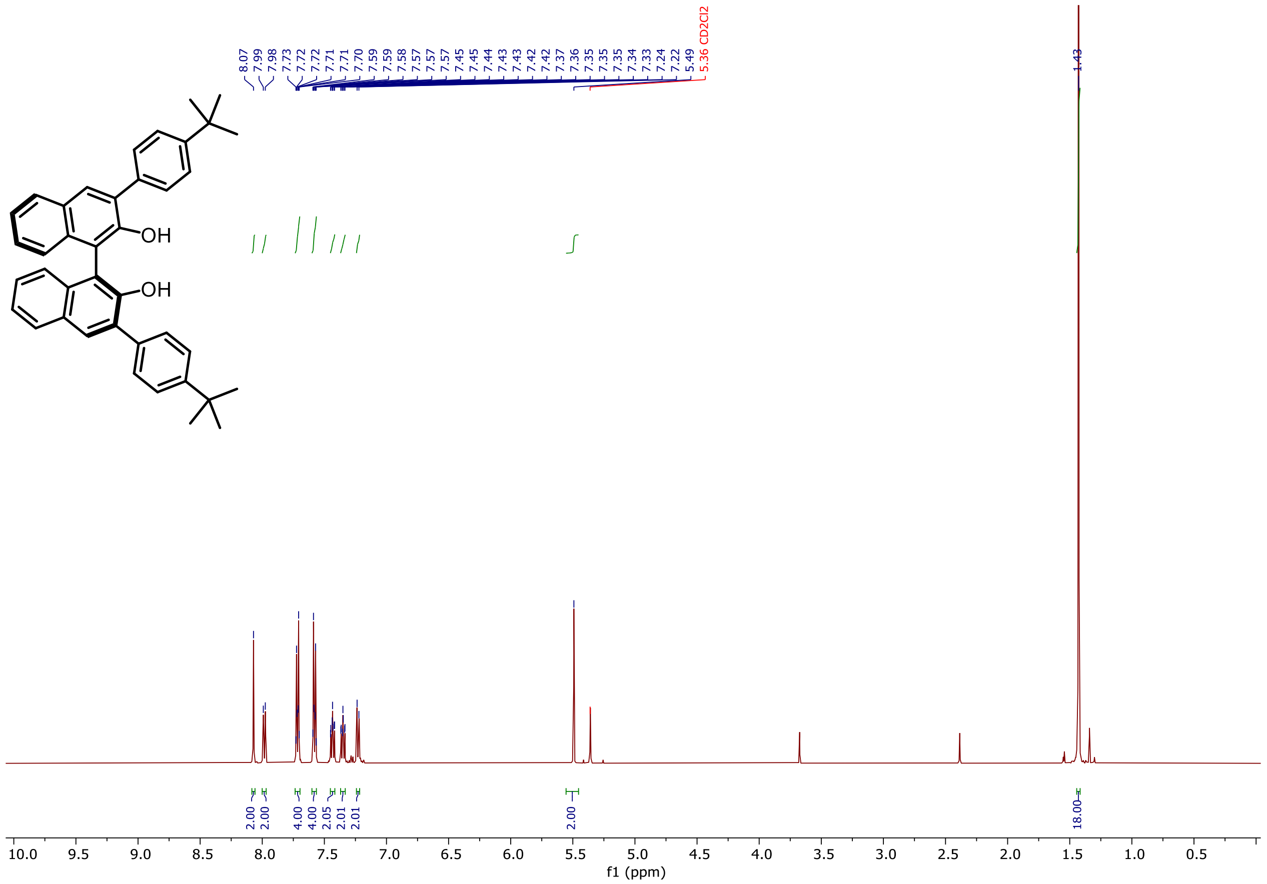
8.07
7.99
7.98
7.73
7.72
7.72
7.71
7.71
7.70
7.59
7.59
7.58
7.57
7.57
7.57
7.45
7.45
7.44
7.43
7.43
7.42
7.37
7.36
7.35
7.35
7.35
7.34
7.33
7.24
7.22
5.49
5.36 CD2Cl2
1.43
OH
OH
2.00
2.00
4.00
4.00
2.05
2.01
2.01
2.00
18.00
10.0 9.5 9.0 8.5 8.0 7.5 7.0 6.5 6.0 5.5 5.0 4.5 4.0 3.5 3.0 2.5 2.0 1.5 1.0 0.5
f1 (ppm)

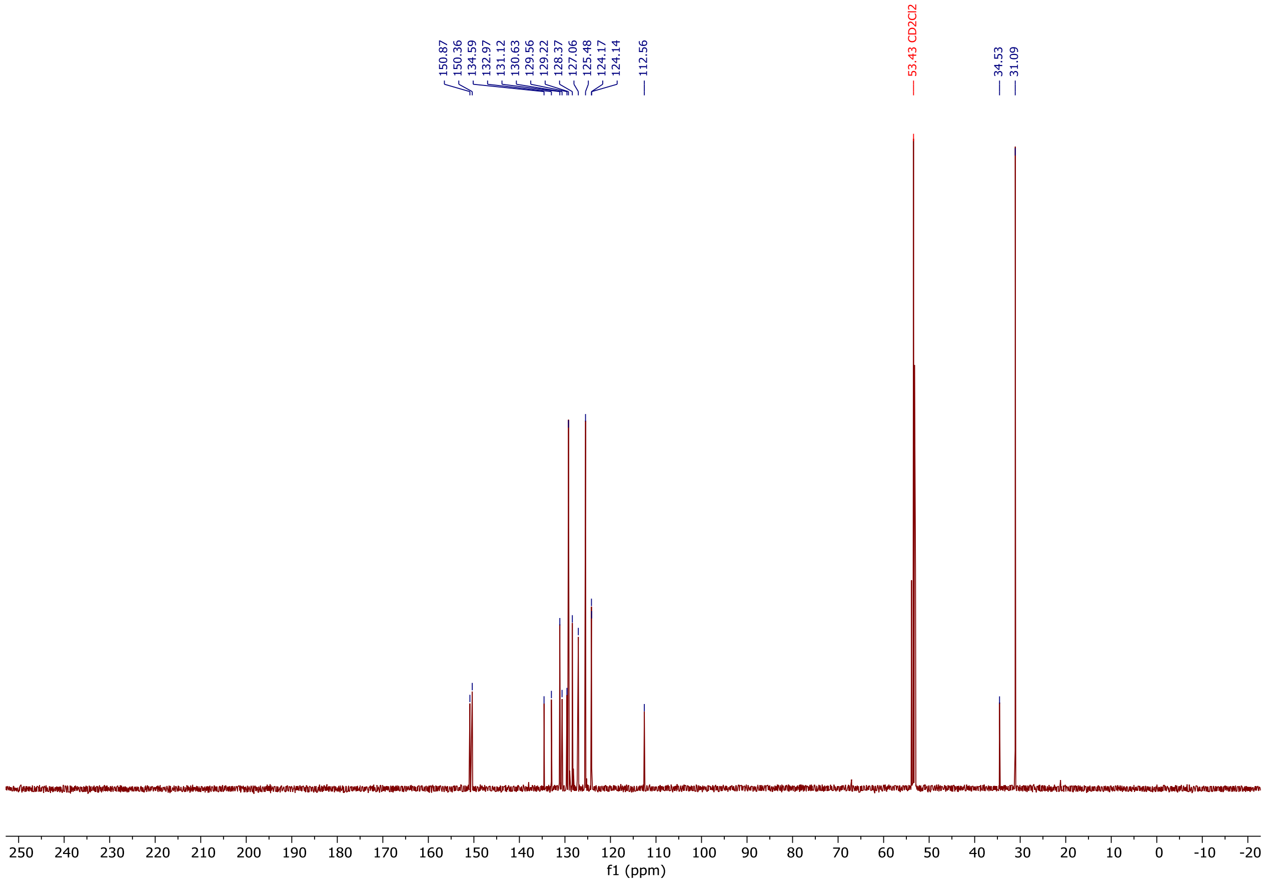
150.87
150.36
134.59
132.97
131.12
130.63
129.56
129.22
128.37
127.06
125.48
124.17
124.14
112.56
53.43 CD2Cl2
34.53
31.09
250 240 230 220 210 200 190 180 170 160 150 140 130 120 110 100 90 80 70 60 50 40 30 20 10 0 -10 -20
f1 (ppm)

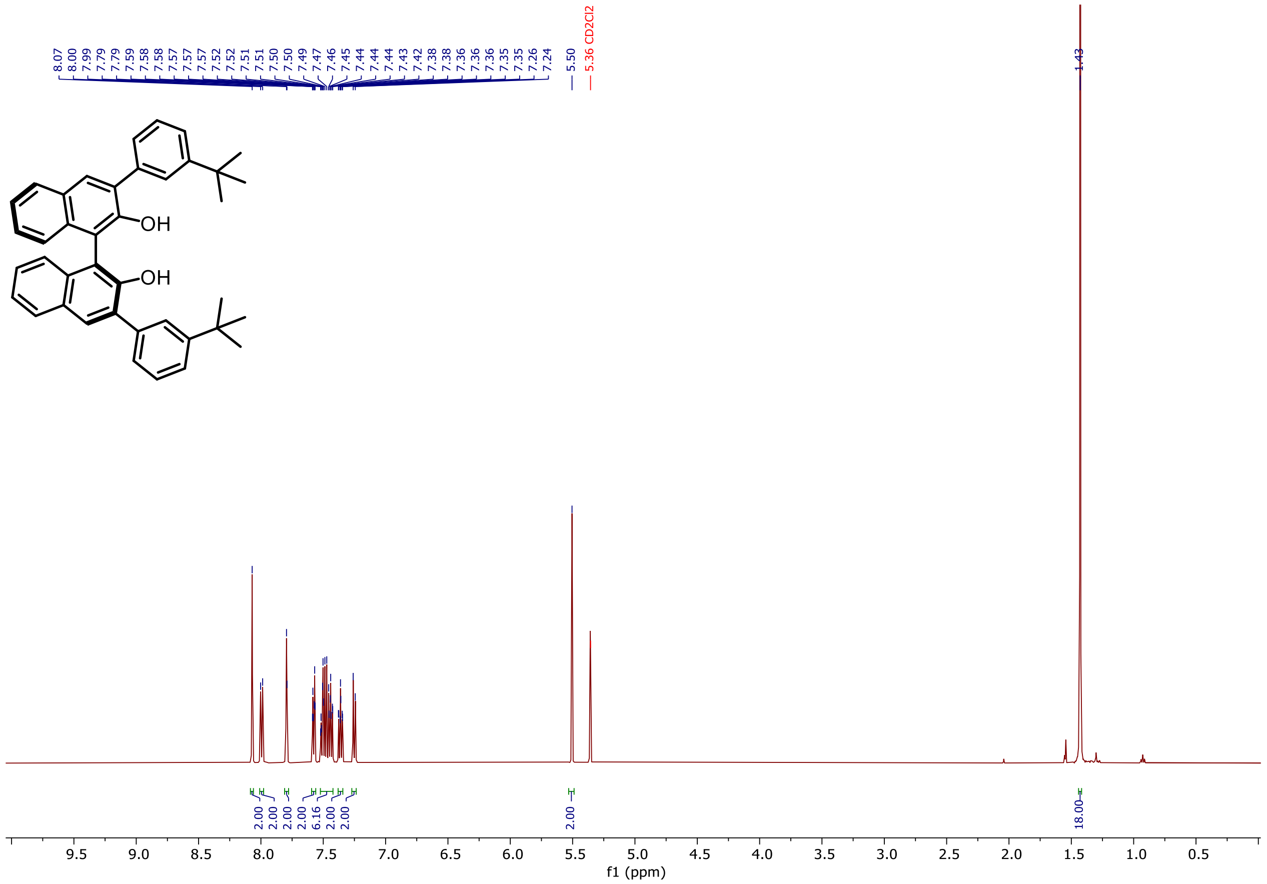
8.07
8.00
7.99
7.79
7.79
7.59
7.58
7.57
7.57
7.57
7.52
7.52
7.51
7.51
7.50
7.50
7.49
7.47
7.46
7.45
7.44
7.44
7.44
7.43
7.42
7.38
7.38
7.36
7.36
7.36
7.35
7.26
7.24
5.50
5.36 CD2Cl2
1.43
OH
OH
2.00
2.00
2.00
2.00
6.16
2.00
2.00
2.00
18.00
f1 (ppm)

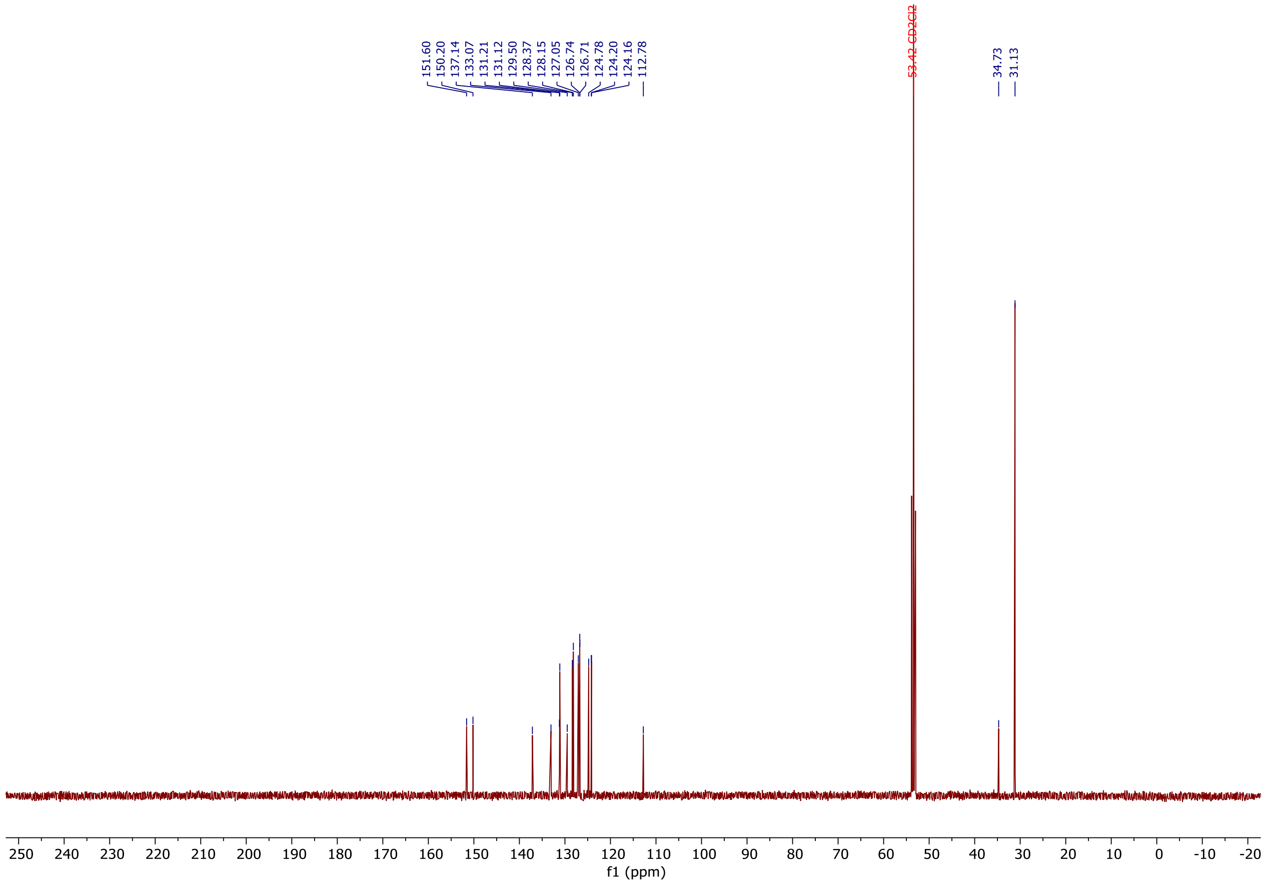
151.60
150.20
137.14
133.07
131.21
131.12
129.50
128.37
128.15
127.05
126.74
126.71
124.78
124.20
124.16
112.78
53.42 CD2Cl2
34.73
31.13
f1 (ppm)

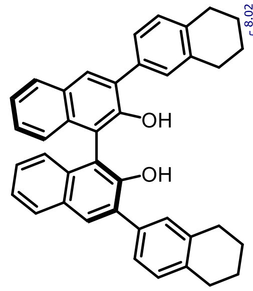
OH
OH

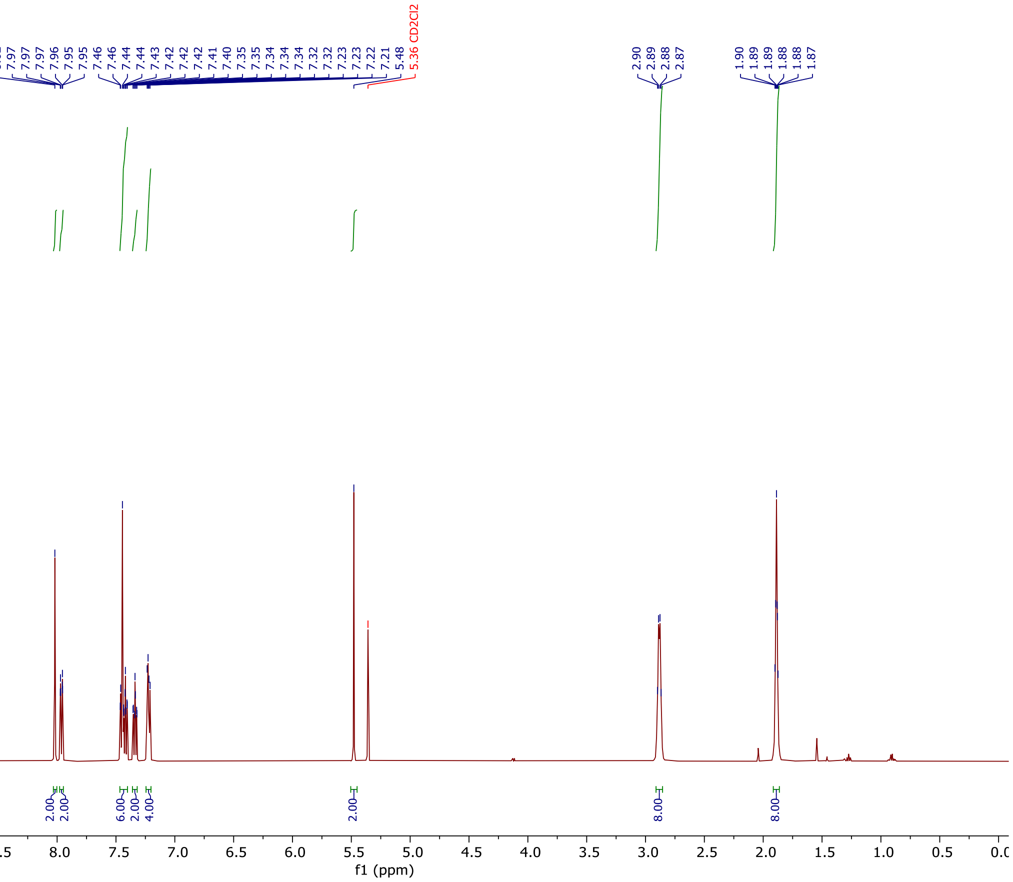
5.36 CD2Cl2
f1 (ppm)

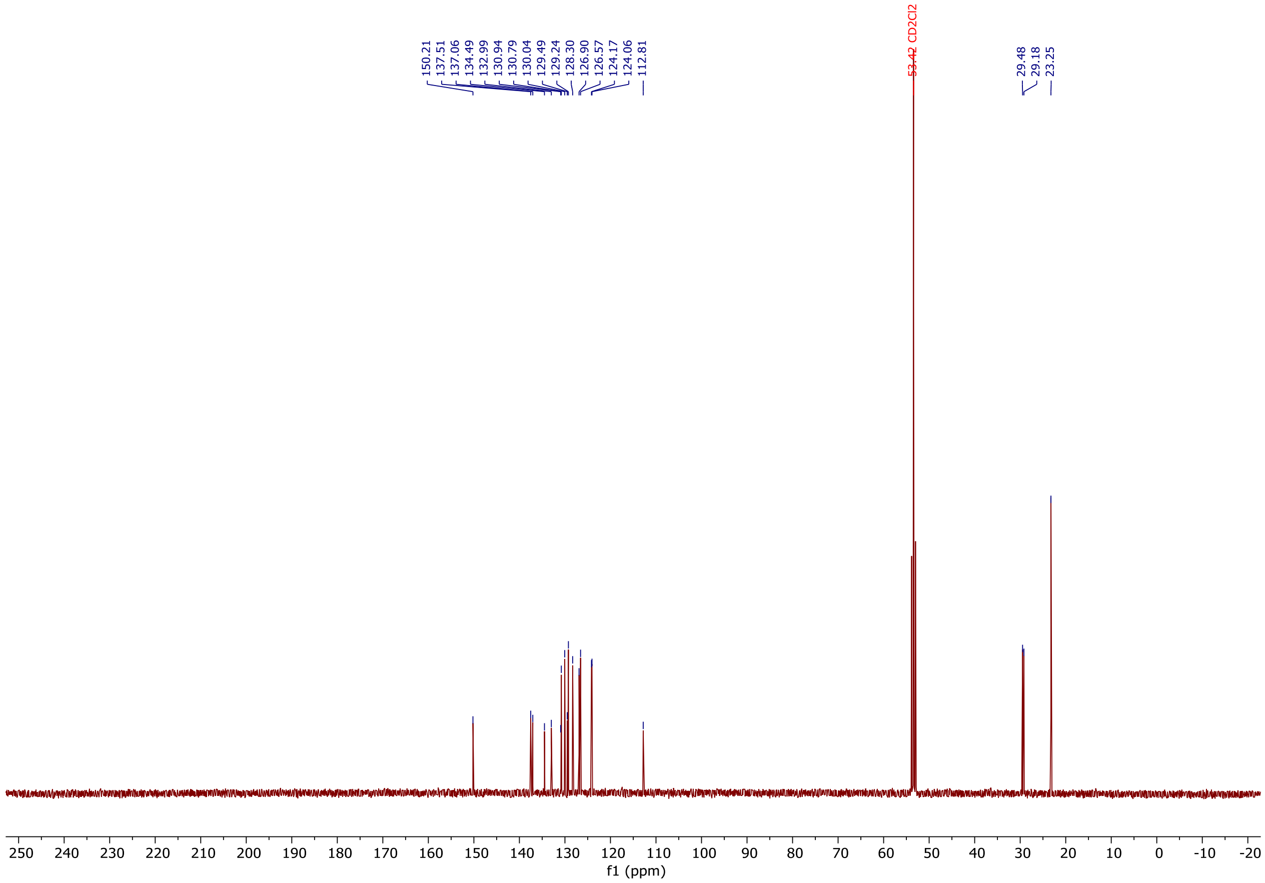
53.42 CD2Cl2
f1 (ppm)

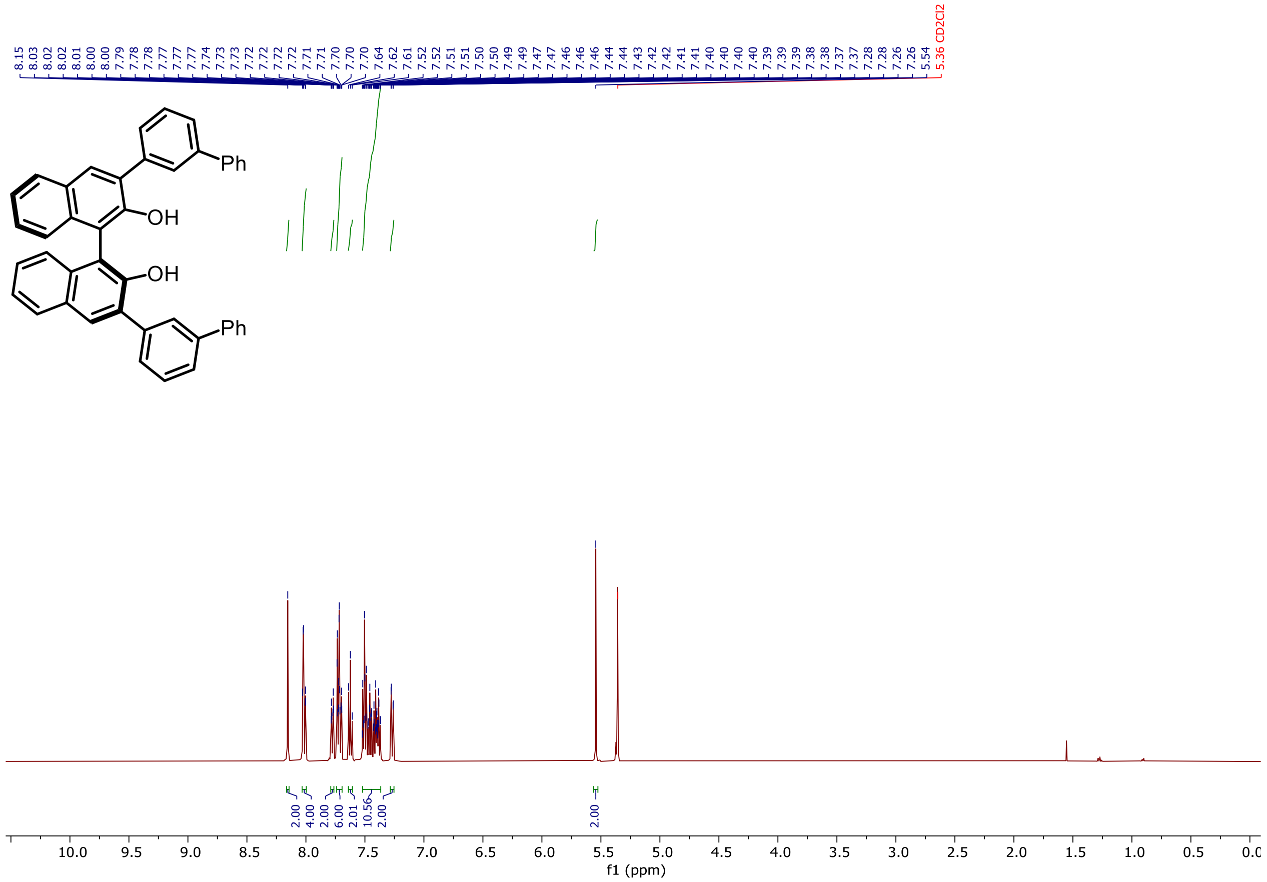

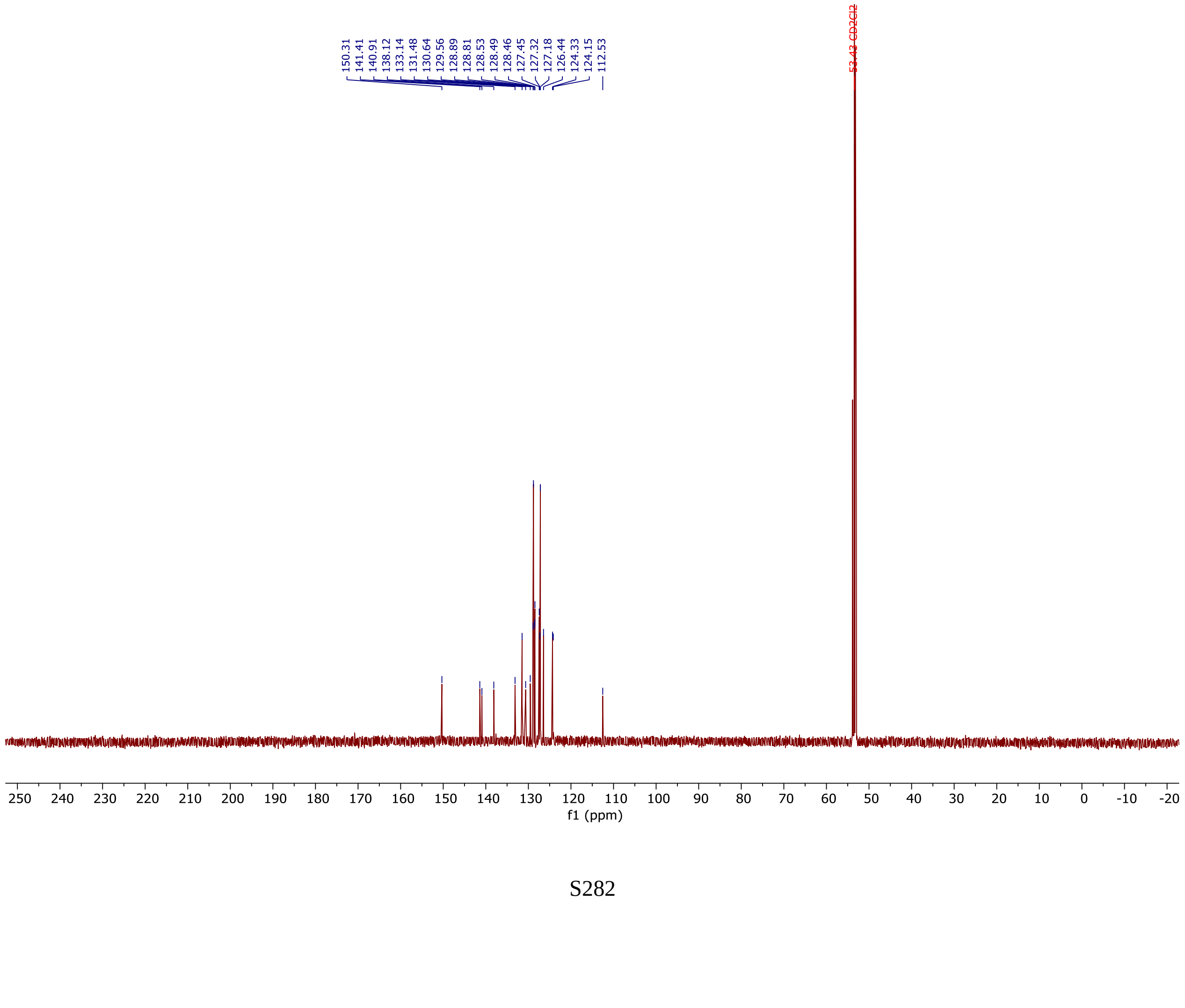

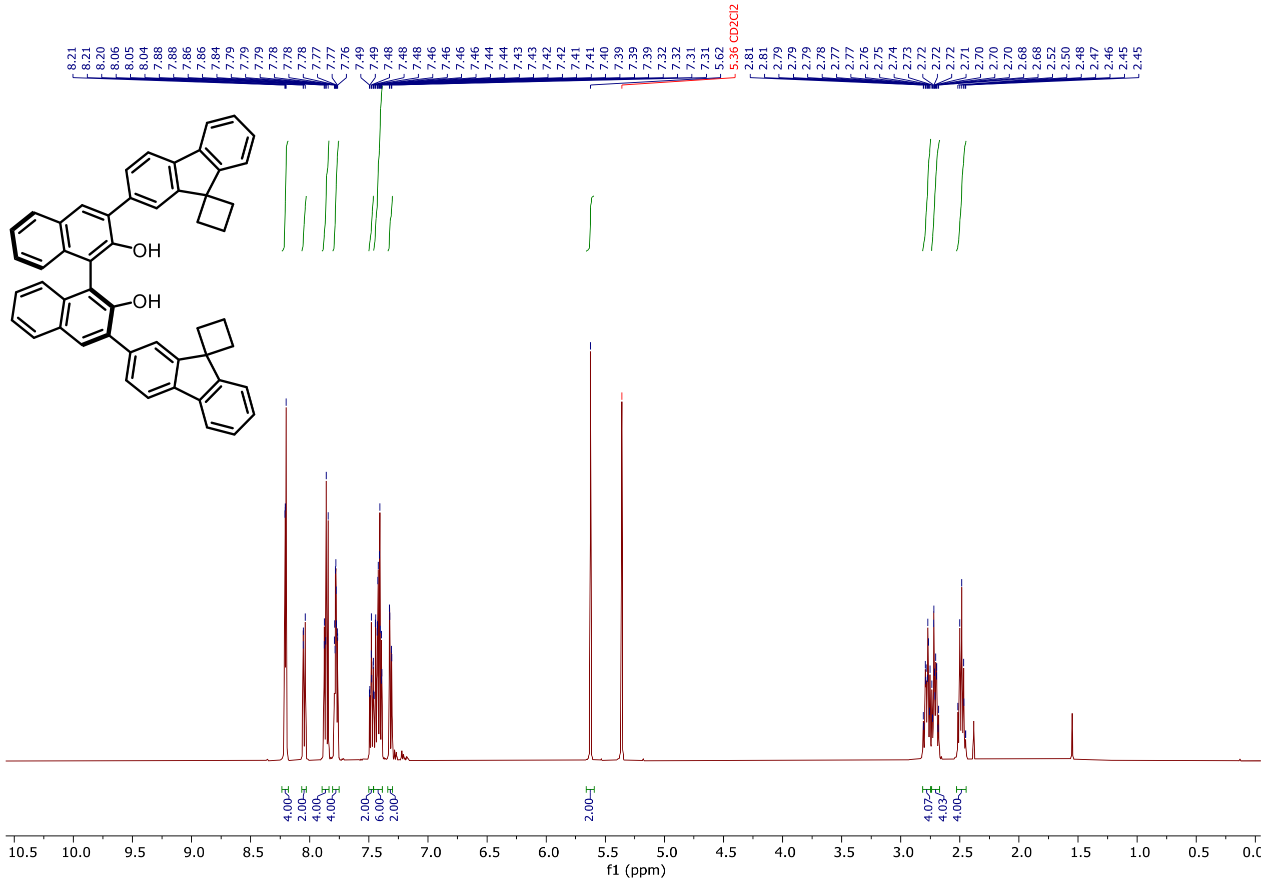
8.21 8.21 8.20 8.06 8.05 8.04 7.88 7.88 7.86 7.86 7.84 7.79 7.79 7.79 7.78 7.78 7.77 7.77 7.76 7.49 7.49 7.48 7.48 7.48 7.46 7.46 7.46 7.46 7.44 7.44 7.43 7.43 7.42 7.42 7.41 7.41 7.40 7.39 7.39 7.39 7.32 7.32 7.31 7.31 5.62
5.36 CD2Cl2
2.81 2.81 2.79 2.79 2.79 2.78 2.77 2.77 2.76 2.75 2.74 2.73 2.73 2.72 2.72 2.72 2.72 2.71 2.71 2.70 2.70 2.70 2.68 2.68 2.52 2.50 2.48 2.47 2.46 2.45 2.45
OH
OH
4.00 2.00 4.00 4.00 2.00 6.00 2.00 2.00 4.07 4.03 4.00
10.5 10.0 9.5 9.0 8.5 8.0 7.5 7.0 6.5 6.0 5.5 5.0 4.5 4.0 3.5 3.0 2.5 2.0 1.5 1.0 0.5 0.0
f1 (ppm)

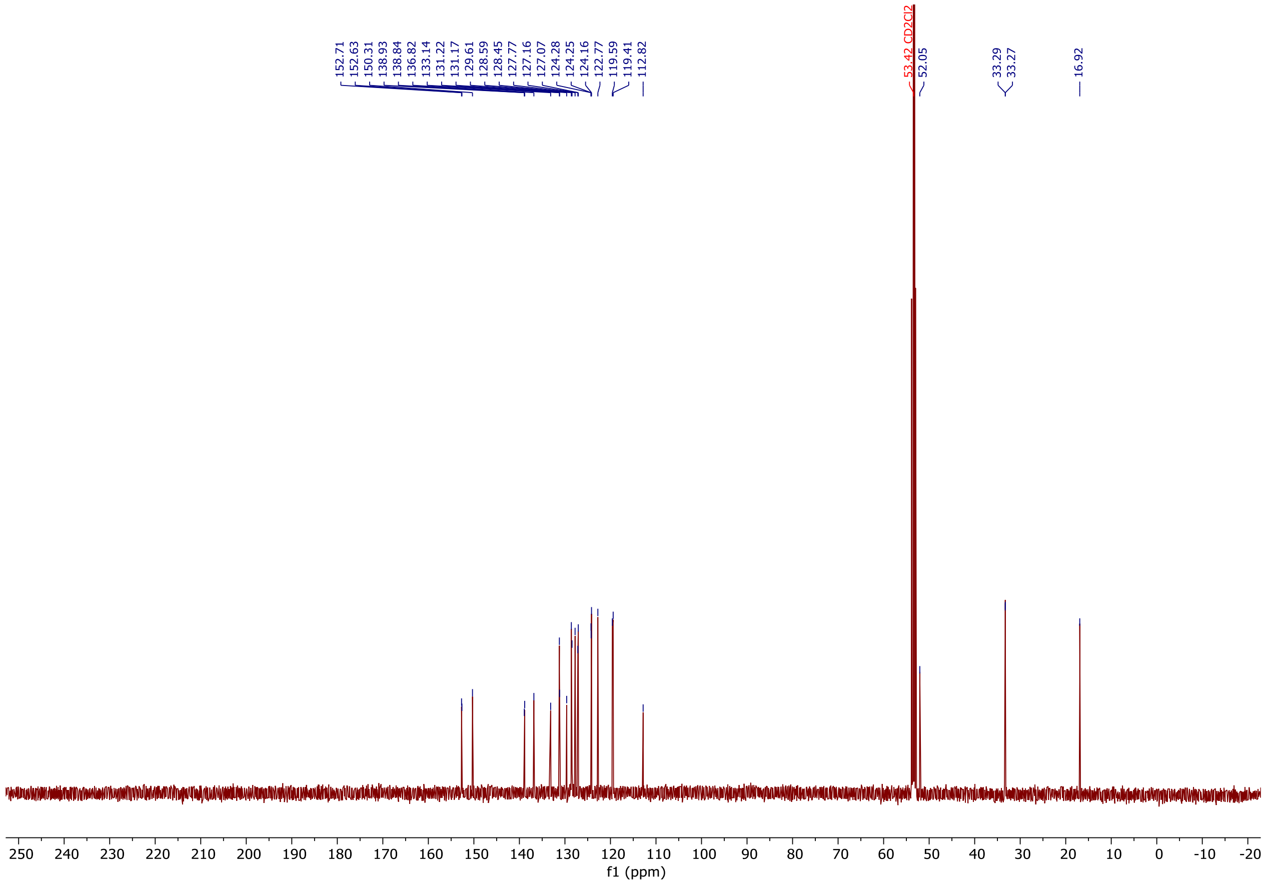
152.71 152.63 150.31 138.93 138.84 136.82 133.14 131.22 131.17 129.61 128.59 128.45 127.77 127.16 127.07 124.28 124.25 124.16 122.77 119.59 119.41 112.82
53.42 CD2Cl2
52.05
33.29 33.27
16.92
250 240 230 220 210 200 190 180 170 160 150 140 130 120 110 100 90 80 70 60 50 40 30 20 10 0 -10 -20
f1 (ppm)

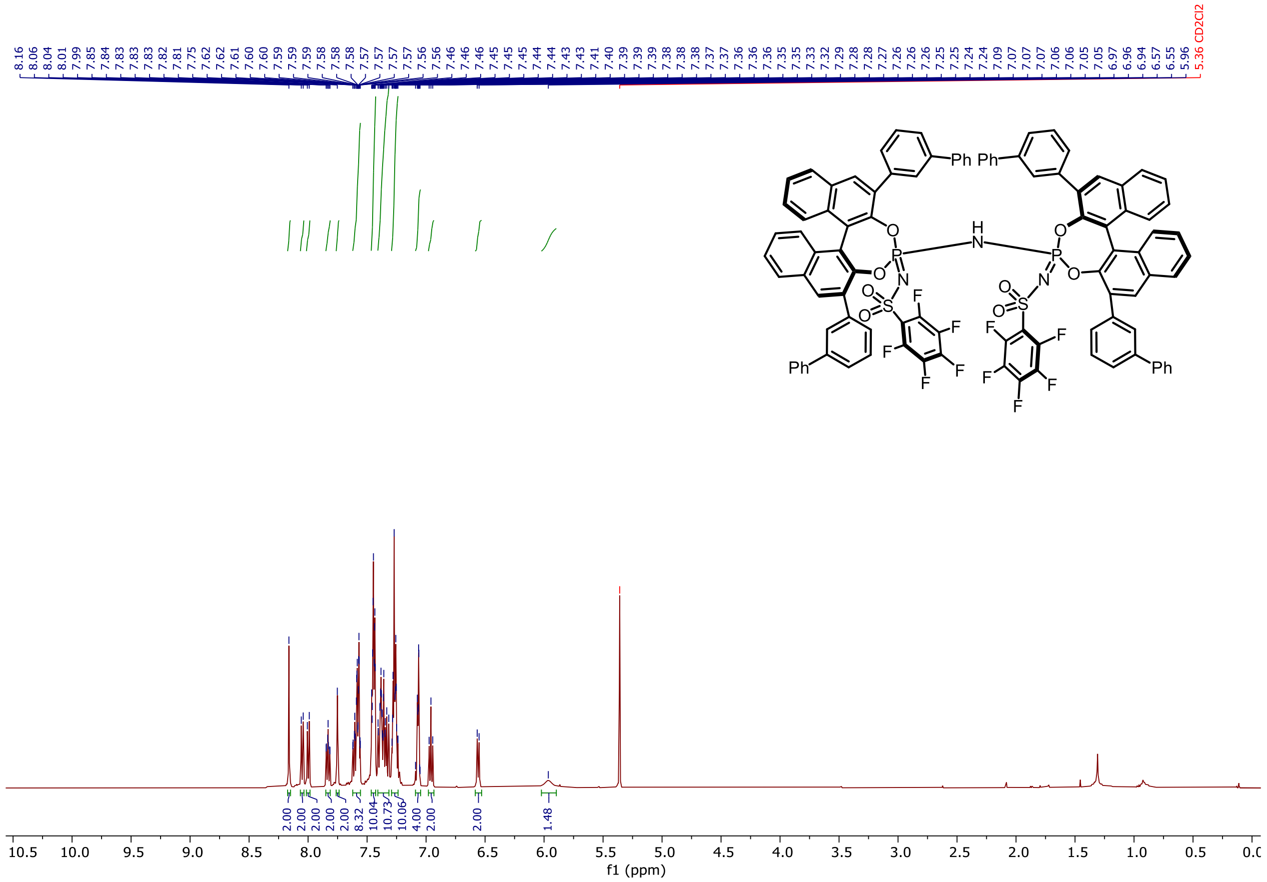

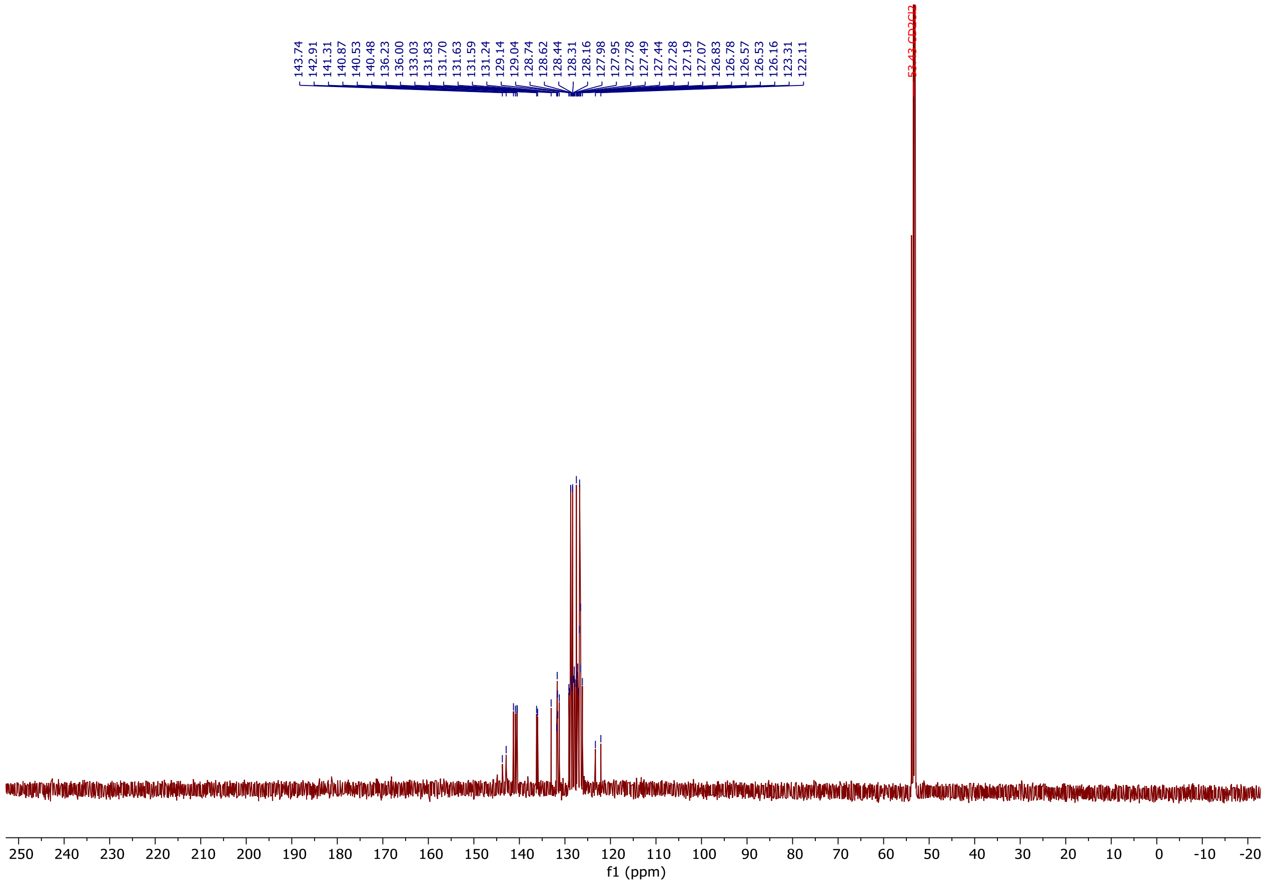

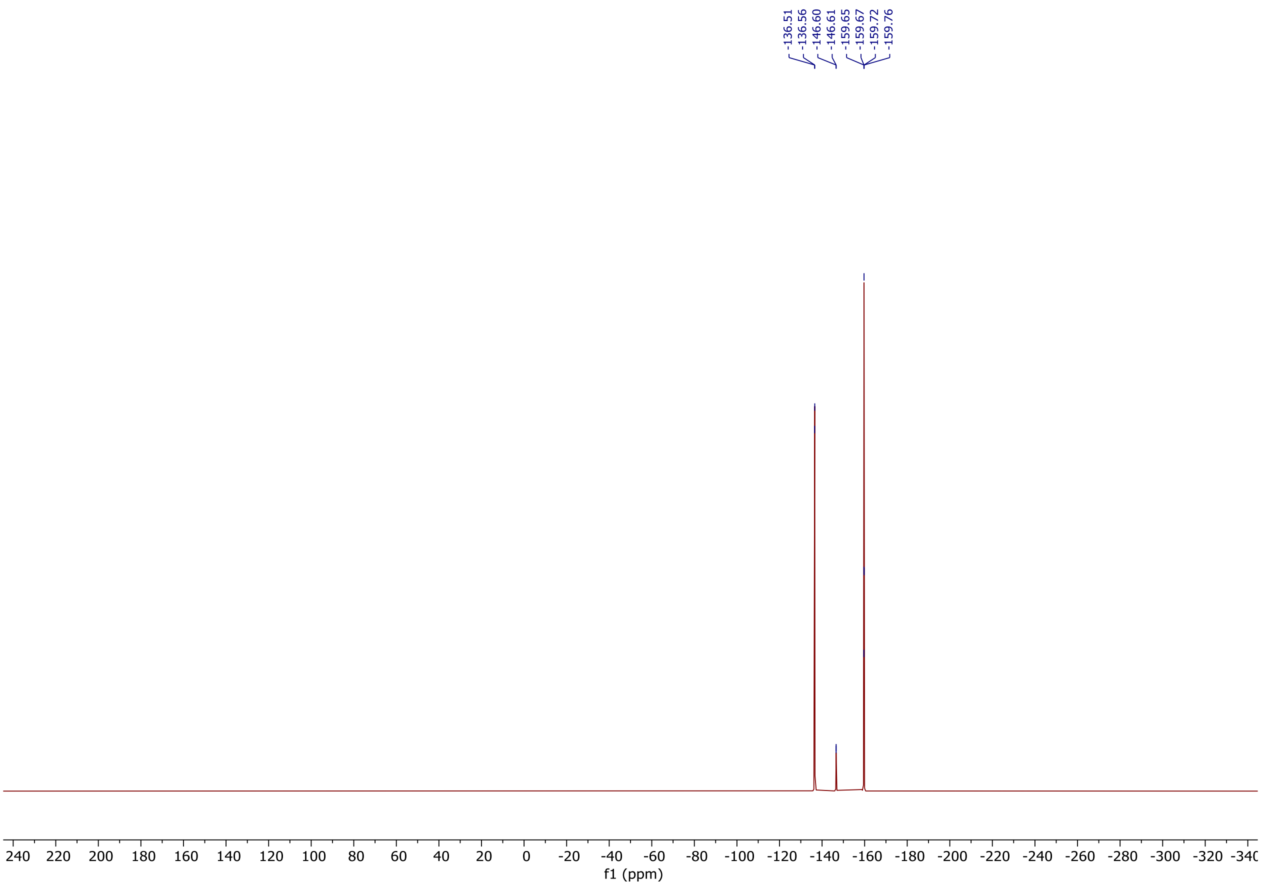
-136.51
-136.56
-146.60
-146.61
-159.65
-159.67
-159.72
-159.76
240 220 200 180 160 140 120 100 80 60 40 20 0 -20 -40 -60 -80 -100 -120 -140 -160 -180 -200 -220 -240 -260 -280 -300 -320 -340
f1 (ppm)

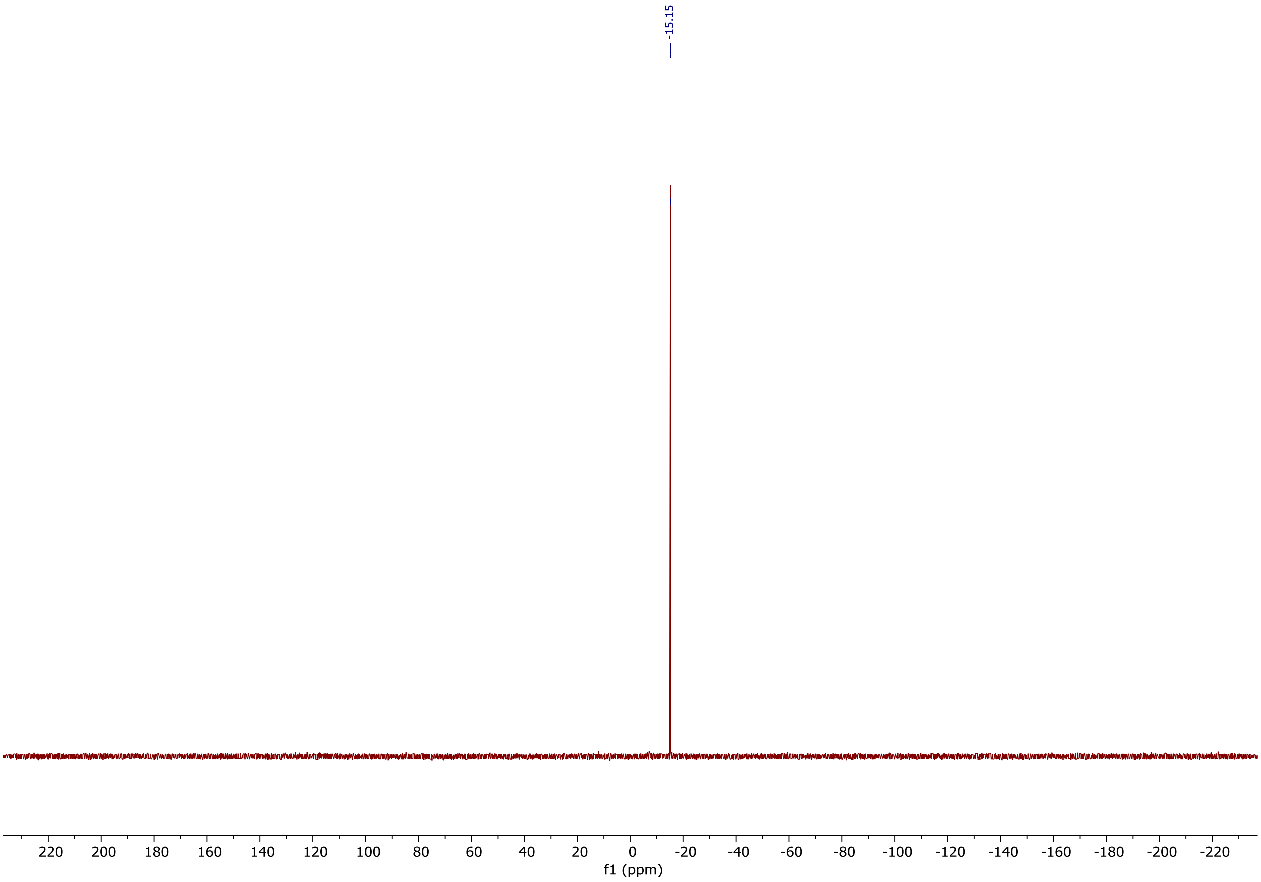
-15.15
220 200 180 160 140 120 100 80 60 40 20 0 -20 -40 -60 -80 -100 -120 -140 -160 -180 -200 -220
f1 (ppm)

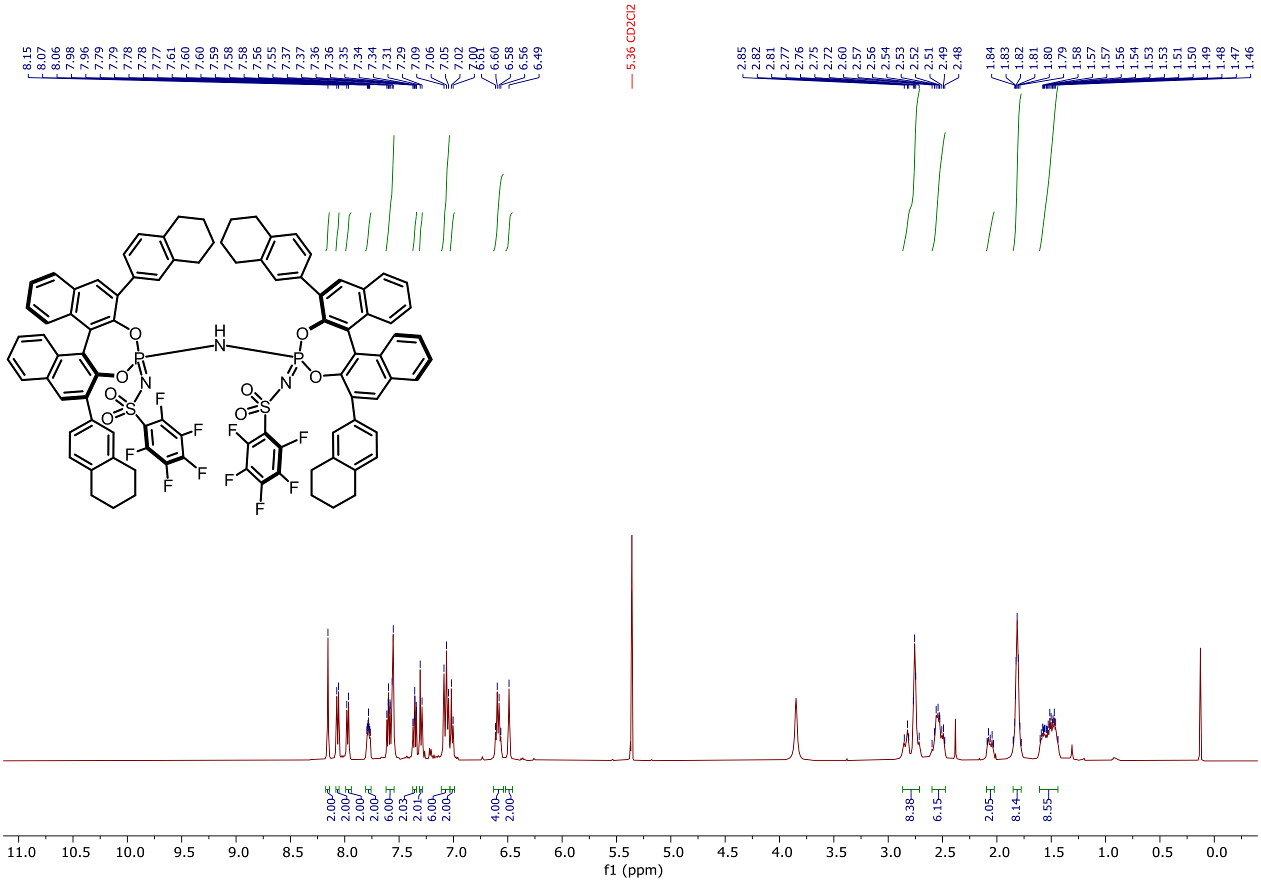
8.15 8.07 8.06 7.98 7.96 7.79 7.79 7.78 7.77 7.61 7.60 7.59 7.58 7.56 7.55 7.37 7.36 7.36 7.35 7.34 7.34 7.31 7.29 7.09 7.06 7.05 7.02 7.00 6.61 6.60 6.58 6.56 6.49
5.36 CD2Cl2
2.85 2.82 2.81 2.77 2.76 2.75 2.72 2.60 2.57 2.56 2.54 2.53 2.52 2.51 2.49 2.48
1.84 1.83 1.82 1.81 1.80 1.79 1.58 1.57 1.57 1.56 1.54 1.53 1.53 1.51 1.50 1.49 1.48 1.47 1.46
2.00 2.00 2.00 2.00 6.00 2.03 2.01 6.00 2.00 4.00 2.00
8.38 6.15 2.05 8.14 8.55
11.0 10.5 10.0 9.5 9.0 8.5 8.0 7.5 7.0 6.5 6.0 5.5 5.0 4.5 4.0 3.5 3.0 2.5 2.0 1.5 1.0 0.5 0.0
f1 (ppm)

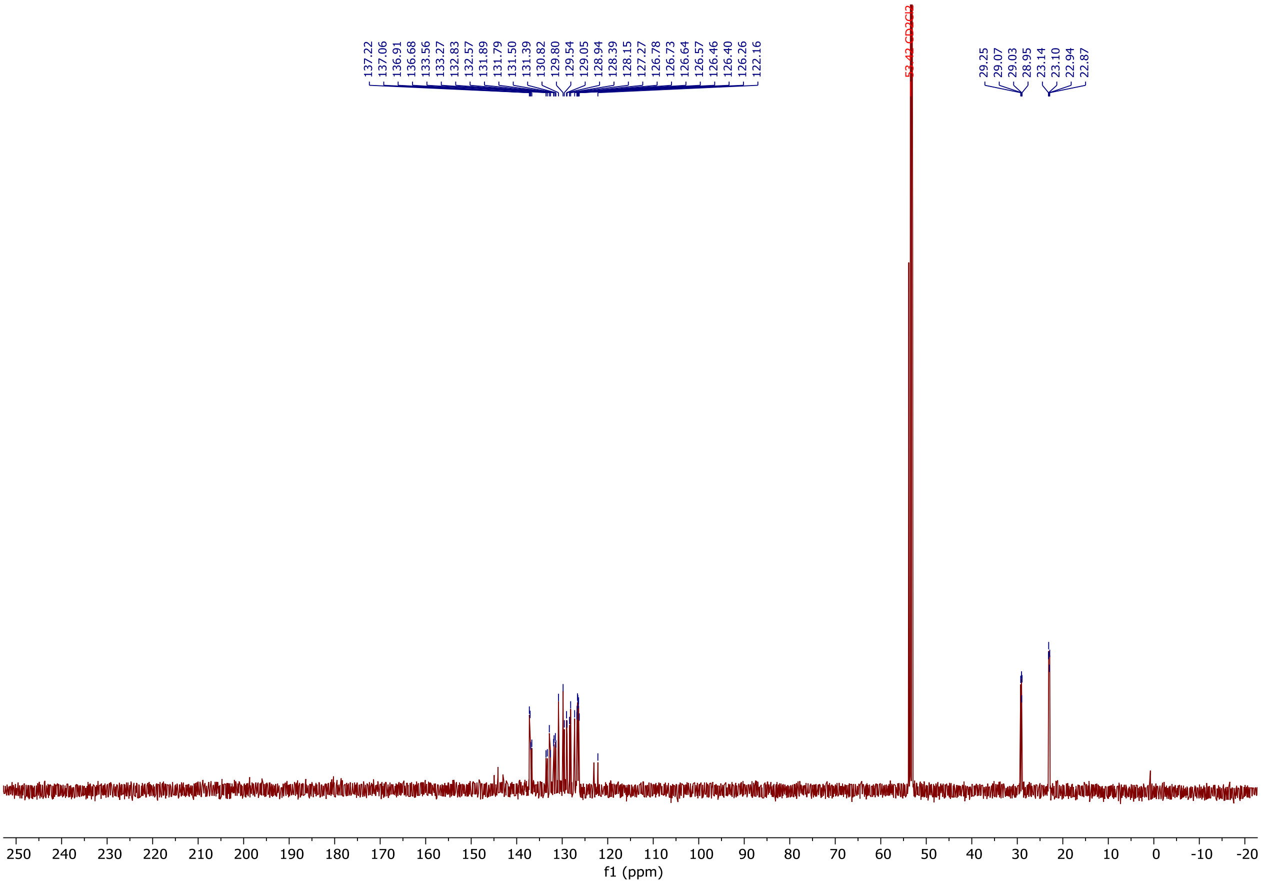
137.22 137.06 136.91 136.68 133.56 133.27 132.83 132.57 131.89 131.79 131.50 131.39 130.82 129.80 129.54 129.05 128.94 128.39 128.15 127.27 126.78 126.73 126.64 126.57 126.46 126.40 126.26 122.16
53.43 CD2Cl2
29.25 29.07 29.03 28.95 23.14 23.10 22.94 22.87
250 240 230 220 210 200 190 180 170 160 150 140 130 120 110 100 90 80 70 60 50 40 30 20 10 0 -10 -20
f1 (ppm)

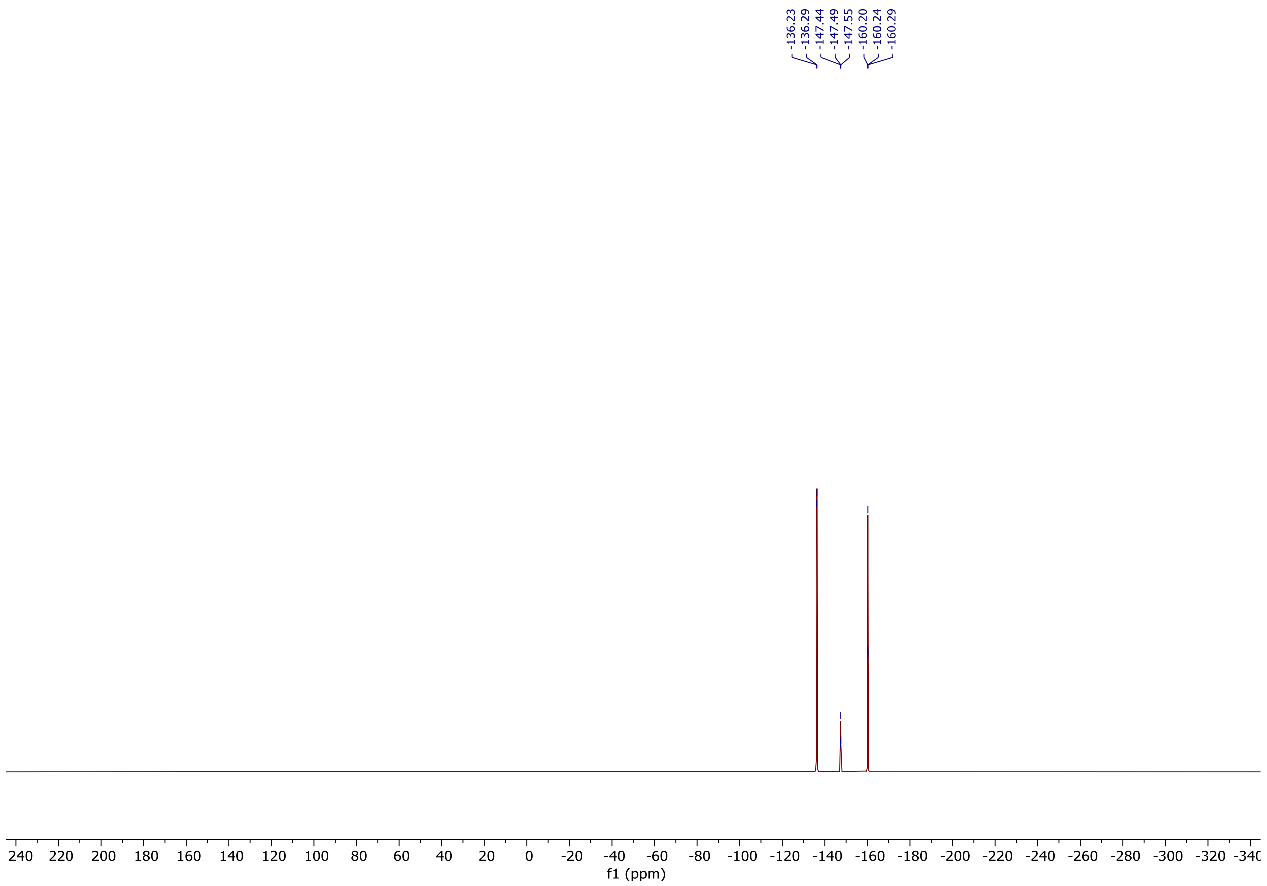
-136.23
-136.29
-147.44
-147.49
-147.55
-160.20
-160.24
-160.29
240 220 200 180 160 140 120 100 80 60 40 20 0 -20 -40 -60 -80 -100 -120 -140 -160 -180 -200 -220 -240 -260 -280 -300 -320 -340
f1 (ppm)

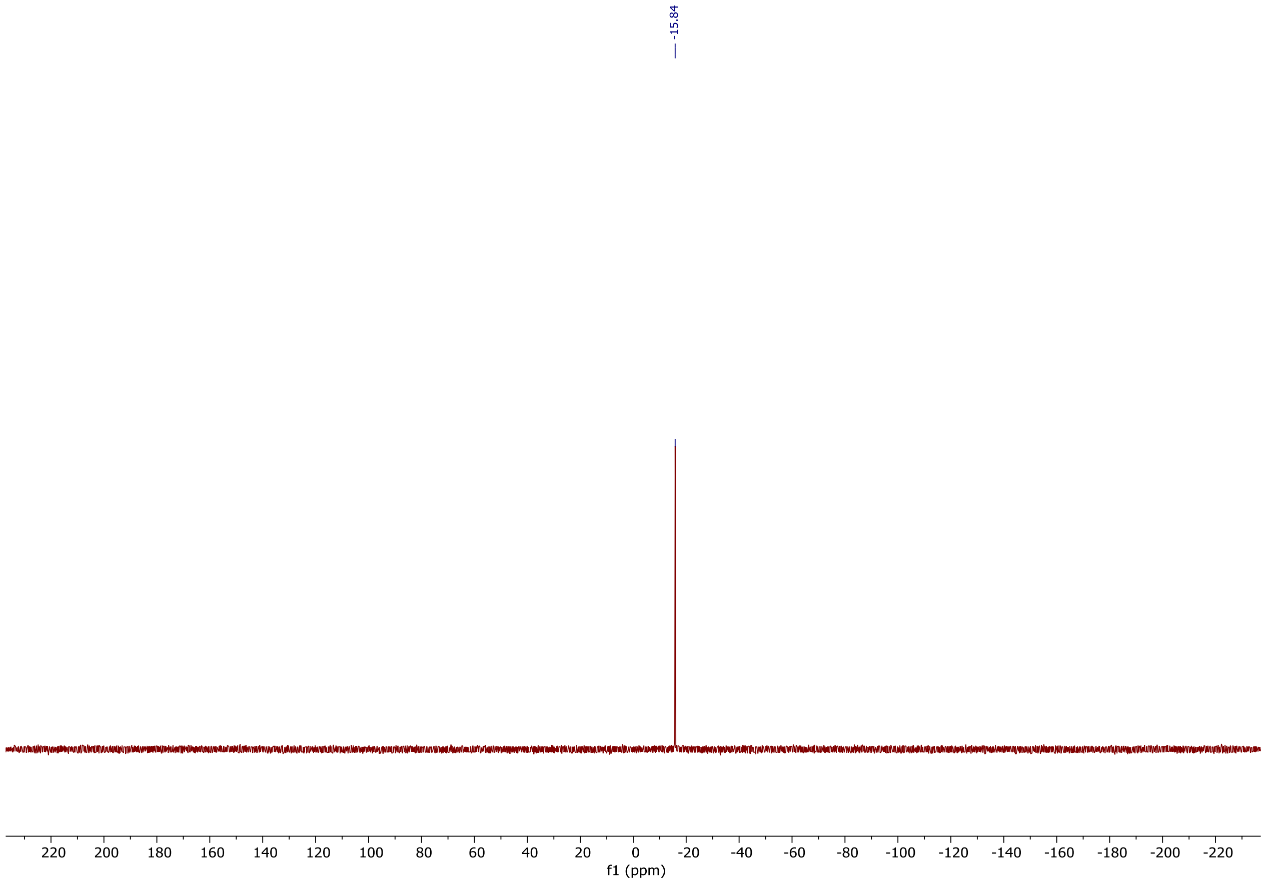
-15.84
220 200 180 160 140 120 100 80 60 40 20 0 -20 -40 -60 -80 -100 -120 -140 -160 -180 -200 -220
f1 (ppm)

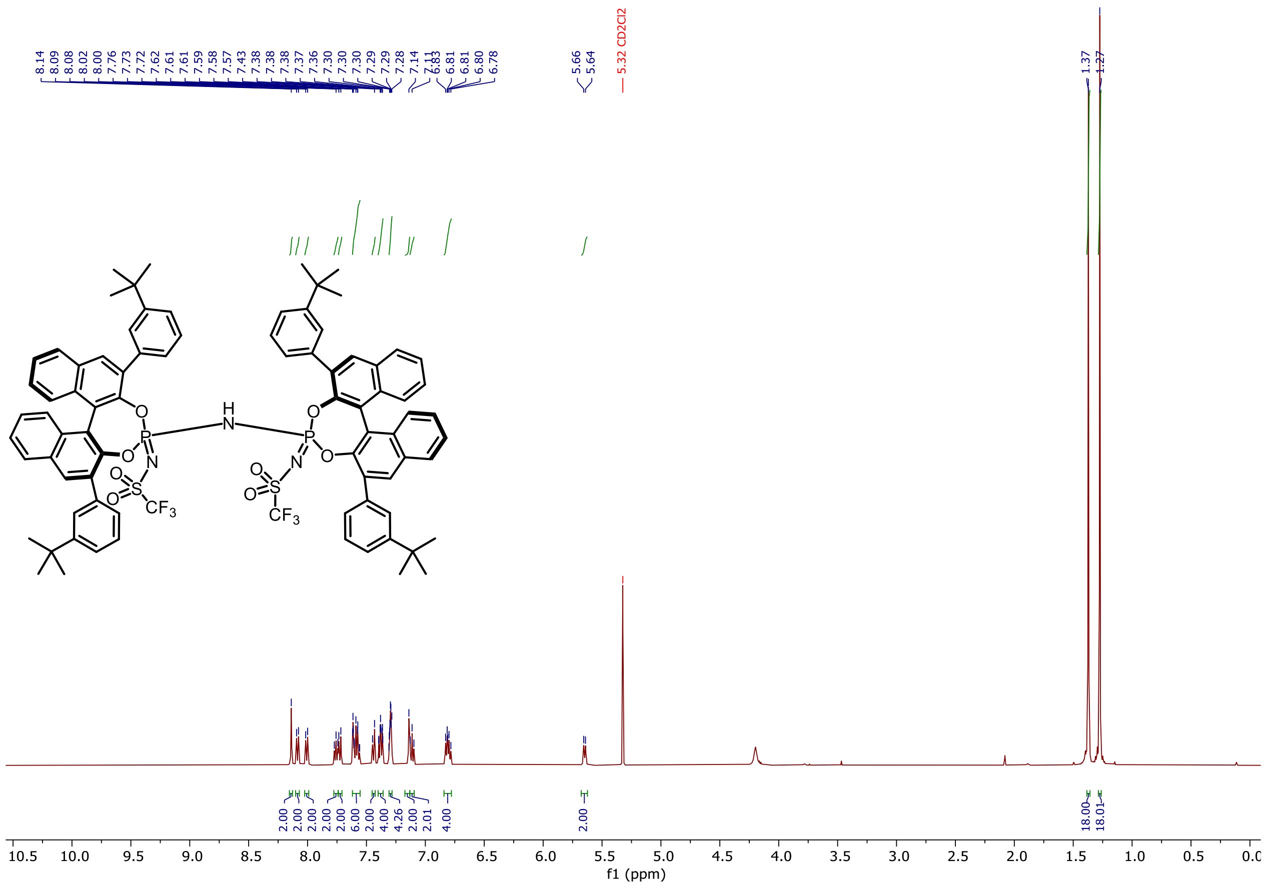
5.32 CD2Cl2
f1 (ppm)

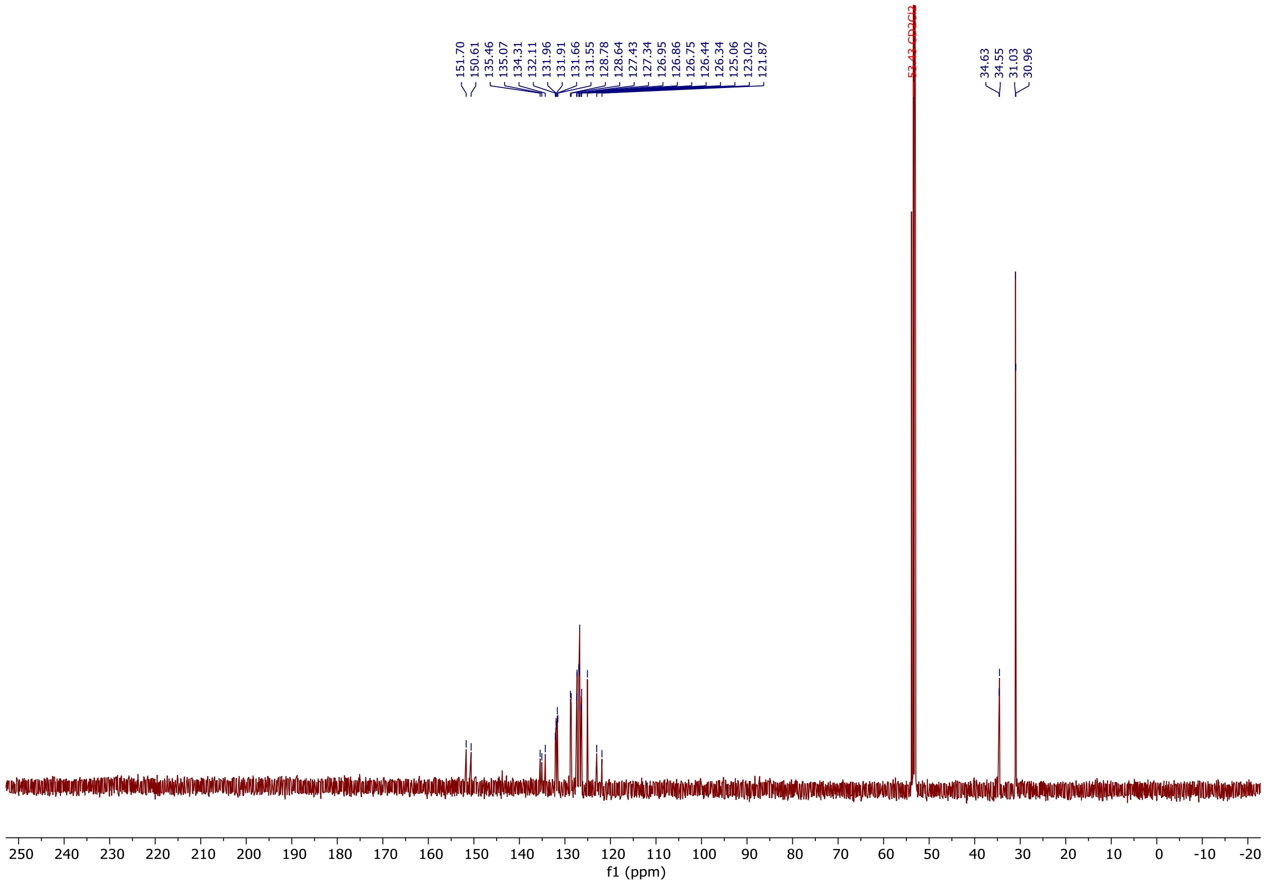
f1 (ppm)

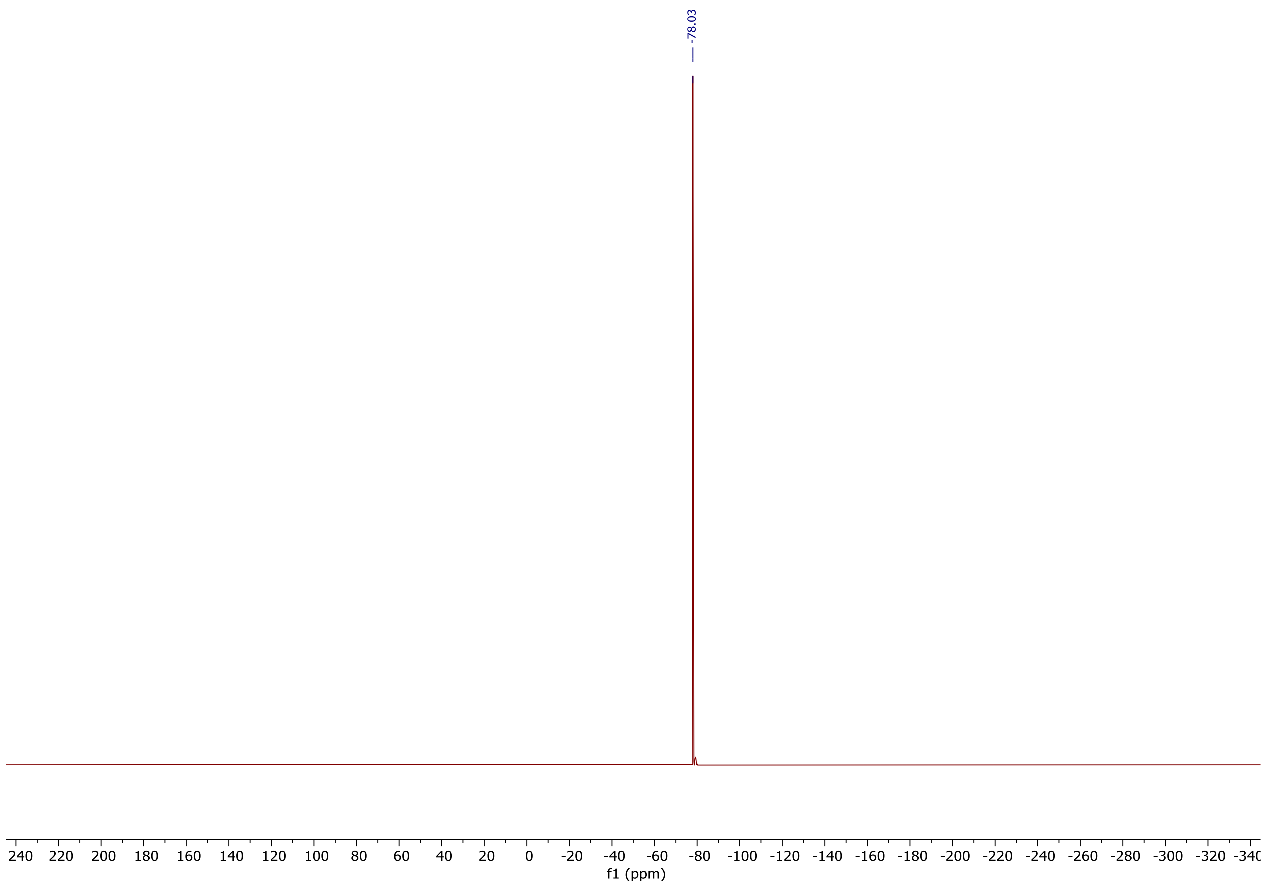
-78.03
240 220 200 180 160 140 120 100 80 60 40 20 0 -20 -40 -60 -80 -100 -120 -140 -160 -180 -200 -220 -240 -260 -280 -300 -320 -340
f1 (ppm)

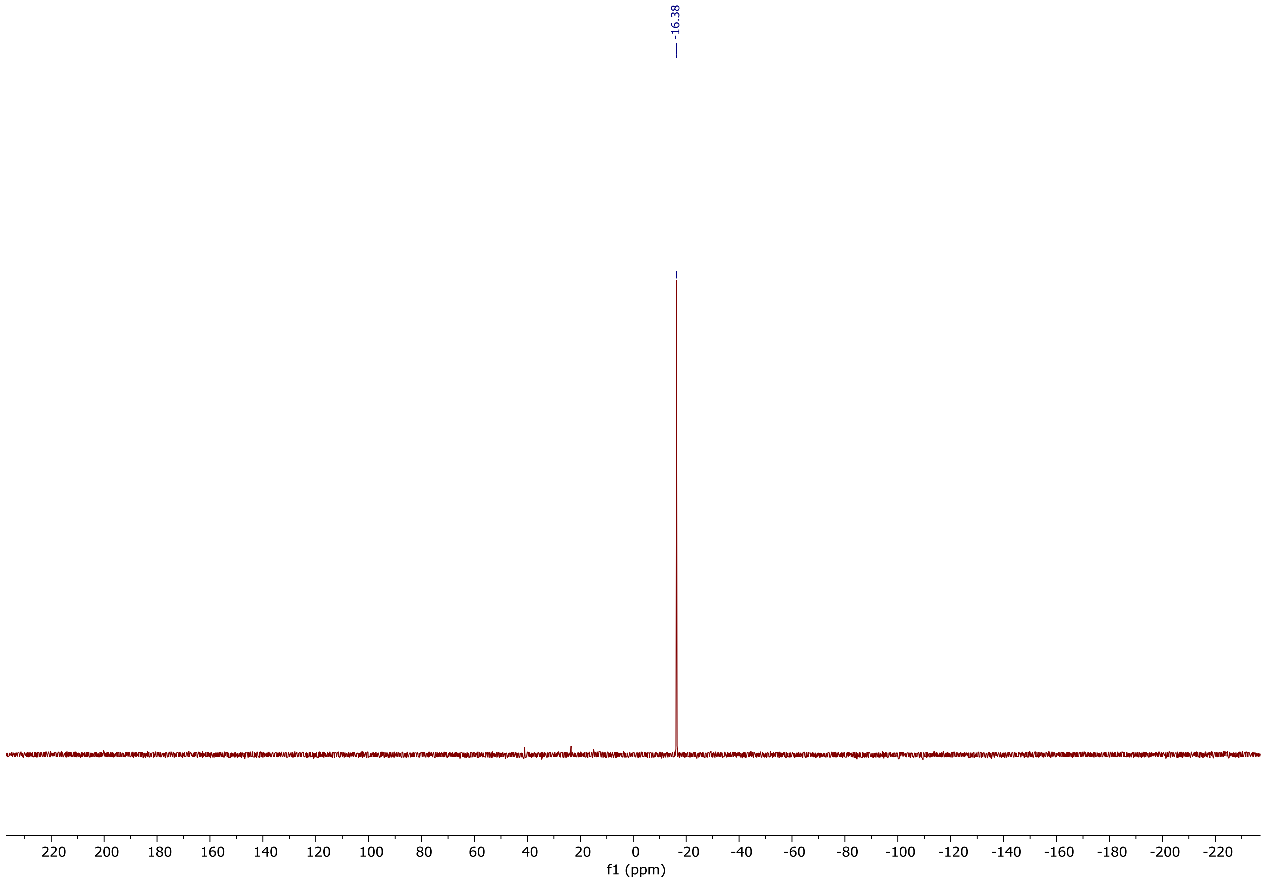
-16.38
220 200 180 160 140 120 100 80 60 40 20 0 -20 -40 -60 -80 -100 -120 -140 -160 -180 -200 -220
f1 (ppm)

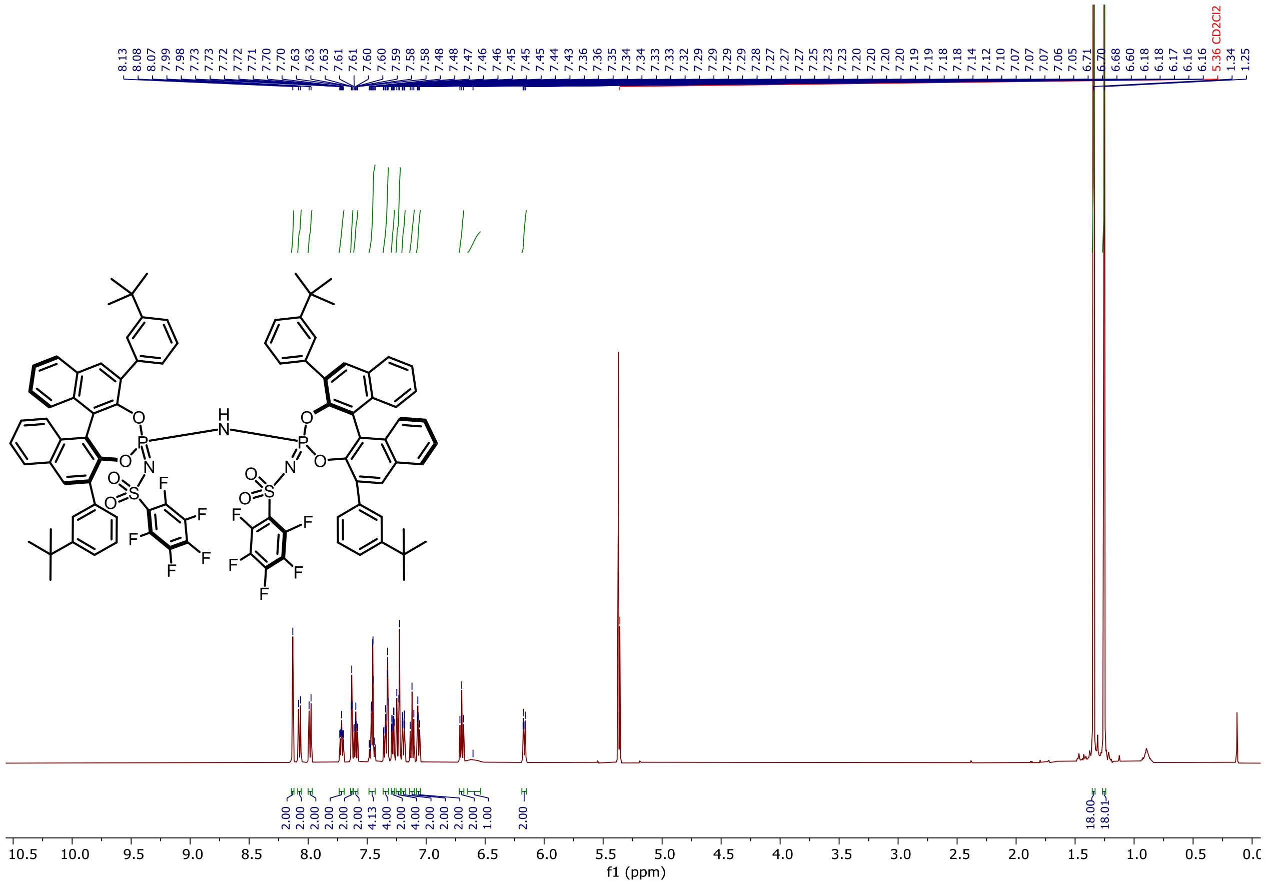
8.13 8.08 8.07 7.99 7.98 7.73 7.73 7.72 7.72 7.71 7.70 7.70 7.63 7.63 7.63 7.61 7.61 7.60 7.60 7.59 7.58 7.58 7.48 7.47 7.46 7.46 7.45 7.45 7.45 7.44 7.43 7.36 7.36 7.35 7.34 7.34 7.33 7.33 7.32 7.29 7.29 7.29 7.28 7.27 7.27 7.27 7.25 7.23 7.23 7.20 7.20 7.20 7.20 7.19 7.19 7.18 7.14 7.12 7.10 7.07 7.07 7.07 7.06 7.05 6.71 6.70 6.68 6.60 6.18 6.18 6.17 6.16 6.16 5.36 CD2Cl2 1.34 1.25
2.00 2.00 2.00 2.00 2.00 4.13 4.00 2.00 4.00 2.00 2.00 2.00 1.00 2.00 18.00 18.01
10.5 10.0 9.5 9.0 8.5 8.0 7.5 7.0 6.5 6.0 5.5 5.0 4.5 4.0 3.5 3.0 2.5 2.0 1.5 1.0 0.5 0.0
f1 (ppm)

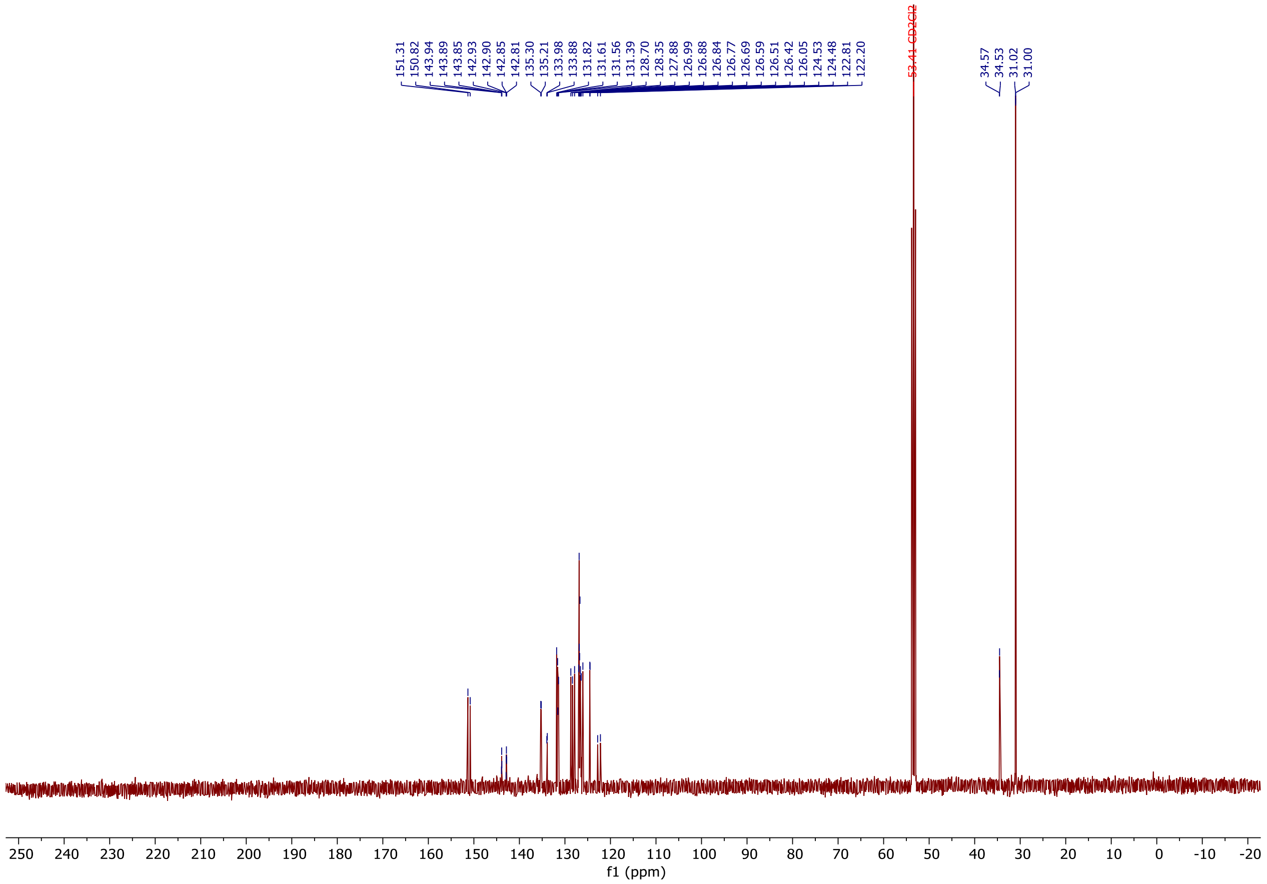
151.31 150.82 143.94 143.89 143.85 142.93 142.90 142.85 142.81 135.30 135.21 133.98 133.88 131.82 131.61 131.56 131.39 128.70 128.35 127.88 126.99 126.88 126.84 126.77 126.69 126.59 126.51 126.42 126.05 124.53 124.48 122.81 122.20
53.41 CD2Cl2
34.57 34.53 31.02 31.00
250 240 230 220 210 200 190 180 170 160 150 140 130 120 110 100 90 80 70 60 50 40 30 20 10 0 -10 -20
f1 (ppm)

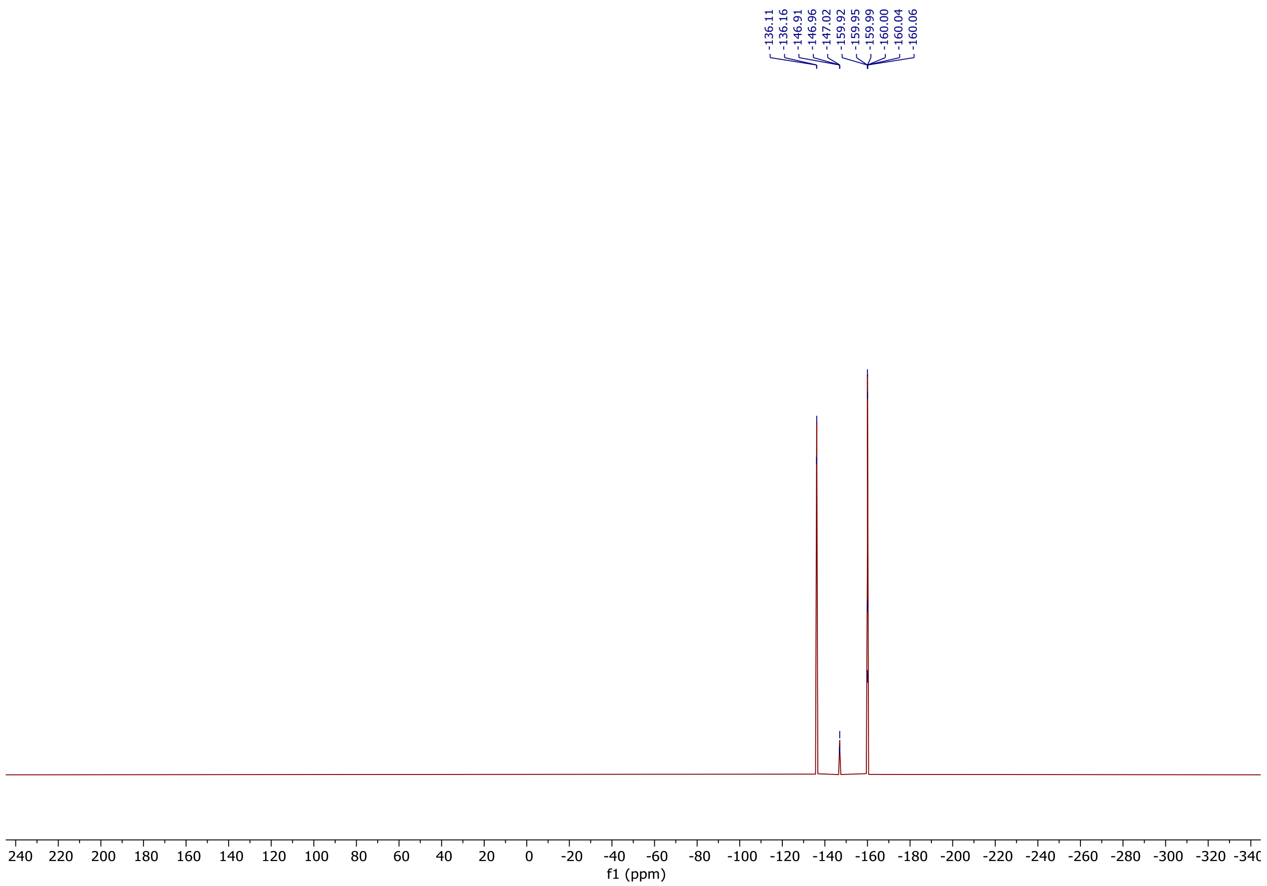
-136.11
-136.16
-146.91
-146.96
-147.02
-159.92
-159.95
-159.99
-160.00
-160.04
-160.06
f1 (ppm)

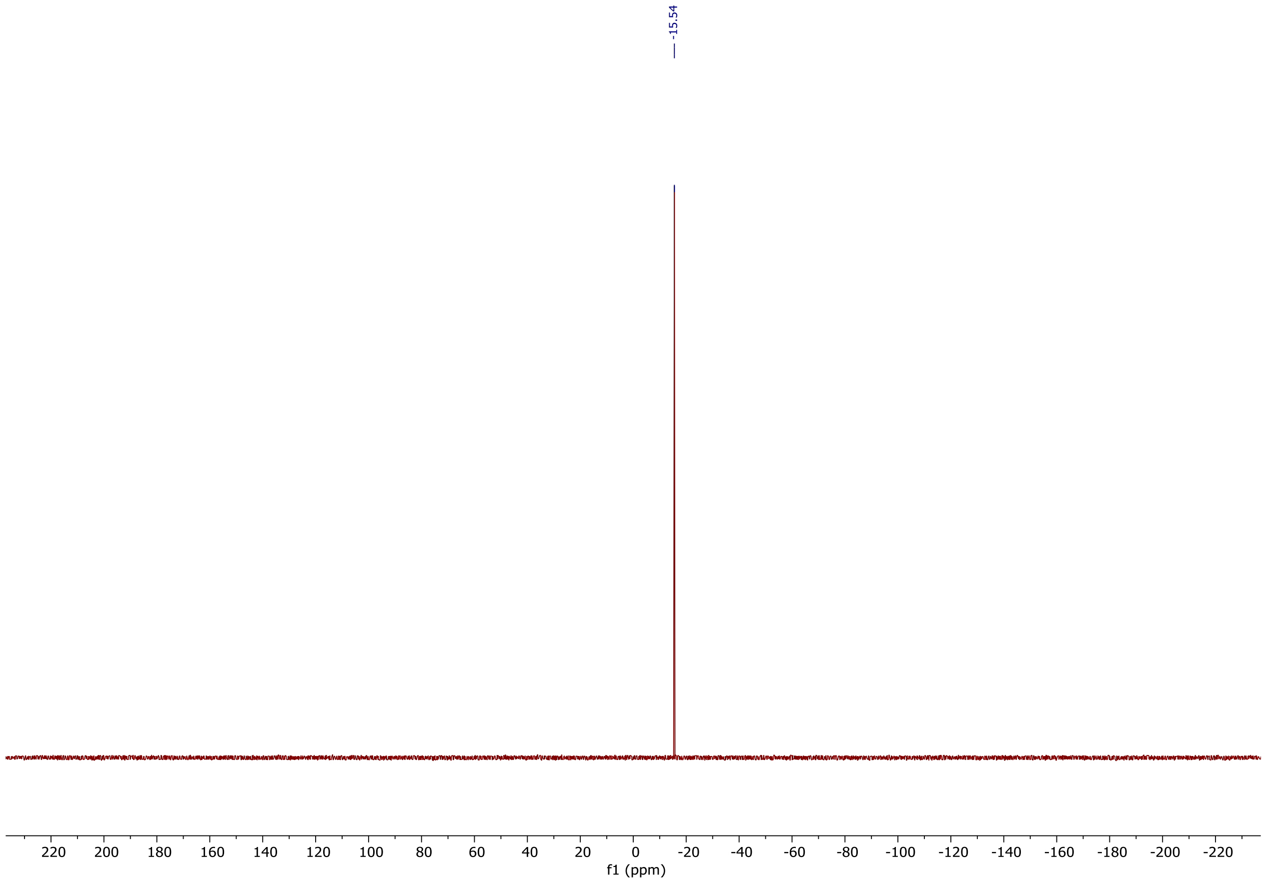
-15.54
f1 (ppm)

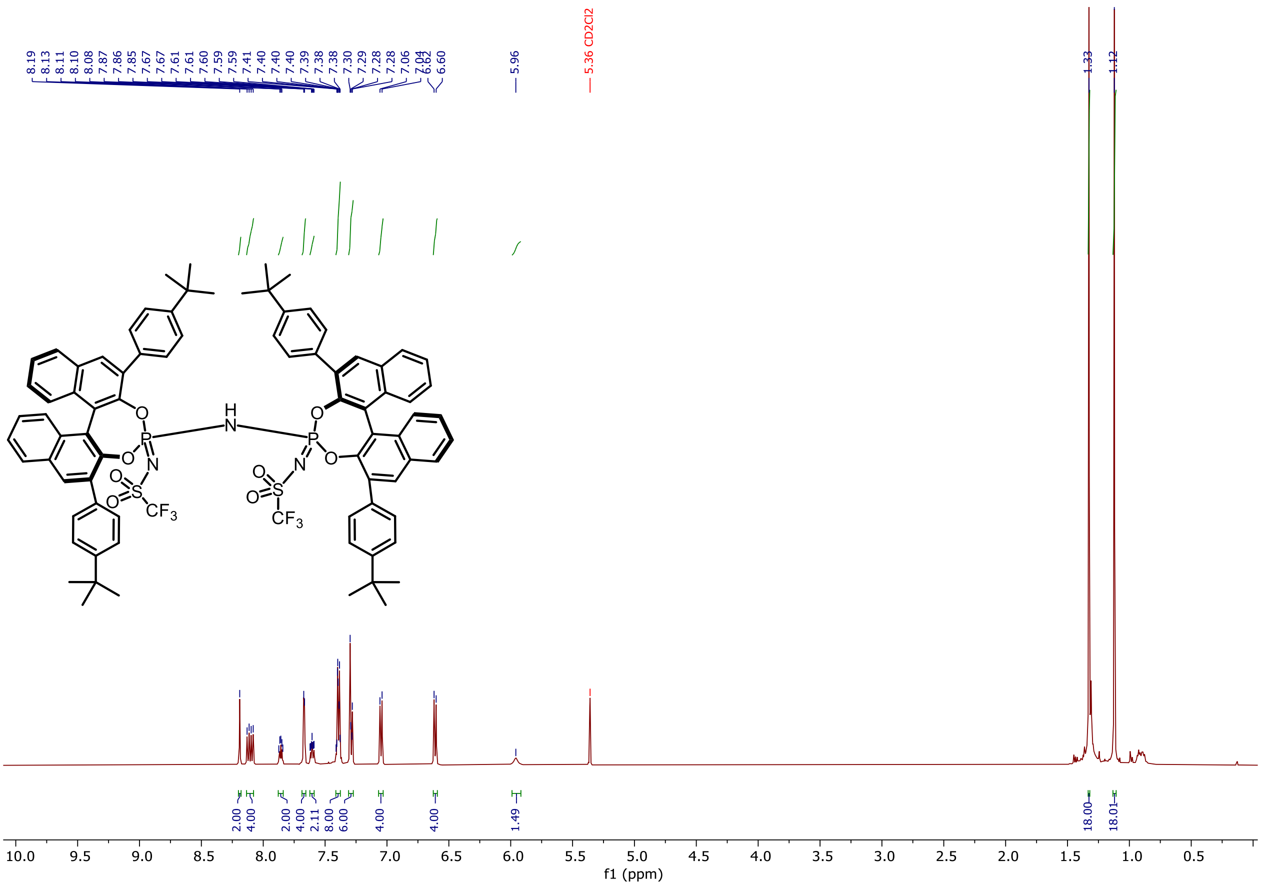
8.19
8.13
8.11
8.10
8.08
7.87
7.86
7.85
7.67
7.67
7.61
7.60
7.59
7.59
7.41
7.40
7.40
7.39
7.38
7.38
7.30
7.29
7.28
7.28
7.06
7.04
6.62
6.60
5.96
5.36 CD2Cl2
1.33
1.12
2.00
4.00
2.00
4.00
2.11
8.00
6.00
4.00
4.00
1.49
18.00
18.01
f1 (ppm)

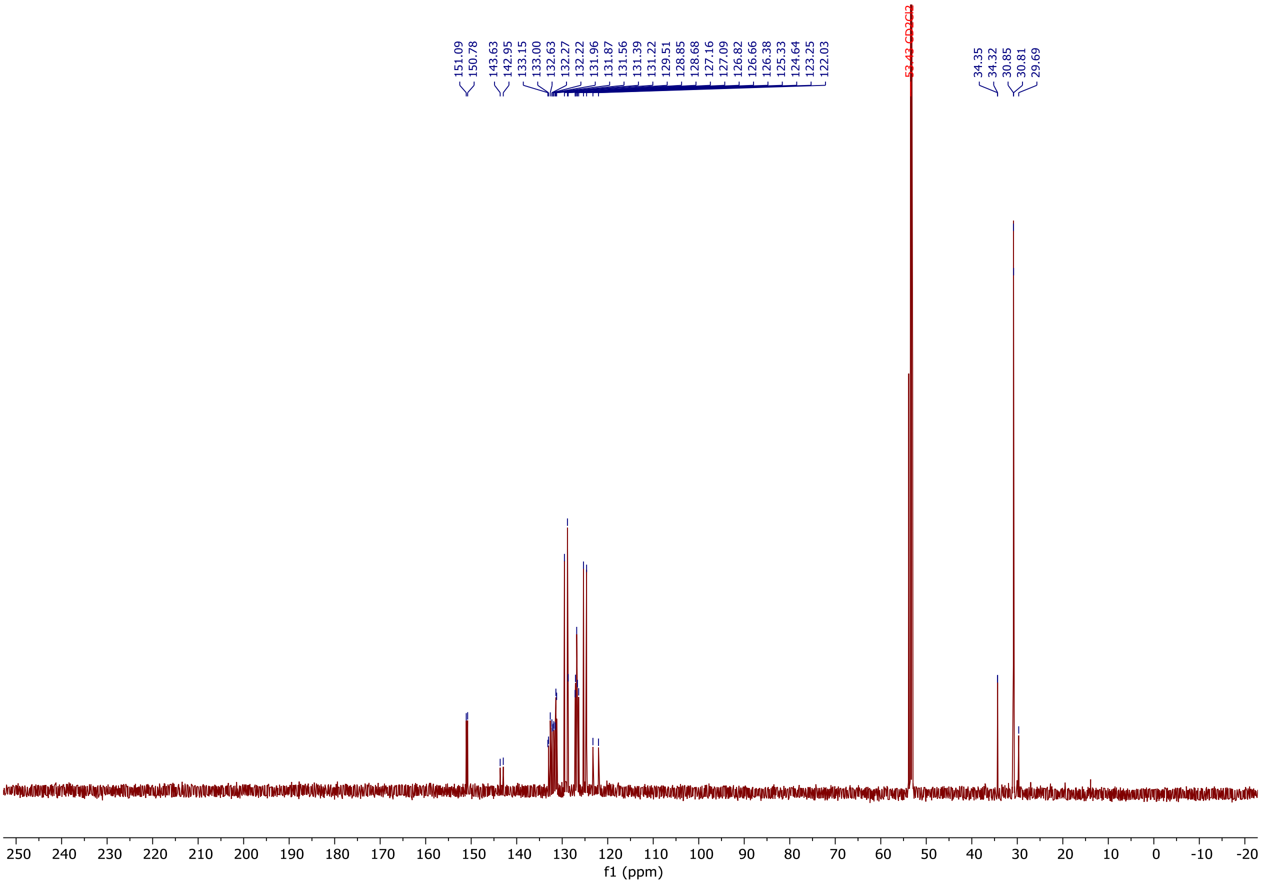
151.09
150.78
143.63
142.95
133.15
133.00
132.63
132.27
132.22
131.96
131.87
131.56
131.39
131.22
129.51
128.85
128.68
127.16
127.09
126.82
126.66
126.38
125.33
124.64
123.25
122.03
53.43 CD2Cl2
34.35
34.32
30.85
30.81
29.69
f1 (ppm)

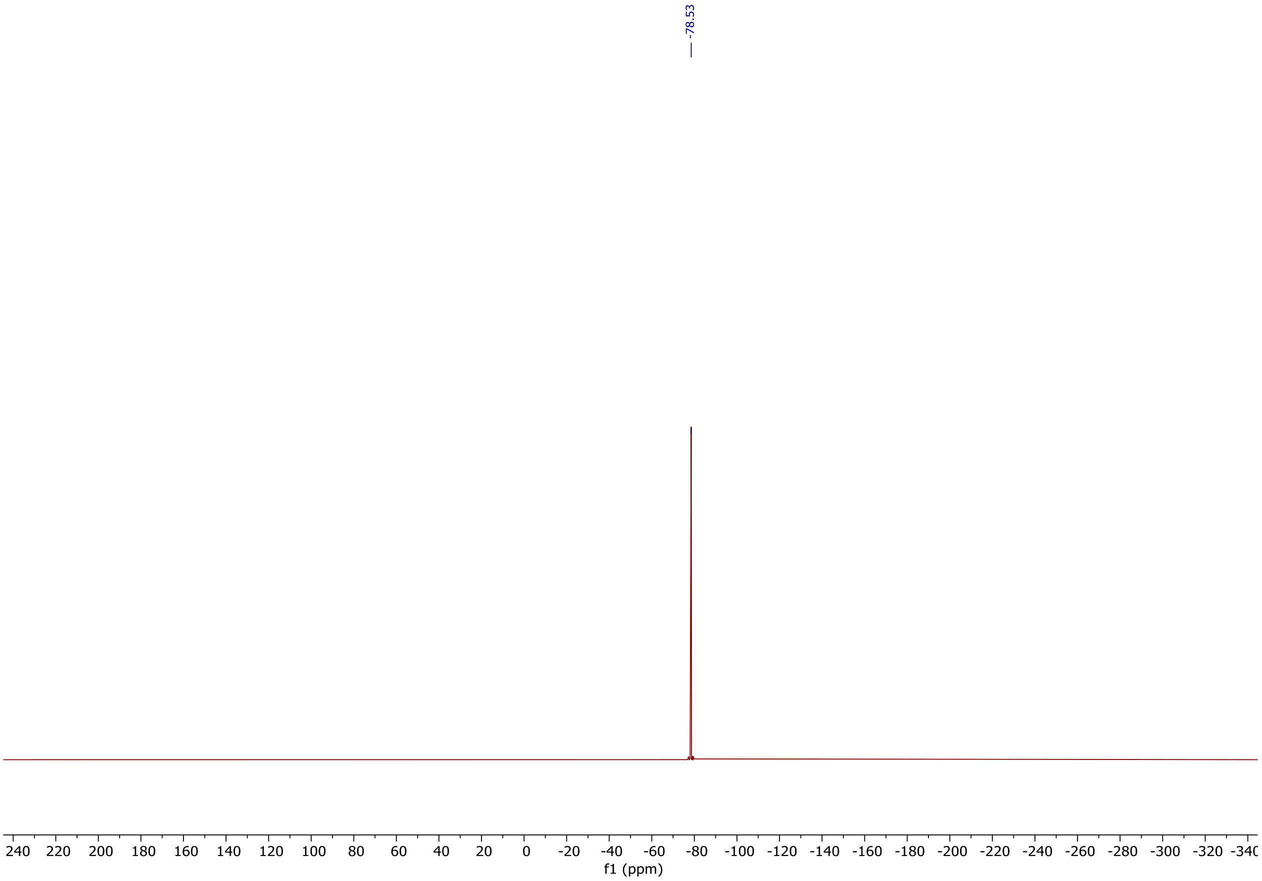
-78.53
240 220 200 180 160 140 120 100 80 60 40 20 0 -20 -40 -60 -80 -100 -120 -140 -160 -180 -200 -220 -240 -260 -280 -300 -320 -340
f1 (ppm)

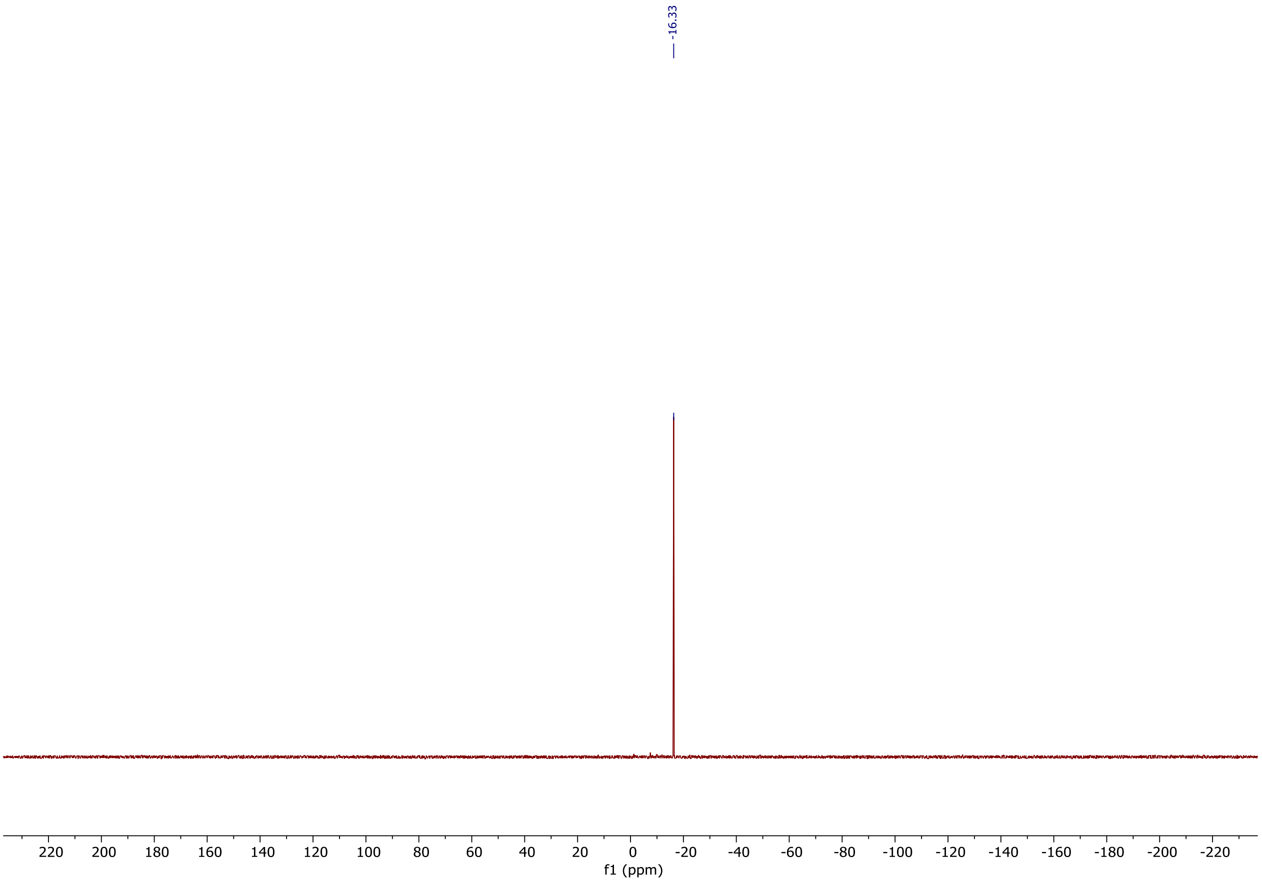
-16.33
220 200 180 160 140 120 100 80 60 40 20 0 -20 -40 -60 -80 -100 -120 -140 -160 -180 -200 -220
f1 (ppm)

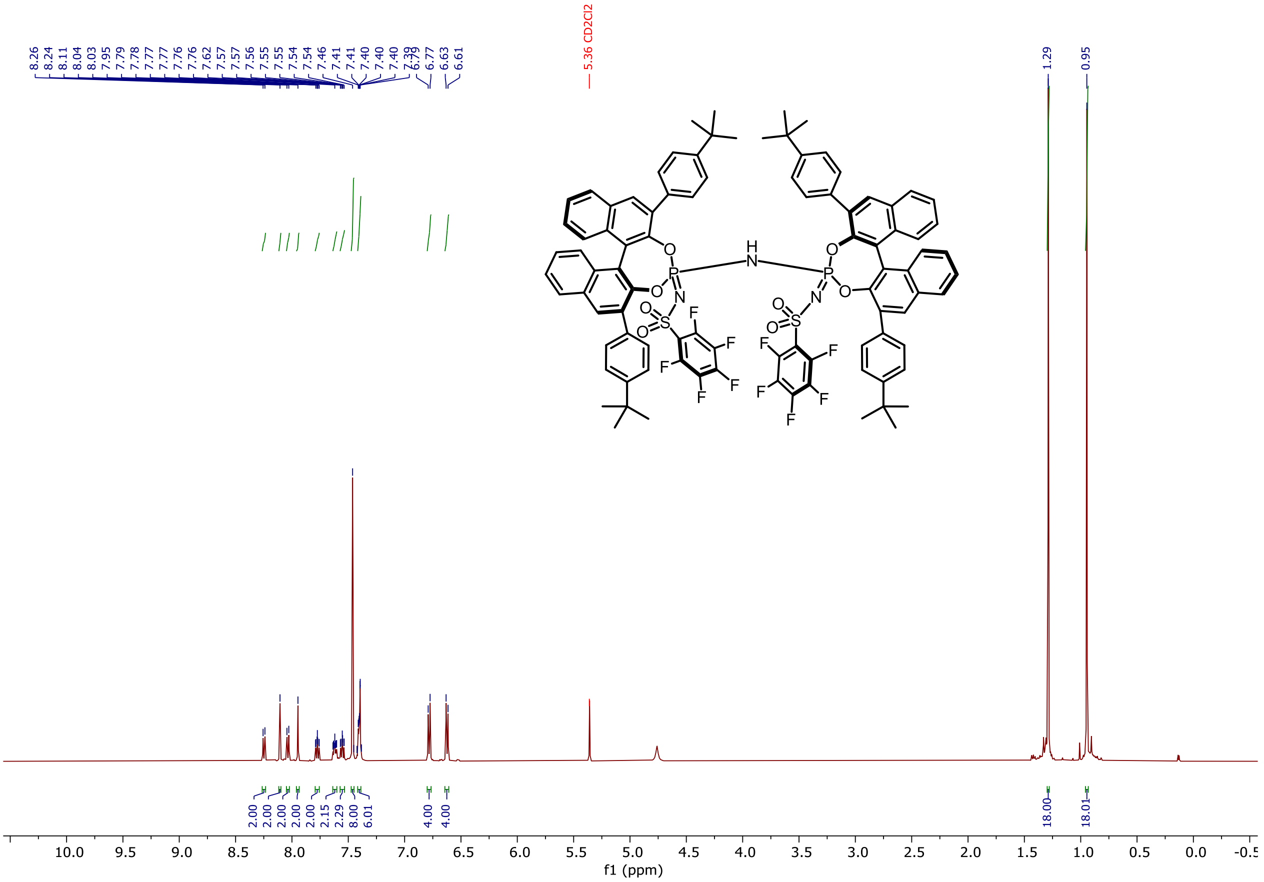
8.26
8.24
8.11
8.04
8.03
7.95
7.79
7.78
7.77
7.77
7.76
7.76
7.62
7.57
7.57
7.56
7.55
7.55
7.54
7.54
7.46
7.41
7.41
7.40
7.40
7.40
7.39
6.79
6.77
6.63
6.61
5.36 CD2Cl2
1.29
0.95
2.00
2.00
2.00
2.00
2.00
2.15
2.29
8.00
6.01
4.00
4.00
18.00
18.01
10.0 9.5 9.0 8.5 8.0 7.5 7.0 6.5 6.0 5.5 5.0 4.5 4.0 3.5 3.0 2.5 2.0 1.5 1.0 0.5 0.0 -0.5
f1 (ppm)

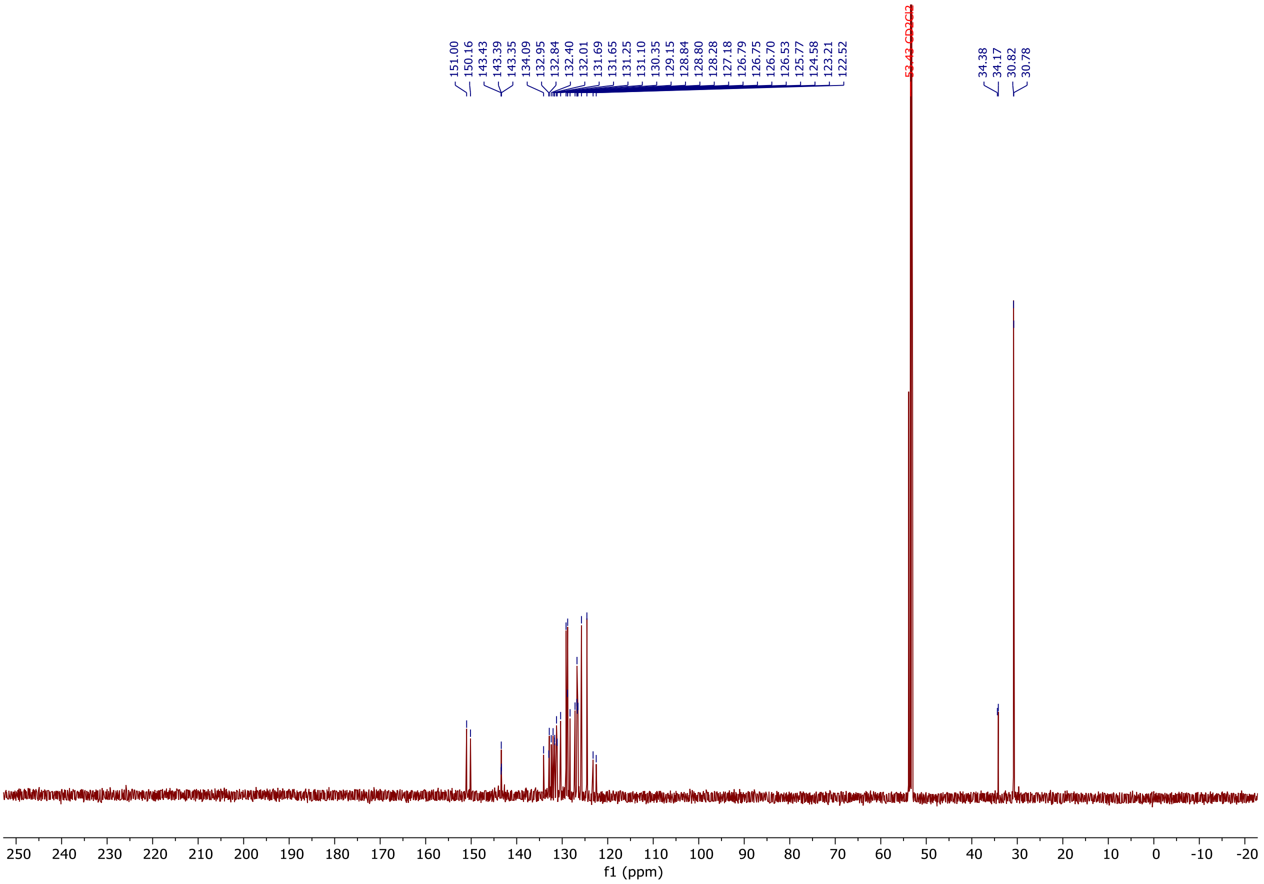
151.00
150.16
143.43
143.39
143.35
134.09
132.95
132.84
132.40
132.01
131.69
131.65
131.25
131.10
130.35
129.15
128.84
128.80
128.28
127.18
126.79
126.75
126.70
126.53
125.77
124.58
123.21
122.52
53.43 CD2Cl2
34.38
34.17
30.82
30.78
250 240 230 220 210 200 190 180 170 160 150 140 130 120 110 100 90 80 70 60 50 40 30 20 10 0 -10 -20
f1 (ppm)

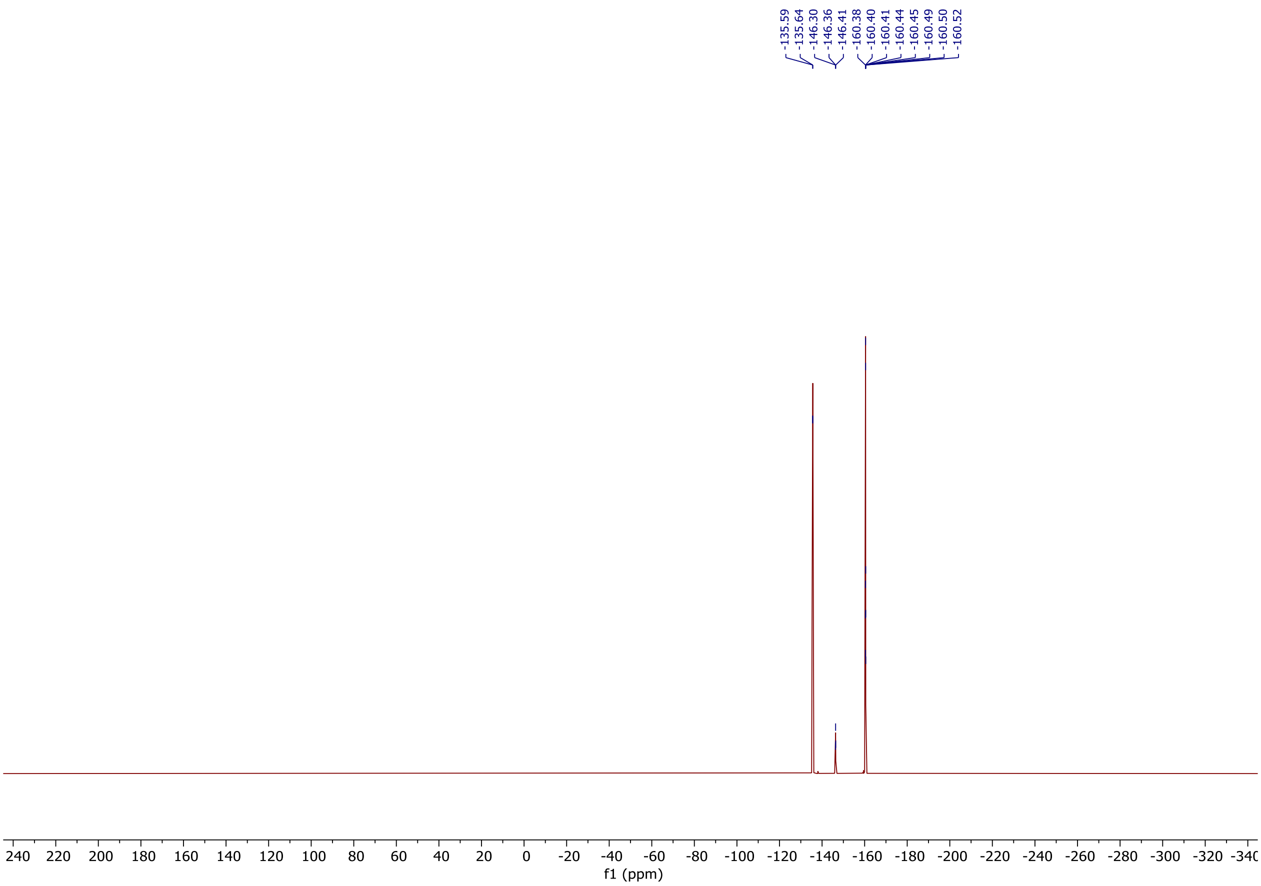
-135.59
-135.64
-146.30
-146.36
-146.41
-160.38
-160.40
-160.41
-160.44
-160.45
-160.49
-160.50
-160.52
240 220 200 180 160 140 120 100 80 60 40 20 0 -20 -40 -60 -80 -100 -120 -140 -160 -180 -200 -220 -240 -260 -280 -300 -320 -340
f1 (ppm)

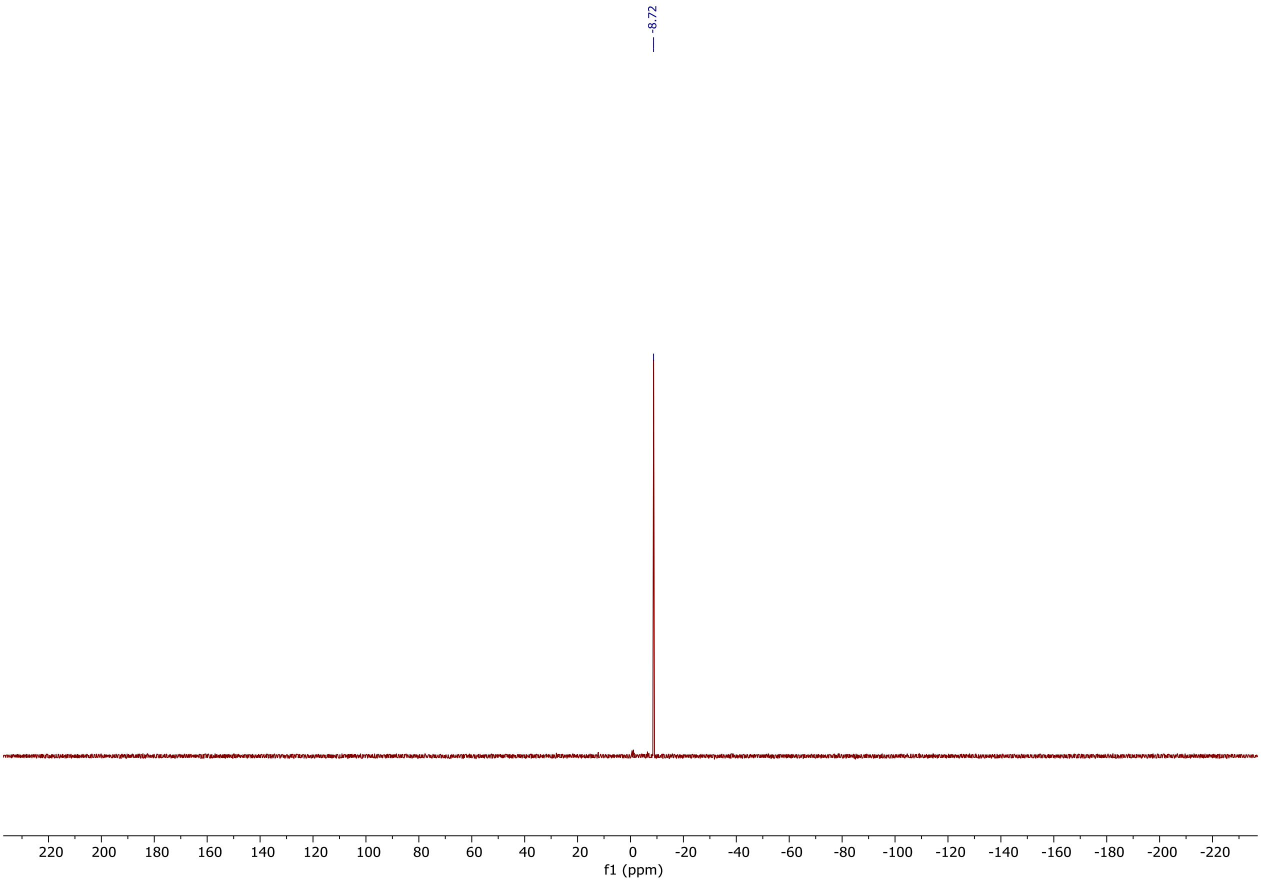
-8.72
220 200 180 160 140 120 100 80 60 40 20 0 -20 -40 -60 -80 -100 -120 -140 -160 -180 -200 -220
f1 (ppm)

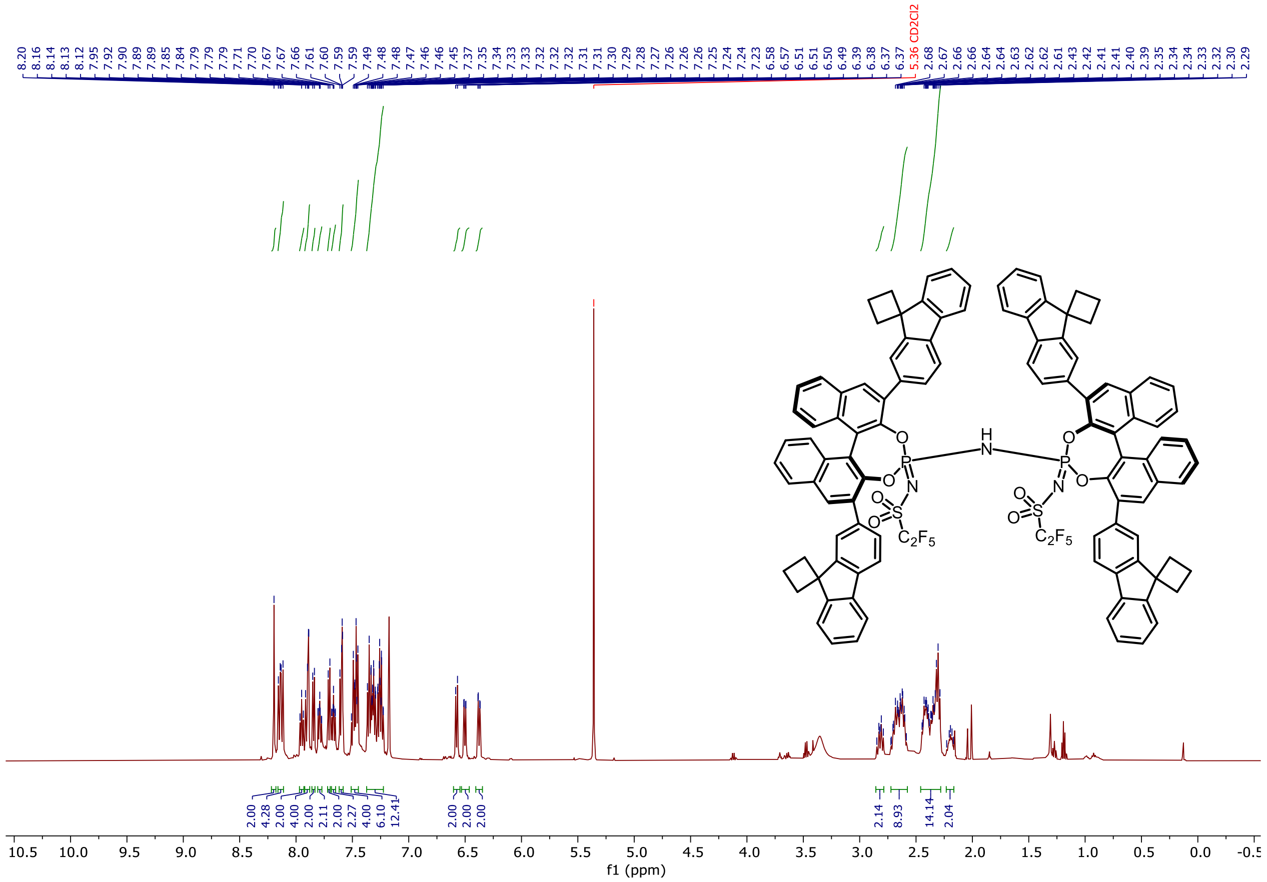

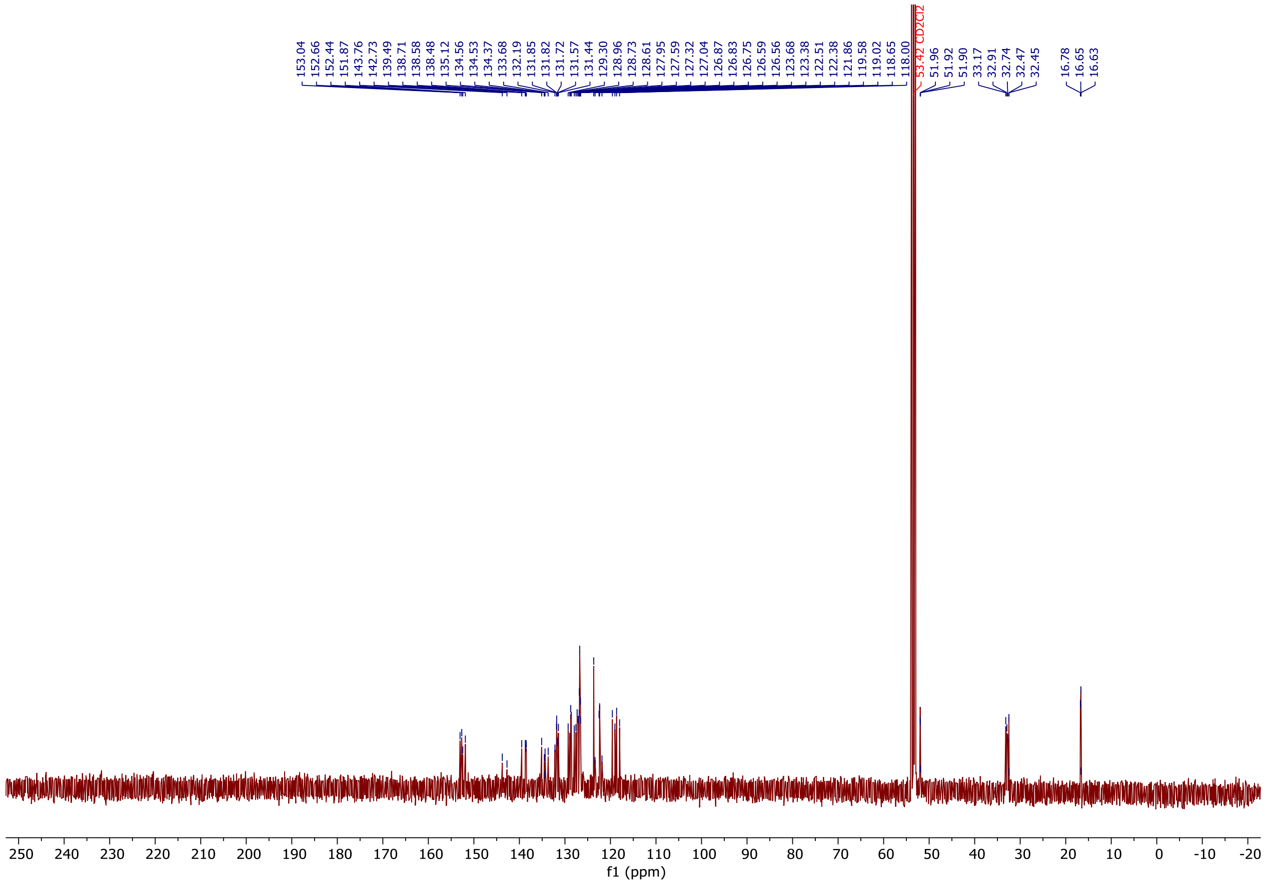

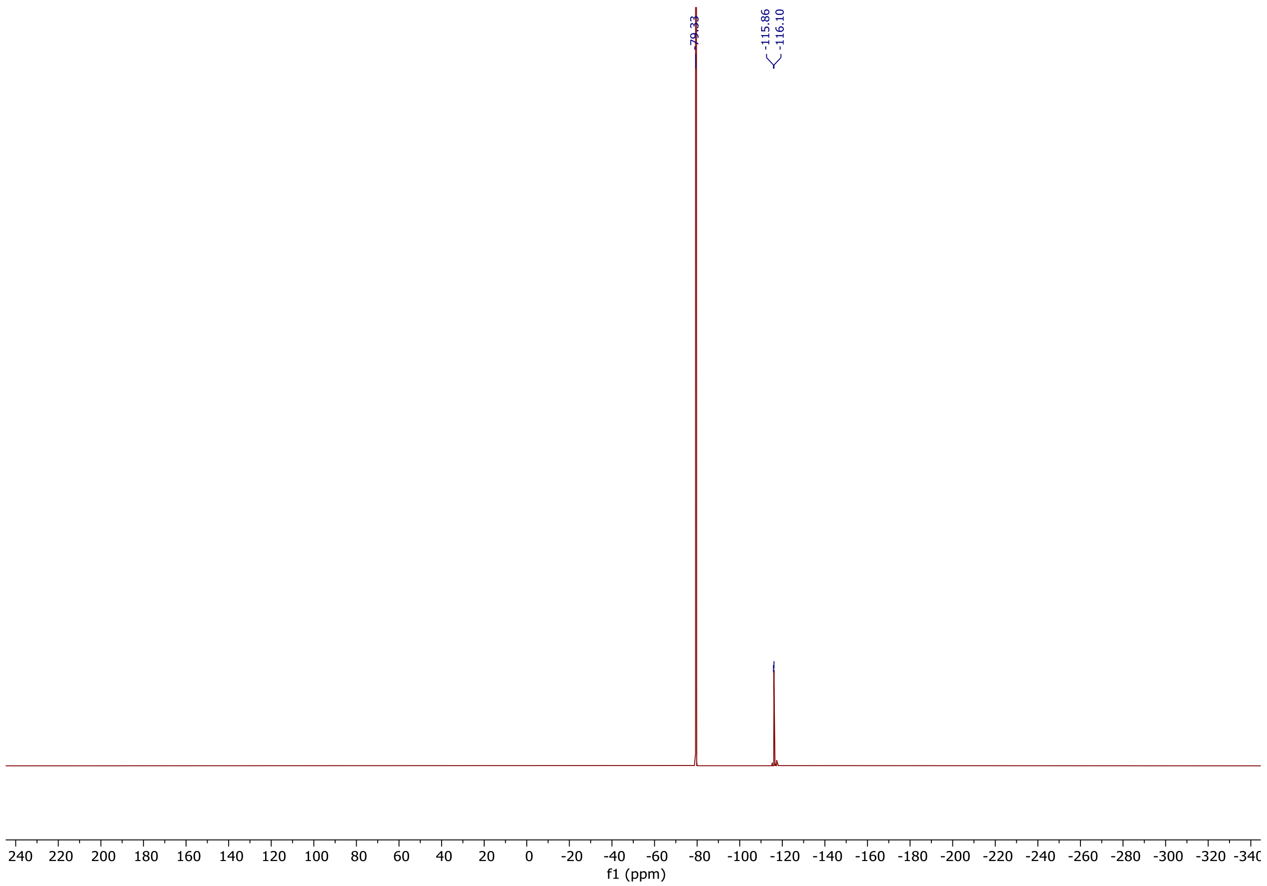
-79.33
-115.86
-116.10
240 220 200 180 160 140 120 100 80 60 40 20 0 -20 -40 -60 -80 -100 -120 -140 -160 -180 -200 -220 -240 -260 -280 -300 -320 -340
f1 (ppm)

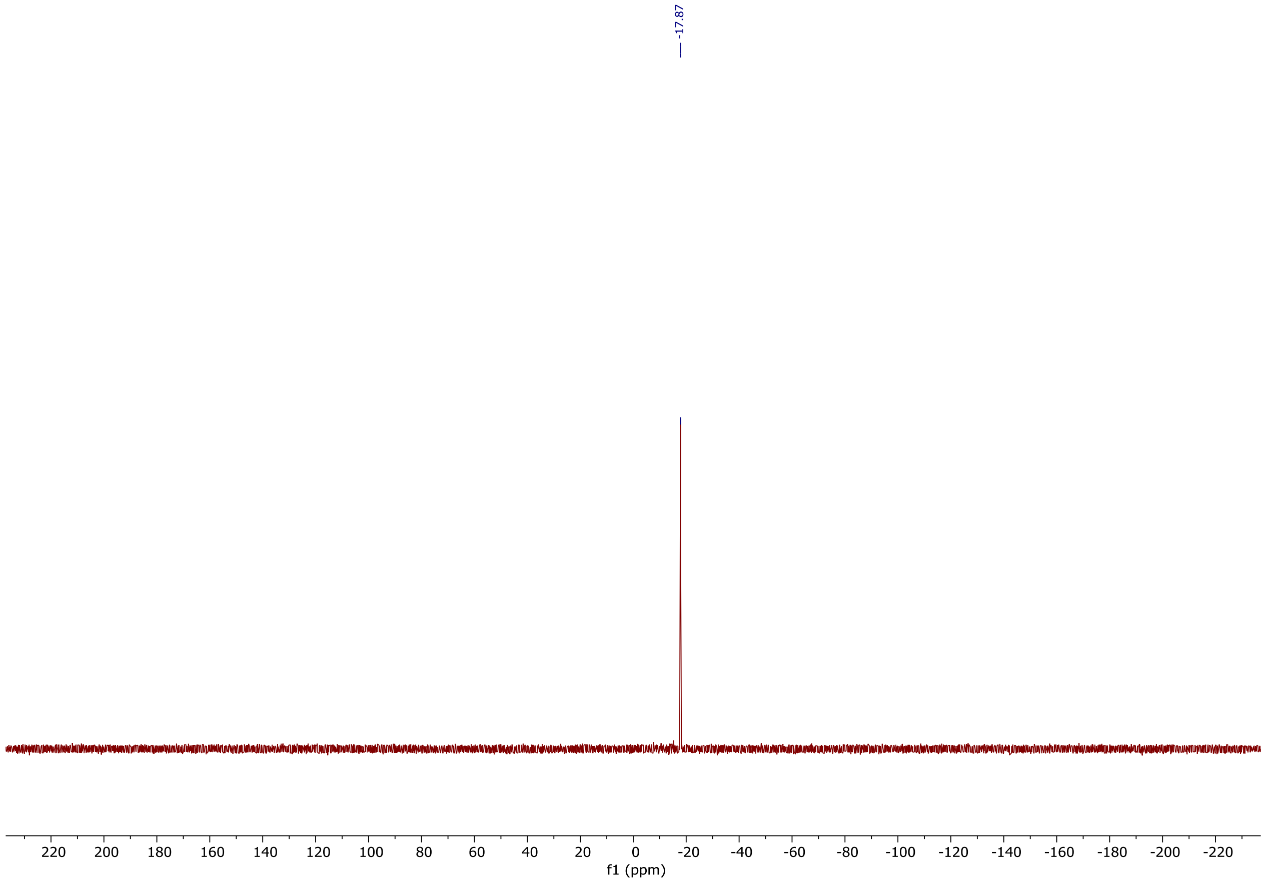
-17.87
220 200 180 160 140 120 100 80 60 40 20 0 -20 -40 -60 -80 -100 -120 -140 -160 -180 -200 -220
f1 (ppm)

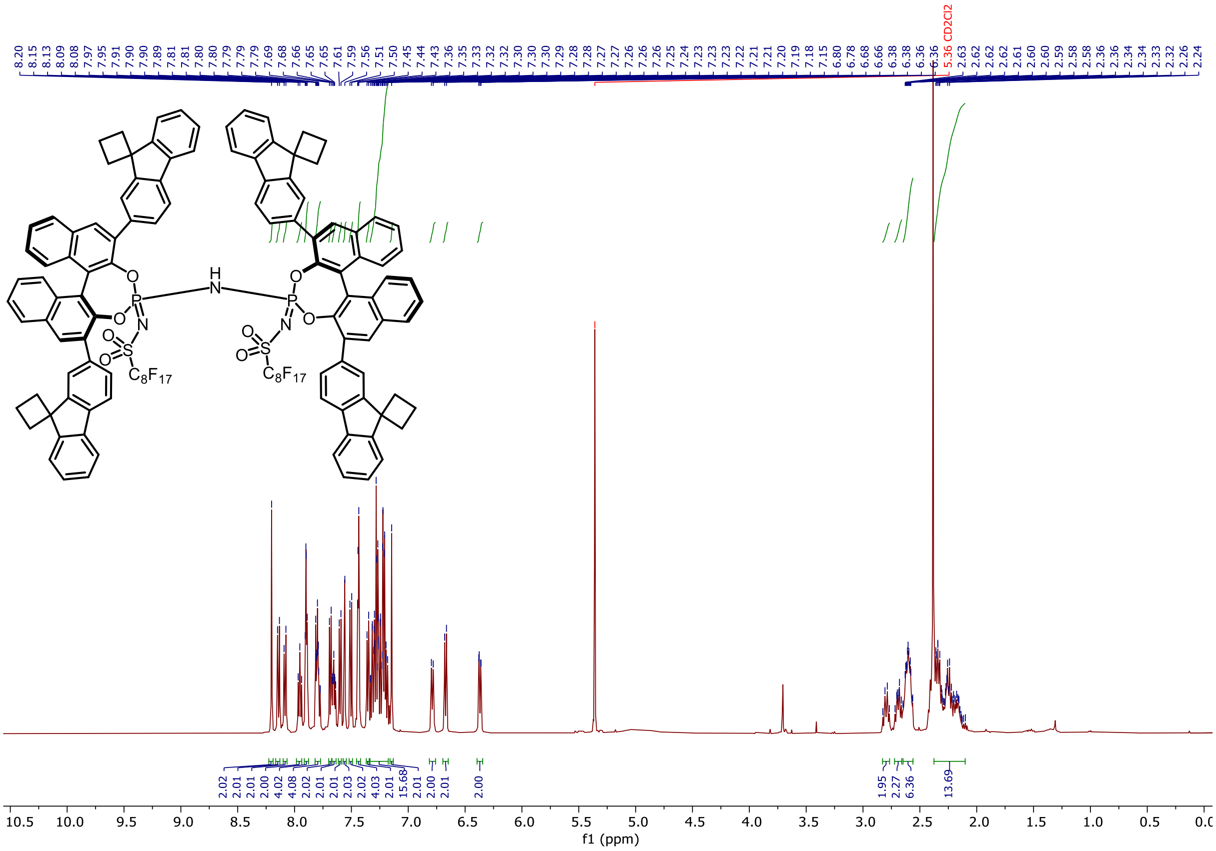
8.20 8.15 8.13 8.09 8.08 7.97 7.95 7.91 7.90 7.90 7.89 7.81 7.81 7.80 7.80 7.79 7.79 7.69 7.68 7.66 7.65 7.65 7.61 7.59 7.56 7.51 7.50 7.45 7.44 7.43 7.36 7.35 7.33 7.32 7.32 7.30 7.30 7.29 7.28 7.28 7.27 7.27 7.26 7.26 7.25 7.24 7.23 7.23 7.22 7.21 7.21 7.20 7.19 7.18 7.15 6.80 6.78 6.68 6.66 6.38 6.38 6.36 6.36
5.36 CD2Cl2
2.63 2.62 2.62 2.61 2.60 2.59 2.58 2.58 2.36 2.34 2.34 2.33 2.32 2.26 2.24
H
N
O
P
N
O
S
C8F17
2.02 2.01 2.01 2.00 4.02 4.08 2.02 2.01 2.01 2.03 2.02 4.03 2.01 15.68 2.01 2.00 2.01 2.00
1.95 2.27 6.36 13.69
10.5 10.0 9.5 9.0 8.5 8.0 7.5 7.0 6.5 6.0 5.5 5.0 4.5 4.0 3.5 3.0 2.5 2.0 1.5 1.0 0.5 0.0
f1 (ppm)

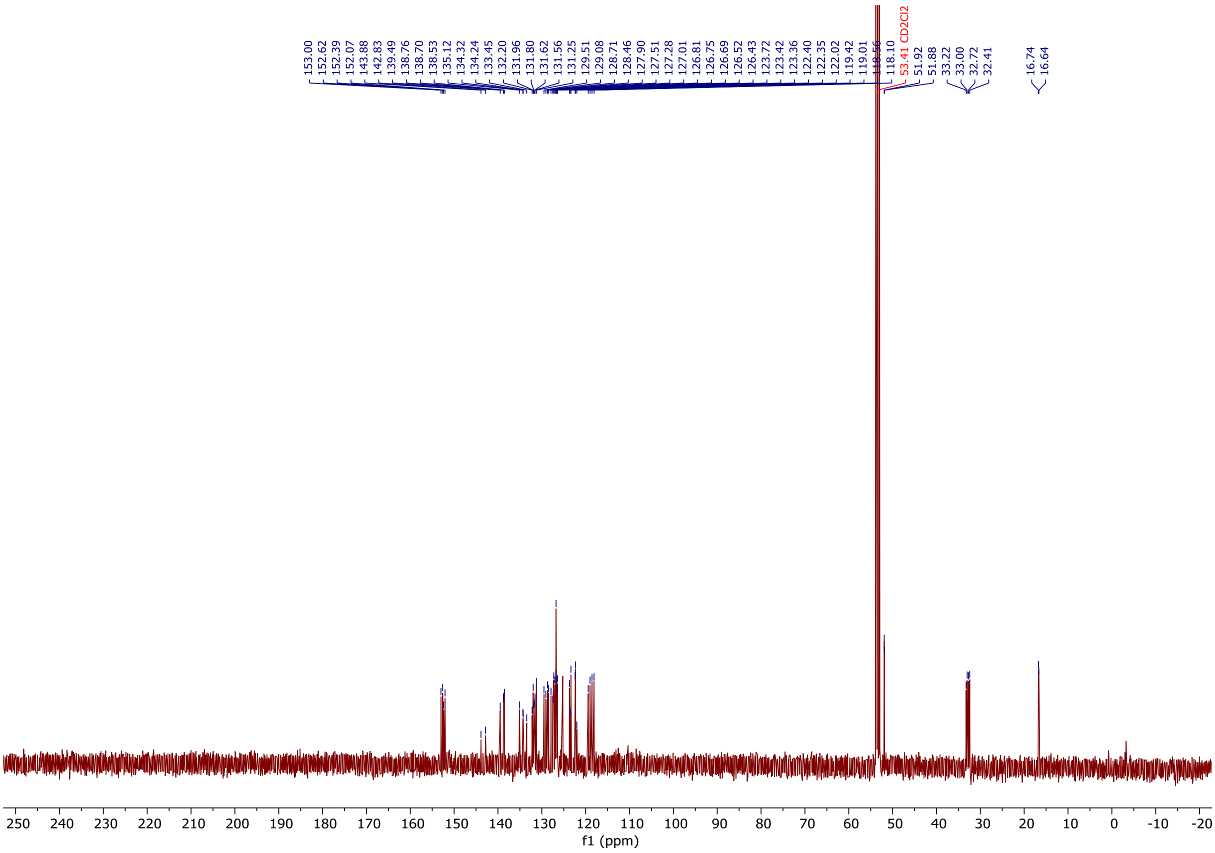
153.00 152.62 152.39 152.07 143.88 142.83 139.49 138.76 138.70 138.53 135.12 134.32 134.24 133.45 132.20 131.96 131.80 131.62 131.56 131.25 129.51 129.08 128.71 128.46 127.90 127.51 127.28 127.01 126.81 126.75 126.69 126.52 126.43 123.72 123.42 123.36 122.40 122.35 122.02 119.42 119.01 118.56 118.10
53.41 CD2Cl2
51.92 51.88 33.22 33.00 32.72 32.41 16.74 16.64
250 240 230 220 210 200 190 180 170 160 150 140 130 120 110 100 90 80 70 60 50 40 30 20 10 0 -10 -20
f1 (ppm)

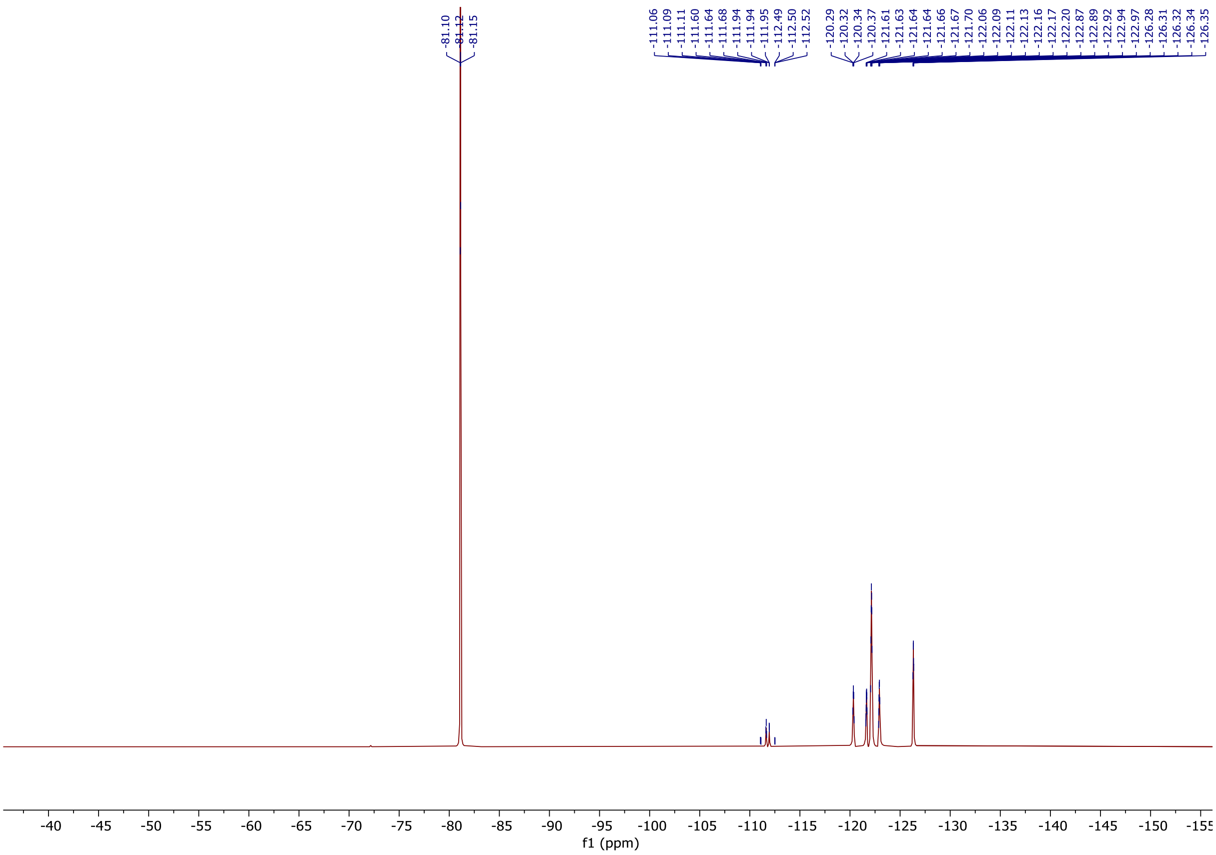
-81.10
-81.12
-81.15
-111.06
-111.09
-111.11
-111.60
-111.64
-111.68
-111.94
-111.94
-111.95
-112.49
-112.50
-112.52
-120.29
-120.32
-120.34
-120.37
-121.61
-121.63
-121.64
-121.64
-121.66
-121.67
-121.70
-122.06
-122.09
-122.11
-122.13
-122.16
-122.17
-122.20
-122.87
-122.89
-122.92
-122.94
-122.97
-126.28
-126.31
-126.32
-126.34
-126.35
f1 (ppm)

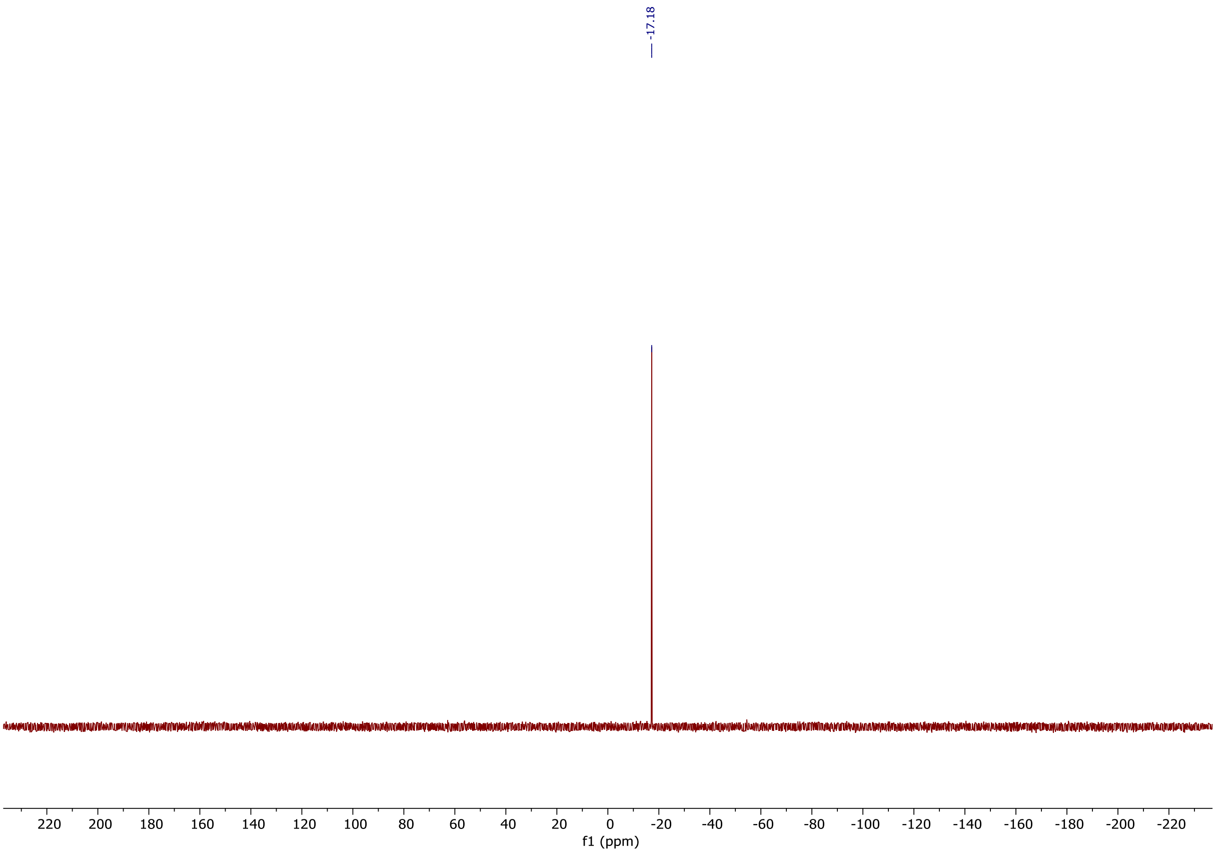
-17.18
f1 (ppm)

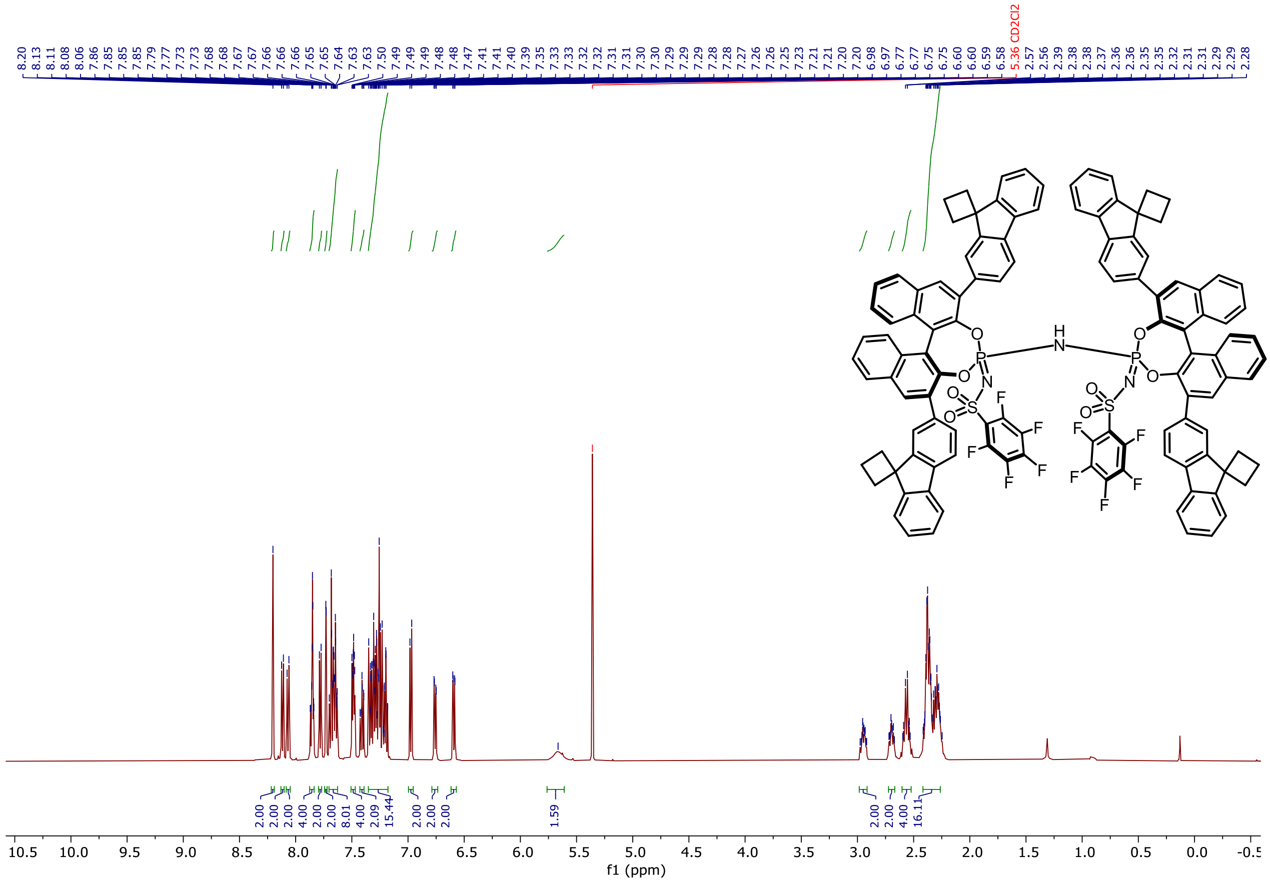

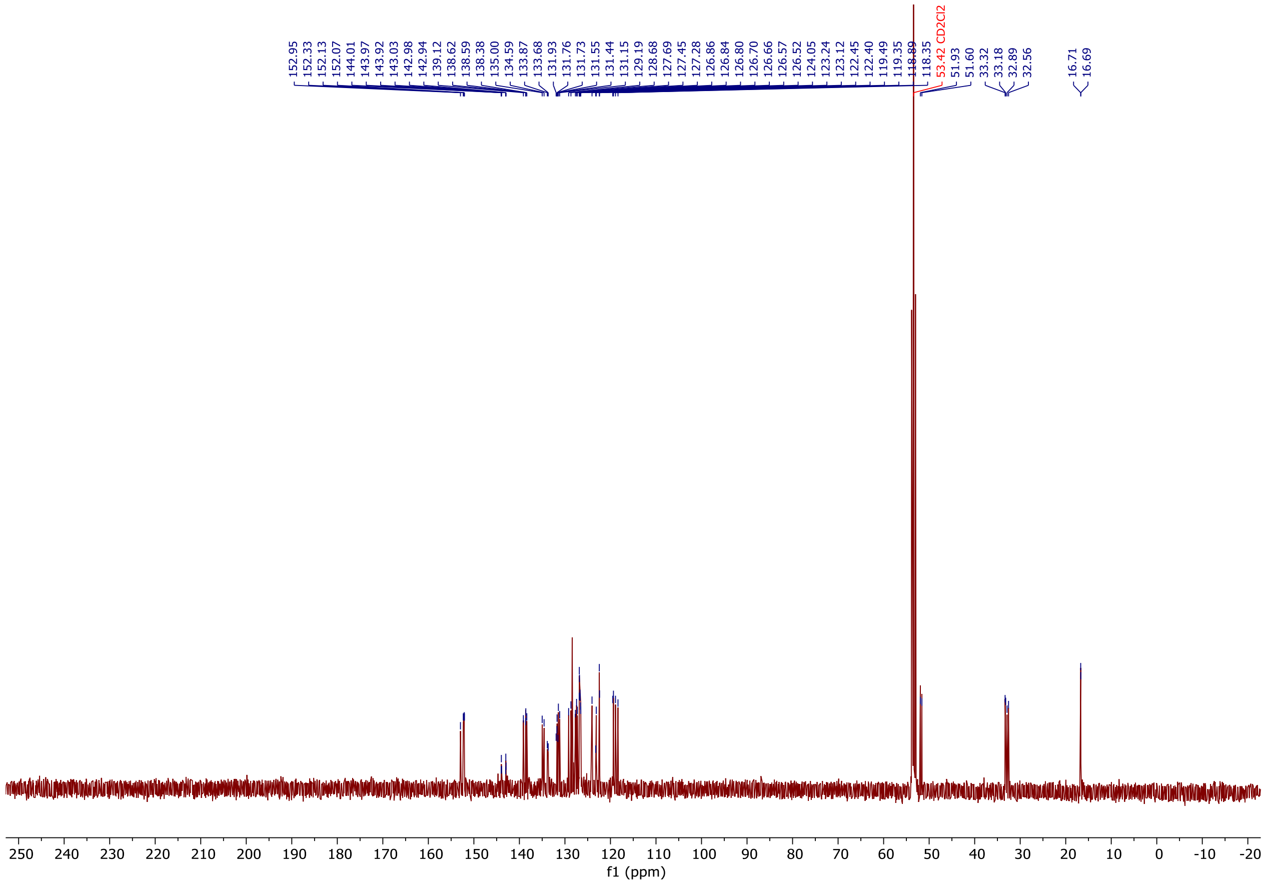

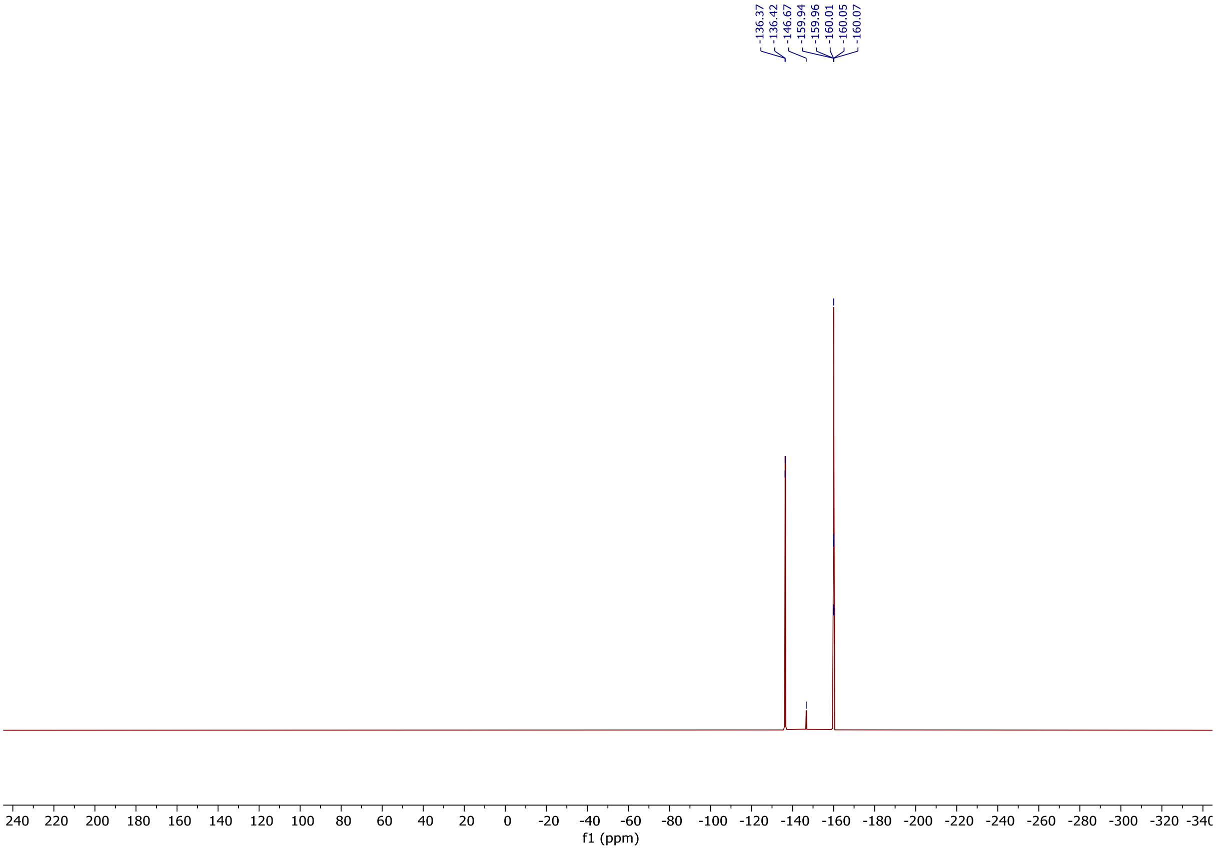
-136.37
-136.42
-146.67
-159.94
-159.96
-160.01
-160.05
-160.07
240 220 200 180 160 140 120 100 80 60 40 20 0 -20 -40 -60 -80 -100 -120 -140 -160 -180 -200 -220 -240 -260 -280 -300 -320 -340
f1 (ppm)

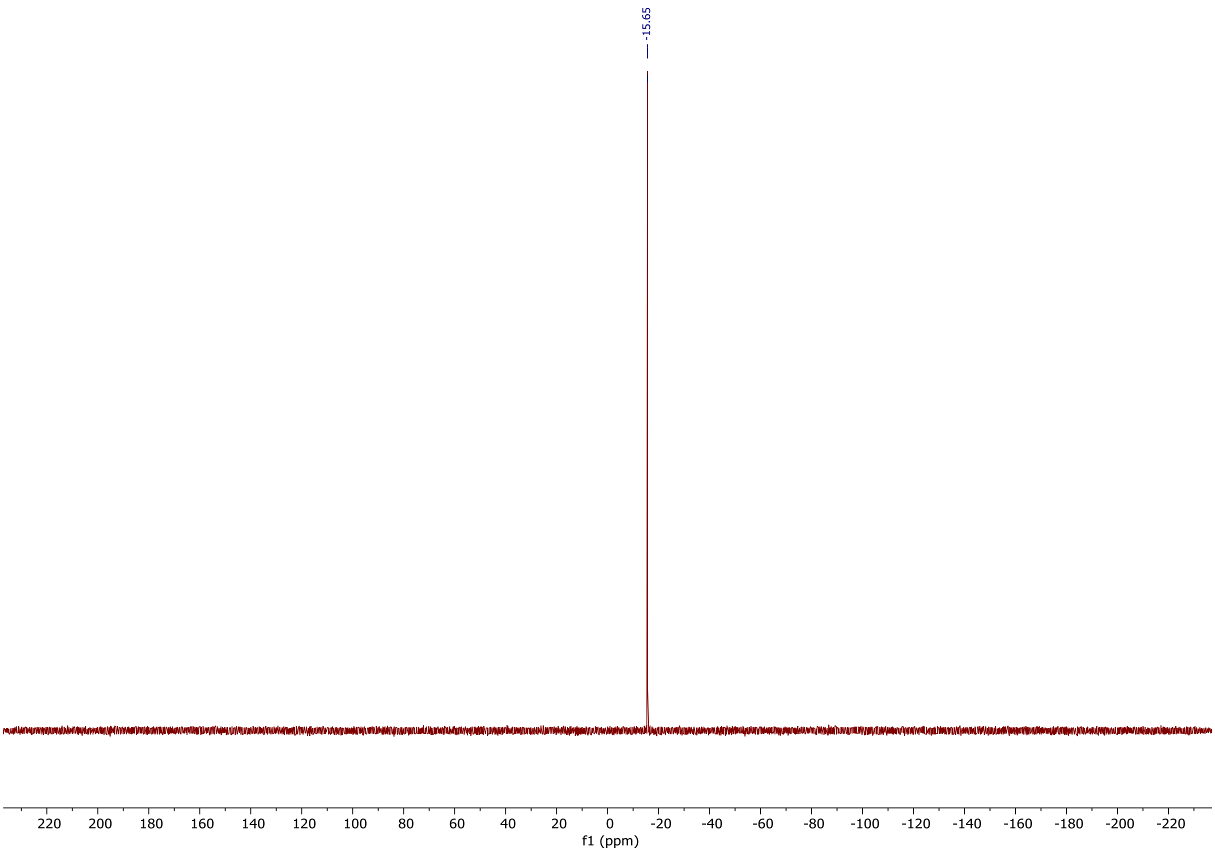
-15.65
220 200 180 160 140 120 100 80 60 40 20 0 -20 -40 -60 -80 -100 -120 -140 -160 -180 -200 -220
f1 (ppm)

12. Reference

1. Ouyang, J.; Maji, R.; Leutzsch, M.; Mitschke, B.; List, B. Design of an Organocatalytic Asymmetric (4+3) Cycloaddition of 2-Indolylalcohols with Dienolsilanes. *J. Am. Chem. Soc.* **2022**, *144*, 8460–8466.
2. Gatzenmeier, T.; Turberg, M.; Yepes, D.; Xie, Y.; Neese, F.; Bistoni, G.; List, B. Scalable and Highly Diastereo- and Enantioselective Catalytic Diels−Alder Reaction of *α,β*-Unsaturated Methyl Esters. *J. Am. Chem. Soc.* **2018**, *140*, 12671–12676.
3. Wenzel, A. G.; Jacobsen, E. N. Asymmetric Catalytic Mannich Reactions Catalyzed by Urea Derivatives: Enantioselective Synthesis of *β*-Aryl-*β*-Amino Acids. *J. Am. Chem. Soc.* **2002**, *124*, 12964–12965.
4. Lin, H.; Xiao, L.-J.; Zhou, M.-J.; Yu, H.-M.; Xie, J.-H.; Zhou, Q.-L. Enantioselective Approach to (−)-Hamigeran B and (−)-4-Bromohamigeran B via Catalytic Asymmetric Hydrogenation of Racemic Ketone To Assemble the Chiral Core Framework. *Org. Lett.* **2016**, *18*, 1434–1437.
5. Bae, H. Y.; List, B. Triflimide: An Overlooked High-Performance Catalyst of the Mukaiyama Aldol Reaction of Silyl Ketene Acetals with Ketones. *Chem. Eur. J.* **2018**, *24*, 13767–13772.
6. Montforts, F.-P.; Gesing-Zibulak, I.; Grammenos, W.; Schneider, M.; Laumen, K. Methyl-jasmonat: Ein Kurzer Weg zum Naturstoff und Seinem Unnaturlichen Enantiomer via Palladium(0)-induzierte, Enantiodivergente Alkylierung von Cyclopent-2-en-1,3-diol-Derivaten. *Helv. Chim. Acta.* **1989**, *72*, 1852–1859.
7. (a) Neese, F. *WIREs: Comput. Mol. Sci.* **2022**, *12*, e1606; (b) Neese, F. *WIREs: Comput. Mol. Sci.* **2018**, *8*, e1327; c) Neese, F. The ORCA program system. *WIREs: Comput. Mol. Sci.* **2012**, *2*, 73–78.
8. Pracht, P.; Bohle, F.; Grimme, S. Automated Exploration of the Low-Energy Chemical Space with Fast Quantum Chemical Methods. *Phys. Chem. Chem. Phys.* **2020**, *22*, 7169–7192.
9. Bannwarth, C.; Ehlert, S.; Grimme, S. GFN2-xTB—An Accurate and Broadly Parametrized Self-Consistent Tight-Binding Quantum Chemical Method with Multipole Electrostatics and Density-Dependent Dispersion Contributions. *J. Chem. Theory Comput.* **2019**, *15*, 1652–1671.
10. Perdew, J. P.; Burke, K.; Ernzerhof, M. Generalized Gradient Approximation Made Simple *Phys. Rev. Lett.* **1996**, *77*, 3865–3868.
11. Grimme, S.; Antony, J.; Ehrlich, S.; Krieg, H. A Consistent and Accurate *Ab Initio* Parametrization of Density Functional Dispersion Correction (Dft-D) for the 94 Elements H-Pu. *J. Chem. Phys.* **2010**, *132*, 154104.
12. Grimme, S.; Ehrlich, S.; Goerigk, L. Effect of the Damping Function in Dispersion Corrected Density Functional Theory. *J. Comput. Chem.* **2011**, *32*, 1456–1465.
13. Weigend, F.; Ahlrichs, R. Balanced Basis Sets of Split Valence, Triple Zeta Valence and Quadruple Zeta Valence Quality for H to Rn: Design and Assessment of Accuracy. *Phys. Chem. Chem. Phys.* **2005**, *7*, 3297–3305.
14. Barone, V.; Cossi, M. Quantum Calculation of Molecular Energies and Energy Gradients in Solution by a Conductor Solvent Model. *J. Phys. Chem. A* **1998**, *102*, 1995–2001.

15. Chai, J.-D.; Head-Gordon, M. Long-Range Corrected Hybrid Density Functionals with Damped Atom–Atom Dispersion Corrections. *Phys. Chem. Chem. Phys.* **2008**, *10*, 6615−6620.
16. Spicher, S.; Grimme, S. Robust Atomistic Modeling of Materials, Organometallic, and Biochemical Systems. A*ngew. Chem. Int. Ed.* **2020**, *59*, 15665−15673.
17. Grimme, S.; Bannwarth, C.; Shushkov, P. A Robust and Accurate Tight-Binding Quantum Chemical Method for Structures, Vibrational Frequencies, and Noncovalent Interactions of Large Molecular Systems Parametrized for All spd-Block Elements (Z = 1–86). *J. Chem. Theory Comput.* **2017**, *13*, 1989–2009.
18. Baldinelli, L.; Angelis, F. D.; Bistoni, G. Unraveling Atomic Contributions to the London Dispersion Energy: Insights into Molecular Recognition and Reactivity. *J. Chem. Theory Comput.* **2024**, *20*, 1923–1931.
19. Regni, G.; Baldinelli, L.; Bistoni, G. A Quantum Chemical Method for Dissecting London Dispersion Energy into Atomic Building Blocks. *ACS Cent. Sci.* **2025**, *11*, 890–898.
20. Neese, F. The ORCA Program System. *WIREs Comput. Mol. Sci.* **2012**, *2*, 73–78.
21. Becke, A. D. Density-Functional Thermochemistry. III. The Role of Exact Exchange. *J. Chem. Phys.* **1993**, *98*, 5648–5652.
22. Lee, C.; Yang, W.; Parr, R. G. Development of the Colle-Salvetti Correlation-Energy Formula into a Functional of the Electron Density. *Phys. Rev. B* **1988**, *37*, 785–789.
23. Weigend, F. Accurate Coulomb-Fitting Basis Sets for H to Rn. *Phys. Chem. Chem. Phys.* **2006**, *8*, 1057–1065.
24. Valeev, E. F. Libint: A Library for the Evaluation of Molecular Integrals of Many-Body Operators over Gaussian Functions, *http://libint.valeyev.net/*.
25. Lu, T.; Chen, F. Multiwfn: A Multifunctional Wavefunction Analyzer. *J. Comput. Chem.* **2012**, *33*, 580–592.
26. Xtb, Version 6.3; University Bonn: **2020**; please refer to *xtb@thch.uni-bonn.de*.
27. T. Lu, molclus program, *http://www.keinsci.com/research/molclus.html*.
